# Sex-specific interneuron vulnerability after traumatic brain injury correlates with neurotrophic signaling and chloride homeostasis, independent of behavioral and network outcomes

**DOI:** 10.3389/fncel.2025.1572213

**Published:** 2025-09-24

**Authors:** Amandine Consumi, Cécile Marcourt, Tayam Tnaimou, Jérôme Laurin, Michael K. E. Schäfer, Christophe Pellegrino, Claudio Rivera

**Affiliations:** 1 Aix Marseille Université, INSERM, INMED, Marseille, France; 2 Aix Marseille Université, CNRS, ISM, Marseille, France; 3 Department of Anaesthesiology, University Medical Center Mainz, Mainz, Germany; 4 Neuroscience Center, HiLife, University of Helsinki, Helsinki, Finland

**Keywords:** trauma, cognitive performance, trophic factor, sex dependence, GABA transmission

## Abstract

Traumatic brain injury (TBI) leads to persistent cognitive and emotional impairments, and growing evidence suggests that sex influences vulnerability through differences in neurotrophic signaling and chloride homeostasis. To investigate these mechanisms, we induced moderate TBI in male and female mice using the controlled cortical impact model and assessed outcomes 30 days post-injury. Behavioral performance was evaluated with the open field, elevated plus maze, and Barnes maze, while hippocampal oscillations, interneuron survival, protein expression (KCC2, NKCC1, p75^NTR^, BDNF), and transcriptomic profiles were analyzed. Locomotor activity was unaffected by TBI. Both sexes showed reduced latency to anxiogenic zones, but only females spent more time in the open arms, suggesting disinhibition. In the Barnes maze, both sexes exhibited spatial memory deficits: females showed early impairments with recovery, while males displayed persistent deficits. Electrophysiological recordings revealed increased theta and alpha power in both sexes, with greater variability in females. PV+ interneurons were selectively reduced in female hippocampi, accompanied by p75^NTR^ upregulation, whereas males exhibited decreased BDNF. Transcriptomic analysis identified female-specific enrichment of calcium signaling, inflammation, and neurogenesis pathways, and NKCC1 upregulation occurred only in females. These findings reveal sex-specific interneuron vulnerability and molecular alterations after TBI, independent of overt behavioral and network outcomes, suggesting distinct mechanistic pathways that converge on similar functional phenotypes and underscoring the importance of sex-informed therapeutic strategies.

## Introduction

Traumatic brain injury (TBI) remains a major global health concern and is among the leading causes of mortality and long-term disability, particularly in industrialized nations (Ng and Lee, 2019; Bondi et al., 2015). Annually, an estimated 50 million individuals experience TBI, with many suffering lasting cognitive, emotional, and neurological impairments. These chronic sequelae are influenced by multiple factors, including injury severity, brain region vulnerability, and the degree of secondary pathophysiological cascades that follow the initial insult (Rabelo et al., 2024; Goubert et al., 2019).

TBI encompasses both primary damage, occurring at the moment of impact, and secondary injuries that evolve over days to months, contributing to long-term complications such as post-traumatic depression (PTD), anxiety-related disorders, and post-traumatic epilepsy (PTE) (Alway et al., 2016; Guekht et al., 2010; Kourdougli et al., 2017; Pease et al., 2024). To model these chronic outcomes, we and others have employed the controlled cortical impact (CCI) model of TBI in mice, a widely accepted paradigm that replicates moderate to severe brain injury and results in persistent behavioral, molecular, and neurophysiological alterations, including PTD-like phenotypes observed 30 days post-injury (Goubert et al., 2019; Tessier et al., 2023; Osier and Dixon, 2016).

Among brain structures, the hippocampus—a region integral to learning, memory, and mood regulation—is particularly vulnerable to TBI. Within the hippocampus, the dentate gyrus (DG) shows pronounced alterations in inhibitory GABAergic signaling, often linked to increased network excitability and cognitive dysfunction (Santhakumar et al., 2001). One critical mechanism involves dysregulation of chloride homeostasis, mediated by the cation-chloride co-transporters, the Na-K-2Cl cotransporter 1 or NKCC1 (the chloride importer) and the chloride extruder K-Cl cotransporter 2 (KCC2). Following TBI, KCC2 internalization and decreased function have been observed, leading to a reversion to immature depolarizing GABA responses and increased pro-apoptotic signaling in male mice (Tessier et al., 2021, 2023; Witkin et al., 2024).

Parvalbumin-positive (PV+) interneurons represent a major subclass of fast-spiking GABAergic interneurons that play a critical role in maintaining excitatory-inhibitory balance within cortical and hippocampal circuits. In the hippocampus, PV + interneurons, including basket and axo-axonic cells, are essential for the generation and synchronization of network oscillations—particularly in the theta (4–12 Hz) and gamma (30–100 Hz) frequency bands—which are crucial for spatial navigation, working memory, and sensorimotor integration (Bartos et al., 2007; Sohal et al., 2009). By precisely timing the output of principal cells, PV + interneurons contribute to the temporal coordination of neuronal ensembles during cognitive tasks. These interneurons are also particularly vulnerable to pathophysiological insults, including TBI, which can result in selective interneuron loss, disruption of inhibitory tone, and alterations in hippocampal oscillatory dynamics (Toth et al., 1997; Huusko et al., 2015; Ruden et al., 2021; Ruden et al., 2021). Given their central role in both network stability and cognitive processing, PV + interneurons are increasingly recognized as key players in post-traumatic circuit dysfunction, and their selective vulnerability may contribute to the emergence of long-term cognitive and behavioral impairments.

Despite growing insights into TBI pathophysiology, most studies to date have focused predominantly on male rodents, limiting our understanding of how females respond to injury. Yet there is substantial evidence for sex-dependent regulation of GABAergic development, particularly during early postnatal stages. Females typically exhibit earlier expression of KCC2 and an accelerated transition to hyperpolarizing GABA action—a process known as the “GABA shift” (Rivera et al., 1999; Galanopoulou, 2008a; Perrot-Sinal et al., 2007). Such developmental differences may confer differential vulnerability or resilience to TBI and could underlie the sex-specific trajectories of recovery observed in clinical and preclinical studies (Levin et al., 2021; Tucker et al., 2016; Wang et al., 2022).

Further complicating these outcomes is the role of neurotrophins, such as BDNF and its receptor p75^NTR^, which are critical regulators of neuronal survival and plasticity after injury. Both neurotrophin signaling and neural oscillatory dynamics—particularly theta and alpha rhythms—may exhibit sex-specific patterns post-TBI and contribute to cognitive and emotional disturbances (Lin et al., 2021; Fedor et al., 2010).

Given these multidimensional influences, the present study seeks to comprehensively characterize sex-specific behavioral, electrophysiological, and molecular alterations in a chronic (30 days post-injury) mouse model of moderate TBI using CCI. We particularly focus on mechanisms related to GABAergic dysfunction, interneuron vulnerability, chloride homeostasis, neurotrophin signaling, and transcriptomic profiles. Understanding how these processes diverge between sexes is crucial for developing targeted, sex-informed therapeutic strategies for TBI.

## Materials and methods

### Animal experiments

Ninety-one (males and females) C57BL/6J mice (10-weeks old) were housed in an enriched environment at INMED animal facility, maintained in a 12 h light / 12 h dark cycle environment with controlled temperature (23 ± 2°C), food and water were given ad libitum. All experiments were conducted in accordance with the French ethical committee which approved all procedures (No APAFiS#38335). All animal experiments complied with the ARRIVE guidelines and were carried out in accordance with the U. K. Animals (Scientific Procedures) Act, 1986 and associated guidelines, EU Directive 2010/63/EU for animal experiments. All methods were performed following the relevant guidelines and regulations.

Animals were excluded when one of the following points was observed: clinical signs of pain and discomfort: prostrated animals with absence of movement and/or hunched posture, ungroomed appearance, weight loss higher than 20%, dehydration, decreased urine/fecal output, piloerection, chronic porphyrin staining around eyes, nose or forelimbs, and rapid respirations and absence of social interaction in the cage and decrease of food and/or water intake. Mice were randomly assigned to their respective experimental group.

### Controlled-cortical impact model (CCI)

The controlled cortical impact (CCI) procedure was carried out in an aseptic environment. Buprenorphine (0.03 mg/kg) was injected subcutaneously (s.c) 30 min before surgery. Mice were then anaesthetized using 4% isoflurane mixed with air and enriched with oxygen (0.3%) before being positioned in a stereotaxic frame (David Kopf Instruments). Body temperature was monitored throughout the procedure using a rectal probe and maintained at 37 ± 2°C with a heating pad (Harvard Apparatus®). A unilateral craniotomy was performed over the right parietal cortex while leaving the dura intact, using a high-speed drill. CCI was performed using a Leica impactor using the following parameters: tip diameter 3 mm, 6 m/s speed, 1.5 mm depth and 200 msec duration. Animals were allowed to recover on the heating pad before their transfer to their home cages.

### Immunohistochemistry

Mice were deeply anaesthetized with isoflurane (3–4% for induction, 1–2% for maintenance) delivered via a vaporizer and nose cone. Transcardiac perfusion was then performed using a 3% paraformaldehyde solution (AntigenFix, Diapath). Brains were post-fixed overnight in 3% paraformaldehyde at 4°C and then coronally sliced with a Leica VT1200S Vibratome. Immunochemistry was performed on free floating slices (60 μm) permeabilized and blocked in PBS with 0.3% Triton X-100 and 5% normal goat serum (NGS) for 1 h at room temperature, stained with primary antibodies diluted in PBS with 5% NGS and 0.1% Triton X-100 at 4°C overnight anti-PV antibodies (Synaptic System, 195,004). After washing using PBS, slices were incubated with the corresponding Alexa Fluor 488 and 555-conjugated secondary antibodies diluted in PBS (1:500), (Thermo Fisher Scientific, Invitrogen A11001) for 2 h at room temperature. Sections were mounted onto Superfrost Plus glass slides in Fluoromount G mounting medium. For each section, serial images were taken using a fluorescence microscope (LSM800 Confocal, Zeiss) equipped with an apotome module with 5 × or 10 × objectives.

### Western blot analysis

Animals were killed by decapitation after deep isoflurane anesthesia. Hippocampi was quickly dissected out and flash-frozen in liquid nitrogen and stored at −80°C. Acquired samples were homogenized in RIPA buffer (50 mM Tris–HCl pH 8; 150 mM NaCl; SDS 0.1%; Deoxycholic Acid 0.5%; 1% Triton X-100) supplemented with Protease/Phosphatase Inhibitor Tablet (ThermoFisher®). Proteins were run on polyacrylamide gel (Bolt 4–12% Bis Tris plus, Invitrogen by ThermoFisher® Scientific) and transferred to a nitrocellulose membrane (GE Healthcare Life Science) for 7 min. After blocking in Tris-buffered saline/ 0.1% Tween/ 5% bovine serum albumin (BSA), membranes were exposed overnight at 4°C to primary antibodies diluted in blocking solution (Tris-buffered saline/ 0.1% tween/ 2.5% BSA). The following primary antibodies were used: Anti-KCC2 (home-made antibody, 1:3000; Ludwig et al., 2003), anti-NKCC1 (DSHB, #528406, 1:2000) and anti-P75^NTR^ (Santa Cruz, Sc-53631, 1:1000). Horse radish peroxidase-conjugated anti-rabbit or anti-mouse IgG (Agilent Dako) were used as secondary antibodies, diluted in 5% BSA at room temperature for 2 h. Finally, bands were detected using Super Signal West Pico (ThermoFisher® Scientific, #34080) using the image analysis software G box (Syngene). NKCC1 and p75^NTR^ signals were normalized with α-Tubulin (Invitrogen, #62204, 1:10000) while KCC2 signal was normalized with β3-Tubulin (Biolegend, #802001, 1:10000). Quantifications were performed using Gel Plot Analyzer plugin on Image J.

### Mature brain-derived neurotrophic factor BDNF (mBDNF) immunoassay (ELISA)

All samples, i.e., standards, controls, blanks and test animals were performed in duplicate. Quantification of mBDNF standard and hippocampal samples was, respectively, performed with mBDNF Rapid ELISA Kit (Biosensis®, BEK-2211-1P/2P—sandwich ELISA—Thebarton, SA, Australia) in the concentrated solutions following the manufacturer’s protocol. The quality control sample ranged between 175 and 325 pg.ml^−1^ and the standard ranged between 7.8–500 pg.ml^−1^ for the mBDNF. After having detected mBDNF with streptavidin-HRP conjugate, the concentration mBDNF were allowed by the addition of a substrate (3,3′, 5,5′-tetramethylbenzidine, TMB). The standard curve was plotted allowing the interpolation of the mBDNF protein concentrations of the sample through a 4-PL regression analysis. Concentrations were determined using the FLUOstar® OPTIMA microplate reader (BMG Labtech, France).

### Behavioral testing

One month following CCI, mice were tested on different tasks assessing locomotor activity as well as cognitive abilities and anxiety. For all behavioral assessments, animals were left for habituation to the behavioral room for one hour prior the tests.

#### Anxiety-like behavior

Mice were first tested for locomotor activity and anxiety-like behavior using the open field test (OFT) and elevated plus maze (EPM). For the OFT, animals are placed in a squared arena and allowed to freely explore for 20 min. Distance traveled, time spent in borders and center as well as frequency to visit each zone during the first 4 min was assessed. The EPM consist of an elevated plus-shaped arena containing 2 open arms and 2 closed arms where animals are placed facing an open arm then allowed to freely explore for 5 min. We measured the distance traveled as well as the time spent in each arm and center of the arena (Kraeuter et al., 2019).

#### Memory and learning abilities

**Figure 1:**
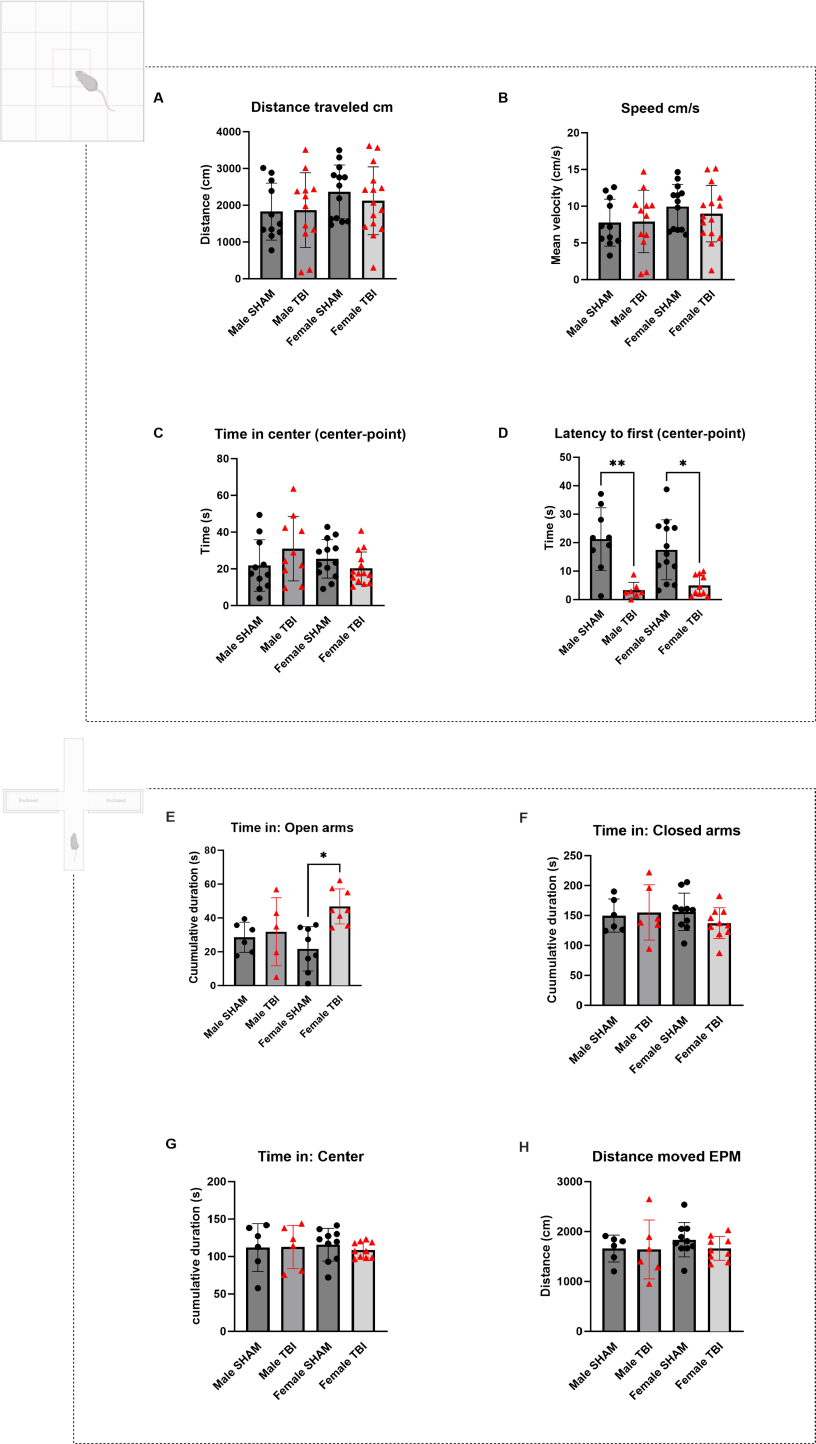
Open field and elevated plus maze test of CCI animals. **(A–D)** Open Field test (OFT). Animals were in the arena for 10 min. **(A)** Distance traveled in the arena expressed in centimeters (cm). **(B)** Average speed of the animals in the arena expressed in cm/s. **(C)** Cumulative time spent in the center of the arena (s). **(D)** Latency to the first entry in the center zone in seconds (s). **(E-H)** Elevated Plus Maze (EPM). Animals were in the arena for 5 min and *n* = 6 per group. **(E)** Cumulative time spent in the open arms of the arena (s). **(F)** Cumulative time spent in the closed arms of the arena (s). **(G)** Cumulative time spent in the center of the arena (s). **(H)** Distance traveled in the arena (cm). Data were analyzed using Two-way ANOVA with post-hock Tukey’s multiple comparisons test. Significance was determined as p* < 0.05; p** < 0.01; p*** < 0.001.

Mice were also tested in the Barnes maze test to assess spatial memory and cognitive flexibility impairments. During training phase 1 (Days 1–5, D1–D5), mice were trained to locate the escape box in a fixed location within the maze. On probe trial 1 (PTD 1), conducted on D6, the escape box was replaced with a false escape to assess memory retention of the previously learned location, corresponding to the probe trial day (PTD). In training phase 2 (D6–D10), mice were trained to locate a new escape box in a different location within the maze. Probe trial 2 (PTD 2) on D11 assessed memory retention of the new escape location (Figure 1A).

Primary latency to find the escape box was measured throughout the experiment as an indicator of learning and memory performance. To assess the impact of sex and traumatic brain injury (TBI) on learning performance in the Barnes maze, we fitted a linear mixed-effects model with Latency as the dependent variable, Training Day, Condition (SHAM vs. TBI), and Sex (Male vs. Female) as fixed effects, and Subject as a random effect. The model included all two-way and three-way interactions.

Mice were trained twice daily for 5 days then tested on day 6 (probe trial day or PTD). Each training consists in a 3 min trial where the animals are freely navigating the platform to find the escape box. If an animal failed to find the escape box within the three-minute delay, it was gently guided to the target hole. In-between each trial, the entire maze, false escapes and target box are cleaned using a solution of 70% ethanol then dried. A one-hour delay separates the 2 training sessions each day. During the second week of training, the target hole is moved on the opposite side of the Barnes’ maze allowing us to assess cognitive flexibility.

On PTD, the target box was replaced with a false escape. Distance traveled, and time to reach the target hole was measured. Recordings and analyses were performed using Ethovision software (Noldus).

### Headplates surgeries

For *in vivo* intrahippocampal multi-site recordings, one-month-old male and female mice (C57BL/6J) were anesthetized using isoflurane (4% for induction and stabilized at 2/2.5%) mixed with air and enriched with oxygen (0.3%) then positioned in a stereotaxic frame (David Kopf Instruments®). Body temperature was monitored throughout the procedure using a rectal probe and maintained at 37 ± 2°C with a heating pad (Harvard Apparatus®). Animals received subcutaneous injections of buprenorphine (0.03 mg/kg) and carprofen (5 mg/mL). A metallic head-plate was attached to the skull using dental cement (Opti-Bond, Kerr) and the recording coordinates (2 mm posterior to and 1.5 mm left of the bregma and 1 mm right of the bregma), in the parietal cortex marked using an indelible marker. Animals were monitored during the following week to make sure the recovery was successful. After a 72 h rest, animals were trained in the Mobile HomeCage® (MHC V5, Neurotar®) for the next 3 days. Two days prior to recording, animals were anesthetized (isoflurane 4% for induction and stabilized at 2/2.5%) and received subcutaneous injections of buprenorphine (0.03 mg/kg) and carprofen (5 mg/mL). Two craniotomies were performed using a 1 mm diameter drill and the recording sites protected using a silicon cast (Kwik-Cast™).

### *In vivo* electrophysiological recordings

Multisite intrahippocampal recordings were performed by implanting a one-shank 16-channel linear probe (A1 × 163 mm-100–177, Neuronexus®) through a 1 mm diameter craniotomy at the posterior parietal level (2 mm posterior, 1.5 mm left of the bregma and 1 mm ventral) on head-restrained animals trained on a Mobile HomeCage (MHC V5, Neurotar®). One month following TBI, animals were recorded twice each recording lasting 10 min. Recordings were acquired using Allego software (SmartBox Pro™, Neuronexus®) and a sampling rate of 30 K Hz per channel.

### Bulk mRNA sequencing and differential gene expression analysis

Bulk mRNA sequencing (RNA-seq) was conducted to profile transcriptomic changes across experimental conditions essentially as previously described (Wang et al., 2022). Briefly, total RNA was extracted from frozen tissue samples using RNAeasy Kit (Qiagen), the quantity was assessed with the Qubit 2.0 and RNA integrity was checked using a RNA 6000 Nano chip on Agilent’s bioanalyzer.

The preparation of barcoded mRNA seq libraries followed an established protocol, using a NEBNext® Poly(A) mRNA Magnetic Isolation Module and NEBNext® Ultra™ II RNA Library Prep Kit designed for Illumina® systems with a final amplification of 12 cycles. Library concentrations were quantified using Invitrogen’s Qubit HS DNA assay and average library size was evaluated using Agilent’s 2,100 Bioanalyzer in conjunction with a HS DNA chip (Wang et al., 2022).

Sequencing was conducted at Novogene (Cambridge, UK) utilizing an Illumina NovaSeq 6,000 sequencing system with a sequencing depth of 30 Mio paired end (150 cycles) reads per sample. Sample quality was evaluated using demultiplexed fastq.gz files. The sequenced reads were trimmed for adapter sequences and processed employing Qiagen’s software CLC Genomics workbench (v21.0.5) with CLC’s default settings for RNASeq analysis. Reads were aligned to the GRCm38 genome (Mouse Genome Sequencing Consortium et al., 2002) employing a minimum read length of 50 bp. The expression value unit is TPM. Results were displayed with ArrayStar 17 (Lasergene) which provided information on the number of mapped reads, target length, source length and position, strand, genes, and gene IDs, annotated according to the mm10 assembly.

Genes were filtered to include only those with at least 7 valid values out of 20 samples and normalized reads > 0.1, thereby excluding low expression genes. The RNAseq data have been deposited as a GEO dataset with the accession number GSE196121: https://www.ncbi.nlm.nih.gov/geo/query/acc.cgi?acc=GSE196121

Differential gene expression (DGE) analysis was performed using the web-based platform iDEP2. The following steps were carried out within iDEP2: (i) Read count data were normalized using DESeq2 normalization to account for variations in sequencing depth and composition. (ii) Differentially expressed genes (DEGs) were identified using DESeq2, with a significance threshold set at a false discovery rate (FDR) of 0.05 except in the case of PV enriched genes that were higher values were allowed. (iii) Data visualization, including heatmaps, principal component analysis (PCA) plots (Supplementary Figure 1), and volcano plots, was used to summarize the expression patterns and statistical results.

### Statistical analysis

All mean values are given with the standard deviation (SD). Normality of each distribution was assessed using a significance level of 5%. Depending on the experimental design, either a two-way ANOVA or a mixed-effects model (for time-dependent variables) was applied using Prism software (GraphPad Software, Inc., La Jolla, CA, United States). The results were further corroborated using Python-based statistical analysis. Significance is set as follows: ∗*p* < 0.05; ∗∗*p* < 0.01; ∗∗∗*p* < 0.001.

## Results

### Mice display sex-specific responses in anxiogenic environment after TBI

#### Locomotor performance

We started by assessing locomotor performance using the open field test (OFT). Parameters included the total distance traveled and average speed of movement during the test. No significant differences were observed between SHAM and TBI groups in either males or females (Distance traveled (cm): Males: SHAM = 1827 ± 775, TBI = 1868 ± 1,018; Females: SHAM = 2,365 ± 202, TBI = 2,122 ± 239. Speed (cm/s): Males: SHAM = 7.7 ± 1.0, TBI = 7.9 ± 1.2, Females: SHAM = 10.0 ± 0.8, TBI = 9.0 ± 1.0, Figures 1A,B *n* = 11,12,13,15 respectively). Statistical analyses using two-way ANOVA confirmed no significant differences across injury status or sex for these measures [*F*_(3,32)_ = 0.846, *p* = 0.48 and *F*_(3,32)_ = 0.81, *p* = 0.49 respectively; *n* = 15]. These findings indicate that one month after insult, locomotor abilities were unaffected by TBI.

#### Disinhibition like behavior in the OFT and EPM

We analyzed the time spent in the center of the arena versus the borders, as well as the latency to the first center entry [Time in the center (s): Males: SHAM = 21.8 ± 14.0, TBI = 31.0 ± 17.5; Females: SHAM = 25.4 ± 10.5, TBI = 20.3 ± 8.9; *n* = 11, 10, 13, 14, respectively; Two-way Anova: *F*_(3,29)_ = 2.2, *p* = 0.112; *n* = 15 Figure 1C]. No significant differences were found between sex and TBI effect. In contrast, latency to first entry showed significant differences for both male and female between SHAM and TBI [Latency to first entry (s): Males: SHAM = 21.3 ± 3.7, TBI = 3.3 ± 1.0, *p* = 0.007; Females: SHAM = 17.3 ± 2.9, TBI = 5.0 ± 1.1, *p* = 0.026; Two-way Anova *F*_(3,20)_ = 7.5; *p* = 0.0015, *n* = 15 Figure 1D**]**. Although no significant differences were observed in the time spent in the center of the open field across sex or injury status, TBI significantly reduced the latency to first center entry in both male and female mice, indicating a facilitated approach to the anxiogenic center zone. This suggests that TBI may alter early exploratory behavior or reduce initial anxiety-like responses, independent of sex. However, the absence of differences in total center time suggests that overall anxiety-like behavior remains largely unaffected.

To further assess anxiety-like behaviors, we utilized the EPM, measuring the time spent in the open arms (OA), center, and closed arms. This test provides insight into exploratory and risk-taking behavior, with increased time in the open arms generally indicating lower anxiety and or increased risk taking [time spent in the open arms (s): Male SHAM: 28.5 ± 3.6, Male TBI: 31.8 ± 9.0, *p* > 0.999; Female SHAM: 21.7 ± 4.6, Female TBI: 46.8 ± 3.7, *p* = 0.014; Two-way Anova: *F*_(3,15)_ = 4.941, *p* = 0.0139], Male *n* = 6, Female *n* = 10; Figure 1E). Female TBI mice tended to spend more time in the open arms compared to SHAM females. This suggests that female TBI mice may exhibit more exploratory or impulsive behavior than males, potentially reflecting a reduced inhibition in response to the stressful environment of the EPM (Pawlak et al., 2012). There were no significant differences between any of the groups in the time spent in the closed arms of the arena or in the center [Time spent in the closed arms (s): Male SHAM: 149.9 ± 11.3; Male TBI: 155.2 ± 18.9; Female SHAM: 156.1 ± 9.8; Female TBI: 137.3 ± 8.1; Two-way Anova: *F*_(3,19)_ = 0.863, *p* = 0.477; Male *n* = 6, Female *n* = 10, Figure 1F]. Time spent in the center (s): [Male SHAM: 111.9 ± 13.0; Male TBI: 112.8 ± 11.7; Female SHAM: 115.8 ± 6.9; Female TBI: 108.8 ± 3.3; Two-way Anova: *F*_(3,19)_ = 0.143, *p* = 0.933; Male *n* = 6, Female *n* = 10, Figure 1G]. Distance moved (cm): [Male SHAM: 1661 ± 110; Male TBI: 1641 ± 240; Female SHAM: 1837 ± 109; Female TBI: 1665 ± 78; Two-way Anova: *F*_(3,18)_ = 0.695, *p* = 0.567; Male *n* = 6, Female *n* = 10, Figure 1H].

In the EPM test, female TBI mice spent significantly more time in the open arms compared to female SHAM controls, suggesting reduced anxiety-like behavior or increased exploratory/risk-taking tendencies following TBI. No significant differences were observed in male mice, indicating a sex-specific behavioral response to injury. Time spent in the closed arms, center zone, and total distance moved did not differ significantly between groups, ruling out general locomotor alterations as a confounding factor. These findings point to a TBI-induced disinhibition or altered threat evaluation in females, potentially consistent with increased impulsivity or impaired risk assessment (Pawlak et al., 2012).

### Male and female mice express specific cognitive impairments after TBI

**Figure 2:**
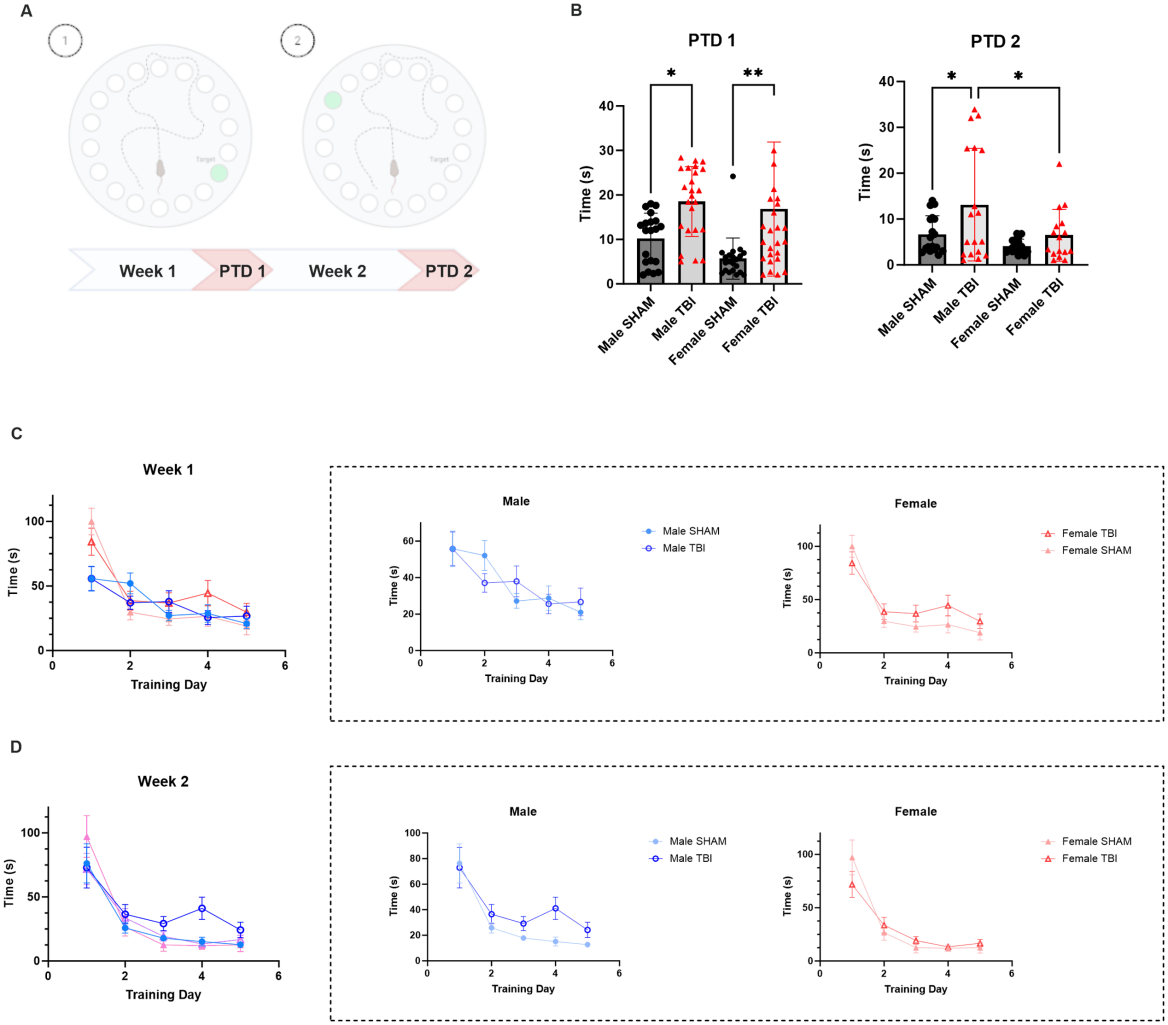
Effect of TBI on spatial memory and cognitive flexibility. **(A)** Schematic representation of the experimental protocol. **(B)** Primary latency to reach the target box on Probe Trial Day (PTD) 1 and 2. **(C)** Learning curve during training week 1 (D1-D5) **(D)** Learning curve during training week 2 (D6-D10). Data were analyzed using Two-way ANOVA with post-hock Tukey’s multiple comparisons test (B) and linear mixed-effects model **(C,D)**. Significance was determined as p* < 0.05; p** < 0.01; p*** < 0.001.

TBI frequently results in deficits in spatial learning and memory with emerging evidence suggesting that females and males may respond differently to brain injury due to differences in hormonal regulation, neuroinflammation, and neuroplasticity (Gölz et al., 2019). To investigate these sex-specific effects, we utilized the Barnes maze task (BMT), a well-established test of spatial learning and memory, to assess spatial cognitive learning and memory performance abilities and cognitive flexibility in male and female mice following TBI (Gawel et al., 2019; Rodríguez Peris et al., 2024). The BMT consisted of two training phases and two probe trials (Figure 2A).

Primary latency to find the escape box was measured throughout the experiment as an indicator of learning and memory performance. To assess the impact of sex and traumatic brain injury (TBI) on learning performance in the Barnes maze, we fitted a linear mixed-effects model with Latency as the dependent variable, Training Day, Condition (Sham vs. TBI), and Sex (Male vs. Female) as fixed effects, and Subject as a random effect. The model included all two-way and three-way interactions.

For the first training week, the analysis revealed a significant main effect of Training Day (*p* < 0.001), indicating that animals across all groups improved performance over time. There was also a significant main effect of Sex (*p* < 0.001), with males displaying lower latencies compared to females (Figure 2C). The main effect of Condition (SHAM vs. TBI) was not statistically significant (*p* = 0.18), although a significant interaction between Training Day and Condition (*p* = 0.032) indicated that the learning trajectories differed between TBI and SHAM animals. Additionally, a significant Training Day × Sex interaction (*p* = 0.002) suggested that learning progression varied between males and females. The three-way interaction among Training Day, Condition, and Sex was not significant (*p* = 0.366), indicating no evidence that the effect of TBI on learning differed by sex (Figure 2C). Ultimately, on PTD1, primary latency was significantly delayed in both male and Female TBI mice [Male SHAM: 10.2 ± 1.3 s vs. TBI: 18.5 ± 1.6 s, *p* = 0.013; Female SHAM: 5.7 ± 1.0 s vs. TBI: 16.8 ± 2.9 s, *p* = 0.004; Two-way Anova: *F*_(3,62)_ = 9.438, *p* < 0.001; *n* = 24, Figure 2B]. Overall, these results indicate that while female TBI mice initially required more time to acquire the task, they ultimately achieved comparable levels of spatial learning and memory retention to non-injured males. TBI disrupted the stability of learning in in both male and female mice, with female mice displaying more significant effects.

On the other hand, during the second training phase the model revealed a significant main effect of Training Day (*p* < 0.001), indicating that latency decreased over time, consistent with learning. There was a trend toward a main effect of Sex (*p* = 0.075), suggesting that males may have slightly lower latencies compared to females, though this did not reach statistical significance. The main effect of Condition (TBI vs. SHAM) was also not significant (*p* = 0.163). Interaction effects, including Training Day × Condition (*p* = 0.120), Training Day × Sex (*p* = 0.129), and the three-way interaction between Training Day, Condition, and Sex (*p* = 0.999) were not statistically significant (Figure 2D). This suggests that, although all groups demonstrated learning across days, there were no robust differences in learning rates between SHAM and TBI groups or between sexes during this week. Interestingly, Male TBI performance during the PTD2 display sensitivity as they tend to take more time to find the escape box compared to SHAM (SHAM: 6.7 ± 1.0 s vs. TBI 13.1 ± 2.9, *p* = 0.019, Figure 2B). Unexpectedly, the performance of female TBI mice did not differ significantly from SHAM on PTD2 [SHAM: 4.0 ± 0.4 s vs. TBI: 6.5 ± 1.4 s, *p* > 0.99; Two-way Anova: *F*_(3,44)_ = 5.374, *p* = 0.003; *n* = 24, Figure 2B].

These results may indicate that TBI disrupts early memory stability in both sexes, but females may exhibit greater initial impairment followed by better recovery over time in cognitive flexibility.

### *In vivo* electrophysiology: hippocampal oscillations are altered in both female and male after TBI

**Figure 3:**
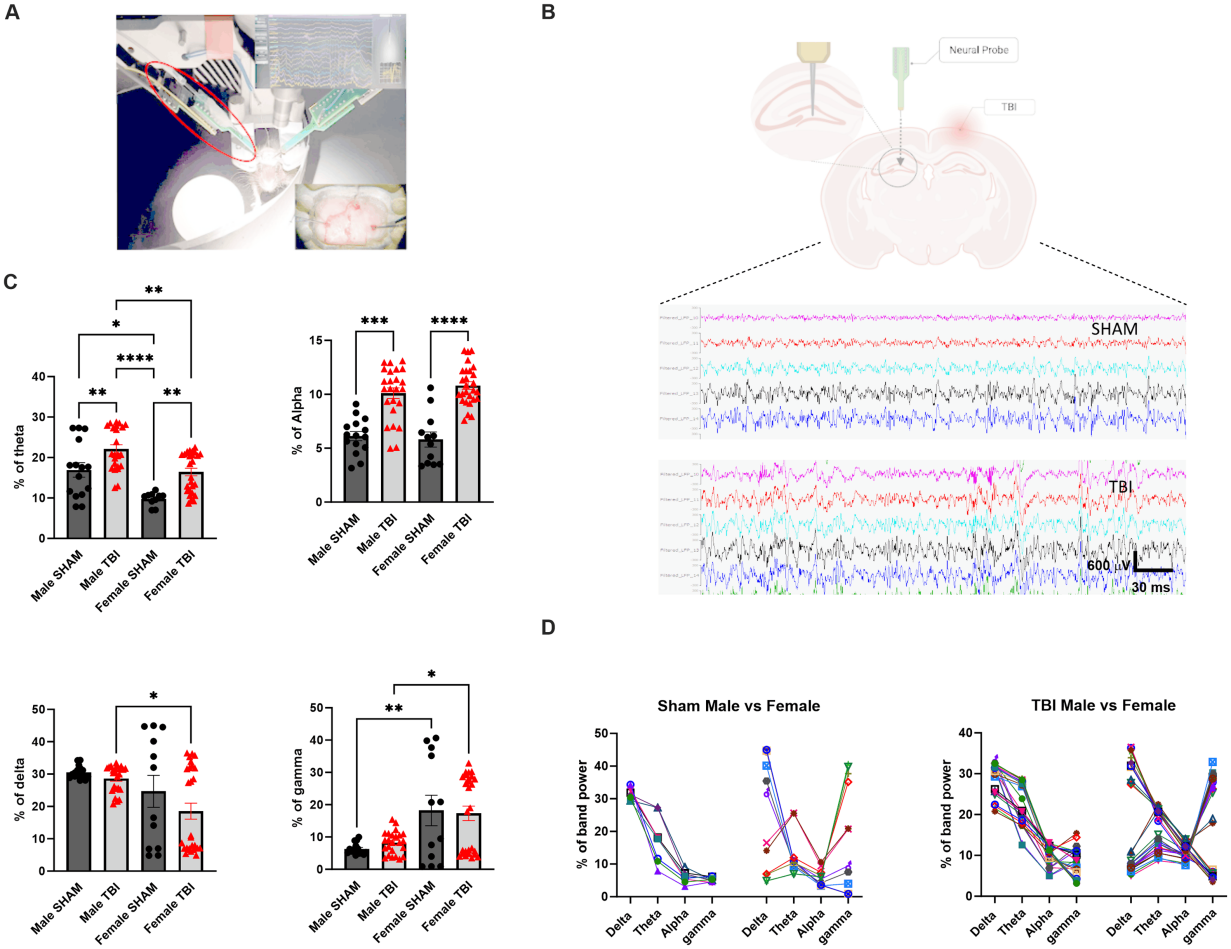
Impact of TBI on brain rhythms. **(A)** Picture of experimental set-up with the animal inside the arena. **(B)** Representative picture of the recording electrode placement on the contralateral side to the lesion and 2 examples of recordings for SHAM and TBI. **(C)** Percentage of different brain rhythms during recording. Brain waves analyzed were specifically: Delta (1–4 Hz), Theta (5–8 Hz), Alpha (9–12 Hz) and Gamma (13–80 Hz) **(D)** Analysis of the pattern of expression of the different brain oscillations depending on sex (males on the left, females on the right) and lesion. Data were analyzed using Two-way ANOVA with post-hock Tukey’s multiple comparisons test. Significance was determined as p* < 0.05; p** < 0.01; p*** < 0.001.

After studying the behavioral components following TBI in males and females, we sought to investigate potential differences at the network level. To achieve this, we performed acute electrophysiological recordings using a linear probe in the CA1 (Figures 3A,B). Specifically, we aimed to determine if brain oscillations were affected after TBI. Previous studies have shown that dysregulation of theta rhythm is associated with spatial memory impairments following TBI in males (Fedor et al., 2010), making it essential to evaluate this in our model. We analyzed the power spectra of the animals and found the following results. The contribution of theta power to the total spectra was significantly higher in males in general (*p* < 0.02, Figure 3C). Additionally, the theta band was altered in TBI compared to their SHAM counterparts for both females and males [female SHAM: 9.8 ± 0.5, *n* = 12 vs. female TBI: 16.5 ± 0.9, *n* = 27; *p* = 0.004; male SHAM: 16.9 ± 1.8, *n* = 15 vs. male TBI: 22.10 ± 1.1, *n* = 24; *p* = 0.002; Two-way Anova: *F*_(3,46)_ = 14.85, *p* < 0.0001; Figure 3C].

Next, we examined other bands to determine if these alterations affected brain rhythms differently. We measured alpha, gamma, and delta oscillations and found that only the alpha band displayed significantly increase in TBI animals [male SHAM: 6.1 ± 0.4%, vs. TBI: 10.1 ± 0.5%, *p* < 0.0001; female SHAM: 5.8 ± 0.7% vs. TBI: 10.8 ± 0.4%, *p* < 0.0001; Two-way Anova: *F*_(3,48)_ = 56.98, *p* < 0.0001, Figure 3C]. However, delta [male SHAM: 30.6 ± 0.5%, *n* = 15 vs. TBI: 28.7 ± 0.8%, *n* = 24, *p* > 0.99; female SHAM: 24.7 ± 4.9%, *n* = 12 vs. TBI: 18.5 ± 2.5%, *n* = 27; *p* > 0.99, Figure 3C] and gamma [male SHAM: 6.3 ± 0.4%, *n* = 15 vs. TBI: 8.3 ± 0.7%, *n* = 24, *p* > 0.99; female SHAM: 18.2 ± 4.7%, *n* = 12 vs. TBI: 17.3 ± 2.3%, *n* = 27, *p* > 0.99, Figure 3C] oscillations were not significantly altered after TBI. However, there were significant differences between female and male TBI [delta two-way Anova: *F*_(3,48)_ = 3.603, *p* = 0.019; gamma two-way Anova: *F*_(3,48)_ = 7.032, *p* = 0.0005]. These results indicate that TBI has specific impact on distinct brain waves patterns in the hippocampus in addition to pre-existing sex dependent differences.

To further explore this, we analyzed the patterns of brain oscillations in both genders. Strikingly, we noticed specific heterogeneity in females, while males displayed more uniform patterns (Figure 3D). These findings suggest a dichotomy between males and females in terms of brain rhythms but an unexpectedly no clear differences in sensitivity to TBI.

### Female PV interneurons display elevated sensitivity for TBI

**Figure 4:**
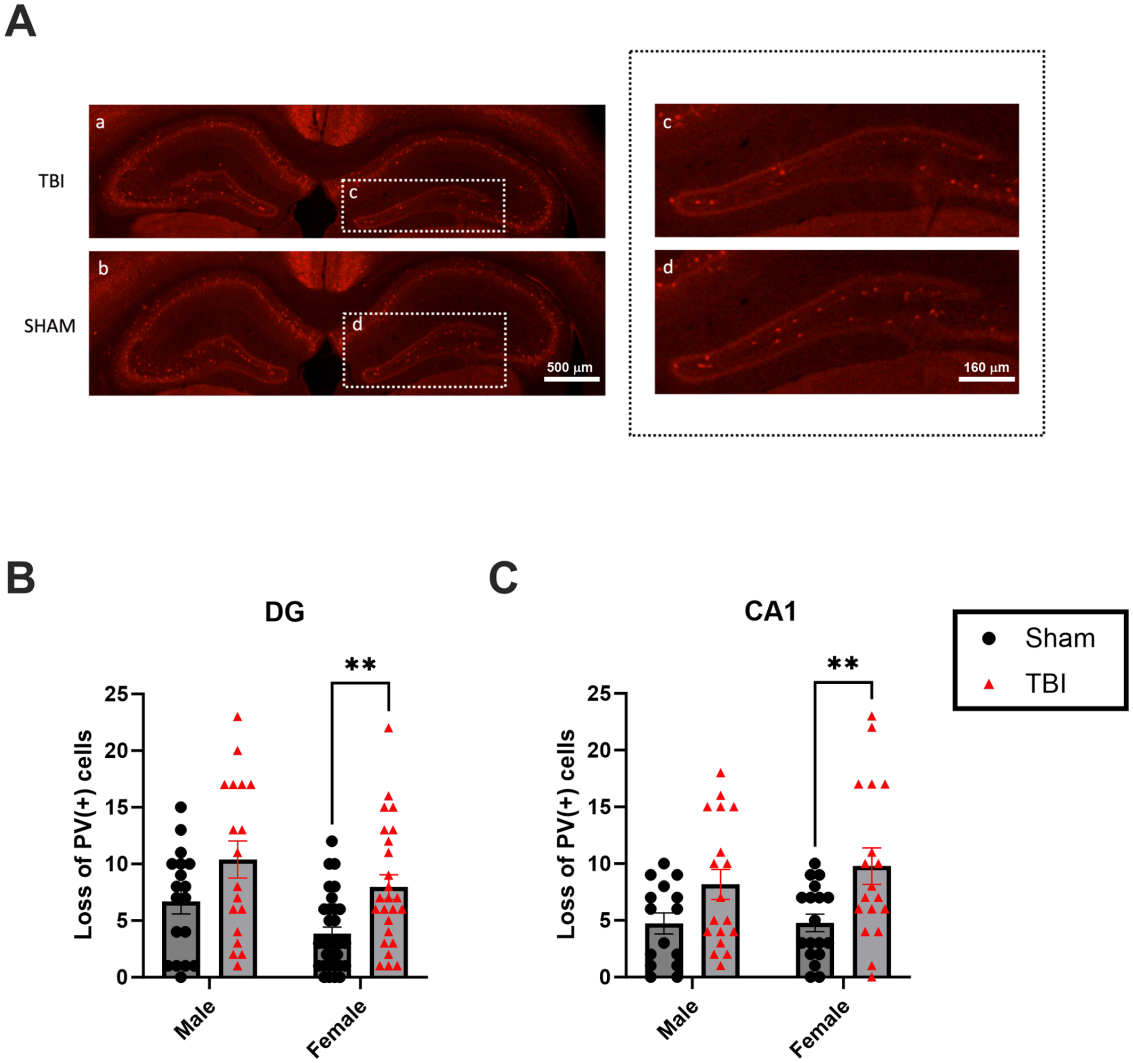
Effect of TBI on PV survival in the DG and CA1. **(A)** Picture of Parvalbumin (PV) immunostainings. **(A.a)** Representative picture of PV staining in SHAM **(A.b)** Representative picture of PV staining in TBI **(A.c)** Close up on the ipsilateral DG in SHAM with PV + cells **(A.d)** Close up on the ipsilateral DG in TBI with a loss of PV + cells. **(B)** Quantification of the loss of total PV + cells in DGs. Here, we calculated the absolute difference of PV + cells between the 2 hemispheres and is plotted as normalized on male SHAM. **(C)** Quantification of the loss of PV + cells in CA1. Results were calculated as above. Data were analyzed using Two-way ANOVA with post-hock Tukey’s multiple comparisons test. Significance was determined as p* < 0.05; p** < 0.01; p*** < 0.001. Calibration bar 500 μm **(Aa,b)** and 160 μm **(Ac,d)**.

In addition to examining cognitive performance and *in vivo* intrahippocampal oscillation, we also investigated the survival of parvalbumin-positive (PV+) interneurons in response to TBI. PV + interneurons play a crucial role in regulating neuronal network activity particularly known to play a key role in generating theta rhythms in the hippocampus (Freund and Antal, 1988) and maintaining balanced excitation and inhibition in the brain. Disruption of these interneurons is frequently associated with cognitive deficits and behavioral changes following TBI. In this section, we assess the survival and integrity of PV + interneurons (Figure 4A) in TBI mice, with a focus on potential sex differences in neuronal survival and their implications for TBI-induced cognitive impairments. Indeed, PV + interneurons are a specifically vulnerable subpopulation after TBI (Tessier et al., 2023). We quantified the loss of interneurons in the granular layer of the DG one month after TBI. Although both sexes presented changes upon TBI, only female loss of PV + interneurons in the DG reached a statistically significant level after TBI [Male SHAM: 6.7 ± 1.1, *n* = 18 vs. Male TBI: 10.4 ± 1.6, *n* = 18, *p* = 0.531; Female SHAM: 3.8 ± 0.8, *n* = 31 vs. Female TBI: 8.0.1 ± 1.1, *n* = 25, *p* = 0.028; Two-way Anova: *F*_(3,58)_ = 5.917; *p* = 0.0014, Figure 4B]. Similar results are found in the CA1 region [Male SHAM: 4.7 ± 0.9, *n* = 18 vs. Male TBI: 8.2 ± 1.3, *n* = 18, *p* = 0.179; Female SHAM: 4.8 ± 0.8, *n* = 18, vs. Female TBI: 9.8 ± 1.6, *n* = 18, *p* = 0.002, Figure 4C]. These results confirm that PV + interneurons are particularly vulnerable to TBI in the CA1 and DG of female mice.

### P75^NTR^ is upregulated in female CA1 after TBI

**Figure 5:**
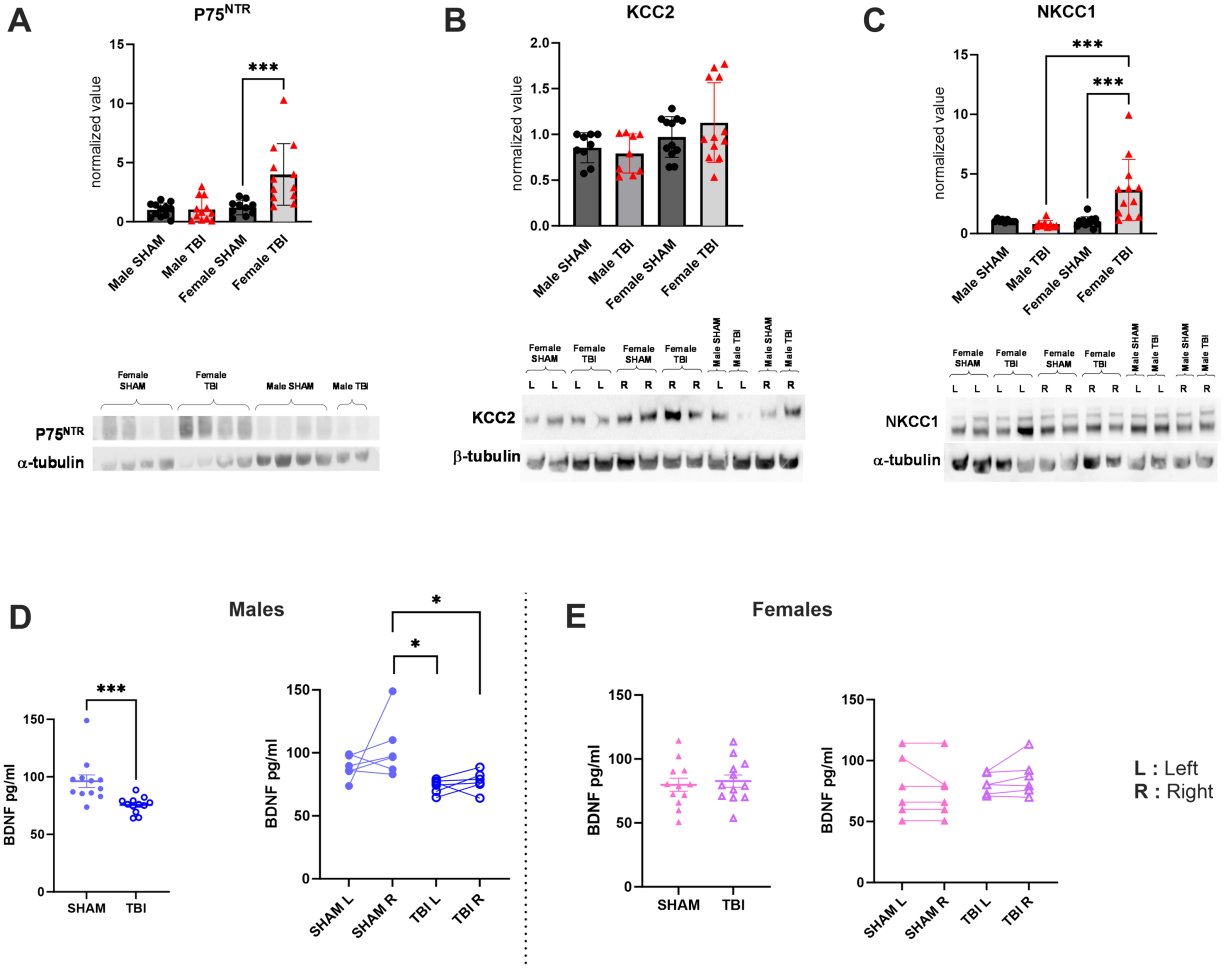
Effect of TBI on chloride co-transporters and trophic factors expression. **(A)** P75^NTR^ expression normalized to the marker α-tubulin. **(B)** KCC2 expression normalized to the marker β-tubulin. **(C)** NKCC1 expression normalized to the marker α-tubulin. **(D)** Quantification of BDNF expression in SHAM/TBI male mice. **(E)** Quantification of BDNF expression in SHAM/TBI female mice. Data were analyzed using Two-way ANOVA with post-hock Tukey’s multiple comparisons test. Significance was determined as p* < 0.05; p** < 0.01; p*** < 0.001.

The results on survival of PV + -interneurons suggested that pro-apoptotic and pro-survival signaling may be regulated differently between females and males. We have previously shown that P75^NTR^ can be upregulated in males under pathological conditions (Shulga et al., 2008, 2012), leading to increased pro-apoptotic signaling. Additionally, P75^NTR^ levels are low in the DG under physiological conditions (Kourdougli et al., 2017), while the pro-survival signaling of BDNF is high. Considering these observations, we investigated the expression of the P75^NTR^. Using Western blot analysis of total hippocampal protein, we found that P75^NTR^ is upregulated in females following TBI [Male SHAM: 1.00 ± 0.2, *n* = 12, TBI: 1.04 ± 0.17, *n* = 12, *p* > 1.0; Female SHAM: 1.2 ± 0.2, *n* = 12, TBI: 4.00 ± 0.75, *n* = 12, *p* = 0.0004; Two-way Anova: *F*_(3,31)_ = 12.54, *p* < 0.0001; Figure 5A]. This data is consistent with previous findings showing an upregulation of p75^NTR^ protein using Western blot analysis 72 h post-TBI in females but not in males (Gölz et al., 2019).

### Hippocampal BDNF is upregulated in male but not in female after TBI

Considering our results showing an increased loss of PV + cells in female TBI mice associated with a higher expression of the pro-apoptotic factor P75^NTR^, we aimed to assess if pro-survival signaling could also be disrupted.

Neurotrophins, such as the BDNF, are known to be involved in “restorative processes” following TBI. Indeed, BDNF is the most abundant neurotrophin expressed in the brain and is involved in several major cellular processes, such as survival, plasticity and axonal growth (Gustafsson et al., 2021). To investigate this question, we quantified the expression of BDNF in hippocampi by ELISA. We found that BDNF expression was significantly decreased in male after TBI (SHAM: 96.2 ± 5.5 pg./mL, *n* = 12, TBI: 75.6 ± 2.0 pg./mL, *n* = 12, *p* = 0.006, Figure 5D) whereas there were no significant differences were noticed in females [SHAM: 79.9 ± 5.1 pg./mL, *n* = 12, TBI: 82.7 ± 4.8 pg./mL, *n* = 12, *p* = 0.964; Two-way Anova: *F*_(3,33)_ = 4.634, *p* = 0.008; Figure 5E]. We also questioned if this BDNF expression disruption was specific to the ipsiletional hemisphere or if both hippocampi were afflicted. To assess that, we proceeded to do the same analysis while this time specifically quantifying BDNF in left (*L* = contralateral) and right (*R* = ipsilateral) hippocampi. Here, our results show that in males the ipsilateral (*R*) side to the lesion is mainly affected (SHAM *R*: 103.8 ± 9.8 pg./mL TBI *R*: 77.5 ± 3.3 pg./mL, *p* = 0.02, Figure 5D) while the contralateral (*L*) side to the lesion tend to be disrupted without showing statistical significance (SHAM *L*: 88.6 ± 3.8 pg./mL TBI *L*: 73.6 ± 2.3 pg./mL, *p* = 0.45, Figure 5D). In contrast, no differences were measured in females on the ipsilateral (*R*) nor the contralateral (*L*) (SHAM *L*: 78.7 ± 10.2 TBI *L:* 80.9 ± 3.4, *p* > 0.9, Figure 5E, SHAM *R:* 74.99 ± 9.1 TBI *R:* 86.4 ± 6.3, *p* > 0.9, Figure 5E).

Taken together these results suggest that PV cell death following TBI may involve the activation of sex and region-specific pathways.

### Hippocampal chloride homeostasis alterations are sex-specific after TBI

In the same manner, we demonstrated in prior studies that TBI results in dysregulations of chloride homeostasis (Goubert et al., 2019). More specifically, we previously assessed the effect of TBI on KCC2 expression and had determined that KCC2 was internalized after TBI which was consistent with changes in chloride homeostasis and GABAergic transmission in the DG (Goubert et al., 2019).

Chloride co-transporter maturation is known to be dimorphic (Galanopoulou, 2008a; Murguía-Castillo et al., 2013; Perrot-Sinal et al., 2007). Specifically, KCC2/NKCC1 maturation varies based on age and sex, with KCC2 being significantly more expressed in females during the neonatal period compared to males, before stabilizing in adulthood. Furthermore, following TBI in males, KCC2 internalization leads to a reversion to an immature-like neuronal state, disrupting GABAergic signaling. Consequently, we examined whether the same pattern was observed following TBI or if a sex-specific response could affect chloride homeostasis. To address this question, we measured the expression of KCC2 and NKCC1 in the hippocampi of both male and female SHAM and TBI. To do so, we once more used western blot of *toto* hippocampi. Here, our data show no differences in KCC2 expression between male and female after TBI [Male SHAM: 0.85 ± 0.05, *n* = 9, TBI: 0.80 ± 0.07, *n* = 9, *p* > 0.99 Female SHAM: 0.97 ± 0.06, *n* = 12, TBI: 1.13 ± 0.13, *n* = 12 *p* > 0.99; Two-way Anova: *F*_(3,27)_ = 1.664, *p* = 0.2; Figure 5C**]**. Moreover, as previously observed in males, no differences were detected in NKCC1 in males (Male SHAM: 1.06 ± 0.04, *n* = 9, TBI: 0.80 ± 0.10, *n* = 9, *p* = 0.97, Figure 5C). Interestingly, in females we observed a significant increase in NKCC1 expression [Female SHAM: 0.98 ± 0.12, *n* = 12, TBI: 3.65 ± 0.74, *n* = 12, *p* = 0.0004; Two-way Anova: *F*_(3,27)_ = 11.20, *p* < 0.0001; Figure 5C**]**. These results indicate that mild TBI appeared to affect chloride homeostasis, similar to the effects observed in severe TBI (Goubert et al., 2019). However, in this context. Females seemed to be more susceptible to these effect and to involve NKCC1 dysregulation.

### Differential gene expression in the TBI brain: sex-specific patterns

**Figure 6:**
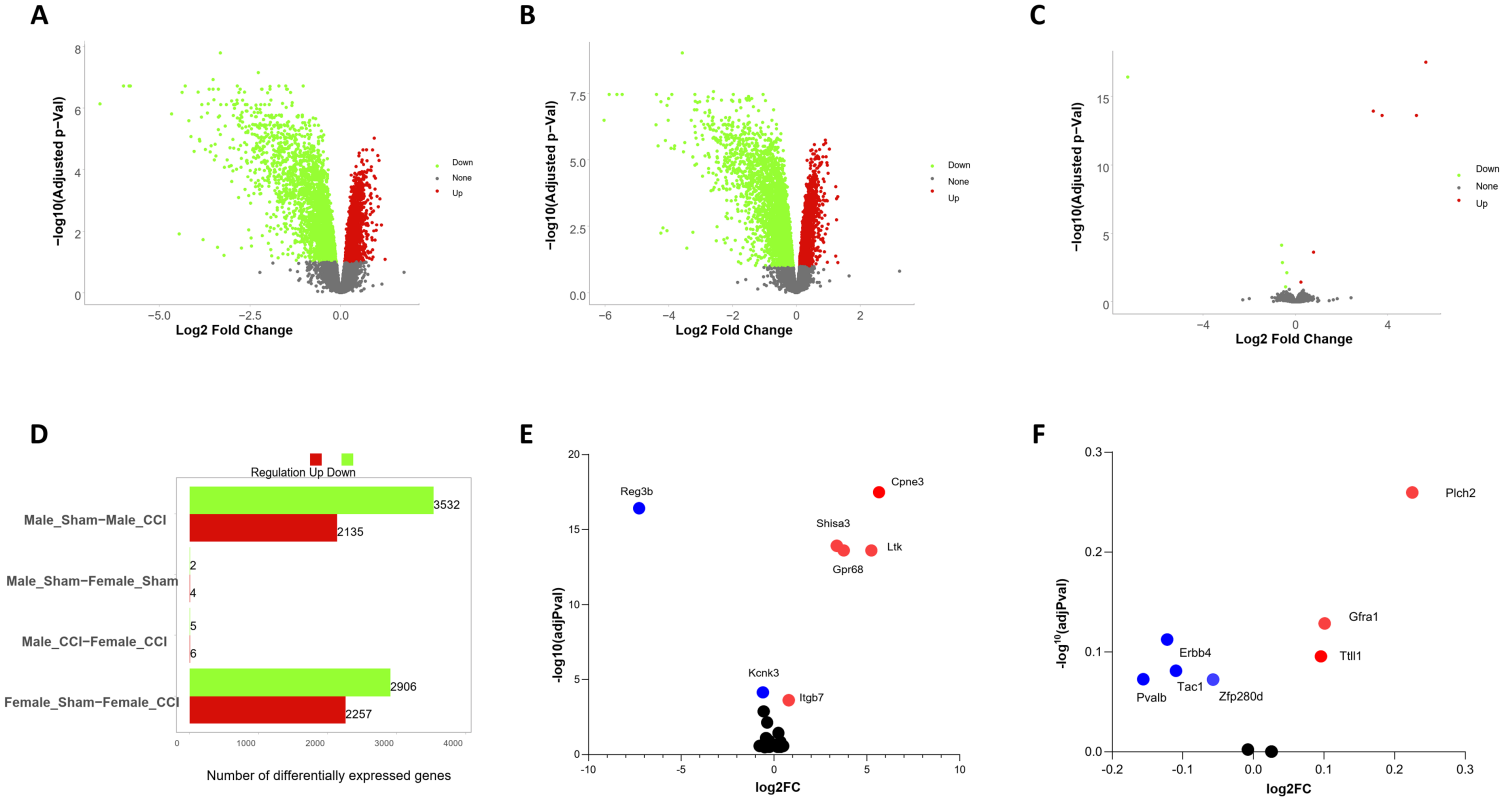
Sex stratified differential gene expression analysis of CCI animals. **(A)** Differentially expressed genes (DEGs) identified in male traumatic brain injury (TBI) control samples compared to male controls, analyzed independently. Nominal significance was defined as a *p*-value < 0.05 and |log2 fold change| > 0.5. Red nodes indicate significantly upregulated genes, while green nodes represent significantly downregulated genes (see Supplementary table). **(B)** DEGs identified in female TBI samples compared to female controls, analyzed as described for males in **(A)**. **(C)** Comparison of DEGs between male and female TBI samples displaying genes specifically differentially expressed in females. **(D)** Total counts of DEGs identified across all brain regions, stratified by sample sex. Total DEG counts for male versus female samples are summarized to highlight sex-specific differences in gene expression patterns. **(E)** Differential expression of genes with highly significant changes. Red nodes indicate significantly upregulated genes, while blue nodes represent significantly downregulated genes. **(F)** Differential expression of interneuron-related genes. Red nodes indicate upregulated genes, while blue nodes represent downregulated genes.

Transcriptomic analysis (Figures 6A,B,D; Supplementary Table 1) identified significant upregulation of Copine 3 (CPNE3) (adj *p* = 3.23*10^−18^), SHISA3 (adj *p* = 1.21*10^−14^), GPR68 (OGR1) (adj *p* = 2.47*10^−14^), and LTK (adj *p* = 2.47*10^−14^) (Figures 6C,E) in the female TBI brain compared to the male TBI brain. These findings suggest sex-specific molecular responses to TBI, particularly in pathways involved in calcium signaling, inflammation, and neurogenesis.

CPNE3, a calcium-binding protein critical for intracellular calcium signaling and cognitive processes, exhibited increased expression in the female TBI brain. Given CPNE3’s role in modulating anxiety and working memory, its upregulation may reflect a protective or compensatory mechanism aimed at mitigating the cognitive and emotional impairments commonly associated with TBI (Chen et al., 2021; Khvotchev and Soloviev, 2024).

The pro-inflammatory mediator SHISA3 was significantly upregulated in the female TBI brain. SHISA3 promotes macrophage M1 polarization via the NF-κB pathway by forming immune complexes with HSPA8, reciprocally activating NF-κB signaling. The elevated SHISA3 expression suggests heightened pro-inflammatory activity in females post-TBI, which may contribute to sex-specific differences in neuroinflammation and neurovascular recovery (Zhang et al., 2024).

GPR68 (OGR1), a proton-sensing G-protein-coupled receptor, was significantly upregulated in females. This receptor, activated by acidic extracellular pH, is known to regulate pH homeostasis, immune responses, and inflammation. Importantly, in macrophages, TDAG8 and, to a lesser extent, OGR1, mediate the inhibition of cytokine production under acidic conditions. The inhibitory effect of acidic pH on cytokine production is significantly reduced in macrophages from TDAG8-deficient mice but is less affected in those lacking OGR1. These findings highlight the nuanced roles of OGR1 and TDAG8 in modulating inflammatory responses, particularly in the context of acidic microenvironments, such as those induced by TBI (Ishii et al., 2005; Ludwig et al., 2003; Mogi et al., 2009; Mohebbi et al., 2012; Okajima, 2013; Seuwen et al., 2006; Wang et al., 2004).

LTK, a receptor tyrosine kinase expressed in the hippocampus, was also upregulated in the female TBI brain. LTK signaling plays a compensatory role in hippocampal neurogenesis when ALK (anaplastic lymphoma kinase) activity is reduced. Its upregulation in females may contribute to enhanced neurogenic responses following injury, potentially mitigating TBI-induced deficits in spatial learning and memory (Katic and Priscan, 2023; Weiss et al., 2012).

Analysis of genes that are known to be enriched in PV + show although not statistically significant sex-specific expression changes (Figure 6F) with decreased expression of Pvalb (adj *p* = 0.07), Tac (adj *p* = 0.08), Erbb4 (adj *p* = 0.11) and (adj p = 0.08) and increased expression of Trip13 (adj *p* = 0.25), Gfra1 (adj *p* = 0.12) and Ttll1 (adj *p* = 0.09). These results go in hand with the altered region-specific differences in PV interneuron survival.

The upregulation of CPNE3, SHISA3, GPR68 (OGR1), and LTK in the female TBI brain highlights distinct sex-specific molecular responses to injury. While CPNE3 and LTK appear to support cognitive and neurogenic recovery, the elevated levels of SHISA3 and GPR68 (OGR1) underscore heightened inflammatory and pH regulatory responses in females. Furthermore, the differential roles of OGR1 and TDAG8 in cytokine inhibition provide insight into the fine-tuned regulation of inflammation in macrophages, with potential implications for sex-specific therapeutic strategies in TBI.

## Discussion

This study provided a detailed multi-level analysis of sex-specific responses to traumatic brain injury (TBI) in mice. By using a model of mild TBI and a comprehensive battery of behavioral, electrophysiological, cellular, and molecular assessments, we identified consistent sex-dependent patterns in post-TBI phenotypes. These findings underscore the need to consider biological sex as a key factor in experimental TBI research and in the development of effective therapies.

After injury, locomotor performance remained stable in both male and female mice, indicating that any behavioral changes were not due to motor impairments. In the OFT, both male and female TBI groups showed a reduced latency to first enter the anxiogenic center zone compared to shams, suggesting some initial disinhibition or altered exploratory drive. However, there were no differences in the total time spent in the center zone, indicating no strong overall shift in anxiety-like behavior due to TBI. In contrast, the EPM revealed a sex-specific effect: female TBI mice spent significantly more time in the open arms than female shams mice (indicative of reduced anxiety or increased risk-taking), whereas male TBI mice did not differ from male shams mice in this measure (Pawlak et al., 2012). This pattern suggests that TBI may increase exploratory or risk-taking behavior selectively in females, a finding consistent with other reports of sex-specific behavioral outcomes after brain trauma.

In BMT, both sexes improved their performance over training, though males generally outperformed females under SHAM conditions. TBI disrupted this learning process in a sex-dependent manner. Female TBI mice showed delayed acquisition of the task and a significantly longer latency to locate the escape hole during the first probe trial (PTD1) compared to female SHAM (Gawel et al., 2019; Rodríguez Peris et al., 2024). Male TBI mice also exhibited impairments in learning and memory, but these were milder at early time points. Notably, by the second training phase and the subsequent probe trial (PTD2), the performance of female TBI mice recovered to levels comparable to uninjured females, whereas male TBI mice continued to display prolonged escape latencies and mild spatial memory deficits. These results suggest that females experience greater initial susceptibility to TBI-related cognitive disruption but also demonstrate a more effective recovery over time. The observed sex differences in spatial memory align with prior studies indicating that males are particularly vulnerable to TBI-induced deficits in hippocampus-dependent learning (Fedor et al., 2010; Gawel et al., 2019). Females showed early learning impairments, their eventual recovery to near-sham performance suggests an ability to engage compensatory neuroplastic mechanisms. Although not directly tested here, previous studies suggest that estrogen may play a role in the enhanced recovery observed in females (Galanopoulou, 2008b; Gupte et al., 2019; Rigon et al., 2016). Over time, such protective factors may help female mice overcome initial deficits in learning and memory.

Electrophysiological recordings revealed that TBI altered neural network activity in the hippocampus of both sexes. In particular, power in the theta (∼4–10 Hz) and alpha (∼10–12 Hz) frequency bands were elevated in TBI mice relative to SHAM, in both males and females. Theta rhythms, which are critical for spatial memory encoding, were notably increased in female TBI mice. While the overall magnitude of oscillatory power change was comparable between injured males and females, the female TBI group showed greater heterogeneity in their oscillatory patterns, hinting at more variable network adaptations post-injury. Disruption of theta oscillations has been linked to impaired hippocampal function and cognitive deficits in TBI models (Fedor et al., 2010), and the sex-specific patterns observed here align with evidence of differential network dynamics and plasticity between males and females after brain injury (Murguía-Castillo et al., 2013).

Histological analyses indicated a sex-specific vulnerability of inhibitory interneurons in the hippocampus. Female TBI mice showed a significant loss of parvalbumin-positive (PV+) interneurons in both the DG and CA1 regions, whereas male TBI mice had relatively preserved PV + cell counts (with no significant loss detected in CA1). This pronounced interneuron loss in females coincided with elevated pro-apoptotic signaling, evidenced by a significant upregulation of the p75^NTR^ neurotrophin receptor in the female hippocampus post-TBI (Gölz et al., 2019). These findings are consistent with reports that female brains exhibit heightened neuroinflammatory and apoptotic responses following TBI (Tessier et al., 2023). Given that PV + interneurons play a critical role in maintaining inhibitory tone and network stability, their loss may contribute to greater circuit dysregulation in female mice after injury. In contrast to this sex divergence in cell vulnerability, we observed an opposite pattern in levels of BDNF in the post-TBI hippocampus. Male TBI mice exhibited a significant reduction in hippocampal BDNF expression compared to SHAM, whereas female TBI mice maintained BDNF levels that were comparable to uninjured controls. This suggests that male recovery may depend more heavily on BDNF-mediated neuroplasticity, which is compromised by injury in males (Gustafsson et al., 2021), while females retain trophic support that could aid in neural repair and functional compensation. The preservation of BDNF in females might partially explain their better late-stage cognitive recovery, as BDNF is known to support synaptic plasticity and neuron survival.

The impact of TBI on neuronal chloride homeostasis and inhibitory signaling also differed by sex. We found no significant change in hippocampal KCC2 expression (which encodes the principal neuronal K^+^/Cl^−^ cotransporter that extrudes chloride and strengthens GABA_A inhibitory effects) in either sex after TBI. However, female TBI mice displayed a marked upregulation of NKCC1, a chloride importing cotransporter that tends to make GABAergic transmission more depolarizing (excitatory). This shift toward an immature-like chloride transporter profile in females — increased NKCC1 without a compensatory increase in KCC2 — could lead to a reduction in inhibitory tone and greater network hyperexcitability post-injury (Goubert et al., 2019; Galanopoulou, 2008a). Notably, prior studies in predominantly male TBI models have reported a downregulation of KCC2 and consequent loss of inhibitory GABAergic signaling (Galanopoulou, 2008a; Goubert et al., 2019). The absence of such KCC2 changes in females, coupled with increased NKCC1, highlights a different pathophysiological trajectory by which female brains may experience post-traumatic disinhibition relative to males.

Transcriptomic analysis further underscored divergent molecular responses to TBI between males and females. In female hippocampal tissue, several genes were significantly upregulated relative to males, including Cpne3 (Chen et al., 2021), Shisa3 (Zhang et al., 2024), Gpr68 (OGR1) (Ishii et al., 2005; Belovicova et al., 2017), and Ltk(Katic and Priscan, 2023; Rodríguez Peris et al., 2024). These genes are linked to distinct biological processes: CPNE3 is involved in calcium signaling and has been implicated in working memory function (Chen et al., 2021); SHISA3 participates in cell signaling pathways and may modulate inflammatory processes (Zhang et al., 2024); GPR68 (OGR1) acts as a proton-sensing receptor that can influence pH-dependent neuroinflammatory and vascular responses (Ishii et al., 2005; Belovicova et al., 2017); and LTK is a receptor tyrosine kinase associated with neurodevelopment and hippocampal neurogenesis (Katic and Priscan, 2023). The female-specific upregulation of LTK is particularly notable, as it may signal enhanced neurogenic potential in the injured female hippocampus, which could help mitigate spatial memory impairments (Rodríguez Peris et al., 2024). The selective engagement of these molecular pathways in females suggests that the female brain activates unique mechanisms after TBI — potentially enhancing calcium-dependent synaptic plasticity, invoking distinct inflammatory/pH homeostatic responses, and promoting regenerative processes to a greater extent than the male brain. This distinctive molecular profile may underlie the paradoxical observation of greater cellular vulnerability in females (e.g., interneuron loss) alongside a strong capacity for functional recovery. In support of this, transcripts related to interneuron populations (including Pvalb [parvalbumin], Erbb4, and Tac1) exhibited trends toward differential expression in females consistent with their greater PV-cell loss, although these particular changes did not reach statistical significance. Taken together, these data illustrate that the female brain’s injury response involves a multifaceted gene expression program that diverges significantly from that of the male, potentially driving the different outcomes observed between sexes.

Overall, this study highlights the necessity of incorporating sex as a critical variable in preclinical TBI studies. A deeper understanding of how male and female brains respond differently to trauma will be essential for the design of more effective, personalized therapeutic interventions, ultimately improving outcomes for both men and women with TBI.

## Significance statement

This study reveals a complex sex-specific dissociation between interneuron survival, neurophysiological alterations, and cognitive outcomes following TBI. Despite greater loss of hippocampal PV + interneurons and elevated pro-apoptotic signaling via p75^NTR^ in females, spatial memory deficits were transient and resolved over time, contrasting with persistent impairments observed in males. This was paralleled by female-specific transcriptional signatures involving genes related to neurogenesis, calcium signaling, and inflammation (e.g., CPNE3, SHISA3, GPR68, LTK), as well as increased NKCC1 expression, suggesting chloride homeostasis disruption. In males, reduced BDNF and localized theta rhythm disruption aligned with sustained cognitive deficits. These results underscore the importance of sex-informed strategies in TBI research and treatment, especially those targeting neurotrophic support, interneuron preservation, and chloride regulation.

## Data Availability

The datasets presented in this study can be found in online repositories. The names of the repository/repositories and accession number(s) can be found in the article/Supplementary material.
