# Supplementary material for: Sex-specific interneuron vulnerability after traumatic brain injury correlates with neurotrophic signaling and chloride homeostasis, independent of behavioral and network outcomes
Source: Front Cell Neurosci. 2025 Sep 24;19:1572213. doi: 10.3389/fncel.2025.1572213 (PMC12504505; doi:10.3389/fncel.2025.1572213)
Supplement: Supplementary file 1 [file Data_Sheet_1.PDF]

symbol	Male_CCI-Female_CCI_log2FC	Male_CCI-Female_CCI_adjPv	Male_CCI-Female_CCI_-log10(adjP
Cpne3	5.654650235	3.23E-18	17.4903475
Reg3b	-7.272360836	3.85E-17	16.4145516
Shisa3	3.375766342	1.21E-14	13.91626425
Ltk	5.241940249	2.47E-14	13.60778018
Gpr68	3.750858564	2.47E-14	13.60778018
Kcnk3	-0.60096543	7.24E-05	4.140069652
Itgb7	0.783732468	0.000237787	3.623812484
Bora	-0.565808447	0.001342715	2.872016311
Mia	-0.37549397	0.007363274	2.132929009
Glt8d2	0.23057398	0.036935231	1.432559182
Ggh	-0.433110448	0.078707613	1.10398326
Fbxo27	-0.266395436	0.12468088	0.90420014
Mkx	0.338140004	0.142993486	0.844683746
Thrb	0.286347638	0.176931355	0.752195197
Svil	-0.396203973	0.178774338	0.747694821
Akip1	-0.511755255	0.183485859	0.736397401
Fcor	0.335457744	0.196013987	0.707712937
Spsb1	0.278503131	0.241177387	0.617663414
Klc2	-0.374282137	0.241177387	0.617663414
Lpar4	0.388856888	0.241177387	0.617663414
Gjc2	0.219745385	0.241177387	0.617663414
Rcn1	0.362530575	0.241177387	0.617663414
Cfap126	-0.335113811	0.241177387	0.617663414
Irx5	-0.606017878	0.249874983	0.602277223
Serpinb6a	0.333337295	0.25303019	0.596827658
L3hypdh	0.267127234	0.260572098	0.58407209
Gjb1	-0.782184834	0.267889722	0.572043948
Mal2	0.211131148	0.267889722	0.572043948
Cep72	-0.298704206	0.267889722	0.572043948
Pip5k1b	0.4901614	0.267889722	0.572043948
Ntrk1	-0.416065972	0.267889722	0.572043948
Dscam	-0.57171008	0.267889722	0.572043948
Luzp1	0.333483427	0.267889722	0.572043948
Relb	0.267571276	0.267889722	0.572043948
Acox2	0.180553034	0.267889722	0.572043948
B3galt2	-0.382152249	0.267889722	0.572043948
Trp53inp1	-0.246614715	0.267889722	0.572043948
Tmem198	0.267275177	0.267889722	0.572043948
Miat	0.220583467	0.267889722	0.572043948
Mmp25	-0.22658628	0.267889722	0.572043948
Kif14	0.22059367	0.267889722	0.572043948
Lamb1	0.217814533	0.267889722	0.572043948
10-Sep	0.186686538	0.267889722	0.572043948
MRVI1	0.324813962	0.270876718	0.567228321
Filip1l	-0.604584956	0.278458564	0.555239421
Sgsm1	0.16461131	0.278458564	0.555239421
Ccdc69	0.457838145	0.278458564	0.555239421

Slc39a8	0.254552039	0.280277579	0.552411642
Upp1	0.20915212	0.280277579	0.552411642
Dhfr	-0.27111293	0.302935515	0.518649809
Pcdha6	-0.25507762	0.302935515	0.518649809
Parvb	0.27098911	0.302935515	0.518649809
Syt13	0.35598189	0.302935515	0.518649809
St14	0.326074823	0.302935515	0.518649809
Tmem219	-0.426794485	0.307323997	0.512403528
Mis18a	0.309006221	0.307323997	0.512403528
Myo10	0.248103691	0.307323997	0.512403528
Syndig1	0.182905393	0.314511126	0.502363987
Kif7	0.243134264	0.314511126	0.502363987
Aifm3	-0.504702968	0.322228232	0.491836411
Nkapl	0.310917448	0.322228232	0.491836411
Akr1b10	0.242297258	0.322228232	0.491836411
Sctr	0.196368101	0.322228232	0.491836411
Rrm1	0.165420935	0.325856049	0.486974213
Cacna1g	0.314009016	0.325856049	0.486974213
Rilpl2	-0.268503399	0.325856049	0.486974213
Slc31a2	0.306688636	0.327232621	0.485143409
Gng10	-0.251675241	0.327232621	0.485143409
Lrp10	0.216468555	0.327232621	0.485143409
Rapgef4	0.179761495	0.327232621	0.485143409
Tubb2b	0.318157429	0.327232621	0.485143409
Ncapg2	0.165411779	0.327232621	0.485143409
Mcur1	-0.671221973	0.328319978	0.48370269
Ptpn4	-0.271735615	0.328319978	0.48370269
Pxdc1	-0.260905721	0.328319978	0.48370269
Tmem179	-0.464740339	0.328319978	0.48370269
Card11	-0.538638495	0.328319978	0.48370269
Bcas1	0.344352381	0.328319978	0.48370269
Zdhhc22	-0.295284228	0.328319978	0.48370269
Cdkn2a	-0.251841386	0.328319978	0.48370269
Cers2	-0.290191963	0.328319978	0.48370269
Slc6a17	0.298799682	0.328319978	0.48370269
Wdr38	0.3683431	0.328319978	0.48370269
Stx1b	-0.220533744	0.328319978	0.48370269
Cd82	0.282389905	0.328319978	0.48370269
Ctso	0.306778956	0.328319978	0.48370269
Cited4	0.583693334	0.328319978	0.48370269
Cacng3	-0.286388198	0.328319978	0.48370269
Brinp1	0.325180262	0.331057998	0.480095916
Sgtb	-0.608587221	0.333654225	0.476703371
Glb1	-0.37144459	0.333654225	0.476703371
Mapk15	-0.438124344	0.333654225	0.476703371
Fuca1	-0.269061259	0.333654225	0.476703371
Mlc1	0.585961303	0.333654225	0.476703371
Olfm4	0.315618406	0.333654225	0.476703371

Lingo1	0.254401516	0.333654225	0.476703371
Insl6	-0.235324261	0.333654225	0.476703371
Nxf7	0.199755502	0.333654225	0.476703371
Zc3h12d	-0.226110749	0.333654225	0.476703371
Teddm3	-0.232060083	0.333654225	0.476703371
Lmtk2	0.259176466	0.333654225	0.476703371
B3gnt9	-0.151364842	0.333654225	0.476703371
Maff	0.275190085	0.333654225	0.476703371
Elovl5	0.182602055	0.333654225	0.476703371
Map3k14	0.147508382	0.333654225	0.476703371
Tmem151	0.358175532	0.333654225	0.476703371
Clec2l	0.254879541	0.333654225	0.476703371
Aprt	-0.685537425	0.333654225	0.476703371
Mctp2	-0.255305865	0.333654225	0.476703371
Il2rb	-0.25038537	0.333654225	0.476703371
Dbx2	0.300620378	0.333654225	0.476703371
Iqsec3	0.177206748	0.333654225	0.476703371
Fgd3	0.189038254	0.333654225	0.476703371
Casp6	0.138925142	0.333654225	0.476703371
Car9	0.165985375	0.333654225	0.476703371
Tmem220	-0.489966085	0.336155289	0.473460051
Snx5	-0.555554389	0.336155289	0.473460051
FAM105A	0.293098906	0.336155289	0.473460051
Tnn	0.264635202	0.336155289	0.473460051
Rec114	0.189710617	0.336155289	0.473460051
Olr1	0.297393922	0.342799857	0.464959368
Tgm3	-0.137415443	0.354118332	0.45085159
PAK7	0.187279366	0.354118332	0.45085159
Flna	0.148239177	0.356669434	0.447734107
St8sia5	0.151947774	0.357181799	0.44711068
Dhcr24	-0.296258577	0.35774227	0.446429741
Natd1	0.182945261	0.35774227	0.446429741
Col8a2	0.230817933	0.35774227	0.446429741
Btg1	0.29813678	0.364002379	0.438895778
Nrp1	0.305641519	0.364283773	0.438560174
Sh3glb1	0.204554706	0.370005009	0.431792396
Hrh1	-0.254743807	0.376939711	0.423728107
Car3	0.20130588	0.376939711	0.423728107
Plscr4	0.255153452	0.379927394	0.420299391
Cux2	0.274323881	0.379927394	0.420299391
Ephb3	0.245294356	0.379927394	0.420299391
Camp	-0.295320182	0.38249923	0.417369434
Defb11	0.256030596	0.395846573	0.40247311
Kcnf1	0.241384703	0.39688385	0.401336573
Nlk	0.158520474	0.39688385	0.401336573
Bsn	0.199410198	0.39688385	0.401336573
Bc1	0.459786063	0.39688385	0.401336573
Mad2l1	0.359609527	0.399696853	0.398269271

BC089491	-0.389068553	0.399696853	0.398269271
Rbpms2	-0.204511131	0.399696853	0.398269271
Kif5a	0.201440609	0.399696853	0.398269271
Bend6	0.150258073	0.401417907	0.396403257
Mmp11	0.19320523	0.403752091	0.393885215
Tfpi	0.148026775	0.41092481	0.386237637
Gas1	-0.210914113	0.41092481	0.386237637
Cryba2	0.326918851	0.411726448	0.385391235
Hbb-y	-0.193178477	0.418944161	0.377843859
Shisa8	0.771732114	0.425895151	0.370697305
Adarb1	-0.44093406	0.425895151	0.370697305
Nfatc4	0.1577667	0.425895151	0.370697305
Afap1l2	0.330035167	0.425895151	0.370697305
Wtip	0.176687521	0.425895151	0.370697305
Scube1	-0.166663731	0.427382721	0.369183041
Sln	-0.209524014	0.428050509	0.368504983
Paox	-0.151677829	0.429664504	0.366870523
Mastl	-0.139135156	0.430096765	0.366433824
HIST1H2AE	-0.224242147	0.430096765	0.366433824
Pdlim1	0.260875387	0.430096765	0.366433824
Shmt1	0.250913834	0.430096765	0.366433824
Cd40	0.196462625	0.430096765	0.366433824
Ogfr	0.240483146	0.434958597	0.361552081
Krt19	0.276290557	0.437639132	0.358883852
Cdkn2b	0.227845404	0.437639132	0.358883852
Nlrc3	0.215619003	0.437639132	0.358883852
Peg13	0.190567601	0.437639132	0.358883852
Lamc1	0.130932479	0.437639132	0.358883852
Zbtb11os1	0.349043676	0.44267768	0.353912374
Arhgap25	0.217443221	0.44267768	0.353912374
Sox10	0.29937188	0.449965951	0.346820348
Cd248	0.181203328	0.449965951	0.346820348
Zfp973	-0.768350991	0.451747196	0.345104535
Cdk6	0.401546601	0.451747196	0.345104535
Ggt1	-0.299164859	0.451747196	0.345104535
Pllp	-0.576231745	0.451747196	0.345104535
Bcl10	0.50774802	0.451747196	0.345104535
Eef2kmt	0.424522314	0.451747196	0.345104535
Actb	-0.328334163	0.451747196	0.345104535
Htr2a	-0.174235947	0.451747196	0.345104535
Pamr1	-0.331777731	0.451747196	0.345104535
Xlr4a	0.485029263	0.451747196	0.345104535
Fbxw7	-0.244284314	0.451747196	0.345104535
Shank2	0.207059941	0.451747196	0.345104535
Tst	0.137588195	0.451747196	0.345104535
Ckap4	-0.217757819	0.451747196	0.345104535
Pld1	0.188909764	0.451747196	0.345104535
Ccdc184	-0.365102856	0.451747196	0.345104535

Pkn3	0.23518337	0.451747196	0.345104535
Hsd11b1	0.206532977	0.451747196	0.345104535
Plp1	-0.213548787	0.451747196	0.345104535
Gypc	-0.524535877	0.451747196	0.345104535
Cldn9	0.382928924	0.451747196	0.345104535
Irak2	-0.243464642	0.451747196	0.345104535
Asap1	-0.395930596	0.451747196	0.345104535
Lsmem2	0.305167214	0.451747196	0.345104535
Kdelr2	0.283419468	0.451747196	0.345104535
WISP1	-0.14792183	0.451747196	0.345104535
Pou6f1	0.269011926	0.451747196	0.345104535
Acss1	-0.140316363	0.451747196	0.345104535
Rap1gap2	-0.174311769	0.451747196	0.345104535
Hpdl	0.189981862	0.451747196	0.345104535
Ephb6	0.184825293	0.451747196	0.345104535
Tnfsf8	0.195986922	0.451747196	0.345104535
Lgi4	0.200085874	0.451747196	0.345104535
Pla1a	0.261679985	0.451747196	0.345104535
Efemp1	0.18780688	0.451747196	0.345104535
Nmrk1	-0.310291092	0.451747196	0.345104535
Marcks	0.175473658	0.451747196	0.345104535
Fgf10	0.269846179	0.451747196	0.345104535
Ninj2	0.277265007	0.451747196	0.345104535
Scn8a	-0.180132757	0.451747196	0.345104535
Plxdc2	0.235969494	0.451747196	0.345104535
Atp8b4	-0.218663784	0.451747196	0.345104535
Etl4	-0.314464038	0.451747196	0.345104535
Psd3	-0.132783095	0.451747196	0.345104535
Nefl	-0.157467512	0.451747196	0.345104535
Mb21d2	-0.204361556	0.451747196	0.345104535
Teddm2	0.236938759	0.451747196	0.345104535
Hcn1	0.137329905	0.451747196	0.345104535
Snx33	0.177696332	0.451747196	0.345104535
Diaph3	0.209277131	0.451747196	0.345104535
Hscb	0.192028404	0.451747196	0.345104535
Gkn3	0.265504411	0.451747196	0.345104535
Ncald	0.211077638	0.451747196	0.345104535
Abcd1	0.669458254	0.451747196	0.345104535
Pdzrn3	-0.160331939	0.451747196	0.345104535
Padi4	0.423981776	0.451747196	0.345104535
Hbegf	-0.421951422	0.451747196	0.345104535
Parp4	0.288440198	0.451747196	0.345104535
Btbd3	-0.181076266	0.451747196	0.345104535
Adamts4	-0.223027687	0.451747196	0.345104535
Htr1a	0.310540151	0.451747196	0.345104535
Prdm1	-0.216153717	0.451747196	0.345104535
Gnpda1	0.149185262	0.451747196	0.345104535
Slc24a2	0.281149809	0.451747196	0.345104535

Trim30c	0.202280706	0.451747196	0.345104535
Sowahc	0.302283155	0.451747196	0.345104535
Plekho2	0.21554521	0.451747196	0.345104535
Hrh2	0.206479709	0.451747196	0.345104535
Tnfaip3	0.199553495	0.451747196	0.345104535
Abcb4	0.31283118	0.451747196	0.345104535
Calu	0.239235683	0.451747196	0.345104535
Gpr22	0.158692595	0.451747196	0.345104535
Ifitm1	0.215504658	0.451747196	0.345104535
Far2	-0.137866364	0.451747196	0.345104535
Tmem200	0.203165836	0.451747196	0.345104535
Slc39a2	0.265521649	0.451747196	0.345104535
Cyfip2	0.198128307	0.451747196	0.345104535
Nox4	0.288570176	0.451747196	0.345104535
Adrb2	0.230305977	0.451747196	0.345104535
Inhba	0.294863534	0.451747196	0.345104535
Malat1	0.128636051	0.451747196	0.345104535
Lipg	-0.401286776	0.451747196	0.345104535
Psors1c2	-0.181558862	0.451747196	0.345104535
Elavl4	0.191251337	0.451747196	0.345104535
Cdc6	0.229636826	0.451747196	0.345104535
Kcnj11	0.150879434	0.451747196	0.345104535
Ccdc122	0.183495141	0.451747196	0.345104535
Gla	0.592213512	0.451747196	0.345104535
Rsu1	-0.195536149	0.451747196	0.345104535
Olfr570	0.175362705	0.453758527	0.343175201
Gmnn	0.224646547	0.454127491	0.342822207
PTRF	0.235296509	0.454235931	0.342718516
Omp	0.105592943	0.454235931	0.342718516
Colec12	0.318507553	0.454235931	0.342718516
Adipor2	0.184780257	0.454235931	0.342718516
Pold4	0.313202379	0.454235931	0.342718516
Tmem25	0.41409762	0.454235931	0.342718516
Echdc3	0.337603812	0.454235931	0.342718516
Lenep	0.218276539	0.454235931	0.342718516
Itpr1	-0.52014221	0.455671619	0.341348021
Clspn	0.286126314	0.455671619	0.341348021
Tnni3	-0.17959226	0.455671619	0.341348021
Il12a	0.250715172	0.455671619	0.341348021
Efr3a	-0.256891565	0.455671619	0.341348021
Gdpd2	-0.229348633	0.463025461	0.334395128
Sdf2l1	0.281201191	0.463025461	0.334395128
Prkar1b	0.130283632	0.463025461	0.334395128
Pxn	-0.5645219	0.464222348	0.333273956
Bcl2l12	-0.455250283	0.464222348	0.333273956
Gna12	0.24174202	0.464222348	0.333273956
Alx4	0.266831618	0.464222348	0.333273956
Dusp2	-0.364713861	0.464222348	0.333273956

Trp53i13	0.388157994	0.464222348	0.333273956
Tmem215	0.190521656	0.464222348	0.333273956
Ptpn7	0.249483861	0.464222348	0.333273956
Smoc2	0.263092632	0.464222348	0.333273956
Rad54b	0.195736658	0.464222348	0.333273956
Ly6a	0.212245966	0.464222348	0.333273956
Ptgs2os	0.260456145	0.464222348	0.333273956
Nrep	0.204150074	0.464222348	0.333273956
Hadha	-0.143701119	0.464222348	0.333273956
Ugdh	0.23988714	0.464222348	0.333273956
Rbks	0.351746316	0.465250671	0.332312992
Ppifos	-0.418451769	0.465250671	0.332312992
Kifc2	0.344576397	0.465250671	0.332312992
Kcnma1	0.234676388	0.465250671	0.332312992
RLTPR	0.146135409	0.465250671	0.332312992
Mtmr10	0.194277039	0.466725486	0.330938483
Ell3	-0.125458136	0.467619281	0.33010759
Cd209f	-0.352204577	0.469559495	0.328309373
6-Sep	0.178185075	0.469559495	0.328309373
FAM129A	0.339975905	0.469559495	0.328309373
Sult2b1	0.214947561	0.469559495	0.328309373
Kank2	0.117566359	0.469559495	0.328309373
Olig2	0.364257478	0.469971378	0.32792859
Deptor	0.198090586	0.470308612	0.327617069
Fxyd7	-0.324012811	0.470308612	0.327617069
Clstn2	-0.249892166	0.470308612	0.327617069
FAM101A	0.175639886	0.470308612	0.327617069
Sult6b1	0.145910343	0.470308612	0.327617069
Maml2	-0.148768462	0.470308612	0.327617069
Zdhhc12	0.375844763	0.470308612	0.327617069
Ddx31	-0.308518159	0.470779507	0.32718245
Tlr12	0.217184919	0.470779507	0.32718245
Poc1b	-0.145913046	0.470779507	0.32718245
HIST1H2B(	0.185767138	0.471593222	0.326432445
Eps8l2	0.170312856	0.474197745	0.324040516
Sorl1	-0.118655858	0.474807202	0.323482702
Slitrk1	-1.01725003	0.477767435	0.320783455
Stxbp1	-0.366316756	0.477767435	0.320783455
Scimp	-0.368548553	0.477767435	0.320783455
Tshz2	0.191236717	0.477767435	0.320783455
Slc25a20	0.595495329	0.477767435	0.320783455
Aspa	0.250490479	0.477767435	0.320783455
Fmnl1	0.236071954	0.477767435	0.320783455
Sec1	-0.245329994	0.477767435	0.320783455
Cdr1	-0.290880064	0.477767435	0.320783455
Ccdc17	-0.101847336	0.477767435	0.320783455
Cst6	-0.183246382	0.477767435	0.320783455
Acsf2	0.264488703	0.477767435	0.320783455

Trim30b	0.613303658	0.477767435	0.320783455
Fmo2	0.287319005	0.477767435	0.320783455
Bard1	-0.231368378	0.477767435	0.320783455
Map4k1	-0.116331361	0.477767435	0.320783455
Kcnb2	-0.196568156	0.477767435	0.320783455
Rims2	0.248389718	0.477767435	0.320783455
Trpc6	0.156491284	0.477767435	0.320783455
Acadl	0.126736855	0.477767435	0.320783455
Reep3	0.1965899	0.477767435	0.320783455
Actl9	0.175398805	0.477767435	0.320783455
Gbp4	0.178040161	0.477767435	0.320783455
Ugt1a6a	0.128394341	0.477767435	0.320783455
Slc2a5	0.385785535	0.477767435	0.320783455
Cdca7	0.167327247	0.477767435	0.320783455
Tcf7l2	0.205689371	0.477767435	0.320783455
Frrs1l	0.254743554	0.477767435	0.320783455
Fabp3	0.148165989	0.477767435	0.320783455
Stag3	0.256842085	0.477767435	0.320783455
AA465934	0.287316211	0.477767435	0.320783455
Chd5	0.125960831	0.477767435	0.320783455
Serpina3g	-0.233359392	0.477767435	0.320783455
Tmem154	0.175131881	0.477767435	0.320783455
Sapcd2	-0.098733994	0.477767435	0.320783455
Clec10a	0.17615579	0.477767435	0.320783455
Pgls	0.14933526	0.477767435	0.320783455
Gpr150	0.17397297	0.477767435	0.320783455
Pnrc2	0.168392957	0.477767435	0.320783455
Sardh	0.192937933	0.477968094	0.320601093
Kirrel	0.203389803	0.48302344	0.316031793
Zfp972	0.15349014	0.485556168	0.313760524
Gab3	-0.657460489	0.485556168	0.313760524
Sema3d	0.167465609	0.486021325	0.313344675
Nphs1os	-0.344608494	0.486988841	0.312480991
Mei1	-0.189715601	0.486988841	0.312480991
Pdia2	-0.344860408	0.48820989	0.311393427
Slc1a6	0.139146649	0.489088846	0.310612241
Nms	-0.213122939	0.491031051	0.308891044
Mapk10	0.308054421	0.492120086	0.307928909
Igfn1	0.288228235	0.493049272	0.307109679
Sec24d	0.157673659	0.494116195	0.306170912
Vstm4	0.242260881	0.494139438	0.306150483
Wnt1	-0.381457695	0.494484186	0.305847593
Gimap6	-0.517858322	0.494657373	0.305695513
Ptgr2	-0.232839677	0.494657373	0.305695513
Adrb1	0.456099105	0.494657373	0.305695513
Pdzd2	-0.131357511	0.494657373	0.305695513
Ppme1	0.158929463	0.494657373	0.305695513
Ccdc86	-0.294329822	0.494657373	0.305695513

Svbp	-0.21803458	0.494657373	0.305695513
Nol4	-0.336763105	0.496085456	0.304443506
Cbr3	0.307405282	0.496481972	0.304096517
Ppib	0.202608341	0.49774903	0.302989578
Syt12	0.419179259	0.49848751	0.302345719
Hcrtr1	-0.692649847	0.500724451	0.300401201
Nell1	-0.892998485	0.500724451	0.300401201
Dlx1as	-0.597685632	0.500724451	0.300401201
Frmd4b	-0.582656733	0.500724451	0.300401201
Ube2ql1	-0.38527668	0.500724451	0.300401201
Cck	2.405014191	0.500724451	0.300401201
Clec1b	0.426412794	0.500724451	0.300401201
Zwilch	-0.299843336	0.500724451	0.300401201
Lefty1	0.354369883	0.500724451	0.300401201
Gfra1	-0.275366786	0.500724451	0.300401201
Nwd2	-0.24209965	0.500724451	0.300401201
Rrad	-0.219848149	0.500724451	0.300401201
Git1	0.150445292	0.500724451	0.300401201
Slc25a24	-0.31241046	0.500724451	0.300401201
Mycl	-0.23692931	0.500724451	0.300401201
Trim37	0.238926393	0.500724451	0.300401201
Mcpt4	-0.164553159	0.500724451	0.300401201
Col11a1	0.209533138	0.500724451	0.300401201
Pip5k1c	0.198591305	0.500724451	0.300401201
Slc39a10	-0.139151591	0.500724451	0.300401201
FAM129B	-0.216476074	0.500724451	0.300401201
Slc5a7	0.175276807	0.500724451	0.300401201
Htr7	-0.421860018	0.500724451	0.300401201
Mefv	0.141661223	0.500724451	0.300401201
Hs3st2	0.276937588	0.500724451	0.300401201
Rnase1	0.371719977	0.500724451	0.300401201
Arid5a	-0.20520564	0.500724451	0.300401201
Ncan	0.174134159	0.500724451	0.300401201
Fam135b	-0.140964961	0.500724451	0.300401201
Sstr3	0.228437051	0.500724451	0.300401201
Btbd8	0.209699511	0.500724451	0.300401201
Cmpk2	-0.321741522	0.500724451	0.300401201
Gpc2	0.16033158	0.500724451	0.300401201
Vmn2r84	-0.47687394	0.500724451	0.300401201
Tmem125	-0.276875212	0.500724451	0.300401201
Des	0.138628304	0.500724451	0.300401201
Nras	0.223988791	0.500724451	0.300401201
Slc22a4	0.178238236	0.500724451	0.300401201
Serpind1	0.164108984	0.500724451	0.300401201
Rn7s2	0.127784873	0.500724451	0.300401201
Rad51ap1	0.15546229	0.500724451	0.300401201
Dab2ip	0.125845041	0.500724451	0.300401201
Slco1a5	0.200986206	0.500724451	0.300401201

Mrps6	0.179882086	0.500724451	0.300401201
Peli3	-0.229362435	0.500724451	0.300401201
Ocm	0.147626283	0.500724451	0.300401201
Rnaseh2b	-0.37422403	0.500724451	0.300401201
Ogn	0.212330143	0.500724451	0.300401201
Tcea3	0.144746619	0.500724451	0.300401201
Aqp9	0.133664044	0.500724451	0.300401201
Map4k4	0.136037177	0.500724451	0.300401201
1-Mar	0.170439439	0.500724451	0.300401201
Atp7a	0.154498069	0.500724451	0.300401201
FAM57B	0.274282669	0.500724451	0.300401201
Dscc1	0.438306944	0.500724451	0.300401201
Oas1c	0.28169736	0.500724451	0.300401201
Slc17a9	-0.158219833	0.500724451	0.300401201
Dlgap1	0.221974454	0.500724451	0.300401201
Ampd3	-0.211159112	0.500724451	0.300401201
Npr3	0.184032492	0.500724451	0.300401201
Ankrd2	0.188230744	0.500724451	0.300401201
Hmgcs2	0.23143227	0.500724451	0.300401201
Pgr	0.142771444	0.500724451	0.300401201
Lbh	0.208963467	0.500724451	0.300401201
Coro2a	0.159245312	0.500724451	0.300401201
Pcdhb8	0.154462431	0.502314048	0.299024676
Atrnl1	0.154329447	0.506008852	0.295841885
Olfr1420	-0.600170338	0.509201605	0.293110236
Rgl3	0.188537265	0.509201605	0.293110236
Zfpm2	0.150214385	0.513272237	0.289652226
Nfkb1	-0.202366728	0.513272237	0.289652226
Pth1r	0.157682376	0.515207079	0.288018179
Apcdd1	-0.373377162	0.517692772	0.285927899
Prkd2	-0.243497478	0.517692772	0.285927899
Gyg	0.313561518	0.517846146	0.285799252
HIST2H2A/	0.178683195	0.518778675	0.285017884
Sod3	-0.607112799	0.519161295	0.284697693
Tmem132	-0.584083117	0.519790337	0.284171798
Car15	0.493768151	0.52037684	0.28368204
Mitd1	-0.166788077	0.52037684	0.28368204
Hr	0.140666527	0.52037684	0.28368204
Ccdc190	0.113186834	0.52037684	0.28368204
Pla2g2d	0.136611947	0.524521888	0.280236384
FAM109B	0.282632741	0.524521888	0.280236384
Kcnh1	0.157071415	0.524521888	0.280236384
Cd34	0.21172679	0.524521888	0.280236384
Rell2	0.241316536	0.52526897	0.279618254
Ank1	0.112506169	0.525742135	0.279227216
Sptbn2	0.353531933	0.525742135	0.279227216
Fkbp1b	0.370601078	0.525742135	0.279227216
Man1c1	0.156231765	0.52590989	0.279088662

Fam71f2	-0.13072162	0.52590989	0.279088662
Coro1b	0.178435624	0.52590989	0.279088662
Lmod2	0.132085377	0.52590989	0.279088662
Dennd5b	0.294096826	0.526310212	0.278758203
Angpt2	0.259435396	0.526310212	0.278758203
Rnf152	-0.265576348	0.526310212	0.278758203
Glrp1	0.217958687	0.526310212	0.278758203
Slc23a3	0.114783448	0.526310212	0.278758203
Gabrb2	0.179276036	0.526310212	0.278758203
Cndp2	0.344813786	0.526310212	0.278758203
Gnai3	-0.147965351	0.526310212	0.278758203
Cacna1a	0.19656349	0.526310212	0.278758203
Klhdc7b	0.139539482	0.526310212	0.278758203
Wnk2	0.221630067	0.526310212	0.278758203
Crygn	0.222009455	0.526310212	0.278758203
Slc28a2	0.101309867	0.526310212	0.278758203
Camk2a	-0.116569536	0.526310212	0.278758203
Sirpb1b	0.189933236	0.526310212	0.278758203
Btbd11	-0.211641002	0.526310212	0.278758203
Galnt10	0.27746832	0.526310212	0.278758203
Myl9	0.163284302	0.526310212	0.278758203
Rbp7	0.170064741	0.526310212	0.278758203
Lnx2	0.238236457	0.526310212	0.278758203
Etv1	-0.209203344	0.526310212	0.278758203
Pacsin1	0.417179657	0.528194337	0.27720626
Spata6	0.168165985	0.53086585	0.275015211
Ryr2	0.255225576	0.53086585	0.275015211
Arrdc4	0.125216184	0.535831261	0.270971953
Slc8a1	-0.823695515	0.538444385	0.268859148
Lilra5	-0.74882074	0.538444385	0.268859148
Kcnh3	-0.806108537	0.538444385	0.268859148
Sox4	-0.631669404	0.538444385	0.268859148
Prkcd	-0.540535652	0.538444385	0.268859148
Gpr162	-0.330475231	0.538444385	0.268859148
Rnh1	-0.410011781	0.538444385	0.268859148
Cort	-0.339953159	0.538444385	0.268859148
Ina	-0.327481415	0.538444385	0.268859148
Prr5l	-0.53265329	0.538444385	0.268859148
MDRL	-0.505893221	0.538444385	0.268859148
Col8a1	-0.545046511	0.538444385	0.268859148
Cap2	0.122869274	0.538444385	0.268859148
Epha4	-0.206709827	0.538444385	0.268859148
Tmem198	-0.307338479	0.538444385	0.268859148
Gxylt2	0.167473461	0.538444385	0.268859148
Ccl27a	0.215376221	0.538444385	0.268859148
Cx3cl1	0.187514745	0.538444385	0.268859148
Camk2g	-0.540371032	0.538444385	0.268859148
Ascl1	0.113256204	0.538444385	0.268859148

AW549542	0.208924078	0.538444385	0.268859148
Neurl2	-0.125302028	0.538444385	0.268859148
Serpinb1a	0.262001876	0.538444385	0.268859148
Scarna6	0.155024292	0.538444385	0.268859148
HIST1H4J	-0.150404746	0.538444385	0.268859148
Tmem181	0.390660777	0.538444385	0.268859148
Inca1	0.162068201	0.538444385	0.268859148
Arntl	0.548816723	0.538444385	0.268859148
Nup43	0.191877121	0.538444385	0.268859148
Tal1	0.117367963	0.538444385	0.268859148
Igsf9b	0.160552308	0.538444385	0.268859148
Nap1l2	0.173345906	0.538444385	0.268859148
Lrrc7	0.161806221	0.538444385	0.268859148
Panx2	0.165188689	0.538444385	0.268859148
Papss2	-0.248952178	0.538444385	0.268859148
Cd1d1	0.182219472	0.538444385	0.268859148
Hspa12a	0.164009679	0.538444385	0.268859148
Inppl1	0.248902977	0.538444385	0.268859148
Clec9a	-0.12344178	0.538444385	0.268859148
Klhl40	0.17105529	0.538444385	0.268859148
Lpp	-0.108258557	0.538444385	0.268859148
Npc1	0.127431193	0.538444385	0.268859148
Fignl2	0.181639987	0.538444385	0.268859148
Gabrd	-0.277457224	0.538444385	0.268859148
Atcay	-0.235716614	0.538444385	0.268859148
Stamos	0.121039341	0.538444385	0.268859148
Slc30a3	0.170740204	0.538444385	0.268859148
Slc10a3	-0.158938391	0.538444385	0.268859148
Kcnc1	0.217662715	0.538444385	0.268859148
Npnt	0.26564923	0.538444385	0.268859148
Fmnl2	0.109885162	0.538444385	0.268859148
Lig1	-0.166170341	0.538444385	0.268859148
ZCCHC16	0.234599179	0.538444385	0.268859148
Mob3c	0.137091696	0.538444385	0.268859148
Plod3	0.151922317	0.538444385	0.268859148
Rab6b	0.179499689	0.538444385	0.268859148
Sox14	0.111684096	0.538444385	0.268859148
Tmod3	0.199939209	0.538444385	0.268859148
Cenpk	-0.261648162	0.538444385	0.268859148
5-Sep	0.248821205	0.538444385	0.268859148
Bbc3	0.179568901	0.538444385	0.268859148
Adamts6	0.113382094	0.538444385	0.268859148
Slc26a8	0.280679186	0.538444385	0.268859148
Scara3	-0.126561858	0.538444385	0.268859148
Ankrd24	-0.096986227	0.538444385	0.268859148
Rab37	-0.274152359	0.538444385	0.268859148
Pcdh7	0.169915903	0.538444385	0.268859148
Traf4	-0.223011441	0.538444385	0.268859148

Ckmt1	0.117161182	0.538444385	0.268859148
Fam78b	0.167769453	0.538444385	0.268859148
Khdrbs3	0.126110446	0.538444385	0.268859148
Gzmm	0.226698114	0.538444385	0.268859148
Dnajc6	0.184802148	0.538444385	0.268859148
Prickle1	-0.159089243	0.538444385	0.268859148
Foxc1	0.221342007	0.538444385	0.268859148
Tspyl5	0.158358763	0.538444385	0.268859148
Creb3l2	-0.118337646	0.538444385	0.268859148
Cenpj	0.379477357	0.538444385	0.268859148
Snhg9	0.176600337	0.538444385	0.268859148
Parm1	0.146377137	0.538444385	0.268859148
Pou3f2	-0.515750232	0.538935882	0.268462901
Mst1r	0.179383992	0.538935882	0.268462901
Leprot	0.483481428	0.538935882	0.268462901
GATSL2	-0.817055639	0.540252078	0.267403554
Adgrl4	-0.461367957	0.540252078	0.267403554
Osbpl6	-0.159150267	0.540252078	0.267403554
Thoc6	-0.257043294	0.540252078	0.267403554
Kcnj10	0.359673675	0.540252078	0.267403554
Nat8f3	-0.139783168	0.540252078	0.267403554
Hmga2	0.203437617	0.540252078	0.267403554
Rps9	0.160896821	0.540252078	0.267403554
Setbp1	0.149812737	0.540252078	0.267403554
Slc22a12	-0.150273678	0.540252078	0.267403554
Sox11	0.446557004	0.540252078	0.267403554
Tssc4	0.138829124	0.540252078	0.267403554
Lrfn5	0.127892976	0.540252078	0.267403554
Trem1	0.195162871	0.54086523	0.266910937
Hgf	-0.616766029	0.54237532	0.26570008
Emx1	0.537045165	0.543142019	0.265086598
Frmpd3	0.182866469	0.543142019	0.265086598
Clvs1	0.254987804	0.543142019	0.265086598
Brsk2	0.336099537	0.543142019	0.265086598
L3mbtl1	-0.156828283	0.543142019	0.265086598
Kcnn1	-0.265412896	0.543142019	0.265086598
Nr1h3	0.157301785	0.545217125	0.263430512
Cyp27a1	-0.165728041	0.546540499	0.262377651
Bean1	0.131055037	0.546730388	0.262226787
Arsk	0.951549011	0.550177794	0.259496943
TMEM206	-0.184576273	0.550177794	0.259496943
Cdhr1	0.406310736	0.550177794	0.259496943
Extl1	-0.278662648	0.550177794	0.259496943
Crygs	0.225272135	0.550177794	0.259496943
Vav2	0.145304566	0.550177794	0.259496943
Zbtb40	0.179848472	0.550177794	0.259496943
Kazald1	0.231129728	0.550177794	0.259496943
Ntrk3	-0.235928214	0.550177794	0.259496943

Mylpf	0.255542992	0.550177794	0.259496943
Garnl3	0.292172004	0.550177794	0.259496943
Crot	-0.400338127	0.550177794	0.259496943
Pabpc5	-0.159568713	0.550177794	0.259496943
Trpc3	0.166208045	0.550177794	0.259496943
Fam72a	0.152382916	0.550177794	0.259496943
Dmtn	0.182358819	0.550177794	0.259496943
Stard4	0.16170183	0.550177794	0.259496943
Tmprss11a	0.178609336	0.550177794	0.259496943
Zfp711	-0.177366972	0.550177794	0.259496943
Srsf9	0.146856806	0.550177794	0.259496943
AW82225	0.152524661	0.550177794	0.259496943
Kcnmb4	-0.459253099	0.550840256	0.258974328
Cln3	-0.436127767	0.550840256	0.258974328
Vkorc1	0.278862997	0.550840256	0.258974328
Id1	-0.25395195	0.550966401	0.258874884
Hapln2	-0.274077974	0.550966401	0.258874884
Camta2	0.149836605	0.550966401	0.258874884
Cd163	-0.30115441	0.550966401	0.258874884
Tmem44	0.12159711	0.550966401	0.258874884
Mos	0.140223324	0.550966401	0.258874884
Lsm11	0.348499906	0.553023555	0.25725637
Lonrf3	-0.584199093	0.554845075	0.255828265
Nhlh2	-0.337900067	0.556224551	0.254749846
Itpkb	-0.244832977	0.556224551	0.254749846
As3mt	-0.283562895	0.556224551	0.254749846
Cnn1	0.203230596	0.556224551	0.254749846
Siglece	-0.320991691	0.556224551	0.254749846
Rep15	0.153582954	0.556224551	0.254749846
Kcnj14	0.219929753	0.556224551	0.254749846
Glod5	0.148392474	0.556224551	0.254749846
STRA13	0.224391889	0.556224551	0.254749846
Strbp	0.237303078	0.556224551	0.254749846
Tmcc3	0.157419075	0.556224551	0.254749846
Clec18a	0.370884453	0.556224551	0.254749846
Wfikkn1	-0.110082854	0.556224551	0.254749846
Mcoln3	0.222266311	0.556224551	0.254749846
Fdft1	-0.187699623	0.556224551	0.254749846
Pipox	0.17224704	0.556224551	0.254749846
Ngfr	0.111330184	0.556224551	0.254749846
Dctpp1	0.509644985	0.556224551	0.254749846
Chrna3	0.344541902	0.556224551	0.254749846
Il11ra2	-0.443341303	0.557909073	0.253436576
Rn7s1	-0.652033572	0.559057568	0.252543469
Lypd6b	-0.294585978	0.559057568	0.252543469
Snurf	-0.603139776	0.559057568	0.252543469
Msx2	-0.545810503	0.559057568	0.252543469
Ddx43	0.224091691	0.559057568	0.252543469

Tiparp	-0.251601142	0.559057568	0.252543469
Pld2	-0.177416287	0.559057568	0.252543469
Rbfox3	-0.206416276	0.559057568	0.252543469
Sgsh	0.234078089	0.559057568	0.252543469
Cyp4x1	0.378688098	0.559057568	0.252543469
Rgn	0.088167648	0.559057568	0.252543469
Bok	0.106905124	0.559057568	0.252543469
Sox3	-0.25001145	0.559057568	0.252543469
Slc10a7	0.457025746	0.559057568	0.252543469
Soga1	-0.120678729	0.559057568	0.252543469
P3h1	0.127840031	0.559057568	0.252543469
Idh1	0.24155128	0.559057568	0.252543469
Col6a5	-0.219934447	0.559057568	0.252543469
Ly75	0.197968063	0.559057568	0.252543469
Rad51c	0.151955766	0.559057568	0.252543469
Fam217b	-0.142340712	0.559057568	0.252543469
Atp2a2	-0.168781427	0.559057568	0.252543469
Pcdhb2	0.360109997	0.559057568	0.252543469
Kitl	-0.119484513	0.559057568	0.252543469
Tmem145	0.172013833	0.559057568	0.252543469
Zfp365	0.152547582	0.559057568	0.252543469
Rasgrp1	-0.396244878	0.559057568	0.252543469
Prdx4	0.216324521	0.559057568	0.252543469
Efcab10	0.309104259	0.559057568	0.252543469
Prokr1	0.159801908	0.559057568	0.252543469
Fam234a	0.335831287	0.559057568	0.252543469
Nqo1	-0.210907777	0.559057568	0.252543469
Ybx1	0.128244375	0.559057568	0.252543469
Arhgap32	0.091482355	0.559057568	0.252543469
Kpna2	0.280085786	0.559057568	0.252543469
Rnf43	0.239497652	0.559057568	0.252543469
Eef1a2	0.232013802	0.559057568	0.252543469
Fgf23	0.190371707	0.559057568	0.252543469
Vipr1	0.175520032	0.559057568	0.252543469
Atxn7l3	0.207049741	0.559057568	0.252543469
Penk	0.144501796	0.559057568	0.252543469
Pole3	0.450682039	0.559877296	0.251907143
Gpr6	0.104270793	0.559877296	0.251907143
Qk	0.168594975	0.560521284	0.251407892
Tstd1	-0.199396416	0.560521284	0.251407892
Defb9	-0.359238953	0.560521284	0.251407892
Ttyh2	0.148832238	0.560521284	0.251407892
Sipa1l1	-0.14009856	0.560521284	0.251407892
Fosl1	0.161648257	0.560521284	0.251407892
Osbpl11	0.196960971	0.560521284	0.251407892
Pm20d1	-0.12106713	0.560521284	0.251407892
Syne1	-0.178558896	0.560764409	0.251219558
Ascl2	0.104438371	0.560764409	0.251219558

Zfp691	-0.101763699	0.561984294	0.250275822
Pgm2l1	0.489814328	0.563170711	0.24935994
Tekt4	-0.428056923	0.563170711	0.24935994
Sox8	-0.630102645	0.564911459	0.248019616
Cd6	0.121255892	0.564911459	0.248019616
Dpp4	0.191137587	0.564911459	0.248019616
Slc1a3	-0.188188802	0.564911459	0.248019616
Lix1l	0.148458234	0.564911459	0.248019616
Lgi1	0.168834679	0.564911459	0.248019616
Stx2	0.356889427	0.564911459	0.248019616
Pxmp2	0.200884013	0.564911459	0.248019616
Psmc3ip	0.218103544	0.565580233	0.247505777
Krtcap2	0.181910128	0.565580233	0.247505777
Csk	0.167284256	0.566464574	0.246827246
Ppp2r1b	-0.390255252	0.568228145	0.245477259
Igsf11	-0.52851268	0.568228145	0.245477259
Klc3	-0.220501022	0.568228145	0.245477259
Sult4a1	-0.520995514	0.568228145	0.245477259
Nhsl1	-0.130611096	0.568228145	0.245477259
Tnfsf10	-0.130905117	0.568228145	0.245477259
Ppp1r3c	0.105760854	0.568228145	0.245477259
Clstn1	0.152179004	0.568228145	0.245477259
Otub2	-0.196104983	0.568228145	0.245477259
Foxo1	-0.111501188	0.568228145	0.245477259
Lncpint	-0.212683515	0.568228145	0.245477259
ERBB2IP	0.143753619	0.568228145	0.245477259
Gabre	-0.199745017	0.568228145	0.245477259
Ly6g6d	0.139891518	0.568228145	0.245477259
Slc12a5	-0.508454065	0.571093191	0.243293018
Sytl2	-0.348921467	0.571093191	0.243293018
Cfap61	0.240357648	0.571093191	0.243293018
Nptx2	-0.183460859	0.571093191	0.243293018
Aak1	-0.191012484	0.571093191	0.243293018
Arap3	-0.103853703	0.571093191	0.243293018
Anxa9	0.142167755	0.571093191	0.243293018
Glb1l	0.182466106	0.571093191	0.243293018
Phldb2	-0.133294945	0.571093191	0.243293018
Myo5a	-0.136131513	0.571093191	0.243293018
Rbp4	0.188494646	0.571093191	0.243293018
Apaf1	-0.209554942	0.571093191	0.243293018
Mast2	0.199887483	0.571093191	0.243293018
Sorcs3	-0.109300629	0.571093191	0.243293018
Lrig1	-0.187047529	0.571093191	0.243293018
Jdp2	-0.286646748	0.571093191	0.243293018
Pgpep1	0.15379498	0.571093191	0.243293018
Crlf2	-0.083669237	0.571093191	0.243293018
Tnfrsf22	0.268561238	0.571093191	0.243293018
Smtn	-0.141291362	0.571093191	0.243293018

Tmem63c	-0.137287418	0.571093191	0.243293018
Asb10	0.290652918	0.573049396	0.241807941
Srebf2	0.150705459	0.573283051	0.241630898
Ephx1	-0.20783287	0.573283051	0.241630898
Adssl1	0.15693528	0.573283051	0.241630898
Nod1	0.116844389	0.573283051	0.241630898
Dalir	0.166779014	0.5739888	0.241096582
Tex11	0.133762393	0.574301095	0.240860356
Rorb	-0.383898854	0.575024354	0.240313761
15-Sep	0.3063839	0.576808317	0.238968486
Zfp109	0.194148445	0.577505141	0.238444145
Faim2	0.293954116	0.577617821	0.238359416
MSX1OS	-0.320874732	0.58002922	0.236550127
Lrfn2	-0.312079848	0.58002922	0.236550127
Ppfia3	-0.444403844	0.58002922	0.236550127
Tor1aip1	-0.29459207	0.58002922	0.236550127
Lrrc38	-0.114623092	0.58002922	0.236550127
Asap2	0.124569679	0.58002922	0.236550127
Bend5	0.248316846	0.58002922	0.236550127
Glis3	0.124831384	0.58002922	0.236550127
Mpp3	0.188640792	0.58002922	0.236550127
Ntpcr	-0.153498144	0.58002922	0.236550127
Unc80	-0.115070989	0.58002922	0.236550127
Pml	0.109013326	0.580227246	0.236401882
Hmgcs1	-0.439598287	0.58081747	0.23596033
Herc3	0.200226424	0.58081747	0.23596033
Ccdc62	0.260520386	0.58081747	0.23596033
Lgals8	-0.20400658	0.58081747	0.23596033
Ccdc13	-0.152031085	0.58081747	0.23596033
Pcsk2	0.146994328	0.58081747	0.23596033
Lgr5	-0.128689047	0.58081747	0.23596033
Enc1	0.164669729	0.58081747	0.23596033
Spred3	0.251516283	0.58081747	0.23596033
DEAR1	0.102735219	0.58081747	0.23596033
Syt16	0.139226287	0.58081747	0.23596033
Fibin	0.147536617	0.58081747	0.23596033
Myadml2	0.257231438	0.58081747	0.23596033
Lsm5	-0.100178028	0.582436914	0.234751108
Il17rc	0.204900354	0.582436914	0.234751108
Itpka	0.114763287	0.582643099	0.234597393
HIST1H4D	-0.35209553	0.583250532	0.234144857
Ticrr	-0.310011313	0.583250532	0.234144857
Ccnf	-0.163065583	0.583250532	0.234144857
Syce2	0.15299974	0.583250532	0.234144857
Rftn1	-0.235160909	0.583250532	0.234144857
Mex3a	-2.000818978	0.583250532	0.234144857
Dock1	0.177350313	0.583250532	0.234144857
Btbd10	0.161998422	0.583250532	0.234144857

Wipi1	0.155245247	0.583250532	0.234144857
Cldn19	0.148250318	0.583250532	0.234144857
Csmd2	-0.292301804	0.583250532	0.234144857
Syt1	0.280961958	0.583250532	0.234144857
Fgf2	0.431601371	0.583250532	0.234144857
Cytl1	-0.209082942	0.583250532	0.234144857
Cdhr4	0.16232968	0.583250532	0.234144857
Ptprz1	0.167248021	0.583250532	0.234144857
Pak1	-0.102739911	0.583250532	0.234144857
Adgrb1	-0.349585929	0.583250532	0.234144857
Tspan15	0.095692643	0.583250532	0.234144857
Prmt8	-0.370266156	0.583250532	0.234144857
Spns3	0.321095592	0.583250532	0.234144857
Cdk5rap2	-0.144168885	0.583250532	0.234144857
Nudt1	-0.142586311	0.583250532	0.234144857
Slc25a21	0.116525228	0.583250532	0.234144857
Rpl18	0.271643799	0.583250532	0.234144857
Rnf157	0.110210667	0.583250532	0.234144857
Zfp273	0.11934449	0.583250532	0.234144857
Fam205a3	0.178589824	0.583250532	0.234144857
Fsd2	0.230830595	0.583250532	0.234144857
Rps12	-0.290120297	0.583250532	0.234144857
Kcnip4	0.280788723	0.583250532	0.234144857
Npm3	0.105738942	0.583250532	0.234144857
Ano6	0.136280175	0.583250532	0.234144857
Pclo	0.125180984	0.583250532	0.234144857
Kif5c	0.245525575	0.583250532	0.234144857
Ccdc136	0.264396193	0.583250532	0.234144857
Rnase6	-0.529293262	0.583250532	0.234144857
Il1rapl1	0.20481785	0.583250532	0.234144857
Myl2	0.147749731	0.583250532	0.234144857
Ttll11	0.178014757	0.583250532	0.234144857
Swap70	0.269404105	0.584336497	0.233336987
Sstr4	0.150742711	0.584336497	0.233336987
Elf5	-0.248578955	0.584336497	0.233336987
Cnp	-0.360329325	0.585046371	0.23280971
Map7d2	0.228791929	0.585046371	0.23280971
Hopx	0.510915737	0.585046371	0.23280971
Cth	-0.511774719	0.585511478	0.232464587
Fzd10	-0.643234042	0.585511478	0.232464587
Mapk8	-0.54055021	0.585511478	0.232464587
HIST4H4	-0.220820148	0.585511478	0.232464587
Rtbdn	-0.341043012	0.585511478	0.232464587
H6pd	-0.137778455	0.585511478	0.232464587
Srms	-0.1932799	0.585511478	0.232464587
AI467606	-0.28556757	0.585511478	0.232464587
Nkx2-9	-0.198166982	0.585511478	0.232464587
Amigo1	-0.178318382	0.585511478	0.232464587

Ccdc78	-0.23810638	0.585511478	0.232464587
Msx1	0.162730393	0.585511478	0.232464587
Fkbp9	-0.119306952	0.585511478	0.232464587
Astn2	0.295997748	0.585511478	0.232464587
Gpr21	0.241604565	0.585511478	0.232464587
Cd81	0.221062213	0.585511478	0.232464587
Tbc1d2b	0.132736151	0.585511478	0.232464587
Trp53cor1	0.273993778	0.585511478	0.232464587
Bmpr1b	0.131912539	0.585511478	0.232464587
Tcf7l1	0.27630302	0.585511478	0.232464587
Sv2b	0.129941881	0.585511478	0.232464587
Sec61b	-0.143494628	0.585511478	0.232464587
St6gal2	-0.104666283	0.585511478	0.232464587
Folh1	0.162613932	0.585511478	0.232464587
Mtmr11	0.194061125	0.585511478	0.232464587
Bicc1	0.346031626	0.585511478	0.232464587
Flrt1	-0.269107423	0.585511478	0.232464587
Tmed3	0.101573196	0.585511478	0.232464587
Psat1	0.135178877	0.585511478	0.232464587
Cald1	0.268218209	0.586043888	0.232069859
Elovl6	0.172566928	0.58658232	0.231671031
Ccer2	0.108534882	0.586615833	0.23164622
Col9a3	-0.124869183	0.586615833	0.23164622
Tpt1	-0.27344937	0.586947562	0.231400697
Raph1	0.306437958	0.586947562	0.231400697
Cdkn1a	0.115768377	0.586957426	0.231393398
Sdcbp2	0.126825781	0.587472685	0.231012322
Nkx6-2	0.194630833	0.587555417	0.230951165
Actr6	0.250826629	0.588941563	0.229927795
Accs	0.233955048	0.589310003	0.229656187
Terc	0.164331955	0.589428206	0.229569086
Klhl33	-0.174814403	0.590227189	0.228980788
Zfr2	0.103850346	0.590238886	0.228972182
Kcne4	0.214890356	0.590238886	0.228972182
Mybl2	-0.146226947	0.590623905	0.228688979
Slc9a9	0.143918373	0.590962608	0.228439997
Myh7b	0.150611341	0.59109244	0.228344595
Gabrg2	0.152770708	0.591193801	0.228270128
Dagla	-0.497004887	0.591288159	0.228200818
Cpne2	0.28708464	0.591288159	0.228200818
Phldb3	0.330555591	0.591288159	0.228200818
Lrrc6	-0.409619862	0.593200856	0.226798231
Serpina9	0.338228054	0.593200856	0.226798231
Eya4	0.12517515	0.593200856	0.226798231
Serpina3i	0.116417352	0.593200856	0.226798231
P2ry14	0.253188697	0.593200856	0.226798231
Surf4	0.116988456	0.595689265	0.224980226
Ech1	-0.11955825	0.5960928	0.224686124

Dpyd	0.210297278	0.5960928	0.224686124
Vopp1	0.090820781	0.5960928	0.224686124
Csrnp3	0.108046972	0.5960928	0.224686124
Prr33	-0.69568593	0.598475936	0.222953308
Kcnab2	0.179564946	0.598475936	0.222953308
Adap1	0.193706887	0.598475936	0.222953308
Nucb2	0.284599188	0.598475936	0.222953308
Suclg2	0.239542004	0.598475936	0.222953308
Cadps	0.138965814	0.598475936	0.222953308
AA986860	-0.172460952	0.598475936	0.222953308
Icam2	-0.166028623	0.598995195	0.222576662
Nsmce1	0.293417349	0.598995195	0.222576662
Myrf	0.263790814	0.598995195	0.222576662
Stxbp3	-0.637581036	0.599563526	0.222164795
Map3k9	-0.280226264	0.599563526	0.222164795
Peg10	0.110634707	0.599563526	0.222164795
Pde4c	-0.17617565	0.599563526	0.222164795
Cox6b2	0.196839206	0.599563526	0.222164795
H2-Q2	0.25923694	0.599563526	0.222164795
Asic2	0.109282409	0.599563526	0.222164795
Car2	0.111829877	0.599563526	0.222164795
Decr1	0.199397172	0.599563526	0.222164795
Mtfr2	-0.175076342	0.599563526	0.222164795
Nt5dc1	0.195505334	0.599563526	0.222164795
Brsk1	-0.142879623	0.599563526	0.222164795
Hs6st3	0.12690768	0.599563526	0.222164795
Fos	0.235564975	0.599563526	0.222164795
Hnrnpf	0.186237486	0.599563526	0.222164795
Faah	0.567623433	0.599563526	0.222164795
Fcer2a	0.304961973	0.599563526	0.222164795
Dock11	0.169424837	0.599563526	0.222164795
Nxn	0.211155823	0.599563526	0.222164795
Ghdc	-0.119749907	0.599563526	0.222164795
LARGE	0.134765212	0.599563526	0.222164795
Tjp3	0.216637621	0.599563526	0.222164795
Pwwp2b	-0.272831431	0.600297335	0.221633585
Lpo	0.545922927	0.600297335	0.221633585
Rassf3	-0.307880272	0.600297335	0.221633585
Peg12	0.188051586	0.600297335	0.221633585
Thsd1	-0.092977599	0.600297335	0.221633585
Hipk4	0.366048836	0.600297335	0.221633585
Il27ra	0.264210831	0.600297335	0.221633585
Galnt3	0.14590639	0.600297335	0.221633585
Cdkn1c	0.129671693	0.600297335	0.221633585
Grm4	0.258165766	0.600297335	0.221633585
Rai2	0.14485494	0.60074405	0.221310522
Faxc	0.106552399	0.60074405	0.221310522
Pcdhga7	0.236668078	0.60074405	0.221310522

Fn3k	-0.243408159	0.601185409	0.220991569
Arhgap26	0.163516695	0.601185409	0.220991569
Sgpp2	0.258664242	0.601185409	0.220991569
Hspa1l	0.167279254	0.601185409	0.220991569
Pdia4	0.276346821	0.601185409	0.220991569
Car4	-0.246314775	0.601185409	0.220991569
Snora73a	-0.082938521	0.601185409	0.220991569
Bhlhe41	0.209416772	0.601185409	0.220991569
Csrnp1	0.210460325	0.601185409	0.220991569
Kcnc4	-0.269151896	0.601185409	0.220991569
Tril	-0.404893325	0.601186249	0.220990961
Klhl34	0.21183936	0.601186249	0.220990961
Calcrl	-0.27184247	0.601186249	0.220990961
Pitpnm3	0.275260945	0.601186249	0.220990961
Gabarap	-0.343598752	0.601186249	0.220990961
Ska1	0.322391474	0.601186249	0.220990961
Sugct	0.269817405	0.601186249	0.220990961
Otop2	-0.194657935	0.601186249	0.220990961
Nrg3	-0.143042236	0.601186249	0.220990961
Itm2b	0.187971709	0.601186249	0.220990961
Ikbip	-0.087277423	0.601186249	0.220990961
Trip6	0.097914928	0.601186249	0.220990961
Pih1d2	0.154590556	0.601186249	0.220990961
Lrrc4	-0.119540079	0.601186249	0.220990961
Tenm2	0.133437274	0.601243614	0.220949524
Dkk3	-0.369062997	0.601288687	0.220916967
Dusp14	1.805779093	0.601288687	0.220916967
Lrrk2	-0.144205778	0.601288687	0.220916967
Resp18	-0.18501582	0.601288687	0.220916967
Arhgdig	-0.162966724	0.601288687	0.220916967
Plxna4	0.344510091	0.601288687	0.220916967
Fam205a4	-0.570961235	0.601288687	0.220916967
Fam163b	0.197271431	0.601288687	0.220916967
Rhbdd1	0.326930527	0.601288687	0.220916967
Nme9	-0.186564636	0.601288687	0.220916967
Ralb	0.155976576	0.601288687	0.220916967
Kin	0.09957856	0.601288687	0.220916967
Shd	0.211786759	0.601288687	0.220916967
Lingo4	-0.207770137	0.601288687	0.220916967
Cat	-0.111633128	0.601288687	0.220916967
Gimap5	0.119400577	0.601288687	0.220916967
Snhg1	-0.158327511	0.601288687	0.220916967
Tspyl4	0.322860007	0.601288687	0.220916967
Chn1	0.115451113	0.601288687	0.220916967
Nr0b1	0.100149229	0.601288687	0.220916967
Hexim2	0.202404149	0.601288687	0.220916967
Klhl3	0.240356153	0.601288687	0.220916967
Rab26os	-0.175227983	0.601288687	0.220916967

Nupr1l	-0.188608484	0.601288687	0.220916967
Clstn3	0.120763178	0.601288687	0.220916967
Cdh5	0.121998327	0.601288687	0.220916967
Nnat	0.114831856	0.601288687	0.220916967
Tmem144	0.110668793	0.601288687	0.220916967
Ccser1	0.355898642	0.601288687	0.220916967
Fanci	-0.168985632	0.601288687	0.220916967
Tmem59l	0.130763643	0.601423701	0.220819461
Slc27a5	0.174492738	0.601696127	0.220622784
Tmprss6	0.231023042	0.604363106	0.218702055
Sorbs3	-0.429822419	0.605055868	0.218204523
Itih3	0.433512388	0.605055868	0.218204523
Ppip5k1	0.168999682	0.605055868	0.218204523
Cdyl2	0.194760842	0.605055868	0.218204523
Nmral1	-0.125477005	0.605055868	0.218204523
Zfyve9	0.129553449	0.605055868	0.218204523
Sik1	-0.186880162	0.605055868	0.218204523
Smim18	0.138244584	0.605055868	0.218204523
Gab2	0.164024178	0.605055868	0.218204523
Sdk2	0.152448458	0.605055868	0.218204523
Srrm4	0.154230517	0.605055868	0.218204523
Ppp1r14a	0.083401994	0.605055868	0.218204523
Nudt17	0.345431894	0.605055868	0.218204523
Galr2	0.256099561	0.605055868	0.218204523
Rfc5	0.457426833	0.605055868	0.218204523
Mavs	0.202924036	0.605055868	0.218204523
Zfp947	0.232435889	0.605055868	0.218204523
Hspa4l	0.118189689	0.605055868	0.218204523
Manba	0.166719139	0.605055868	0.218204523
Acy3	0.118898379	0.605055868	0.218204523
Scai	-0.137419913	0.605055868	0.218204523
Prkcb	-0.088752329	0.605055868	0.218204523
Pde4a	0.145934167	0.605055868	0.218204523
Zdhhc8	0.123889359	0.605055868	0.218204523
Homer2	-0.121128424	0.605055868	0.218204523
Mob3a	0.098370328	0.605055868	0.218204523
Gjb3	0.157734074	0.605055868	0.218204523
Ccl24	-0.191407133	0.605340424	0.218000324
Olfr288	-0.318328929	0.605709137	0.217735875
Dapk2	-0.125277056	0.605709137	0.217735875
Crabp2	-0.091225995	0.605709137	0.217735875
Stxbp6	-0.162267226	0.605709137	0.217735875
Ttbk1	-0.193517815	0.606424371	0.217223353
Ttc39b	-0.542997074	0.606505866	0.217164994
GATSL2	0.176963517	0.606505866	0.217164994
Dgkz	0.116117949	0.606505866	0.217164994
Vwa1	-0.379841184	0.606797927	0.216955912
Ngef	-0.119540874	0.606797927	0.216955912

Pbp2	0.118775566	0.606797927	0.216955912
Dmxl2	-0.184606739	0.607333221	0.216572963
Pknox2	0.122745446	0.607333221	0.216572963
Sox9	0.15952994	0.607933668	0.216143804
Casz1	-0.103567249	0.607933668	0.216143804
Cdc42ep5	-0.150556393	0.610964364	0.213984121
Crct1	0.141998173	0.610964364	0.213984121
Tmem51	0.125639463	0.610964364	0.213984121
Gck	0.159234246	0.610964364	0.213984121
Lrrc10b	0.137915392	0.610964364	0.213984121
Ugt8a	0.085831453	0.610964364	0.213984121
Ankrd1	-0.150871331	0.610964364	0.213984121
Prr36	-0.105628677	0.610964364	0.213984121
Galnt7	0.580374925	0.611545665	0.213571108
Lgi2	0.296779003	0.611545665	0.213571108
Hdhd3	0.213818143	0.611545665	0.213571108
Mast3	0.779848351	0.611545665	0.213571108
Klhdc3	-0.194565739	0.611669332	0.213483294
Eci2	-0.180854038	0.611669332	0.213483294
Slitrk3	-0.412059097	0.611926376	0.213300827
Hsf4	-0.448951205	0.612414581	0.212954477
B3gnt7	-0.499011781	0.612414581	0.212954477
Serinc3	0.640723251	0.612414581	0.212954477
Rfx8	-0.184292996	0.612414581	0.212954477
FAM19A1	0.179464403	0.612414581	0.212954477
Ubqln4	-0.092822711	0.612414581	0.212954477
Vmn2r29	0.147750043	0.612414581	0.212954477
Kcnk9	-0.127205874	0.612414581	0.212954477
Pea15a	0.125946607	0.612414581	0.212954477
Oxr1	-0.118087531	0.612414581	0.212954477
P2ry2	0.239554557	0.612414581	0.212954477
Mgst2	0.115756919	0.612414581	0.212954477
Egflam	-0.136929189	0.612414581	0.212954477
Slc7a11	0.158197055	0.612414581	0.212954477
Col14a1	-0.129215301	0.612414581	0.212954477
Dock3	0.123883614	0.612414581	0.212954477
Nkx3-1	-0.245567208	0.612564305	0.212848313
Fmr1nb	-0.130824298	0.612564305	0.212848313
Rxfp2	0.157066661	0.612564305	0.212848313
Tmem104	-0.466315327	0.612564305	0.212848313
Gna14	-0.458161187	0.612564305	0.212848313
Gjd2	0.132262733	0.612564305	0.212848313
Mypopos	0.100663941	0.612564305	0.212848313
Smox	0.225544153	0.612564305	0.212848313
Tmem14a	0.108825693	0.612564305	0.212848313
Rit2	0.120396335	0.612564305	0.212848313
Noxo1	0.12489418	0.612564305	0.212848313
Ralgapa1	-0.265680801	0.612619733	0.212809018

INADL	0.140205587	0.612619733	0.212809018
Tmbim1	0.247095951	0.612619733	0.212809018
Capza1	0.234132514	0.612619733	0.212809018
Vgf	0.183308196	0.612619733	0.212809018
Npas3	-0.206100583	0.612619733	0.212809018
Arhgap20	0.233656346	0.612619733	0.212809018
Zfp612	0.183696443	0.612619733	0.212809018
Snai2	0.172308887	0.612619733	0.212809018
Cdk16	0.149724474	0.612619733	0.212809018
Pglyrp1	0.180097585	0.612619733	0.212809018
Spa17	0.166844596	0.612619733	0.212809018
Ankrd33b	0.257892495	0.612619733	0.212809018
Gba	0.13280668	0.612619733	0.212809018
Suox	0.228472679	0.612619733	0.212809018
Adcy9	0.139906809	0.612619733	0.212809018
Myrip	0.246989602	0.613118804	0.212455364
Dusp3	-0.228378646	0.614024309	0.211814435
Magt1	0.13027146	0.614024309	0.211814435
Adora1	-0.248059108	0.614024309	0.211814435
Snrpn	0.11882242	0.614024309	0.211814435
Rhoa	0.235476172	0.614719362	0.211323107
Vmn2r87	0.204779012	0.614828493	0.211246014
Slc12a9	0.098580383	0.614828493	0.211246014
B4galnt3	-0.1273996	0.615343721	0.210882226
Pcdha3	-0.116872927	0.615343721	0.210882226
Pcsk2os2	0.149561514	0.615365102	0.210867137
Arhgef40	0.092946367	0.615878362	0.210505054
Slc12a4	0.151453214	0.616074717	0.210366614
Cnksr3	-0.394401033	0.617238631	0.209546901
FAM132B	-0.160843479	0.617238631	0.209546901
Hmgn1	0.297637046	0.617238631	0.209546901
Ptk2	0.141870289	0.617238631	0.209546901
C1ql1	0.239843157	0.617238631	0.209546901
Sfxn5	0.130796169	0.617238631	0.209546901
Slc41a3	0.168737092	0.617238631	0.209546901
Ankrd34a	0.129405377	0.617238631	0.209546901
Plcd3	-0.131487228	0.617238631	0.209546901
Fam229a	0.13221308	0.617238631	0.209546901
Mfap2	0.227352152	0.617238631	0.209546901
Cntnap1	-0.254026395	0.61733344	0.209480197
Cntn4	0.129407602	0.61733344	0.209480197
Fstl3	0.09708965	0.61733344	0.209480197
Kif21a	0.160789474	0.61733344	0.209480197
DNAIC2	0.119275995	0.617887645	0.209090489
Pcna	0.094293657	0.618370882	0.208750969
AU040972	-0.502676264	0.6190902	0.208246071
Chrm2	-0.29653239	0.6190902	0.208246071
Alg14	0.429406043	0.6190902	0.208246071

Sft2d2	-0.169709075	0.6190902	0.208246071
Wfs1	0.203499803	0.6190902	0.208246071
Marco	-0.104823994	0.6190902	0.208246071
SETDB2-PI	-0.225825533	0.6190902	0.208246071
Tshz3	-0.082631875	0.6190902	0.208246071
D17WSU9	-0.437036141	0.61945777	0.207988295
Jag1	-0.330729085	0.61945777	0.207988295
Ppm1j	-0.315949814	0.61945777	0.207988295
Neurod1	-0.215057994	0.61945777	0.207988295
Mboat1	0.136164798	0.61945777	0.207988295
Ablim1	-0.314960571	0.61945777	0.207988295
Apba2	-0.123438786	0.61945777	0.207988295
Pear1	0.19084091	0.61945777	0.207988295
Anxa11	0.110652785	0.61945777	0.207988295
Klhl5	0.166441821	0.61945777	0.207988295
Btn2a2	0.190679146	0.61945777	0.207988295
Sh3bgrl	-0.201536643	0.61945777	0.207988295
Atxn1	0.200370362	0.61945777	0.207988295
Carlr	0.141654634	0.61945777	0.207988295
Prrg3	-0.103050537	0.61945777	0.207988295
Sema4f	0.096936746	0.61945777	0.207988295
Lpgat1	0.153173274	0.61945777	0.207988295
Bmper	0.138409388	0.61945777	0.207988295
Tspan5	0.169008659	0.61945777	0.207988295
Nppc	0.140481405	0.61945777	0.207988295
Cln8	0.278931921	0.61945777	0.207988295
Hspb9	0.106885259	0.61945777	0.207988295
Arhgef6	0.336965849	0.61945777	0.207988295
Mpp2	-0.112759347	0.61945777	0.207988295
Grip2	0.164939916	0.61945777	0.207988295
Hpn	0.150622151	0.61945777	0.207988295
Snph	-0.128683256	0.61945777	0.207988295
Krt222	-0.113562226	0.61945777	0.207988295
Snx8	-0.145340879	0.61945777	0.207988295
Sncb	-0.119306559	0.61945777	0.207988295
Ndrg3	0.211659217	0.61945777	0.207988295
Ak8	0.095493477	0.61945777	0.207988295
Apool	-0.659705409	0.619629006	0.20786826
Galc	-0.415561118	0.619629006	0.20786826
Akap14	-0.490029754	0.619629006	0.20786826
C1qtnf2	-0.428602869	0.619629006	0.20786826
Idh2	-0.378533252	0.619629006	0.20786826
Mast1	-0.346196138	0.619629006	0.20786826
Idi1	-0.432756246	0.619629006	0.20786826
Capns1	-0.402342182	0.619629006	0.20786826
Nectin4	0.140006643	0.619629006	0.20786826
Atp2a1	-0.263745626	0.619629006	0.20786826
Mapre1	-0.2769541	0.619629006	0.20786826

Cd101	0.128581552	0.619629006	0.20786826
Fam169a	-0.381794372	0.619629006	0.20786826
Creg2	0.262871395	0.619629006	0.20786826
Sec14l3	-0.321563566	0.619629006	0.20786826
Acyp1	-0.151585644	0.619629006	0.20786826
Tgfb2	0.368137541	0.619629006	0.20786826
Hrk	-0.172152958	0.619629006	0.20786826
FAM188B2	-0.226573502	0.619629006	0.20786826
Smpx	0.17919973	0.619629006	0.20786826
C4a	-0.247551793	0.619629006	0.20786826
Eml5	-0.224786472	0.619629006	0.20786826
Txndc17	0.129452634	0.619629006	0.20786826
Ankrd34b	-0.161139535	0.619629006	0.20786826
Prdm6	-0.304478538	0.619629006	0.20786826
Olfm1	0.152740532	0.619629006	0.20786826
Phgdh	-0.152013956	0.619629006	0.20786826
Rplp2	-0.144334004	0.619629006	0.20786826
Gabra3	0.165240562	0.619629006	0.20786826
Il18rap	-0.114662833	0.619629006	0.20786826
Usp32	0.128092158	0.619629006	0.20786826
Prrt3	0.159660483	0.619629006	0.20786826
Polr2h	0.553875979	0.619629006	0.20786826
Lin7a	-0.221151959	0.619629006	0.20786826
Hepacam	0.161749411	0.619629006	0.20786826
Syt7	-0.192301059	0.619629006	0.20786826
Camk4	0.142385884	0.619629006	0.20786826
3-Sep	0.098705028	0.619629006	0.20786826
Spocd1	-0.182298449	0.619629006	0.20786826
Ccdc3	0.164957337	0.619629006	0.20786826
Plekho1	0.746023616	0.619629006	0.20786826
Gng12	0.200286523	0.619629006	0.20786826
Rab27a	0.168676431	0.619629006	0.20786826
Nap1l3	0.51272451	0.619629006	0.20786826
Cpxm2	0.158025915	0.619629006	0.20786826
Irx6	0.12256316	0.619629006	0.20786826
Ggt7	-0.122586665	0.619629006	0.20786826
Madd	0.250912328	0.619629006	0.20786826
Fmo1	-0.142456288	0.619629006	0.20786826
Drd5	0.213897865	0.619629006	0.20786826
Mri1	-0.141248429	0.619629006	0.20786826
Il12rb1	0.272965075	0.619629006	0.20786826
Taldo1	0.214761907	0.619629006	0.20786826
Tshr	0.21941459	0.619629006	0.20786826
Carhsp1	0.224919471	0.619629006	0.20786826
Nat1	0.087047788	0.619629006	0.20786826
Prss35	0.118639549	0.619629006	0.20786826
Plxdc1	-0.16942394	0.619629006	0.20786826
Nrip3	0.1043035	0.619629006	0.20786826

Stxbp5l	0.104957921	0.619629006	0.20786826
Sass6	0.117138082	0.619629006	0.20786826
Palm3	0.356536261	0.619629006	0.20786826
Chp2	0.213699012	0.619629006	0.20786826
Rnf180	0.278856972	0.619629006	0.20786826
Itga2	0.143433079	0.619629006	0.20786826
Tmem81	0.103212331	0.619629006	0.20786826
Adamts19	0.157085593	0.619629006	0.20786826
Pantr1	-0.340504263	0.619629006	0.20786826
Spata5l1	0.127425073	0.619629006	0.20786826
Rab11fip4	0.165596043	0.619629006	0.20786826
Strip2	0.186917592	0.619629006	0.20786826
Sntg1	-0.114896796	0.619629006	0.20786826
Fam131b	-0.126443716	0.619629006	0.20786826
Ptprk	0.159616901	0.619629006	0.20786826
Ndp	0.101120777	0.619629006	0.20786826
Erg	0.132737081	0.619629006	0.20786826
Prkaa2	0.133583264	0.619629006	0.20786826
Tubg2	-0.200392341	0.619629006	0.20786826
Lhx1	0.243591774	0.619629006	0.20786826
Cradd	-0.174166104	0.619629006	0.20786826
Htr3a	-0.136288969	0.619629006	0.20786826
Dlgap3	0.128390977	0.619629006	0.20786826
Scg5	0.297051592	0.619629006	0.20786826
Tradd	0.198372049	0.619629006	0.20786826
Pcsk9	0.326384753	0.619629006	0.20786826
Clec14a	0.160905008	0.619629006	0.20786826
BC048507	-0.298541077	0.619629006	0.20786826
Tmem240	0.200547516	0.619629006	0.20786826
Magi2	0.187978943	0.619629006	0.20786826
Retnlg	0.096974038	0.619629006	0.20786826
Ccdc90b	0.169960928	0.619629006	0.20786826
Acot10	0.16365476	0.619629006	0.20786826
FAM155A	0.256586151	0.619629006	0.20786826
H2-DMa	-0.123652768	0.619629006	0.20786826
Myo1c	0.180715964	0.619629006	0.20786826
SCN2A1	0.268968708	0.619629006	0.20786826
SSFA2	-0.112763761	0.619629006	0.20786826
Arl6ip6	-0.145216493	0.619629006	0.20786826
Spn	0.12729004	0.619629006	0.20786826
Fbxo34	-0.199828645	0.619629006	0.20786826
Ppfia2	0.115097521	0.619629006	0.20786826
Fgf13	-0.108650971	0.619629006	0.20786826
ERO1LB	-0.128527571	0.619629006	0.20786826
Pira2	0.121312463	0.619629006	0.20786826
Fbn2	-0.213979636	0.619629006	0.20786826
Fut9	-0.209397988	0.619629006	0.20786826
Tm7sf2	-0.250448	0.619629006	0.20786826

Mipol1	-0.262843756	0.619629006	0.20786826
Clvs2	-0.133712087	0.619629006	0.20786826
Drd1	-0.176189704	0.619629006	0.20786826
C1qtnf5	0.133319755	0.619629006	0.20786826
Epb41l1	0.170115732	0.619629006	0.20786826
Ddx25	0.219148206	0.619629006	0.20786826
Cdk5r2	-0.170881309	0.619629006	0.20786826
Apba1	0.377806607	0.619629006	0.20786826
Nap1l5	-0.154153642	0.619629006	0.20786826
Adcy2	0.100351707	0.619629006	0.20786826
Scrg1	0.11687038	0.619629006	0.20786826
Aqp1	-0.298947427	0.619629006	0.20786826
Tm6sf1	-0.12134418	0.619629006	0.20786826
Pola2	0.146249011	0.619629006	0.20786826
Prima1	0.132511517	0.619629006	0.20786826
Acad12	0.08289348	0.619629006	0.20786826
Fzd6	0.347056333	0.619629006	0.20786826
Prkcz	-0.171772691	0.619629006	0.20786826
Dpp10	0.295418258	0.619629006	0.20786826
St8sia1	0.233069627	0.619629006	0.20786826
Lrrc10	0.108623399	0.619629006	0.20786826
Lrp4	0.159378149	0.619629006	0.20786826
Phox2a	-0.097930024	0.619629006	0.20786826
Dlgap4	0.096628636	0.619629006	0.20786826
Fam102b	0.105341897	0.619629006	0.20786826
Orc1	-0.14518575	0.619629006	0.20786826
Prss50	0.132765242	0.619629006	0.20786826
Trappc6a	0.116715111	0.619629006	0.20786826
Itga1	0.250318607	0.619629006	0.20786826
Apoc3	0.118896074	0.619629006	0.20786826
Sdcbp	0.281434956	0.619629006	0.20786826
Vcl	-0.101759523	0.619629006	0.20786826
HIST1H3I	0.111214364	0.619629006	0.20786826
Ttc7	0.104821831	0.619629006	0.20786826
Akap6	0.116489938	0.619629006	0.20786826
Syngr1	0.099297207	0.619629006	0.20786826
Aldh1l1	0.442477717	0.619629006	0.20786826
Abhd12b	0.161611117	0.619629006	0.20786826
Rbm3os	0.163870935	0.619629006	0.20786826
Cacna2d1	0.145043233	0.619629006	0.20786826
Slc9a5	-0.210349694	0.619629006	0.20786826
Il17rb	0.350354555	0.619629006	0.20786826
Snx9	0.097952994	0.619629006	0.20786826
Zfhx2os	0.114670609	0.619629006	0.20786826
Olfm3	0.133947042	0.619629006	0.20786826
Ankrd44	0.14988148	0.619629006	0.20786826
Slc38a4	-0.0991774	0.619629006	0.20786826
Wee1	0.143401075	0.619629006	0.20786826

Zfp867	0.096867473	0.619629006	0.20786826
Epb41l3	0.153882729	0.619629006	0.20786826
Lix1	0.234772659	0.619629006	0.20786826
Galk1	0.085210276	0.61967304	0.207837398
Icos	0.111885408	0.620658077	0.207147589
Dusp4	-0.268158917	0.620933721	0.206954754
Iqsec1	-0.122677375	0.620933721	0.206954754
Ptk7	0.146355603	0.620933721	0.206954754
Rpl36	0.101814462	0.620933721	0.206954754
Pou3f4	-0.12291953	0.620933721	0.206954754
Hsph1	-0.137427229	0.620933721	0.206954754
Oaz3	0.293779341	0.620933721	0.206954754
Cyp4f17	0.145004624	0.620933721	0.206954754
Tbx1	-0.130668643	0.620933721	0.206954754
Chsy1	-0.190798899	0.620933721	0.206954754
Npepl1	0.088151029	0.620933721	0.206954754
Map1a	-0.110016591	0.620933721	0.206954754
Frzb	-0.128692369	0.620933721	0.206954754
Ost4	0.220488427	0.620933721	0.206954754
Peg3	0.43131388	0.620933721	0.206954754
Mir124a-1	0.118789846	0.620933721	0.206954754
Zfp579	0.118697718	0.620933721	0.206954754
Nrp2	-0.136670854	0.620933721	0.206954754
Cthrc1	0.086856509	0.620933721	0.206954754
Pnlip	-0.269305024	0.621947926	0.206245976
Kcnq5	0.157068882	0.621947926	0.206245976
Hivep3	-0.166028947	0.621947926	0.206245976
Il12rb2	0.187007667	0.621947926	0.206245976
Slitrk4	0.337951649	0.621947926	0.206245976
Serpini1	0.166250905	0.621947926	0.206245976
Mt3	0.157025565	0.621953324	0.206242207
Zbtb37	0.232887344	0.622376673	0.205946693
Ldoc1	0.255481581	0.623071491	0.20546212
Ttc3	0.135669467	0.624118466	0.204732968
Nhlrc3	0.246608908	0.624118466	0.204732968
Uaca	-0.094228224	0.624118466	0.204732968
Arpc5	0.1901064	0.624118466	0.204732968
Tbx3	0.132484678	0.624118466	0.204732968
Ss18	-0.194281598	0.624488332	0.204475672
Plcl2	0.101749833	0.624488332	0.204475672
Akr1c13	0.1028655	0.624488332	0.204475672
FAM60A	0.186587534	0.624488332	0.204475672
Gjd3	0.207101496	0.624488332	0.204475672
Hba-a1	0.120675017	0.624488332	0.204475672
Plcd4	-0.119324009	0.624488332	0.204475672
Tmem156	0.11617466	0.625072239	0.204069789
Odf3b	0.132108933	0.625963766	0.203450806
Stau2	0.381679423	0.625963766	0.203450806

Gls	0.212932695	0.626239666	0.203259428
Brinp2	0.216448557	0.626948097	0.202768412
Cep170b	-0.201277101	0.6285605	0.201652914
Mustn1	-0.144904304	0.6285605	0.201652914
Gria3	0.119553737	0.6285605	0.201652914
Nr1d1	0.114514204	0.6285605	0.201652914
Btg2	0.134461628	0.6285605	0.201652914
Spock1	0.183035314	0.6285605	0.201652914
Slc43a1	0.416910659	0.628607341	0.201620551
Alox12	0.136370237	0.628607341	0.201620551
Rpl10a	-0.418796777	0.628656366	0.201586682
Hacd2	-0.138535152	0.628656366	0.201586682
Prkca	-0.182970694	0.628656366	0.201586682
Exoc3l4	-0.23700104	0.628656366	0.201586682
Ppp1r37	-0.224893562	0.628656366	0.201586682
BC055324	0.181777696	0.628656366	0.201586682
Mta3	-0.14820324	0.628656366	0.201586682
Rax	0.165471428	0.628656366	0.201586682
Sh3rf3	0.136971656	0.628656366	0.201586682
Degs2	-0.094788474	0.628656366	0.201586682
Flt3	0.126831392	0.628656366	0.201586682
Lrfn1	-0.103698641	0.628656366	0.201586682
Uox	-0.134712392	0.628656366	0.201586682
Pon1	0.124348501	0.628656366	0.201586682
Sptssa	-0.25182983	0.628656366	0.201586682
Pdia5	0.361685296	0.628656366	0.201586682
Spred2	-0.103699829	0.628656366	0.201586682
CHD3OS	0.260433879	0.628656366	0.201586682
Rspo2	-0.237832748	0.628656366	0.201586682
Nkiras1	-0.087243351	0.628656366	0.201586682
Hrct1	0.119804956	0.628656366	0.201586682
Top3a	0.087512988	0.628656366	0.201586682
Nos2	0.085632181	0.628656366	0.201586682
Twf1	-0.13384087	0.628656366	0.201586682
Crym	0.099306959	0.628656366	0.201586682
Lrp8os3	-0.134794011	0.628656366	0.201586682
Shkbp1	0.182654223	0.628656366	0.201586682
Exd2	-0.13189482	0.628656366	0.201586682
Adamts7	0.352958702	0.628656366	0.201586682
Ccdc149	-0.259510781	0.628656366	0.201586682
Tpm2	-0.087962272	0.628656366	0.201586682
TMEM27	0.112384916	0.628656366	0.201586682
Naalad2	0.131321886	0.628656366	0.201586682
Wdr81	-0.120647546	0.628656366	0.201586682
Rhoq	-0.119159977	0.628656366	0.201586682
Pcdhb12	0.104043533	0.628656366	0.201586682
Slc16a7	0.10832375	0.628656366	0.201586682
Ldlrad3	0.148631519	0.628656366	0.201586682

Slc16a9	0.127093782	0.628656366	0.201586682
Rock2	0.222802674	0.628656366	0.201586682
Ppp1r1b	0.185928615	0.628656366	0.201586682
Hbb-bs	0.33989645	0.628656366	0.201586682
Nlrp1a	0.150879044	0.628656366	0.201586682
Stt3a	0.124061445	0.628656366	0.201586682
Camkk1	-0.157345638	0.628656366	0.201586682
Nr2e1	0.126080683	0.628656366	0.201586682
Itgbl1	0.070310234	0.628656366	0.201586682
Atg9a	0.105622524	0.628656366	0.201586682
Moxd1	0.333674351	0.628656366	0.201586682
Chrd	0.100174078	0.628656366	0.201586682
Rcan2	0.104456169	0.628656366	0.201586682
Ctsw	0.115459177	0.628717037	0.201544771
Col4a2	0.350170039	0.629125378	0.201262796
Lamc2	0.153545071	0.629125378	0.201262796
Arhgap18	0.525484433	0.629125378	0.201262796
Pcdhac2	-0.128923883	0.629125378	0.201262796
Klhl25	0.174697693	0.629125378	0.201262796
Slc25a13	0.113535651	0.630113757	0.200581039
Rbm43	0.260301002	0.631391981	0.199700938
Adgb	-0.306440398	0.631722138	0.199473903
Pawr	0.419273571	0.631722138	0.199473903
Kremen2	0.15013704	0.631722138	0.199473903
LNP	0.133810146	0.631722138	0.199473903
Unc79	0.290788476	0.631722138	0.199473903
Tnip2	-0.177296598	0.631722138	0.199473903
Tmem45a	-0.123814235	0.631722138	0.199473903
Ralyl	0.09028277	0.631722138	0.199473903
Tjp2	-0.251786578	0.632472427	0.198958403
Sh3gl2	0.122532579	0.632472427	0.198958403
Ormdl1	-0.101007823	0.633352578	0.198354458
Dnm1	0.114391271	0.633988539	0.197918593
Plch2	0.12710117	0.634009461	0.197904262
Prkg2	0.152055408	0.634690069	0.197438297
Fam131a	-0.095105047	0.634935002	0.197270731
Fuca2	-0.174476775	0.634935002	0.197270731
Prorsd1	0.163593245	0.634935002	0.197270731
Adam18	0.189084607	0.634935002	0.197270731
Plpp3	-0.489008787	0.634969234	0.197247317
Mreg	-0.455651915	0.634969234	0.197247317
Gsto2	-0.520265976	0.634969234	0.197247317
Zfp229	-0.258290519	0.634969234	0.197247317
Gsg1	0.211432045	0.634969234	0.197247317
Ly6e	-0.163729313	0.634969234	0.197247317
Dnajc27	-0.253729748	0.634969234	0.197247317
Mc5r	0.208627039	0.634969234	0.197247317
Cbx7	0.243020615	0.634969234	0.197247317

Kcnrg	0.154510468	0.634969234	0.197247317
Wnt4	-0.16954567	0.634969234	0.197247317
Cyp11a1	0.144678282	0.634969234	0.197247317
Tunar	0.310822164	0.634969234	0.197247317
Barx2	-0.15373744	0.634969234	0.197247317
Pdk4	-0.112663296	0.634969234	0.197247317
H2-T24	0.384278017	0.634969234	0.197247317
Uchl5	-0.127639902	0.634969234	0.197247317
Sec11a	0.29875137	0.634969234	0.197247317
Tnfrsf11b	0.173588354	0.634969234	0.197247317
Gjb5	0.119686552	0.634969234	0.197247317
Pla2g7	0.188725931	0.634969234	0.197247317
Plat	0.293041676	0.634969234	0.197247317
Sulf1	0.114370094	0.634969234	0.197247317
Notch1	0.128682223	0.634969234	0.197247317
Gpr25	0.153846739	0.634969234	0.197247317
Nectin1	0.099203702	0.634969234	0.197247317
Abtb2	-0.143373327	0.634969234	0.197247317
Tanc2	0.161833892	0.634969234	0.197247317
TMEM8C	0.103439015	0.634969234	0.197247317
Tpst2	-0.16236642	0.634969234	0.197247317
Ryk	0.148038822	0.634969234	0.197247317
Mpl	0.125301893	0.634969234	0.197247317
Fam189a1	-0.197897849	0.634969234	0.197247317
H3f3a	-0.391583099	0.635343486	0.196991418
Marf1	0.149526265	0.635343486	0.196991418
Kcnk13	0.225812321	0.635343486	0.196991418
CTGF	-0.077426939	0.635343486	0.196991418
Atox1	0.345248846	0.636816158	0.195985926
Pmel	-0.292956259	0.636816158	0.195985926
Kcnj5	-0.119960824	0.636816158	0.195985926
Paqr6	0.162855364	0.636816158	0.195985926
Aoc3	0.14182979	0.637256956	0.195685415
Chrnb2	-0.120922005	0.637734953	0.19535978
Gdf11	-0.190246303	0.63787742	0.195262771
Acot5	-0.160457566	0.63787742	0.195262771
Gpr50	-0.120568658	0.63787742	0.195262771
Wnt5a	0.088641641	0.63787742	0.195262771
Mms22l	0.174003819	0.63787742	0.195262771
Tspan12	-0.113459103	0.63787742	0.195262771
Zfp369	-0.131715825	0.63787742	0.195262771
Rgs7	-0.140550845	0.638324512	0.194958478
Acss2	0.153960247	0.638324512	0.194958478
Pnoc	0.339607673	0.638324512	0.194958478
Cyp20a1	-0.152702833	0.638324512	0.194958478
Lrrc4c	-0.207804326	0.638324512	0.194958478
Adgra1	0.128081279	0.638324512	0.194958478
Spag16	0.129931778	0.638492693	0.194844068

Dzank1	0.149153777	0.638492693	0.194844068
Marcksl1	-0.093454634	0.638613919	0.19476162
Chdh	-0.283765839	0.638913918	0.194557651
Pter	0.093781074	0.638913918	0.194557651
Doc2a	-0.748242559	0.639079208	0.194445311
Ccdc92b	-0.303848272	0.639079208	0.194445311
Kcnq3	-0.338279039	0.639079208	0.194445311
Sema4d	-0.209195969	0.639079208	0.194445311
Camta1	-0.169558216	0.639079208	0.194445311
Pla2g3	-0.194358727	0.639079208	0.194445311
Dlk2	-0.105279353	0.639079208	0.194445311
Arhgap15	0.154563496	0.639079208	0.194445311
Rimbp2	0.163454897	0.639079208	0.194445311
Rps6kc1	0.240623642	0.639079208	0.194445311
Ccl21b	0.132314553	0.639079208	0.194445311
Atp7b	0.08539696	0.639079208	0.194445311
Adam21	0.079867312	0.639079208	0.194445311
Cacng7	0.140365099	0.639079208	0.194445311
Elovl7	0.095863238	0.639079208	0.194445311
Tmem200	0.105229728	0.639079208	0.194445311
Slc22a6	0.072111062	0.639079208	0.194445311
Fbxl2	0.108024975	0.639079208	0.194445311
Cd164l2	-0.13595291	0.639079208	0.194445311
Tppp	-0.082513229	0.639079208	0.194445311
Dct	0.140541778	0.639079208	0.194445311
Rab6a	0.096351108	0.639079208	0.194445311
Mapk3	-0.092263838	0.639079208	0.194445311
Zfp763	0.325633954	0.639300993	0.194294621
Wdfy2	0.161078856	0.639300993	0.194294621
Morc2b	0.112901489	0.639300993	0.194294621
Klhl32	0.245154335	0.639357994	0.194255901
Glrb	-0.108880879	0.639357994	0.194255901
Flrt2	0.1098654	0.639357994	0.194255901
Reps2	0.111847185	0.639357994	0.194255901
Ccdc177	-0.078982769	0.639357994	0.194255901
Ttll7	-0.088003834	0.639357994	0.194255901
Fndc4	-0.094754322	0.639357994	0.194255901
Mpst	-0.099412498	0.639943007	0.193858703
Ormdl2	-0.76085588	0.640393394	0.193553156
Adgrf4	0.309097291	0.640393394	0.193553156
Colgalt1	0.160523107	0.640393394	0.193553156
Sez6l	-0.247790703	0.640592169	0.193418375
FAM150B	0.10600946	0.640592169	0.193418375
Pcdh20	0.133827893	0.640592169	0.193418375
Rcvrn	0.118749573	0.640592169	0.193418375
Galt	0.142202293	0.640592169	0.193418375
Micu2	0.090512173	0.640592169	0.193418375
Xcl1	0.126118918	0.640592169	0.193418375

Ssr4	-0.155531039	0.640592169	0.193418375
Ska3	-0.157058236	0.640592169	0.193418375
Tub	-0.152378105	0.640592169	0.193418375
Tle2	0.100832432	0.640592169	0.193418375
Gal3st1	-0.202606587	0.641150828	0.193039792
Psg16	-0.138916019	0.641150828	0.193039792
Myo18b	-0.22470158	0.641150828	0.193039792
Cdc42se1	-0.256835639	0.641150828	0.193039792
Ptgir	0.115156664	0.641150828	0.193039792
Fads2	-0.373347564	0.641654582	0.1926987
Dync2h1	-0.150909175	0.641654582	0.1926987
Ggact	-0.117503909	0.641654582	0.1926987
Tmem71	-0.102134262	0.641654582	0.1926987
Abca6	-0.124057026	0.641654582	0.1926987
Ccdc18	-0.143649204	0.641654582	0.1926987
Rnf112	-0.197788042	0.641654582	0.1926987
Mmp23	-0.116369671	0.641654582	0.1926987
Pkd2l1	0.165336289	0.641654582	0.1926987
Smim13	0.156536701	0.641654582	0.1926987
Usp33	0.127088624	0.641654582	0.1926987
Ttc22	-0.10207456	0.641862616	0.192557918
Hbq1b	0.085632096	0.641862616	0.192557918
Stbd1	0.119974844	0.641862616	0.192557918
Sec61a1	0.191096687	0.641862616	0.192557918
Kcna3	0.125170251	0.641862616	0.192557918
Jph3	-0.124779159	0.641862616	0.192557918
Fbxo30	0.104410283	0.641862616	0.192557918
Ptgds	0.224582966	0.641990497	0.1924714
Ksr2	0.183401027	0.641990497	0.1924714
Ccdc115	-0.103361497	0.641990497	0.1924714
Vsig10	0.169702004	0.643037135	0.191763946
Atp13a5	-0.373133851	0.643037135	0.191763946
Tom1l2	0.163178735	0.643037135	0.191763946
Grin1	-0.122603361	0.644583349	0.190720918
Ank3	0.097058159	0.644583349	0.190720918
Rack1	-0.796326802	0.645712623	0.189960724
Ctdsp1	0.147216087	0.645712623	0.189960724
Rps18	0.322212159	0.645712623	0.189960724
Hba-a2	0.095189733	0.645712623	0.189960724
Lepr	0.24799068	0.645712623	0.189960724
Csf2ra	0.17569821	0.645712623	0.189960724
Osgepl1	0.178450056	0.645712623	0.189960724
Lax1	0.099208231	0.645712623	0.189960724
Magel2	-0.195727469	0.645712623	0.189960724
Mkrn2os	0.142351343	0.645712623	0.189960724
Lmf2	0.093934841	0.645712623	0.189960724
Kank1	0.276995782	0.645712623	0.189960724
Scarb2	-0.087164249	0.645712623	0.189960724

Wnt2b	0.094145776	0.645712623	0.189960724
Mark1	-0.125342604	0.645712623	0.189960724
Zmiz2	0.112222068	0.645712623	0.189960724
Gtdc1	-0.25227011	0.647375371	0.188843827
Ccdc160	0.115242605	0.647375371	0.188843827
Thrsp	0.110248007	0.650278247	0.186900774
Ehbp1l1	-0.343641262	0.650628529	0.186666898
Olfr690	-0.207011918	0.651117102	0.186340898
Cacnb3	-0.077455022	0.651296517	0.186221244
Klhl35	-0.152855542	0.652011376	0.185744827
Zbtb7b	0.113187352	0.652011376	0.185744827
Ccs	0.103328539	0.652011376	0.185744827
Iqcg	0.128625328	0.652011376	0.185744827
Fv1	0.078970108	0.652011376	0.185744827
Tmem134	0.08712669	0.652011376	0.185744827
Trim9	0.190803624	0.652011376	0.185744827
Sppl2a	0.097437591	0.652011376	0.185744827
Ly6i	-0.399083086	0.652986318	0.185095918
Srpk1	0.148109723	0.652986318	0.185095918
Rhou	0.137617926	0.652986318	0.185095918
Dennd2c	0.122945871	0.652986318	0.185095918
Atoh7	-0.162314243	0.652986318	0.185095918
Sirpb1a	0.181231381	0.652986318	0.185095918
Fam181b	-0.195855112	0.653537337	0.184729596
Pycr1	0.110285018	0.653537337	0.184729596
Asb13	-0.150241818	0.653537337	0.184729596
Ppp2r2c	0.122531552	0.654183497	0.184300416
Sfrp4	-0.167716595	0.655087771	0.183700508
Clu	0.199052289	0.655146616	0.183661498
Ankrd9	-0.146762452	0.655193833	0.183630199
Arhgef25	-0.239391135	0.65546797	0.183448526
Susd2	0.098245281	0.65546797	0.183448526
Tyk2	0.252613703	0.655577214	0.18337615
Myl6b	0.161242228	0.655577214	0.18337615
CCDC64	-0.109797557	0.655577214	0.18337615
Dok6	-0.206079364	0.655702063	0.18329345
Foxs1	0.077970257	0.655702063	0.18329345
Arhgap27	0.333363705	0.655702063	0.18329345
Pip4k2c	0.111860885	0.656418951	0.182818889
Nop9	-0.105286927	0.656788068	0.182574746
Sptan1	-0.202497294	0.656986849	0.182443323
Atad2	0.324606816	0.656986849	0.182443323
Lrrc3b	0.272648737	0.656986849	0.182443323
Mrps28	0.178239996	0.656986849	0.182443323
Osbpl10	-0.118788615	0.656986849	0.182443323
Trpc4	-0.168240564	0.656986849	0.182443323
Cnksr2	0.31830763	0.656986849	0.182443323
Idua	0.33562525	0.656986849	0.182443323

Pola1	0.114293017	0.656986849	0.182443323
SHFM1	0.130794533	0.656986849	0.182443323
Rbms1	0.315482125	0.656986849	0.182443323
Urah	0.13851975	0.656986849	0.182443323
P2ry1	0.163542376	0.656986849	0.182443323
Rnf183	-0.186636794	0.656986849	0.182443323
Cr1l	0.085338523	0.656986849	0.182443323
Plxna2	0.199322678	0.656986849	0.182443323
Ripply3	-0.193636019	0.656986849	0.182443323
C77080	0.248912245	0.657205801	0.182298612
Ccl19	0.147599682	0.657205801	0.182298612
Ssr3	0.139755054	0.657205801	0.182298612
Habp4	0.208200502	0.657205801	0.182298612
Kcna5	0.255536409	0.657205801	0.182298612
Gjc1	0.243198524	0.657955603	0.18180341
Btc	-0.518658744	0.658465307	0.181467102
Krt26	0.141543813	0.658465307	0.181467102
Lrig3	0.168477328	0.658507336	0.181439382
Sh3bgrl3	0.121405021	0.659141789	0.181021154
Fam186b	0.119894476	0.659141789	0.181021154
Prdm11	0.068364506	0.659141789	0.181021154
Uimc1	0.078860458	0.659161141	0.181008403
Fbxo31	-0.319085578	0.659435059	0.180827967
Nfix	0.214828272	0.659590157	0.180725834
Dancr	0.160919366	0.659601588	0.180718308
Gria4	0.302779875	0.659601588	0.180718308
Lhx9	0.131571616	0.659601588	0.180718308
Hdac4	0.0978018	0.659601588	0.180718308
Pcnx2	0.141318543	0.659601588	0.180718308
Bmpr1a	0.160770053	0.659648213	0.18068761
FAM65C	-0.43253343	0.659842908	0.180559447
Flywch1	-0.254333114	0.659842908	0.180559447
Plxnb1	0.121957401	0.659842908	0.180559447
Elovl4	-0.11221366	0.659842908	0.180559447
Slc45a1	0.198680898	0.659842908	0.180559447
Adrb3	0.079332401	0.659842908	0.180559447
Golim4	0.253455977	0.659842908	0.180559447
Wdfy1	-0.162511416	0.659842908	0.180559447
Tgfa	0.098889818	0.659842908	0.180559447
Gabra4	0.175098416	0.659842908	0.180559447
Pnkd	0.117741296	0.659842908	0.180559447
Nmnat2	0.103397635	0.66000671	0.180451649
Coa4	0.152215939	0.660211801	0.180316717
Slc4a10	-0.287253553	0.660220216	0.180311182
Ccdc85a	0.140508393	0.660220216	0.180311182
Irf2bp2	0.091196813	0.660220216	0.180311182
Xkr5	-0.53123922	0.66087219	0.179882523
Ptprn2	0.258918302	0.66087219	0.179882523

Inpp4a	0.289515578	0.66087219	0.179882523
Klf12	0.338542304	0.66087219	0.179882523
Rapgefl1	-0.17683453	0.66087219	0.179882523
Tnk2	0.107612214	0.66087219	0.179882523
Zfp516	-0.149127698	0.66087219	0.179882523
Cfap52	0.194430932	0.66087219	0.179882523
Vat1	0.09590588	0.66087219	0.179882523
Pex5l	0.347260546	0.66087219	0.179882523
R74862	-0.083144973	0.661541843	0.179442681
Chsy3	0.126073469	0.661541843	0.179442681
Gnmt	0.237102647	0.661541843	0.179442681
Ly6g6e	-0.089606368	0.661541843	0.179442681
Hells	0.212543822	0.661790017	0.179279789
Ccdc8	-0.168482415	0.661847448	0.179242102
Fbxo41	-0.316968709	0.662929184	0.178532862
Cyb561a3	-0.178770916	0.662929184	0.178532862
Sgsm3	-0.096480262	0.662929184	0.178532862
Fam126b	0.128665293	0.662929184	0.178532862
Zfp114	0.121810007	0.662929184	0.178532862
Gtf2a1l	-0.134935741	0.662929184	0.178532862
Sema3b	0.166912666	0.662929184	0.178532862
Rnf149	0.106632679	0.662929184	0.178532862
G0s2	0.244842407	0.662929184	0.178532862
Mag	0.107877922	0.662929184	0.178532862
Tmem256	0.236374801	0.662929184	0.178532862
Pold1	0.238822325	0.662929184	0.178532862
Ptcd2	0.145709839	0.662929184	0.178532862
Limd2	0.170230528	0.662992551	0.178491351
FAM96A	-0.123316079	0.663012113	0.178478537
Usp31	0.279943326	0.663221199	0.178341601
Map3k1	0.085644916	0.663221199	0.178341601
Nrgn	0.113177387	0.663282212	0.17830165
Arrb2	-0.108798105	0.66329486	0.178293368
Ppp1r14c	0.211159905	0.66329486	0.178293368
Wdr47	-0.101371749	0.66329486	0.178293368
Kcnn2	-0.103533038	0.66329486	0.178293368
Vsig2	0.298457254	0.66329486	0.178293368
BC017643	0.123332469	0.66329486	0.178293368
Shank3	-0.151827223	0.663619294	0.178080996
Bik	-0.135254832	0.663619294	0.178080996
Lrrc14b	0.117887029	0.663619294	0.178080996
Hmgn2	0.130856729	0.663619294	0.178080996
Sort1	0.139700332	0.663872053	0.177915613
Ceacam1(	-0.331696329	0.663872053	0.177915613
Rab25	-0.209677835	0.666316118	0.176319681
Lrch4	0.195064313	0.666316118	0.176319681
Acadm	0.138367619	0.666316118	0.176319681
Map2k3	0.139644636	0.666316118	0.176319681

Dlgap2	-0.162533106	0.666316118	0.176319681
Ahr	0.102854939	0.666479377	0.176213285
Cenpp	-0.105975415	0.666479377	0.176213285
Jazf1	0.311547757	0.668559372	0.174860019
Ttl	-0.171175691	0.668573576	0.174850792
Rps26	-0.097531641	0.668573576	0.174850792
Plekhg1	-0.700181496	0.668829468	0.1746846
Ezr	-0.394515438	0.668829468	0.1746846
Zcchc24	-0.317254787	0.668829468	0.1746846
Efs	-0.202126684	0.668829468	0.1746846
Pgr15l	-0.140612448	0.668829468	0.1746846
Gramd1b	0.142264995	0.668829468	0.1746846
Arap2	0.368873968	0.668829468	0.1746846
Pi4ka	0.169505716	0.668829468	0.1746846
Pak2	0.216991463	0.668829468	0.1746846
Mfsd6	-0.202195892	0.668829468	0.1746846
Snx16	0.094039911	0.668829468	0.1746846
Enpp4	0.113599453	0.668829468	0.1746846
Tmem47	0.124403573	0.668829468	0.1746846
Fcho1	-0.074098008	0.668829468	0.1746846
Megf11	0.135675661	0.668829468	0.1746846
WBSCR17	0.119957336	0.668829468	0.1746846
Slc24a3	-0.07002741	0.668829468	0.1746846
Acot2	-0.157002446	0.668829468	0.1746846
Stac3	-0.083327792	0.668829468	0.1746846
Hmgcr	0.163928417	0.668829468	0.1746846
Rasgrf1	0.242505349	0.668829468	0.1746846
Rarres2	0.262288904	0.668829468	0.1746846
Btrc	0.151672557	0.668829468	0.1746846
Foxc2	0.097511769	0.669820406	0.174041626
Bcl11a	0.134254509	0.670502026	0.173599905
Map9	-0.11911245	0.6710158	0.173267254
Trhr2	-0.130676533	0.672090118	0.17257249
Slc6a7	0.117339505	0.672090118	0.17257249
Rln3	-0.45916792	0.672862668	0.172073567
Thnsl2	-0.601846215	0.672862668	0.172073567
Slc15a2	-0.338915973	0.672862668	0.172073567
Sms	0.17385517	0.672862668	0.172073567
Etaa1os	0.345734574	0.672862668	0.172073567
Pkib	-0.266745962	0.672862668	0.172073567
Gadd45b	-0.101074091	0.672862668	0.172073567
Nr4a3	0.18675758	0.672862668	0.172073567
Mbd5	-0.105503823	0.672862668	0.172073567
Duxbl1	0.128581247	0.672862668	0.172073567
Nucb1	0.097829845	0.672862668	0.172073567
Dbndd2	0.066768612	0.672862668	0.172073567
Ldlr	-0.115946716	0.672862668	0.172073567
Avp	-0.444203962	0.672862668	0.172073567

Camk2n2	-0.222943867	0.672862668	0.172073567
Hrc	0.117309569	0.672862668	0.172073567
Pcdh9	-0.110133999	0.672862668	0.172073567
Dmrt2	0.095747093	0.672862668	0.172073567
Slc46a1	-0.344192609	0.672862668	0.172073567
Trim54	0.124379384	0.672862668	0.172073567
Slc38a1	0.103744843	0.672862668	0.172073567
Vps13a	-0.123638279	0.672862668	0.172073567
Aph1a	0.106071833	0.672862668	0.172073567
Rnasel	0.100052695	0.672862668	0.172073567
Tacstd2	-0.10314686	0.672862668	0.172073567
Zfp648	0.071102864	0.672862668	0.172073567
H3f3b	0.171709237	0.672862668	0.172073567
Oprd1	-0.122578616	0.672862668	0.172073567
Slamf6	-0.139861541	0.672862668	0.172073567
Kcp	0.128547695	0.672862668	0.172073567
Begain	-0.203269862	0.672862668	0.172073567
Laptm4a	0.170493715	0.672862668	0.172073567
11-Mar	0.125860275	0.672862668	0.172073567
Rasgef1b	0.240303242	0.672928961	0.172030781
Ppfibp1	0.118609351	0.672928961	0.172030781
Slc29a2	0.190099877	0.673165717	0.17187801
Trabd2b	0.137587871	0.673189039	0.171862964
Cdh24	-0.503693652	0.673254489	0.171820742
Dach1	0.09631054	0.673254489	0.171820742
Rps2	-0.139435404	0.673254489	0.171820742
Mphosph6	0.257102685	0.673254489	0.171820742
Mgat3	-0.070788311	0.673254489	0.171820742
Car8	0.103862184	0.673254489	0.171820742
Mypop	0.269703254	0.673254489	0.171820742
Ica1	-0.164276441	0.673254489	0.171820742
Lrrc36	0.160567378	0.67403975	0.171314491
Gpr37l1	-0.186783484	0.674305127	0.171143538
Atp2b1	0.271403148	0.674750901	0.170856527
Wipf2	0.323132241	0.674886198	0.170769454
Sertad1	-0.260968467	0.674886198	0.170769454
Ube2t	-0.130007474	0.674886198	0.170769454
Vti1a	0.096987686	0.674886198	0.170769454
Got1	0.231650175	0.674886198	0.170769454
Trim59	0.106453387	0.674886198	0.170769454
Anxa8	-0.123417938	0.674886198	0.170769454
Foxl2	0.116058446	0.674886198	0.170769454
Ttbk2	-0.364502033	0.674886198	0.170769454
Cdk5rap3	0.091129583	0.674886198	0.170769454
Cfi	0.21781566	0.674886198	0.170769454
Herc1	0.112358185	0.674886198	0.170769454
Ttc34	-0.140036872	0.674886198	0.170769454
Phkg1	0.180664684	0.674886198	0.170769454

Pim2	0.077817184	0.674886198	0.170769454
Cxcl2	0.161077589	0.674886198	0.170769454
Hyal2	0.082758244	0.674886198	0.170769454
Fanca	0.11735225	0.674886198	0.170769454
Plk5	-0.335629715	0.675243273	0.170539734
Dclre1c	0.215898139	0.675243273	0.170539734
Sox21	0.259803531	0.675243273	0.170539734
Ehd1	-0.124278589	0.675243273	0.170539734
Arhgap40	0.110640377	0.675243273	0.170539734
Vav3	-0.162352692	0.675243273	0.170539734
Mfsd4a	-0.135550123	0.675243273	0.170539734
Lmo4	0.15932439	0.675243273	0.170539734
Agtpbp1	0.192726169	0.675243273	0.170539734
Nell2	0.124301252	0.675243273	0.170539734
Mturn	0.155010063	0.675243273	0.170539734
Gng11	-0.128855985	0.675243273	0.170539734
Bnip2	-0.09469343	0.675243273	0.170539734
Nkrf	0.097835388	0.675243273	0.170539734
Sox5	0.286578544	0.67538288	0.170449952
Prepl	0.188616298	0.67538288	0.170449952
Zrsr1	0.28419798	0.67538288	0.170449952
Megf9	0.31818708	0.675531373	0.170354477
Ddx21	0.076559676	0.676141386	0.169962481
Cplx2	-0.281444771	0.676141386	0.169962481
Mcm7	-0.166202169	0.676141386	0.169962481
Rian	0.112557646	0.676141386	0.169962481
Notch3	0.103219194	0.676141386	0.169962481
Add2	0.118104173	0.676141386	0.169962481
Mycbp2	0.159777375	0.676198784	0.169925614
P4htm	0.276139134	0.676198784	0.169925614
Ccsap	0.091892552	0.676198784	0.169925614
Msmo1	-0.124976514	0.676198784	0.169925614
Rpl32	0.100692796	0.676198784	0.169925614
Exo1	0.103496927	0.676198784	0.169925614
Cds2	0.157749479	0.676774108	0.169556265
Lmbrd2	0.22197689	0.676774108	0.169556265
Cyb561	0.151468614	0.676774108	0.169556265
Foxn2	0.172736573	0.677116104	0.169336857
Nr4a1	0.121141827	0.678763296	0.16828165
Rab34	0.119544164	0.678763296	0.16828165
Ttc7b	0.130360482	0.678763296	0.16828165
TMEM254A	0.111996577	0.680113912	0.167418341
Rela	0.1151663	0.680113912	0.167418341
Kcnq2	-0.253847068	0.680119968	0.167414474
Rnf220	0.252246313	0.680648711	0.167076973
Sptb	-0.112597285	0.680648711	0.167076973
Fam126a	0.166601647	0.681129075	0.166770581
Fkbp4	0.117075138	0.68157625	0.166485551

Ppp1r3f	-0.163771287	0.68157625	0.166485551
Zbtb18	0.125110261	0.68157625	0.166485551
Gsk3a	-0.083735663	0.68157625	0.166485551
Slc5a6	-0.259175121	0.68157625	0.166485551
Pcdha10	0.187769638	0.681648492	0.166439522
Celsr1	0.159771003	0.682171219	0.166106608
Actrt3	-0.103198223	0.682177829	0.1661024
Rnase12	0.169881014	0.683142969	0.165488397
Mapk9	-0.381499162	0.684088678	0.164887598
PRKCDBP	0.08474758	0.68421312	0.164808602
Pcdha2	0.450910374	0.68421312	0.164808602
Rgs19	0.513139685	0.68421312	0.164808602
Syn1	0.186297405	0.68421312	0.164808602
Zfp112	0.199038229	0.684507379	0.164621866
Ash1l	0.110122009	0.685418486	0.164044187
Hdac9	0.15311623	0.685442498	0.164028972
Fsd1	0.183680271	0.685442498	0.164028972
Dlc1	-0.201181989	0.686556129	0.163323952
Slc35d1	0.347694971	0.686556129	0.163323952
Prrt2	-0.083209608	0.686556129	0.163323952
Uhmk1	-0.119521818	0.686556129	0.163323952
Ubxn2a	-0.118154532	0.686556129	0.163323952
Calm3	-0.139044344	0.686784849	0.163179294
Pde4d	0.27141122	0.687175904	0.162932078
Nova1	-0.283253997	0.687175904	0.162932078
Gramd2	0.095035805	0.687175904	0.162932078
Fkbp14	-0.080777996	0.687175904	0.162932078
Slc13a5	0.071082054	0.687175904	0.162932078
Slc44a2	-0.29317613	0.687253141	0.162883267
Ptgdr	-0.071482169	0.687253141	0.162883267
Gjc3	0.132128051	0.68781208	0.162530201
Smad5	0.088285376	0.68781208	0.162530201
Ccdc65	-0.523584301	0.688233162	0.162264405
Ttc9b	0.292364316	0.688233162	0.162264405
Slc35d3	-0.277012824	0.688233162	0.162264405
Vsig10l	0.179406662	0.688233162	0.162264405
Plin4	0.139861106	0.688233162	0.162264405
Slc17a7	-0.221976279	0.688233162	0.162264405
Hps5	0.261762946	0.688233162	0.162264405
Frmpd4	0.188976052	0.688233162	0.162264405
Triobp	-0.39854261	0.688233162	0.162264405
Adam11	0.122350947	0.688233162	0.162264405
Mob3b	0.192331828	0.688233162	0.162264405
Ssx9	0.622732801	0.688233162	0.162264405
Lpcat4	0.167965667	0.688233162	0.162264405
Sstr2	-0.13250477	0.688233162	0.162264405
Ankrd45	-0.249699973	0.688233162	0.162264405
Acap3	0.097894448	0.688233162	0.162264405

Synpo	0.127044175	0.688233162	0.162264405
Uprt	0.191272452	0.688233162	0.162264405
Cox6a2	0.175766806	0.688233162	0.162264405
Tmem165	0.095328365	0.688233162	0.162264405
Enpp3	0.118756093	0.688233162	0.162264405
Dlx4os	0.21691491	0.688233162	0.162264405
Adm	0.240462474	0.688233162	0.162264405
Krt9	0.126782182	0.688233162	0.162264405
Cnih3	0.0961501	0.688233162	0.162264405
Cyp2j12	0.106322484	0.688233162	0.162264405
Alyref2	0.177014995	0.688233162	0.162264405
Efhc1	0.077351558	0.688233162	0.162264405
ADRBK1	0.364358162	0.688233162	0.162264405
Stip1	-0.110042512	0.688233162	0.162264405
Wnt7b	-0.09787312	0.688233162	0.162264405
Ddah1	-0.111227439	0.688233162	0.162264405
FAM212B	-0.120134864	0.688233162	0.162264405
Pts	0.113157467	0.688233162	0.162264405
AU041133	0.215239428	0.688233162	0.162264405
Hic1	0.101399437	0.688233162	0.162264405
Tgif2	0.357387698	0.688233162	0.162264405
Map6d1	-0.295367242	0.688233162	0.162264405
Smyd1	0.131456868	0.688233162	0.162264405
Galnt1	0.100349329	0.688233162	0.162264405
Sema5a	-0.082982829	0.688233162	0.162264405
Pip4k2b	0.12072582	0.688233162	0.162264405
Cdh18	-0.087752699	0.688233162	0.162264405
Elane	0.131046258	0.688233162	0.162264405
Npas2	0.140274659	0.688233162	0.162264405
Dleu7	0.118135992	0.688233162	0.162264405
Bri3bp	0.159260167	0.688233162	0.162264405
Ptpn1	0.093289739	0.688233162	0.162264405
Ube2e2	0.116212629	0.688233162	0.162264405
Wbp1l	0.087658035	0.688233162	0.162264405
Mocos	-0.094498008	0.688233162	0.162264405
BB557941	0.133007912	0.688233162	0.162264405
Tmem178	-0.086090208	0.688233162	0.162264405
Ar	0.091656791	0.688233162	0.162264405
Tsacc	0.174997376	0.688233162	0.162264405
Mtap	0.10866721	0.688233162	0.162264405
Lgals7	0.087715584	0.688233162	0.162264405
Fgf12	0.092319624	0.688233162	0.162264405
Adamts15	0.181798831	0.688233162	0.162264405
Mdh1	-0.121129178	0.688233162	0.162264405
Dnajb13	0.281024447	0.688233162	0.162264405
Haus8	0.243794574	0.688496941	0.162097985
Trib1	0.07119341	0.688496941	0.162097985
Gzmc	0.102939246	0.688496941	0.162097985

Evc2	0.15619434	0.688496941	0.162097985
Tnfsf13os	0.083533466	0.688496941	0.162097985
Nudt7	-0.646829473	0.689472373	0.161483131
Gprasp2	-0.311541033	0.689472373	0.161483131
Oprl1	-0.275472569	0.689472373	0.161483131
Hykk	-0.279451523	0.689472373	0.161483131
G6pdx	-0.140062997	0.689472373	0.161483131
Mocs1	0.503716574	0.689472373	0.161483131
Cnga2	0.153938148	0.689472373	0.161483131
Jam2	-0.087146479	0.689472373	0.161483131
Upf2	0.133482603	0.689472373	0.161483131
Irx3	-0.133407565	0.689472373	0.161483131
Whrn	0.124160407	0.689472373	0.161483131
Fam234b	0.110755833	0.689472373	0.161483131
Atp6v1c2	0.164313564	0.689472373	0.161483131
Nme6	-0.111001597	0.689472373	0.161483131
Gulp1	0.130908269	0.689472373	0.161483131
F5	0.255481465	0.689472373	0.161483131
Srgap3	0.087995943	0.689472373	0.161483131
Cd3g	0.149710142	0.689472373	0.161483131
Gpr63	0.102245748	0.689472373	0.161483131
Pcdha9	-0.141324749	0.689472373	0.161483131
Rpl7a	-0.074116193	0.689472373	0.161483131
Rfx2	0.087039866	0.689472373	0.161483131
Boc	0.084807895	0.689472373	0.161483131
Lrrc9	0.145295412	0.689472373	0.161483131
Mir17hg	0.089186186	0.689472373	0.161483131
Hdac1	0.17283001	0.689472373	0.161483131
Ankrd13d	-0.188595805	0.689472373	0.161483131
Rexo2	0.186174968	0.689472373	0.161483131
Cers4	-0.147703667	0.689472373	0.161483131
Tmem212	-0.067547587	0.689472373	0.161483131
Arhgap36	0.155112548	0.689472373	0.161483131
Mospd2	0.205548712	0.689472373	0.161483131
Zbtb2	0.084291377	0.690022675	0.161136637
Abhd8	-0.218322789	0.690675699	0.160725824
Cyp4f15	-0.152932786	0.690675699	0.160725824
Tm7sf3	0.144039596	0.690675699	0.160725824
Zfp820	0.11015681	0.690675699	0.160725824
Tlcd2	0.165706093	0.690675699	0.160725824
Fam89b	-0.212021239	0.690741498	0.160684452
Rgs17	-0.232668062	0.690741498	0.160684452
Amigo2	0.134904387	0.690741498	0.160684452
Phpt1	-0.158785968	0.690741498	0.160684452
Wls	0.169462583	0.690741498	0.160684452
Pfkfb4	0.117704875	0.690741498	0.160684452
Erbb4	-0.184038072	0.690741498	0.160684452
Il1b	0.09973169	0.690741498	0.160684452

Acer2	0.174737697	0.690741498	0.160684452
Neu2	0.142823411	0.690741498	0.160684452
Tmem168	0.119199916	0.690741498	0.160684452
Atp6v0a1	0.134579027	0.690741498	0.160684452
Lta	-0.121372289	0.690741498	0.160684452
Ttc12	0.09757523	0.690741498	0.160684452
Slc41a1	0.084244971	0.690741498	0.160684452
Scube2	0.090452952	0.690741498	0.160684452
Bax	0.121905501	0.690741498	0.160684452
Mog	0.109309996	0.690741498	0.160684452
Soga3	0.1017587	0.690741498	0.160684452
Zfp846	0.092026346	0.690741498	0.160684452
Sertm1	-0.114957237	0.690741498	0.160684452
C1qtnf9	-0.194865736	0.690741498	0.160684452
Pitpnc1	0.101904231	0.690741498	0.160684452
Gdf7	-0.132756874	0.690741498	0.160684452
Cpeb3	-0.154730724	0.690741498	0.160684452
Ccdc22	0.07563266	0.690741498	0.160684452
Spats2l	0.123315838	0.690741498	0.160684452
Tspyl1	-0.169865261	0.690741498	0.160684452
Synj1	0.099656406	0.690741498	0.160684452
Stra6	-0.147663432	0.690741498	0.160684452
Foxo6	0.123107342	0.690741498	0.160684452
Traf1	-0.202731334	0.690741498	0.160684452
Capn12	0.131004428	0.690741498	0.160684452
Cdh22	0.086215591	0.690741498	0.160684452
Kif3c	0.093073645	0.690865755	0.160606334
Pnma1	0.144648336	0.690865755	0.160606334
Nyap2	0.115503342	0.690865755	0.160606334
Artn	-0.372512576	0.690976239	0.160536887
Gpsm2	-0.31895747	0.690976239	0.160536887
Stmn3	-0.079033202	0.690976239	0.160536887
Srpk3	-0.251268068	0.690976239	0.160536887
Ttc23	0.152642185	0.690976239	0.160536887
Slco4c1	0.111725139	0.690976239	0.160536887
Iqsec2	0.185519554	0.691200034	0.160396249
Dlg4	0.133211783	0.691200034	0.160396249
Magee1	-0.123185081	0.691200034	0.160396249
Rhof	0.091977377	0.691200034	0.160396249
BC030336	-0.1105868	0.691200034	0.160396249
Nfya	-0.095484649	0.691200034	0.160396249
Rundc3a	0.147415119	0.691200034	0.160396249
Ccdc121	0.270962306	0.691200034	0.160396249
Cryga	-0.090945238	0.691200034	0.160396249
Opcml	-0.121196293	0.691200034	0.160396249
Plekhm3	0.193348705	0.691200034	0.160396249
Tigd5	0.150592002	0.691200034	0.160396249
Ptpru	-0.152220727	0.691573929	0.160161387

Fmo5	0.11398614	0.691573929	0.160161387
Twsg1	-0.331879592	0.691948799	0.15992604
Chst8	-0.41337999	0.691948799	0.15992604
Engase	0.108234388	0.691948799	0.15992604
Cox8b	-0.16073138	0.691948799	0.15992604
Kcnj12	-0.315074197	0.691948799	0.15992604
Alkbh6	-0.176477718	0.691948799	0.15992604
Cyp2f2	0.107922348	0.691948799	0.15992604
Tnfrsf19	0.180637882	0.691948799	0.15992604
Rpe65	0.212675056	0.691948799	0.15992604
Sobp	-0.088092639	0.691948799	0.15992604
Galnt18	0.104502245	0.691948799	0.15992604
Zfp710	0.198960539	0.691948799	0.15992604
CCDC129	-0.133490512	0.691948799	0.15992604
Serpinb9b	0.116132936	0.691948799	0.15992604
Uts2b	0.128767056	0.691948799	0.15992604
Wnt7a	0.08895993	0.691948799	0.15992604
Cinp	0.180217106	0.691948799	0.15992604
Nsmf	0.11606241	0.691948799	0.15992604
Pafah2	0.09395419	0.691948799	0.15992604
Phf21b	-0.076576115	0.691948799	0.15992604
Susd4	0.101794737	0.691948799	0.15992604
Wwc1	0.105086283	0.691948799	0.15992604
Adhfe1	0.068923556	0.691948799	0.15992604
Crybg3	0.215992447	0.691948799	0.15992604
Apba3	-0.153546337	0.691948799	0.15992604
Zfp296	-0.077418031	0.691948799	0.15992604
Adgra2	-0.065910413	0.691948799	0.15992604
Prss43	0.296744299	0.691948799	0.15992604
Ifitm7	0.095736941	0.691948799	0.15992604
Rab38	0.15935514	0.69284091	0.159366477
Hacl1	-0.362147984	0.693646987	0.158861496
Lztfl1	-0.364146819	0.693717235	0.158817515
Dek	0.190027946	0.693717235	0.158817515
Haus5	-0.130729264	0.695843159	0.157488638
HIST2H2BI	-0.308667386	0.696072921	0.157345261
Arhgef4	-0.282367864	0.696072921	0.157345261
Golt1a	0.135996634	0.696072921	0.157345261
Nsf	0.326048645	0.696072921	0.157345261
Zfp385b	0.171938899	0.696072921	0.157345261
Myh7	0.168711151	0.696072921	0.157345261
AK010878	-0.22858592	0.696072921	0.157345261
Pnma3	-0.080605403	0.696072921	0.157345261
Rap1a	0.267146014	0.696072921	0.157345261
Yes1	-0.139503476	0.696072921	0.157345261
Cend1	0.127692143	0.696072921	0.157345261
Cdh8	0.15238906	0.696072921	0.157345261
Tmem79	0.211036567	0.696072921	0.157345261

Cyb5rl	0.072105687	0.696072921	0.157345261
L1cam	0.142446744	0.696072921	0.157345261
Rbms2	0.123754822	0.696072921	0.157345261
Cyp7b1	0.20033137	0.696072921	0.157345261
Antxr1	0.112581858	0.696072921	0.157345261
Zfpm1	-0.106910034	0.696072921	0.157345261
Gucy1a2	-0.261166936	0.696072921	0.157345261
Atp6v1g2	0.131891252	0.696072921	0.157345261
Rara	0.084141813	0.696072921	0.157345261
Vpreb3	0.072780025	0.696072921	0.157345261
Sspn	-0.104444099	0.696072921	0.157345261
Mknk1	-0.218097185	0.696072921	0.157345261
Acer3	0.101667483	0.696072921	0.157345261
Eif5a2	0.119786516	0.696072921	0.157345261
Bcat1	0.156714431	0.696072921	0.157345261
Chic2	0.120230369	0.696072921	0.157345261
Dpm1	-0.166201055	0.696072921	0.157345261
Cd4	-0.219741853	0.696072921	0.157345261
Sdc3	0.25920251	0.696072921	0.157345261
BZRAP1	-0.098239516	0.696072921	0.157345261
Atp2b3	0.127219622	0.696072921	0.157345261
HRASLS	0.148850836	0.696072921	0.157345261
Map3k13	-0.153941779	0.696072921	0.157345261
B3galnt1	-0.20261424	0.696072921	0.157345261
HIST1H1D	0.097359125	0.696072921	0.157345261
Alg5	0.102850231	0.696072921	0.157345261
Ibsp	-0.137853161	0.696072921	0.157345261
Cdk10	0.107024458	0.696072921	0.157345261
Moap1	-0.17939454	0.696072921	0.157345261
Trp53	0.110293511	0.696072921	0.157345261
Snap91	0.158963597	0.696072921	0.157345261
Zmpste24	-0.102469249	0.696072921	0.157345261
Hbq1a	-0.099670893	0.696072921	0.157345261
Pcdhgc4	0.142251163	0.696072921	0.157345261
Usf3	0.134706631	0.696072921	0.157345261
Myo6	0.192477135	0.696072921	0.157345261
Sco2	-0.103921396	0.696072921	0.157345261
Clec16a	-0.112521727	0.696072921	0.157345261
Ccdc92	0.059034707	0.696072921	0.157345261
Mfap3l	0.070675441	0.696072921	0.157345261
Dcaf5	0.2198457	0.696072921	0.157345261
Herc2	0.126640869	0.696072921	0.157345261
Sestd1	0.39787098	0.696072921	0.157345261
Lrrn3	0.126486618	0.696072921	0.157345261
Rb1cc1	0.230136973	0.696072921	0.157345261
Gstm2	-0.120782794	0.696072921	0.157345261
Usp13	0.24194479	0.696072921	0.157345261
Smpd2	0.165047284	0.696072921	0.157345261

Myt1l	-0.149529926	0.696072921	0.157345261
Acap1	0.149352418	0.696072921	0.157345261
Lingo2	0.129619546	0.696072921	0.157345261
Ubl4a	0.253158666	0.696072921	0.157345261
Tspan33	-0.108301261	0.696072921	0.157345261
Ap1s3	-0.097038446	0.696072921	0.157345261
Smim8	0.120994444	0.696072921	0.157345261
Prlr	-0.123045626	0.696072921	0.157345261
Plxna1	-0.07433865	0.696072921	0.157345261
Plppr5	0.105345209	0.696465181	0.157100591
Ephb2	0.075242537	0.696473242	0.157095564
Il6st	-0.426226998	0.697529118	0.156437659
Gli3	-0.129889987	0.697529118	0.156437659
Nat2	-0.315069242	0.697529118	0.156437659
Ttc39aos1	0.137918425	0.697529118	0.156437659
Samd10	0.096939063	0.697529118	0.156437659
Mfsd7a	0.182776497	0.697529118	0.156437659
Cd69	0.249888597	0.697529118	0.156437659
Acadvl	0.063499693	0.697529118	0.156437659
Pank4	-0.083593181	0.697529118	0.156437659
Sacs	0.100737065	0.697529118	0.156437659
Gda	0.1349378	0.697529118	0.156437659
Trp53bp1	0.157634874	0.698375836	0.155910796
Aars2	-0.079926675	0.698375836	0.155910796
Mgst3	0.201898279	0.698494901	0.15583676
Bbs1	-0.099180163	0.698494901	0.15583676
Galntl6	0.121228691	0.698494901	0.15583676
Fbxo9	-0.173531689	0.698494901	0.15583676
Particl	0.125047515	0.698494901	0.15583676
Ap3m2	0.262739062	0.698494901	0.15583676
Rab3a	0.153126085	0.698494901	0.15583676
Pde5a	-0.074210555	0.699011354	0.15551577
Ceacam2	0.177743553	0.699274066	0.155352578
Tead1	0.071278466	0.699535853	0.155190022
Hsd11b2	-0.111162082	0.699784358	0.155035769
Fads3	0.368774818	0.700406461	0.154649856
Qrfpr	-0.145276132	0.700406461	0.154649856
Psd	-0.096330482	0.700406461	0.154649856
Adora2a	-0.367402744	0.700406461	0.154649856
HIST1H2BI	0.09691595	0.700406461	0.154649856
Zfp738	-0.09361441	0.700406461	0.154649856
Scgn	0.080510831	0.700406461	0.154649856
Scn2b	0.143919028	0.700406461	0.154649856
Stmn1	0.125309508	0.700406461	0.154649856
Mapk8ip3	-0.111418646	0.700406461	0.154649856
Gcnt2	-0.106169966	0.700406461	0.154649856
Pde1a	-0.099919881	0.700406461	0.154649856
Lmx1a	0.075842493	0.700406461	0.154649856

Sell	0.116690394	0.700406461	0.154649856
Chac1	0.071345918	0.700406461	0.154649856
Rpusd1	-0.117198089	0.700406461	0.154649856
Tcf25	-0.123760495	0.700406461	0.154649856
Otud7a	-0.166480494	0.70111443	0.154211094
Purb	-0.131813117	0.70136971	0.154052994
Kcnmb2	-0.194948464	0.702199579	0.153539435
Slc6a20a	-0.192321877	0.70247301	0.153370358
Slc8a2	0.07557599	0.70247301	0.153370358
Pitpna	-0.25983013	0.702475095	0.153369068
Zbtb12	-0.29557817	0.702475095	0.153369068
Pcdhb4	0.143858867	0.702475095	0.153369068
Glyctk	-0.160004385	0.702475095	0.153369068
Rps11	0.114812692	0.702475095	0.153369068
Nt5e	0.086119495	0.702475095	0.153369068
B9d1os	0.141191763	0.702475095	0.153369068
FAM160A1	0.267114971	0.702576404	0.15330644
Nkiras2	0.120999823	0.702576404	0.15330644
Acsbg1	0.12590429	0.702576404	0.15330644
Iqcf5	0.17954135	0.70260954	0.153285957
Sgsm2	-0.290056423	0.70260954	0.153285957
Ush1g	-0.24770659	0.70260954	0.153285957
Pantr2	-0.438799142	0.70260954	0.153285957
Sod2	0.278216328	0.70260954	0.153285957
Ablim3	1.639959219	0.70260954	0.153285957
Fau	-0.363359012	0.70260954	0.153285957
Tmed5	-0.32193214	0.70260954	0.153285957
Vwc2	-0.393320104	0.70260954	0.153285957
Pfkfb3	-0.308055529	0.70260954	0.153285957
Zfp456	-0.173695149	0.70260954	0.153285957
Kcnd1	-0.259604917	0.70260954	0.153285957
Onecut3	-0.283177327	0.70260954	0.153285957
Snx7	-0.160979423	0.70260954	0.153285957
Lrrc8b	-0.198726337	0.70260954	0.153285957
Alas2	-0.294550565	0.70260954	0.153285957
Crispld2	0.18751894	0.70260954	0.153285957
Ppp3r1	0.318051605	0.70260954	0.153285957
FAM73B	-0.103238816	0.70260954	0.153285957
Sirt6	-0.249157745	0.70260954	0.153285957
Yap1	0.260104358	0.70260954	0.153285957
Map3k12	-0.115051373	0.70260954	0.153285957
Ctnna2	0.127979092	0.70260954	0.153285957
Rgma	0.144689761	0.70260954	0.153285957
Ywhag	0.139491273	0.70260954	0.153285957
Gpt2	-0.238561357	0.70260954	0.153285957
Adipor1	-0.166969319	0.70260954	0.153285957
Map2k4	-0.115519825	0.70260954	0.153285957
Cntrl	0.084779204	0.70260954	0.153285957

Gabrq	-0.323371376	0.70260954	0.153285957
Pdgfrb	0.303745946	0.70260954	0.153285957
Myo3b	-0.1415029	0.70260954	0.153285957
Oprk1	-0.379964863	0.70260954	0.153285957
Plcxd1	-0.251936018	0.70260954	0.153285957
B3galt5	0.187279405	0.70260954	0.153285957
ELMSAN1	-0.248391689	0.70260954	0.153285957
Rangrf	-0.153852777	0.70260954	0.153285957
Gnai1	0.204116056	0.70260954	0.153285957
Slc30a4	0.166201203	0.70260954	0.153285957
Nrn1l	-0.281168207	0.70260954	0.153285957
Rps6ka2	0.14342526	0.70260954	0.153285957
Upp2	-0.139491105	0.70260954	0.153285957
Map3k11	-0.172554278	0.70260954	0.153285957
Map3k5	-0.088350592	0.70260954	0.153285957
Optn	-0.121784895	0.70260954	0.153285957
BE692007	-0.14471782	0.70260954	0.153285957
Gch1	-0.085636592	0.70260954	0.153285957
AI182371	0.234587152	0.70260954	0.153285957
Bche	0.194771832	0.70260954	0.153285957
AA415398	0.123746301	0.70260954	0.153285957
Gnb5	-0.121717808	0.70260954	0.153285957
Cnnm1	-0.416591652	0.70260954	0.153285957
Rgsl1	-0.1804312	0.70260954	0.153285957
Slc46a3	0.129591915	0.70260954	0.153285957
Tyro3	0.136148963	0.70260954	0.153285957
Als2cl	0.128054859	0.70260954	0.153285957
Tram1l1	-0.139228423	0.70260954	0.153285957
Ffar3	-0.173132019	0.70260954	0.153285957
Lrrc42	0.185723968	0.70260954	0.153285957
Bex1	0.321032175	0.70260954	0.153285957
Gas7	-0.118154766	0.70260954	0.153285957
Gpr61	-0.111946381	0.70260954	0.153285957
Traf6	0.164224835	0.70260954	0.153285957
Zfp180	0.24417566	0.70260954	0.153285957
Srd5a3	0.198639106	0.70260954	0.153285957
Rpl18a	0.151113214	0.70260954	0.153285957
Nrk	-0.213692868	0.70260954	0.153285957
Aff3	0.09123621	0.70260954	0.153285957
Slc3a2	0.144900959	0.70260954	0.153285957
Rpp25	-0.156650338	0.70260954	0.153285957
Gamt	-0.23208128	0.70260954	0.153285957
Rasal2	-0.194517184	0.70260954	0.153285957
Zyx	-0.288164758	0.70260954	0.153285957
Kcna1	-0.198710298	0.70260954	0.153285957
Ano2	-0.192492116	0.70260954	0.153285957
Dpp8	0.184380713	0.70260954	0.153285957
Pou2f2	0.365345482	0.70260954	0.153285957

Mrpl52	-0.14785717	0.70260954	0.153285957
Rasl11a	-0.123168116	0.70260954	0.153285957
Ppm1a	0.176235226	0.70260954	0.153285957
Lrp3	0.143793241	0.70260954	0.153285957
Grk5	0.132683546	0.70260954	0.153285957
Sh2b3	-0.072617789	0.70260954	0.153285957
Epb41l4b	0.091994196	0.70260954	0.153285957
Zfp81	-0.297637844	0.70260954	0.153285957
Ppl	-0.135661563	0.70260954	0.153285957
Lysmd1	0.244209926	0.70260954	0.153285957
Cdc42bpa	-0.132600628	0.70260954	0.153285957
Kat2b	0.107920659	0.70260954	0.153285957
Pcdh12	-0.08614104	0.70260954	0.153285957
BC055402	0.188168764	0.70260954	0.153285957
Commd1	0.100599112	0.70260954	0.153285957
Gldc	0.130350142	0.70260954	0.153285957
Hunk	0.320265769	0.70260954	0.153285957
Abca5	-0.235903731	0.70260954	0.153285957
Insig1	-0.143753234	0.70260954	0.153285957
Nudt3	0.130410246	0.70260954	0.153285957
Asphd2	0.076145355	0.70260954	0.153285957
Bcl2l2	0.105724042	0.70260954	0.153285957
Ccdc73	0.084672996	0.70260954	0.153285957
Stmn2	0.23783159	0.70260954	0.153285957
Ypel4	-0.157206078	0.70260954	0.153285957
Dock10	-0.113540161	0.70260954	0.153285957
Ccdc166	0.185111615	0.70260954	0.153285957
Npvf	-0.133399162	0.70260954	0.153285957
Dram2	0.099464536	0.70260954	0.153285957
Tmem82	0.08992163	0.70260954	0.153285957
Wdr90	-0.081346572	0.70260954	0.153285957
Gpr158	-0.22239197	0.70260954	0.153285957
Kcns2	-0.135931519	0.70260954	0.153285957
Rpl27	-0.076034638	0.70260954	0.153285957
Asnsd1	0.100519652	0.70260954	0.153285957
Dsg1c	0.073331181	0.70260954	0.153285957
Rab3c	-0.205621679	0.70260954	0.153285957
Rap1gds1	0.10015353	0.70260954	0.153285957
Katnal1	-0.086946186	0.70260954	0.153285957
Mcm4	0.19318025	0.70260954	0.153285957
Daglb	0.108024096	0.70260954	0.153285957
AW01173[illegible]	0.104941872	0.70260954	0.153285957
KDELC2	0.11571713	0.70260954	0.153285957
Socs7	-0.166344873	0.70260954	0.153285957
Nmt2	0.110117829	0.70260954	0.153285957
Tsnax	0.083055885	0.70260954	0.153285957
Acss3	-0.086953184	0.70260954	0.153285957
Grik3	0.092516679	0.70260954	0.153285957

Apol6	-0.170449888	0.70260954	0.153285957
Cbx6	0.124547771	0.70260954	0.153285957
Rab44	0.159697081	0.70260954	0.153285957
HIST1H4M	-0.129027332	0.70260954	0.153285957
Adgrl1	0.229271126	0.70260954	0.153285957
Lamb2	-0.076714024	0.70260954	0.153285957
Mapt	0.109283555	0.70260954	0.153285957
Bhlhe23	-0.111878233	0.70260954	0.153285957
Zbtb45	0.098693493	0.70260954	0.153285957
Gpr179	-0.152462036	0.70260954	0.153285957
Zfp27	0.090899276	0.70260954	0.153285957
Fam149a	-0.121114463	0.70260954	0.153285957
Ammecr1	0.104373664	0.70260954	0.153285957
Celf5	0.215853392	0.70260954	0.153285957
Cacna1b	0.194670297	0.70260954	0.153285957
Exog	-0.150390405	0.70260954	0.153285957
Csn3	-0.086851033	0.70260954	0.153285957
Tsen54	0.141791503	0.70260954	0.153285957
Colq	0.302086965	0.70260954	0.153285957
Dlx6	0.085032044	0.70260954	0.153285957
Rab5c	0.184047691	0.70260954	0.153285957
Akap13	-0.160952005	0.70260954	0.153285957
Pam	0.120451848	0.70260954	0.153285957
Eef1a1	-0.102321228	0.70260954	0.153285957
Mat2b	0.097111456	0.70260954	0.153285957
Btn1a1	0.123032965	0.70260954	0.153285957
Cspg4	0.070301193	0.70260954	0.153285957
Coro2b	0.314900597	0.70260954	0.153285957
Rab15	0.155969815	0.70260954	0.153285957
Arrdc1	0.093686014	0.70260954	0.153285957
Fth1	0.150775605	0.70260954	0.153285957
Kng2	0.176302592	0.70260954	0.153285957
Echs1	0.103818677	0.70260954	0.153285957
Asb11	0.123217628	0.70260954	0.153285957
Cyb5r2	-0.109199725	0.70260954	0.153285957
WDR63	0.089016755	0.70260954	0.153285957
Depdc5	0.092807387	0.70260954	0.153285957
Btf3	0.120092551	0.70260954	0.153285957
Gprasp1	0.106961381	0.70260954	0.153285957
Sostdc1	-0.095300503	0.70260954	0.153285957
Mir670hg	0.203441558	0.70260954	0.153285957
Amph	0.095128494	0.70260954	0.153285957
Mapre2	-0.09323431	0.70260954	0.153285957
Mir124-2h	0.108650875	0.70260954	0.153285957
Med13l	0.126124335	0.70260954	0.153285957
BC02406[illegible]	0.151437277	0.70260954	0.153285957
Sgca	0.243772147	0.70260954	0.153285957
Tmem169	0.099537407	0.70260954	0.153285957

Ptprd	0.095827037	0.70260954	0.153285957
Cdh7	0.08393452	0.70260954	0.153285957
Zfp689	0.121385268	0.70260954	0.153285957
Amd1	0.105371315	0.70260954	0.153285957
Actl6a	-0.10788209	0.70260954	0.153285957
Ceacam1	-0.083367351	0.70260954	0.153285957
Reep2	0.111870184	0.70260954	0.153285957
Psmb10	-0.159376322	0.70260954	0.153285957
Odc1	-0.109316821	0.70260954	0.153285957
Kcnd3os	0.107657534	0.70260954	0.153285957
Comp	0.105757894	0.70260954	0.153285957
Map10	-0.10092863	0.70260954	0.153285957
Opa3	0.167635395	0.70260954	0.153285957
Irgq	0.086622256	0.70260954	0.153285957
Tmem130	0.084760384	0.70260954	0.153285957
Txndc5	0.110289956	0.70260954	0.153285957
Rassf2	-0.070652424	0.70260954	0.153285957
Extl3	0.117930183	0.70260954	0.153285957
Slc35f3	0.099497914	0.70260954	0.153285957
Itih5	0.131668253	0.70260954	0.153285957
Sphkap	0.185740816	0.70260954	0.153285957
Ttc9	0.177700071	0.70260954	0.153285957
Asxl3	0.133435996	0.70260954	0.153285957
Ppp2r3d	-0.155196226	0.70260954	0.153285957
Primpol	-0.107774096	0.70260954	0.153285957
Tmem88b	0.131717277	0.70260954	0.153285957
Sprn	-0.089087727	0.70260954	0.153285957
Itm2a	0.080689965	0.70260954	0.153285957
Prox1	-0.085223971	0.70260954	0.153285957
Prkce	-0.216411078	0.70260954	0.153285957
Gabrr2	-0.14429258	0.70260954	0.153285957
Rac3	0.191509261	0.70260954	0.153285957
Dnm3	0.092142615	0.70260954	0.153285957
Fgfr1op2	0.125529968	0.70260954	0.153285957
Fat3	-0.055868181	0.70260954	0.153285957
Gnl1	0.187308617	0.70260954	0.153285957
Klrb1c	0.113791486	0.70260954	0.153285957
Chst5	0.116672401	0.70260954	0.153285957
Diras1	-0.128005427	0.70260954	0.153285957
Mapk8ip2	-0.177592519	0.70260954	0.153285957
Pianp	0.099540693	0.70260954	0.153285957
Tpmt	-0.089901023	0.70260954	0.153285957
Ralgds	-0.09284538	0.70260954	0.153285957
Dclk1	-0.075855802	0.70260954	0.153285957
Slc16a4	-0.202111521	0.70260954	0.153285957
Pttg1ip	-0.070019883	0.70260954	0.153285957
Gsg1l	0.092579023	0.70260954	0.153285957
Tox	-0.120924195	0.70260954	0.153285957

Cep128	-0.071730534	0.70260954	0.153285957
Creb5	-0.098345577	0.70260954	0.153285957
Mroh3	0.127309468	0.70260954	0.153285957
AI987944	-0.10961615	0.70260954	0.153285957
Fndc5	0.080862179	0.70260954	0.153285957
Ilk	0.108710695	0.70260954	0.153285957
Rps28	-0.195948781	0.70260954	0.153285957
Uqcc3	-0.168026408	0.70260954	0.153285957
Ptn	-0.092728058	0.70260954	0.153285957
Cage1	-0.108080148	0.70260954	0.153285957
Arrdc3	0.08934436	0.70260954	0.153285957
Nckipsd	-0.150893779	0.70260954	0.153285957
Cdk5rap1	-0.201445366	0.70260954	0.153285957
Ap5b1	0.088796529	0.70260954	0.153285957
Mup2	0.107404655	0.70260954	0.153285957
Gtf2h2	0.120670033	0.70260954	0.153285957
Mmp24	0.075504664	0.70260954	0.153285957
Trp53bp2	0.109021665	0.70260954	0.153285957
Slco1b2	0.130440838	0.70260954	0.153285957
Mak	-0.084923774	0.70260954	0.153285957
Nxnl2	0.104542574	0.70260954	0.153285957
Zfp341	0.134026695	0.70260954	0.153285957
Smad1	0.216602073	0.70260954	0.153285957
Rmst	-0.18558091	0.70260954	0.153285957
Jakmip2	0.117226597	0.70260954	0.153285957
Prickle2	0.096334588	0.70260954	0.153285957
Lrrc24	0.143162171	0.70260954	0.153285957
Hilpda	0.183630383	0.70260954	0.153285957
Ublcp1	0.084508918	0.70260954	0.153285957
Bace2	0.092301522	0.70260954	0.153285957
Slc26a2	-0.164945449	0.70260954	0.153285957
Syngr3	0.074026761	0.70260954	0.153285957
Adgre5	0.073840773	0.70260954	0.153285957
Rab39	0.067449309	0.70260954	0.153285957
Cdh10	0.153669027	0.70260954	0.153285957
Gpr19	-0.126788619	0.70260954	0.153285957
Grm1	0.202900554	0.70260954	0.153285957
PNMAL1	0.113627713	0.70260954	0.153285957
Barhl2	0.129389307	0.70260954	0.153285957
Tor1aip2	0.12271923	0.70260954	0.153285957
GGNBP2O:	0.121775811	0.70260954	0.153285957
Rnf11	0.145539742	0.70260954	0.153285957
Ttc19	0.101597512	0.70260954	0.153285957
Tbc1d9	-0.116931885	0.70260954	0.153285957
Kif1c	-0.175844858	0.70260954	0.153285957
Ston2	-0.088980095	0.70260954	0.153285957
Lnx1	0.087025151	0.70260954	0.153285957
Adamts13	0.110485454	0.70260954	0.153285957

Osbp2	0.096625411	0.70260954	0.153285957
Gapt	0.090975067	0.70260954	0.153285957
Sirt3	0.063768497	0.70260954	0.153285957
Ppp5c	-0.085009722	0.702678399	0.153243397
Baalc	0.100004008	0.703558122	0.152700019
Slc36a1	-0.354177719	0.703642719	0.152647802
Cdk14	-0.251386054	0.703642719	0.152647802
Ugp2	0.14800404	0.703642719	0.152647802
Yaf2	0.143713975	0.703642719	0.152647802
Snrpe	-0.215313396	0.703772356	0.152567796
Bhlhe40	0.135201545	0.703772356	0.152567796
Mmp17	-0.106983509	0.703772356	0.152567796
Hif1a	0.090877941	0.704204766	0.15230104
Igdcc4	-0.135898768	0.704204766	0.15230104
Tspan8	0.084184172	0.704204766	0.15230104
Htr1f	0.131495179	0.704204766	0.15230104
Ginm1	0.14855607	0.704249758	0.152273294
Slc35f2	0.111994897	0.704249758	0.152273294
Rnf223	-0.241845663	0.704924345	0.151857491
Rps16	-0.139393947	0.704924345	0.151857491
Tab3	-0.071232999	0.704924345	0.151857491
Jrk	-0.12861599	0.704924345	0.151857491
Zfp7	0.18192539	0.704924345	0.151857491
Zmynd8	0.128651983	0.704924345	0.151857491
Pvr	0.142337935	0.704924345	0.151857491
AKAP2	-0.12147517	0.704924345	0.151857491
Cd300e	-0.093079624	0.704924345	0.151857491
Il18	-0.081371464	0.704924345	0.151857491
Tango2	0.157226566	0.704924345	0.151857491
Pcbd2	0.072625699	0.70502297	0.151796733
Rnf6	0.229680679	0.705430431	0.151545809
Wnk3	0.149105571	0.705430431	0.151545809
Zfp69	-0.116377048	0.705430431	0.151545809
Myb	0.260822732	0.705430431	0.151545809
Dgkh	0.311108212	0.705458031	0.151528818
AW55198	0.081900475	0.705696809	0.151381846
Srpk2	0.139954152	0.706032446	0.15117534
Rmnd5a	0.237720164	0.706071671	0.151151213
Olfr461	-0.097883763	0.706071671	0.151151213
Ociad2	-0.20825064	0.706157008	0.151098727
Rnf215	-0.313704245	0.706157008	0.151098727
Dio3	0.096757276	0.706157008	0.151098727
Ppbp	0.127960307	0.706679209	0.150777686
Smarca2	0.212897549	0.706703493	0.150762762
Pfn4	0.08781458	0.706703493	0.150762762
Lao1	0.168180579	0.706804333	0.150700797
Nxpe3	0.170391792	0.706804333	0.150700797
Pcmt1	0.136214785	0.706904451	0.150639284

Hspa12b	-0.135396474	0.706936488	0.150619602
Ttc28	-0.11430756	0.706936488	0.150619602
Adam10	-0.157093723	0.706936488	0.150619602
Mad2l2	0.08759058	0.706936488	0.150619602
Mtfp1	0.188912643	0.706936488	0.150619602
Ppp2r5e	0.20395722	0.706936488	0.150619602
Meg3	0.236762515	0.706936488	0.150619602
Syt4	0.262318805	0.706936488	0.150619602
Il11	0.073858591	0.706936488	0.150619602
Rfx4	-0.202108517	0.707118693	0.150507682
Zfp951	0.084513153	0.707118693	0.150507682
Mcpt2	0.095294032	0.707367577	0.150354851
Scn7a	0.161296047	0.708230646	0.149825284
Btbd16	0.181213848	0.708230646	0.149825284
Pitpnm2	0.106462447	0.708230646	0.149825284
Fam189b	0.13817285	0.708230646	0.149825284
Irs4	-0.132294422	0.708230646	0.149825284
Megf10	0.095132585	0.708230646	0.149825284
Aldh4a1	-0.262096701	0.708360173	0.149745865
Tmem8b	-0.136603229	0.708360173	0.149745865
Ipo9	0.397377518	0.708360173	0.149745865
Plekha6	-0.098105445	0.708360173	0.149745865
Entpd4	0.152585303	0.708360173	0.149745865
Olfr883	-0.097383155	0.708360173	0.149745865
Nadk2	-0.213442761	0.708993673	0.14935764
Ccl21a	-0.096386078	0.708993673	0.14935764
Elavl2	-0.104593055	0.708993673	0.14935764
Cast	0.133727964	0.708993673	0.14935764
Nicn1	0.096989555	0.708993673	0.14935764
Snx21	0.077821488	0.708993673	0.14935764
Ing3	0.129963582	0.708993673	0.14935764
Ift57	0.098042948	0.708993673	0.14935764
Isoc1	0.101605173	0.708993673	0.14935764
Rusc1	-0.17211929	0.709522307	0.149033946
Pkmyt1	-0.081748046	0.709522307	0.149033946
Nxph1	-0.277274704	0.709743071	0.148898838
Aox1	-0.392135932	0.709743071	0.148898838
Fbxo44	-0.097930472	0.709743071	0.148898838
Epm2aip1	0.123020909	0.709743071	0.148898838
Fam78a	0.167030392	0.709743071	0.148898838
Fgf1	0.079582287	0.709743071	0.148898838
Esrrg	0.106893862	0.709743071	0.148898838
Acbd7	0.134855561	0.709743071	0.148898838
Cib2	-0.076884903	0.709743071	0.148898838
Fsip1	-0.09814466	0.709743071	0.148898838
Pilrb2	0.07241531	0.709743071	0.148898838
Dnase1l2	-0.075427094	0.709913284	0.148794697
Zfp418	-0.334325734	0.710045067	0.148714085

Prlhr	-0.117376522	0.710066664	0.148700876
Pspc1	-0.080342411	0.710066664	0.148700876
Abcg1	0.163002758	0.710146337	0.148652149
Napg	-0.296551316	0.710618476	0.148363505
Snapin	-0.077176372	0.710618476	0.148363505
Timp2	-0.110306785	0.711610966	0.147757368
Polr2c	0.064379666	0.711610966	0.147757368
Ankrd28	0.127162963	0.711610966	0.147757368
Calm1	-0.123533098	0.711980536	0.147531879
Pja1	0.161101523	0.711997261	0.147521677
Syn3	-0.108316166	0.711997261	0.147521677
Six3	0.391534928	0.711997261	0.147521677
Syp	0.125194779	0.711997261	0.147521677
Etfa	0.663200471	0.711997261	0.147521677
Slc30a7	0.112890137	0.711997261	0.147521677
Relt	0.125090178	0.711997261	0.147521677
Sv2a	0.07364855	0.711997261	0.147521677
Zfp276	-0.119654304	0.711997261	0.147521677
DSCR3	-0.132182997	0.711997261	0.147521677
Pla2g4c-p	0.11703548	0.711997261	0.147521677
Necab1	0.101145478	0.711997261	0.147521677
FAM84A	0.293480729	0.711997261	0.147521677
Cbarp	0.31697645	0.711997261	0.147521677
CECR6	0.116585839	0.711997261	0.147521677
Ehbp1	-0.110903545	0.711997261	0.147521677
Slc25a19	-0.104944271	0.711997261	0.147521677
Mmel1	0.116832876	0.711997261	0.147521677
Glg1	-0.178159754	0.711997261	0.147521677
Nr2f1	-0.096370412	0.711997261	0.147521677
Wdfy3	0.092074948	0.711997261	0.147521677
HIST1H2A(	-0.11735316	0.711997261	0.147521677
Pcdhb5	0.101878875	0.711997261	0.147521677
Hps3	0.18578626	0.712217077	0.147387618
Acvr1b	-0.167964415	0.71231506	0.147327873
Fzd4	0.101471268	0.71231506	0.147327873
Dnajc25	0.109593025	0.71231506	0.147327873
Zbtb32	-0.150229253	0.71231506	0.147327873
Zfr	0.093364754	0.71231506	0.147327873
Frmd5	-0.084629819	0.71231506	0.147327873
Fpgs	0.261309347	0.71257447	0.147169742
Csnk1g2	0.222711837	0.71257447	0.147169742
Dnase1	0.221311026	0.71257447	0.147169742
Ntm	-0.099929337	0.712917192	0.146960912
Adgrl2	-0.253961753	0.712992041	0.146915318
St3gal6	-0.276419711	0.712992041	0.146915318
Nr2c2	-0.1618686	0.712992041	0.146915318
Ncs1	0.108188485	0.712992041	0.146915318
Trpc5	0.106424909	0.712992041	0.146915318

Sgip1	-0.156365799	0.712992041	0.146915318
Cyb5r1	-0.151005456	0.712992041	0.146915318
Nsa2	-0.19175377	0.712992041	0.146915318
Nav3	0.207766209	0.712992041	0.146915318
Fndc9	0.077584336	0.712992041	0.146915318
Dhrs13os	-0.064682407	0.712992041	0.146915318
Nae1	-0.132287223	0.712992041	0.146915318
Csad	0.155977992	0.712992041	0.146915318
Calm2	0.258456324	0.713486133	0.146614463
Ubl4b	-0.256493641	0.713608457	0.146540012
Rhobtb2	0.195959595	0.713789295	0.14642997
Josd1	-0.230855361	0.714052197	0.14627004
Thnsl1	0.08662066	0.714052197	0.14627004
Cyp51	0.131942829	0.714052197	0.14627004
Msh2	0.439128671	0.714052197	0.14627004
Pidd1	0.090274005	0.714052197	0.14627004
Ier5	0.11999861	0.714052197	0.14627004
Chrac1	0.317511248	0.714052197	0.14627004
Lancl3	0.092993657	0.714052197	0.14627004
Prss22	-0.075305668	0.714052197	0.14627004
Rangap1	0.080908118	0.714052197	0.14627004
Mapre3	0.148983057	0.714052334	0.146269957
Pitpnm1	0.202616207	0.714052334	0.146269957
Rnf14	0.120827878	0.714052334	0.146269957
Tacr1	-0.227211923	0.714087513	0.146248561
Igdcc3	-0.430476064	0.714087513	0.146248561
E2f7	0.245129757	0.714087513	0.146248561
Actl6b	-0.121823874	0.714087513	0.146248561
Gstt3	0.179362279	0.714087513	0.146248561
Snap29	0.120757967	0.714087513	0.146248561
Sh3d21	0.093707142	0.714087513	0.146248561
Git2	-0.135457336	0.714087513	0.146248561
Calhm2	0.123691643	0.714087513	0.146248561
Ctbs	-0.124099908	0.714087513	0.146248561
HIST1H2AF	0.143755348	0.714087513	0.146248561
Itgae	-0.096623444	0.714087513	0.146248561
Rgs7bp	0.064123599	0.714087513	0.146248561
Arhgap28	0.131725488	0.714087513	0.146248561
Cxcl12	0.123044557	0.714198193	0.146181253
Slc26a6	-0.341506051	0.714229244	0.146162372
Tbc1d8	-0.218102182	0.714229244	0.146162372
Pik3ip1	-0.227756901	0.714229244	0.146162372
Celf6	-0.261876545	0.714229244	0.146162372
Tnfsf14	-0.198792742	0.714229244	0.146162372
Kifap3	-0.640477811	0.714229244	0.146162372
Rpl35	0.200920639	0.714229244	0.146162372
Tmem38a	-2.290752937	0.714229244	0.146162372
Crtc1	-0.124767824	0.714229244	0.146162372

Birc6	-0.286500949	0.714229244	0.146162372
Ankub1	0.195762188	0.714229244	0.146162372
Degs1	-0.184845336	0.714229244	0.146162372
Slc19a3	0.117792579	0.714229244	0.146162372
Nbea	-0.155809784	0.714229244	0.146162372
Zfp239	-0.171158058	0.714229244	0.146162372
H2-Ke6	-0.208612922	0.714229244	0.146162372
Rbfox2	0.18592639	0.714229244	0.146162372
Sgcg	-0.11621427	0.714229244	0.146162372
Arhgef5	0.175071943	0.714229244	0.146162372
Tal2	-0.093710094	0.714229244	0.146162372
Rusc2	-0.178822429	0.714229244	0.146162372
Ctif	0.405752641	0.714229244	0.146162372
Spcs3	0.249530042	0.714229244	0.146162372
Podxl2	-0.202450744	0.714229244	0.146162372
Syna	0.103045684	0.714229244	0.146162372
Gli1	0.181550445	0.714229244	0.146162372
Apbb1	-0.210878116	0.714229244	0.146162372
Cxcr2	0.112993149	0.714229244	0.146162372
Efcab9	0.105746446	0.714229244	0.146162372
Hsf1	0.109342415	0.714229244	0.146162372
Brdt	0.167542658	0.714229244	0.146162372
Haglr	0.107713348	0.714229244	0.146162372
Helb	-0.287754483	0.714229244	0.146162372
Klhl29	0.069443941	0.714229244	0.146162372
Rpl29	-0.091501943	0.714229244	0.146162372
Stk32a	0.135253565	0.714229244	0.146162372
Cryz	-0.086333988	0.714229244	0.146162372
Crebl2	0.105876473	0.714229244	0.146162372
Slc31a1	0.075971397	0.714229244	0.146162372
Pla2g4b	0.104742131	0.714229244	0.146162372
Rftn2	-0.105423938	0.714229244	0.146162372
Rnf208	0.085450984	0.714229244	0.146162372
Mansc4	0.094315987	0.714229244	0.146162372
Neurog1	-0.126645141	0.714229244	0.146162372
Kif17	-0.081585944	0.714229244	0.146162372
Ppm1h	0.081464784	0.714229244	0.146162372
Rhobtb1	0.196107315	0.714229244	0.146162372
Ptprcap	0.118934937	0.714229244	0.146162372
Pemt	0.093117089	0.714229244	0.146162372
Rhox5	0.137822477	0.714229244	0.146162372
Tstd3	-0.086872281	0.714229244	0.146162372
Magi1	-0.075032289	0.714229244	0.146162372
Appbp2	0.180763498	0.714229244	0.146162372
Med14	0.103725917	0.714229244	0.146162372
Gpr3	0.184030626	0.714229244	0.146162372
Atp2a3	0.149432876	0.714229244	0.146162372
HIST1H2BI	-0.164833071	0.714229244	0.146162372

Sqle	0.223217457	0.714229244	0.146162372
Camk1d	-0.19831731	0.714229244	0.146162372
Ubtd2	0.089333128	0.714229244	0.146162372
Vps50	-0.094089	0.714229244	0.146162372
Setdb2	0.072641116	0.714229244	0.146162372
Abca8a	0.098072648	0.714229244	0.146162372
Meis1	-0.264453956	0.714229244	0.146162372
Lrrtm1	0.19466031	0.714229244	0.146162372
Slit2	0.134759483	0.714229244	0.146162372
Klrg2	0.263613011	0.714229244	0.146162372
Dnajc5	0.093361759	0.714229244	0.146162372
Raver1	0.085514385	0.714229244	0.146162372
Dync1i1	0.100075688	0.714229244	0.146162372
Pld6	-0.058817906	0.714229244	0.146162372
Map3k15	0.139209273	0.714229244	0.146162372
Mdn1	-0.197732452	0.714229244	0.146162372
Mamld1	-0.10469921	0.714229244	0.146162372
Sytl1	0.098573154	0.714229244	0.146162372
Car7	-0.123642134	0.714873012	0.145771098
Pik3c2a	0.095449812	0.714873012	0.145771098
Kif1a	0.12449642	0.715169085	0.145591267
Spock2	0.105165185	0.715692268	0.145273675
GUCY1B3	-0.28961499	0.716110671	0.145019855
Zfp966	-0.156976442	0.716611087	0.144716477
Fam168b	0.098962003	0.716611087	0.144716477
Ppfibp2	-0.292011229	0.716691103	0.144667987
Matn1	0.123510207	0.716691103	0.144667987
Kpna1	-0.157538438	0.716691103	0.144667987
Atg10	-0.13877808	0.716691103	0.144667987
Banf2os	0.223419507	0.716691103	0.144667987
Limk1	-0.173299645	0.716691103	0.144667987
Svop	-0.211186536	0.716691103	0.144667987
Nme2	-0.114988246	0.716691103	0.144667987
Ing2	0.102241506	0.716691103	0.144667987
Dbndd1	-0.084931587	0.716691103	0.144667987
Ccdc40	-0.295552087	0.716691103	0.144667987
Amdhd1	0.089388884	0.716691103	0.144667987
Ltf	0.096665941	0.716691103	0.144667987
Ccdc173	-0.160651598	0.716691103	0.144667987
Ooep	0.112445483	0.716691103	0.144667987
Nrcam	-0.114186409	0.716691103	0.144667987
Rnf31	0.099734221	0.716691103	0.144667987
CCDC151	-0.158337934	0.716691103	0.144667987
Trim32	-0.175841243	0.716691103	0.144667987
Rps13	0.093989373	0.716691103	0.144667987
Sema3a	0.125305441	0.716691103	0.144667987
Tceal1	-0.103042085	0.716691103	0.144667987
Qsox1	-0.081824628	0.716691103	0.144667987

Prr15	0.164188311	0.716691103	0.144667987
Pmepa1	-0.13349106	0.716814253	0.144593368
Tmem151	0.103949142	0.716928651	0.144524063
Usp43	-0.10226135	0.716962402	0.144503618
Inmt	0.13971546	0.716962402	0.144503618
Ppm1e	0.071096418	0.716962402	0.144503618
Ccnd2	-0.11921107	0.716962402	0.144503618
Rabgef1	-0.175384886	0.71711726	0.144409825
Pcdhb6	-0.2116148	0.717157801	0.144385273
Hecw2	-0.156761847	0.717157801	0.144385273
Celsr3	0.2066691	0.717157801	0.144385273
Necap1	0.099340728	0.717157801	0.144385273
Unc13b	0.248653076	0.717157801	0.144385273
Mtbp	0.11994405	0.717157801	0.144385273
Mafk	-0.138141144	0.717157801	0.144385273
Hadhb	-0.174865536	0.717157801	0.144385273
Twist2	-0.146088604	0.717157801	0.144385273
Nudt4	-0.145306641	0.717157801	0.144385273
Uap1	-0.077698138	0.717157801	0.144385273
Vwa7	-0.110396775	0.717157801	0.144385273
Psmg4	-0.129082794	0.717157801	0.144385273
Zfp51	-0.131128176	0.717157801	0.144385273
Map6	0.236153036	0.717157801	0.144385273
Armcx4	0.106555338	0.717157801	0.144385273
Lamtor2	0.083558201	0.717157801	0.144385273
Zfp853	-0.065724188	0.717157801	0.144385273
Dnhd1	-0.088668611	0.717157801	0.144385273
Ppm1k	0.079503505	0.717157801	0.144385273
Lmo1	-0.062406944	0.717157801	0.144385273
Slc27a4	0.192549618	0.717157801	0.144385273
Erlin1	-0.075863352	0.717157801	0.144385273
Sh2d4b	-0.104187081	0.717157801	0.144385273
Isca1	0.062036683	0.717157801	0.144385273
Lhx5	-0.105323028	0.717157801	0.144385273
Gria2	-0.090875787	0.717157801	0.144385273
Sypl2	0.165433189	0.717157801	0.144385273
Slc9a6	0.146177161	0.717157801	0.144385273
Prr22	0.13184952	0.717157801	0.144385273
Eif4g3	0.102937925	0.717157801	0.144385273
Xbp1	0.202256343	0.718206954	0.143750394
Chrm1	0.401126378	0.718430413	0.143615291
Plcb1	-0.280305771	0.718430413	0.143615291
Atf6	-0.385445915	0.718430413	0.143615291
Ogfod1	-0.22643818	0.718430413	0.143615291
Gfpt2	0.116178221	0.718430413	0.143615291
Zfp74	-0.181738858	0.718430413	0.143615291
Apc	-0.129035304	0.718430413	0.143615291
Apeh	-0.145427732	0.718430413	0.143615291

Pcsk6	-0.154463755	0.718430413	0.143615291
Rgs8	-0.243254223	0.718430413	0.143615291
Kat5	-0.286415689	0.718430413	0.143615291
Zbed3	-0.210170135	0.718430413	0.143615291
RGAG4	-0.180165132	0.718430413	0.143615291
Hmbs	0.20714234	0.718430413	0.143615291
Hoxb4	-0.103553658	0.718430413	0.143615291
Scml2	-0.173724414	0.718430413	0.143615291
Nckap1	-0.100650809	0.718430413	0.143615291
Pinx1	0.095320742	0.718430413	0.143615291
Islr2	-0.076869317	0.718430413	0.143615291
Chml	0.123478176	0.718430413	0.143615291
Tmc7	0.175055853	0.718430413	0.143615291
Nudt14	-0.108217267	0.718430413	0.143615291
Arsg	-0.098532758	0.718430413	0.143615291
Acox3	0.117749525	0.718430413	0.143615291
Zfp41	0.185000367	0.718430413	0.143615291
Far1	0.140060454	0.718430413	0.143615291
APITD1	0.203420068	0.718430413	0.143615291
Ptma	0.109962342	0.718430413	0.143615291
Bola2	0.161467518	0.718430413	0.143615291
Alyref	0.187532544	0.718430413	0.143615291
Col2a1	-0.161069493	0.718430413	0.143615291
Actr1b	0.099522036	0.718430413	0.143615291
Zfp455	0.46960584	0.718430413	0.143615291
Ins2	-0.104853014	0.718430413	0.143615291
Kcnh4	0.090369228	0.718430413	0.143615291
Mkln1os	-0.10446445	0.718430413	0.143615291
Zyg11b	0.306443231	0.718430413	0.143615291
Scand1	0.091702201	0.718430413	0.143615291
Zkscan4	0.167758771	0.718430413	0.143615291
Prss45	-0.120129721	0.718430413	0.143615291
Tmem205	0.062709105	0.718430413	0.143615291
Ica1l	-0.107784575	0.718430413	0.143615291
Pcdhb3	-0.090045641	0.718430413	0.143615291
Ager	0.185633565	0.718430413	0.143615291
Epm2a	0.075637569	0.718430413	0.143615291
Sgk3	-0.079657582	0.718430413	0.143615291
Mpdz	0.095852215	0.718430413	0.143615291
Ldhd	0.071505345	0.718430413	0.143615291
Gad1os	0.10892207	0.718430413	0.143615291
Armcx1	-0.173502492	0.718430413	0.143615291
Cpa4	0.117693516	0.718430413	0.143615291
Metap1d	0.097228249	0.718430413	0.143615291
St3gal5	-0.144693933	0.718430413	0.143615291
Tnfsf18	0.133591813	0.718430413	0.143615291
Zbtb39	0.114064499	0.718430413	0.143615291
Sft2d1	0.07643329	0.718430413	0.143615291

Gabra5	-0.08995553	0.718430413	0.143615291
Abcg4	0.139432189	0.718430413	0.143615291
Acp7	-0.082724907	0.718430413	0.143615291
Raver2	0.210964916	0.718430413	0.143615291
Rabl2	0.254904537	0.718430413	0.143615291
Adm2	-0.12943506	0.718430413	0.143615291
Anln	0.089895132	0.718430413	0.143615291
Camkmt	0.099150252	0.718430413	0.143615291
Asphd1	0.175718474	0.718430413	0.143615291
Adam5	0.117953391	0.718430413	0.143615291
Ywhah	0.084863493	0.718430413	0.143615291
S1pr5	-0.084393945	0.718430413	0.143615291
Adat1	-0.106016725	0.718430413	0.143615291
Man1a	-0.132389926	0.718430413	0.143615291
Map1b	0.11331167	0.718430413	0.143615291
Nrip2	-0.061116069	0.718430413	0.143615291
Apol8	0.077787222	0.718430413	0.143615291
Zic1	-0.104862542	0.718430413	0.143615291
Ptpn13	0.114136343	0.718430413	0.143615291
Ncmap	-0.099672595	0.718430413	0.143615291
Tecpr2	-0.089176716	0.718430413	0.143615291
Tagap1	0.075992714	0.718430413	0.143615291
Ngrn	0.074710054	0.718430413	0.143615291
Zbtb7a	0.094283481	0.718430413	0.143615291
Ndel1	0.101312999	0.718430413	0.143615291
Mblac1	-0.084938637	0.718430413	0.143615291
Spaca9	-0.073020196	0.718430413	0.143615291
HIST1H4A	-0.14940706	0.718430413	0.143615291
Capn6	0.160921465	0.718430413	0.143615291
Lef1	0.136932954	0.718430413	0.143615291
Med12l	0.115068736	0.718430413	0.143615291
Tenm4	0.114848496	0.718430413	0.143615291
Magi3	-0.174892535	0.718430413	0.143615291
Rnf165	-0.096736873	0.718430413	0.143615291
Strn4	0.118586267	0.718430413	0.143615291
Lrrtm2	0.100333802	0.718430413	0.143615291
CCDC53	0.07710672	0.718430413	0.143615291
Dennd6b	0.090211941	0.718430413	0.143615291
Dapk1	0.069342153	0.718430413	0.143615291
Olfr550	0.097025333	0.718430413	0.143615291
Gpr33	0.094008306	0.718430413	0.143615291
Ncapd2	-0.060436895	0.718430413	0.143615291
Fyn	-0.091815502	0.718430413	0.143615291
Notch4	-0.116088018	0.718430413	0.143615291
Gnaz	0.159483434	0.718430413	0.143615291
Pxdn	0.135107021	0.718430413	0.143615291
Slc12a2	0.080985793	0.718430413	0.143615291
Ppargc1a	-0.125839881	0.718430413	0.143615291

Ntf3	0.072513276	0.718430413	0.143615291
Nrxn1	0.181657378	0.718430413	0.143615291
Arpp19	-0.124258702	0.718430413	0.143615291
Tcaf1	0.111131104	0.718430413	0.143615291
Slitrk5	0.079295207	0.718430413	0.143615291
Clk1	-0.107702254	0.718430413	0.143615291
Sox2	0.209494524	0.718430413	0.143615291
Nt5dc3	-0.109755294	0.718430413	0.143615291
Rnf150	0.07535745	0.719016065	0.143261406
Slc15a4	0.121452445	0.719364878	0.14305077
Nt5c3	0.096008084	0.719500011	0.142969195
Thsd4	-0.254321596	0.719560099	0.142932927
Cyp2j6	-0.191322505	0.719560099	0.142932927
Scnn1a	0.671590206	0.719560099	0.142932927
S100a3	0.727917286	0.719560099	0.142932927
B3gat1	0.077712599	0.719560099	0.142932927
Igf2	-0.131349427	0.719560099	0.142932927
Cntn3	0.083497455	0.719560099	0.142932927
Mknk2	0.090330518	0.719560099	0.142932927
Pafah1b1	0.142492195	0.720389382	0.142432697
Ing1	0.088874529	0.720389382	0.142432697
Lrrtm4	-0.08834197	0.720389382	0.142432697
Slc7a3	-0.120076022	0.720389382	0.142432697
Dnajb5	0.223414891	0.720389382	0.142432697
Cmtm4	-0.141165595	0.720389382	0.142432697
Naaa	-0.121659852	0.720389382	0.142432697
Eid3	0.096992529	0.720389382	0.142432697
Paqr9	0.147962933	0.720389382	0.142432697
Sorcs1	0.080843128	0.720389382	0.142432697
Cd5	0.077529166	0.720389382	0.142432697
Cd7	0.161145123	0.720389382	0.142432697
Htr1d	0.1058318	0.720389382	0.142432697
Cd164	0.111768477	0.720389382	0.142432697
Appl1	0.241310502	0.720389382	0.142432697
Asrgl1	-0.100789559	0.720389382	0.142432697
Trafd1	-0.071731399	0.720389382	0.142432697
Cemip	0.256758324	0.72052255	0.142352422
Tmem158	0.16983398	0.722211285	0.14133573
Ccr10	-0.118483605	0.722248378	0.141313425
Nxpe4	-0.087740149	0.7222976	0.141283828
Fam117a	-0.098548044	0.722382086	0.141233032
Slc3a1	0.135317389	0.722399107	0.1412228
Layn	-0.233921923	0.722508753	0.141156887
Tmtc1	0.101144844	0.722508753	0.141156887
Rapgef3	0.203496647	0.722508753	0.141156887
Slc4a11	0.088826164	0.722508753	0.141156887
Zbbx	0.208021344	0.723401779	0.140620427
Dzip3	-0.160434576	0.723645685	0.140474023

Parva	-0.075497889	0.723645685	0.140474023
Endov	-0.179661967	0.723645685	0.140474023
Nod2	0.205653521	0.723645685	0.140474023
Nup35	0.096314835	0.723645685	0.140474023
Dip2c	0.155577546	0.724370679	0.140039137
Syngap1	-0.153348388	0.724370679	0.140039137
Ccdc32	0.151745973	0.724534787	0.139940758
Vcpkmt	0.245883046	0.724985497	0.139670681
Taf1a	-0.148813893	0.725099265	0.139602535
Pcdha1	0.205257899	0.725099265	0.139602535
Elac1	0.102916759	0.725099265	0.139602535
Hs3st3a1	0.094494383	0.725266799	0.139502203
Shcbp1l	0.13076786	0.725411131	0.139415785
Mup5	0.17281462	0.725720909	0.139230364
Sfn	0.110330255	0.725720909	0.139230364
Atp1b1	0.13177524	0.725720909	0.139230364
Nit2	-0.17456339	0.725720909	0.139230364
Zfp521	0.09883514	0.725860789	0.139146664
Pde6g	0.080284429	0.725860789	0.139146664
Podnl1	-0.223142402	0.725922181	0.139109933
PABPC1L2	-0.083011266	0.726097191	0.139005243
Pfkm	-0.12092948	0.726097191	0.139005243
Csnk2a2	-0.067928464	0.726097191	0.139005243
Gabarapl1	0.081445757	0.726097191	0.139005243
Spryd4	-0.070736086	0.726097191	0.139005243
Smg1	0.210370041	0.726590752	0.138710134
Smpd4	0.092848498	0.727083614	0.138415643
Coil	-0.231675605	0.727648044	0.138078634
Usp34	-0.1889586	0.727648044	0.138078634
Snhg8	-0.191788897	0.727648044	0.138078634
Zfp827	0.093586581	0.727648044	0.138078634
Dffa	-0.162657494	0.727648044	0.138078634
Fbxl18	0.443884919	0.727648044	0.138078634
Eaf1	-0.132457485	0.727648044	0.138078634
Itgb4	0.099002625	0.727648044	0.138078634
Mgrn1	-0.329718352	0.727648044	0.138078634
C2cd4c	0.268331071	0.727648044	0.138078634
Snhg11	0.09559834	0.727648044	0.138078634
Phactr4	-0.113234485	0.727648044	0.138078634
Mybph	0.123214654	0.727648044	0.138078634
AI606473	0.106960932	0.727648044	0.138078634
Pak6	0.096267948	0.727648044	0.138078634
Cdk5r1	0.115400426	0.727648044	0.138078634
Hes7	-0.118506164	0.727648044	0.138078634
Lonrf2	0.266666609	0.727648044	0.138078634
Wrap73	0.157034042	0.727648044	0.138078634
Psen2	0.136890664	0.727648044	0.138078634
Pdzd9	-0.081746527	0.727648044	0.138078634

Trpv3	-0.08832472	0.727648044	0.138078634
FAM198A	-0.071736207	0.727648044	0.138078634
Rbfox1	-0.123481089	0.727648044	0.138078634
Map3k10	-0.201299297	0.727648044	0.138078634
Cntn6	0.073530115	0.727648044	0.138078634
FAM160A2	0.279148447	0.727648044	0.138078634
Phlpp2	-0.070007564	0.727648044	0.138078634
Kctd1	-0.172003767	0.727648044	0.138078634
Kndc1	0.152184306	0.727648044	0.138078634
Msi1	-0.06723429	0.727648044	0.138078634
Zc3h13	-0.116373439	0.727648044	0.138078634
Rpl10	0.435042367	0.727648044	0.138078634
Thegl	0.079192139	0.727648044	0.138078634
Gt(ROSA)2	0.077136877	0.727648044	0.138078634
DYX1C1	-0.185518372	0.727648044	0.138078634
Zfp808	0.100775795	0.727648044	0.138078634
Pim3	0.113732715	0.727648044	0.138078634
Agr2	0.10380126	0.727648044	0.138078634
Vil1	0.11181505	0.727648044	0.138078634
AI839979	0.084876041	0.727648044	0.138078634
Olfr1344	0.144948711	0.727648044	0.138078634
Epc2	0.148981814	0.727648044	0.138078634
Socs5	0.074986064	0.727648044	0.138078634
Spg21	-0.081918503	0.727648044	0.138078634
Ube2v2	-0.080277687	0.727648044	0.138078634
Pigv	0.242174803	0.727648044	0.138078634
Card6	-0.179678451	0.727648044	0.138078634
Cdk7	0.221385348	0.727648044	0.138078634
Sypl	-0.10405306	0.727648044	0.138078634
Grm7	-0.119935537	0.727648044	0.138078634
Ace2	0.089517157	0.727648044	0.138078634
Dlk1	-0.106864002	0.727648044	0.138078634
Mettl11b	-0.261573695	0.728086736	0.137816881
Tsga13	0.088988513	0.728086736	0.137816881
Gatm	-0.078227623	0.72811626	0.13779927
Llgl1	-0.098878097	0.72811626	0.13779927
Prex2	-0.308568666	0.72904261	0.137247088
Tmc1	-0.10434282	0.729414675	0.137025503
Htra1	-0.081378721	0.729507399	0.136970299
Disp2	0.155947666	0.729654486	0.136882743
Xlr3b	-0.102300888	0.729654486	0.136882743
Zbtb4	-0.148772548	0.729654486	0.136882743
Bbs10	-0.085120342	0.729756404	0.136822085
Adgrb2	0.085770286	0.730156525	0.13658403
Mfap5	-0.129147799	0.730758643	0.136226039
Lhfpl4	0.11674817	0.730758643	0.136226039
Sorbs2	-0.099078468	0.730758643	0.136226039
Gga3	0.201406807	0.730758643	0.136226039

Traip	0.101906592	0.730758643	0.136226039
Ergic1	0.085661346	0.731115811	0.136013824
Cys1	0.092661435	0.731115811	0.136013824
FAM196A	-0.150592861	0.731193141	0.135967891
Lurap1	-0.1234697	0.731193141	0.135967891
Rpgrip1l	0.247475602	0.731414016	0.135836722
Dsc2	0.0896235	0.731414016	0.135836722
Mzb1	0.119267203	0.731454057	0.135812947
Bbs4	-0.113077045	0.731454057	0.135812947
Mall	0.080639558	0.731454057	0.135812947
Zfp960	-0.096116642	0.731454057	0.135812947
C1ql2	-0.161004739	0.731454057	0.135812947
Pdzd7	-0.186793771	0.731679683	0.135679004
Vmn2r46	0.125158003	0.731890951	0.135553622
Dnph1	-0.218309123	0.732033872	0.135468823
Bcas1os2	-0.213436301	0.732033872	0.135468823
Phyhip	-0.474941043	0.732033872	0.135468823
Snap47	-0.101009663	0.732033872	0.135468823
Pja2	0.166435151	0.732033872	0.135468823
Ube2o	-0.155926348	0.732033872	0.135468823
Rab3d	0.117659548	0.732033872	0.135468823
Best3	0.292506372	0.732033872	0.135468823
Gja3	-0.087769053	0.732033872	0.135468823
Grem1	0.15089387	0.732033872	0.135468823
Gimap1	-0.119048992	0.732033872	0.135468823
Nqo2	-0.091746892	0.732033872	0.135468823
Lrrc56	-0.144979155	0.732033872	0.135468823
Usp27x	-0.084451771	0.732033872	0.135468823
Prl2c2	0.091085464	0.732033872	0.135468823
Eps15	0.071642975	0.732033872	0.135468823
Trpm4	0.188166962	0.732033872	0.135468823
Arhgap44	0.177038796	0.732033872	0.135468823
Zdhhc23	-0.218904661	0.732033872	0.135468823
Isoc2a	-0.244883323	0.732033872	0.135468823
Spata45	0.566089902	0.732033872	0.135468823
Zfp874a	-0.116229754	0.732033872	0.135468823
Ntn1	-0.094089399	0.732033872	0.135468823
Dgke	0.113781569	0.732033872	0.135468823
Ppp1r12b	0.106002512	0.732033872	0.135468823
Khk	0.094591591	0.732033872	0.135468823
Ptprm	-0.187856835	0.732033872	0.135468823
Qpct	0.138219526	0.732033872	0.135468823
Krt90	0.091578705	0.732033872	0.135468823
Zfp148	-0.106540311	0.732033872	0.135468823
Otp	0.211073715	0.732033872	0.135468823
Il12b	0.077548118	0.732033872	0.135468823
FAM134A	-0.28996657	0.732659046	0.135098084
Ranbp17	0.100010046	0.732659046	0.135098084

Nhlrc1	-0.20832525	0.732881967	0.134965964
Akt3	-0.140305059	0.732881967	0.134965964
Spin1	0.071354612	0.732881967	0.134965964
Plpp6	0.110995518	0.732881967	0.134965964
Ttr	-0.146986748	0.732984436	0.134905247
Glmn	-0.169629432	0.733081246	0.134847891
Bap1	0.076352707	0.733091147	0.134842025
Efcab5	0.11684618	0.733673561	0.134497131
Lrrc69	-0.078552821	0.733683927	0.134490995
Plet1os	0.181163561	0.733846239	0.134394927
Ildr1	-0.212192561	0.733846239	0.134394927
Zfp970	-0.080574471	0.733846239	0.134394927
Zfp280d	-0.28792724	0.733925387	0.13434809
Eomes	-0.216216355	0.733925387	0.13434809
Stox1	0.111552912	0.733925387	0.13434809
Mapk4	-0.148863003	0.733925387	0.13434809
Nptx1	-0.149690779	0.733925387	0.13434809
Gid4	0.106095075	0.733925387	0.13434809
Chst10	0.148499831	0.733925387	0.13434809
FAM179A	0.090099918	0.733925387	0.13434809
Ahi1	-0.092570809	0.733925387	0.13434809
Dph3	0.126458027	0.733925387	0.13434809
Btbd6	0.094257382	0.733925387	0.13434809
Dact3	0.321210704	0.733925387	0.13434809
Ppa2	0.110193331	0.733925387	0.13434809
Nudt6	0.114338209	0.733925387	0.13434809
St6galnac!	-0.106585551	0.733925387	0.13434809
Rem2	0.115235439	0.733925387	0.13434809
Nanp	0.08026191	0.733925387	0.13434809
Dcx	-0.117099998	0.733925387	0.13434809
Txnl4b	-0.153834019	0.733925387	0.13434809
Fbxo45	-0.109214393	0.733925387	0.13434809
Ldlrad4	-0.080530258	0.733925387	0.13434809
Map3k3	-0.09436365	0.733925387	0.13434809
Zc3h12b	0.102443342	0.733925387	0.13434809
Clmn	0.092987864	0.733925387	0.13434809
Areg	-0.24572026	0.734068665	0.134263314
Mbp	0.126673998	0.734092494	0.134249217
Lin9	-0.161199472	0.734396628	0.134069326
Ypel3	0.182164078	0.734396628	0.134069326
Ly6d	-0.2581078	0.734396628	0.134069326
TCTEX1D1	-0.095105017	0.734396628	0.134069326
Cntnap5a	-0.182286636	0.734396628	0.134069326
Dusp5	-0.375759454	0.734396628	0.134069326
Prr29	0.094185486	0.734396628	0.134069326
Adar	0.127719791	0.734396628	0.134069326
AI115009	0.072831581	0.734396628	0.134069326
Glis1	-0.074034672	0.734396628	0.134069326

Rtf1	0.255779574	0.734396628	0.134069326
Drosha	-0.090824731	0.734396628	0.134069326
Carns1	-0.159026346	0.734396628	0.134069326
Ermn	-0.151636841	0.734396628	0.134069326
Kcnip1	-0.079816043	0.734396628	0.134069326
Bcs1l	0.086327315	0.734396628	0.134069326
Serpinb9	0.100451913	0.734396628	0.134069326
Ets1	-0.288060856	0.734396628	0.134069326
Gal3st3	-0.098121783	0.734396628	0.134069326
Tob2	0.209675277	0.734396628	0.134069326
Aph1c	0.110057402	0.734396628	0.134069326
Efhd2	-0.104521857	0.734396628	0.134069326
B4galt2	-0.110229251	0.734396628	0.134069326
AW20949:	-0.171108121	0.734396628	0.134069326
Amotl1	0.111934004	0.734396628	0.134069326
Lrrc1	-0.094372907	0.734396628	0.134069326
Arhgef10	0.16460624	0.734396628	0.134069326
Lhfpl3	0.135742687	0.734396628	0.134069326
Kl	0.109648831	0.734396628	0.134069326
Sash1	0.104095461	0.734396628	0.134069326
Adamts17	-0.057289754	0.734396628	0.134069326
Ntf5	0.233738493	0.734396628	0.134069326
Hsp90aa1	-0.294455903	0.734396628	0.134069326
Gadl1	-0.143468731	0.734396628	0.134069326
Oprm1	0.111140144	0.734396628	0.134069326
MKL2	0.085946158	0.734396628	0.134069326
Slc10a4	0.106722273	0.734396628	0.134069326
Tnfsf13	-0.102616948	0.734396628	0.134069326
Enox1	0.118331849	0.734396628	0.134069326
Fam183b	-0.108396233	0.734396628	0.134069326
Rhot1	0.071467268	0.734396628	0.134069326
Cd70	-0.108086677	0.734396628	0.134069326
Ptprj	-0.187696608	0.734826974	0.13381491
Acaa2	0.089708559	0.734826974	0.13381491
Platr14	0.117133528	0.734826974	0.13381491
Asns	0.143732414	0.734826974	0.13381491
Fto	0.636791006	0.734938201	0.133749178
Tmem258	0.096705589	0.734970806	0.133729911
Upk1a	0.158778298	0.735022989	0.133699078
Dab1	-0.159977146	0.735022989	0.133699078
Tollip	0.091378307	0.735022989	0.133699078
Setd7	-0.307192335	0.735070056	0.133671268
Nmnat3	0.089087717	0.735070056	0.133671268
Ythdc2	-0.081709899	0.735070056	0.133671268
Derl3	0.121704197	0.735070056	0.133671268
Nxpe2	0.202453313	0.735070056	0.133671268
Gab1	-0.116147498	0.735070056	0.133671268
Fam50a	0.095916542	0.735070056	0.133671268

Hectd2	-0.167644549	0.735070056	0.133671268
Ankrd42	0.09345005	0.735070056	0.133671268
Tyrp1	-0.269832938	0.735070056	0.133671268
Alox8	-0.150045758	0.735355005	0.133502948
Katna1	0.10145342	0.735355005	0.133502948
Zfp382	0.066833124	0.735632071	0.133339346
Agtr2	0.105207661	0.735684641	0.133308311
Pura	-0.179756654	0.735707681	0.13329471
Crbn	-0.168919649	0.735707681	0.13329471
Mpdu1	-0.476968912	0.735707681	0.13329471
Lrrc3	-0.132092965	0.735707681	0.13329471
Mroh1	-0.186668417	0.735707681	0.13329471
Ggn	-0.208672818	0.735707681	0.13329471
Arhgap22	0.104008232	0.735707681	0.13329471
Patl1	-0.364078671	0.735707681	0.13329471
Pmvk	-0.181949439	0.735707681	0.13329471
Dusp1	0.135227298	0.735707681	0.13329471
Rps20	0.115796958	0.735707681	0.13329471
Stoml1	0.178801877	0.735707681	0.13329471
Pnmt	0.10229554	0.735707681	0.13329471
Rnf32	-0.112485605	0.735707681	0.13329471
Pxmp4	0.081785757	0.735707681	0.13329471
Adam22	0.216117395	0.735707681	0.13329471
Gdap1	-0.186473072	0.735707681	0.13329471
Syt8	-0.082232093	0.735707681	0.13329471
Usp53	-0.078006767	0.735707681	0.13329471
Adat3	0.056017997	0.735707681	0.13329471
Ubxn2b	-0.07469975	0.735707681	0.13329471
PLATR17	0.136842433	0.735707681	0.13329471
Ptprn	-0.073448723	0.735707681	0.13329471
Arhgef18	-0.065599439	0.735707681	0.13329471
Slc4a8	0.062030368	0.735707681	0.13329471
Arhgap35	0.081954955	0.735707681	0.13329471
Atp1a3	0.124082518	0.735707681	0.13329471
Ranbp6	-0.075612734	0.735707681	0.13329471
Etfdh	-0.182766092	0.735707681	0.13329471
Sel1l3	0.109827626	0.735707681	0.13329471
Cyp26a1	0.168510004	0.735707681	0.13329471
Epg5	-0.091573683	0.735707681	0.13329471
Usp14	0.065814564	0.735707681	0.13329471
Cpd	-0.127970878	0.735971571	0.133138961
Slc23a2	0.081968521	0.735971571	0.133138961
Kmt5a	0.135275447	0.735971571	0.133138961
Isg20l2	0.074608978	0.735971571	0.133138961
Mettl24	0.212444097	0.736427993	0.132869711
Cstf2	0.081185147	0.736723808	0.132695295
Mfn2	-0.10783402	0.736723808	0.132695295
Pde6h	0.184157573	0.737390583	0.132302413

Fgf21	0.116792552	0.738257652	0.131792043
Tuba4a	0.185090734	0.7389887	0.131362202
Smyd3	-0.15556719	0.7389887	0.131362202
Nupl1	0.165038157	0.7389887	0.131362202
Nckap5l	0.194173038	0.7389887	0.131362202
Fdps	0.09143843	0.7389887	0.131362202
Rbbp8	-0.119297908	0.7389887	0.131362202
Ptch1	0.184062593	0.739210432	0.131231913
Klhl30	0.119362132	0.739210432	0.131231913
Glt28d2	0.152759902	0.739210432	0.131231913
Adra1a	-0.211508582	0.739210432	0.131231913
DDX39	0.447137667	0.739210432	0.131231913
Snhg18	-0.064740023	0.739210432	0.131231913
Fbxo15	0.174442304	0.739210432	0.131231913
Slc9a7	-0.107962178	0.739210432	0.131231913
Grin2d	0.088665908	0.739210432	0.131231913
Hcar1	-0.191330861	0.739563874	0.131024311
Chchd6	-0.087144015	0.739563874	0.131024311
Zfp513	0.203253981	0.739563874	0.131024311
Wdr73	0.091159892	0.739563874	0.131024311
Tmem218	0.071441377	0.739563874	0.131024311
Dynll2	-0.12624121	0.739563874	0.131024311
Csmd1	0.086628051	0.739563874	0.131024311
AI661453	-0.112865888	0.739563874	0.131024311
Slc25a18	-0.085840214	0.739563874	0.131024311
H2-M10.2	-0.06072497	0.739768048	0.13090443
Trak1	-0.114554371	0.74021408	0.130642658
Lrrc39	-0.127505385	0.74021408	0.130642658
Thpo	0.06850612	0.74021408	0.130642658
Ahcy	0.107099197	0.74021408	0.130642658
Dazap2	0.212386973	0.74021408	0.130642658
DOPEY1	0.08515558	0.74021408	0.130642658
Fpr1	0.092047838	0.740300614	0.130591891
HIST2H2BE	0.123877364	0.740300614	0.130591891
Gas6	0.195273206	0.740300614	0.130591891
Cacna1c	-0.100624397	0.740437253	0.130511739
Plk3	-0.176550348	0.740519828	0.130463308
Slc22a21	-0.100309687	0.740704475	0.130355031
Zfp946	0.091469078	0.740704475	0.130355031
Usb1	-0.073926121	0.741244767	0.130038359
Shc4	0.232143145	0.741244767	0.130038359
Smkr-ps	0.081043233	0.74132747	0.129989907
Lman1	-0.074365383	0.74142438	0.129933137
Pcgf1	-0.121053027	0.741458496	0.129913154
Lactb2	0.339619252	0.741458496	0.129913154
Slc25a23	0.078320632	0.741458496	0.129913154
Akain1	0.118385271	0.741660868	0.129794635
Nostrin	0.14588553	0.74177445	0.12972813

Rgs18	-0.067553277	0.74177445	0.12972813
Kmt2a	-0.253580003	0.742368209	0.129380635
Htt	-0.291731988	0.742368209	0.129380635
Id4	0.104443852	0.742368209	0.129380635
Alpl	-0.073687889	0.742368209	0.129380635
Caskin1	-0.116072135	0.742368209	0.129380635
E2f5	0.100550514	0.742368209	0.129380635
Scx	-0.227279797	0.742957439	0.129036064
Galnt14	-0.1146947	0.742957439	0.129036064
Atmin	-0.099123392	0.742957439	0.129036064
Ncoa7	0.156357765	0.742957439	0.129036064
Ddx55	0.098281552	0.742957439	0.129036064
Aard	-0.087730938	0.742957439	0.129036064
Impdh1	-0.093470874	0.742957439	0.129036064
Chst1	-0.112749132	0.742957439	0.129036064
Slc25a47	-0.129429581	0.743163495	0.128915631
Pakap	0.105523465	0.743233806	0.128874545
Mul1	0.102627583	0.74332055	0.12882386
Morc4	0.090878328	0.743877499	0.128498578
Trib3	0.116534602	0.743918445	0.128474673
Zfp385c	-0.168667844	0.744032508	0.128408089
Ogfrl1	0.101178819	0.744032508	0.128408089
Mtcl1	0.104183856	0.744032508	0.128408089
Aff2	0.084519165	0.744084696	0.128377628
Trim44	0.061990773	0.744084696	0.128377628
Afg3l2	0.07744678	0.744084696	0.128377628
Cnbd2	0.300554567	0.744211949	0.128303361
Pls1	0.079844289	0.744211949	0.128303361
Dhdds	-0.21587727	0.744211949	0.128303361
Atpaf1	-0.096467543	0.744211949	0.128303361
Rab19	0.123254433	0.744211949	0.128303361
Atg13	-0.16773947	0.744211949	0.128303361
Itsn1	0.11100095	0.744211949	0.128303361
Bmp1	-0.129593024	0.744211949	0.128303361
Bpifb1	-0.073674339	0.744211949	0.128303361
Lpar2	0.202099146	0.744211949	0.128303361
Lzts3	0.116655185	0.744211949	0.128303361
Ssbp2	-0.106475035	0.744211949	0.128303361
Atp6v1c1	-0.178634226	0.744211949	0.128303361
Snapc1	0.08768112	0.744211949	0.128303361
Zer1	-0.137104505	0.744211949	0.128303361
Mast4	0.081263041	0.744211949	0.128303361
Plpp4	0.098758591	0.744211949	0.128303361
Pnpla8	0.065898458	0.744211949	0.128303361
Etv6	-0.104511824	0.744211949	0.128303361
Ccdc28a	-0.076390748	0.744211949	0.128303361
Serp2	0.113778752	0.744211949	0.128303361
F2r	-0.197886538	0.744490223	0.128141001

Mas1	-0.165006	0.744490223	0.128141001
Vps13c	0.080349833	0.744515063	0.128126511
ADRBK2	0.150378727	0.744515063	0.128126511
Gpr31b	0.078048174	0.744535839	0.128114392
Slc2a1	0.39372555	0.744692477	0.128023033
Slc1a4	-0.071767736	0.744692477	0.128023033
Tgfb1i1	-0.199282194	0.745055962	0.127811106
Tmem29	-0.089309279	0.745055962	0.127811106
Myct1	-0.471244169	0.745843458	0.127352315
Flot2	-0.237256939	0.745843458	0.127352315
Tceal5	0.181005545	0.745843458	0.127352315
Grik1	-0.168270671	0.745843458	0.127352315
Lypd2	0.185270889	0.745843458	0.127352315
Zc2hc1a	0.281022889	0.745843458	0.127352315
BC067074	0.321121682	0.745843458	0.127352315
Mup19	0.099906487	0.745843458	0.127352315
Arhgap21	0.104316498	0.745843458	0.127352315
Plscr3	-0.120118781	0.745843458	0.127352315
Plekhg5	0.14545237	0.745843458	0.127352315
Vash1	-0.127637525	0.745843458	0.127352315
Zscan26	0.106394338	0.745843458	0.127352315
Ildr2	0.117804147	0.745843458	0.127352315
Thy1	-0.194390891	0.745843458	0.127352315
Catsper3	0.235976745	0.745843458	0.127352315
Krt15	-0.239231916	0.745843458	0.127352315
Etfb	0.124189338	0.745843458	0.127352315
Lrrc49	0.072273687	0.745843458	0.127352315
S100a14	-0.109238444	0.745843458	0.127352315
Bnc2	0.081336991	0.745843458	0.127352315
Lurap1l	0.229575185	0.745843458	0.127352315
Mob2	0.06558649	0.745843458	0.127352315
Alkbh8	0.173231045	0.745843458	0.127352315
Fgf9	0.143585949	0.745843458	0.127352315
Gpr171	0.086212849	0.745843458	0.127352315
Proz	-0.070580717	0.745843458	0.127352315
FAM69B	-0.052588766	0.745843458	0.127352315
Vrk1	-0.068390396	0.745843458	0.127352315
Gabpa	0.112441466	0.745843458	0.127352315
Slc1a1	-0.13187979	0.745968301	0.127279627
Ttc33	-0.253720123	0.745990375	0.127266776
Rps4x	0.196368472	0.745990375	0.127266776
Olfr463	-0.06892732	0.745990375	0.127266776
Cyp39a1	-0.056751383	0.745990375	0.127266776
Banp	0.097103803	0.746181772	0.127155364
Zcchc18	-0.103348487	0.746425469	0.12701355
Rragb	-0.223542113	0.746660533	0.126876804
Emcn	-0.183475955	0.74696238	0.12670127
Acvr1c	0.121588093	0.74696238	0.12670127

Slc16a2	0.084208986	0.74696238	0.12670127
Gnaq	0.133289581	0.74696238	0.12670127
Mnat1	0.164694645	0.74696238	0.12670127
Chmp1b	0.300656726	0.74696238	0.12670127
Ppp1r32	0.07562836	0.74746057	0.126411712
Sil1	0.11507196	0.747641132	0.126306814
Zfp474	0.094607466	0.747688409	0.126279352
Lrrc18	0.071586921	0.747688409	0.126279352
Ppp1r36	-0.085222311	0.747688409	0.126279352
Msc	-0.065927283	0.747688409	0.126279352
Pdzph1	-0.100137615	0.747688409	0.126279352
Slc18a1	-0.192359249	0.747824876	0.126200092
AI225912	-0.092517995	0.748154667	0.12600861
Vrk3	0.116688913	0.748154667	0.12600861
Lrrtm3	-0.102152347	0.748154667	0.12600861
Sap25	0.106799976	0.748154667	0.12600861
CXX1B	-0.088352807	0.748411054	0.125859806
Zfp568	0.064907348	0.748411054	0.125859806
Sec14l5	-0.061418387	0.74865491	0.125718323
Galk2	0.103504113	0.748839716	0.12561113
Rbm20	0.0840818	0.748868382	0.125594505
Madcam1	0.128262266	0.749114307	0.125451909
Cxxc4	-0.130175829	0.749114307	0.125451909
Tfap2d	0.093657819	0.749735059	0.12509218
Ncoa2	0.087495719	0.750029219	0.124921818
Krt20	-0.396497254	0.750258857	0.124788869
Fbxl22	-0.055641933	0.750258857	0.124788869
Mecp2	0.078040033	0.750258857	0.124788869
Lrrc32	-0.12765533	0.750258857	0.124788869
Arrb1	-0.065216377	0.750258857	0.124788869
Sh2d4a	0.134743297	0.750258857	0.124788869
Cdc25b	0.245683353	0.750258857	0.124788869
Radil	0.075208938	0.750258857	0.124788869
Vamp2	0.061392836	0.750258857	0.124788869
Klhl13	-0.127367602	0.75053547	0.124628778
Ctxn1	0.167072212	0.75053547	0.124628778
Tacc2	-0.141644633	0.750737274	0.124512021
Lhcgr	0.077147326	0.750737274	0.124512021
Slc26a10	0.103603162	0.750938699	0.124395514
Rpl7	-0.264966317	0.750938699	0.124395514
HIST1H3F	0.081180055	0.750938699	0.124395514
Fam219a	-0.064096334	0.750938699	0.124395514
Alx3	-0.078227676	0.750938699	0.124395514
MFSD7B	0.084324722	0.750938699	0.124395514
Eif4enif1	0.073728526	0.751003067	0.12435829
Plekha2	0.249513256	0.751022201	0.124347224
Tmem30b	0.120331761	0.751126654	0.124286826
Smg7	0.261310817	0.751424465	0.124114669

Tysnd1	-0.103517741	0.751424465	0.124114669
Mical3	0.073626864	0.752327665	0.123592968
Tmem150	-0.148332856	0.752723719	0.123364399
R3hdm4	0.134398973	0.752934232	0.123242957
Ppcdc	-0.128999266	0.752934232	0.123242957
Cnpy2	0.272581284	0.752934232	0.123242957
Efcab11	-0.076901527	0.752934232	0.123242957
Adarb2	-0.099464645	0.752934232	0.123242957
Cdip1	0.260638964	0.752934232	0.123242957
Prrc2b	0.059141064	0.752934232	0.123242957
Borcs5	0.169487919	0.752934232	0.123242957
Slc25a1	-0.07131149	0.753078649	0.123159665
Hspa2	-0.089302762	0.753078649	0.123159665
Zfp300	-0.090878391	0.753411744	0.122967614
Tdgf1	0.137810775	0.754424297	0.122384333
Ndst3	0.08929189	0.754424297	0.122384333
Tecr	0.131692972	0.754662787	0.122247065
Cab39	-0.178406535	0.754662787	0.122247065
C77370	-0.367073363	0.754826421	0.122152907
Cpne1	-0.24555147	0.754826421	0.122152907
Mcm9	0.119691712	0.754826421	0.122152907
Tmem204	0.090806998	0.754826421	0.122152907
Xpo7	0.087103325	0.754826421	0.122152907
Efna4	-0.106195944	0.754826421	0.122152907
Mib2	0.065273837	0.754826421	0.122152907
Negr1	0.074740567	0.754826421	0.122152907
Esyt3	-0.121852533	0.754826421	0.122152907
Lsm2	-0.082119972	0.754826421	0.122152907
Rock1	-0.061304989	0.755074329	0.122010295
Sugp2	0.110871873	0.755422247	0.121810229
Lyrm7	-0.089430386	0.755422247	0.121810229
Fhl2	-0.103697141	0.755647613	0.121680685
Macrod2	0.205682838	0.755647613	0.121680685
Rtn2	0.196359094	0.755647613	0.121680685
Fam13b	-0.101915626	0.755647613	0.121680685
Tmsb10	0.149466042	0.755689301	0.121656727
Ndrg4	0.090984922	0.75576967	0.121610541
Wdr13	0.080025092	0.756032181	0.121459718
Frmd6	-0.081424726	0.756032181	0.121459718
Fbxo33	0.077376191	0.756032181	0.121459718
Braf	-0.078659568	0.756049822	0.121449584
Asmt	-0.107938193	0.756049822	0.121449584
Zfp939	0.102562329	0.756250879	0.121334108
Ptchd1	0.09108178	0.756250879	0.121334108
Gstt2	-0.101520118	0.756250879	0.121334108
Hnrnpul2	-0.117977776	0.756250879	0.121334108
Icmt	0.096930272	0.756376789	0.121261807
Espnl	-0.099592832	0.756376789	0.121261807

Gramd1c	0.130791174	0.756376789	0.121261807
Saa1	0.109095515	0.756376789	0.121261807
Psap	-0.133054944	0.756376789	0.121261807
Rfc4	0.209986392	0.756376789	0.121261807
10-Mar	0.075761781	0.756376789	0.121261807
Fam170b	0.070721203	0.756376789	0.121261807
Rps10	0.141245775	0.756376789	0.121261807
Rtn4rl2	0.078038054	0.756376789	0.121261807
Lancl2	-0.089144176	0.756376789	0.121261807
Fancf	-0.096737787	0.756376789	0.121261807
Tssk3	-0.080679127	0.756376789	0.121261807
Ppp3cb	0.083486333	0.756376789	0.121261807
St3gal3	-0.093117577	0.756376789	0.121261807
Slc7a5	0.085869238	0.756600682	0.121133271
Slc25a42	0.149788773	0.756778989	0.121030934
Edil3	-0.065751384	0.756809108	0.12101365
Atp6v0a2	0.072581647	0.756809108	0.12101365
Hcn3	-0.103720546	0.756809108	0.12101365
Ccnj	-0.281628285	0.757618755	0.120549283
Edn3	-0.07224795	0.757618755	0.120549283
Sstr1	0.220442311	0.757618755	0.120549283
Usp45	-0.287510535	0.757618755	0.120549283
Pard6a	0.079531964	0.757618755	0.120549283
Cenpv	0.098665135	0.757618755	0.120549283
Pnp2	0.272282006	0.757618755	0.120549283
Spryd3	0.071247404	0.757618755	0.120549283
Ap3b2	0.067942101	0.757618755	0.120549283
Cbs	0.089458326	0.757618755	0.120549283
Adh5	-0.059205399	0.757618755	0.120549283
Abcd4	0.145279879	0.757618755	0.120549283
Olfr691	-0.095603853	0.757618755	0.120549283
Lsr	0.187011798	0.757618755	0.120549283
Dlg3	0.076256147	0.757957443	0.120355178
Hoxd1	0.086995107	0.757966787	0.120349824
Smim10l1	-0.122070151	0.758301051	0.120158342
Nalcn	0.191006801	0.758301051	0.120158342
Epdr1	-0.226959991	0.758509017	0.120039252
Krt16	-0.158070223	0.758509017	0.120039252
Slc38a10	-0.121100913	0.758509017	0.120039252
Pdxp	-0.114451084	0.758509017	0.120039252
Filip1	0.079737801	0.758509017	0.120039252
Pcdhb9	0.090923141	0.758509017	0.120039252
Jmjd4	-0.118575347	0.758509017	0.120039252
Hspa1b	-0.144094295	0.758509017	0.120039252
Mrps15	-0.13411741	0.758509017	0.120039252
Arnt2	0.115718924	0.758509017	0.120039252
Gpr165	0.107405029	0.758509017	0.120039252
Tnpo2	0.07197362	0.758509017	0.120039252

Trmt61b	0.074030202	0.758509017	0.120039252
Dnaja1	-0.078989647	0.758509017	0.120039252
Morn3	0.049110969	0.758509017	0.120039252
Morc3	0.221929476	0.758656989	0.119954537
Fkbp3	0.119151656	0.758656989	0.119954537
METTL21B	-0.108316115	0.758656989	0.119954537
2-Mar	-0.22614739	0.758656989	0.119954537
Sbspon	0.083561649	0.758656989	0.119954537
Nr3c1	0.087724431	0.758656989	0.119954537
Mcoln1	0.098381074	0.758656989	0.119954537
Stk40	0.142032398	0.758656989	0.119954537
Clic4	0.085656677	0.758656989	0.119954537
Cmya5	0.136031138	0.758656989	0.119954537
Reep1	0.087075548	0.758656989	0.119954537
Iqgap2	0.073879151	0.758656989	0.119954537
Pard3b	0.097730208	0.758656989	0.119954537
Tomm40l	0.11694575	0.758656989	0.119954537
Rnf24	-0.443577788	0.758736026	0.119909295
Arfgef2	-0.279775877	0.758736026	0.119909295
Zmat3	-0.178935507	0.758736026	0.119909295
Sel1l	-0.155347973	0.758736026	0.119909295
Vps13b	-0.149101334	0.758736026	0.119909295
Srd5a1	-0.209930297	0.758736026	0.119909295
Parp6	0.150563148	0.758736026	0.119909295
C1ql3	-0.129376937	0.758736026	0.119909295
Psmc5	0.107346145	0.758736026	0.119909295
Metrnl	-0.119880963	0.758736026	0.119909295
Atrn	-0.077778771	0.758736026	0.119909295
Borcs7	0.113374183	0.758736026	0.119909295
Car14	-0.177071513	0.758736026	0.119909295
Fgf14	-0.269130579	0.758736026	0.119909295
Mrln	0.116806649	0.758736026	0.119909295
Krt5	0.11620863	0.758736026	0.119909295
Trmt5	0.146920027	0.758736026	0.119909295
Gstm6	0.157250445	0.758736026	0.119909295
Dusp22	0.107377927	0.758736026	0.119909295
Olfr1507	-0.075174951	0.758736026	0.119909295
Kcnd3	0.110072209	0.758736026	0.119909295
Creld1	0.072334254	0.758736026	0.119909295
Opn1mw	0.105796864	0.758736026	0.119909295
Psph	-0.064406216	0.758736026	0.119909295
Samd15	0.136793935	0.758736026	0.119909295
Gp1bb	0.111782938	0.758736026	0.119909295
Tnrc6b	-0.157367153	0.758736026	0.119909295
Plagl2	0.145048484	0.758736026	0.119909295
Stard9	0.104824066	0.758736026	0.119909295
Haus1	0.098708194	0.758736026	0.119909295
Fuz	0.075798915	0.758736026	0.119909295

Arhgef33	0.222894697	0.758736026	0.119909295
Rasl2-9	-0.242821911	0.758736026	0.119909295
Pcdhgb4	0.167813164	0.758736026	0.119909295
Atp8a2	0.190277305	0.758736026	0.119909295
Ndufa4l2	0.096735412	0.758736026	0.119909295
Vps45	-0.139476426	0.758736026	0.119909295
Dscaml1	0.158137062	0.758736026	0.119909295
Calb1	-0.15577449	0.758736026	0.119909295
Micall1	0.294877558	0.758736026	0.119909295
Kng1	-0.104807374	0.758736026	0.119909295
Ramp2	-0.226692826	0.758736026	0.119909295
B3gat3	-0.235346725	0.758736026	0.119909295
Adam23	0.222121825	0.758736026	0.119909295
Ebp	-0.090008174	0.758736026	0.119909295
Abcg3	0.087978112	0.758736026	0.119909295
Fgf5	-0.133478471	0.758736026	0.119909295
Nt5c2	0.090095121	0.758736026	0.119909295
Adam1b	-0.09224512	0.758736026	0.119909295
Chgb	0.101331292	0.758736026	0.119909295
Dysf	0.077634817	0.758736026	0.119909295
Gstk1	0.093913525	0.758736026	0.119909295
Fbxo4	0.113613696	0.758736026	0.119909295
Lnpep	0.061410218	0.758736026	0.119909295
Tacr2	0.2301522	0.758736026	0.119909295
Nfe2l3	-0.063474043	0.758736026	0.119909295
Tcf3	-0.176572213	0.758736026	0.119909295
Chordc1	0.079349092	0.758736026	0.119909295
S100a7a	0.082027422	0.758736026	0.119909295
Pbx3	0.094133333	0.758736026	0.119909295
Ift172	-0.11445883	0.758736026	0.119909295
Gpn1	0.178074248	0.758736026	0.119909295
Pik3c3	-0.09602357	0.758736026	0.119909295
Polr3a	-0.077886818	0.758736026	0.119909295
Pgbd5	-0.077347378	0.758736026	0.119909295
Pacs2	0.085104079	0.758736026	0.119909295
Tbc1d31	0.069173379	0.758736026	0.119909295
Pfkp	-0.197577059	0.758736026	0.119909295
Clock	0.210558509	0.75915513	0.119669469
Nfatc3	-0.124400773	0.759155675	0.119669157
Rpl41	0.073905151	0.759155675	0.119669157
Mllt6	0.1233325	0.75918234	0.119653903
Rwdd2a	0.165886708	0.75918234	0.119653903
Ttll5	-0.271699926	0.759366286	0.119548688
Scamp5	-0.211214585	0.759366286	0.119548688
Dpysl3	0.079738832	0.759479364	0.119484022
Dcdc2b	-0.11996438	0.759479364	0.119484022
Ngb	-0.104271991	0.759479364	0.119484022
Lcorl	-0.234518675	0.759495213	0.119474959

Rnmt	0.11536973	0.759495213	0.119474959
Lsg1	0.122394976	0.759495213	0.119474959
Nln	-0.150751489	0.759495213	0.119474959
Guca1b	0.113329882	0.759495213	0.119474959
Ociad1	0.139479963	0.759495213	0.119474959
Glcci1	0.087345376	0.759495213	0.119474959
Slc44a5	0.087364629	0.759495213	0.119474959
Arhgap39	0.166782227	0.759495213	0.119474959
Fank1	0.100280173	0.759495213	0.119474959
Copg1	0.216474742	0.759495213	0.119474959
Fgfr1	0.137724611	0.759495213	0.119474959
Pgam5	0.076931309	0.759495213	0.119474959
Akap17b	0.146879779	0.759495213	0.119474959
Rcc1	-0.179109154	0.75980809	0.119296087
Akr1b3	0.091517248	0.75980809	0.119296087
Sec13	-0.083358297	0.75980809	0.119296087
Tmem41b	-0.195330157	0.759955161	0.119212031
Ipo7	-0.179232811	0.760386979	0.118965329
Senp2	-0.135555761	0.76057235	0.118859467
Slit3	0.217196878	0.760815595	0.118720594
Rilpl1	-0.066207789	0.760815595	0.118720594
Tjap1	-0.077504454	0.760815595	0.118720594
Clhc1	0.071374435	0.760815595	0.118720594
Clip2	-0.046461852	0.760815595	0.118720594
Adk	-0.128115275	0.760815595	0.118720594
Mrap	0.116178329	0.760888742	0.118678841
Prickle3	0.173562172	0.760894561	0.118675521
Atxn2	0.088532784	0.760894561	0.118675521
Pkn1	0.134914633	0.76093024	0.118655157
Vmn2r85	-0.100568249	0.761093804	0.118561814
Hbb-bt	-0.146298949	0.761093804	0.118561814
Atf2	-0.181867415	0.761093804	0.118561814
Coa7	-0.126166884	0.761093804	0.118561814
Ppp2cb	0.232774415	0.761124989	0.118544019
Stk26	-0.172668797	0.761124989	0.118544019
Cox11	0.182364508	0.761124989	0.118544019
Dut	0.057816561	0.761124989	0.118544019
NAT6	0.090889355	0.761124989	0.118544019
Ctnna1	0.10085092	0.761124989	0.118544019
Cxcr6	0.105634444	0.761124989	0.118544019
Pwp2	0.056256249	0.761124989	0.118544019
Gsr	0.064307422	0.761124989	0.118544019
Slc29a1	-0.099755808	0.761124989	0.118544019
Robo3	-0.092061383	0.761124989	0.118544019
Klhdc2	-0.109606509	0.761124989	0.118544019
Hsf2bp	-0.168299026	0.761124989	0.118544019
Mat2a	0.115449447	0.761597371	0.118274564
Dnaaf5	-0.10651011	0.761597371	0.118274564

Med12	-0.176801478	0.761800244	0.118158893
DFNB59	-0.11775226	0.76183771	0.118137534
Fbxw13	-0.123350731	0.761952176	0.118072286
Slc16a6	-0.085947809	0.761952176	0.118072286
Lrp11	-0.15136748	0.761952176	0.118072286
Cebpg	-0.113297338	0.761952176	0.118072286
Usp15	-0.099391181	0.762130388	0.117970721
Lck	0.0636358	0.762201592	0.117930148
Prkar2a	0.090502768	0.762201592	0.117930148
Pof1b	-0.067258511	0.762201592	0.117930148
Tbc1d22b	-0.070777441	0.762201592	0.117930148
Gpr153	0.134214273	0.762925135	0.117518077
Cd83	0.136131744	0.762925135	0.117518077
Actl10	-0.076249451	0.762925135	0.117518077
Ttc32	-0.088550988	0.762925135	0.117518077
Lefty2	-0.101694791	0.763401482	0.117247001
VPRBP	0.07007621	0.763402051	0.117246678
Anks4b	0.144910608	0.764580836	0.116576591
Vstm5	-0.069481343	0.764589622	0.116571601
Atg4a	-0.069121662	0.764812461	0.116445045
Mtmr7	0.129221295	0.764812461	0.116445045
Aimp2	-0.127387706	0.764812461	0.116445045
AV051173	0.129280529	0.764812461	0.116445045
Ccdc187	-0.123790836	0.764812461	0.116445045
Bcl7a	-0.098339531	0.764812461	0.116445045
Nrg1	-0.093702179	0.764812461	0.116445045
Fam124b	-0.077572213	0.764812461	0.116445045
Exoc6b	0.066222875	0.764812461	0.116445045
Nptn	0.142769605	0.764812461	0.116445045
Slc2a13	-0.121970049	0.764812461	0.116445045
Txnl4a	0.055080125	0.764812461	0.116445045
Plcz1	-0.092434331	0.764812461	0.116445045
Trappc6b	-0.097727678	0.764812461	0.116445045
Frk	0.145840953	0.764812461	0.116445045
Poln	0.296381644	0.764812461	0.116445045
Dmrt3	-0.180956172	0.765689592	0.115947256
Mospd3	0.081279758	0.765808475	0.115879832
Slc4a2	-0.464732388	0.766137891	0.115693058
Rtn3	-0.219475102	0.766137891	0.115693058
Them6	0.189849269	0.766137891	0.115693058
Ppp1r9b	-0.087676705	0.766137891	0.115693058
Wnt5b	-0.067097723	0.766137891	0.115693058
Vegfc	0.06007817	0.766137891	0.115693058
Asb18	0.154504449	0.766137891	0.115693058
Ranbp2	0.187215154	0.766137891	0.115693058
Frem1	-0.083369673	0.766137891	0.115693058
Itfg2	0.141347002	0.766137891	0.115693058
Tmem74b	-0.136400208	0.766611536	0.11542465

Klf3	-0.068105454	0.766611536	0.11542465
Crmp1	0.150517265	0.766798852	0.115318546
Ube2q2	-0.186284123	0.766798852	0.115318546
Hs3st1	0.100200235	0.766798852	0.115318546
Dppa3	-0.194138505	0.766798852	0.115318546
ATP5SL	-0.239146108	0.766798852	0.115318546
Kif24	0.081265048	0.766798852	0.115318546
Heatr5b	0.096288299	0.766798852	0.115318546
Zfp964	0.328677727	0.766798852	0.115318546
Mccc1os	0.173384456	0.766798852	0.115318546
Rabl6	-0.121909722	0.766798852	0.115318546
Hspa4	0.101939202	0.766798852	0.115318546
Gpc4	-0.108232888	0.766798852	0.115318546
Ldhal6b	0.060394215	0.766798852	0.115318546
Rn7sk	0.081165729	0.766798852	0.115318546
Asb3	0.133547207	0.766798852	0.115318546
Inha	0.067431924	0.766798852	0.115318546
LRRC16B	-0.150877625	0.766798852	0.115318546
Gemin2	0.096801018	0.766798852	0.115318546
USMG5	0.055557745	0.766798852	0.115318546
Prpf40b	0.153042345	0.766798852	0.115318546
Lmbr1	0.140454803	0.766798852	0.115318546
Brd9	-0.092149115	0.766907887	0.115256796
Col23a1	-0.093726463	0.766907887	0.115256796
Lin7b	0.067349156	0.767134544	0.11512846
Edaradd	0.117853354	0.767134544	0.11512846
Sox7	-0.116555835	0.767306274	0.115031251
Egfl6	-0.269545225	0.767483168	0.114931141
Acsl5	0.139002861	0.767483168	0.114931141
Tab2	-0.194471677	0.767483168	0.114931141
Cyp4f13	-0.102532452	0.767483168	0.114931141
Vmn2r57	0.103511398	0.767483168	0.114931141
Abhd11	0.08797509	0.767483168	0.114931141
Atp6v1b2	-0.071643287	0.767756032	0.114776763
Lars2	0.079648918	0.767805705	0.114748665
Ulk1	0.129248633	0.768047535	0.114611901
Angptl7	-0.18734636	0.768047535	0.114611901
Klhl8	-0.062279057	0.768047535	0.114611901
Dnah1	0.069802076	0.768047535	0.114611901
Arhgef11	-0.140337006	0.76885436	0.114155918
Lipo2	-0.163843892	0.76885436	0.114155918
Gng2	0.131396502	0.76885436	0.114155918
Xkr8	0.133675438	0.76885436	0.114155918
Elf2	-0.093730123	0.76885436	0.114155918
Rab8a	-0.084441991	0.76885436	0.114155918
Trem3	-0.090253779	0.76885436	0.114155918
Gabbr1	0.102738542	0.76885436	0.114155918
Lyrm4	0.055748465	0.76885436	0.114155918

Cntn5	0.136026036	0.769341089	0.113881072
Ppp1r9a	-0.141613999	0.769341089	0.113881072
Cbr4	0.050432326	0.769341089	0.113881072
Mid1ip1	0.094323903	0.769432742	0.113829337
Shc2	0.071458835	0.769490038	0.113796998
Wwox	0.074320032	0.769490038	0.113796998
Rnd3	0.074603525	0.769490038	0.113796998
Fbp1	0.108237606	0.769490038	0.113796998
Tmco6	-0.223910442	0.769516351	0.113782148
Celrr	0.052794407	0.769516351	0.113782148
Slc35g2	0.114379065	0.769516351	0.113782148
Fam89a	-0.086818618	0.769516351	0.113782148
Dusp15	0.053542453	0.769516351	0.113782148
Capsl	0.114042622	0.769516351	0.113782148
Rpl3l	0.124419348	0.769516351	0.113782148
Dcaf6	0.109658765	0.769516351	0.113782148
Efhc2	-0.135709483	0.769588759	0.113741285
Hint3	-0.384668125	0.769681704	0.113688837
Fam107a	-0.211345246	0.769681704	0.113688837
H2AFJ	-0.162047649	0.769681704	0.113688837
Gapdh-ps:	-0.154012567	0.769681704	0.113688837
Rps19	0.158246281	0.769681704	0.113688837
Cep83	-0.233801278	0.769681704	0.113688837
Rhcg	-0.084980802	0.769681704	0.113688837
Insig2	-0.082154153	0.769681704	0.113688837
Msx3	0.09261645	0.769681704	0.113688837
Pik3cd	0.184040526	0.769681704	0.113688837
Prkch	0.112493933	0.769681704	0.113688837
Klf4	0.103783081	0.769681704	0.113688837
Scg3	-0.086679938	0.769681704	0.113688837
Hagh	-0.087238545	0.769681704	0.113688837
FAM103A1	0.150752962	0.769681704	0.113688837
Oat	0.169127682	0.769681704	0.113688837
Fh1	0.081470799	0.769681704	0.113688837
Terb1	0.082134935	0.769681704	0.113688837
Hmg20a	-0.067720772	0.769681704	0.113688837
Klrb1b	0.137658294	0.769681704	0.113688837
Opa1	-0.051060944	0.769681704	0.113688837
Lif	0.121810093	0.769681704	0.113688837
Ankdd1b	0.082530629	0.769681704	0.113688837
Sptbn4	0.317062007	0.769681704	0.113688837
Lrp8	0.080570459	0.769681704	0.113688837
Syvn1	0.091268106	0.769681704	0.113688837
Glul	-0.097543959	0.769681704	0.113688837
Bhlhe22	0.081861456	0.769681704	0.113688837
Atp6v0e2	-0.066434208	0.769681704	0.113688837
Sdhaf3	-0.062632069	0.769681704	0.113688837
Nsmce2	0.175124073	0.769681704	0.113688837

Foxk2	-0.075041532	0.769681704	0.113688837
Aup1	0.125629176	0.769681704	0.113688837
Lncppara	0.100413658	0.769681704	0.113688837
Cyp26b1	0.089034392	0.769681704	0.113688837
Dsp	-0.081626543	0.769681704	0.113688837
Impa2	0.071472279	0.769681704	0.113688837
Rpl5	0.103532586	0.769681704	0.113688837
Stap1	0.077510792	0.769681704	0.113688837
Polr1d	0.114440873	0.769681704	0.113688837
Ppp2r2b	-0.158672963	0.769681704	0.113688837
Ahsa1	0.234447339	0.769681704	0.113688837
Clasp2	0.181608635	0.769681704	0.113688837
Crybb3	-0.086094772	0.769681704	0.113688837
Gzmb	0.064791863	0.769681704	0.113688837
Zbtb3	0.220875829	0.769681704	0.113688837
Bckdha	-0.068732279	0.769779423	0.113633702
Ly6k	-0.128094089	0.769779423	0.113633702
Ascl5	0.093282139	0.769779423	0.113633702
Med26	0.097220295	0.770779753	0.113069702
Nr1d2	-0.276756857	0.771202677	0.112831472
Agbl4	0.223888228	0.771202677	0.112831472
Lmbrd1	-0.08302784	0.771202677	0.112831472
Kat6b	0.083719545	0.771202677	0.112831472
Dpp9	0.091297327	0.771202677	0.112831472
Armc5	-0.10015675	0.771202677	0.112831472
Map4k2	-0.102515568	0.771202677	0.112831472
Ccno	-0.109876281	0.771202677	0.112831472
Oit3	0.068453874	0.771202677	0.112831472
A4gnt	0.094630802	0.771202677	0.112831472
Cenpu	-0.054560848	0.771202677	0.112831472
Olfr539	0.068472346	0.771202677	0.112831472
Rabepk	0.088140428	0.771208286	0.112828313
Dnm1l	0.091842837	0.771208286	0.112828313
Spred1	0.125738068	0.771208286	0.112828313
Zap70	-0.090192506	0.771208286	0.112828313
Myl1	0.055239066	0.771344528	0.112751597
Nhsl2	0.080006048	0.771344528	0.112751597
Vgll2	-0.255445996	0.771387948	0.112727151
Stard8	0.101172558	0.771517435	0.112654255
Pecam1	0.109598706	0.7716652	0.112571085
Zdhhc2	0.173069155	0.7716652	0.112571085
Slc25a39	0.293332926	0.771772031	0.112510965
Fmr1	-0.212593478	0.771972094	0.112398399
Rbm7	-0.20407191	0.771972094	0.112398399
Hspbp1	-0.268462093	0.771972094	0.112398399
Sox6	-0.224852978	0.771972094	0.112398399
Wdr12	-0.463719187	0.771972094	0.112398399
Mettl17	-0.19048068	0.771972094	0.112398399

Bloc1s1	0.201827464	0.771972094	0.112398399
Snx14	0.178233845	0.771972094	0.112398399
Ralgapb	-0.170680644	0.771972094	0.112398399
Lsm10	0.088359139	0.771972094	0.112398399
Cadm2	0.112884017	0.771972094	0.112398399
Gprin1	0.113859385	0.771972094	0.112398399
Pcdhb11	0.268984161	0.771972094	0.112398399
Il27	-0.098163854	0.771972094	0.112398399
Lbr	0.182716286	0.771972094	0.112398399
Rps5	-0.153275396	0.771972094	0.112398399
Ptpn14	0.110294322	0.771972094	0.112398399
Spon2	0.09232334	0.771972094	0.112398399
DIEXF	0.083893606	0.771972094	0.112398399
Gdf1	-0.143623523	0.771972094	0.112398399
Casc1	-0.124047674	0.771972094	0.112398399
Pram1	-0.096874386	0.771972094	0.112398399
Slc36a4	0.1526656	0.771972094	0.112398399
Il1rapl2	-0.102551389	0.771972094	0.112398399
Hmgb4	0.197451875	0.771972094	0.112398399
Slc4a3	0.20218189	0.771972094	0.112398399
Ylpm1	0.100546238	0.771972094	0.112398399
Arsb	-0.142504767	0.771972094	0.112398399
Syngr4	-0.083674871	0.771972094	0.112398399
Nek5	-0.09547618	0.771972094	0.112398399
Cd99l2	0.099313401	0.771972094	0.112398399
Pet117	0.181193365	0.771972094	0.112398399
Tmco5	-0.122238325	0.771972094	0.112398399
Fzd1	0.119716992	0.771972094	0.112398399
Ubr4	-0.077431467	0.771972094	0.112398399
Zfp804b	-0.170321531	0.771972094	0.112398399
Wdr4	0.071817189	0.771972094	0.112398399
Tspan13	-0.066826138	0.771972094	0.112398399
Lyst	-0.171368323	0.771972094	0.112398399
Rnpep	-0.166822145	0.771972094	0.112398399
Hid1	-0.198560381	0.771972094	0.112398399
Peli1	0.082640385	0.771972094	0.112398399
Prkcg	-0.21113312	0.771972094	0.112398399
Icam4	-0.214129976	0.771972094	0.112398399
Grik4	0.070163319	0.771972094	0.112398399
Nhp2	0.13492729	0.771972094	0.112398399
Pou6f2	0.104802498	0.771972094	0.112398399
Arhgef19	0.061828517	0.771972094	0.112398399
Spsb3	0.059286961	0.771972094	0.112398399
Prf1	0.18919791	0.771972094	0.112398399
Snhg15	-0.089687181	0.771972094	0.112398399
Rasa3	0.068836499	0.771972094	0.112398399
Tgm4	0.287153586	0.771972094	0.112398399
Erbb3	-0.096987448	0.771972094	0.112398399

Rbm12b1	0.148540732	0.771972094	0.112398399
Six4	0.136353908	0.771972094	0.112398399
Adam9	0.114062329	0.771972094	0.112398399
Map1lc3a	-0.091520308	0.771972094	0.112398399
Fgf7	0.073672066	0.771972094	0.112398399
Sord	-0.091103068	0.771972094	0.112398399
FAM49A	0.100692162	0.771972094	0.112398399
Cttnbp2	0.135967025	0.771972094	0.112398399
CAHM	0.115372298	0.771972094	0.112398399
Gtpbp10	0.158393484	0.771972094	0.112398399
Agmo	-0.107988133	0.771972094	0.112398399
Eapp	0.724791463	0.771972094	0.112398399
Snrpb2	-0.107781144	0.771972094	0.112398399
FAM196B	0.149298381	0.771972094	0.112398399
Maneal	0.106480501	0.771972094	0.112398399
Mpo	0.1409868	0.771972094	0.112398399
Kctd12b	0.23855928	0.771972094	0.112398399
Rab9b	-0.066575966	0.771972094	0.112398399
Nubp1	0.186859894	0.771972094	0.112398399
Mettl3	0.103026608	0.771972094	0.112398399
Faap20	0.104287808	0.771972094	0.112398399
Sacm1l	0.073133156	0.771972094	0.112398399
Slc35f4	0.121559609	0.771972094	0.112398399
Pip5k1a	0.072744295	0.771972094	0.112398399
Foxo6os	0.085361599	0.771972094	0.112398399
Itga7	0.125204945	0.771972094	0.112398399
Picalm	0.113302956	0.771972094	0.112398399
Dcaf12l1	0.129772624	0.771972094	0.112398399
Crlf3	0.077534766	0.771972094	0.112398399
Rgcc	-0.158214172	0.771972094	0.112398399
Arhgef9	0.071005019	0.771972094	0.112398399
Nccrp1	-0.076496965	0.771972094	0.112398399
Trmt10a	-0.09213584	0.771972094	0.112398399
Efcab14	-0.148778071	0.771972094	0.112398399
Slc26a11	-0.061024408	0.771972094	0.112398399
Tnfsf9	0.080220139	0.771972094	0.112398399
Papolb	0.084679338	0.771972094	0.112398399
St6galnac	-0.084230063	0.771972094	0.112398399
BC021891	0.080699	0.771972094	0.112398399
Cckar	0.23706825	0.771972094	0.112398399
Grid1	0.066352724	0.771972094	0.112398399
Lzic	-0.133840274	0.771972094	0.112398399
Megf8	0.057427885	0.771972094	0.112398399
Pign	0.096984559	0.771972094	0.112398399
Cyp2e1	-0.079547572	0.771972094	0.112398399
Ccdc125	0.111631516	0.771972094	0.112398399
Zfp941	0.07641144	0.771972094	0.112398399
Nif3l1	0.156593461	0.771972094	0.112398399

Erp29	0.113489574	0.771972094	0.112398399
Coch	0.073061751	0.771972094	0.112398399
Foxo3	-0.050002627	0.771972094	0.112398399
Akap10	0.221656219	0.771972094	0.112398399
Mybphl	-0.106170886	0.771972094	0.112398399
Siae	0.582463341	0.771972094	0.112398399
Ccnt1	-0.087259872	0.771972094	0.112398399
Ncoa1	0.096583077	0.771972094	0.112398399
Lad1	-0.150064015	0.771972094	0.112398399
Zgpat	-0.119250946	0.771972094	0.112398399
Dgki	-0.108629746	0.771972094	0.112398399
Dnaja2	0.08357531	0.771972094	0.112398399
Smoc1	0.084485585	0.771972094	0.112398399
Fnbp1l	0.096310249	0.771972094	0.112398399
Rptor	0.061466699	0.771972094	0.112398399
Fbxl16	-0.167715503	0.771972094	0.112398399
Pparg	0.073358359	0.771972094	0.112398399
Nudcd2	0.07414607	0.771972094	0.112398399
D4ERTD61	0.185256514	0.772396015	0.112159975
Col4a4	0.165106816	0.772478193	0.112113772
Morn4	0.150880029	0.772478193	0.112113772
B4gat1	-0.226065533	0.772478193	0.112113772
Zmat2	-0.129780766	0.772478193	0.112113772
Glud1	-0.102812436	0.772478193	0.112113772
Osbpl5	0.100624528	0.772478193	0.112113772
Sp6	0.126316938	0.772478193	0.112113772
Map2	0.154338163	0.772478193	0.112113772
Bcar1	0.080213155	0.772478193	0.112113772
Umad1	0.074218687	0.772478193	0.112113772
Sp2	0.071312852	0.772478193	0.112113772
Ezh1	0.096220434	0.772478193	0.112113772
Jade1	0.172434984	0.772478193	0.112113772
Nfkbid	-0.089804754	0.772572826	0.112060572
Mroh7	0.063153195	0.772875658	0.111890371
Zfp251	0.111149522	0.773156157	0.111732781
Ppp1r10	-0.236933438	0.773156157	0.111732781
BC029214	0.063038092	0.773190038	0.11171375
Sncaip	-0.208671372	0.773436919	0.111575101
Cxcl3	0.260874689	0.773685187	0.111435718
FAM213B	-0.274491073	0.773685187	0.111435718
6-Mar	-0.114137604	0.773685187	0.111435718
Wdr49	0.094331681	0.773685187	0.111435718
Abr	-0.10018759	0.773685187	0.111435718
Styk1	0.066376679	0.773685187	0.111435718
Gramd3	-0.080869123	0.773685187	0.111435718
Plekhm2	0.069037969	0.773685187	0.111435718
Zfp953	-0.141303567	0.773685187	0.111435718
Pla2g5	-0.150976663	0.773848618	0.111343989

Rhot2	-0.205932884	0.773848618	0.111343989
Epb41l5	-0.222307799	0.773848618	0.111343989
Sun2	0.078665259	0.773848618	0.111343989
Btbd9	-0.116725937	0.773848618	0.111343989
Ugcg	0.106429017	0.773848618	0.111343989
Sox18	-0.102537283	0.773848618	0.111343989
Ptger1	-0.079321668	0.773848618	0.111343989
Aco1	0.156993695	0.773848618	0.111343989
Aplp2	0.134163424	0.773848618	0.111343989
Siglecg	-0.089980495	0.773848618	0.111343989
Zfp791	0.14832378	0.773848618	0.111343989
Ephx2	-0.063614974	0.773848618	0.111343989
Pde4dip	-0.063913429	0.773848618	0.111343989
Matk	-0.12877686	0.773848618	0.111343989
Tspan17	-0.067213109	0.773848618	0.111343989
Chrna10	-0.055362578	0.773848618	0.111343989
Cry2	0.054056254	0.773868927	0.111332591
Acad10	0.115861965	0.773868927	0.111332591
Mterf2	-0.079887597	0.774049534	0.111231246
Gapdhs	0.074235973	0.774049534	0.111231246
Shisa7	-0.107126774	0.774049534	0.111231246
Ndst2	-0.166451684	0.774992355	0.110702582
Klra2	-0.085106574	0.774992355	0.110702582
Tmem233	0.179846015	0.775060785	0.110664236
Myh10	0.228138376	0.775060785	0.110664236
11-Sep	-0.087784079	0.775060785	0.110664236
Wdr7	-0.08239827	0.775060785	0.110664236
Polg2	-0.157916938	0.775060785	0.110664236
Rpn2	0.129298574	0.775060785	0.110664236
Ptger2	-0.074618596	0.775060785	0.110664236
Aox3	0.072904378	0.775060785	0.110664236
Cdk18	0.105279186	0.775060785	0.110664236
Klrd1	0.252430988	0.775060785	0.110664236
Cldn5	0.062141262	0.775060785	0.110664236
Fsd1l	0.07427974	0.775243994	0.11056159
Rab28	-0.063811057	0.775243994	0.11056159
DOPEY2	0.137450561	0.775421374	0.110462232
Borcs6	0.266307937	0.775421374	0.110462232
Klhl28	0.08756125	0.775421374	0.110462232
Mkks	0.094458991	0.775421374	0.110462232
Trpv4	0.066702121	0.775421374	0.110462232
Erv3	-0.134499304	0.775421374	0.110462232
Drp2	-0.117208894	0.775421374	0.110462232
Kif1b	0.177839628	0.775421374	0.110462232
Pappa2	-0.081405954	0.775421374	0.110462232
Cables2	0.09544107	0.775421374	0.110462232
Dlg2	-0.22377167	0.775421374	0.110462232
Pex26	0.062196676	0.775421374	0.110462232

Camsap1	-0.31751872	0.775421374	0.110462232
Mlana	0.194647262	0.775421374	0.110462232
1-Sep	0.137223677	0.775421374	0.110462232
Spin4	-0.141393443	0.775421374	0.110462232
Gon4l	0.099397986	0.775421374	0.110462232
Mydgf	0.070946576	0.775421374	0.110462232
Ift81	0.127981264	0.775421374	0.110462232
Phldb1	0.123459972	0.775421374	0.110462232
Piwil2	0.133752068	0.775421374	0.110462232
Anapc13	0.104143056	0.775421374	0.110462232
Trpt1	-0.050299935	0.775421374	0.110462232
Gsx1	0.162584254	0.775421374	0.110462232
Dst	0.062751881	0.775421374	0.110462232
Fads6	0.190342808	0.775421374	0.110462232
Wdsub1	0.090378031	0.775421374	0.110462232
Tmed8	0.128439325	0.775421374	0.110462232
Ms4a4d	-0.056140207	0.775421374	0.110462232
Rnpepl1	0.063343073	0.775421374	0.110462232
Atl3	-0.162075246	0.775502288	0.110416917
Gja5	-0.068262773	0.775502288	0.110416917
Spx	-0.060095313	0.775516133	0.110409163
Chpt1	-0.138118691	0.775516133	0.110409163
Atp8b5	-0.134880704	0.77568167	0.110316471
Pkdcc	-0.130593982	0.775909192	0.110189103
Slc39a12	-0.064159713	0.775909192	0.110189103
Tubgcp6	-0.128459004	0.775909192	0.110189103
Tarbp2	-0.127651904	0.776121353	0.110070368
Eif4a2	0.106489829	0.776121353	0.110070368
Rpl22	-0.072988992	0.776121353	0.110070368
Eno2	-0.063553408	0.776271225	0.109986512
Btd	-0.053623605	0.776271225	0.109986512
Tmem132	0.075766438	0.776271225	0.109986512
Klhl36	0.225425021	0.776271225	0.109986512
Aqp3	-0.097788562	0.776271225	0.109986512
Cdk15	-0.075064925	0.776271225	0.109986512
Atp9a	-0.059173681	0.776271225	0.109986512
Lyzl4	0.068535162	0.776271225	0.109986512
Pigt	0.095793371	0.776271225	0.109986512
Hip1r	-0.087580955	0.776271225	0.109986512
Snx6	0.081892398	0.776283621	0.109979577
Rnf128	-0.078179659	0.776283621	0.109979577
Tmem114	0.144418275	0.776552431	0.109829216
Cyp4b1	0.062771371	0.776552431	0.109829216
Atp8b3	-0.115326594	0.776552431	0.109829216
Ttpal	0.065227573	0.776552431	0.109829216
Meaf6	-0.081769091	0.776552431	0.109829216
Dusp28	0.081742647	0.776552431	0.109829216
Arhgef17	0.06892175	0.776552431	0.109829216

Pcdhb15	0.080932374	0.776628443	0.109786708
Abcb9	-0.328554208	0.776957826	0.109602555
Per1	0.122411442	0.776957826	0.109602555
Atp2c2	0.261306316	0.776957826	0.109602555
Cacna1e	-0.109143998	0.776957826	0.109602555
Bptf	0.073595638	0.776957826	0.109602555
Zfp644	-0.099329025	0.777242184	0.109443637
Slc7a1	0.111433083	0.777242184	0.109443637
Trim6	-0.151401607	0.777300402	0.109411108
Tnfsf11	0.094026349	0.777319461	0.109400459
Dock5	0.335789328	0.777697352	0.10918938
Ccdc170	0.121645017	0.777697352	0.10918938
Trim75	-0.220198737	0.777697352	0.10918938
Srcin1	0.097340964	0.777697352	0.10918938
Ust	0.277138418	0.777697352	0.10918938
Sox12	-0.076032714	0.777697352	0.10918938
Kcnj13	-0.076252711	0.777697352	0.10918938
Tox3	-0.154314183	0.777697352	0.10918938
Lyrm2	0.05888693	0.777697352	0.10918938
Uqcc1	-0.145744113	0.777697352	0.10918938
Rab3gap1	0.087040013	0.777697352	0.10918938
Anp32b	0.08536032	0.777697352	0.10918938
Cacnb2	0.062270352	0.777697352	0.10918938
Robo2	0.128321635	0.777697352	0.10918938
Rd3l	0.086534396	0.777697352	0.10918938
Fbxl17	0.076490596	0.777697352	0.10918938
Myo5c	-0.094687334	0.777697352	0.10918938
Kbtbd11	-0.120076573	0.777697352	0.10918938
Ptpro	0.087692282	0.777697352	0.10918938
Dpp6	-0.261868423	0.777697352	0.10918938
Dpt	0.137702315	0.777697352	0.10918938
Prkacb	0.144587795	0.777697352	0.10918938
Klrk1	-0.142704807	0.777852159	0.109102939
Usp19	0.174852959	0.777852159	0.109102939
Serpinb5	-0.068331284	0.777852159	0.109102939
Ror2	-0.070298234	0.777852159	0.109102939
Tcfl5	-0.119285925	0.777852159	0.109102939
Atp5g2	0.132667625	0.777852159	0.109102939
FAM57A	0.087612508	0.777852159	0.109102939
Mipep	0.19304062	0.778082488	0.108974359
Il17c	0.069736227	0.778082488	0.108974359
Cdc42bpg	-0.185973849	0.77823882	0.10888711
Ago2	0.07676216	0.77823882	0.10888711
Rpl6	-0.171329348	0.77823882	0.10888711
Ankrd11	-0.176810259	0.77823882	0.10888711
Actr3b	-0.056202074	0.77823882	0.10888711
Hsd3b6	0.120671545	0.77823882	0.10888711
Olfr920	0.122883699	0.77823882	0.10888711

Asb14	0.160482237	0.77823882	0.10888711
Rpl28	0.15487071	0.77823882	0.10888711
Plk4	0.1273279	0.77823882	0.10888711
Tmem253	0.119667447	0.77823882	0.10888711
Glp2r	-0.099325564	0.77823882	0.10888711
Vcpip1	0.121415493	0.77823882	0.10888711
Plch1	0.112805001	0.77823882	0.10888711
Rgs9	-0.119210626	0.77823882	0.10888711
Senp5	-0.084496032	0.77823882	0.10888711
Mef2d	0.120648748	0.77823882	0.10888711
Stard10	0.237765255	0.77823882	0.10888711
Dlec1	0.143931399	0.77823882	0.10888711
Setd4	0.07693929	0.77823882	0.10888711
Sgce	0.096175013	0.77823882	0.10888711
Tmem45b	0.077776764	0.77823882	0.10888711
Cmc2	0.061532855	0.77823882	0.10888711
Eldr	-0.049630728	0.77823882	0.10888711
Zbtb11	0.152452885	0.77823882	0.10888711
Brinp3	0.168824614	0.77823882	0.10888711
Atf5	-0.072705409	0.77823882	0.10888711
Prex1	0.073127423	0.77823882	0.10888711
Xk	-0.071319111	0.77823882	0.10888711
Ss18l1	-0.048276488	0.77823882	0.10888711
Ppp1r11	0.073492694	0.77823882	0.10888711
Commd8	0.181368601	0.77823882	0.10888711
Lhfpl5	0.056056033	0.77823882	0.10888711
Tspan14	0.084583695	0.77823882	0.10888711
Slc6a15	0.096380531	0.77823882	0.10888711
Cd8b1	0.09532909	0.77823882	0.10888711
Rrp8	-0.063830753	0.77823882	0.10888711
Aftph	0.112556167	0.77823882	0.10888711
Zbtb46	0.068919892	0.77823882	0.10888711
Dpagt1	-0.050739608	0.77823882	0.10888711
Alkbh1	0.07811106	0.77823882	0.10888711
Rapgef2	-0.075146218	0.77823882	0.10888711
Gfra4	-0.079067584	0.77823882	0.10888711
Klf7	0.086799583	0.77823882	0.10888711
Nr0b2	-0.069822968	0.77823882	0.10888711
Dok5	0.046918222	0.77823882	0.10888711
AW14615	0.076485236	0.77823882	0.10888711
Csmd3	0.06597329	0.77823882	0.10888711
Fmn2	0.050877688	0.77823882	0.10888711
Huwe1	0.303331683	0.778287969	0.108859683
Slc25a14	-0.167492361	0.778376945	0.108810036
Prr5	0.126615355	0.778786268	0.108581715
Ier2	-0.101058696	0.778786268	0.108581715
Gchfr	-0.063479675	0.778786268	0.108581715
Ocstamp	0.093661113	0.778786268	0.108581715

Hspb2	0.207218755	0.778786268	0.108581715
Htra4	0.067073224	0.779328745	0.108279305
Dhrs7	0.136287167	0.779413464	0.108232096
Ttll1	0.090335714	0.779413464	0.108232096
Ssx2ip	-0.056622046	0.779490527	0.108189158
Nf1	0.18770369	0.779677104	0.108085219
Snrk	0.136350744	0.779677104	0.108085219
Lypd6	-0.125230945	0.779677104	0.108085219
Fam13a	-0.141700503	0.779677104	0.108085219
Ror1	-0.084185785	0.779677104	0.108085219
Aqp12	-0.077337892	0.779677104	0.108085219
Sdr39u1	0.08222067	0.779677104	0.108085219
Lrrc71	-0.094730183	0.779677104	0.108085219
Ubl7	0.094763204	0.779677104	0.108085219
Cct6b	0.100945097	0.779677104	0.108085219
Exosc5	0.072332556	0.779677104	0.108085219
Ifit1bl1	0.143997922	0.779677104	0.108085219
Fam98c	-0.093860784	0.779677104	0.108085219
Wdr86	0.181680667	0.779978346	0.107917454
Fmn1	0.301836743	0.780205817	0.107790816
Acpp	0.081051678	0.780205817	0.107790816
Pln	0.079429195	0.780205817	0.107790816
Fosl2	0.083070806	0.780562161	0.107592506
Ccdc186	-0.09033133	0.780562161	0.107592506
Plekhd1	-0.083929048	0.780562161	0.107592506
Jph2	-0.097460205	0.780562161	0.107592506
Rpl13a	-0.19153488	0.780562161	0.107592506
Cetn4	0.056287145	0.780562161	0.107592506
Strip1	-0.198343532	0.780562161	0.107592506
Rgl2	-0.139406546	0.780562161	0.107592506
Tmem132	-0.083533902	0.780562161	0.107592506
Socs6	0.197362475	0.780686581	0.107523286
Napa	0.094924552	0.780753968	0.1074858
Neurl4	-0.09605927	0.780753968	0.1074858
Tm9sf1	0.067636706	0.780860882	0.107426333
Cidea	-0.07053808	0.780870865	0.107420781
Idh3a	0.113973734	0.780870865	0.107420781
Zfp773	-0.107912429	0.780870865	0.107420781
METTL20	0.081470404	0.780870865	0.107420781
Ube3a	0.081215817	0.780870865	0.107420781
ZBED6	-0.108205897	0.780870865	0.107420781
HIST2H2A(	-0.069050742	0.780870865	0.107420781
Mrpl38	-0.105451752	0.780870865	0.107420781
Nubp2	0.05888433	0.780870865	0.107420781
Pygb	0.232021172	0.781077805	0.107305703
Helz	-0.144255977	0.781089586	0.107299152
Apc2	-0.337585087	0.781089586	0.107299152
Klhl12	0.095069512	0.781089586	0.107299152

Foxd2	-0.074666206	0.781133898	0.107274515
Hira	0.105639415	0.781181828	0.107247868
Fam3a	-0.07246354	0.781523548	0.107057932
Cdadc1	-0.137656906	0.782789242	0.106355152
Fam229b	0.053361989	0.782789242	0.106355152
Ints10	-0.258763132	0.782893828	0.106297131
Styx	-0.0897201	0.783337178	0.106051261
Got1l1	0.076440675	0.783337178	0.106051261
Rita1	0.07146281	0.7835814	0.105915881
Gstm4	-0.08440829	0.7835814	0.105915881
Pdpk1	0.202280304	0.783587356	0.105912581
Kctd13	0.090785252	0.783587356	0.105912581
Mocs3	0.288136669	0.783587356	0.105912581
Tcf20	-0.117618996	0.783591787	0.105910125
Tle6	-0.06344927	0.783591787	0.105910125
Usp29	-0.155661262	0.783591787	0.105910125
Wbp2	0.075271597	0.783591787	0.105910125
Ripk2	0.077111148	0.783591787	0.105910125
Smyd5	-0.060672391	0.783591787	0.105910125
Ptgis	-0.199080975	0.783591787	0.105910125
Zfp935	0.081603259	0.783606976	0.105901706
Igfbp4	-0.068295789	0.783606976	0.105901706
Rapgef5	0.095138867	0.783606976	0.105901706
Spag5	0.069795458	0.783606976	0.105901706
Ucma	0.402050778	0.783965643	0.10570297
Neil1	0.140515273	0.783965643	0.10570297
Crip3	-0.088338829	0.784096572	0.105630445
Zbtb17	0.06653749	0.784096572	0.105630445
Txn1	0.090096413	0.784096572	0.105630445
Suds3	-0.082746198	0.784096572	0.105630445
Cadm3	0.085599105	0.784134658	0.10560935
Csnk1e	0.196715775	0.784167968	0.105590902
Aunip	-0.3816203	0.784434793	0.105443152
Ces1a	-0.082135676	0.784947267	0.105159518
Rad51d	0.174596561	0.784947267	0.105159518
Tmem91	-0.099036492	0.784947267	0.105159518
Hsdl2	-0.13105171	0.785091186	0.105079898
Lrrc29	0.092817893	0.785424552	0.104895527
Ube2n	0.380392341	0.785564074	0.104818386
Afg3l1	-0.156054179	0.785564074	0.104818386
Rad1	0.267946371	0.785564074	0.104818386
Tmem266	0.137917693	0.785564074	0.104818386
Kdm6b	0.123219765	0.785564074	0.104818386
Cd209g	0.103613724	0.785564074	0.104818386
Lrrc34	-0.065552219	0.785564074	0.104818386
Arhgef1	-0.08056497	0.785564074	0.104818386
Wdr76	0.137897992	0.785564074	0.104818386
Abcc5	-0.302263638	0.785564074	0.104818386

Myoc	0.119759223	0.785564074	0.104818386
Cklf	0.112656797	0.785564074	0.104818386
Slc12a6	-0.126813943	0.785564074	0.104818386
Cd8a	0.083964132	0.785564074	0.104818386
Ino80dos	-0.136722669	0.785564074	0.104818386
Fst	-0.091202129	0.785564074	0.104818386
Tmem150	0.066390771	0.785564074	0.104818386
Efcab12	0.080344485	0.785564074	0.104818386
Ano10	0.077342743	0.785564074	0.104818386
Chd7	0.17910544	0.785564074	0.104818386
Rrh	0.084645837	0.785564074	0.104818386
Cacnb1	-0.093360698	0.785564074	0.104818386
Gpr146	-0.067035365	0.785564074	0.104818386
Pop1	-0.06744815	0.785564074	0.104818386
Acvrl1	0.060214291	0.785564074	0.104818386
Pepd	0.084555223	0.785564074	0.104818386
Capn2	0.244741075	0.785564074	0.104818386
Zfp248	0.064833997	0.785564074	0.104818386
Rab11fip3	0.168910929	0.785564074	0.104818386
Tgds	-0.110285936	0.785564074	0.104818386
Ccdc81	0.069889405	0.785564074	0.104818386
Sele	0.301488616	0.785564074	0.104818386
Nars	-0.073932854	0.785564074	0.104818386
Spns2	-0.05596312	0.785564074	0.104818386
Wscd1	-0.078588765	0.785564074	0.104818386
Taf10	0.073696422	0.785564074	0.104818386
Nans	0.049389374	0.785564074	0.104818386
Fbxl7	-0.232691896	0.786053224	0.104548047
Fam163a	-0.057248721	0.786053224	0.104548047
Sfxn3	-0.132402165	0.786367387	0.104374507
Cmip	0.160529613	0.786367387	0.104374507
Mfge8	-0.183601456	0.786367387	0.104374507
Cercam	0.04903288	0.786367387	0.104374507
Saysd1	-0.163940643	0.786737187	0.104170321
Cacfd1	-0.191724116	0.786737187	0.104170321
Ano5	-0.10973543	0.786737187	0.104170321
Wnt3	-0.101483202	0.786737187	0.104170321
Rnf41	0.055525207	0.786737187	0.104170321
Matr3	0.131949275	0.786737187	0.104170321
Prdm16	0.096592103	0.786737187	0.104170321
Klhl15	-0.200398242	0.786737187	0.104170321
Tmppe	-0.075626405	0.786737187	0.104170321
Uspl1	0.057082614	0.786737187	0.104170321
Serf2	-0.072405544	0.786737187	0.104170321
Spry2	0.202975916	0.787015029	0.104016974
Ppp1r26	-0.100851169	0.787015029	0.104016974
Cntd1	0.086054916	0.787015029	0.104016974
Tusc2	-0.124372568	0.787180664	0.103925582

Igf1r	0.292009557	0.787334283	0.103840838
Pdcl3	-0.110955994	0.787385013	0.103812856
Rpl14	0.210649179	0.787385013	0.103812856
Cldn12	0.101436126	0.787385013	0.103812856
Hace1	-0.115587817	0.787385013	0.103812856
Rab11fip5	0.114020421	0.787385013	0.103812856
Bex2	-0.175180192	0.787385013	0.103812856
Dkk2	-0.209809617	0.787385013	0.103812856
Myo9a	0.082165771	0.787385013	0.103812856
Abhd5	0.07187058	0.787385013	0.103812856
Lancl1	0.074768337	0.787385013	0.103812856
MLLT4	0.063192811	0.787385013	0.103812856
Zfp799	-0.072643184	0.787385013	0.103812856
Gpr141	-0.263068653	0.787426108	0.10379019
Zim1	0.099479594	0.787426108	0.10379019
Snx25	-0.164390933	0.787426108	0.10379019
Jpx	0.069597269	0.787426108	0.10379019
Fbxl19	-0.186171501	0.787426108	0.10379019
Depdc1b	-0.157920516	0.787426108	0.10379019
Mrm1	0.077847446	0.787426108	0.10379019
Aldh1b1	0.073357338	0.787426108	0.10379019
Fam83f	0.13649444	0.787426108	0.10379019
Pde8b	0.067301551	0.787426108	0.10379019
Ugt1a6b	0.045748378	0.787426108	0.10379019
P3h4	0.121724495	0.78754035	0.103727186
Carmn	-0.343959752	0.787543592	0.103725398
Npdc1	-0.237657208	0.787543592	0.103725398
Nup93	0.093979055	0.787543592	0.103725398
Impact	0.157228839	0.787543592	0.103725398
Zfp457	-0.07868055	0.787543592	0.103725398
Sun1	-0.103786041	0.787543592	0.103725398
Pih1d1	0.139481279	0.787543592	0.103725398
Mtf2	-0.102865113	0.787543592	0.103725398
Shisa6	-0.282889935	0.787543592	0.103725398
Chic1	-0.07618972	0.787543592	0.103725398
Rpl11	0.074485262	0.787543592	0.103725398
Gclm	0.144889126	0.787543592	0.103725398
Adam3	0.188159026	0.787543592	0.103725398
Kantr	0.075670776	0.787543592	0.103725398
Mycn	-0.068398393	0.787543592	0.103725398
Elmo3	0.12851632	0.787543592	0.103725398
Pmm2	-0.101299846	0.787543592	0.103725398
Gnb1	0.073646257	0.787543592	0.103725398
Gpm6a	-0.131805046	0.787543592	0.103725398
Aig1	0.067200984	0.787543592	0.103725398
Napb	0.110688197	0.787543592	0.103725398
Slc35f6	0.071920555	0.787543592	0.103725398
Asgr2	-0.342638134	0.787861013	0.10355039

Syne3	-0.167569397	0.787861013	0.10355039
Phkb	0.120142465	0.788121724	0.103406701
Pcsk2os1	-0.228404318	0.788442599	0.103229919
Ap4s1	-0.665385582	0.788442599	0.103229919
Pcgf2	0.213127265	0.788442599	0.103229919
Eif2ak1	0.082348528	0.788442599	0.103229919
Ucn3	0.483419696	0.788447818	0.103227044
D16Ertd47	-0.070104397	0.788447818	0.103227044
Hyou1	0.166713242	0.788531014	0.103181221
Dlg1	0.077889174	0.788877244	0.102990571
Spryd7	0.089573598	0.789101196	0.102867298
Susd6	0.049588327	0.789101196	0.102867298
Gmeb2	-0.108557492	0.789101196	0.102867298
Epn1	-0.081126812	0.789101196	0.102867298
Hsd17b7	-0.082247024	0.789101196	0.102867298
Cobll1	0.054816739	0.789153352	0.102838595
Mbnl1	-0.205015941	0.78956406	0.102612629
Rtp3	0.075084558	0.78973651	0.102517784
Tsga10	-0.329941331	0.789938056	0.102406963
Dclre1b	0.064382116	0.789938056	0.102406963
Lmntd1	0.109124269	0.789938056	0.102406963
Alkbh3os1	0.119899263	0.789938056	0.102406963
Xrcc3	0.069954699	0.790092434	0.102322097
Ptges2	0.165102417	0.790092434	0.102322097
Olfr1034	0.078651385	0.790092434	0.102322097
Ky	0.237867387	0.790092434	0.102322097
Dnajb6	0.097419605	0.790092434	0.102322097
Nek3	-0.275586941	0.790179592	0.102274191
Ptges3l	0.058302892	0.790179592	0.102274191
Dixdc1	0.10392604	0.790179592	0.102274191
Polr2i	0.057317677	0.790179592	0.102274191
Sptbn1	0.113681092	0.790179592	0.102274191
Sbno1	-0.082367885	0.790179592	0.102274191
Flt1	-0.101795435	0.790179592	0.102274191
Tlcd1	-0.090945405	0.790179592	0.102274191
Prr19	-0.065709619	0.790179592	0.102274191
Nup160	-0.104817531	0.790209488	0.10225776
Tie1	-0.130242964	0.790209488	0.10225776
Phlda3	0.072027888	0.790209488	0.10225776
Bcl2	-0.122211453	0.790209488	0.10225776
Pank1	0.065842594	0.790209488	0.10225776
Mmaa	-0.076825286	0.790209488	0.10225776
Klhl18	-0.134869765	0.790209488	0.10225776
Kbtbd8	0.126251201	0.790209488	0.10225776
Ncor1	0.064432931	0.790209488	0.10225776
Tmbim6	-0.133193675	0.7902299	0.102246542
Gbx1	-0.102158879	0.7902299	0.102246542
Hspa8	0.261809559	0.7902299	0.102246542

Srpx2	-0.074871752	0.790514863	0.10208996
Tube1	0.058607974	0.790514863	0.10208996
Usp7	0.10233571	0.790514863	0.10208996
8-Sep	-0.081199299	0.790514863	0.10208996
Dpy19l4	0.089245374	0.790514863	0.10208996
Gad1	0.245041012	0.790514863	0.10208996
Crb2	0.079561726	0.790514863	0.10208996
Csrp3	0.139197689	0.790514863	0.10208996
Shprh	0.07396794	0.790514863	0.10208996
Got2	-0.076242878	0.790686858	0.10199548
Mcmbp	0.064134671	0.790686858	0.10199548
Thsd7a	0.119239341	0.790738134	0.101967316
Apex2	-0.070657413	0.790738134	0.101967316
Zfp575	-0.244486085	0.790766279	0.101951859
GYLTL1B	0.340401986	0.790766279	0.101951859
Pop5	0.243358377	0.790766279	0.101951859
Hspb1	0.108306994	0.790864677	0.101897821
Poc5	-0.052983762	0.790864677	0.101897821
Znrf1	-0.096624091	0.790864677	0.101897821
Rbp2	0.358861136	0.790864677	0.101897821
Rnls	0.084602622	0.790887781	0.101885134
Srrm4os	0.126184403	0.791827369	0.101369491
Prr14l	-0.225343091	0.791827369	0.101369491
Polm	0.094591581	0.791827369	0.101369491
Dnttip2	-0.149942099	0.791827369	0.101369491
Cachd1	-0.196404164	0.791827369	0.101369491
LDOC1L	-0.173177917	0.791827369	0.101369491
Pdgfd	0.574423322	0.791827369	0.101369491
Aldh2	-0.100570414	0.791827369	0.101369491
Spata24	-0.094302301	0.791827369	0.101369491
Ahcyl2	-0.151914777	0.791827369	0.101369491
Odf3l1	0.090820297	0.791827369	0.101369491
Shq1	0.29553612	0.791827369	0.101369491
Taf13	-0.241679904	0.791827369	0.101369491
Letm2	-0.117984617	0.791827369	0.101369491
Cdc123	0.088162946	0.791827369	0.101369491
Hnf1b	-0.090941124	0.791827369	0.101369491
Gpt	0.069035868	0.791827369	0.101369491
Smarcc2	-0.067035475	0.791827369	0.101369491
Cd59b	0.185029694	0.791827369	0.101369491
Klhdc9	-0.112132748	0.791827369	0.101369491
Iqcb1	0.149297809	0.791827369	0.101369491
Tmtc4	0.067244544	0.791827369	0.101369491
SEPN1	0.077450082	0.791827369	0.101369491
Xrra1	0.082881747	0.791827369	0.101369491
Nuak1	-0.075014274	0.791827369	0.101369491
Rpl12	0.079920073	0.791827369	0.101369491
Kif13b	-0.058891078	0.791827369	0.101369491

Eef1b2	0.057929036	0.791827369	0.101369491
Mapk6	0.100106721	0.791827369	0.101369491
Arfgap1	0.08837578	0.791827369	0.101369491
Tekt5	0.072986026	0.791827369	0.101369491
Serinc1	0.092413575	0.791827369	0.101369491
Ppef2	0.141269484	0.791827369	0.101369491
Kif2a	0.098997024	0.791827369	0.101369491
Msra	-0.155059953	0.791827369	0.101369491
Cdc42ep1	-0.098650555	0.791827369	0.101369491
Srrm3	0.078927691	0.791827369	0.101369491
Dll1	-0.096491859	0.791827369	0.101369491
Tagln3	0.059633062	0.791827369	0.101369491
Ddhd2	0.093214526	0.791827369	0.101369491
Lrrc51	0.052728182	0.791827369	0.101369491
Klf9	0.061538505	0.791827369	0.101369491
Mdm2	0.073740237	0.791827369	0.101369491
Poc1a	0.11569505	0.791827369	0.101369491
Tmem86b	0.080270991	0.791827369	0.101369491
Prkx	-0.063516521	0.791827369	0.101369491
Ubfd1	-0.047132445	0.791827369	0.101369491
Tex261	0.079813026	0.791827369	0.101369491
Cyb561d1	-0.113498908	0.791827369	0.101369491
Dvl1	0.126109042	0.791827369	0.101369491
Rab3ip	-0.087610827	0.791827369	0.101369491
Fbln7	-0.084181544	0.791827369	0.101369491
Gtf2h4	0.156477139	0.791827369	0.101369491
Hdac5	-0.084630686	0.791827369	0.101369491
Gpr135	-0.065695421	0.791827369	0.101369491
Zfp523	0.139646364	0.791827369	0.101369491
Aplf	0.08262884	0.791827369	0.101369491
Fam43a	0.071717242	0.791827369	0.101369491
Rab11fip1	0.086443869	0.791827369	0.101369491
Tcta	-0.057125802	0.791827369	0.101369491
Pex11b	0.120052638	0.791827369	0.101369491
Scube3	0.122804875	0.791827369	0.101369491
Zfp781	-0.092656116	0.791827369	0.101369491
Zdhhc21	-0.160560182	0.791996889	0.101276524
Ubqln1	0.091458649	0.792281723	0.101120363
Fam189a2	-0.178800367	0.792342875	0.101086843
Stk25	-0.151935848	0.792342875	0.101086843
Ppp1r13l	-0.105986335	0.792342875	0.101086843
Dzip1	-0.154586254	0.792342875	0.101086843
Naa16	-0.123918803	0.792342875	0.101086843
Hcn4	0.073288612	0.792342875	0.101086843
Pnpla3	0.538015493	0.792342875	0.101086843
Cdo1	-0.068423976	0.792342875	0.101086843
Nceh1	0.068141859	0.792342875	0.101086843
Pex5	0.140250557	0.792342875	0.101086843

Wdr46	0.069075736	0.792342875	0.101086843
Timm10b	0.126545745	0.792342875	0.101086843
Kynu	0.075394042	0.792342875	0.101086843
PNMAL2	0.105073821	0.792342875	0.101086843
Atl1	0.099213948	0.792342875	0.101086843
Laptm4b	0.076374183	0.792342875	0.101086843
AI480526	-0.082553334	0.792342875	0.101086843
Ppp3ca	-0.183991106	0.792342875	0.101086843
Rufy3	-0.128459067	0.792342875	0.101086843
Panx1	0.080238169	0.792342875	0.101086843
Alpk1	0.086360343	0.792342875	0.101086843
Epha6	-0.815345975	0.792342875	0.101086843
Snrnp40	0.113035479	0.792342875	0.101086843
Lpar3	0.061160645	0.792342875	0.101086843
Stxbp2	0.128688436	0.792342875	0.101086843
Smarca1	0.189014641	0.792342875	0.101086843
Sidt2	-0.112716347	0.792519021	0.100990306
Myo16	-0.107596011	0.792519021	0.100990306
Gpr88	-0.067870923	0.79258352	0.100954962
Plekhg4	0.064459526	0.79274663	0.100865596
Tacc1	0.145282655	0.79274663	0.100865596
Tcf12	-0.075171616	0.79274663	0.100865596
Tmem39a	0.114881425	0.79274663	0.100865596
Pappa	-0.165087444	0.793102094	0.100670903
Nudcd3	-0.077471026	0.793102094	0.100670903
GAREM	0.052231091	0.793114608	0.100664051
Slc4a4	-0.102849177	0.793132637	0.100654179
Gdnf	0.056028696	0.793132637	0.100654179
Zc3h6	0.081443143	0.793178819	0.100628892
Cubn	-0.049209541	0.793375418	0.10052126
Myo1a	-0.173850784	0.793867917	0.100251749
Lcmt2	0.136435184	0.793867917	0.100251749
Elavl3	-0.117438277	0.793867917	0.100251749
Npffr1	0.190220445	0.793867917	0.100251749
Efna3	0.062805574	0.793867917	0.100251749
Cpsf4l	-0.081984994	0.793867917	0.100251749
HDGFRP3	-0.082547189	0.793867917	0.100251749
Arfgef1	0.068539398	0.793867917	0.100251749
Mppe1	-0.094266326	0.793867917	0.100251749
Emp2	-0.089394158	0.793867917	0.100251749
Draxin	-0.124479185	0.793867917	0.100251749
Surf1	-0.131658018	0.793981426	0.100189657
Rpl13	0.089634538	0.793984062	0.100188215
Dnajc3	-0.108931394	0.794062742	0.100145181
Adpgk	0.088722316	0.794263291	0.100035509
Ubald1	0.164112842	0.794263291	0.100035509
Uba6	-0.046545881	0.794263291	0.100035509
Ssmem1	-0.12657548	0.794263291	0.100035509

Amh	-0.162469403	0.794275454	0.100028858
Arl8b	0.061744428	0.794275454	0.100028858
Ighmbp2	0.054100363	0.794275454	0.100028858
Nipal4	-0.11004627	0.794275454	0.100028858
Cxcr3	0.086061624	0.794275454	0.100028858
Usp25	0.098271277	0.794517024	0.099896793
Clip1	-0.135385846	0.794602313	0.099850175
Car1	0.157837443	0.794602313	0.099850175
Vps33a	0.084668438	0.794602313	0.099850175
Mtus1	0.095973439	0.794602313	0.099850175
Fxr2	-0.133849612	0.794602313	0.099850175
Kctd17	0.075210304	0.794602313	0.099850175
3-Mar	-0.05818679	0.794602313	0.099850175
Bin3	0.084356718	0.794602313	0.099850175
Ryr1	0.096948766	0.794602313	0.099850175
Aldh9a1	0.04995157	0.794602313	0.099850175
Ptp4a2	0.088900739	0.794602313	0.099850175
Foxa1	-0.091742662	0.794602313	0.099850175
Kcnj8	-0.107714144	0.794602313	0.099850175
Rtn1	0.145899391	0.794602313	0.099850175
Hmg20b	0.092787151	0.794602313	0.099850175
Nab2	-0.112415476	0.79464501	0.099826839
Rnft2	-0.081161874	0.79464501	0.099826839
TMEM246	-0.061913123	0.795185427	0.099531588
Tyw3	-0.052248947	0.795185427	0.099531588
Dpysl5	-0.11474147	0.795325377	0.09945516
Katnbl1	0.248463283	0.795374679	0.099428239
Hs3st4	-0.100661961	0.795374679	0.099428239
Trmt1	0.067968273	0.795374679	0.099428239
Trat1	-0.062101404	0.795374679	0.099428239
Sult6b2	0.112869437	0.795778146	0.099207992
Olfml1	0.071653658	0.79611121	0.099026261
Fry	-0.075457533	0.796188642	0.098984022
Mitf	-0.116732253	0.796188642	0.098984022
CCDC94	-0.134261763	0.796367558	0.09888644
Scp2	-0.067252892	0.796367558	0.09888644
Dhx34	0.364936031	0.796367558	0.09888644
Zfp280b	-0.219478256	0.796367558	0.09888644
Sys1	0.050054928	0.796367558	0.09888644
Zc3h7b	-0.149900538	0.796367558	0.09888644
Ak2	0.370022068	0.796367558	0.09888644
Rpl27a	0.081444653	0.796367558	0.09888644
Schip1	0.236176131	0.796367558	0.09888644
Epha8	0.04796604	0.796367558	0.09888644
Isl1	0.074865891	0.796367558	0.09888644
Epx	0.06281942	0.796367558	0.09888644
Adad2	-0.111032707	0.796367558	0.09888644
Gng8	0.063298436	0.796367558	0.09888644

Tmem38b	0.073640533	0.796367558	0.09888644
Pgbd1	0.075929627	0.796367558	0.09888644
Ccdc77	-0.068761053	0.796367558	0.09888644
Tbx6	0.0751285	0.796367558	0.09888644
Cdon	0.233896103	0.796367558	0.09888644
Slc2a3	0.130052765	0.796642571	0.09873649
Tecpr1	0.122473828	0.796717047	0.09869589
Aifm2	-0.072004143	0.796900791	0.098595742
Rbm24	0.119946838	0.797026327	0.098527333
Ajuba	0.221798604	0.797026327	0.098527333
Lmntd2	0.059347694	0.797442953	0.098300375
Ubxn11	-0.162103242	0.797446729	0.098298319
Zfp449	0.118163759	0.797446729	0.098298319
Trappc11	-0.158753671	0.797446729	0.098298319
Pofut1	-0.178847111	0.797446729	0.098298319
Mcmdc2	0.115018532	0.797446729	0.098298319
Adprm	-0.095254842	0.797446729	0.098298319
Yars	0.064388072	0.797446729	0.098298319
Ttc16	-0.161898025	0.797446729	0.098298319
ZUFSP	-0.056678194	0.797446729	0.098298319
Tsc1	0.081433091	0.797446729	0.098298319
Allc	0.046524172	0.797446729	0.098298319
Mdc1	0.139086174	0.797446729	0.098298319
Ppih	-0.092880397	0.797446729	0.098298319
Srr	0.112086248	0.797446729	0.098298319
Fbxw5	0.151097314	0.797446729	0.098298319
Dnal1	0.108354522	0.797446729	0.098298319
Fam216b	0.181654564	0.797446729	0.098298319
ALS2CR11	0.096797266	0.797446729	0.098298319
Ddost	0.051627363	0.797446729	0.098298319
Clasp1	0.109895716	0.797446729	0.098298319
Lrtm2	0.064534776	0.797446729	0.098298319
Atp1b2	0.071223836	0.797446729	0.098298319
Adgrg6	-0.109836033	0.797635712	0.09819541
Ndufa10	0.22841965	0.797635712	0.09819541
Rasl11b	-0.101000659	0.797635712	0.09819541
Zfp467	0.127527591	0.798403233	0.097777713
Ptprv	0.165689576	0.798403233	0.097777713
Fance	-0.170449546	0.798403233	0.097777713
Mettl2	0.081879844	0.798403233	0.097777713
Fam216a	0.102233782	0.798403233	0.097777713
Hpcal1	0.067360117	0.798403233	0.097777713
Pik3r6	-0.068485639	0.798403233	0.097777713
Tmed2	-0.106791072	0.798403233	0.097777713
Immp2l	0.074728415	0.79855472	0.097695319
Sugt1	-0.053864143	0.79855472	0.097695319
Lsmem1	0.065607415	0.798614352	0.097662889
Tmem229	-0.316492621	0.798651855	0.097642495

Cep19	-0.18981239	0.798651855	0.097642495
Rprd1a	-0.189621544	0.798651855	0.097642495
Psenen	-0.169719509	0.798651855	0.097642495
Med22	-0.23085934	0.798651855	0.097642495
Hcn2	-0.159411319	0.798651855	0.097642495
Ints7	0.328343777	0.798651855	0.097642495
RNF219	0.171027962	0.798651855	0.097642495
Zfp503	-0.136675528	0.798651855	0.097642495
Igip	0.152058148	0.798651855	0.097642495
Cntn1	0.174178712	0.798651855	0.097642495
Rhob	0.092016888	0.798651855	0.097642495
Gcc2	0.102313885	0.798651855	0.097642495
Slc35e2	-0.081726237	0.798651855	0.097642495
Sgcb	0.208931608	0.798651855	0.097642495
Ccer1	-0.105983082	0.798651855	0.097642495
Smim11	0.075021244	0.798651855	0.097642495
Cln6	-0.076979661	0.798651855	0.097642495
Polrmt	-0.082652141	0.798651855	0.097642495
Dock6	-0.173698898	0.798651855	0.097642495
Mrps5	0.099175451	0.798651855	0.097642495
Slc6a13	0.274426379	0.798651855	0.097642495
Nfe2l1	-0.074860351	0.798651855	0.097642495
Sec11c	0.074798388	0.798651855	0.097642495
Fez1	0.067780855	0.798651855	0.097642495
Ccar2	0.109244305	0.798651855	0.097642495
Rasip1	0.28593014	0.798651855	0.097642495
Gabrb1	-0.060891729	0.798651855	0.097642495
Col24a1	-0.070021484	0.798651855	0.097642495
Phf1	0.059925957	0.798651855	0.097642495
HIST2H3B	-0.139028548	0.798651855	0.097642495
CXX1C	0.172249858	0.798651855	0.097642495
Tfap2c	0.071151585	0.798651855	0.097642495
Tmem191	0.121986769	0.798651855	0.097642495
Hapln3	0.058726902	0.798651855	0.097642495
Pcdh11x	-0.081771969	0.798651855	0.097642495
Mypn	-0.061493334	0.798651855	0.097642495
Apom	-0.061061556	0.798651855	0.097642495
Bphl	-0.103462971	0.798651855	0.097642495
Krt77	0.086497686	0.798651855	0.097642495
Npb	0.065556278	0.798651855	0.097642495
Slc5a1	-0.093264824	0.798651855	0.097642495
Slc25a26	0.084824678	0.798651855	0.097642495
Foxn3	0.161405269	0.798651855	0.097642495
Rnf151	0.066037151	0.798651855	0.097642495
Tor2a	0.119364285	0.798651855	0.097642495
Furin	0.060072974	0.798651855	0.097642495
Map4k3	-0.063007211	0.798651855	0.097642495
Hnrnpab	-0.083893038	0.798651855	0.097642495

Wbp1	0.061857217	0.798651855	0.097642495
Gdap2	0.101723073	0.798651855	0.097642495
Fzd3	0.081218223	0.798651855	0.097642495
Tenm1	0.124810397	0.798651855	0.097642495
Crhbp	-0.084227519	0.798651855	0.097642495
Tmem97	0.130969708	0.798651855	0.097642495
Fam20b	0.075208195	0.798651855	0.097642495
Zfp398	0.085825484	0.798651855	0.097642495
Crtam	0.067271215	0.798651855	0.097642495
Phactr1	-0.057490304	0.798651855	0.097642495
Kif26a	-0.066041952	0.798651855	0.097642495
Klhl22	0.095442873	0.798651855	0.097642495
Ddn	-0.078503257	0.798651855	0.097642495
Tctn2	0.138209108	0.798651855	0.097642495
Olfr1511	0.074601953	0.798651855	0.097642495
WDR34	0.087917738	0.798651855	0.097642495
Tspyl3	-0.057990054	0.798651855	0.097642495
Sh3bp5l	0.134821219	0.798651855	0.097642495
HIST1H3C	0.071280514	0.798651855	0.097642495
Zfp106	-0.072800169	0.798651855	0.097642495
Ndufaf6	0.063305275	0.798651855	0.097642495
Usp11	-0.064034102	0.798651855	0.097642495
Rpl23	0.08838289	0.798651855	0.097642495
Proca1	0.093769755	0.798651855	0.097642495
Gabrg3	0.051898223	0.798651855	0.097642495
Peli2	0.047519555	0.798651855	0.097642495
Tbc1d25	-0.094191252	0.798651855	0.097642495
Plec	0.125318294	0.798651855	0.097642495
Acsl4	0.072106658	0.798651855	0.097642495
Arhgef3	0.111332732	0.798651855	0.097642495
Zfp551	0.116739399	0.798651855	0.097642495
Lipe	-0.082176722	0.798651855	0.097642495
Pigg	0.087791753	0.798651855	0.097642495
Zfp709	-0.095699949	0.798651855	0.097642495
Cbfa2t2	0.070640071	0.798651855	0.097642495
Sh2d1a	0.078499024	0.798651855	0.097642495
Sesn2	-0.049626431	0.798651855	0.097642495
Fgf18	-0.102942791	0.798651855	0.097642495
Mef2b	-0.122553476	0.798651855	0.097642495
Sympk	0.064086188	0.798651855	0.097642495
Trim26	0.052123901	0.798651855	0.097642495
Dio3os	0.098750982	0.798725796	0.097602289
Zdhhc7	-0.073428201	0.798725796	0.097602289
Zc3h15	0.112647433	0.798725796	0.097602289
Ddx19b	-0.086228881	0.799002015	0.097452125
Rpl39	-0.089074654	0.799002015	0.097452125
Smad7	-0.07442618	0.799002015	0.097452125
Ak4	0.143459373	0.799057356	0.097422046

Gnb4	0.239308572	0.799560805	0.097148503
Mtfr1	-0.163918983	0.799560805	0.097148503
App	-0.072306362	0.799560805	0.097148503
Lrrc74b	0.205719369	0.799560805	0.097148503
Spata21	0.128708843	0.799560805	0.097148503
Exoc7	0.137797991	0.799560805	0.097148503
ZNRD1AS	-0.15803069	0.799560805	0.097148503
Eml6	-0.126551	0.799560805	0.097148503
Tchh	-0.117031798	0.799560805	0.097148503
Gpr85	0.091380142	0.799560805	0.097148503
Fbxw10	-0.143519119	0.799560805	0.097148503
Slc52a3	-0.147821764	0.799560805	0.097148503
Rfng	0.073483377	0.799560805	0.097148503
Fam8a1	0.102713824	0.799560805	0.097148503
Rprd2	0.063432508	0.799560805	0.097148503
C2cd4a	0.116815742	0.799560805	0.097148503
Scrn3	0.07752928	0.799560805	0.097148503
Wnt16	-0.067074046	0.799560805	0.097148503
Iba57	-0.099131343	0.799560805	0.097148503
Ccnjl	0.078977111	0.799560805	0.097148503
Lgals2	-0.071182938	0.799560805	0.097148503
Rdx	0.061695629	0.799560805	0.097148503
Tssk6	0.072692927	0.799560805	0.097148503
Ankrd63	0.129637102	0.799560805	0.097148503
Pfn2	-0.097420103	0.799560805	0.097148503
Nek7	0.073592892	0.799560805	0.097148503
Cryab	-0.085528475	0.799560805	0.097148503
Npffr2	0.068386139	0.799560805	0.097148503
Caps2	0.079088452	0.799560805	0.097148503
Ids	0.167464439	0.799560805	0.097148503
Dnmbp	0.101851597	0.799560805	0.097148503
Zfp219	0.054428473	0.799560805	0.097148503
DDX26B	-0.050950922	0.799560805	0.097148503
Lclat1	0.128773981	0.799560805	0.097148503
Unc5a	0.132501437	0.799560805	0.097148503
Nufip2	0.077663807	0.799560805	0.097148503
Nsun4	0.077452025	0.799560805	0.097148503
Gpr137	0.190794629	0.799560805	0.097148503
Tusc3	0.071773798	0.799560805	0.097148503
Fgfr3	0.070833118	0.799560805	0.097148503
Ensa	-0.091282902	0.799560805	0.097148503
Tmem26	0.143906784	0.799560805	0.097148503
BC051226	0.049003944	0.799560805	0.097148503
Szt2	0.082947912	0.799560805	0.097148503
FAM208B	0.127763952	0.799560805	0.097148503
Nedd4l	-0.043133872	0.799560805	0.097148503
Ccdc127	-0.052598934	0.799560805	0.097148503
Sc5d	0.047085614	0.799560805	0.097148503

Nphp4	0.134060584	0.799943488	0.096940693
Tesk2	-0.128732403	0.800132914	0.096837864
Tmem150	-0.168168395	0.80025913	0.096769362
Rpusd3	-0.122931703	0.800268284	0.096764394
Timp4	0.161845744	0.800285146	0.096755244
Elmod1	0.192823646	0.800446173	0.096667868
Ankrd66	0.087706115	0.800446173	0.096667868
Nlgn2	-0.101556985	0.800446173	0.096667868
Gmpr2	0.106774209	0.800446173	0.096667868
Sulf2	-0.123537691	0.800446173	0.096667868
Pcdha5	0.074774168	0.800446173	0.096667868
Tmem116	0.065482318	0.800446173	0.096667868
Serinc4	0.054018818	0.800446173	0.096667868
Emc4	-0.224729653	0.800804313	0.096473596
Pcdhb16	-0.119892331	0.800804313	0.096473596
Pde8a	-0.102934258	0.800804313	0.096473596
Timm10	0.109268824	0.800804313	0.096473596
Hebp1	0.11961582	0.800804313	0.096473596
Ltb4r2	-0.056546251	0.800804313	0.096473596
Chga	-0.062719459	0.800804313	0.096473596
Mff	0.062077869	0.800804313	0.096473596
Rpsa	-0.21892506	0.800804313	0.096473596
Tm4sf5	0.084005634	0.800804313	0.096473596
Kcng4	-0.118507297	0.800804313	0.096473596
Sema3c	-0.076095929	0.800804313	0.096473596
Kmt2c	0.060431933	0.800804313	0.096473596
Stk39	0.098380148	0.800804313	0.096473596
Wnt8b	-0.083455164	0.800804313	0.096473596
Ubap1l	-0.178206345	0.800856033	0.096445548
Ciao1	0.095789179	0.800856033	0.096445548
SELO	-0.16805596	0.800856033	0.096445548
Jag2	-0.07494412	0.800856033	0.096445548
Snhg4	-0.083273548	0.800856033	0.096445548
Tro	-0.056088712	0.800856033	0.096445548
Il34	0.362395149	0.800958644	0.096389907
TMEM2	-0.116583723	0.800958644	0.096389907
Ubox5	0.087398048	0.800958644	0.096389907
Mthfr	0.163726304	0.800958644	0.096389907
Hibadh	0.157142327	0.800958644	0.096389907
Masp1	0.093647719	0.800958644	0.096389907
Pdss1	0.086324232	0.800958644	0.096389907
Oma1	-0.063693947	0.800958644	0.096389907
Enah	0.109609435	0.800958644	0.096389907
Gata6	0.149280953	0.800958644	0.096389907
Hax1	-0.083647707	0.800958644	0.096389907
Leng9	-0.048721054	0.800958644	0.096389907
Prpf8	0.11537344	0.801163799	0.096278683
Zmynd19	-0.138648332	0.801163799	0.096278683

Pou4f1	0.074177251	0.801163799	0.096278683
Taf7l	0.10389434	0.801163799	0.096278683
Rnf138	-0.074569651	0.801163799	0.096278683
Sgms1	-0.0770774	0.801163799	0.096278683
Olfr316	0.154722099	0.801163799	0.096278683
Dus3l	-0.059520608	0.801214132	0.096251399
BC048546	0.176566555	0.801403051	0.096149009
Olfr559	-0.064724262	0.801403051	0.096149009
Ubald2	0.172748172	0.801403051	0.096149009
Tbx21	-0.066132636	0.801465261	0.096115297
Ttll8	-0.088627737	0.801522059	0.096084521
Atp6ap1l	-0.166464025	0.801522059	0.096084521
Entpd6	-0.063865444	0.801767071	0.095951784
Clcn6	0.142159477	0.801964808	0.095844689
Prrt1	0.103194242	0.802010337	0.095820034
Gpc5	-0.115727675	0.802271378	0.095678702
Mks1	0.071536383	0.802271378	0.095678702
Prss23	-0.057247397	0.802271378	0.095678702
Usp2	0.055455056	0.802271378	0.095678702
Tbce	0.05857819	0.802271378	0.095678702
Zfp868	-0.106198621	0.802271378	0.095678702
Cc2d1b	-0.04386677	0.802271378	0.095678702
Minpp1	-0.225252914	0.802320265	0.095652238
Efcab2	-0.27516072	0.802320265	0.095652238
Itga11	0.060748787	0.802320265	0.095652238
S100g	-0.119502031	0.802320265	0.095652238
Pcyt1a	-0.105036194	0.802432784	0.095591336
Defb25	0.139426761	0.80258509	0.095508913
Mterf1b	0.123394493	0.80258509	0.095508913
Eed	0.081226146	0.80258509	0.095508913
Usp35	0.307574154	0.80258509	0.095508913
Chchd5	-0.181410943	0.80258509	0.095508913
Eif4h	-0.119299617	0.80258509	0.095508913
Plcxd3	-0.065954996	0.80258509	0.095508913
Nvl	0.07157738	0.80258509	0.095508913
Pilrb1	0.185851615	0.80258509	0.095508913
Fam151b	0.095535706	0.80258509	0.095508913
Tnfrsf18	0.084475698	0.80258509	0.095508913
Cst11	-0.157578406	0.80258509	0.095508913
Sned1	-0.157578406	0.80258509	0.095508913
Slc30a1	0.061495638	0.80258509	0.095508913
Npas1	0.135068854	0.80258509	0.095508913
Tigar	0.092450938	0.80258509	0.095508913
Adamts2	-0.075152552	0.80258509	0.095508913
Dcp2	0.136301917	0.80258509	0.095508913
Stum	-0.059647087	0.80258509	0.095508913
Cldn1	0.196235167	0.80258509	0.095508913
Stk33	-0.072424726	0.80258509	0.095508913

Tmem19	0.056162393	0.80258509	0.095508913
Greb1l	0.075256452	0.80258509	0.095508913
Bsph1	0.079466022	0.80258509	0.095508913
Nrde2	-0.055569961	0.80258509	0.095508913
Dph7	0.130360422	0.80258509	0.095508913
Clcn2	0.056897127	0.80258509	0.095508913
Rpusd4	0.120210441	0.80258509	0.095508913
Ankrd29	0.069314557	0.80258509	0.095508913
AW04773(	-0.072029337	0.802698307	0.095447653
Gpr83	-0.099745951	0.802998713	0.095285151
Car6	0.061888335	0.802998713	0.095285151
Il23a	-0.156668772	0.803137211	0.095210252
Abhd6	0.053971175	0.803137211	0.095210252
Mtm1	0.090820216	0.803137211	0.095210252
Ltbp3	0.066575611	0.803204229	0.095174014
Ackr4	0.124074733	0.803381294	0.095078285
Hsp90ab1	-0.162284321	0.804183021	0.094645101
Sfrp1	0.117784439	0.804918418	0.094248135
Cpn1	-0.41043687	0.805218215	0.09408641
Sp9	-0.160378079	0.805218215	0.09408641
Ppa1	0.089717432	0.805218215	0.09408641
Slc25a43	0.083833155	0.805537313	0.093914338
Klrc1	0.084194526	0.805537313	0.093914338
Azin1	0.08712756	0.805537313	0.093914338
Elp3	0.104980066	0.805537313	0.093914338
Kbtbd7	0.096559284	0.805537313	0.093914338
Tapbpl	-0.143025716	0.805537313	0.093914338
Gdf10	0.060257426	0.805537313	0.093914338
Zfp354b	0.066591454	0.805537313	0.093914338
Slc25a32	0.111531416	0.805537313	0.093914338
Vps13d	0.070130212	0.805537313	0.093914338
Agl	-0.124815122	0.805537313	0.093914338
Gpd1l	0.119570152	0.805537313	0.093914338
Tbc1d17	0.055832082	0.805537313	0.093914338
Cct7	-0.07288528	0.805537313	0.093914338
Spata20	0.125356087	0.805537313	0.093914338
Ptchd4	0.060336958	0.805537313	0.093914338
Polr2d	-0.062594618	0.805537313	0.093914338
Crocc	0.072582625	0.805537313	0.093914338
Fem1b	-0.048655134	0.805537313	0.093914338
Spata18	-0.058449235	0.805537313	0.093914338
Jakmip1	0.052257812	0.805537313	0.093914338
Tmub2	0.100610553	0.805537313	0.093914338
Gltpd2	0.144173889	0.805537313	0.093914338
Ndufs1	0.139745066	0.805537313	0.093914338
Wdr45	-0.080178094	0.805928686	0.093703386
Rasd1	0.050637539	0.806182326	0.093566727
Emsy	0.078571017	0.806590571	0.093346859

Zfp595	-0.176485167	0.806590571	0.093346859
Ap2m1	-0.074713516	0.806590571	0.093346859
Cgnl1	0.075538347	0.806590571	0.093346859
Gabrb3	-0.064899271	0.806599871	0.093341852
Cerk	-0.177561943	0.806621683	0.093330108
Serpina3f	0.065780948	0.806621683	0.093330108
Dynlt1c	-0.285253843	0.806858885	0.093202414
Ccp110	0.090257526	0.806858885	0.093202414
Ccdc159	0.089009459	0.806962363	0.093146721
Ppp1r13b	-0.132485295	0.80698824	0.093132794
Dnajb1	0.058408507	0.807229416	0.093003021
Hk1	-0.110852456	0.807229416	0.093003021
Commd6	-0.111423319	0.807229416	0.093003021
Sh3bp4	0.16256632	0.807412633	0.09290446
Chd9	0.168025619	0.807412633	0.09290446
Ranbp3l	0.071401063	0.807412633	0.09290446
Ube2w	1.009678964	0.807412633	0.09290446
Nrxn3	-0.059387203	0.807412633	0.09290446
Tcte2	-0.270488571	0.807458234	0.092879932
Kcng2	0.06786946	0.80753439	0.092838974
Klc1	0.070993778	0.80753439	0.092838974
Scarf1	0.10014188	0.807788653	0.092702252
Nlrp10	0.108614722	0.807840829	0.092674201
Dennd4b	-0.090516452	0.808212181	0.092474608
Nkpd1	0.081689031	0.808212181	0.092474608
Csdc2	0.090977108	0.808212181	0.092474608
Mill2	0.141233601	0.808212181	0.092474608
Tipin	0.102457285	0.808212181	0.092474608
Aaas	-0.086298893	0.808212181	0.092474608
Pigp	0.129189387	0.808212181	0.092474608
Meox1	-0.116783921	0.808212181	0.092474608
Zfp458	-0.042913217	0.808212181	0.092474608
Ripply2	-0.109752866	0.808212181	0.092474608
Wnt10b	0.128498759	0.808212181	0.092474608
Fam204a	-0.074639411	0.808425071	0.092360227
Zfp37	-0.087709462	0.808638545	0.092245561
Ovca2	-0.25780388	0.808684388	0.092220941
Grhl1	-0.243432592	0.808684388	0.092220941
Dennd4c	0.148991334	0.808684388	0.092220941
Lmbr1l	0.126245949	0.808684388	0.092220941
Lrrc58	0.071609911	0.808684388	0.092220941
Serinc5	0.06169057	0.808684388	0.092220941
Spag9	0.049525718	0.808684388	0.092220941
Inpp5b	-0.106439123	0.808684388	0.092220941
Micu3	-0.051015229	0.808684388	0.092220941
Bbs9	-0.072808628	0.808684388	0.092220941
Jchain	0.07064422	0.808684388	0.092220941
Wsb2	0.111723376	0.808684388	0.092220941

Prdm15	0.116908496	0.808784584	0.092167135
Kiss1r	0.099364494	0.808785334	0.092166733
Ak3	0.114631295	0.808859841	0.092126726
Cand1	0.199626501	0.808859841	0.092126726
Kit	0.137688599	0.808863693	0.092124658
Tln2	0.162438624	0.808863693	0.092124658
Npm1	-0.079729901	0.808863693	0.092124658
Rpl23a	-0.096597177	0.808863693	0.092124658
Zfp141	-0.054668397	0.808863693	0.092124658
Prkcsh	-0.134973504	0.808944924	0.092081046
Slit1	0.069051398	0.808944924	0.092081046
Pnpla2	-0.087181188	0.809094753	0.092000615
Phactr3	0.091143217	0.809094753	0.092000615
Cisd1	0.147794756	0.809094753	0.092000615
Vegfa	0.15812394	0.809094753	0.092000615
Pik3r3	-0.263211155	0.809094753	0.092000615
Arf4	0.088938262	0.809094753	0.092000615
Malt1	0.164208675	0.809094753	0.092000615
Cyp2j8	-0.166853801	0.809094753	0.092000615
Aldh7a1	-0.051515816	0.809094753	0.092000615
Oscp1	-0.116643942	0.809247538	0.091918613
Klhl38	0.239233256	0.809483934	0.091791767
Snx32	0.072873127	0.80971181	0.091669526
Cd300lg	0.104112121	0.809716465	0.091667029
Ccdc91	-0.101469541	0.809716465	0.091667029
Pomt1	-0.067401583	0.809716465	0.091667029
Sycp3	0.080074732	0.809716465	0.091667029
Mhrt	-0.168022173	0.809716465	0.091667029
Mtor	-0.067739386	0.809761567	0.091642839
Mlh3	-0.089578291	0.809761567	0.091642839
Tspan31	0.120972026	0.809761567	0.091642839
Egr4	0.157502839	0.809778408	0.091633808
Trim7	0.07896558	0.809778408	0.091633808
Htatip2	0.106568042	0.809778408	0.091633808
G3bp2	-0.077545145	0.809778408	0.091633808
Snx13	0.065824667	0.809778408	0.091633808
Rpe	0.099196774	0.809778408	0.091633808
Tmem50a	0.064637642	0.809778408	0.091633808
Erc2	-0.115701434	0.809778408	0.091633808
Xpnpep3	-0.084501865	0.809778408	0.091633808
Col17a1	0.060401345	0.809778408	0.091633808
Phykpl	0.096910329	0.809778408	0.091633808
Fndc3b	0.062219949	0.809778408	0.091633808
Egln3	-0.055056143	0.809778408	0.091633808
Rad21l	0.07159104	0.809778408	0.091633808
D6Wsu16[illegible]	-0.102425141	0.809778408	0.091633808
Dmxl1	-0.168050728	0.809778408	0.091633808
Pcdhb21	-0.062107787	0.809778408	0.091633808

Dact1	-0.048340678	0.809778408	0.091633808
LECT1	0.104658271	0.809778408	0.091633808
Fbxo48	-0.141107455	0.809778408	0.091633808
Zc2hc1c	0.063802346	0.809778408	0.091633808
Lama5	-0.079394487	0.809778408	0.091633808
BC05140٤	0.07487756	0.809778408	0.091633808
Flt4	-0.104022936	0.809778408	0.091633808
Mest	0.212045578	0.809778408	0.091633808
Tarm1	-0.068467652	0.809778408	0.091633808
Abhd17a	0.057084508	0.809801895	0.091621211
Polk	-0.142785765	0.810048129	0.091489177
Ndfip2	0.0644478	0.810048129	0.091489177
Ndfip1	-0.049176151	0.810400004	0.091300565
Ap2b1	0.068702526	0.810411238	0.091294545
Ghitm	0.096374899	0.81065202	0.091165531
Tmem222	0.090679123	0.81065202	0.091165531
Ndufa2	0.057324348	0.81065202	0.091165531
Prph	-0.123556692	0.810660098	0.091161203
Epha1	-0.070680323	0.810660098	0.091161203
Gpn2	-0.074727311	0.810660098	0.091161203
Apol7b	-0.045964167	0.810660098	0.091161203
Pld3	-0.086315794	0.810660098	0.091161203
ZAK	0.136220433	0.810660098	0.091161203
Fut10	-0.069073654	0.810660098	0.091161203
Alkbh2	-0.082312784	0.810660098	0.091161203
WHSC1	0.110521331	0.810825432	0.091072638
Zfp651	-0.083122438	0.810825432	0.091072638
Pds5b	0.061937795	0.810825432	0.091072638
Smad3	-0.146045339	0.810830021	0.09107018
Rasl10b	-0.066626851	0.810830021	0.09107018
Nfia	0.12888555	0.810830021	0.09107018
Ehhadh	0.077564707	0.810986591	0.090986326
Cdkl1	0.0864563	0.810986591	0.090986326
GLTSCR1L	0.152480847	0.810986591	0.090986326
Spire1	0.11135769	0.810986591	0.090986326
Nkain1	-0.050521456	0.811052083	0.090951256
Trim3	-0.069161079	0.811072113	0.090940531
Zfp770	-0.053485135	0.811166501	0.090889993
Fbll1	-0.082878795	0.81117961	0.090882974
Yif1a	-0.080281071	0.81117961	0.090882974
Znrf2	-0.046270915	0.81117961	0.090882974
F2rl3	-0.074567178	0.811494589	0.090714372
Mroh6	0.159358676	0.811653168	0.090629512
Fetub	-0.169846831	0.811653168	0.090629512
Arid3a	0.069585314	0.811653168	0.090629512
Rpl15	0.126001971	0.812629891	0.090107207
Fam120b	0.089299315	0.812629891	0.090107207
Mthfd2	-0.109968024	0.812629891	0.090107207

Rpl36a	0.082405299	0.812742514	0.090047022
Pak3	-0.211753308	0.81330105	0.089748667
Xylt1	0.101309891	0.813515916	0.089633946
Fam185a	0.107129973	0.813621227	0.08957773
Nyap1	-0.191608489	0.813824578	0.089469198
Atp6v1a	-0.065804766	0.813824578	0.089469198
Lhx2	-0.106449207	0.813824578	0.089469198
Prim1	0.062072225	0.813824578	0.089469198
Mcam	0.106831303	0.813824578	0.089469198
Rnf113a1	-0.174390841	0.81413124	0.08930558
TMEM35	-0.230176602	0.81413124	0.08930558
Zfat	-0.236060394	0.81413124	0.08930558
Dym	0.122827211	0.81413124	0.08930558
PROSC	0.177160003	0.81413124	0.08930558
Zfp438	-0.105705682	0.81413124	0.08930558
Dao	0.201850384	0.81413124	0.08930558
Tmx4	-0.136724049	0.81413124	0.08930558
Pdzd4	-0.08271483	0.81413124	0.08930558
Ascl3	0.059636089	0.81413124	0.08930558
Fbxl8	0.060680717	0.81413124	0.08930558
Pdia3	0.056135388	0.81413124	0.08930558
Etnk1	0.075940492	0.81413124	0.08930558
Tnik	-0.069586002	0.81413124	0.08930558
Fam221b	-0.085002788	0.81413124	0.08930558
Bicd1	0.11412908	0.81413124	0.08930558
Nek9	0.068382023	0.81413124	0.08930558
Shpk	-0.091816774	0.81413124	0.08930558
Als2	0.091009439	0.81413124	0.08930558
Limch1	0.095747335	0.81413124	0.08930558
Zfp653	0.104139196	0.81413124	0.08930558
Col20a1	-0.043821432	0.81413124	0.08930558
Alkbh4	0.096557271	0.81413124	0.08930558
Zfp318	0.076424862	0.81413124	0.08930558
Rgs12	-0.072331376	0.81413124	0.08930558
Sdk1	0.069105254	0.81413124	0.08930558
Rsrp1	-0.096369182	0.81413124	0.08930558
B3galt1	0.096234036	0.814387071	0.08916913
Samd12	-0.065533338	0.814546432	0.089084154
Tprkb	-0.274689913	0.814546432	0.089084154
Sucla2	-0.072885956	0.814546432	0.089084154
Zmym2	-0.069720875	0.814793872	0.088952246
Slc25a22	-0.053352995	0.814793872	0.088952246
Hnrnph3	-0.222419344	0.815046559	0.088817582
Zfp87	-0.074112217	0.815046559	0.088817582
Rabggta	-0.095842006	0.815046559	0.088817582
Ap1b1	0.100325324	0.815046559	0.088817582
Nlgn1	0.064560127	0.815046559	0.088817582
Tceal6	0.041529153	0.815046559	0.088817582

Lgals12	-0.159543899	0.815083424	0.088797939
Camk2b	0.048211069	0.81512807	0.088774151
Gpr15	-0.060705543	0.81512807	0.088774151
GRRP1	-0.079063629	0.81517119	0.088751178
Cdc42	-0.080831129	0.81517119	0.088751178
Uba52	-0.08063195	0.81517119	0.088751178
Rgs11	-0.0791301	0.81517119	0.088751178
Cd2ap	-0.059362214	0.81517119	0.088751178
Tmem159	0.07695307	0.81517119	0.088751178
Zfhx2	-0.055297813	0.81517119	0.088751178
Nptxr	0.083352776	0.81517119	0.088751178
Agbl2	-0.05203643	0.81517119	0.088751178
Dlx4	-0.052710642	0.81517119	0.088751178
Mad1l1	-0.071632376	0.81517119	0.088751178
Gpr89	0.100457646	0.81517119	0.088751178
Lysmd4	-0.120083058	0.815377423	0.088641318
Rmdn3	0.072384982	0.815693746	0.088472868
Kctd15	0.107022707	0.815693746	0.088472868
Alg11	0.134579047	0.815758644	0.088438316
Hdac11	-0.067119488	0.815874464	0.08837666
Atp5k	0.076767692	0.815930357	0.088346908
HIST1H1A	-0.099192964	0.815930357	0.088346908
Raet1e	-0.099150805	0.815930357	0.088346908
Cblc	0.057460775	0.815930357	0.088346908
Srrm2	-0.149682629	0.815930357	0.088346908
Dhx40	0.075282426	0.816399676	0.088097176
Rnf17	-0.073112824	0.816399676	0.088097176
Rpain	-0.092970622	0.816622915	0.087978437
Gle1	-0.064411671	0.816974012	0.087791758
Qtrt1	0.161011953	0.816974012	0.087791758
Adra2b	-0.204991827	0.81730296	0.087616928
TSTA3	-0.306408194	0.81730296	0.087616928
Irx2	0.182835691	0.81730296	0.087616928
Tmem179	-0.29786918	0.81730296	0.087616928
Hip1	-0.118280049	0.81730296	0.087616928
Mboat7	-0.072148177	0.81730296	0.087616928
Bmp6	-0.129377434	0.81730296	0.087616928
Ccdc42	-0.112069298	0.81730296	0.087616928
Mapk13	-0.095469247	0.81730296	0.087616928
Msto1	0.100760402	0.81730296	0.087616928
Arhgdia	0.093111273	0.81730296	0.087616928
Pcgf5	-0.108151398	0.81730296	0.087616928
Ppp4c	0.084130429	0.81730296	0.087616928
Kansl3	0.286183507	0.81730296	0.087616928
Fam222a	-0.173462486	0.81730296	0.087616928
Pkd1l2	0.058940574	0.81730296	0.087616928
Pcdhga4	0.088767714	0.81730296	0.087616928
Cmtm5	0.09227129	0.81730296	0.087616928

Ttc17	0.088602561	0.81730296	0.087616928
Lysmd3	0.085038559	0.81730296	0.087616928
Ddx49	0.125103174	0.81730296	0.087616928
Abhd3	0.061158781	0.81730296	0.087616928
Mrps27	0.10276646	0.81730296	0.087616928
Arl4aos	-0.166451249	0.81730296	0.087616928
Rbm33	0.087334666	0.81730296	0.087616928
Pcdh15	0.053384371	0.81730296	0.087616928
Ruvbl1	-0.131837833	0.81730296	0.087616928
Scarb1	0.082309818	0.81730296	0.087616928
Ccdc181	-0.086964946	0.81730296	0.087616928
St8sia3	-0.090901412	0.81730296	0.087616928
Tspan18	0.09510046	0.81730296	0.087616928
Pom121	0.076062403	0.81730296	0.087616928
Nme7	-0.091311833	0.81730296	0.087616928
Cryzl1	0.094556417	0.81730296	0.087616928
Etaa1	0.134218304	0.81730296	0.087616928
Gphn	0.076363663	0.81730296	0.087616928
Hgsnat	-0.090843356	0.81730296	0.087616928
Gdf9	0.138239633	0.81730296	0.087616928
Sgcz	0.059237539	0.81730296	0.087616928
Rasal1	-0.049839696	0.81730296	0.087616928
Treml4	-0.089671189	0.81730296	0.087616928
Cycs	0.055820734	0.81730296	0.087616928
Cd2	0.094527598	0.81730296	0.087616928
Tulp3	0.161272005	0.81730296	0.087616928
Mthfsl	0.227651514	0.81730296	0.087616928
Myo1d	0.058155794	0.81730296	0.087616928
Bet1	-0.04934885	0.81730296	0.087616928
Pum2	0.10190402	0.81730296	0.087616928
Hbp1	0.068961071	0.81730296	0.087616928
Comtd1	-0.053510139	0.81730296	0.087616928
Gpr12	0.069122571	0.81730296	0.087616928
Abcb6	-0.094730311	0.81730296	0.087616928
Zbtb7c	-0.053560752	0.81730296	0.087616928
Zbtb20	-0.092440155	0.81730296	0.087616928
Rtn4rl1	0.083088401	0.81730296	0.087616928
AGPAT9	0.050795856	0.81730296	0.087616928
Pno1	0.101442409	0.81730296	0.087616928
Fbrsl1	0.066064659	0.81730296	0.087616928
Taf9b	0.06569157	0.81730296	0.087616928
Axin2	0.127789056	0.81730296	0.087616928
Ddit3	-0.046362345	0.81730296	0.087616928
Rab27b	0.148035255	0.81730296	0.087616928
Zxdb	-0.044402033	0.81730296	0.087616928
Pcdhb10	0.074760521	0.81730296	0.087616928
Arhgap10	-0.065183961	0.81730296	0.087616928
Cdc42ep2	0.068379306	0.81730296	0.087616928

Rab43	0.08452699	0.81730296	0.087616928
Ube4bos3	0.075488951	0.81730296	0.087616928
Foxe3	0.125533449	0.81730296	0.087616928
Lrrn2	0.056037086	0.81730296	0.087616928
Zar1l	-0.082174797	0.81730296	0.087616928
Xylt2	-0.055752038	0.81730296	0.087616928
Trex2	-0.068393632	0.81730296	0.087616928
Zdhhc17	-0.096047898	0.81730296	0.087616928
Tomm40	-0.06907898	0.81730296	0.087616928
Ints9	0.097396137	0.81730296	0.087616928
Thap7	0.09249593	0.81730296	0.087616928
Pias2	0.09224218	0.81730296	0.087616928
Nup62cl	0.041048024	0.81730296	0.087616928
Prx	-0.188369057	0.817683493	0.087414769
Lrrc8d	0.088985491	0.817696832	0.087407685
Wars2	0.073313267	0.817955543	0.0872703
Nxt1	-0.108196845	0.818090312	0.08719875
Il1rl1	-0.067173545	0.818166927	0.08715808
Slc28a1	-0.174396726	0.818382784	0.087043515
Nsg1	-0.168948453	0.818382784	0.087043515
Vax1	-0.225342208	0.818382784	0.087043515
Zfp704	0.062124102	0.818382784	0.087043515
Eif4a1	0.097245455	0.818382784	0.087043515
Nt5c1a	-0.064020479	0.818382784	0.087043515
Il20rb	-0.039336012	0.818382784	0.087043515
Mkrn1	-0.078485586	0.818555016	0.086952126
Ctxn2	0.112395941	0.818736425	0.086855888
Rab8b	0.087872502	0.818909436	0.086764125
Zfp454	0.087120155	0.818952675	0.086741194
Fbxo17	-0.23010339	0.81905821	0.086685232
Rpp14	-0.215376671	0.81905821	0.086685232
Nemf	0.088462329	0.81905821	0.086685232
Plet1	0.082321669	0.81905821	0.086685232
Smim1	0.053392442	0.81905821	0.086685232
Gsc2	0.074132686	0.81905821	0.086685232
Ric3	0.078513642	0.81905821	0.086685232
Nme3	0.14363244	0.81905821	0.086685232
Ift22	0.135824888	0.819195714	0.086612329
Adss	-0.078959157	0.819195714	0.086612329
Gdi1	0.25638066	0.819195714	0.086612329
Slc25a44	-0.055851781	0.819195714	0.086612329
Pink1	0.195993116	0.819463576	0.086470346
Asic4	-0.13803451	0.819463576	0.086470346
CCDC108	-0.087049721	0.819463576	0.086470346
Baz1b	0.145071511	0.819463576	0.086470346
Meig1	0.058109154	0.819463576	0.086470346
Frmd3	0.053694508	0.819463576	0.086470346
Gpd2	-0.215032633	0.819853608	0.086263688

Afap1l1	0.215977766	0.819853608	0.086263688
Zfp605	0.0646664	0.819853608	0.086263688
Nudt22	-0.055242153	0.820699685	0.085815733
Grifin	-0.106161045	0.820809348	0.085757706
Dbn1	-0.131357945	0.821080383	0.085614324
Pcdhb17	-0.07806672	0.821138618	0.085583523
Aatk	-0.098512986	0.821138618	0.085583523
Slc13a4	-0.166840173	0.821161242	0.085571557
Serac1	-0.054652137	0.82119055	0.085556057
F8a	-0.532561151	0.821281349	0.08550804
Cdv3	-0.28406386	0.821292224	0.085502289
Iglon5	-0.068855415	0.821292224	0.085502289
Cyp19a1	0.101354298	0.821292224	0.085502289
Hspa9	-0.1615934	0.821292224	0.085502289
Slc25a12	0.262601424	0.821858839	0.08520277
Wdr37	0.120383968	0.821892951	0.085184744
Ctnnal1	0.066663724	0.822044523	0.08510466
Cep85l	0.10415609	0.822044523	0.08510466
F8	-0.052893359	0.82205776	0.085097667
Mettl1	-0.063037111	0.82205776	0.085097667
Fbxo2	0.059794889	0.82205776	0.085097667
Clps	-0.059395423	0.82205776	0.085097667
Ccdc87	-0.067959747	0.822187833	0.085028954
Abcf2	-0.072933597	0.822187833	0.085028954
Wdr93	0.054788803	0.822203399	0.085020732
Mcf2l	-0.057342316	0.822203399	0.085020732
Med24	-0.104520459	0.82269935	0.084758846
Spata9	0.110078201	0.823134178	0.084529365
Nfic	0.040124443	0.823134178	0.084529365
Amer1	-0.055946957	0.823357816	0.084411387
Ncor2	-0.115481863	0.823357816	0.084411387
Atg4b	0.11489381	0.823357816	0.084411387
Aox4	-0.08487146	0.823357816	0.084411387
Trim33	0.183960243	0.823539156	0.084315747
Kctd18	-0.165707112	0.823672205	0.084245589
Tuba1a	-0.121689283	0.823672205	0.084245589
Eif1ax	-0.16264692	0.823672205	0.084245589
Znfx1	-0.073251874	0.823672205	0.084245589
Nos1	0.224866635	0.823672205	0.084245589
Ube2b	0.076190344	0.823672205	0.084245589
Fam76b	-0.075600684	0.823672205	0.084245589
Lrp1b	0.119269434	0.823672205	0.084245589
Fam227a	0.06124631	0.823672205	0.084245589
Spats1	0.052030671	0.823672205	0.084245589
Haus6	-0.18639245	0.823672205	0.084245589
Spo11	-0.064978273	0.823672205	0.084245589
Slc6a8	-0.160883767	0.823672205	0.084245589
Slc26a5	-0.061836263	0.823672205	0.084245589

Ppp1r16b	0.088382092	0.823672205	0.084245589
Fubp3	0.083995451	0.823672205	0.084245589
Pcbp3	-0.067906689	0.823837709	0.084158333
Bmx	-0.142259565	0.823905502	0.084122597
Elovl2	-0.066087332	0.823905502	0.084122597
Gpr27	-0.0789519	0.82403951	0.084051965
Csnk1g3	-0.78144592	0.824127536	0.084005575
Wdr74	-0.112452792	0.824127536	0.084005575
Trank1	0.068287512	0.824127536	0.084005575
Lgals4	-0.232434591	0.824127536	0.084005575
Sav1	0.087162981	0.824127536	0.084005575
Klhl26	0.060429118	0.824127536	0.084005575
Eci3	0.105901187	0.824127536	0.084005575
Ppp1r16a	0.107680632	0.824127536	0.084005575
BC05101[illegible]	-0.0907864	0.824127536	0.084005575
Tma16	0.083020077	0.824127536	0.084005575
Tmem229	0.215885557	0.824150575	0.083993434
Ap3m1	-0.112820401	0.824150575	0.083993434
Fancm	-0.043474465	0.824150575	0.083993434
Pin1	0.082314919	0.824150575	0.083993434
Dnajb3	-0.090926103	0.824150575	0.083993434
Ankfy1	-0.071168891	0.824150575	0.083993434
Amhr2	0.133284189	0.824150575	0.083993434
Cbfa2t3	-0.083520693	0.824150575	0.083993434
Cfap99	0.110767038	0.824150575	0.083993434
St6galnac[illegible]	-0.126174442	0.824150575	0.083993434
Foxq1	0.082667838	0.824150575	0.083993434
Celf2	-0.287931176	0.824207298	0.083963544
Josd2	-0.077119736	0.824207298	0.083963544
Rnft1	-0.106797049	0.824207298	0.083963544
Fbxw17	-0.083727727	0.824207298	0.083963544
Dag1	0.096006272	0.824207298	0.083963544
Ferd3l	0.105021019	0.824207298	0.083963544
Mrpl32	-0.078201335	0.824207298	0.083963544
Aptx	0.109415332	0.824207298	0.083963544
Usp40	-0.077102963	0.824207298	0.083963544
Fbp2	-0.051443587	0.824207298	0.083963544
Hibch	-0.123072843	0.824207298	0.083963544
Pot1b	0.088218096	0.824438363	0.083841808
Pcdhgc5	-0.223251783	0.824798873	0.083651941
Prss41	0.099666985	0.824798873	0.083651941
Atp5e	-0.171458461	0.824798873	0.083651941
Olfr692	-0.057365868	0.824798873	0.083651941
Cd80	0.079697065	0.824798873	0.083651941
Kat7	-0.083848595	0.824798873	0.083651941
Slc25a46	-0.048030512	0.824798873	0.083651941
Ap3s2	0.097968055	0.824798873	0.083651941
Ttc30a1	-0.055981731	0.824798873	0.083651941

Frat1	0.043683646	0.824798873	0.083651941
Spag4	0.16032421	0.824798873	0.083651941
DPCR1	0.059996548	0.824798873	0.083651941
Cacna2d3	-0.048363405	0.824798873	0.083651941
Dusp23	0.080802334	0.824798873	0.083651941
Kbtbd4	-0.091799982	0.824873636	0.083612577
Cryba1	0.080564089	0.824873636	0.083612577
Rnf187	-0.061147719	0.824944745	0.08357514
Tmem239	0.049912904	0.824944745	0.08357514
Wasl	0.080447927	0.824944745	0.08357514
Diablo	-0.145769582	0.825032469	0.083528959
Mesp2	-0.06629922	0.825215605	0.083432568
Gng7	-0.124537838	0.825215605	0.083432568
Taf1	-0.176802402	0.825368347	0.083352191
Samd14	0.066629123	0.825368347	0.083352191
Tom1l1	-0.097038693	0.825368347	0.083352191
Scin	-0.104627445	0.825368347	0.083352191
St3gal2	-0.06694587	0.825368347	0.083352191
Syf2	0.118563051	0.825368347	0.083352191
Asb17	0.048561933	0.825368347	0.083352191
Stpg1	-0.079534746	0.825855192	0.083096097
Pask	-0.081260504	0.825857979	0.083094631
Rdh10	0.111725181	0.825857979	0.083094631
Cmbl	-0.089765467	0.826045398	0.082996084
Uqcrh	0.21754618	0.826216835	0.08290596
Grip1	-0.098314972	0.826237991	0.08289484
Kmt2d	0.062309357	0.82631327	0.082855273
Sez6l2	0.098966199	0.826435205	0.082791191
Foxb2	0.218136715	0.826435205	0.082791191
Leprotl1	-0.049857906	0.826435205	0.082791191
Lasp1	-0.076873936	0.826435205	0.082791191
Erh	0.078876515	0.826435205	0.082791191
Cyhr1	-0.086028008	0.82652095	0.082746134
Trim71	-0.107701341	0.827106545	0.082438543
Atp6v1h	-0.182842059	0.827106545	0.082438543
Slc25a27	0.062129667	0.827106545	0.082438543
Vash2	0.058697493	0.827106545	0.082438543
BC005561	0.078367907	0.827106545	0.082438543
Maml1	-0.092315178	0.827204907	0.082386898
Pcp4	0.081559232	0.827386517	0.082291561
Fam171a2	0.119527744	0.827386517	0.082291561
Fam71e1	-0.061712978	0.827836956	0.08205519
Dio2	-0.111067355	0.827918708	0.082012304
N4bp2l1	-0.117313528	0.827918708	0.082012304
Cabp5	-0.055760958	0.827918708	0.082012304
Ndufv1	-0.241736022	0.827977083	0.081981684
Atg4c	-0.05974607	0.828195686	0.081867036
Myod1	-0.062588051	0.828195686	0.081867036

Fgfr2	0.061554466	0.828195686	0.081867036
Cntrob	0.077577526	0.828195686	0.081867036
Serpine3	-0.274447039	0.828920672	0.08148703
L3mbtl2	-0.084837606	0.828920672	0.08148703
Fndc3c1	-0.172197475	0.828920672	0.08148703
Incenp	0.178769397	0.828920672	0.08148703
Pacs1	0.079322332	0.828920672	0.08148703
Ncam2	0.096507265	0.828920672	0.08148703
Abl1	0.123215923	0.828920672	0.08148703
Ubtf	0.068076785	0.828920672	0.08148703
Efl1	-0.101014434	0.828920672	0.08148703
Dnajc24	-0.07909938	0.828920672	0.08148703
Ppp2ca	-0.075448015	0.828920672	0.08148703
Rab42	0.100558834	0.828920672	0.08148703
Btf3l4	0.045669588	0.828920672	0.08148703
PRUNE	-0.066247682	0.828920672	0.08148703
Asic1	-0.076266857	0.828920672	0.08148703
Pdxk	0.101873793	0.828920672	0.08148703
Dhx30	0.105693245	0.828920672	0.08148703
Fkbp15	0.151501242	0.828920672	0.08148703
Uggt2	0.077186523	0.828920672	0.08148703
Gpatch2l	0.267082556	0.828920672	0.08148703
Tmprss9	0.053913205	0.828920672	0.08148703
Tspan2os	0.067900124	0.828920672	0.08148703
Itga10	0.206282205	0.828920672	0.08148703
Mc4r	-0.06639356	0.828920672	0.08148703
Cnst	0.121543519	0.828920672	0.08148703
Syt14	0.13421575	0.829015207	0.081437503
Snx4	0.075777716	0.829903823	0.080972235
Krt8	-0.073126801	0.829937233	0.080954751
Cgn	0.080244669	0.829943391	0.080951529
Cep112	0.07563436	0.829960756	0.080942442
Rnf130	-0.237677572	0.829968662	0.080938305
TCEB1	-0.384744642	0.829968662	0.080938305
Kcnh2	-0.089472863	0.829968662	0.080938305
Dcbld2	-0.109722281	0.829968662	0.080938305
Ndst4	-0.046951755	0.829968662	0.080938305
Amt	-0.132935916	0.829968662	0.080938305
Gdf5	0.07748361	0.829968662	0.080938305
Fendrr	0.040705918	0.829968662	0.080938305
Msh5	0.122308792	0.829968662	0.080938305
Rps19bp1	-0.086124637	0.829968662	0.080938305
Acot7	0.044516305	0.829968662	0.080938305
Hes5	-0.090356763	0.829968662	0.080938305
FAM19A5	0.076815566	0.829968662	0.080938305
Cgrrf1	-0.069587296	0.829968662	0.080938305
Camsap3	0.069048587	0.829968662	0.080938305
Pth2r	-0.096891167	0.829968662	0.080938305

Khdc3	-0.09696133	0.829968662	0.080938305
Zdbf2	-0.042120637	0.829968662	0.080938305
Grk6	0.086631925	0.829968662	0.080938305
Tox2	0.075058114	0.829968662	0.080938305
Camsap2	0.075054413	0.829968662	0.080938305
Cnrip1	-0.054806229	0.830288986	0.080770723
Pgm1	-0.047690855	0.830288986	0.080770723
Cstf2t	0.227766524	0.830464857	0.080678741
Cyp46a1	-0.257445076	0.830464857	0.080678741
Slc39a3	0.087682242	0.830611772	0.080601918
Asb2	0.072106429	0.830611908	0.080601847
C2cd3	0.128895091	0.830728186	0.080541054
Tmem30a	0.074217412	0.830728186	0.080541054
Ttc14	0.074208612	0.830728186	0.080541054
Smim17	0.169793764	0.830930433	0.080435335
Trim36	0.143393593	0.83101508	0.080391095
Src	0.090542533	0.83101508	0.080391095
Olfr1102	0.044493313	0.83101508	0.080391095
Ccnl2	0.092437623	0.831244191	0.080271377
D3Ertd751	-0.09070527	0.831301793	0.080241283
Slc6a1	0.187737654	0.831301793	0.080241283
Rcor3	0.154295373	0.831301793	0.080241283
Ccnl1	-0.074950574	0.831301793	0.080241283
Plppr1	-0.05046933	0.831301793	0.080241283
Myh15	-0.073397398	0.831301793	0.080241283
Mrpl50	-0.088913359	0.831301793	0.080241283
Prmt7	-0.073579303	0.831301793	0.080241283
Slamf1	0.10102459	0.831301793	0.080241283
Pomk	0.061516728	0.831484031	0.080146087
Pmaip1	0.149999041	0.831575392	0.080098371
WISP3	0.073177938	0.831994294	0.079879652
Pex6	0.063629009	0.832049345	0.079850917
Hsd17b4	0.088451507	0.832073078	0.079838529
Gars	-0.072913369	0.832417774	0.079658655
Wdr1	-0.093824984	0.832417774	0.079658655
Tcte1	-0.064209107	0.833026889	0.07934098
Lztr1	0.039678496	0.833026889	0.07934098
Dazl	0.086510606	0.833150542	0.079276519
Igf2r	0.095892771	0.833150542	0.079276519
Mark2	0.192255231	0.833381886	0.079155943
Kcnq4	0.117941557	0.833608668	0.079037778
Ambra1	-0.0624887	0.833608668	0.079037778
Sh3bgrl2	0.201211096	0.833608668	0.079037778
Gjb2	0.113116897	0.833608668	0.079037778
Igbp1	-0.175968145	0.833660157	0.079010954
Atg2b	0.060374903	0.833681828	0.078999665
Ank2	0.072158724	0.833681828	0.078999665
Dusp7	-0.062695823	0.833730318	0.078974405

Tgfbr3l	-0.065776833	0.833813926	0.078930856
FAM58B	-0.113685593	0.833970545	0.078849288
Cog6	0.098244314	0.833970545	0.078849288
Nr2c2ap	-0.090709331	0.833970545	0.078849288
Sumf1	-0.085391139	0.833970545	0.078849288
Gipc3	0.06411133	0.833970545	0.078849288
Sybu	-0.071734919	0.833970545	0.078849288
Zscan22	-0.071705657	0.833970545	0.078849288
Tmem43	0.066403341	0.833970545	0.078849288
Lrrc47	-0.169721226	0.833970545	0.078849288
Tsen15	0.083614524	0.833970545	0.078849288
Tmem177	0.153436455	0.833970545	0.078849288
Lrrc19	-0.080691279	0.834029763	0.078818451
Arih2	0.15210935	0.834029763	0.078818451
Pigo	0.118822176	0.834029763	0.078818451
Trim27	0.066417632	0.834029763	0.078818451
Man1a2	-0.064072549	0.834029763	0.078818451
Smurf2	-0.073193721	0.834029763	0.078818451
Gmps	0.081792093	0.834029763	0.078818451
Mybl1	0.06867181	0.834029763	0.078818451
Ptpn21	0.051943077	0.834029763	0.078818451
Frem2	0.069735853	0.834029763	0.078818451
Ackr3	0.075944293	0.834029763	0.078818451
Pycr2	0.058160665	0.834029763	0.078818451
Atrx	0.079682484	0.834029763	0.078818451
Gcat	0.070631573	0.834029763	0.078818451
Tnnt3	0.079964478	0.834029763	0.078818451
BC068281	-0.050540969	0.834029763	0.078818451
Ptcd1	-0.076121792	0.834564712	0.078539982
Nkx1-2	-0.073348827	0.834693815	0.078472805
LPPOS	-0.232505422	0.834870842	0.078380706
Atp13a4	-0.274312596	0.835056688	0.078284041
Txk	0.111219627	0.835056688	0.078284041
Nnmt	0.092857735	0.835672474	0.077963903
Prrc1	0.068530899	0.83580611	0.077894459
Gypa	0.049120243	0.835855533	0.077868778
Alg8	0.194135492	0.83596453	0.077812149
Dnaja4	-0.207100068	0.83631472	0.077630259
Asprv1	1.455541581	0.83631472	0.077630259
Slc35e4	0.37726538	0.83631472	0.077630259
Kdm7a	0.103294222	0.83631472	0.077630259
KIF1BP	0.102555294	0.83631472	0.077630259
Pars2	0.062618185	0.83631472	0.077630259
Myo1h	0.077309524	0.83631472	0.077630259
Hus1	0.07842001	0.83631472	0.077630259
Decr2	-0.121485915	0.83631472	0.077630259
Tas2r137	0.048001406	0.83631472	0.077630259
Mrpl35	0.079610416	0.83631472	0.077630259

Tm2d3	0.085334792	0.83631472	0.077630259
Il17b	0.075574411	0.83631472	0.077630259
Rarg	0.046203124	0.83631472	0.077630259
Lipt1	0.228772343	0.83631472	0.077630259
Cep152	-0.142567515	0.83631472	0.077630259
Nolc1	-0.055855756	0.83631472	0.077630259
Elmo1	-0.08517723	0.83631472	0.077630259
Zbtb14	0.086497727	0.83631472	0.077630259
Atp6v1e1	0.097816404	0.83631472	0.077630259
Extl2	0.103355625	0.83631472	0.077630259
Zhx3	0.200016115	0.83631472	0.077630259
Clcn1	-0.096441929	0.83631472	0.077630259
Msrb3	-0.074371019	0.83631472	0.077630259
Prok2	-0.07337008	0.83631472	0.077630259
Gpatch1	-0.070496541	0.83631472	0.077630259
Lrrc8a	0.0666697	0.83631472	0.077630259
Tnpo1	-0.063329312	0.83631472	0.077630259
Agk	0.04337886	0.836325524	0.077624649
Slc7a4	-0.114391431	0.836325524	0.077624649
Kcnd2	0.06699848	0.836325524	0.077624649
Kbtbd2	0.064341586	0.836325524	0.077624649
Spink10	0.06272034	0.836325524	0.077624649
Cxxc5	-0.199939737	0.836325524	0.077624649
Nme5	-0.038693838	0.836325524	0.077624649
Trim46	0.05669823	0.836325524	0.077624649
Cpsf6	0.510185123	0.836325524	0.077624649
B4galnt4	0.060166653	0.836325524	0.077624649
Abracl	0.06896503	0.836325524	0.077624649
Zfp30	-0.068693402	0.836342781	0.077615687
Bola3	0.094564532	0.836342781	0.077615687
Siva1	0.068725312	0.836342781	0.077615687
Nek1	-0.158219913	0.836626819	0.077468217
Wsb1	-0.144240907	0.836626819	0.077468217
Sgk2	0.090649583	0.836626819	0.077468217
Nudt18	-0.06852946	0.836626819	0.077468217
Terf1	0.173296398	0.836723085	0.077418249
Scd3	-0.06405411	0.836723085	0.077418249
Eri1	-0.12710144	0.836723085	0.077418249
Crat	-0.066748637	0.836723085	0.077418249
Ipo4	-0.083168205	0.836723085	0.077418249
Dpm3	-0.081683351	0.836723085	0.077418249
Serpinb2	-0.068640537	0.836723085	0.077418249
Ak5	-0.081393103	0.836723085	0.077418249
Rab35	0.157009289	0.836723085	0.077418249
Lmcd1	-0.065941989	0.836723085	0.077418249
Pttg1	0.061803653	0.836723085	0.077418249
Rnf125	-0.118320978	0.836723085	0.077418249
Ciita	0.073249721	0.836723085	0.077418249

Tmpo	0.088753488	0.836723085	0.077418249
AW49522:	0.063749564	0.836723085	0.077418249
Celf3	-0.068704049	0.836723085	0.077418249
Myom2	0.067926649	0.836723085	0.077418249
Tpst1	-0.129284004	0.836938741	0.077306328
Cnpy4	-0.128908821	0.836938741	0.077306328
Ttc30a2	-0.114975902	0.836938741	0.077306328
Platr4	-0.104739183	0.836938741	0.077306328
Nlrx1	-0.21399233	0.836938741	0.077306328
Sh2d6	-0.1397561	0.836938741	0.077306328
Dip2a	0.087431741	0.836938741	0.077306328
Recql5	0.071211257	0.836938741	0.077306328
Olfr324	-0.112902155	0.836938741	0.077306328
Agfg1	-0.099120002	0.836938741	0.077306328
Lss	0.07089892	0.836938741	0.077306328
Timeless	0.196786528	0.836938741	0.077306328
ADCK4	0.068964884	0.836938741	0.077306328
Vwc2l	-0.077283193	0.836938741	0.077306328
St8sia2	0.058182171	0.836938741	0.077306328
Cfap97	0.065595468	0.836938741	0.077306328
Vmp1	0.075563603	0.836938741	0.077306328
Orm2	-0.208982402	0.836938741	0.077306328
Aldh18a1	0.08283078	0.836938741	0.077306328
Rsad1	0.074864044	0.836938741	0.077306328
Adcy6	-0.221743627	0.836938741	0.077306328
Abi2	-0.098459708	0.836938741	0.077306328
Qars	-0.143018097	0.836938741	0.077306328
Wfikkn2	-0.066043636	0.836938741	0.077306328
Trappc8	-0.063255906	0.836938741	0.077306328
Paqr3	-0.130697563	0.836938741	0.077306328
B3gnt2	-0.074095482	0.836938741	0.077306328
GDAP10	0.098257958	0.836938741	0.077306328
Aph1b	-0.099631353	0.836938741	0.077306328
Lhpp	0.109545058	0.836938741	0.077306328
Rrp12	0.059587466	0.836938741	0.077306328
M6pr	-0.08811266	0.836938741	0.077306328
Prdm2	0.081937381	0.836938741	0.077306328
Ankrd50	0.073645699	0.836938741	0.077306328
Tjp1	-0.05600564	0.836938741	0.077306328
NESPAS	-0.075701755	0.836938741	0.077306328
Olfr1423	-0.067184705	0.836938741	0.077306328
Rps25	-0.066880832	0.836938741	0.077306328
Snrnp35	-0.066303911	0.836938741	0.077306328
Tmprss5	0.172988538	0.836938741	0.077306328
Bag4	0.047851593	0.836938741	0.077306328
Baiap3	0.19118167	0.836938741	0.077306328
Dusp8	0.126300394	0.836938741	0.077306328
Rnf214	0.041115139	0.836938741	0.077306328

Gpld1	-0.053095856	0.836938741	0.077306328
Hsd17b11	0.065436725	0.836938741	0.077306328
Grin1os	-0.097771526	0.836938741	0.077306328
Bcan	0.075434229	0.836938741	0.077306328
Sec61g	0.083691938	0.836938741	0.077306328
Gpx6	0.05686937	0.836938741	0.077306328
Polb	-0.072063492	0.836938741	0.077306328
Zbtb38	-0.036490834	0.836938741	0.077306328
Pitx2	0.069486823	0.836938741	0.077306328
Itpr3	0.094570985	0.836938741	0.077306328
Kidins220	0.087572623	0.836938741	0.077306328
Stx19	-0.068763882	0.836938741	0.077306328
Sdf4	0.114983004	0.836938741	0.077306328
WDR92	-0.101205502	0.836938741	0.077306328
Clns1a	-0.078877294	0.836938741	0.077306328
Sdha	0.066628239	0.836938741	0.077306328
Ly6g	0.066160801	0.836938741	0.077306328
Lrrc73	-0.122938385	0.837059449	0.077243697
Ccdc89	0.077971847	0.837059449	0.077243697
Ppp2r1a	0.203313451	0.837059449	0.077243697
Ccdc71l	0.296307646	0.837059449	0.077243697
Gpat4	0.063842411	0.837059449	0.077243697
Pcp4l1	-0.063631042	0.837059449	0.077243697
Nmur2	0.068863362	0.837059449	0.077243697
Fam205a1	0.069697059	0.837059449	0.077243697
Rnf216	0.114727691	0.837059449	0.077243697
Pitx1	0.081994452	0.837059449	0.077243697
Mafa	0.06590212	0.837059449	0.077243697
Ptk2b	0.043083093	0.837227658	0.077156433
Smc2os	0.07616108	0.837770371	0.076875003
Vamp4	0.081265239	0.837770371	0.076875003
Zfp444	-0.107561549	0.837837803	0.076840048
Mrpl23	0.167983496	0.8379102	0.076802523
Hsd17b10	-0.084167	0.838101563	0.07670335
Pitrm1	0.042452398	0.838101563	0.07670335
Pfdn2	-0.139429581	0.838143131	0.07668181
Pax6	0.074239339	0.838307721	0.076596534
Wdyhv1	0.046380005	0.838307721	0.076596534
Klhdc10	-0.052630853	0.838307721	0.076596534
Hspb7	0.080667136	0.83863004	0.076429585
Papolg	0.089703047	0.83863004	0.076429585
Dnah11	0.063529808	0.83863004	0.076429585
Ddx46	-0.059914316	0.83863004	0.076429585
Ccdc33	0.058230474	0.83863004	0.076429585
Rhd	0.078670074	0.838927862	0.076275382
Celsr2	0.092738509	0.839036578	0.076219105
Ttc1	-0.083836592	0.839036578	0.076219105
Supt5	0.06472528	0.839036578	0.076219105

Anks1b	0.072347384	0.839036578	0.076219105
Agps	0.083251766	0.839036578	0.076219105
Dgkq	0.095299223	0.839036578	0.076219105
Phka2	-0.088134943	0.839036578	0.076219105
Vstm2l	-0.122414197	0.839036578	0.076219105
Vps53	-0.059117328	0.839124428	0.076173636
Akr1a1	-0.069513832	0.839234359	0.076116744
Mettl21c	0.056873248	0.839234359	0.076116744
Pak1ip1	-0.091341454	0.839234359	0.076116744
Clcn4	-0.067484593	0.839234359	0.076116744
Fars2	-0.060831866	0.839234359	0.076116744
Spata1	-0.089146774	0.839234359	0.076116744
Knop1	-0.056465032	0.839234359	0.076116744
Timm8b	-0.147811135	0.839309526	0.076077848
Ift88	0.081201028	0.839309526	0.076077848
MTAP7D3	0.094903018	0.839309526	0.076077848
Rpp25l	-0.066557479	0.839309526	0.076077848
Zar1	0.065984643	0.839309526	0.076077848
Cog8	0.070305169	0.839309526	0.076077848
Actn2	-0.212815589	0.839309526	0.076077848
Nfxl1	-0.063303133	0.839309526	0.076077848
Pax2	-0.064063319	0.839309526	0.076077848
Txnrd1	0.053972482	0.839309526	0.076077848
Cyp17a1	-0.070330847	0.839318478	0.076073215
Tmem208	-0.047171653	0.839330532	0.076066979
Ube2k	-0.080970686	0.839412672	0.076024479
Pou2af1	0.179122074	0.839532599	0.075962436
Ldhb	0.06677187	0.839532599	0.075962436
Smim9	0.061853163	0.839532599	0.075962436
Inhbe	-0.274745573	0.839532599	0.075962436
Rab11a	-0.060230021	0.839532599	0.075962436
Mga	-0.058392322	0.839532599	0.075962436
Hepacam2	-0.174905809	0.839532599	0.075962436
Trim23	0.053395815	0.839532599	0.075962436
Sfmbt2	-0.046254409	0.839532599	0.075962436
Hif1an	-0.168078668	0.839532599	0.075962436
Cul3	-0.05989495	0.839532599	0.075962436
Akap7	0.125549903	0.839745921	0.075852097
Rad18	-0.085414995	0.839745921	0.075852097
Ythdc1	-0.072108759	0.839745921	0.075852097
Zfp541	0.092743946	0.839745921	0.075852097
Tmem42	0.056514827	0.839745921	0.075852097
Samm50	0.106544992	0.839745921	0.075852097
Kcnmb4os	0.196972898	0.839745921	0.075852097
KDELC1	0.063372921	0.839745921	0.075852097
Upk3a	0.097187287	0.839897346	0.075773791
Trp53inp2	-0.236728061	0.840442944	0.075491765
Suv39h2	-0.262492638	0.840442944	0.075491765

Atp6v0d1	0.168895517	0.840442944	0.075491765
Nova2	-0.079782993	0.840442944	0.075491765
Kpna6	-0.054660646	0.840442944	0.075491765
Slc14a2	0.071148132	0.840442944	0.075491765
Derl1	0.167356636	0.840442944	0.075491765
Zwint	-0.058410829	0.840442944	0.075491765
St8sia6	-0.11046351	0.840442944	0.075491765
Ralgapa2	0.05631825	0.840442944	0.075491765
Rnf225	-0.062780107	0.840442944	0.075491765
Efr3b	0.08011321	0.840442944	0.075491765
Rnf167	-0.088192403	0.840442944	0.075491765
Ndufb4	0.170220212	0.840442944	0.075491765
Spry4	-0.057342599	0.840442944	0.075491765
Disp1	0.056511463	0.840442944	0.075491765
Cyct	0.062892572	0.840442944	0.075491765
Apol10b	-0.066713554	0.840569286	0.075426483
Traf5	0.080422959	0.840755665	0.075330198
Coa6	-0.107885418	0.840755665	0.075330198
Tdrd7	0.041662781	0.840755665	0.075330198
Usp9x	-0.062514878	0.840755665	0.075330198
Ankle2	0.063086494	0.840755665	0.075330198
Add1	0.062528536	0.840755665	0.075330198
Lta4h	-0.179784859	0.840979782	0.075214445
Hdx	-0.172376217	0.841005035	0.075201404
Syt5	0.182584707	0.841005035	0.075201404
Scd4	-0.123398438	0.841005035	0.075201404
Mta2	0.075267739	0.841005035	0.075201404
Sema4c	0.051305854	0.841005035	0.075201404
Taf1c	-0.106149356	0.841005035	0.075201404
Dync1li1	0.070538717	0.841005035	0.075201404
Rec8	0.201512989	0.841005035	0.075201404
Hc	-0.083596556	0.841005035	0.075201404
Rhebl1	-0.106800259	0.841005035	0.075201404
Samd1	0.119308418	0.841005035	0.075201404
Zfp677	-0.115296352	0.841005035	0.075201404
Zkscan8	0.068935886	0.841005035	0.075201404
Dmwd	-0.111531088	0.841005035	0.075201404
Zfp39	0.075869096	0.841005035	0.075201404
Kctd3	-0.078334883	0.841005035	0.075201404
Bnip3	0.051389233	0.841005035	0.075201404
Alkbh3	-0.095080647	0.841005035	0.075201404
Trerf1	-0.11367618	0.841005035	0.075201404
AU021092	-0.185888542	0.841005035	0.075201404
Zfp58	-0.068827379	0.841005035	0.075201404
Tceal3	0.099565604	0.841005035	0.075201404
Opn1sw	0.041505754	0.841005035	0.075201404
Bod1l	-0.117240944	0.841005035	0.075201404
Coprs	0.098942178	0.841005035	0.075201404

Ppt1	0.114122217	0.841005035	0.075201404
Tnks	-0.084817232	0.841005035	0.075201404
Ndufaf5	0.272659893	0.841005035	0.075201404
Fut4	-0.143108686	0.841005035	0.075201404
Ap2a1	-0.079914974	0.841005035	0.075201404
Tslp	0.043811065	0.841005035	0.075201404
Tsc22d1	0.153519635	0.841005035	0.075201404
Ms4a15	-0.054683642	0.841005035	0.075201404
Kcnu1	-0.132040452	0.841005035	0.075201404
Krit1	-0.051825334	0.841005035	0.075201404
Ccdc66	0.056208947	0.841005035	0.075201404
Hmgxb3	-0.109477457	0.841005035	0.075201404
Mutyh	0.073242404	0.841005035	0.075201404
Rbsn	-0.076573061	0.841005035	0.075201404
Shroom3	0.107799416	0.841005035	0.075201404
Pex1	-0.068601295	0.841005035	0.075201404
Dhodh	0.176383547	0.841005035	0.075201404
Terf2	0.06755821	0.841005035	0.075201404
Mthfsd	0.071559019	0.841005035	0.075201404
Wdr82	0.084427606	0.841005035	0.075201404
Arf3	-0.06079473	0.841005035	0.075201404
Nfatc2	-0.058190355	0.841005035	0.075201404
Cplx4	0.049163817	0.841005035	0.075201404
Smarcd1	-0.0550219	0.841005035	0.075201404
Ect2l	0.053093669	0.841005035	0.075201404
Limk2	0.17268117	0.841005035	0.075201404
Ppie	0.098342286	0.841005035	0.075201404
Ptgs2	0.06733879	0.841005035	0.075201404
Prkag3	0.138344292	0.841005035	0.075201404
Cpsf4	-0.136037427	0.841005035	0.075201404
Prm1	0.057058367	0.841005035	0.075201404
Isoc2b	0.09438163	0.841005035	0.075201404
Atf1	0.337918684	0.841005035	0.075201404
Mcts1	-0.098554421	0.841005035	0.075201404
Cep104	0.033954818	0.841005035	0.075201404
Zfp286	0.063683807	0.841005035	0.075201404
Pspn	-0.071578708	0.841005035	0.075201404
Phex	0.062221174	0.841005035	0.075201404
Cc2d2a	0.061325424	0.841005035	0.075201404
Wdr27	0.042881525	0.841005035	0.075201404
AI846148	0.070826009	0.84166702	0.07485969
Topbp1	0.061030894	0.84166702	0.07485969
Ncoa4	-0.060601307	0.84166702	0.07485969
Nob1	0.097394035	0.841792009	0.074795201
Pitpnb	0.13340828	0.841792009	0.074795201
Esd	0.169857151	0.841826807	0.074777249
Ubd	-0.034319669	0.841826807	0.074777249
Dcp1b	-0.149018484	0.841848083	0.074766273

Olfr287	0.141889943	0.841848083	0.074766273
Tle3	0.312594032	0.841848083	0.074766273
Pnpla6	-0.129216132	0.841848083	0.074766273
Dync1h1	0.060862101	0.841848083	0.074766273
Cep83os	-0.164573819	0.841848083	0.074766273
HIST1H1E	-0.085764448	0.841848083	0.074766273
Stxbp5	-0.073604074	0.841848083	0.074766273
Ola1	0.084098963	0.841848083	0.074766273
Gtf3c6	0.095554524	0.841848083	0.074766273
Zfp322a	-0.093839195	0.841848083	0.074766273
Snhg5	-0.150135439	0.841848083	0.074766273
Vps41	0.071138899	0.841848083	0.074766273
Mansc1	-0.056320863	0.841848083	0.074766273
Enpp5	0.075547104	0.841848083	0.074766273
D5Ertd579	-0.075716877	0.841848083	0.074766273
Pdik1l	-0.096943525	0.841848083	0.074766273
Gstm5	0.111928968	0.841848083	0.074766273
Asl	-0.051981916	0.841848083	0.074766273
Hes6	-0.105639399	0.841848083	0.074766273
Ankrd17	0.068156698	0.841848083	0.074766273
AF529169	0.143066143	0.841848083	0.074766273
Acsl6	0.05294316	0.841848083	0.074766273
Fam168a	-0.061377732	0.841848083	0.074766273
Zbtb9	0.075276534	0.841848083	0.074766273
Zcchc10	0.045434088	0.841848083	0.074766273
Lgalsl	-0.044633495	0.841848083	0.074766273
Atp1b3	0.059538967	0.841848083	0.074766273
Dand5	0.059989581	0.841848083	0.074766273
Zfp760	-0.060170696	0.841848083	0.074766273
Zfand2a	0.078202042	0.841848083	0.074766273
Galnt16	-0.07691816	0.841848083	0.074766273
Actr8	-0.064826052	0.841848083	0.074766273
Tbc1d16	-0.061814063	0.841848083	0.074766273
Per2	-0.094901044	0.841848083	0.074766273
Chadl	-0.05490523	0.841848083	0.074766273
Trmt12	0.056145713	0.841848083	0.074766273
Stradb	0.049995437	0.841848083	0.074766273
Slc9b2	0.061574766	0.841848083	0.074766273
Ubqln2	-0.113055526	0.84202072	0.074677222
Hs2st1	-0.163434003	0.84202072	0.074677222
Sdhc	0.126372188	0.84202072	0.074677222
FAM84B	-0.266438694	0.84202072	0.074677222
Cdcp1	0.176657874	0.84202072	0.074677222
Gdap1l1	-0.19525752	0.84202072	0.074677222
Mapkbp1	-0.141838573	0.84202072	0.074677222
Dap3	-0.139655383	0.84202072	0.074677222
BC051628	0.086337419	0.84202072	0.074677222
Wdtc1	-0.118326006	0.84202072	0.074677222

Pcdh1	0.092749902	0.84202072	0.074677222
Pum1	0.113031884	0.84202072	0.074677222
Ccdc167	0.110424131	0.84202072	0.074677222
Atp11a	0.170744268	0.84202072	0.074677222
Vipr2	0.119383357	0.84202072	0.074677222
Eya1	0.07856167	0.84202072	0.074677222
Fam228b	-0.08553799	0.84202072	0.074677222
Atf4	0.081497463	0.84202072	0.074677222
Oxtr	0.056678757	0.84202072	0.074677222
Ndufs2	0.05936039	0.84202072	0.074677222
Cfap77	-0.061792508	0.84202072	0.074677222
Erc1	0.064038561	0.84202072	0.074677222
Cyth2	-0.189091884	0.84202072	0.074677222
Fgf2os	0.154829205	0.84202072	0.074677222
Prkaa1	0.063605639	0.84202072	0.074677222
Adora2b	0.130132155	0.84202072	0.074677222
Ndufaf2	0.095086426	0.84202072	0.074677222
Pcyt1b	0.093931718	0.84202072	0.074677222
Rbm10	0.083470398	0.84202072	0.074677222
Rabgap1	-0.082834449	0.84202072	0.074677222
Tpd52	0.057374589	0.84202072	0.074677222
Cd3d	-0.075711195	0.84202072	0.074677222
Zfp658	-0.065778607	0.84202072	0.074677222
BC048403	-0.054862768	0.84202072	0.074677222
Accsl	0.08292696	0.84202072	0.074677222
Mrpl54	-0.085440506	0.84202072	0.074677222
Scnn1g	0.052521194	0.84202072	0.074677222
Pde7a	-0.043833714	0.84202072	0.074677222
Aire	0.08414629	0.84202072	0.074677222
Itgb8	0.084078525	0.84202072	0.074677222
Rap2b	-0.078268862	0.84202072	0.074677222
HIST1H4B	0.066420387	0.84202072	0.074677222
Chp1	0.051840715	0.84202072	0.074677222
Smap2	-0.065485841	0.84202072	0.074677222
Proser1	0.064634722	0.84202072	0.074677222
Zfp652os	0.054776928	0.84202072	0.074677222
Ncstn	-0.170074584	0.84202072	0.074677222
Myh3	0.138192246	0.84202072	0.074677222
Ift122	0.102604716	0.84202072	0.074677222
Hsbp1l1	0.504608359	0.84202072	0.074677222
Ppp1r12c	-0.065997733	0.84202072	0.074677222
Dgkk	0.095776149	0.84202072	0.074677222
Olfr1535	0.066383115	0.84202072	0.074677222
Pcmtd2	-0.056952299	0.84202072	0.074677222
Gdpd1	0.071577972	0.84202072	0.074677222
Pafah1b2	0.04262152	0.84202072	0.074677222
Plekha7	-0.07714031	0.84202072	0.074677222
Sp7	-0.082016147	0.84202072	0.074677222

Shisa9	0.080869377	0.84202072	0.074677222
Vps9d1	0.062112908	0.84202072	0.074677222
Mettl5	-0.116574978	0.84202072	0.074677222
Ciapin1	0.188694964	0.84202072	0.074677222
Cd300ld4	-0.039495029	0.84202072	0.074677222
Aloxe3	-0.068381502	0.84202072	0.074677222
BC029722	-0.156946979	0.84202072	0.074677222
Gnl3l	-0.0505559	0.84202072	0.074677222
Rpl34	0.054696064	0.84202072	0.074677222
Prrg2	0.070691654	0.84202072	0.074677222
Snx1	0.063496764	0.84202072	0.074677222
Arl13b	0.070680058	0.84202072	0.074677222
Akap11	0.091274925	0.84202072	0.074677222
Bag5	0.045829474	0.84202072	0.074677222
Pim1	-0.055641283	0.84202072	0.074677222
Abcg2	-0.047798889	0.84202072	0.074677222
Mzt1	-0.095913491	0.84202072	0.074677222
Slc25a2	0.111325372	0.84202072	0.074677222
Fzd9	0.065201053	0.84202072	0.074677222
Azin2	-0.059039489	0.84202072	0.074677222
Gpr176	-0.08780068	0.84202072	0.074677222
TMEM189	-0.057347736	0.84202072	0.074677222
Me3	-0.057177446	0.84202072	0.074677222
Insl5	-0.053533726	0.84202072	0.074677222
Adat2	0.192417994	0.84202072	0.074677222
HIST1H2BI	-0.055776542	0.84202072	0.074677222
Lypd3	0.163691459	0.84202072	0.074677222
Hars2	-0.138413673	0.84202072	0.074677222
Chat	0.046246376	0.84202072	0.074677222
Rab11b	-0.088786369	0.84202072	0.074677222
Snora23	-0.082954888	0.84202072	0.074677222
HIST1H2A(	0.060414416	0.84202072	0.074677222
Larp6	0.090549784	0.84202072	0.074677222
Zfand2b	-0.04604674	0.84202072	0.074677222
Sub1	0.06586669	0.84202072	0.074677222
Neurod4	-0.047238523	0.84202072	0.074677222
Cntnap4	0.137188454	0.84202072	0.074677222
Mtcp1	-0.05741453	0.84202072	0.074677222
RFWD2	0.165529816	0.84202072	0.074677222
Mrpl24	-0.058589331	0.84202072	0.074677222
Usp5	-0.059294374	0.84202072	0.074677222
Zfp949	0.060589137	0.84202072	0.074677222
Mvd	0.063182174	0.84202072	0.074677222
Bcor	0.058592181	0.84202072	0.074677222
Cd46	0.057768318	0.84202072	0.074677222
Prox2	0.057677097	0.84202072	0.074677222
Lrrc20	0.057495068	0.84202072	0.074677222
Chrnb4	0.057337436	0.84202072	0.074677222

Chrne	0.057330576	0.84202072	0.074677222
Asb17os	0.057243716	0.84202072	0.074677222
Klf15	0.05094109	0.842193414	0.074588159
Prdm10	-0.09844664	0.84225584	0.074555969
Zfp119a	-0.17943138	0.84225584	0.074555969
Rubie	0.134765446	0.84225584	0.074555969
Prdm8	-0.148475307	0.84225584	0.074555969
Elf3	-0.296960283	0.84225584	0.074555969
Pdcd11	0.063768404	0.84225584	0.074555969
Mkln1	0.097981349	0.84225584	0.074555969
Emc10	-0.086478514	0.84225584	0.074555969
Iqck	-0.164388455	0.84225584	0.074555969
Dhrs13	-0.060305172	0.84225584	0.074555969
Ppm1d	0.083354289	0.84225584	0.074555969
Fam184b	-0.154542445	0.84225584	0.074555969
Polr3d	-0.059124082	0.84225584	0.074555969
Rnf123	0.067568413	0.84225584	0.074555969
Ddx24	0.122779646	0.84225584	0.074555969
Parl	0.068909853	0.84225584	0.074555969
Unc5c	0.093123589	0.84225584	0.074555969
Dtymk	0.138947583	0.842315365	0.074525277
Tns4	0.053982993	0.842315365	0.074525277
Cog1	0.149615833	0.842415028	0.074473894
Plcg1	-0.111097913	0.842415028	0.074473894
Sqstm1	-0.140439113	0.842415028	0.074473894
Vezt	0.069192333	0.842488644	0.074435944
Kyat3	-0.09049845	0.842567439	0.074395328
Igflr1	-0.247839602	0.842614272	0.074371189
Siglec15	0.079568303	0.842614272	0.074371189
Tsr2	0.067917785	0.842614272	0.074371189
Prol1	0.107258744	0.842614272	0.074371189
Golga7	0.099802762	0.842614272	0.074371189
Abcd3	-0.111177851	0.842614272	0.074371189
Cilp2	-0.075705535	0.842665534	0.074344769
Ism1	0.109104101	0.842665534	0.074344769
Mfsd8	0.076213981	0.842665534	0.074344769
Serpinb11	0.070499058	0.842665534	0.074344769
Slc6a16	0.221974405	0.842665534	0.074344769
Tatdn3	-0.07184698	0.84289873	0.0742246
Spag6l	0.134227928	0.842940256	0.074203205
Skor2	0.080273911	0.842940256	0.074203205
Oxt	-0.151532212	0.842970242	0.074187756
Cbx2	-0.063948857	0.842970242	0.074187756
Cnot9	-0.08522394	0.842970242	0.074187756
CXX1A	0.067777449	0.842970242	0.074187756
Slc35b4	-0.110792897	0.842970242	0.074187756
Rheb	-0.050801895	0.842970242	0.074187756
Prkaca	-0.077709313	0.842970242	0.074187756

Psme3	-0.050356869	0.842970242	0.074187756
Zfp870	-0.073536251	0.842970242	0.074187756
Rpl35a	-0.065704248	0.842970242	0.074187756
Sp4	-0.070044866	0.842970242	0.074187756
Lipc	-0.092481366	0.842970242	0.074187756
Ntsr2	-0.151710109	0.842989947	0.074177604
Rragd	0.369405567	0.843830818	0.073744618
Pik3r2	0.055826062	0.843830818	0.073744618
Hmgcl	-0.064606831	0.843870502	0.073724194
Ier5l	-0.145374487	0.843890009	0.073714155
Ndufaf1	0.098035638	0.84406988	0.073621597
Zfp563	0.074184243	0.844095559	0.073608384
Aoc2	-0.083104618	0.844118977	0.073596336
Gzf1	0.05990019	0.844118977	0.073596336
Palm	0.068984176	0.844118977	0.073596336
Kcne2	0.070968794	0.844118977	0.073596336
H2AFB3	0.093916584	0.844118977	0.073596336
PLA2G16	0.049392103	0.844118977	0.073596336
Hnf1a	-0.082675844	0.844241743	0.073533178
Coq2	-0.10661864	0.844241743	0.073533178
Dusp26	0.060901637	0.844241743	0.073533178
Tigd3	-0.059307971	0.844241743	0.073533178
Abcc4	0.086764464	0.84425111	0.073528359
Dnah7c	0.088965212	0.84425111	0.073528359
Calca	-0.153489961	0.844389947	0.073456946
Amy2a5	0.079044666	0.844389947	0.073456946
Mfap3	-0.055403062	0.844389947	0.073456946
Mpp7	0.081921205	0.844468924	0.073416328
Ccpg1	-0.076512043	0.844468924	0.073416328
Zfyve28	0.078342718	0.844468924	0.073416328
Ccdc34	-0.144278286	0.844468924	0.073416328
Bloc1s5	-0.087142494	0.844468924	0.073416328
Hs6st2	-0.153612594	0.845089589	0.073097248
Ccna1	-0.329664817	0.84511303	0.073085202
Gtf2ird2	-0.079241341	0.84511303	0.073085202
Gatad2a	0.047897625	0.845149806	0.073066304
Cc2d2b	-0.048021483	0.845149806	0.073066304
F12	-0.114406922	0.845372795	0.072951732
Qsox2	0.100846788	0.845372795	0.072951732
Fscn1	-0.110175711	0.845372795	0.072951732
Ccdc152	0.097387618	0.845372795	0.072951732
Foxred2	-0.033122555	0.845372795	0.072951732
Sgf29	0.055465575	0.845372795	0.072951732
Ppid	0.087446846	0.845372795	0.072951732
Pafah1b3	0.083408339	0.845372795	0.072951732
Sh3glb2	0.086973558	0.845372795	0.072951732
Rsrc1	-0.088337012	0.845372795	0.072951732
Capn5	0.087056472	0.845372795	0.072951732

Cars	-0.066269033	0.845372795	0.072951732
Adcy3	0.169946633	0.845372795	0.072951732
Tmem164	0.121723162	0.845372795	0.072951732
AW12168€	-0.070929772	0.845372795	0.072951732
Fem1c	-0.086074849	0.845390659	0.072942555
Kctd9	-0.225554524	0.845402119	0.072936668
Slc25a25	-0.190912316	0.845402119	0.072936668
Pcdha4	0.064603191	0.845402119	0.072936668
Ccni	-0.13830328	0.845402119	0.072936668
Arhgef10l	-0.051275144	0.845402119	0.072936668
Mab21l1	-0.072826143	0.845402119	0.072936668
VIMP	-0.070836801	0.845402119	0.072936668
Ddx19a	-0.047653405	0.845530396	0.072870775
Thbs3	0.246709206	0.845530396	0.072870775
Il31ra	0.054523245	0.845576552	0.072847069
Tmtc3	0.076798325	0.845984785	0.072637448
Smyd2	-0.259194184	0.846197645	0.072528188
Brcc3	-0.203748401	0.846197645	0.072528188
Kank3	-0.170470758	0.846197645	0.072528188
Foxj3	-0.112154135	0.846197645	0.072528188
Lama2	-0.097299512	0.846197645	0.072528188
Kif3b	-0.101494831	0.846197645	0.072528188
Memo1	-0.079776383	0.846197645	0.072528188
Adam15	-0.072934334	0.846197645	0.072528188
Zbed4	-0.133444878	0.846197645	0.072528188
Aspscr1	0.070333638	0.846197645	0.072528188
Gmppb	-0.047441149	0.846197645	0.072528188
Rabep2	0.195528824	0.846197645	0.072528188
Pcyt2	-0.096365259	0.846197645	0.072528188
CK137956	0.054754513	0.846197645	0.072528188
Hmgn5	0.076703205	0.846197645	0.072528188
Shroom4	-0.072529381	0.846197645	0.072528188
Cul9	0.047890756	0.846197645	0.072528188
Gc	-0.056343711	0.846197645	0.072528188
Ap1m2	0.09936528	0.846197645	0.072528188
Aipl1	0.054295158	0.846197645	0.072528188
Pcdhga9	0.098817442	0.846197645	0.072528188
Zfp831	-0.062409954	0.846197645	0.072528188
Rexo1	-0.217007004	0.846197645	0.072528188
Zfp622	-0.05871811	0.846197645	0.072528188
Olfr94	-0.108505044	0.846197645	0.072528188
Lsm14b	-0.137956137	0.846197645	0.072528188
Cerkl	0.067896179	0.846197645	0.072528188
Dhx29	-0.186924575	0.846197645	0.072528188
Wrn	0.050497861	0.846197645	0.072528188
Pet100	-0.069177059	0.846197645	0.072528188
Tmem200	-0.062238822	0.846197645	0.072528188
Spty2d1	-0.068713571	0.846197645	0.072528188

Cdk9	-0.185536335	0.846197645	0.072528188
Mapk1ip1l	0.039234076	0.846197645	0.072528188
Myo19	-0.062255742	0.846197645	0.072528188
Tex36	-0.10509709	0.846197645	0.072528188
Axdnd1	-0.175521697	0.846197645	0.072528188
Ppp1r2	0.058346354	0.846197645	0.072528188
Slc22a5	0.045699566	0.846197645	0.072528188
Rps24	-0.18351783	0.846197645	0.072528188
Ipo11	-0.06625883	0.846197645	0.072528188
Dlat	-0.060706025	0.846197645	0.072528188
Dlx6os1	0.051409045	0.846197645	0.072528188
Zscan29	0.097438262	0.846197645	0.072528188
Eogt	0.068292234	0.846197645	0.072528188
Chd2	0.058626014	0.846197645	0.072528188
Ipo8	-0.044970764	0.846197645	0.072528188
Ext2	0.181041267	0.846197645	0.072528188
FAM63A	0.053707356	0.846197645	0.072528188
Shisa4	0.236288581	0.846197645	0.072528188
Prrc2c	0.061679823	0.846197645	0.072528188
Prkab1	-0.084050642	0.846197645	0.072528188
Pcnx	0.061007069	0.846197645	0.072528188
Tktl1	0.068857786	0.846197645	0.072528188
Vti1b	0.129780397	0.846197645	0.072528188
Smim10l2	0.055339659	0.846197645	0.072528188
Wdr33	0.068806037	0.846197645	0.072528188
Drd2	-0.059612246	0.846197645	0.072528188
Zfp92	-0.076314687	0.846197645	0.072528188
Crem	0.135716852	0.846197645	0.072528188
Tomm34	0.122558108	0.846197645	0.072528188
Atxn7l3b	0.063150745	0.846197645	0.072528188
Slc6a6	-0.112068041	0.846197645	0.072528188
Ankzf1	0.050556493	0.846197645	0.072528188
Amn	-0.126724785	0.846197645	0.072528188
Dxo	0.046637789	0.846197645	0.072528188
Sirt4	-0.188038986	0.846197645	0.072528188
Rpl19	0.051119659	0.846197645	0.072528188
Zfp938	-0.085758489	0.846197645	0.072528188
Psrc1	0.191602698	0.846197645	0.072528188
Ssh2	-0.130961902	0.846197645	0.072528188
Rad51b	0.256950111	0.846197645	0.072528188
Tinf2	-0.070178694	0.846197645	0.072528188
Larp1b	-0.04847227	0.846197645	0.072528188
Dctd	-0.13011518	0.846197645	0.072528188
Lin28b	-0.059889204	0.846197645	0.072528188
Zswim6	0.084873575	0.846197645	0.072528188
Plppr2	-0.053278872	0.846197645	0.072528188
Mbtd1	-0.05300734	0.846197645	0.072528188
Polr1b	0.062719075	0.846197645	0.072528188

Egfem1	0.101932467	0.846197645	0.072528188
Ubash3b	0.192018012	0.846197645	0.072528188
Uba3	0.058780425	0.846197645	0.072528188
Pla2g2c	0.127546722	0.846197645	0.072528188
Iqcc	-0.046241996	0.846197645	0.072528188
Zfp784	0.065394017	0.846197645	0.072528188
Mdga2	0.085230007	0.846197645	0.072528188
Cpeb1	-0.035460664	0.846197645	0.072528188
Ube2j1	-0.034750686	0.846197645	0.072528188
Mrps11	-0.082114681	0.846197645	0.072528188
Odf4	0.064996738	0.846197645	0.072528188
Pcdhga3	0.044147826	0.846197645	0.072528188
MGEA5	0.064598857	0.846197645	0.072528188
Rsph14	-0.051994538	0.846197645	0.072528188
Rpa2	0.049804101	0.846197645	0.072528188
Ubr3	0.062512496	0.846197645	0.072528188
Pdcd7	0.047768216	0.846197645	0.072528188
Tnfaip8l1	-0.039632482	0.846197645	0.072528188
Trmt11	0.104569753	0.846197645	0.072528188
Soat2	-0.08692123	0.846197645	0.072528188
Cenpo	0.08971644	0.846197645	0.072528188
Exoc3l	-0.07379833	0.846197645	0.072528188
Aldoa	-0.10960371	0.846197645	0.072528188
Sp3	0.060352088	0.846197645	0.072528188
Nps	0.054534559	0.846197645	0.072528188
Rabggtb	-0.450043951	0.846197645	0.072528188
Krtap28-1:	0.037069471	0.846197645	0.072528188
Elfn2	-0.086128443	0.846197645	0.072528188
Nxph2	-0.036800435	0.846197645	0.072528188
Dcstamp	0.193717855	0.846197645	0.072528188
Zfp638	-0.068687673	0.846197645	0.072528188
Pigm	0.053504576	0.846197645	0.072528188
Rpl24	0.05253526	0.846197645	0.072528188
Rgs14	-0.147047883	0.846215667	0.072518939
Zfp692	0.151675126	0.846215667	0.072518939
Mroh4	-0.319179021	0.846215667	0.072518939
Tmem50b	0.097660664	0.846215667	0.072518939
Det1	-0.140179853	0.846215667	0.072518939
Pdhx	0.072750953	0.846215667	0.072518939
Dlx5	0.094642142	0.846215667	0.072518939
Pde1c	-0.17240577	0.846215667	0.072518939
Slc5a10	0.054383757	0.846215667	0.072518939
Coq4	-0.094760269	0.846215667	0.072518939
Tmx2	0.104336147	0.846215667	0.072518939
Tor1b	-0.052208896	0.846215667	0.072518939
Ap3d1	0.057167901	0.846215667	0.072518939
Pgs1	0.104152996	0.846215667	0.072518939
Ly6c1	0.054021686	0.846215667	0.072518939

Wrnip1	-0.076379816	0.846215667	0.072518939
Apoa1	-0.089386634	0.846215667	0.072518939
FAM150A	0.091271656	0.846215667	0.072518939
Trio	0.064162174	0.846215667	0.072518939
Surf6	0.052092093	0.846215667	0.072518939
Txndc11	-0.170591639	0.846323257	0.072463724
Traf3ip2	0.065393628	0.84638968	0.07242964
Sult3a1	-0.107419406	0.84638968	0.07242964
Stac	0.072672345	0.846588252	0.072327762
Ftx	0.194416444	0.846938841	0.07214795
Stox2	-0.054700246	0.846938841	0.07214795
Rabep1	0.080394578	0.846938841	0.07214795
Prickle4	-0.057273832	0.846938841	0.07214795
Ajap1	-0.126624496	0.846938841	0.07214795
Atn1	0.086437869	0.846938841	0.07214795
Zfp940	-0.114326	0.846938841	0.07214795
Dip2b	0.087232677	0.846938841	0.07214795
Cd247	0.059769561	0.846938841	0.07214795
Dhrs2	-0.153914901	0.846938841	0.07214795
Mc3r	0.067801029	0.846938841	0.07214795
Prkab2	0.053248328	0.846938841	0.07214795
Rnf7	0.059291782	0.846938841	0.07214795
Ank	-0.054582699	0.847305338	0.071960057
Dalrd3	-0.048239085	0.847305338	0.071960057
Tbata	0.071059586	0.847305338	0.071960057
Fitm1	-0.064486385	0.847350053	0.071937139
Zfp445	0.171405878	0.847591707	0.071813301
Farp1	-0.048103464	0.847591707	0.071813301
Arhgef15	0.12622361	0.847591707	0.071813301
Actg1	0.068192054	0.847591707	0.071813301
Rab3gap2	-0.050247575	0.847591707	0.071813301
Nags	-0.048377809	0.847591707	0.071813301
Tpi1	0.052595389	0.847591707	0.071813301
Cpt1b	0.06409879	0.847591707	0.071813301
Lmx1b	-0.037234056	0.847591707	0.071813301
Mrgprh	0.048189909	0.848027417	0.071590107
C1qtnf7	-0.079163509	0.848134426	0.071535308
Gpbp1	0.170658215	0.848345518	0.07142723
Rarb	-0.193796141	0.848345518	0.07142723
Vstm2a	0.141730855	0.848345518	0.07142723
Ap1ar	0.138994368	0.848345518	0.07142723
Kri1	0.071750964	0.848345518	0.07142723
Chd6	-0.083945043	0.848345518	0.07142723
Brms1	-0.099772632	0.848345518	0.07142723
Gfi1b	0.066414932	0.848345518	0.07142723
Atp5b	0.071625893	0.848345518	0.07142723
Crim1	0.086791889	0.848345518	0.07142723
IL1F9	-0.093771562	0.848345518	0.07142723

Mrgbp	-0.157081836	0.848345518	0.07142723
Ehf	0.0591299	0.848345518	0.07142723
Mbd6	0.177102962	0.848345518	0.07142723
ZCCHC5	0.092032837	0.848345518	0.07142723
Vta1	0.071142388	0.848345518	0.07142723
Snn	0.247421372	0.848345518	0.07142723
Vegfd	0.100156349	0.848345518	0.07142723
E2f1	0.072554579	0.848345518	0.07142723
Lrrc46	-0.101535242	0.848345518	0.07142723
Ebf2	0.074451485	0.848345518	0.07142723
AIM1	-0.054303598	0.848345518	0.07142723
Irx1	-0.055856011	0.848345518	0.07142723
Pira1	-0.068539749	0.848345518	0.07142723
Rbl2	-0.081110756	0.848345518	0.07142723
Itgb1bp1	0.049023196	0.848345518	0.07142723
Cdx1	-0.066555676	0.848345518	0.07142723
Eif2b4	0.137340277	0.848345518	0.07142723
Bri3	0.077877594	0.848345518	0.07142723
Trim63	0.057598548	0.848345518	0.07142723
Sart3	-0.051188107	0.848345518	0.07142723
Dctn1	-0.050869371	0.848345518	0.07142723
Emx2	-0.042938693	0.848345518	0.07142723
TMEM194	0.054591897	0.848345518	0.07142723
Hhip	-0.037505586	0.848345518	0.07142723
Hectd1	0.072537437	0.848345518	0.07142723
Itga2b	0.071020818	0.848345518	0.07142723
Srp54b	0.051571507	0.848345518	0.07142723
Mmgt2	-0.175761649	0.848345518	0.07142723
Lrrc27	-0.038470207	0.848345518	0.07142723
Hypk	0.080856048	0.848345518	0.07142723
C2cd4b	-0.060594483	0.848345518	0.07142723
Net1	-0.066708658	0.848345518	0.07142723
Dars2	0.079381148	0.848345518	0.07142723
Krtdap	0.076903723	0.848345518	0.07142723
Vapb	0.068093992	0.848345518	0.07142723
C2cd5	-0.168886357	0.848345518	0.07142723
Bmp8a	0.051278811	0.848345518	0.07142723
Armt1	0.050559157	0.848345518	0.07142723
Ganc	0.033866719	0.848345518	0.07142723
Lalba	0.14152598	0.848374072	0.071412613
Ccnyl1	0.077900892	0.848490241	0.071353148
Zc3h4	-0.08343291	0.848833015	0.071177737
Arl6ip5	-0.262535697	0.848833015	0.071177737
Akr1c14	-0.057872221	0.848833015	0.071177737
Ska2	-0.089218418	0.848833015	0.071177737
Bcl9	0.040192046	0.848833015	0.071177737
Scg2	0.042125151	0.848833015	0.071177737
Dgcr2	-0.107144925	0.848833015	0.071177737

Calr4	-0.081582661	0.848833015	0.071177737
Tacr3	0.142539457	0.848833015	0.071177737
Rfc1	0.114612546	0.848833015	0.071177737
Skp2	-0.095798816	0.848833015	0.071177737
Prss36	0.147030226	0.848833015	0.071177737
Arhgap33c	0.04641476	0.848833015	0.071177737
Dtx4	-0.060652367	0.848833015	0.071177737
Mtrf1l	0.077231397	0.848833015	0.071177737
Abca2	-0.061064638	0.848833015	0.071177737
Defb42	0.050309955	0.848833015	0.071177737
Zfp335os	0.050197965	0.848833015	0.071177737
Pdyn	0.124335919	0.848900171	0.071143379
Tmco5b	-0.286952248	0.848974405	0.071105403
Gcfc2	-0.038404793	0.849140351	0.071020521
En2	-0.139068417	0.849289906	0.070944038
Inpp4b	-0.128117371	0.849289906	0.070944038
Bcl11b	-0.149222347	0.849289906	0.070944038
Pnpt1	0.082017551	0.849289906	0.070944038
Tardbp	0.065263642	0.849289906	0.070944038
Ift20	0.290300122	0.849289906	0.070944038
Nbas	-0.066838509	0.849289906	0.070944038
Pcm1	-0.15420898	0.849398507	0.070888507
Mtif2	-0.125657668	0.849398507	0.070888507
Mras	-0.074640355	0.849398507	0.070888507
Rbm18	-0.046070353	0.849466603	0.070853691
Pcdhga10	0.061215613	0.849485295	0.070844135
Commd10	-0.083891338	0.849603315	0.070783802
Snx24	-0.130576032	0.849603315	0.070783802
Lamtor4	0.043927619	0.849603315	0.070783802
Zdhhc5	0.053535047	0.849603315	0.070783802
Cd47	0.105162576	0.849603315	0.070783802
Stim2	-0.048409605	0.849603315	0.070783802
Eri3	0.070137309	0.849603315	0.070783802
Ube3c	-0.141445508	0.849748402	0.070709643
Pced1b	-0.075828809	0.849748402	0.070709643
Bcl7b	0.077402038	0.849748402	0.070709643
Immt	0.078125912	0.849748402	0.070709643
Ogdhl	0.057115108	0.849748402	0.070709643
Ptgfr	0.05818053	0.849748402	0.070709643
Dkc1	0.066746437	0.849748402	0.070709643
Olfr543	-0.133015996	0.850275823	0.070440169
Xxylt1	-0.089711856	0.850749034	0.070198536
Agap2	0.076953728	0.850749034	0.070198536
Slc9a4	0.065346076	0.850749034	0.070198536
Ocrl	-0.161705704	0.850918526	0.070112021
Fam210b	0.091030253	0.850918526	0.070112021
Taf4b	-0.049376715	0.850976086	0.070082644
Dbpht2	-0.06942346	0.851182598	0.069977264

Urb2	0.089770021	0.851182598	0.069977264
Med9	0.060091996	0.851188352	0.069974328
Tbc1d22a	-0.040886077	0.851188352	0.069974328
Rnf126	-0.133106359	0.851325782	0.069904214
Snx2	0.113060472	0.851325782	0.069904214
Nipsnap1	0.077108258	0.851325782	0.069904214
Rps17	-0.143989591	0.851325782	0.069904214
Ier3	0.068464425	0.851325782	0.069904214
Padi3	-0.056697767	0.851325782	0.069904214
Acsm4	0.047770387	0.851325782	0.069904214
Prr32	0.133387067	0.851325782	0.069904214
Exoc1	-0.063040286	0.851325782	0.069904214
Brwd1	-0.048337776	0.851325782	0.069904214
Emb	0.080105326	0.851325782	0.069904214
Pcnx4	0.079934389	0.851325782	0.069904214
CSRP2BP	-0.044711858	0.851325782	0.069904214
Lin52	-0.057892036	0.851325782	0.069904214
Rpl17	0.033500038	0.851325782	0.069904214
Wbp2nl	0.066139474	0.851325782	0.069904214
ST5	-0.106641675	0.851677659	0.069724745
Yod1	0.398769762	0.851677659	0.069724745
St3gal1	-0.083283678	0.851677659	0.069724745
Cyc1	-0.162524826	0.851677659	0.069724745
Ccm2l	-0.085891587	0.851677659	0.069724745
Trit1	-0.060668746	0.851677659	0.069724745
Susd5	-0.058148882	0.851677659	0.069724745
Nhlrc4	-0.065885191	0.851677659	0.069724745
Usp20	-0.064947736	0.851677659	0.069724745
Gsdma	0.17978722	0.851677659	0.069724745
Cfap53	0.081603321	0.851677659	0.069724745
Chmp7	0.118429852	0.851677659	0.069724745
Pef1	-0.073418053	0.851681887	0.069722589
Rab10os	-0.058808795	0.851681887	0.069722589
Supt4a	-0.141717018	0.851798857	0.069662947
Akirin1	-0.047874409	0.851798857	0.069662947
Lpcat1	0.046558955	0.851798857	0.069662947
Fam217a	-0.084038161	0.851798857	0.069662947
Tepp	0.11215395	0.851798857	0.069662947
Herpud1	-0.059570832	0.851798857	0.069662947
Tsc22d3	-0.0549872	0.851798857	0.069662947
Smlr1	-0.053878757	0.851979293	0.06957096
Eml4	0.107557549	0.851979293	0.06957096
Ankrd61	0.058793327	0.851979293	0.06957096
Zc3h8	0.046900014	0.851979293	0.06957096
Kcnt1	-0.073095481	0.851979293	0.06957096
Gpalpp1	-0.054547191	0.851979293	0.06957096
Mtmr12	-0.056909506	0.851979293	0.06957096
Nudt8	0.399874107	0.851979293	0.06957096

Vnn1	-0.048583072	0.851979293	0.06957096
Ccnt2	-0.146652188	0.851979293	0.06957096
Zranb2	0.076624317	0.851979293	0.06957096
MARS	0.097910117	0.852122329	0.069498054
Kras	0.069708035	0.852122329	0.069498054
Vkorc1l1	-0.142906864	0.852175864	0.069470771
Kctd4	-0.108016882	0.852175864	0.069470771
Sh3pxd2a	-0.113202369	0.852175864	0.069470771
Kcnip2	-0.081489992	0.852175864	0.069470771
Zfp866	0.131647381	0.852175864	0.069470771
Tmem170	0.053244833	0.852175864	0.069470771
Ppox	-0.066299286	0.852175864	0.069470771
Rffl	-0.054573557	0.852175864	0.069470771
CCDC155	0.063113387	0.852175864	0.069470771
Marveld3	0.059401003	0.852175864	0.069470771
Tspan6	0.108018277	0.852175864	0.069470771
Ston1	-0.138482771	0.852175864	0.069470771
1-Mar	0.080474846	0.852175864	0.069470771
Trim13	-0.104679676	0.852175864	0.069470771
Nelfe	-0.048891671	0.852175864	0.069470771
Myh11	-0.115967987	0.852175864	0.069470771
Pkp1	0.128636342	0.852175864	0.069470771
Avpi1	0.039012405	0.852175864	0.069470771
Esrrb	0.105457635	0.852175864	0.069470771
Ankrd13b	0.085308659	0.852175864	0.069470771
Cdk5	-0.075793881	0.852175864	0.069470771
Capn9	0.067312112	0.852175864	0.069470771
Zcwpw1	0.059867316	0.852175864	0.069470771
Fam169b	0.054670459	0.852175864	0.069470771
Slc27a2	-0.048011774	0.852175864	0.069470771
Npsr1	-0.05187316	0.852175864	0.069470771
Coro1c	-0.06017042	0.852175864	0.069470771
Ints1	0.058049419	0.852175864	0.069470771
Ube2e1	0.113198213	0.852175864	0.069470771
Recql	0.049128256	0.852175864	0.069470771
Dcaf8	-0.07572161	0.852175864	0.069470771
Shbg	0.081392899	0.852175864	0.069470771
PRAMEF8	0.049397441	0.852175864	0.069470771
Wasf1	0.081423334	0.852267967	0.069423835
Emx2os	0.047368308	0.852267967	0.069423835
Igfbp1	-0.048837298	0.852267967	0.069423835
Paip2b	-0.086597816	0.852477082	0.069317288
Chka	-0.07256824	0.85253186	0.069289382
Dnajc4	0.082442435	0.85253186	0.069289382
Abhd17c	-0.129858516	0.85283069	0.06913718
Lsamp	0.3587355	0.85283069	0.06913718
Ccl28	0.238384403	0.85283069	0.06913718
Adnp	0.18102068	0.85283069	0.06913718

Eif2s2	-0.067257469	0.85283069	0.06913718
Kansl1	-0.081925988	0.85283069	0.06913718
Dtwd2	0.088905843	0.85283069	0.06913718
Fibp	-0.095386689	0.85283069	0.06913718
Ltbp4	-0.055954345	0.85283069	0.06913718
Tmem89	0.545129129	0.85283069	0.06913718
Alg2	0.112324056	0.85283069	0.06913718
Ppil6	-0.102629257	0.85283069	0.06913718
Igsf21	-0.141502388	0.85283069	0.06913718
Hectd3	-0.115607649	0.85283069	0.06913718
Egfl8	-0.08946848	0.85283069	0.06913718
Pygm	-0.068010093	0.85283069	0.06913718
Aagab	0.2304115	0.85283069	0.06913718
Foxp1	0.066566987	0.85283069	0.06913718
Stard6	0.07966547	0.85283069	0.06913718
Slc9a3	0.086044301	0.85283069	0.06913718
Prpf38b	0.055179731	0.85283069	0.06913718
Pcdhb20	-0.107645742	0.85283069	0.06913718
Glra4	-0.102127101	0.85283069	0.06913718
Polr3g	0.063737217	0.85283069	0.06913718
Cd320	0.062083107	0.85283069	0.06913718
Zfp637	0.064214812	0.85283069	0.06913718
Ankrd46	-0.075575452	0.85283069	0.06913718
Olfr56	-0.055285607	0.85283069	0.06913718
Uvrag	0.083234363	0.85283069	0.06913718
Rhbdl1	0.071957757	0.85283069	0.06913718
Akap8l	-0.165063492	0.85283069	0.06913718
Glp1r	-0.085202691	0.85283069	0.06913718
Thtpa	-0.056989578	0.85283069	0.06913718
Esam	-0.116795674	0.85283069	0.06913718
AI463170	-0.077935627	0.85283069	0.06913718
Polr2j	0.056187447	0.85283069	0.06913718
Lonrf1	0.057434042	0.85283069	0.06913718
Zfp697	0.062360756	0.85283069	0.06913718
Eif5b	-0.126917342	0.85283069	0.06913718
Phc2	-0.05493424	0.85283069	0.06913718
Ucn	-0.066350623	0.85283069	0.06913718
Tcp11l1	-0.052577329	0.85283069	0.06913718
RSG1	0.045300079	0.85283069	0.06913718
Slc30a9	0.071448469	0.85283069	0.06913718
Sec22c	-0.065807234	0.85283069	0.06913718
Klf11	-0.060094067	0.85283069	0.06913718
Utp14b	-0.047400678	0.85283069	0.06913718
Rbpj	-0.086531426	0.85283069	0.06913718
Jade2	0.069413057	0.85283069	0.06913718
Setx	-0.066960084	0.85283069	0.06913718
Derl2	0.12085217	0.85283069	0.06913718
Rnf170	0.062584098	0.85283069	0.06913718

FAM179B	-0.053655972	0.85283069	0.06913718
Cox6c	0.031737423	0.85283069	0.06913718
Gpr108	0.084912672	0.85283069	0.06913718
Adamts3	0.047195647	0.85283069	0.06913718
Duoxa1	0.071075486	0.85283069	0.06913718
Ppm1b	0.129012393	0.85283069	0.06913718
Actr10	-0.059852662	0.85283069	0.06913718
Dnajb2	0.062943126	0.85283069	0.06913718
Heca	0.047301085	0.85283069	0.06913718
Hpcal4	-0.103084268	0.853034804	0.069033249
8-Mar	0.18440638	0.853034804	0.069033249
Fn3krp	0.053610898	0.853034804	0.069033249
Scgb1a1	0.087685827	0.853034804	0.069033249
Phospho2	0.070962119	0.853034804	0.069033249
Spg20	-0.061096838	0.853034804	0.069033249
PIH1H3B	0.044484769	0.853034804	0.069033249
Asb5	0.112594393	0.853034804	0.069033249
Gpr75	0.069124558	0.853034901	0.0690332
Mettl16	0.065129468	0.853034901	0.0690332
Fam174b	-0.090364413	0.853334985	0.068880449
Wfdc10	-0.158527696	0.853885882	0.068600167
Npepps	0.146583208	0.853885882	0.068600167
Cdh4	0.070865177	0.853885882	0.068600167
Stk32c	0.374724158	0.853885882	0.068600167
Cep97	0.073793905	0.853885882	0.068600167
Phf20l1	0.102201362	0.853885882	0.068600167
Auh	-0.046753457	0.853885882	0.068600167
Arih1	0.045341268	0.854208497	0.068436113
Olfr520	0.044770081	0.854208497	0.068436113
Zbtb33	-0.117218739	0.854208497	0.068436113
Zfp174	0.05804038	0.854208497	0.068436113
Aym1	0.1081941	0.854322312	0.068378251
Tfap2b	-0.164053751	0.854376678	0.068350615
Smco3	0.065948165	0.85479706	0.06813698
Snx19	0.042673155	0.854862292	0.068103839
Stfa2l1	0.068013862	0.854862292	0.068103839
Mrps12	-0.062314381	0.856148226	0.067451039
Pi15	0.14573866	0.856401069	0.067322799
Cmtm8	0.078918476	0.856431055	0.067307593
H2-DMb2	0.04347952	0.856431055	0.067307593
Acot3	0.096187782	0.856431055	0.067307593
Nat8f5	0.063092943	0.856431055	0.067307593
Thap1	0.046198602	0.856798528	0.067121288
Batf3	-0.082631691	0.857007749	0.067015251
Fastkd2	0.071549608	0.857007749	0.067015251
Fcnb	0.067458485	0.857007749	0.067015251
Romo1	-0.070122582	0.857204544	0.066915535
S1pr4	0.043131439	0.857204544	0.066915535

Wdr36	0.071850661	0.85730263	0.066865844
Kctd2	-0.059077219	0.85730263	0.066865844
Tmem184	0.031385764	0.85730263	0.066865844
Dexi	-0.098515234	0.857615429	0.066707415
Mmd	0.04962428	0.857615429	0.066707415
Fam166b	0.048612424	0.857781712	0.066623217
Phtf2	0.059300711	0.857888937	0.066568933
Pgap1	0.098354475	0.857916699	0.066554879
Kcnmb4os	0.183700446	0.857916699	0.066554879
Zdhhc15	-0.067976244	0.857916699	0.066554879
Arfip2	0.114554709	0.857916699	0.066554879
Adgrb3	-0.0502845	0.857916699	0.066554879
Herc4	0.05928671	0.857916699	0.066554879
Narf	0.053419491	0.857916699	0.066554879
Kif3a	-0.048387114	0.857916699	0.066554879
Cyp21a1	0.150094998	0.857916699	0.066554879
Rab33b	-0.07222658	0.857916699	0.066554879
Gng13	-0.048552851	0.857969393	0.066528205
Scrn1	-0.060362308	0.858088094	0.066468124
Cacna2d4	0.06193667	0.858305497	0.066358106
Gas5	-0.045612148	0.858305497	0.066358106
Cct8l1	-0.164527731	0.858464431	0.066277694
D3ERTD25	0.146804086	0.858464431	0.066277694
Tmcc2	0.06584415	0.858464431	0.066277694
Lrfn3	0.062102494	0.858576753	0.066220875
Ctps2	0.082033443	0.858629598	0.066194145
Acr	0.075865926	0.858629598	0.066194145
Ykt6	0.128063278	0.858629598	0.066194145
Platr27	0.094883759	0.858629598	0.066194145
Hspbap1	-0.089578514	0.858629598	0.066194145
Vpreb2	0.053492904	0.858629598	0.066194145
Ctnnbl1	-0.111241402	0.858991532	0.066011117
Armc6	-0.169022457	0.859285892	0.065862318
Gdpd3	-0.149698701	0.859285892	0.065862318
Zfp446	-0.050434793	0.859285892	0.065862318
Mup3	0.136371924	0.859285892	0.065862318
Arhgef2	0.041179868	0.859285892	0.065862318
Gucy2e	0.041734069	0.859285892	0.065862318
Echdc2	0.060632023	0.859285892	0.065862318
Pla2g4e	0.048858767	0.859285892	0.065862318
Lect2	-0.047230088	0.859285892	0.065862318
Prodh	0.089251911	0.859285892	0.065862318
Synj2bp	0.045364702	0.859285892	0.065862318
Tac4	-0.131749844	0.859286109	0.065862209
Stap2	-0.055534283	0.859286109	0.065862209
mt-Rnr2	-0.133859543	0.859286109	0.065862209
Map2k7	0.101779472	0.859588827	0.065709238
Ctnnd2	-0.069951485	0.859588827	0.065709238

Gemin6	-0.059171279	0.859588827	0.065709238
Fbxw11	0.043100096	0.859588827	0.065709238
Tshb	0.058881689	0.859609997	0.065698542
Smim14	0.060829381	0.859694535	0.065655834
BC005624	-0.077148466	0.86029773	0.065351223
Bnipl	0.055327811	0.860383083	0.065308137
Tymp	-0.0939477	0.860383083	0.065308137
Apoh	0.218133185	0.860677292	0.065159655
Pgrmc2	-0.073144403	0.860939616	0.065027308
Shh	-0.055104993	0.860939616	0.065027308
Glyr1	0.087422224	0.860939616	0.065027308
Jtb	-0.053493507	0.861097753	0.064947544
Irf2bp1	0.109231916	0.861097753	0.064947544
Prrg4	-0.039020383	0.861097753	0.064947544
Syndig1l	-0.099517416	0.861098725	0.064947054
Alg1	-0.065313017	0.861233913	0.064878877
Zfp532	0.124177066	0.861233913	0.064878877
Pcdhgb2	-0.047750724	0.861233913	0.064878877
Gbf1	-0.055691928	0.861233913	0.064878877
Mib1	-0.1552298	0.861320317	0.064835309
Ppp1r35	0.114748058	0.861320317	0.064835309
Ino80c	-0.127293278	0.861320317	0.064835309
Ybey	-0.103230626	0.861320317	0.064835309
Yae1d1	-0.104106481	0.861320317	0.064835309
Nxph3	0.105511375	0.861320317	0.064835309
Nacc1	0.083248663	0.861320317	0.064835309
Ostc	0.096040791	0.861320317	0.064835309
Rasgef1a	-0.072867828	0.861320317	0.064835309
Pcdhb22	-0.15185502	0.861320317	0.064835309
Sparcl1	-0.115968681	0.861320317	0.064835309
Myom1	-0.084597837	0.861320317	0.064835309
Zfp101	-0.068806649	0.861320317	0.064835309
Ccdc141	-0.123172292	0.861320317	0.064835309
Armc1	-0.081159484	0.861320317	0.064835309
Ocel1	0.143865575	0.861320317	0.064835309
Pgm5	0.060261892	0.861320317	0.064835309
Nol10	-0.107638096	0.861320317	0.064835309
Otud7b	0.083875777	0.861320317	0.064835309
Nr6a1	-0.069063725	0.861320317	0.064835309
Sergef	0.065823013	0.861320317	0.064835309
Ppp1r15b	0.048757588	0.861320317	0.064835309
Hif3a	0.095308154	0.861320317	0.064835309
IKBKAP	0.065959764	0.861320317	0.064835309
Qdpr	-0.055792444	0.861320317	0.064835309
Cntnap2	-0.118044545	0.861320317	0.064835309
Skor1	-0.059861553	0.861320317	0.064835309
Purg	-0.113463079	0.861320317	0.064835309
Ccdc58	0.068495818	0.861320317	0.064835309

Prpf40a	0.049596028	0.861320317	0.064835309
Rbpjl	0.073262147	0.861320317	0.064835309
Zbtb42	-0.056197716	0.861320317	0.064835309
Pik3cb	-0.12284351	0.861320317	0.064835309
Lhx6	0.110069451	0.861320317	0.064835309
Rnf10	-0.114538177	0.861320317	0.064835309
Phf3	0.056678292	0.861320317	0.064835309
Hmcn1	0.060272586	0.861320317	0.064835309
Cdipt	0.10784847	0.861320317	0.064835309
Spire2	-0.040803075	0.861320317	0.064835309
TCEB3	-0.051988027	0.861320317	0.064835309
Pck2	0.05506948	0.861320317	0.064835309
Vps37b	-0.097847653	0.861320317	0.064835309
4-Mar	-0.06489471	0.861320317	0.064835309
Ttll12	-0.113872641	0.861320317	0.064835309
Srcap	0.053562885	0.861320317	0.064835309
Trim43c	-0.035145075	0.861320317	0.064835309
Ankrd35	-0.042421844	0.861320317	0.064835309
Cmas	-0.070106359	0.861320317	0.064835309
Acbd4	-0.055415799	0.861320317	0.064835309
Zdhhc20	0.059958116	0.861320317	0.064835309
Ak7	-0.093592315	0.861320317	0.064835309
Zdhhc1	-0.092985256	0.861320317	0.064835309
Mettl23	-0.101542217	0.861320317	0.064835309
Scrn2	0.054185768	0.861320317	0.064835309
Atad1	0.110873498	0.861320317	0.064835309
Gpd1	0.047255669	0.861320317	0.064835309
Shroom1	-0.044184659	0.861320317	0.064835309
Ssh3	-0.062896123	0.861320317	0.064835309
FAM208A	0.063672823	0.861320317	0.064835309
Tbpl1	0.264392758	0.861320317	0.064835309
NHP2L1	0.067408271	0.861320317	0.064835309
Col4a3	-0.049763288	0.861320317	0.064835309
Lmna	0.077375877	0.861320317	0.064835309
Eef1akmt1	-0.034425812	0.861320317	0.064835309
Etohd2	0.074162021	0.861320317	0.064835309
Pdzd3	0.105712791	0.861320317	0.064835309
Cuedc2	-0.05204283	0.861320317	0.064835309
Auts2	-0.06892457	0.861320317	0.064835309
Angpt4	0.044590379	0.861320317	0.064835309
Fam174a	0.054997208	0.861326666	0.064832107
Asb1	-0.061819494	0.861431068	0.064779469
Rgmb	0.104563184	0.861431068	0.064779469
Inpp5j	0.060945815	0.861481621	0.064753984
Bckdhb	-0.05281381	0.861535873	0.064726634
Zscan18	-0.34854854	0.861674334	0.064656843
Ptger3	0.059854163	0.861674334	0.064656843
Elk4	0.088495208	0.862020384	0.064482465

Tmem63a	-0.068531492	0.862092618	0.064446074
Ciart	-0.132005817	0.862187392	0.064398332
Fam220a	0.048681692	0.86228124	0.064351062
Nacc2	-0.11470414	0.862493177	0.064244332
Acd	-0.069861899	0.862493177	0.064244332
Pkd2l2	-0.091362721	0.862493177	0.064244332
Txnrd2	-0.151589643	0.862493177	0.064244332
Ltbp1	-0.061035708	0.862493177	0.064244332
Bpnt1	0.063384717	0.862493177	0.064244332
Fbln5	-0.073884254	0.862493177	0.064244332
Wdr43	-0.040190186	0.862493177	0.064244332
Mtpn	0.076922973	0.862493177	0.064244332
Wfdc15a	-0.032255785	0.862493177	0.064244332
Elp2	0.185098786	0.862493177	0.064244332
Abra	0.050562579	0.862569243	0.064206032
Sri	-0.115229814	0.862569243	0.064206032
Tenm3	0.037670005	0.862700765	0.064139817
Dcps	0.051726366	0.862780672	0.064099592
Fen1	0.032235663	0.863034874	0.063971655
Ddx27	0.083178103	0.863396556	0.063789688
Usp10	-0.066474063	0.863396556	0.063789688
Scaf1	-0.106544663	0.863625361	0.063674613
Zfp157	-0.059739437	0.863625361	0.063674613
Mycbp	-0.137019544	0.863625361	0.063674613
Cdc42bpb	0.067006158	0.863625361	0.063674613
Mtmr4	-0.109410165	0.863795617	0.063589004
Atxn7l2	-0.0959579	0.863795617	0.063589004
Dusp10	0.059911706	0.863795617	0.063589004
Elp5	0.097805942	0.863795617	0.063589004
Obscn	-0.049320479	0.863795617	0.063589004
Slc8a3	-0.045953696	0.863795617	0.063589004
Stk38	-0.07172823	0.863795617	0.063589004
Commd4	0.081245713	0.863795617	0.063589004
Has3	-0.055504119	0.863795617	0.063589004
Tmem127	-0.092263504	0.863795617	0.063589004
Kdm1b	0.077124745	0.863795617	0.063589004
WBSCR16	-0.125886965	0.863795617	0.063589004
Nlrp4f	0.031471836	0.863795617	0.063589004
L3mbtl3	-0.12638418	0.863926036	0.063523437
Cfl1	0.123918297	0.863926036	0.063523437
Pnma2	0.068016493	0.863926036	0.063523437
DXBAY18	-0.089066258	0.863926036	0.063523437
Wnk4	0.134215928	0.863926036	0.063523437
Rpph1	-0.072692083	0.863926036	0.063523437
Tspan2	0.057207298	0.863926036	0.063523437
Spop	-0.099191321	0.863926036	0.063523437
Slc11a2	-0.151532233	0.863926036	0.063523437
Rps3	-0.059139831	0.863926036	0.063523437

Cd209d	-0.051073048	0.863926036	0.063523437
Armcx2	0.046451145	0.863926036	0.063523437
Creb1	0.099022436	0.863926036	0.063523437
B9d2	0.05451102	0.864000063	0.063486226
Psmb6	-0.084813332	0.864073413	0.063449358
Ssu2	0.04335313	0.864073413	0.063449358
Inpp5e	-0.117158652	0.864073413	0.063449358
Cdk2ap1	0.12718736	0.864073413	0.063449358
Rab39b	0.090015986	0.864073413	0.063449358
Nlgn3	0.064781212	0.864073413	0.063449358
Gfod2	0.144238919	0.864073413	0.063449358
Nop58	-0.156472706	0.864120662	0.063425611
Atp6v1d	-0.072918673	0.864120662	0.063425611
Jakmip3	-0.106765981	0.864120662	0.063425611
Zdhhc9	-0.220979454	0.864120662	0.063425611
Tmem160	-0.110011943	0.864120662	0.063425611
Haus3	-0.135927077	0.864120662	0.063425611
Jkamp	-0.06943405	0.864120662	0.063425611
FAM46B	0.063462169	0.864120662	0.063425611
Fchsd2	0.116088345	0.864120662	0.063425611
Leo1	-0.044713482	0.864120662	0.063425611
Dtl	0.155236094	0.864120662	0.063425611
Sumf2	0.094230912	0.864120662	0.063425611
Ushbp1	-0.065970864	0.864120662	0.063425611
Shmt2	0.05127588	0.864120662	0.063425611
Eya2	-0.068244092	0.864120662	0.063425611
Sycp2l	0.075139272	0.864120662	0.063425611
Trpm2	-0.084541303	0.864120662	0.063425611
Vps25	0.060196635	0.864120662	0.063425611
Mxra7	-0.089545555	0.864120662	0.063425611
Cd79a	-0.061688751	0.864120662	0.063425611
Cgref1	0.054897889	0.864120662	0.063425611
Fhl1	-0.040220962	0.864120662	0.063425611
Mapk14	-0.053996873	0.864120662	0.063425611
Zfp53	0.049934646	0.864120662	0.063425611
Ralbp1	0.094359076	0.864120662	0.063425611
FAM195A	-0.080700818	0.864120662	0.063425611
Colec11	-0.152149725	0.864120662	0.063425611
Mpv17l	-0.064120889	0.864120662	0.063425611
Pcid2	-0.05421466	0.864120662	0.063425611
Esrra	0.07525976	0.864120662	0.063425611
Luc7l	-0.088188291	0.864120662	0.063425611
Chst9	-0.105528379	0.864120662	0.063425611
Gng3	0.033750034	0.864120662	0.063425611
Sema3e	-0.072522192	0.864120662	0.063425611
Mtag2	0.042813345	0.864120662	0.063425611
Pcdhga5	-0.101919793	0.864165246	0.063403204
Tas2r123	-0.085487706	0.864165246	0.063403204

Armcx5	0.114913714	0.864165246	0.063403204
Qprt	-0.098160636	0.864236522	0.063367385
Tspan7	-0.091294776	0.864236522	0.063367385
Syne4	0.122840997	0.864236522	0.063367385
Ifna2	0.161567703	0.864236522	0.063367385
Pnrc1	-0.122145002	0.864236522	0.063367385
Ddx1	0.357086389	0.864236522	0.063367385
Fbxo46	-0.126941077	0.864236522	0.063367385
Afmid	0.052556515	0.864236522	0.063367385
Polr3c	-0.099124661	0.864236522	0.063367385
Cpsf7	0.064854347	0.864236522	0.063367385
Ap5s1	0.108842318	0.864236522	0.063367385
Amer2	0.053877366	0.864236522	0.063367385
Fam181a	0.050111078	0.864236522	0.063367385
Elof1	-0.068587488	0.864236522	0.063367385
Acad9	-0.047114675	0.864236522	0.063367385
PLIN2_1	-0.17644419	0.864332717	0.063319048
Ppp1r14d	0.053042856	0.864332717	0.063319048
Itih2	0.054047787	0.864332717	0.063319048
Gprc5c	0.068687352	0.864332717	0.063319048
Slc15a1	0.092630825	0.864332717	0.063319048
Omg	0.068186178	0.864332717	0.063319048
Clic5	0.101450751	0.864332717	0.063319048
Fbxo24	-0.12359222	0.864332717	0.063319048
Tex2	0.123351753	0.864332717	0.063319048
Klhl14	0.267569247	0.864332717	0.063319048
Macf1	-0.052958989	0.864332717	0.063319048
Mdm1	-0.091784983	0.864332717	0.063319048
Ets2	0.096635868	0.864332717	0.063319048
Atp6ap2	0.049412733	0.864332717	0.063319048
Itih4	0.032993974	0.864332717	0.063319048
Ltn1	-0.056666183	0.864332717	0.063319048
Vps4a	0.060253966	0.864332717	0.063319048
Chia1	0.059963303	0.864332717	0.063319048
Sdc2	-0.076504394	0.864332717	0.063319048
Haghl	0.084140643	0.864332717	0.063319048
Tspyl2	0.04227151	0.864332717	0.063319048
Adck1	-0.049831441	0.864487637	0.063241213
Zfp286os	0.06133866	0.864571693	0.063198988
Rimbp3	-0.08503755	0.864571693	0.063198988
Bcl2l15	-0.066381355	0.864571693	0.063198988
Polr3e	0.042149716	0.864973715	0.06299709
Coq10a	-0.22026639	0.865068035	0.062949735
Tpbg	0.28766446	0.865068035	0.062949735
Rbmx2	-0.067897391	0.865475666	0.062745139
Tm6sf2	0.304674421	0.865541149	0.06271228
Atg9b	-0.064093914	0.865541149	0.06271228
Tet1	-0.166896757	0.865541149	0.06271228

L2hgdh	0.112490299	0.865550184	0.062707747
Cited2	-0.040708416	0.865550184	0.062707747
Ip6k1	-0.043067631	0.865688983	0.062638109
Smdt1	-0.048261796	0.866042428	0.062460831
Nr3c2	0.052491153	0.866059718	0.062452161
Rrnad1	-0.142939191	0.866183417	0.062390135
Imp4	-0.138104867	0.866183417	0.062390135
Rps6ka3	-0.113303944	0.866183417	0.062390135
Axin1	0.081758892	0.866183417	0.062390135
Maged2	0.067345498	0.866183417	0.062390135
Mn1	-0.115082637	0.866183417	0.062390135
Gopc	-0.074258009	0.866183417	0.062390135
HIST1H2BN	-0.071987626	0.866183417	0.062390135
Acsbg2	-0.093861082	0.866183417	0.062390135
Tbc1d22b	0.052507224	0.866183417	0.062390135
Serpinb1c	-0.08051223	0.866183417	0.062390135
SYNJ2BP-C	0.037122905	0.866183417	0.062390135
EPT1	-0.037557411	0.866183417	0.062390135
Ndufs6	0.055865079	0.866183417	0.062390135
Ak1	0.041908319	0.866183417	0.062390135
Dnajc19	-0.124336591	0.866353171	0.062305031
Unc45a	-0.111671744	0.866561586	0.062200567
Miip	0.078141055	0.866561586	0.062200567
Nudt19	0.049618074	0.867306357	0.06182747
Kcnk10	0.098434788	0.867306357	0.06182747
Hnrnph2	-0.069985698	0.867306357	0.06182747
Ghr	0.049304836	0.867306357	0.06182747
Nepro	0.052690482	0.867306357	0.06182747
Slc35a2	-0.063144139	0.867306357	0.06182747
HIST1H3A	-0.056545211	0.867306357	0.06182747
Acmsd	-0.085696479	0.867447222	0.06175694
Hbs1l	0.060297476	0.867447222	0.06175694
Prrxl1	-0.201570921	0.86767275	0.061644042
Tnnt2	-0.142927389	0.86767275	0.061644042
Sp1	0.065830014	0.86767275	0.061644042
Rgs5	-0.055827628	0.867686895	0.061636962
Ptpre	-0.084865619	0.867800431	0.061580138
Hnrnpul1	0.13193341	0.867800431	0.061580138
Myadm	-0.169696847	0.868023347	0.061468594
Zfp420	0.114060705	0.868023347	0.061468594
Apbb3	0.076307252	0.868023347	0.061468594
Psma4	-0.060597412	0.868023347	0.061468594
Bcl2l13	0.075409251	0.868023347	0.061468594
Olfr1396	-0.131563077	0.868023347	0.061468594
Fnta	0.042838106	0.868023347	0.061468594
Dlst	0.047977392	0.868023347	0.061468594
Pex14	-0.070180741	0.868082874	0.061438812
Prkci	0.041325564	0.868237759	0.061361331

Birc7	0.089550419	0.868380496	0.061289939
Mtg1	-0.095609644	0.868380496	0.061289939
Ankrd31	0.076080638	0.868380496	0.061289939
Slc25a37	0.05905517	0.868380496	0.061289939
Ebpl	0.060715806	0.868380496	0.061289939
B4galt6	0.092664667	0.868380496	0.061289939
Adgrg5	-0.087239815	0.868380496	0.061289939
Myh1	0.049896651	0.868380496	0.061289939
Cog7	0.054253072	0.868380496	0.061289939
Prkar1a	-0.052277111	0.868380496	0.061289939
Slc1a2	0.044457306	0.868380496	0.061289939
Tmem14c	-0.07709135	0.868380496	0.061289939
Fndc7	-0.102178892	0.868394958	0.061282707
Smu1	-0.046054163	0.868806525	0.061076926
Ifrd1	0.07257595	0.869090848	0.060934824
Ephb4	-0.053319964	0.869118542	0.060920984
Wbscr25	0.14547952	0.869118542	0.060920984
Marveld2	-0.156675485	0.869118542	0.060920984
Snw1	0.051496572	0.869118542	0.060920984
Diaph2	0.067672139	0.869118542	0.060920984
Ndrg1	0.059703046	0.869118542	0.060920984
Ccdc106	0.130282898	0.869118542	0.060920984
Ppp2r5b	-0.051008724	0.869118542	0.060920984
Cap1	0.166076291	0.869118542	0.060920984
Srp14	-0.058705955	0.869118542	0.060920984
Crkl	-0.076376991	0.869118542	0.060920984
Cda	0.074490984	0.869118542	0.060920984
Fat4	-0.044270661	0.869118542	0.060920984
Trim45	0.134801057	0.869118542	0.060920984
Ribc1	0.093427271	0.869118542	0.060920984
Esf1	0.063328438	0.869118542	0.060920984
Pdrg1	0.073726153	0.869118542	0.060920984
Tcerg1l	0.037825996	0.869319083	0.060820787
Rhobtb3	0.07702438	0.869529	0.060715929
Gpr174	-0.138217204	0.869529	0.060715929
Slc18a2	0.113527537	0.869529	0.060715929
CPSF3L	0.08078627	0.869529	0.060715929
Tstd2	-0.071593611	0.869719494	0.060620796
Hmgn3	0.041801487	0.869719494	0.060620796
FGFR1OP	0.043829872	0.869719494	0.060620796
Mettl21a	0.055198293	0.869719494	0.060620796
Guk1	0.113166262	0.869746966	0.060607077
Rprd1b	-0.057447786	0.869746966	0.060607077
Wnk1	-0.088591437	0.869885928	0.060537695
Rrp7a	0.105735446	0.869885928	0.060537695
Fdxr	-0.045790244	0.869885928	0.060537695
BC04976[illegible]	0.046031013	0.869885928	0.060537695
Pus7	-0.06555197	0.869885928	0.060537695

Ghrh	-0.060771566	0.86997258	0.060494435
Armcx3	-0.288666286	0.870142741	0.060409498
Prpf31	-0.135197603	0.870142741	0.060409498
Eid1	-0.050615656	0.870179831	0.060390987
Mme	0.055043391	0.870307309	0.060327369
Slc7a6os	0.056840742	0.870422377	0.060269952
Grsf1	-0.168187892	0.870490371	0.060236029
Lysmd2	0.110821804	0.870490371	0.060236029
Snhg14	-0.055619973	0.870490371	0.060236029
Pou1f1	0.110720477	0.870490371	0.060236029
Mapk8ip1	0.032441465	0.870490371	0.060236029
Skil	0.057852075	0.870490371	0.060236029
Xkr4	0.100842959	0.870490371	0.060236029
Ndufb6	0.057312984	0.870490371	0.060236029
TLDC1	0.064466583	0.870490371	0.060236029
Trmo	0.04892174	0.870490371	0.060236029
Nkx2-2os	-0.055263913	0.870490371	0.060236029
Aqp2	-0.049008782	0.870490371	0.060236029
Pex2	0.057158107	0.870490371	0.060236029
Arl1	-0.049893786	0.870490371	0.060236029
Zfyve26	-0.047619973	0.870490371	0.060236029
Stkld1	-0.040502612	0.870490371	0.060236029
Pkdrej	-0.040483829	0.870490371	0.060236029
Ptprf	0.188139595	0.870490371	0.060236029
Hipk3	-0.072390536	0.870490371	0.060236029
Erp27	0.074215656	0.870490371	0.060236029
Sp3os	0.073012449	0.870490371	0.060236029
Iws1	0.17924992	0.870490371	0.060236029
Mns1	0.040573855	0.870490371	0.060236029
Klf13	0.040449729	0.870490371	0.060236029
Coa3	0.040410023	0.870490371	0.060236029
Snta1	-0.161236525	0.87049658	0.060232931
Snrnp200	0.03172025	0.870582986	0.060189825
LRRC48	0.040332877	0.870582986	0.060189825
Ccnh	-0.048732664	0.870680296	0.060141284
Dnajc11	0.050931881	0.870760424	0.060101318
Gps1	0.087899835	0.870937068	0.060013225
Smg9	0.093200299	0.870937068	0.060013225
Mfsd11	0.047216644	0.870937068	0.060013225
Aco2	-0.117174535	0.87099766	0.059983012
Mplkip	0.115041351	0.87099766	0.059983012
Hook3	0.06332963	0.87099766	0.059983012
Itgav	-0.123845268	0.87099766	0.059983012
Araf	-0.161032993	0.87099766	0.059983012
Cdc5l	0.067614278	0.87099766	0.059983012
Ywhab	0.092085683	0.87099766	0.059983012
Arl4d	0.08983881	0.87099766	0.059983012
Cic	-0.039953879	0.87099766	0.059983012

Retnlb	0.18536844	0.87099766	0.059983012
Alx1	-0.147208714	0.87099766	0.059983012
Pdzd8	-0.101175036	0.87099766	0.059983012
Aimp1	0.038889802	0.87099766	0.059983012
Ankhd1	-0.045236415	0.87099766	0.059983012
Mybpc3	-0.040084769	0.87099766	0.059983012
Tpgs2	0.081137368	0.87099766	0.059983012
Cfap206	0.050785919	0.87099766	0.059983012
Rell1	0.040081874	0.87099766	0.059983012
Kif13a	0.183637441	0.87111914	0.059922444
Trim34b	0.045133014	0.871138023	0.05991303
Kcnj4	0.041162775	0.871138023	0.05991303
Aars	-0.055814783	0.871138023	0.05991303
Hs3st3b1	0.062956787	0.871138023	0.05991303
Tnfrsf17	0.059274588	0.871138023	0.05991303
Mrs2	0.265885505	0.871138023	0.05991303
Slitrk2	0.068414052	0.871138023	0.05991303
FAM73A	0.062380197	0.871138023	0.05991303
Pcf11	0.084534531	0.871138023	0.05991303
Rnf114	-0.073063924	0.871138023	0.05991303
Ube3b	-0.04852532	0.871138023	0.05991303
Rnf113a2	0.117906067	0.871138023	0.05991303
Snhg10	-0.039987951	0.871138023	0.05991303
Mrpl48	-0.096209304	0.871138023	0.05991303
ERO1L	-0.039938928	0.871138023	0.05991303
P2rx1	0.061575654	0.871138023	0.05991303
Myoz1	0.046724124	0.871138023	0.05991303
Pdss2	-0.067884217	0.871138023	0.05991303
Atg2a	-0.032020627	0.871138023	0.05991303
Kat2a	0.056849625	0.871414302	0.059775317
Med17	0.241984095	0.871549406	0.059707989
Ppp2r5c	0.081713918	0.871549406	0.059707989
Spcs2	0.064896044	0.871549406	0.059707989
Chrm5	0.086003328	0.871549406	0.059707989
Nat8f1	-0.043151473	0.871549406	0.059707989
Mia3	-0.049489247	0.871549406	0.059707989
Il18r1	0.109525222	0.871549406	0.059707989
Cldn34c1	0.061992702	0.872098477	0.059434472
Gemin7	0.050597871	0.872098477	0.059434472
Hebp2	0.077116782	0.872098477	0.059434472
Katnb1	0.101692418	0.872098477	0.059434472
Rab11fip2	-0.047036151	0.872098477	0.059434472
Idh3b	0.042074014	0.872098477	0.059434472
Gsta4	0.039690882	0.872098477	0.059434472
Anks1	0.100858657	0.872243068	0.059362473
Ffar4	-0.154062664	0.872341491	0.059313471
Gtf2h5	-0.078028259	0.872341491	0.059313471
Neurl1a	-0.057815482	0.872341491	0.059313471

Fbxo3	0.063483309	0.872699833	0.059135107
Kcnj16	-0.108394932	0.872699833	0.059135107
Inpp5a	-0.12319916	0.872772	0.059099195
Arl14	-0.319656191	0.872772	0.059099195
Prom1	-0.205644592	0.872772	0.059099195
Gripap1	-0.17830084	0.872772	0.059099195
Rad17	-0.10899341	0.872772	0.059099195
Iah1	-0.084865691	0.872772	0.059099195
Uqcc2	0.08652244	0.872772	0.059099195
Zfyve21	-0.078711349	0.872772	0.059099195
Ecm2	-0.068829362	0.872772	0.059099195
Cox7b	-0.145737965	0.872772	0.059099195
Zc3hc1	-0.058474294	0.872772	0.059099195
Gtf2f1	0.133102563	0.872772	0.059099195
Nktr	-0.133050866	0.872772	0.059099195
Smap1	-0.101113817	0.872772	0.059099195
Mllt11	-0.085081974	0.872772	0.059099195
Egfr	0.067601954	0.872772	0.059099195
Napepld	-0.084730223	0.872772	0.059099195
Krt14	-0.065464864	0.872772	0.059099195
Mcidas	0.098782572	0.872772	0.059099195
Mxd4	0.060685233	0.872772	0.059099195
Dmtf1	0.097268395	0.872772	0.059099195
Ccdc9	0.203090059	0.872772	0.059099195
Sfmbt1	0.099842291	0.872772	0.059099195
Ccdc12	0.074238372	0.872772	0.059099195
Piwil4	-0.049489775	0.872772	0.059099195
HIST1H2BI	-0.082805365	0.872772	0.059099195
BC065397	-0.082878988	0.872772	0.059099195
Sh3gl1	-0.075875839	0.872772	0.059099195
Spata2	-0.046284279	0.872772	0.059099195
Plpp7	0.071025411	0.872772	0.059099195
Fam83c	-0.049877657	0.872772	0.059099195
Rpl37a	0.052283951	0.872772	0.059099195
Acoxl	-0.071059532	0.872772	0.059099195
Kif9	-0.066307022	0.872772	0.059099195
Cd200	-0.065473754	0.872772	0.059099195
Ehd3	0.089518014	0.872772	0.059099195
Hook1	0.061912533	0.872772	0.059099195
Klrb1a	0.083985506	0.872772	0.059099195
Cacna1d	0.136968127	0.872772	0.059099195
Mir155hg	0.143104124	0.872772	0.059099195
Myom3	0.069217746	0.872772	0.059099195
Lat	0.047735033	0.872772	0.059099195
Grpel2	-0.039772349	0.872772	0.059099195
BC021614	-0.068447499	0.872772	0.059099195
Tbp	0.059007401	0.872772	0.059099195
Zfp61	0.05593573	0.872772	0.059099195

Fndc8	0.05701136	0.872772	0.059099195
Dctn3	-0.071773059	0.872772	0.059099195
Otud1	0.078746778	0.872772	0.059099195
Astn1	0.031643247	0.872772	0.059099195
Klhdc8a	0.139161788	0.872772	0.059099195
Snca	0.069424761	0.872772	0.059099195
Dhx8	0.123843739	0.872772	0.059099195
Stk24	0.045398029	0.872772	0.059099195
CCDC114	0.049024756	0.872772	0.059099195
Atp5a1	0.099452779	0.872772	0.059099195
Ccdc120	0.238963382	0.872772	0.059099195
Ankrd52	-0.055402817	0.872772	0.059099195
Cblb	0.065581292	0.872772	0.059099195
Trpc1	-0.078489668	0.872772	0.059099195
Glt1d1	-0.035737274	0.872772	0.059099195
Rad23b	0.084189444	0.872772	0.059099195
Nat10	-0.042986385	0.872772	0.059099195
Hdac6	-0.072536488	0.872772	0.059099195
Psmb2	-0.102642796	0.872772	0.059099195
Gpr45	-0.109883839	0.872772	0.059099195
Osbpl9	-0.043694336	0.872772	0.059099195
Ccdc107	-0.072171507	0.872772	0.059099195
HIST1H2BI	0.060980093	0.872772	0.059099195
C4bp	-0.039447633	0.872772	0.059099195
Spice1	-0.03893129	0.872772	0.059099195
Slu7	-0.038906177	0.872772	0.059099195
Chd3	0.063608665	0.872772	0.059099195
Bmp7	-0.070707486	0.872772	0.059099195
Btbd2	-0.117960619	0.872772	0.059099195
TTC25	0.05390617	0.872772	0.059099195
Bud31	-0.081583821	0.872772	0.059099195
Pomgnt1	0.100106653	0.872772	0.059099195
BC031361	0.098651042	0.872772	0.059099195
Agap3	0.083960266	0.872772	0.059099195
Srsf6	0.066229589	0.872772	0.059099195
Gstp2	0.092773115	0.872772	0.059099195
Upk3bl	0.099683721	0.872772	0.059099195
Ptpa	0.049392876	0.872772	0.059099195
B4galt5	0.105631729	0.872772	0.059099195
Nsun5	-0.083667879	0.872772	0.059099195
Camkv	-0.042105245	0.872772	0.059099195
Dhx57	0.044584518	0.872772	0.059099195
FAM160B2	0.03934084	0.872772	0.059099195
Asb4	0.039249062	0.872772	0.059099195
Uxs1	0.039036754	0.872772	0.059099195
Rbm15	0.038883624	0.872772	0.059099195
Ddx23	0.038864548	0.872772	0.059099195
Crtc3	0.038712847	0.872772	0.059099195

Erich6	0.064929353	0.872781499	0.059094469
Cdc37l1	0.063689848	0.872866082	0.059052382
Pms2	0.171076679	0.873012628	0.058979474
Rdh14	-0.063757231	0.873012628	0.058979474
Sowaha	-0.059725946	0.873012628	0.058979474
Fkrp	0.079855014	0.873154996	0.058908656
Jph1	0.081492191	0.873154996	0.058908656
Orc4	-0.090890572	0.873154996	0.058908656
Arpc5l	0.076402848	0.873154996	0.058908656
Ap1g1	-0.038580894	0.873371541	0.058800964
Wdr59	-0.145328283	0.873425118	0.058774323
Lpin2	-0.088455821	0.873425118	0.058774323
Snx27	-0.06340498	0.873425118	0.058774323
Ssbp3	0.049599173	0.873425118	0.058774323
Ddt	-0.103164372	0.873425118	0.058774323
Zfp266	-0.089578149	0.873425118	0.058774323
Nxph4	0.070552417	0.873425118	0.058774323
Pebp4	-0.046361508	0.873425118	0.058774323
Bscl2	-0.079591019	0.873425118	0.058774323
Jarid2	0.066548978	0.873570208	0.058702186
Klhl11	-0.065142272	0.873631876	0.058671528
Iars	0.145287787	0.873631876	0.058671528
Olfr1510	0.069287738	0.873631876	0.058671528
Zfp958	0.038445352	0.873631876	0.058671528
Tmem192	-0.102350662	0.87385388	0.058561181
Cstad	-0.038405933	0.87385388	0.058561181
Kcnk5	0.06875615	0.874076601	0.058450506
Gpc6	-0.046819258	0.874076601	0.058450506
Msantd3	-0.177728452	0.874076601	0.058450506
Col25a1	-0.056598004	0.874076601	0.058450506
Pogk	0.053153446	0.874076601	0.058450506
MURC	-0.056814746	0.874076601	0.058450506
Cdc7	0.044390342	0.874076601	0.058450506
Smg8	-0.033109407	0.874076601	0.058450506
Dmrta1	-0.038291389	0.874238956	0.058369845
Adgrl3	0.040927805	0.874238956	0.058369845
BC10617[illegible]	-0.044884209	0.874919036	0.058032134
Rsph1	0.12621398	0.874945261	0.058019117
Lin7c	0.056715316	0.87495516	0.058014203
Rlbp1	0.10294982	0.87495516	0.058014203
Fpgt	-0.075010087	0.87495516	0.058014203
Snx22	-0.047496354	0.875080788	0.057951851
Cox5a	0.062242083	0.875173187	0.057905996
Dtx1	0.070590695	0.875223754	0.057880904
Grhl3	0.100256501	0.875223754	0.057880904
Fat1	0.16070534	0.875223754	0.057880904
Gaa	-0.069369369	0.875223754	0.057880904
Rragc	0.064557233	0.875223754	0.057880904

Sema6b	-0.06841263	0.875223754	0.057880904
Noc4l	-0.081644432	0.875223754	0.057880904
Wdr17	0.048116993	0.875223754	0.057880904
Dcun1d4	-0.083159661	0.875223754	0.057880904
Lgr4	-0.038091033	0.875223754	0.057880904
Jsrp1	-0.038064341	0.875223754	0.057880904
Cep41	0.067504701	0.875223754	0.057880904
Chit1	0.136913914	0.875260617	0.057862612
Haao	-0.038026762	0.875408519	0.057789231
Sars	-0.071300442	0.875420707	0.057783185
Grip1os2	-0.038010729	0.875420707	0.057783185
Sigirr	-0.047898848	0.875482063	0.057752748
Sis	0.050228184	0.875482063	0.057752748
Runx1t1	0.062732289	0.875482063	0.057752748
Fez2	-0.112874234	0.875553342	0.05771739
Cenpb	-0.13361383	0.875553342	0.05771739
Mstn	0.048257034	0.875553342	0.05771739
Dlx1	0.084523437	0.875553342	0.05771739
Ttc21a	0.052531341	0.875553342	0.05771739
Slc4a7	0.062254009	0.875553342	0.05771739
Dolk	0.04611156	0.875553342	0.05771739
Ube2m	-0.052283763	0.875777148	0.057606391
Dohh	-0.054494232	0.875777148	0.057606391
DFNA5	-0.085827674	0.875816019	0.057587116
Pstk	0.075195709	0.875816019	0.057587116
Zfp260	-0.122175882	0.875816019	0.057587116
RTFDC1	-0.102613516	0.875816019	0.057587116
Atad5	-0.034380028	0.875816019	0.057587116
Tsnaxip1	-0.096682463	0.875816019	0.057587116
Rassf5	0.111627836	0.875883108	0.057553849
Zfp524	0.138095536	0.875883108	0.057553849
Cept1	0.164129778	0.875883108	0.057553849
Rps3a1	-0.079363095	0.875883108	0.057553849
Mmp16	-0.083197881	0.875883108	0.057553849
Taf6l	0.102390237	0.875883108	0.057553849
Smarcal1	0.037273422	0.875883108	0.057553849
Tmem185	0.147961836	0.875883108	0.057553849
Usp4	0.13246113	0.875883108	0.057553849
Zscan21	0.082680135	0.875883108	0.057553849
Mettl4	-0.103921157	0.875883108	0.057553849
HIST1H2AK	0.067926271	0.875883108	0.057553849
Cldn8	0.041165954	0.875883108	0.057553849
Samd3	-0.059410198	0.875883108	0.057553849
Rassf1	-0.042872687	0.875883108	0.057553849
Atp2b4	-0.124606829	0.875883108	0.057553849
Ucp1	0.053024839	0.875883108	0.057553849
Snhg3	-0.05826078	0.875883108	0.057553849
Tmem238	0.0640949	0.875883108	0.057553849

Coq5	0.115280494	0.875883108	0.057553849
Alms1	0.04402931	0.875883108	0.057553849
Olfr545	0.037646909	0.875883108	0.057553849
Clta	0.102767752	0.875913693	0.057538684
Acnat1	0.052608076	0.875913693	0.057538684
Megf6	0.064376664	0.875913693	0.057538684
Gsk3b	-0.061313487	0.875923443	0.05753385
Exoc3	-0.063721552	0.875923443	0.05753385
Ndufa4	0.393934211	0.875923443	0.05753385
Otud3	0.097167877	0.875923443	0.05753385
Nutf2	-0.052469925	0.875923443	0.05753385
Tpcn1	0.168257886	0.875923443	0.05753385
Atxn7l1	-0.040383557	0.875923443	0.05753385
Avil	-0.048609755	0.875923443	0.05753385
Ctnna3	-0.055718143	0.875923443	0.05753385
Igf1os	-0.068345178	0.875923443	0.05753385
Fjx1	-0.039177365	0.875923443	0.05753385
Ice1	0.054182426	0.875923443	0.05753385
Fam122b	0.069879634	0.875923443	0.05753385
Sde2	-0.072981835	0.875923443	0.05753385
Evpl	0.046845964	0.875923443	0.05753385
Cnot6	0.049984134	0.876006465	0.057492689
Bpifa1	0.079558554	0.876006465	0.057492689
Zfp955b	0.069677354	0.876006465	0.057492689
Proser3	0.045894593	0.876006465	0.057492689
FAM132A	-0.090620293	0.876031495	0.05748028
BE949265	-0.037419081	0.876031495	0.05748028
Aamdc	-0.037419081	0.876031495	0.05748028
Ppp2r5d	-0.265711116	0.876355545	0.057319661
Egfl7	-0.116984049	0.876355545	0.057319661
Camk2d	-0.067791755	0.876355545	0.057319661
Pcdhb19	-0.196453724	0.876355545	0.057319661
Wdr48	-0.088833864	0.876355545	0.057319661
Cyp1a1	-0.215252865	0.876355545	0.057319661
Dnah9	0.205267422	0.876355545	0.057319661
Myo18a	0.163424985	0.876355545	0.057319661
Reln	-0.119445366	0.876355545	0.057319661
Bcdin3d	0.08941079	0.876355545	0.057319661
Gpr1	-0.282687822	0.876355545	0.057319661
Atxn2l	-0.049536649	0.876355545	0.057319661
Perp	-0.118074088	0.876355545	0.057319661
C86187	-0.06120526	0.876355545	0.057319661
Masp2	-0.063805694	0.876355545	0.057319661
Cep350	-0.058496259	0.876355545	0.057319661
Cryl1	-0.11613202	0.876355545	0.057319661
Grem2	-0.124181143	0.876355545	0.057319661
Zc3h3	0.078799073	0.876355545	0.057319661
Majin	-0.076199399	0.876355545	0.057319661

Mtmr9	0.108872859	0.876355545	0.057319661
Gkap1	0.057346988	0.876355545	0.057319661
Nipa1	0.067416636	0.876355545	0.057319661
Scn9a	-0.048227468	0.876355545	0.057319661
Epha5	-0.105433064	0.876355545	0.057319661
Zp3r	-0.044823249	0.876355545	0.057319661
Mrps24	-0.041988813	0.876355545	0.057319661
Esrp2	0.090280461	0.876355545	0.057319661
Unk	0.100218152	0.876355545	0.057319661
Popdc2	0.063783877	0.876355545	0.057319661
Mboat2	-0.062173262	0.876355545	0.057319661
Trim62	0.055231261	0.876355545	0.057319661
Rbak	0.130946802	0.876355545	0.057319661
Msl1	0.047260292	0.876355545	0.057319661
Eno1b	-0.072843727	0.876355545	0.057319661
Paqr5	0.10367449	0.876355545	0.057319661
Kcnip3	0.047641848	0.876355545	0.057319661
Snhg6	-0.08397274	0.876355545	0.057319661
IMPAD1	0.094271731	0.876355545	0.057319661
Nrbp1	-0.058000687	0.876355545	0.057319661
Gfm1	0.103418244	0.876355545	0.057319661
FAM69C	0.091212413	0.876355545	0.057319661
Aktip	-0.066765798	0.876355545	0.057319661
Casp8ap2	0.07270891	0.876355545	0.057319661
Itga3	-0.084580541	0.876355545	0.057319661
Syt3	0.043088935	0.876355545	0.057319661
Xrcc4	-0.049279167	0.876355545	0.057319661
Bin1	-0.058113363	0.876355545	0.057319661
Krt25	0.061795189	0.876355545	0.057319661
Poli	0.086667797	0.876355545	0.057319661
NGFRAP1	-0.049054096	0.876355545	0.057319661
Gys2	-0.045378234	0.876355545	0.057319661
Dgkd	-0.041503941	0.876355545	0.057319661
Slc35a3	-0.064671023	0.876355545	0.057319661
Dhh	0.040112256	0.876355545	0.057319661
Utp23	-0.044644395	0.876355545	0.057319661
Wdr24	-0.049329784	0.876355545	0.057319661
Tarsl2	0.050579945	0.876355545	0.057319661
Plag1	-0.037309391	0.876355545	0.057319661
Uvssa	-0.036919571	0.876355545	0.057319661
Tns2	-0.036912643	0.876355545	0.057319661
Slc39a14	-0.036862918	0.876355545	0.057319661
Plxna3	0.068421735	0.876355545	0.057319661
H2-M5	-0.028746804	0.876355545	0.057319661
Myo9b	0.039896159	0.876355545	0.057319661
Swi5	0.059567262	0.876355545	0.057319661
Pag1	-0.037280597	0.876355545	0.057319661
Pvt1	-0.032119523	0.876355545	0.057319661

Pofut2	-0.053298421	0.876355545	0.057319661
G2e3	0.055151589	0.876355545	0.057319661
Cisd2	0.062867678	0.876355545	0.057319661
Ablim2	0.037276044	0.876355545	0.057319661
Rpa3	0.071817492	0.876698865	0.057149556
Haus7	-0.036759703	0.876857184	0.057071136
Tvp23bos	0.09490547	0.876951238	0.057024554
Efcc1	-0.125905206	0.877072284	0.056964613
Ssbp4	-0.124815026	0.877072284	0.056964613
Tekt1	-0.074706589	0.877072284	0.056964613
Zfp703	-0.227614549	0.877072284	0.056964613
Zfp128	-0.073060018	0.877072284	0.056964613
Trpm8	0.051612864	0.877072284	0.056964613
Gata3	-0.068686638	0.877072284	0.056964613
Tfr2	0.048396033	0.877072284	0.056964613
Uts2r	0.0972557	0.877072284	0.056964613
Hcfc1	0.058457052	0.877072284	0.056964613
Tesk1	0.042327706	0.877072284	0.056964613
Tectb	-0.120518903	0.877072284	0.056964613
Armc8	-0.092361553	0.877072284	0.056964613
Cep126	0.058065432	0.877072284	0.056964613
Hgs	0.068640182	0.877072284	0.056964613
Rap2c	-0.066388994	0.877072284	0.056964613
Trabd	-0.053132331	0.877072284	0.056964613
Nudt12	0.062865619	0.877072284	0.056964613
FAM19A3	-0.050988846	0.877072284	0.056964613
Wars	-0.059993001	0.877072284	0.056964613
Acot13	-0.089878201	0.877072284	0.056964613
Acat2	-0.065727019	0.877072284	0.056964613
Zdhhc14	0.038735663	0.877072284	0.056964613
Rnf44	0.072463966	0.877072284	0.056964613
Ifnar1	0.054190813	0.877072284	0.056964613
Gimap7	0.062159459	0.877072284	0.056964613
B3gnt6	-0.090341178	0.877072284	0.056964613
H2-T-ps	-0.115411347	0.877072284	0.056964613
Erich2	-0.06750351	0.877072284	0.056964613
Polr2e	0.062829637	0.877072284	0.056964613
Patz1	0.050049766	0.877072284	0.056964613
Ammecr1l	-0.123006453	0.877072284	0.056964613
Xntrpc	0.033002836	0.877072284	0.056964613
Mrps18c	0.052238237	0.877072284	0.056964613
Rtkn	0.092437802	0.877072284	0.056964613
Nox3	0.060388339	0.877072284	0.056964613
Mphosph9	0.092796699	0.877072284	0.056964613
Btnl2	-0.036419794	0.877072284	0.056964613
Etnppl	0.033909668	0.877072284	0.056964613
Map2k6	-0.085715285	0.877072284	0.056964613
Midn	0.077877665	0.877072284	0.056964613

BC051142	-0.051519922	0.877072284	0.056964613
Prom2	-0.069602755	0.877072284	0.056964613
Rasgef1c	0.063565219	0.877072284	0.056964613
AU019823	-0.040164502	0.877072284	0.056964613
Serpinb12	0.036604939	0.877072284	0.056964613
Hotairm1	-0.136752525	0.877584487	0.056711062
Lipf	0.038875658	0.877584487	0.056711062
Stard3	-0.045893157	0.877584487	0.056711062
Tuba3a	-0.274052482	0.877749421	0.056629448
Tm4sf19	0.064927186	0.877749421	0.056629448
Zfp971	-0.135701413	0.877749421	0.056629448
Slc16a5	0.098061558	0.877749421	0.056629448
Dld	0.075622651	0.87796902	0.056520809
Slc35e1	0.086969699	0.878000322	0.056505325
FOPNL	-0.144133661	0.878000322	0.056505325
Ceacam9	0.069415316	0.878000322	0.056505325
Ano7	-0.036226328	0.878000322	0.056505325
Mzf1	0.041236711	0.878000322	0.056505325
Tbc1d7	-0.071721728	0.87810695	0.056452586
Plvap	0.057025194	0.87810695	0.056452586
TSSC1	-0.049982874	0.87810695	0.056452586
Rxrg	-0.071202505	0.87810695	0.056452586
Klk1	-0.02832099	0.87810695	0.056452586
Snapc3	0.048122578	0.87810695	0.056452586
Lrrc52	-0.102983752	0.87810695	0.056452586
Acbd6	0.093871773	0.87810695	0.056452586
Rsph4a	-0.101841544	0.878129568	0.056441399
Spag8	0.041848081	0.878129568	0.056441399
Bicd2	-0.214499357	0.878190626	0.056411203
Zfp12	-0.161748763	0.878190626	0.056411203
Prss53	0.201879227	0.878190626	0.056411203
Gins4	-0.125029508	0.878190626	0.056411203
Zhx1	-0.178819488	0.878190626	0.056411203
Wnt2	0.15220952	0.878190626	0.056411203
Crebbp	-0.098830602	0.878190626	0.056411203
Cep57l1	0.060729569	0.878190626	0.056411203
Tnks1bp1	-0.072352985	0.878190626	0.056411203
Rs1	0.096135342	0.878190626	0.056411203
Rhpn2	0.121478767	0.878190626	0.056411203
Alpk3	0.116826261	0.878190626	0.056411203
Slc37a3	0.056019936	0.878190626	0.056411203
Adcy5	-0.103523901	0.878190626	0.056411203
AI837181	0.099466827	0.878190626	0.056411203
Tnfrsf4	-0.211672121	0.878190626	0.056411203
Ly6g5b	-0.057183823	0.878190626	0.056411203
Man2a1	0.050752683	0.878190626	0.056411203
Plppr4	-0.058531818	0.878190626	0.056411203
Smtnl2	0.099738731	0.878190626	0.056411203

Nrsn2	-0.072478572	0.878190626	0.056411203
Dpys	0.069548256	0.878190626	0.056411203
Ep400	-0.06742237	0.878190626	0.056411203
Tbc1d15	0.07667059	0.878190626	0.056411203
Nup205	0.049682244	0.878190626	0.056411203
Dis3l2	-0.160380654	0.878190626	0.056411203
Cngb1	-0.057241985	0.878190626	0.056411203
Prob1	0.064527143	0.878190626	0.056411203
Hps1	0.151510688	0.878190626	0.056411203
Plek2	-0.052199504	0.878190626	0.056411203
Plekhm1	0.050285543	0.878190626	0.056411203
Zfp788	0.059866012	0.878190626	0.056411203
Dnajb8	0.073446131	0.878190626	0.056411203
Slain2	0.046845584	0.878190626	0.056411203
Higd1b	-0.040604532	0.878190626	0.056411203
Dcc	0.062331001	0.878190626	0.056411203
Cd300ld2	-0.085165184	0.878190626	0.056411203
Tmem11	0.063496314	0.878190626	0.056411203
Ift74	-0.292740934	0.878190626	0.056411203
Srp68	0.044477929	0.878190626	0.056411203
Kcnk1	-0.049464232	0.878190626	0.056411203
Smg6	0.059608248	0.878190626	0.056411203
Chchd10	0.045001527	0.878190626	0.056411203
SLMO2	0.098774261	0.878190626	0.056411203
Pltp	-0.064220018	0.878190626	0.056411203
Lias	0.075396878	0.878190626	0.056411203
Edar	-0.0480982	0.878190626	0.056411203
Zfp451	-0.057843978	0.878190626	0.056411203
Mon1b	0.065547687	0.878190626	0.056411203
Mbd2	-0.078319212	0.878190626	0.056411203
Tatdn1	0.085980984	0.878190626	0.056411203
Gps2	-0.061218784	0.878190626	0.056411203
Rpusd2	-0.045463408	0.878190626	0.056411203
Sfrp2	0.143133056	0.878190626	0.056411203
Zpr1	0.048210266	0.878190626	0.056411203
Tefm	-0.076787069	0.878190626	0.056411203
Trim69	-0.082542435	0.878190626	0.056411203
Cndp1	-0.08813432	0.878190626	0.056411203
Cdk19	-0.072160256	0.878190626	0.056411203
Xpa	0.041612563	0.878190626	0.056411203
Ikzf2	-0.064875845	0.878190626	0.056411203
Pcdhgb5	0.059196953	0.878190626	0.056411203
Ext1	0.06136923	0.878190626	0.056411203
P2ry10	0.122419441	0.878190626	0.056411203
B9d1	-0.048222548	0.878190626	0.056411203
Bmyc	-0.073849184	0.878190626	0.056411203
Cpsf2	0.054851491	0.878190626	0.056411203
Pde10a	0.133841806	0.878190626	0.056411203

ATP5F1	0.053589892	0.878190626	0.056411203
Ghsr	0.048947747	0.878190626	0.056411203
Speg	-0.064697023	0.878190626	0.056411203
Pgam1	-0.054215707	0.878190626	0.056411203
Tef	-0.032971723	0.878190626	0.056411203
Cldn34c2	-0.095744107	0.878190626	0.056411203
Erlin2	-0.045823964	0.878190626	0.056411203
Mrpl13	0.073575323	0.878190626	0.056411203
Prpf18	-0.058664325	0.878190626	0.056411203
Fam178b	0.030383776	0.878190626	0.056411203
Ccdc172	-0.068108617	0.878190626	0.056411203
Fam193a	0.105987673	0.878190626	0.056411203
Ndufa6	-0.045217842	0.878190626	0.056411203
Ccdc96	0.051163772	0.878190626	0.056411203
Timm17b	0.076195341	0.878190626	0.056411203
Ace	-0.042437853	0.878190626	0.056411203
Bhlha15	0.066485617	0.878190626	0.056411203
Zfp14	-0.043682663	0.878190626	0.056411203
Ube2q1	0.079020849	0.878190626	0.056411203
Kazn	-0.053882973	0.878190626	0.056411203
Lvrn	-0.058616885	0.878190626	0.056411203
Chd8	-0.138591505	0.878190626	0.056411203
Cpvl	0.048162431	0.878190626	0.056411203
Arhgap6	0.064603745	0.878190626	0.056411203
Irs2	-0.070885708	0.878190626	0.056411203
Rundc3b	0.048604772	0.878190626	0.056411203
Sertad3	-0.063919089	0.878190626	0.056411203
Crtac1	0.045786508	0.878190626	0.056411203
Rchy1	0.150011672	0.878190626	0.056411203
Nanos2	-0.054139686	0.878190626	0.056411203
Actn3	-0.064809975	0.878190626	0.056411203
Stambpl1	0.066630873	0.878190626	0.056411203
Rdh11	-0.032194466	0.878190626	0.056411203
Olfr46	-0.053879188	0.878190626	0.056411203
Tob1	0.035340889	0.878190626	0.056411203
NRP	0.046767815	0.878190626	0.056411203
Onecut1	0.035269111	0.878190626	0.056411203
Pias4	0.101045597	0.878190626	0.056411203
Phf20	-0.135776502	0.878190626	0.056411203
Supv3l1	-0.045008527	0.878190626	0.056411203
Sccpdh	0.055536532	0.878190626	0.056411203
Chchd7	-0.053290919	0.878190626	0.056411203
Dtd1	0.045146402	0.878190626	0.056411203
Dgcr6	-0.035864821	0.878190626	0.056411203
Prkcq	-0.03539559	0.878190626	0.056411203
Plekhs1	-0.035372945	0.878190626	0.056411203
Trim16	-0.039633221	0.878190626	0.056411203
LACE1	-0.04687694	0.878190626	0.056411203

Rspo3	-0.044641084	0.878190626	0.056411203
Pcsk1n	0.042420791	0.878190626	0.056411203
Fam124a	-0.056821436	0.878190626	0.056411203
Igf2os	0.08064368	0.878190626	0.056411203
Hinfp	0.034357963	0.878190626	0.056411203
Daxx	0.038501306	0.878190626	0.056411203
Celf1	0.045438696	0.878190626	0.056411203
Telo2	-0.073408346	0.878190626	0.056411203
Scd2	-0.04887471	0.878190626	0.056411203
Acsm5	-0.074586776	0.878190626	0.056411203
Acadsb	0.051193859	0.878190626	0.056411203
Ncaph2	0.045707479	0.878190626	0.056411203
Mirg	0.062237402	0.878190626	0.056411203
Zswim8	-0.067258663	0.878190626	0.056411203
Mrps7	0.046043369	0.878190626	0.056411203
Usp28	0.03600757	0.878190626	0.056411203
Ranbp1	0.035907047	0.878190626	0.056411203
Tbxa2r	0.035702899	0.878190626	0.056411203
Fam25c	0.0356517	0.878190626	0.056411203
Enkur	0.027885436	0.878190626	0.056411203
Cox20	-0.148692956	0.878430085	0.056292799
Prss30	-0.244869067	0.878430085	0.056292799
Adnp2	-0.082430485	0.878430085	0.056292799
Arf6	-0.053669298	0.878430085	0.056292799
Chl1	0.093070916	0.878430085	0.056292799
Sufu	0.051415874	0.878430085	0.056292799
Itk	-0.045925009	0.878430085	0.056292799
Eng	0.074728919	0.878430085	0.056292799
Amer3	-0.086294216	0.878430085	0.056292799
Wdr95	0.047204941	0.878430085	0.056292799
Spg11	-0.098020673	0.878430085	0.056292799
Sohlh1	0.11886187	0.878430085	0.056292799
Mcu	0.074628644	0.878430085	0.056292799
Gad2	-0.121113851	0.878430085	0.056292799
Kank4os	0.051279519	0.878430085	0.056292799
Paxip1	0.040949856	0.878430085	0.056292799
Ebf3	0.038504469	0.878430085	0.056292799
Epb41l4ac	0.091035799	0.878430085	0.056292799
D6Ertd474	0.078922798	0.878430085	0.056292799
Oxsm	-0.074480974	0.878430085	0.056292799
Nsun6	-0.100241714	0.878430085	0.056292799
Jam3	0.035048248	0.878430085	0.056292799
Saa2	0.034935125	0.878430085	0.056292799
Brms1l	0.034905983	0.878430085	0.056292799
Itfg1	0.104015673	0.878463447	0.056276305
Glipr1l1	0.110370294	0.878463447	0.056276305
Gng4	-0.040930205	0.878463447	0.056276305
Ccdc24	0.074778207	0.878463447	0.056276305

Vars	0.058782607	0.878507871	0.056254343
Bcat2	0.073978613	0.878507871	0.056254343
BC049715	0.070936175	0.878507871	0.056254343
Tubb3	-0.067006276	0.87852197	0.056247373
Amot	0.05601394	0.87852197	0.056247373
Zfp358	0.049570113	0.87852197	0.056247373
Golga2	0.083851387	0.87852197	0.056247373
Gtf2e1	-0.067248747	0.878541019	0.056237957
Lrrc2	0.150633928	0.87856731	0.05622496
AU023762	0.103676303	0.87856731	0.05622496
Traf7	-0.053704674	0.879019376	0.056001552
Bpifb4	-0.101466422	0.879019376	0.056001552
Zscan12	0.061224597	0.879019376	0.056001552
Mmab	-0.033016252	0.879019376	0.056001552
Mphosph8	-0.097831658	0.879019376	0.056001552
H3f3c	0.087325403	0.879019376	0.056001552
Gpr137c	0.055723622	0.879019376	0.056001552
Cnot2	0.134874005	0.879019376	0.056001552
Srsf2	-0.061934784	0.879019376	0.056001552
Tbrg1	-0.040953014	0.879019376	0.056001552
Taf6	0.054694764	0.879019376	0.056001552
Spatc1l	-0.034718438	0.879019376	0.056001552
Arpc2	-0.034695471	0.879019376	0.056001552
Lamtor1	-0.034676582	0.879019376	0.056001552
Pnck	0.050517377	0.879019376	0.056001552
Sfrp5	0.065443504	0.879019376	0.056001552
Tmem120	0.094413497	0.879019376	0.056001552
Gba2	0.107962561	0.879019376	0.056001552
Tex29	-0.051025789	0.879019376	0.056001552
Zfp759	-0.075693017	0.879019376	0.056001552
Timm23	-0.042911302	0.879245523	0.055889834
Chrna5	-0.065053644	0.879245523	0.055889834
Hus1b	0.059447541	0.879300349	0.055862754
Rere	-0.050498222	0.879300349	0.055862754
Slc16a14	0.114401598	0.879312545	0.055856731
Rps7	-0.071168986	0.879400203	0.055813438
Pth2	0.035256806	0.879647338	0.055691407
Agap1	0.054565502	0.879681354	0.055674613
Pde6d	0.255609788	0.880200207	0.055418534
Rbbp7	0.080602468	0.88045456	0.055293053
Slc30a10	0.041573877	0.88045456	0.055293053
Afp	-0.057100785	0.88045456	0.055293053
Rad9a	-0.048915691	0.88045456	0.055293053
Klra9	0.07211427	0.88045456	0.055293053
Cep70	-0.037464278	0.88045456	0.055293053
Fbxo11	0.03441911	0.88045456	0.055293053
Foxl2os	-0.100582748	0.880515177	0.055263154
Champ1	0.176161097	0.880515177	0.055263154

Yars2	-0.088238768	0.880515177	0.055263154
Ache	-0.088074376	0.880515177	0.055263154
Mfap1b	-0.055024948	0.880515177	0.055263154
Ankef1	-0.10136551	0.880515177	0.055263154
Lmo3	0.058321109	0.880515177	0.055263154
Mrpl3	-0.072464545	0.880515177	0.055263154
Eny2	-0.065390348	0.880515177	0.055263154
Tmem255	-0.095399387	0.880515177	0.055263154
Mios	0.050295879	0.880515177	0.055263154
Otub1	0.059045845	0.880515177	0.055263154
Dhx32	0.048751552	0.880515177	0.055263154
Cnot3	0.079967183	0.880515177	0.055263154
Neurl1b	-0.071289734	0.880515177	0.055263154
Oacyl	-0.120807041	0.880515177	0.055263154
Retnla	0.047452624	0.880515177	0.055263154
Terf2ip	0.044354647	0.880515177	0.055263154
Cfhr2	0.066806228	0.880515177	0.055263154
Zbtb5	0.119974272	0.880515177	0.055263154
Fam193b	0.086031444	0.880515177	0.055263154
Cd2bp2	0.039868807	0.880515177	0.055263154
Asf1a	-0.030477911	0.880515177	0.055263154
Spata3	-0.08449991	0.880515177	0.055263154
Ubr2	-0.034242827	0.880515177	0.055263154
Acvr2b	-0.121825224	0.880515177	0.055263154
Rpl39l	0.042167959	0.880515177	0.055263154
Glt8d1	-0.061240749	0.880515177	0.055263154
Pi4k2a	0.034358917	0.880515177	0.055263154
Zfp65	0.043431257	0.8806548	0.055194293
Gskip	-0.076369019	0.880698893	0.05517255
Mbnl3	0.136387691	0.880703926	0.055170067
D7Ertd128	0.047510467	0.880703926	0.055170067
Akr1c12	0.071741828	0.880823395	0.055111159
Zfp110	-0.062467944	0.880897772	0.055074488
Sf3b5	0.034130032	0.88101682	0.0550158
Lipo3	0.069505746	0.881167367	0.054941595
Tubgcp2	0.231945225	0.881167367	0.054941595
Arhgap42	-0.063978026	0.881167367	0.054941595
Aqr	0.033019788	0.881167367	0.054941595
Galnt11	-0.034128898	0.881167367	0.054941595
Fam161b	-0.055109096	0.881323964	0.054864421
Amn1	-0.04757256	0.881323964	0.054864421
Krr1	-0.035352304	0.881323964	0.054864421
Bsx	0.144138979	0.881379427	0.054837091
Sae1	-0.118713866	0.881393254	0.054830278
Fzd2	0.091775409	0.881393254	0.054830278
Tmod2	0.067632296	0.881393254	0.054830278
Pdhb	-0.051803226	0.881393254	0.054830278
Ssbp1	-0.041593573	0.881393254	0.054830278

Hhipl1	-0.119191995	0.881393254	0.054830278
Rnf182	-0.077230041	0.881393254	0.054830278
Zkscan16	0.083245239	0.881393254	0.054830278
Dcdc2a	-0.044525629	0.881393254	0.054830278
Zfp931	-0.058977965	0.881393254	0.054830278
Plxnc1	0.053410003	0.881393254	0.054830278
Tas2r108	-0.050283224	0.881393254	0.054830278
Atp8a1	0.063430896	0.881393254	0.054830278
Svip	0.036822745	0.881393254	0.054830278
Gpr55	0.057886301	0.881393254	0.054830278
Mnd1	0.063422327	0.881393254	0.054830278
Sgcd	-0.096783414	0.881393254	0.054830278
Tecta	0.08053569	0.881393254	0.054830278
Ago3	-0.050927157	0.881393254	0.054830278
Kncn	0.03233311	0.881393254	0.054830278
Foxh1	-0.078856042	0.881393254	0.054830278
Gucy2c	-0.060313373	0.881393254	0.054830278
Calcoco2	-0.036150965	0.881393254	0.054830278
Mpp1	0.084310579	0.881393254	0.054830278
Kcna4	-0.03390299	0.881393254	0.054830278
Malsu1	-0.044802694	0.881393254	0.054830278
Intu	0.062365338	0.881393254	0.054830278
Polr3b	-0.081361763	0.881393254	0.054830278
Sft2d3	-0.038246385	0.881393254	0.054830278
Rassf8	0.059069196	0.881393254	0.054830278
Zfp956	0.045776946	0.881393254	0.054830278
Nap1l1	0.033823309	0.881393254	0.054830278
Prrg1	0.049508709	0.881713955	0.054672285
Sfxn4	0.048026528	0.881713955	0.054672285
Rpa1	0.055475451	0.881713955	0.054672285
PARK2	-0.088575326	0.881749056	0.054654996
Mcph1	0.059968162	0.881749056	0.054654996
Zfp536	-0.10848586	0.881749056	0.054654996
Arhgap29	0.062181394	0.881749056	0.054654996
Zfp91	0.04815705	0.881749056	0.054654996
Pld5	0.133727985	0.881749056	0.054654996
Zfp821	-0.100142423	0.881749056	0.054654996
Zfp235	0.041823072	0.881749056	0.054654996
CLCF1-PO	0.035686501	0.881749056	0.054654996
Gpr4	0.037335802	0.881749056	0.054654996
Trpa1	0.043765738	0.881749056	0.054654996
Mbnl2	-0.093952576	0.881749056	0.054654996
Tcp10b	-0.076074791	0.881749056	0.054654996
Dach2	-0.03334037	0.881749056	0.054654996
Rai1	-0.112955974	0.881749056	0.054654996
Rictor	-0.03365788	0.881749056	0.054654996
Cyb5a	-0.038603713	0.881749056	0.054654996
Zfand1	0.052438114	0.881749056	0.054654996

Gpr18	0.089247285	0.881868954	0.054595946
Zbtb8a	-0.061083998	0.881868954	0.054595946
IL1F5	-0.080411809	0.881868954	0.054595946
Tbc1d12	-0.046848268	0.881868954	0.054595946
Cnot7	-0.049124101	0.881868954	0.054595946
Cul1	0.075935986	0.881868954	0.054595946
Olah	0.041738462	0.881868954	0.054595946
Yif1b	-0.080282553	0.881868954	0.054595946
Orai2	0.074014245	0.881868954	0.054595946
Rxrb	-0.073285174	0.881868954	0.054595946
Car11	-0.073219401	0.881868954	0.054595946
Reep5	0.198924048	0.881868954	0.054595946
Umodl1	0.146077307	0.881953232	0.054554444
Ncdn	0.059254004	0.881953232	0.054554444
Traf3ip1	-0.079547871	0.881953232	0.054554444
Tmem60	-0.113159933	0.881953232	0.054554444
Arhgap1	0.049455946	0.881953232	0.054554444
Ift27	-0.066360284	0.881953232	0.054554444
Socs4	-0.050155282	0.881953232	0.054554444
Pfdn5	0.053430277	0.881953232	0.054554444
Plekhb2	-0.06160217	0.881953232	0.054554444
Wdr66	0.070555004	0.881953232	0.054554444
Efcab3	0.094274943	0.88208833	0.054487924
Tbc1d10c	-0.119901562	0.882093277	0.054485488
Rabgap1l	0.073674701	0.882093277	0.054485488
Trim68	-0.053162803	0.882302435	0.054382522
WHSC1L1	-0.050324197	0.882302435	0.054382522
Tfdp1	0.374643414	0.882760571	0.054157073
Arhgap31	0.076125931	0.882760571	0.054157073
Ddb2	-0.090244399	0.882949359	0.054064205
Ndufs7	0.038939652	0.883071676	0.054004045
Ndufa8	0.116322517	0.883139732	0.053970576
Hs1bp3	-0.140716181	0.88320715	0.053937424
Sdad1	0.033340588	0.88320715	0.053937424
Gbp8	-0.114657313	0.883252955	0.053914901
Gnl3	-0.098883343	0.883252955	0.053914901
Tra2b	-0.039763923	0.883252955	0.053914901
Neto2	-0.039556624	0.883252955	0.053914901
Morn2	-0.132106923	0.883252955	0.053914901
Pten	0.086054742	0.88337563	0.053854586
Nsmce4a	-0.049852071	0.883760212	0.053665555
Gga1	-0.080022158	0.883760212	0.053665555
Nipa2	0.061810196	0.883760212	0.053665555
Nup62	-0.038963748	0.883986842	0.053554199
FAM65A	0.041669746	0.884086629	0.053505178
Rbbp5	-0.09827105	0.884165328	0.053466519
Uchl4	-0.105219655	0.884165328	0.053466519
Abcb1a	-0.046025919	0.884241496	0.053429108

Lemd1	0.039137651	0.884241496	0.053429108
Vps26b	0.16527492	0.884241496	0.053429108
Tescl	0.042535008	0.884574862	0.053265407
Scaper	0.130246592	0.884576054	0.053264821
Mapk7	-0.212012729	0.884576054	0.053264821
Tmem33	-0.053116726	0.884576054	0.053264821
Igsf8	0.083105059	0.884576054	0.053264821
Ankrd6	0.116448702	0.884576054	0.053264821
Glrx2	-0.181821207	0.884733782	0.05318739
Btbd7	0.124329892	0.884733782	0.05318739
SKIV2L2	-0.02675396	0.884845517	0.053132545
N4bp2	-0.193728468	0.884847636	0.053131505
B4galnt1	-0.088466803	0.884847636	0.053131505
Vps8	0.095864123	0.884847636	0.053131505
Rmnd1	-0.164892997	0.884847636	0.053131505
Ppp2r3c	-0.042021889	0.884847636	0.053131505
Tph1	-0.050505073	0.884847636	0.053131505
Metap1	0.058603598	0.884847636	0.053131505
Arhgef7	-0.0511244	0.884847636	0.053131505
Mchr1	-0.107308802	0.884847636	0.053131505
Rsph6a	-0.080454858	0.884847636	0.053131505
Zfp934	-0.076773115	0.884847636	0.053131505
B4galt3	0.050599581	0.884847636	0.053131505
Eif3l	0.042026705	0.884847636	0.053131505
Lhx1os	-0.060148442	0.884847636	0.053131505
H1f0	-0.056065768	0.884847636	0.053131505
Fads1	0.124771508	0.884847636	0.053131505
Kdelr1	-0.043034354	0.884847636	0.053131505
Cltc	0.040296909	0.884847636	0.053131505
Zfp609	0.116593957	0.884847636	0.053131505
Nbeal2	-0.042255596	0.884847636	0.053131505
Ppp6r1	0.141427416	0.884847636	0.053131505
PRKRIR	0.05453214	0.884847636	0.053131505
Prokr2	0.095615197	0.884847636	0.053131505
Atp8b2	0.06675355	0.884847636	0.053131505
C1qtnf4	-0.075909886	0.884847636	0.053131505
Kcnmb3	0.073117143	0.884847636	0.053131505
Rnf13	0.046277992	0.884847636	0.053131505
Sarm1	0.047838562	0.884847636	0.053131505
Slc26a7	0.035228056	0.884847636	0.053131505
Chchd4	-0.060760128	0.884847636	0.053131505
Rpl22l1	0.04654988	0.884847636	0.053131505
Nufip1	-0.032863705	0.884847636	0.053131505
H2AFB1	0.089504835	0.884847636	0.053131505
Dbx1	0.240153792	0.884847636	0.053131505
Mgl2	0.039387578	0.884847636	0.053131505
Rps6ka4	0.033026215	0.884847636	0.053131505
Sap30bp	0.033012195	0.884847636	0.053131505

Tex264	0.032947592	0.884847636	0.053131505
Tug1	0.032841812	0.884847636	0.053131505
Lsm3	0.032829405	0.884847636	0.053131505
Eefsec	-0.07066918	0.884887903	0.053111742
Tex22	0.042323704	0.885073095	0.053020861
Zfp819	-0.10140207	0.885073095	0.053020861
Gtf2a1	0.045117325	0.885073095	0.053020861
Alas1	0.051895052	0.885286	0.052916404
AI197445	-0.204741534	0.88538793	0.052866403
ICK	-0.112075669	0.88538793	0.052866403
Myh2	0.092576797	0.88538793	0.052866403
Msh4	-0.094416984	0.88538793	0.052866403
Dync1li2	-0.14386905	0.88538793	0.052866403
Bag6	-0.06310822	0.88538793	0.052866403
Ndufb9	0.151933301	0.88538793	0.052866403
Adgrf2	-0.111869187	0.88538793	0.052866403
Slc26a9	0.100899829	0.88538793	0.052866403
Nprl2	0.090535911	0.88538793	0.052866403
MESDC1	-0.060789462	0.88538793	0.052866403
Dnajb14	0.06339171	0.88538793	0.052866403
BC04855[illegible]	-0.051744488	0.88538793	0.052866403
Grtp1	-0.080167221	0.88538793	0.052866403
Ces1e	-0.055530476	0.88538793	0.052866403
Nelfb	-0.046308131	0.88538793	0.052866403
Dusp16	-0.0509981	0.88538793	0.052866403
Zfp2	0.100711017	0.88538793	0.052866403
Gemin8	0.105294642	0.88538793	0.052866403
Ccdc88c	0.049946881	0.88538793	0.052866403
Wdr26	-0.062006404	0.88538793	0.052866403
Stat4	-0.071025101	0.88538793	0.052866403
Pax7	0.140680594	0.88538793	0.052866403
Tbc1d9b	-0.055881792	0.88538793	0.052866403
Rnf8	-0.069791992	0.88538793	0.052866403
Ido1	-0.038788897	0.88538793	0.052866403
Ctf2	-0.057266839	0.88538793	0.052866403
Slc17a8	0.040881645	0.88538793	0.052866403
Ap1s2	-0.335382422	0.88538793	0.052866403
Cd3e	0.032644	0.88538793	0.052866403
Orc5	0.032549068	0.88538793	0.052866403
Sos2	-0.180968463	0.885407715	0.052856698
DUXBL2	0.247391254	0.885407715	0.052856698
Dnajc18	-0.200947136	0.885407715	0.052856698
Apbb2	0.122400878	0.885407715	0.052856698
Zfp366	0.065830252	0.885407715	0.052856698
Myl7	0.179773622	0.885407715	0.052856698
Gna11	0.084609165	0.885407715	0.052856698
Cep290	-0.277457218	0.885407715	0.052856698
Myl3	0.049162624	0.885407715	0.052856698

Dvl3	0.125013175	0.885407715	0.052856698
Dmpk	0.08429589	0.885407715	0.052856698
Zfp672	0.089434373	0.885407715	0.052856698
Serpinc1	-0.106473179	0.885407715	0.052856698
Tank	0.063563974	0.885407715	0.052856698
Gtf2h3	0.036390092	0.885407715	0.052856698
Nelfcd	0.060084177	0.885407715	0.052856698
Steap2	0.06419756	0.885407715	0.052856698
Plin5	-0.046977304	0.885407715	0.052856698
Cfap69	0.099312733	0.885407715	0.052856698
FAM49B	0.077106648	0.885407715	0.052856698
Zfp90	0.066992225	0.885407715	0.052856698
Pmis2	0.092496914	0.885407715	0.052856698
Rgs2	-0.085513263	0.885407715	0.052856698
Izumo1	0.056896524	0.885407715	0.052856698
Nrip1	0.053224368	0.885407715	0.052856698
Uqcrc2	0.082939439	0.885407715	0.052856698
Ubr1	-0.120555443	0.885407715	0.052856698
Helq	0.128712368	0.885407715	0.052856698
Acox1	0.061769566	0.885407715	0.052856698
SLMO1	-0.038773977	0.885407715	0.052856698
Urb1	0.045368635	0.885407715	0.052856698
Ergic3	-0.051803249	0.885407715	0.052856698
Pcmtd1	0.047135072	0.885407715	0.052856698
Tbc1d19	-0.088162214	0.885407715	0.052856698
Hyls1	0.09386045	0.885407715	0.052856698
Tmem101	0.047449338	0.885407715	0.052856698
Arl5b	0.047545027	0.885407715	0.052856698
Olfr99	-0.072255234	0.885407715	0.052856698
Rpl36al	-0.081804711	0.885407715	0.052856698
Myg1	0.051024048	0.885407715	0.052856698
Vsig4	0.043038542	0.885407715	0.052856698
Lekr1	0.063181885	0.885407715	0.052856698
Gabpb2	-0.090447554	0.885407715	0.052856698
Chmp3	0.04317739	0.885407715	0.052856698
Lrif1	0.076660646	0.885407715	0.052856698
Slc28a3	-0.102190777	0.885407715	0.052856698
Wfdc18	0.07045693	0.885407715	0.052856698
Ube2r2	0.06264381	0.885407715	0.052856698
Fstl5	-0.13975693	0.885407715	0.052856698
Ythdf1	-0.163425401	0.885407715	0.052856698
Cul2	-0.03231698	0.885407715	0.052856698
Adra2c	0.043788103	0.885407715	0.052856698
Pik3ca	0.070863378	0.885407715	0.052856698
Lrp1	-0.061592355	0.885407715	0.052856698
Cd55b	0.042403059	0.885407715	0.052856698
Vps51	0.051930095	0.885407715	0.052856698
Atg14	-0.132947415	0.885407715	0.052856698

Urgcp	0.044267326	0.885407715	0.052856698
Hnmt	0.062261125	0.885407715	0.052856698
Cdr2l	-0.029409896	0.885407715	0.052856698
Fign	0.059364609	0.885407715	0.052856698
Lyrm1	0.036099466	0.885407715	0.052856698
Cer1	0.032336434	0.885407715	0.052856698
Pithd1	0.032260854	0.885407715	0.052856698
Zfp36l3	0.03221501	0.885407715	0.052856698
Pyurf	-0.095637623	0.885554431	0.05278474
Lmtk3	-0.066851559	0.885554431	0.05278474
Zfp367	0.029797494	0.885554431	0.05278474
Nosip	0.077343979	0.885554431	0.05278474
Pde6c	-0.296273563	0.885651549	0.052737113
Os9	0.09626443	0.885651549	0.052737113
Trim2	-0.088150354	0.885651549	0.052737113
Cideb	-0.078789767	0.885651549	0.052737113
Dnajc16	-0.131538232	0.885651549	0.052737113
Slc41a2	-0.086463358	0.885651549	0.052737113
Map1lc3b	0.09798046	0.885651549	0.052737113
Zfp472	-0.196096529	0.885651549	0.052737113
Pyroxd1	0.166821019	0.885651549	0.052737113
Ptpn9	0.074826785	0.885651549	0.052737113
Cldn10	0.101892254	0.885651549	0.052737113
Fam171b	0.132307819	0.885651549	0.052737113
Ints3	-0.057760892	0.885651549	0.052737113
Irf2bpl	-0.065474366	0.885651549	0.052737113
THAP8	0.253468908	0.885651549	0.052737113
Arpc3	-0.044320393	0.885651549	0.052737113
Ndufb2	-0.111068157	0.885651549	0.052737113
B3glct	0.188472486	0.885651549	0.052737113
Nrl	-0.116419294	0.885651549	0.052737113
Cracr2a	-0.11133455	0.885651549	0.052737113
Serinc2	-0.126892056	0.885651549	0.052737113
Defb47	-0.098785835	0.885651549	0.052737113
Dlg5	-0.06675701	0.885651549	0.052737113
Aarsd1	-0.059670919	0.885651549	0.052737113
Tfpt	-0.073304221	0.885651549	0.052737113
Ralgps1	0.064555418	0.885651549	0.052737113
Dnajc17	-0.071832101	0.885651549	0.052737113
Gja6	0.041797771	0.885651549	0.052737113
Rbp3	-0.060102289	0.885651549	0.052737113
Lsm4	0.057912128	0.885651549	0.052737113
Mtmr1	0.061857044	0.885651549	0.052737113
Wdr89	0.10227428	0.885651549	0.052737113
Abcc1	0.072988426	0.885651549	0.052737113
Abcb7	0.105322076	0.885651549	0.052737113
Ttpa	0.073830763	0.885651549	0.052737113
Olfr1349	0.057274556	0.885651549	0.052737113

Klhl1	-0.044702903	0.885651549	0.052737113
Desi1	0.061615048	0.885651549	0.052737113
Nkd2	-0.1062902	0.885651549	0.052737113
Zcchc9	0.072346087	0.885651549	0.052737113
Shb	-0.089972719	0.885651549	0.052737113
Kcng1	-0.084967582	0.885651549	0.052737113
Pkn2	-0.103396245	0.885651549	0.052737113
Crebrf	0.207415156	0.885651549	0.052737113
Zrsr2	-0.066661046	0.885651549	0.052737113
Platr22	0.201891468	0.885651549	0.052737113
Jmjd6	0.06510543	0.885651549	0.052737113
Cep44	0.043494343	0.885651549	0.052737113
Tulp2	-0.054351163	0.885651549	0.052737113
Xlr3a	0.134178189	0.885651549	0.052737113
Uhrf1bp1l	-0.118880174	0.885651549	0.052737113
Ctrb1	0.063512354	0.885651549	0.052737113
Lhfp	0.056341912	0.885651549	0.052737113
Sgms2	-0.05575042	0.885651549	0.052737113
Tasp1	-0.041909804	0.885651549	0.052737113
Lrpprc	0.049774682	0.885651549	0.052737113
Usp36	-0.086922084	0.885651549	0.052737113
Mtfr1l	-0.05286643	0.885651549	0.052737113
Mrpl46	-0.034859922	0.885651549	0.052737113
Tpsg1	-0.124009936	0.885651549	0.052737113
Ndufb10	0.06788527	0.885651549	0.052737113
Rbpms	-0.037354589	0.885651549	0.052737113
Scd1	0.061760152	0.885651549	0.052737113
Tac2	0.070987717	0.885651549	0.052737113
Ptprs	0.047124738	0.885651549	0.052737113
Gpi1	-0.050135716	0.885651549	0.052737113
FAM160B1	-0.032687936	0.885651549	0.052737113
BC037704	-0.039792309	0.885651549	0.052737113
Defb14	0.056775677	0.885651549	0.052737113
Larp1	0.048319257	0.885651549	0.052737113
Eif4e	0.085754985	0.885651549	0.052737113
Ankrd13c	0.033258442	0.885651549	0.052737113
Rexo4	-0.095739641	0.885651549	0.052737113
Tmem87b	-0.095772554	0.885651549	0.052737113
Cltb	0.091141312	0.885651549	0.052737113
Gtf2i	0.062677119	0.885651549	0.052737113
Ate1	0.042410803	0.885651549	0.052737113
Chrna7	-0.177471901	0.885651549	0.052737113
Polr2m	0.094030976	0.885651549	0.052737113
Cdkl2	-0.124548088	0.885651549	0.052737113
Prps2	0.079523543	0.885651549	0.052737113
Jph4	0.055979814	0.885651549	0.052737113
Lmf1	0.043996692	0.885651549	0.052737113
Ttc26	0.046123819	0.885651549	0.052737113

Tmed1	0.083227294	0.885651549	0.052737113
Mrps16	-0.047109847	0.885651549	0.052737113
Cysltr2	0.037808378	0.885651549	0.052737113
Gne	-0.056249524	0.885651549	0.052737113
Cyp4a12b	0.02711793	0.885651549	0.052737113
Cdc16	0.180575389	0.885651549	0.052737113
Sephs1	0.071244865	0.885651549	0.052737113
Bmpr2	-0.046527065	0.885651549	0.052737113
Fam161a	0.071734943	0.885651549	0.052737113
Hmgcll1	-0.041055041	0.885651549	0.052737113
Pdk2	0.044536183	0.885651549	0.052737113
Col26a1	-0.027770471	0.885651549	0.052737113
Klra17	-0.03068292	0.885651549	0.052737113
Col9a2	0.056662794	0.885651549	0.052737113
Tex9	0.041293732	0.885651549	0.052737113
Rmnd5b	0.050280061	0.885651549	0.052737113
Sytl4	0.051881339	0.885651549	0.052737113
Mir99ahg	-0.034596529	0.885651549	0.052737113
Ethe1	-0.032038843	0.885651549	0.052737113
Vsx2	-0.059971159	0.885651549	0.052737113
Tmx3	-0.031933168	0.885651549	0.052737113
Cd59a	-0.031886306	0.885651549	0.052737113
Noc3l	-0.031344358	0.885651549	0.052737113
Atp4a	0.04855591	0.885651549	0.052737113
Nudt16	-0.087954351	0.885651549	0.052737113
Zfp869	0.164046944	0.885651549	0.052737113
Dtx3	-0.084426355	0.885651549	0.052737113
Mterf4	-0.053036434	0.885651549	0.052737113
Naaladl2	0.038544888	0.885651549	0.052737113
Pgf	-0.046605447	0.885651549	0.052737113
Ctns	-0.061141946	0.885651549	0.052737113
Ilf2	0.129575076	0.885651549	0.052737113
Wt1	-0.026646865	0.885651549	0.052737113
Appl2	0.150884323	0.885651549	0.052737113
Ano3	-0.034549608	0.885651549	0.052737113
Trmt61a	-0.047521829	0.885651549	0.052737113
Uros	0.031747559	0.885651549	0.052737113
Mvb12a	0.031556009	0.885651549	0.052737113
Tmem223	-0.100290235	0.885806818	0.052660982
Zfp64	0.151485503	0.885872631	0.052628716
Zdhhc6	-0.143948368	0.886161377	0.052487182
Mtx2	-0.081241845	0.886161377	0.052487182
Naa60	0.144301214	0.886161377	0.052487182
Ankib1	0.036406779	0.886161377	0.052487182
Pigyl	0.061056304	0.886161377	0.052487182
Fyttd1	0.041193833	0.886161377	0.052487182
Acaca	0.072604855	0.886161377	0.052487182
Psma1	-0.078885242	0.886161377	0.052487182

Shf	-0.100984718	0.886161377	0.052487182
Cops8	-0.124210861	0.886163743	0.052486023
Cops7a	-0.06345893	0.886163743	0.052486023
Kif26b	0.041056364	0.886163743	0.052486023
Cd276	0.145553936	0.886377961	0.052381051
Plekha3	-0.092994984	0.886419503	0.052360697
Il15	0.053927779	0.886419503	0.052360697
Ell	-0.052875328	0.886419503	0.052360697
Rb1	0.033770898	0.886419503	0.052360697
Vps37d	0.086706074	0.886419503	0.052360697
Pigb	-0.080371607	0.886419503	0.052360697
Kmt2e	0.048814712	0.886419503	0.052360697
Tvp23b	-0.04723884	0.886419503	0.052360697
Zic2	0.038010026	0.886419503	0.052360697
Pmp2	-0.043762345	0.886493646	0.052324373
Tkfc	-0.052148564	0.886493646	0.052324373
Btbd19	-0.12811279	0.886584452	0.052279889
Arl6ip1	-0.101436606	0.886584452	0.052279889
Nol3	0.104190676	0.886584452	0.052279889
Anapc16	0.046147558	0.886584452	0.052279889
Sema3f	-0.055004007	0.886584452	0.052279889
Abt1	0.040903921	0.886584452	0.052279889
Dmd	-0.031100637	0.886584452	0.052279889
Evi5l	-0.065977	0.886584452	0.052279889
Krt71	-0.13053171	0.887025745	0.052063775
Mrpl57	0.046702942	0.887025745	0.052063775
Gfer	-0.157376068	0.887112686	0.05202121
Zfp60	-0.085935692	0.887112686	0.05202121
Smim6	-0.078427895	0.887112686	0.05202121
Ddx51	-0.060142009	0.887112686	0.05202121
Gca	0.061504243	0.887112686	0.05202121
Abcc12	0.038541915	0.887112686	0.05202121
Zfp933	0.050659765	0.887112686	0.05202121
Duox2	0.164407045	0.887112686	0.05202121
Pcx	-0.052810857	0.887112686	0.05202121
Slc6a9	-0.110395632	0.887112686	0.05202121
Nomo1	0.067386288	0.887112686	0.05202121
Fam20a	-0.049473358	0.887112686	0.05202121
Ccdc30	-0.173013033	0.887112686	0.05202121
BC042761	0.237927251	0.887112686	0.05202121
Iqcf4	-0.09880676	0.887112686	0.05202121
Mrto4	-0.126357565	0.887112686	0.05202121
Ndufs4	0.11611926	0.887112686	0.05202121
Rpia	-0.047819146	0.887112686	0.05202121
Ppil2	-0.079022485	0.887112686	0.05202121
Kifc3	-0.080402194	0.887112686	0.05202121
Tmprss4	-0.045295748	0.887112686	0.05202121
Clcn7	-0.070634337	0.887112686	0.05202121

Phtf1	-0.047912548	0.887112686	0.05202121
Tns1	0.06725428	0.887112686	0.05202121
Naa25	-0.058159293	0.887112686	0.05202121
Ppig	0.072572132	0.887112686	0.05202121
Zfp597	0.053622289	0.887112686	0.05202121
Ccdc60	0.047611402	0.887112686	0.05202121
Igf2bp3	0.062110735	0.887112686	0.05202121
Dlx2	-0.063090924	0.887112686	0.05202121
Lrrc23	0.046697031	0.887112686	0.05202121
Lsm1	0.041927608	0.887112686	0.05202121
Nampt	-0.082365029	0.887112686	0.05202121
Aldh8a1	0.043694044	0.887112686	0.05202121
Ctf1	-0.05111362	0.887112686	0.05202121
Dcun1d2	0.036421307	0.887112686	0.05202121
Rnf111	0.11896386	0.887112686	0.05202121
Zkscan5	0.029225869	0.887112686	0.05202121
Klf2	0.068967116	0.887112686	0.05202121
D1ERTD62	0.050089255	0.887112686	0.05202121
Hhipl2	-0.039992318	0.887112686	0.05202121
Mex3c	-0.07226734	0.887112686	0.05202121
Acp2	-0.099101755	0.887112686	0.05202121
Greb1	-0.074761658	0.887112686	0.05202121
Crtc2	0.0721529	0.887112686	0.05202121
Zfp874b	-0.030978376	0.887112686	0.05202121
Nutm1	-0.030858285	0.887112686	0.05202121
Eif4a3	-0.030831664	0.887112686	0.05202121
Zfp708	0.046659384	0.887112686	0.05202121
Pgk1	-0.042511665	0.887112686	0.05202121
Timm9	0.036529659	0.887112686	0.05202121
OBFC1	-0.078252415	0.887112686	0.05202121
Pbx1	0.072582938	0.887112686	0.05202121
Ccdc124	-0.032028877	0.887112686	0.05202121
Ice2	0.030771324	0.887112686	0.05202121
Atg4d	-0.074556501	0.88725015	0.051953918
Pus7l	-0.039717719	0.88725015	0.051953918
Usp47	0.08055951	0.887294109	0.051932402
Vps35	-0.086667536	0.887380931	0.051889908
Usp42	-0.056949402	0.887380931	0.051889908
Krtap7-1	0.098359171	0.887380931	0.051889908
Kremen1	-0.06124802	0.887380931	0.051889908
AI314180	0.103051503	0.887380931	0.051889908
Apoa2	-0.034409735	0.887380931	0.051889908
Epb41l4a	-0.082358907	0.887380931	0.051889908
Cldn7	0.084199082	0.887380931	0.051889908
Gtf3c1	0.068520726	0.887380931	0.051889908
Prdx2	0.047657274	0.887380931	0.051889908
Trim17	0.058533929	0.887380931	0.051889908
D17H6S5[illegible]	0.041533293	0.887380931	0.051889908

Mlf1	0.078698189	0.887380931	0.051889908
Dnlz	-0.146745804	0.887380931	0.051889908
Amotl2	0.057910579	0.887380931	0.051889908
Cnot4	-0.075676089	0.887380931	0.051889908
Kank4	-0.068161432	0.887380931	0.051889908
FAM101B	-0.056517129	0.887380931	0.051889908
Nelfa	0.04891249	0.887380931	0.051889908
Nop14	0.052122559	0.887380931	0.051889908
Map4k5	0.042184065	0.887380931	0.051889908
Chm	-0.046445806	0.887380931	0.051889908
Ccdc63	0.103418722	0.887380931	0.051889908
Cpm	0.048584925	0.887380931	0.051889908
Ppm1f	-0.06068455	0.887380931	0.051889908
Rmdn1	-0.044018597	0.887380931	0.051889908
Dsg2	-0.07208687	0.887380931	0.051889908
Lyplal1	0.153152454	0.887380931	0.051889908
Tfe3	0.054594734	0.887380931	0.051889908
Cask	0.078672106	0.887380931	0.051889908
Pick1	0.048415972	0.887380931	0.051889908
Kifc5b	0.073417373	0.887380931	0.051889908
Pqbp1	-0.063559646	0.887380931	0.051889908
Zfp72	-0.047916334	0.887448394	0.051856892
Mid2	-0.078076122	0.887505173	0.051829107
Setmar	0.051871477	0.887505173	0.051829107
Alg3	0.058671426	0.887505173	0.051829107
Bpifc	0.104268378	0.887505173	0.051829107
Zmym4	-0.04978568	0.887505173	0.051829107
Sgta	0.064340197	0.887505173	0.051829107
Wdr41	0.128026986	0.887505173	0.051829107
Dnajb7	0.067641461	0.887505173	0.051829107
Cenpq	-0.130006619	0.887505173	0.051829107
Sat2	0.049695986	0.887505173	0.051829107
Sox30	0.048255678	0.887505173	0.051829107
Ago4	0.03320717	0.887505173	0.051829107
Ttc27	0.040758071	0.887505173	0.051829107
H2-M2	-0.074439007	0.887505173	0.051829107
Cmah	0.055652729	0.887566817	0.051798943
Traf3	-0.05402449	0.887566817	0.051798943
Me1	-0.106390148	0.887568476	0.051798131
Kcna7	-0.141040326	0.887805852	0.051681996
Slc52a2	-0.039603604	0.887891798	0.051639956
Riok3	0.050988402	0.887891798	0.051639956
Ptpn5	-0.227167301	0.887933098	0.051619755
Mobp	-0.146358144	0.887933098	0.051619755
Brap	0.235736462	0.887933098	0.051619755
Arhgef12	-0.06293347	0.887933098	0.051619755
Tmc3	0.079843799	0.887933098	0.051619755
Abhd16a	-0.12524188	0.887933098	0.051619755

Sh2b1	-0.075160147	0.887933098	0.051619755
Trrap	-0.08300343	0.887933098	0.051619755
Bvht	0.075368776	0.887933098	0.051619755
Mpzl2	0.292969762	0.887933098	0.051619755
PTPRG	-0.065927628	0.887933098	0.051619755
DGCR14	-0.051887381	0.887933098	0.051619755
Rufy2	-0.086418076	0.887933098	0.051619755
Fubp1	0.046051152	0.887933098	0.051619755
Klhl23	0.038956214	0.887933098	0.051619755
Tcp1	0.046884165	0.887933098	0.051619755
Ccdc82	-0.103317078	0.887933098	0.051619755
Bdp1	-0.058074101	0.887933098	0.051619755
Krtap17-1	-0.055463716	0.887933098	0.051619755
Bend4	-0.031095341	0.887933098	0.051619755
Anp32e	0.0624897	0.887933098	0.051619755
Gphb5	-0.037839975	0.887933098	0.051619755
CYP2C44	0.056211317	0.887933098	0.051619755
Micu1	-0.048223895	0.887933098	0.051619755
Rnf115	0.050020012	0.887933098	0.051619755
Xrn2	0.079185426	0.887933098	0.051619755
Psmd9	-0.218098492	0.887933098	0.051619755
Glis2	-0.055729682	0.887933098	0.051619755
Btg3	0.046816123	0.888154636	0.051511413
Thap4	0.052600525	0.888154636	0.051511413
Rpp30	-0.054738669	0.888154636	0.051511413
BC003965	0.030154598	0.888198388	0.051490019
Sfxn1	-0.088392811	0.888679644	0.051254767
Zkscan14	0.153485269	0.88870086	0.051244399
Nog	-0.091681388	0.88870086	0.051244399
Klri2	-0.186769581	0.88870086	0.051244399
Cfap20	-0.098132438	0.88870086	0.051244399
Ptch2	0.070004246	0.88870086	0.051244399
Ppp6r2	0.048747204	0.88870086	0.051244399
Olfr1028	0.099804138	0.88870086	0.051244399
Scn3b	0.092145241	0.88870086	0.051244399
Dhps	0.165897696	0.88870086	0.051244399
Fibcd1	0.177296144	0.88870086	0.051244399
Cep95	-0.08391632	0.88870086	0.051244399
Mro	0.043209083	0.88870086	0.051244399
Gapvd1	0.032060047	0.88870086	0.051244399
HDGFRP2	0.052963485	0.88870086	0.051244399
Rassf6	0.040564777	0.88870086	0.051244399
Harbi1	0.081212859	0.88870086	0.051244399
Rpp21	-0.061820551	0.88870086	0.051244399
Daam2	-0.079225988	0.88870086	0.051244399
Ebf1	-0.045693394	0.88870086	0.051244399
Fbxl12	-0.051470333	0.88870086	0.051244399
Sfswap	0.046149213	0.88870086	0.051244399

Cwf19l2	-0.057042618	0.88870086	0.051244399
Abhd13	0.041383899	0.88870086	0.051244399
Heatr4	-0.052450833	0.88870086	0.051244399
Tmem143	-0.030033867	0.88870086	0.051244399
Col13a1	-0.029949704	0.88870086	0.051244399
Cox7b2	0.04183101	0.88870086	0.051244399
Arid3c	0.032204936	0.88870086	0.051244399
Creb3l3	-0.027117021	0.88870086	0.051244399
Supt3	0.059507121	0.88870086	0.051244399
Sbds	0.054677146	0.88870086	0.051244399
Heg1	0.044188508	0.88870086	0.051244399
Prpf19	0.030043825	0.88870086	0.051244399
Pdzrn4	0.156072792	0.888723159	0.051233503
Stambp	-0.053648121	0.888791862	0.051199931
Dnajc30	-0.117607772	0.888834629	0.051179034
Hsd17b1	-0.043387774	0.888851574	0.051170754
Tmprss3	0.066586434	0.888985951	0.051105103
Epb41	0.056827917	0.888985951	0.051105103
Aldob	0.088949839	0.888985951	0.051105103
Col6a4	-0.151396616	0.889035873	0.051080714
Xpnpep1	0.048106103	0.889035873	0.051080714
Ascc1	0.065845201	0.889035873	0.051080714
Notum	-0.052920065	0.889035873	0.051080714
Gsx2	-0.065388458	0.889035873	0.051080714
Pot1a	-0.037699468	0.889035873	0.051080714
Tppp3	-0.053400551	0.889035873	0.051080714
Tex38	0.070218991	0.889035873	0.051080714
Slc25a30	0.076906562	0.889035873	0.051080714
Cass4	-0.227217741	0.88913095	0.051034272
Hps4	0.171960717	0.88913095	0.051034272
Aste1	0.030860102	0.88913095	0.051034272
Gp2	0.056576025	0.88913095	0.051034272
Bms1	-0.042515956	0.88913095	0.051034272
Dcaf13	0.230596978	0.88913095	0.051034272
Stard3nl	0.049125096	0.88913095	0.051034272
Neto1	-0.079094647	0.88913095	0.051034272
Fam3c	0.051432216	0.88913095	0.051034272
Wdr35	-0.072623595	0.88913095	0.051034272
Hhla1	0.044673543	0.88913095	0.051034272
Rpl30	-0.065440922	0.88913095	0.051034272
Clcn3	0.050943548	0.88913095	0.051034272
P2rx6	-0.040336644	0.88913095	0.051034272
Ptprr	-0.041304767	0.88913095	0.051034272
Porcn	0.113998935	0.88913095	0.051034272
Gss	0.079259028	0.88913095	0.051034272
Dhdh	0.051130551	0.88913095	0.051034272
Palb2	0.029805956	0.88913095	0.051034272
Ambp	0.029730598	0.88913095	0.051034272

TMEM55A	0.055113193	0.889274919	0.050963956
Fam110b	-0.054179392	0.889506304	0.05085097
Padi1	0.057279528	0.889506304	0.05085097
Xcr1	0.048690886	0.889572701	0.050818553
Capzb	0.07971021	0.88964996	0.050780836
Bag1	0.033564092	0.88964996	0.050780836
Prr18	-0.036461231	0.88967779	0.050767251
Trappc12	-0.068856365	0.889944879	0.050636892
Ap1g2	0.136121776	0.889944879	0.050636892
Snx17	0.045903493	0.889944879	0.050636892
Rps6ka5	-0.034682199	0.889944879	0.050636892
Smarce1	0.035062801	0.889944879	0.050636892
Mcee	0.035811946	0.889944879	0.050636892
Vmn1r90	0.044223889	0.889969625	0.050624816
Spink12	0.044805615	0.889969625	0.050624816
Nkx1-1	0.141941889	0.88998579	0.050616927
Mafg	-0.222300554	0.88998579	0.050616927
Phc1	-0.145791964	0.88998579	0.050616927
Cystm1	-0.077381796	0.88998579	0.050616927
Ide	-0.087008967	0.88998579	0.050616927
Lemd3	-0.066351383	0.88998579	0.050616927
Zfp292	0.04824274	0.88998579	0.050616927
Prelp	0.049821999	0.88998579	0.050616927
C87436	-0.093408568	0.88998579	0.050616927
Zfp94	0.049996853	0.88998579	0.050616927
Cetn2	0.140922191	0.88998579	0.050616927
Rxfp1	-0.040913665	0.88998579	0.050616927
Dock4	-0.082037662	0.88998579	0.050616927
Fyco1	0.079200893	0.88998579	0.050616927
PIRA6	0.092943777	0.88998579	0.050616927
Pdlim3	0.07557066	0.88998579	0.050616927
Chmp2b	-0.069865734	0.88998579	0.050616927
Cnnm2	0.039520669	0.88998579	0.050616927
Nckap5	0.06013104	0.88998579	0.050616927
AA414992	-0.057824074	0.88998579	0.050616927
Sh3bp5	-0.042717316	0.88998579	0.050616927
Fam210a	-0.066496028	0.88998579	0.050616927
Psmc1	0.061527452	0.88998579	0.050616927
Atpif1	-0.033599308	0.88998579	0.050616927
Ccdc39	-0.066157952	0.88998579	0.050616927
Itpkc	-0.124890612	0.88998579	0.050616927
Sox6os	0.041045157	0.88998579	0.050616927
Apob	-0.052595737	0.88998579	0.050616927
Usp30	-0.047820558	0.88998579	0.050616927
Sephs2	0.112407082	0.88998579	0.050616927
Col28a1	0.062224214	0.88998579	0.050616927
Tars	-0.054451544	0.88998579	0.050616927
Ttc4	-0.087871625	0.88998579	0.050616927

Casc3	0.077037354	0.88998579	0.050616927
Rbm34	0.059103005	0.88998579	0.050616927
Epn2	0.043334855	0.88998579	0.050616927
Atpaf2	0.033390027	0.88998579	0.050616927
Med9os	-0.049881161	0.88998579	0.050616927
Pikfyve	0.067647546	0.88998579	0.050616927
Numbl	0.113905835	0.88998579	0.050616927
Ccser2	-0.049166758	0.88998579	0.050616927
Tdrd6	0.088977027	0.88998579	0.050616927
B3galt6	-0.089619047	0.88998579	0.050616927
Ppp1r8	0.042321352	0.88998579	0.050616927
Asxl1	0.055931046	0.88998579	0.050616927
Ascl4	0.044085867	0.88998579	0.050616927
Abcg5	0.071052738	0.88998579	0.050616927
Tmem216	-0.06757593	0.88998579	0.050616927
Eif2a	-0.06005964	0.88998579	0.050616927
Aass	-0.048458586	0.88998579	0.050616927
Psmf1	0.054284961	0.88998579	0.050616927
Supt6	-0.047805315	0.88998579	0.050616927
Vtn	-0.124978904	0.88998579	0.050616927
Timm8a1	0.099870003	0.88998579	0.050616927
Olfr689	-0.049478506	0.88998579	0.050616927
Fam207a	0.056295671	0.88998579	0.050616927
Fgf20	-0.03951917	0.88998579	0.050616927
Nagk	0.110895918	0.88998579	0.050616927
Rasa2	-0.036964639	0.88998579	0.050616927
Wdr72	0.105991411	0.88998579	0.050616927
Ppargc1b	-0.029478494	0.88998579	0.050616927
Lctl	-0.029406292	0.88998579	0.050616927
G6pc3	0.09247891	0.88998579	0.050616927
Rnf185	-0.053236121	0.88998579	0.050616927
Aven	0.086927789	0.88998579	0.050616927
Ropn1	-0.059853085	0.88998579	0.050616927
Pah	0.145691775	0.88998579	0.050616927
Pde2a	0.049152373	0.88998579	0.050616927
Fastk	-0.030263828	0.88998579	0.050616927
Selenbp2	-0.038698576	0.88998579	0.050616927
Npff	0.045900214	0.88998579	0.050616927
Bad	0.040532426	0.88998579	0.050616927
Il20ra	-0.055126486	0.890073693	0.050574035
Mbl2	0.054481066	0.890073693	0.050574035
Rnf139	0.060977143	0.890073693	0.050574035
SDCCAG3	0.059418161	0.890073693	0.050574035
Rtp2	-0.035500686	0.890073693	0.050574035
Izumo4	0.029178018	0.890073693	0.050574035
Aggf1	0.097990711	0.890122864	0.050550044
Dgcr8	-0.148017465	0.890368287	0.050430317
Bmi1	-0.067323139	0.890368287	0.050430317

Tubg1	-0.057867056	0.890368287	0.050430317
GUCY1A3	-0.050637678	0.890368287	0.050430317
Slurp1	0.050771306	0.890368287	0.050430317
Bex6	0.101130891	0.890368287	0.050430317
Cdk20	0.146122257	0.890368287	0.050430317
Myh4	-0.055676942	0.890368287	0.050430317
Best1	0.088949097	0.890368287	0.050430317
Tpm3	0.051321574	0.890368287	0.050430317
Acsl3	0.067437923	0.890368287	0.050430317
Sclt1	-0.072805844	0.890368287	0.050430317
Rnf148	0.077744274	0.890368287	0.050430317
Syn2	0.092183041	0.890368287	0.050430317
AI507597	0.070639339	0.890368287	0.050430317
Vdr	-0.040391478	0.890368287	0.050430317
Nuggc	-0.074039945	0.890368287	0.050430317
Arv1	0.120319642	0.890368287	0.050430317
Ap4b1	-0.051301379	0.890368287	0.050430317
Galr1	-0.07793165	0.890368287	0.050430317
Mal	-0.1208663	0.890368287	0.050430317
Tmprss7	0.060022772	0.890368287	0.050430317
Zmym3	-0.050703738	0.890368287	0.050430317
Smim7	-0.140581898	0.890368287	0.050430317
Gsto1	0.060075308	0.890368287	0.050430317
Stx6	0.158672476	0.890368287	0.050430317
Trmt10b	0.057884907	0.890368287	0.050430317
Atp5g3	0.373309089	0.890368287	0.050430317
Stard13	0.132432794	0.890368287	0.050430317
Txlng	0.092642675	0.890368287	0.050430317
Clic6	-0.060763319	0.890368287	0.050430317
Scn3a	-0.050828481	0.890368287	0.050430317
Rergl	0.061373727	0.890368287	0.050430317
Scaf8	-0.059138571	0.890368287	0.050430317
Lgi3	-0.046122172	0.890368287	0.050430317
Emc3	0.177707978	0.890368287	0.050430317
Npbwr1	-0.052890859	0.890368287	0.050430317
Casd1	-0.048007633	0.890368287	0.050430317
Carm1	-0.044146739	0.890368287	0.050430317
Col15a1	0.054161394	0.890368287	0.050430317
Ano8	0.072620793	0.890368287	0.050430317
Cdkl3	0.075960538	0.890368287	0.050430317
Mink1	0.036278379	0.890368287	0.050430317
CCDC36	-0.056655119	0.890368287	0.050430317
Trmu	-0.092414376	0.890368287	0.050430317
Ern1	-0.03899652	0.890368287	0.050430317
Tmco1	-0.095352908	0.890368287	0.050430317
Gpr62	0.058254936	0.890368287	0.050430317
Vps18	-0.088402757	0.890368287	0.050430317
M1ap	0.04630625	0.890368287	0.050430317

Rabif	-0.027770634	0.890368287	0.050430317
Zfp13	0.047373443	0.890368287	0.050430317
Men1	0.056017394	0.890368287	0.050430317
Gabrg1	-0.031984371	0.890368287	0.050430317
Rbm46	-0.066510711	0.890368287	0.050430317
Rps8	-0.040806847	0.890368287	0.050430317
Nfx1	-0.039218562	0.890368287	0.050430317
Serpina3c	0.078746656	0.890368287	0.050430317
Mmrn2	0.040942212	0.890368287	0.050430317
Shroom2	0.037247329	0.890368287	0.050430317
Tcea2	0.042041437	0.890368287	0.050430317
Sipa1l3	0.035062035	0.890368287	0.050430317
Farp2	0.06925226	0.890368287	0.050430317
Itga8	0.061766428	0.890368287	0.050430317
Acaa1a	-0.321291834	0.890368287	0.050430317
Ppip5k2	-0.111047443	0.890503985	0.050364133
Ampd2	-0.065949847	0.890599239	0.05031768
Sec24b	0.046853421	0.890599239	0.05031768
Slc25a4	-0.052559881	0.890599239	0.05031768
Tbx3os1	0.051054242	0.890599239	0.05031768
Gramd4	0.043052137	0.890645071	0.050295331
Olfr31	0.048112445	0.890721871	0.050257884
Noct	-0.051022187	0.890721871	0.050257884
Wdr5	-0.025787666	0.890721871	0.050257884
Tmx1	0.088838823	0.890943908	0.050149638
Srek1ip1	-0.100289483	0.890945419	0.050148901
Ndufa5	0.076272586	0.890945419	0.050148901
Sec23a	0.097393646	0.890945419	0.050148901
Rsbn1l	0.037491807	0.890945419	0.050148901
Plpp1	-0.096847028	0.890945419	0.050148901
Orm1	0.055199758	0.890945419	0.050148901
TMEM136	0.105915773	0.890945419	0.050148901
Cldn17	-0.052235537	0.890945419	0.050148901
Pms1	-0.039974914	0.890945419	0.050148901
Ppil3	-0.061593925	0.891085799	0.050080478
Arc	0.048918387	0.891085799	0.050080478
Chac2	-0.11500231	0.891143658	0.050052279
Dmbx1	0.064997165	0.891143658	0.050052279
Ints12	0.039584	0.891143658	0.050052279
Ahcyl1	0.065708165	0.891143658	0.050052279
Fgfbp3	0.073805861	0.891143658	0.050052279
Golga4	0.054066772	0.891143658	0.050052279
Gpaa1	-0.029939876	0.891143658	0.050052279
Zkscan2	-0.025796186	0.891143658	0.050052279
Nfkbil1	-0.02859541	0.891144591	0.050051825
Adam2	0.030667555	0.891185346	0.050031963
Fancg	-0.068999004	0.891185346	0.050031963
Rps6	-0.047793084	0.891186971	0.050031172

Map4	0.057669431	0.891186971	0.050031172
Ctbp1	0.093747817	0.891209101	0.050020387
Atp10b	-0.146156188	0.89125151	0.049999721
Ncoa6	0.050522171	0.89125151	0.049999721
AA414768	0.105411963	0.891444393	0.049905742
Rab14	-0.088917069	0.891444393	0.049905742
Frmd8os	-0.10559692	0.891489713	0.049883664
Spaca1	-0.070391325	0.891489713	0.049883664
Nub1	-0.036109525	0.891489713	0.049883664
Zmiz1	-0.089682489	0.891489713	0.049883664
Tbca	-0.041970362	0.891489713	0.049883664
Gtpbp8	-0.065551511	0.891489713	0.049883664
Morf4l1	0.081276619	0.891489713	0.049883664
Alb	-0.039856398	0.891489713	0.049883664
Pam16	0.046494678	0.891489713	0.049883664
Tsc2	-0.09239293	0.891489713	0.049883664
Agpat1	-0.051545787	0.891493401	0.049881867
Gigyf2	-0.07261911	0.891516362	0.049870682
Nr2f2	-0.056259918	0.891516362	0.049870682
Mier1	-0.145325374	0.891516362	0.049870682
Tdrkh	0.116133167	0.891516362	0.049870682
Tmem68	0.065842206	0.891516362	0.049870682
Pdzd11	-0.080263413	0.891516362	0.049870682
Akr1b7	0.062855616	0.891516362	0.049870682
Tfb1m	0.10100892	0.891516362	0.049870682
Ap5m1	-0.048769977	0.891516362	0.049870682
Slc47a1	-0.075977363	0.891516362	0.049870682
Pde12	-0.096336038	0.891516362	0.049870682
Supt20	0.034313898	0.891516362	0.049870682
Ogg1	-0.039991137	0.891516362	0.049870682
Nr5a2	-0.037465744	0.891516362	0.049870682
Arhgap33	0.068688979	0.891516362	0.049870682
Spata5	0.163603214	0.891516362	0.049870682
Psmd8	0.047505015	0.891516362	0.049870682
Cuzd1	0.040352411	0.891516362	0.049870682
Maged1	-0.060066894	0.891516362	0.049870682
Ube2v1	0.041133872	0.891516362	0.049870682
Arhgap27(	-0.075061187	0.891516362	0.049870682
Arfrp1	-0.033184559	0.891516362	0.049870682
Vdac3	0.028394241	0.891516362	0.049870682
Dntt	-0.065758087	0.891564586	0.049847191
Stk36	0.18284002	0.891564586	0.049847191
Slc38a8	0.075019452	0.891564586	0.049847191
Paupar	-0.085316251	0.891564586	0.049847191
Abtb1	-0.214564677	0.891564586	0.049847191
Irf4	0.076764662	0.891564586	0.049847191
Gml	0.05407926	0.891564586	0.049847191
Acad11	0.037902744	0.891564586	0.049847191

Scamp4	-0.134732088	0.891564586	0.049847191
Olfr878	-0.089310279	0.891564586	0.049847191
Lhfpl1	0.059012501	0.891564586	0.049847191
Lrrd1	0.062958699	0.891564586	0.049847191
Edem3	0.196496542	0.891564586	0.049847191
APOPT1	-0.037714656	0.891564586	0.049847191
Pcsk5	0.049876672	0.891564586	0.049847191
Tmem64	0.05234997	0.891564586	0.049847191
Lzts2	-0.070349537	0.891564586	0.049847191
Pnpo	-0.054171714	0.891564586	0.049847191
Armc9	-0.051030952	0.891564586	0.049847191
Abca4	-0.037472983	0.891564586	0.049847191
Slc45a4	0.05882938	0.891564586	0.049847191
Pla2r1	0.065910327	0.891564586	0.049847191
Zfp414	0.051571744	0.891564586	0.049847191
Ctnnb1	-0.058765108	0.891564586	0.049847191
Prss54	-0.180662902	0.891714866	0.049773993
Atp12a	-0.088736545	0.891714866	0.049773993
Nhs	-0.144799225	0.891714866	0.049773993
Zfyve27	-0.105621704	0.891714866	0.049773993
Zfp668	-0.118387448	0.891714866	0.049773993
Sec31b	-0.096515795	0.891714866	0.049773993
Unc13c	-0.089950636	0.891714866	0.049773993
Slc2a12	0.108246871	0.891714866	0.049773993
Olfr55	0.082268086	0.891714866	0.049773993
Azi2	-0.183330957	0.891714866	0.049773993
Lrrfip2	-0.188685314	0.891714866	0.049773993
Vat1l	0.139121062	0.891714866	0.049773993
Bbof1	-0.110463939	0.891714866	0.049773993
Fbxo22	-0.108911428	0.891714866	0.049773993
Eif3g	0.064971363	0.891714866	0.049773993
Hey2	-0.061176161	0.891714866	0.049773993
Zfp641	0.094828713	0.891714866	0.049773993
Tgoln1	-0.110131603	0.891714866	0.049773993
Fastkd1	-0.042058235	0.891714866	0.049773993
Ggps1	0.073549887	0.891714866	0.049773993
Mbd4	0.083525662	0.891714866	0.049773993
Cyp2u1	0.036911123	0.891714866	0.049773993
Ifngr2	-0.096666328	0.891714866	0.049773993
Stam	-0.056763028	0.891714866	0.049773993
Ece1	0.145607224	0.891714866	0.049773993
Wac	-0.054807875	0.891714866	0.049773993
Zfp142	0.073241666	0.891714866	0.049773993
Pla2g10	0.231491273	0.891714866	0.049773993
Ogt	-0.056897148	0.891714866	0.049773993
Cd96	0.038156659	0.891714866	0.049773993
Egr2	-0.091723358	0.891714866	0.049773993
PAPD7	-0.066998018	0.891714866	0.049773993

Zdhhc4	-0.050409828	0.891714866	0.049773993
Rem1	-0.077442096	0.891714866	0.049773993
Las1l	0.040751342	0.891714866	0.049773993
Chrnb1	0.054112593	0.891714866	0.049773993
Pwp1	0.047169594	0.891714866	0.049773993
Cacybp	0.046942896	0.891714866	0.049773993
Olfr1508	0.05309495	0.891714866	0.049773993
Aasdh	0.076317202	0.891714866	0.049773993
P2rx5	-0.082799201	0.891714866	0.049773993
Fgd6	0.089878722	0.891714866	0.049773993
Mpzl3	0.076103925	0.891714866	0.049773993
GLTSCR2	0.07805447	0.891714866	0.049773993
Stk3	0.04374131	0.891714866	0.049773993
Zhx2	-0.057082641	0.891714866	0.049773993
Rxra	0.041074484	0.891714866	0.049773993
Upk1b	-0.067799693	0.891714866	0.049773993
Olfr877	-0.034336377	0.891714866	0.049773993
Olfr61	0.082178778	0.891714866	0.049773993
Psmb5	0.078669243	0.891714866	0.049773993
Serpina12	-0.063989938	0.891714866	0.049773993
Rnase10	-0.034518299	0.891714866	0.049773993
Rilp	0.054207182	0.891714866	0.049773993
Kdm4d	-0.078763194	0.891714866	0.049773993
Grin3a	0.049869509	0.891714866	0.049773993
Slc6a5	-0.03573959	0.891714866	0.049773993
Zfp772	-0.052543181	0.891714866	0.049773993
Cep57	0.04580865	0.891714866	0.049773993
Mgat5b	-0.045939164	0.891714866	0.049773993
Tekt3	0.10763449	0.891714866	0.049773993
Depdc7	0.034997624	0.891714866	0.049773993
Olfr736	0.046184996	0.891714866	0.049773993
Naaladl1	0.033248739	0.891714866	0.049773993
Defb12	0.039947108	0.891714866	0.049773993
Rpl8	0.121984038	0.891714866	0.049773993
Dhrs11	0.05585671	0.891714866	0.049773993
Calr3	-0.051112094	0.891714866	0.049773993
Slco1a4	0.05075506	0.891714866	0.049773993
Klhl41	0.042191379	0.891714866	0.049773993
Lyve1	0.045554571	0.891714866	0.049773993
Ccr9	0.044671509	0.891714866	0.049773993
Zfp507	-0.073036855	0.891714866	0.049773993
Ccdc157	0.059579387	0.891714866	0.049773993
Atp6v0c	0.042965111	0.891714866	0.049773993
Mllt1	0.032420551	0.891714866	0.049773993
Ube2z	-0.048226991	0.891714866	0.049773993
Clip3	0.069368489	0.891714866	0.049773993
Klk7	-0.044770507	0.891714866	0.049773993
Pglyrp2	-0.049852061	0.891714866	0.049773993

Errfi1	0.042524418	0.891714866	0.049773993
Sik2	0.043571701	0.891714866	0.049773993
Plppr3	0.106784478	0.891714866	0.049773993
Zfc3h1	0.064255399	0.891714866	0.049773993
Polr2g	0.058662104	0.891714866	0.049773993
Med28	0.103641003	0.891714866	0.049773993
Arnt	0.038596352	0.891714866	0.049773993
Egln2	-0.095136491	0.891714866	0.049773993
Chkb	-0.047383723	0.891714866	0.049773993
Platr10	0.048769554	0.891714866	0.049773993
Ciz1	-0.042500057	0.891714866	0.049773993
Kti12	0.046884624	0.891714866	0.049773993
Cpne4	0.056597593	0.891714866	0.049773993
Mylk2	-0.101551312	0.891714866	0.049773993
Gemin4	0.047682803	0.891714866	0.049773993
Lca5l	0.100206151	0.891714866	0.049773993
Mpc2	-0.120489625	0.891714866	0.049773993
Zfp945	-0.040687535	0.891714866	0.049773993
Tmie	-0.041621023	0.891714866	0.049773993
MUM1	-0.074276499	0.891714866	0.049773993
Prss1	-0.033427457	0.891714866	0.049773993
Tmeff1	0.056471025	0.891714866	0.049773993
Frem3	-0.035820234	0.891714866	0.049773993
Zfp560	-0.048396682	0.891714866	0.049773993
Pdha1	0.058236204	0.891714866	0.049773993
Mprip	-0.047746707	0.891714866	0.049773993
Olfr687	0.040415083	0.891714866	0.049773993
Rad50	-0.044601499	0.891714866	0.049773993
Cluh	0.037018412	0.891714866	0.049773993
Smim12	-0.030737664	0.891714866	0.049773993
Wfdc9	0.02870368	0.891714866	0.049773993
Tctn3	0.040167427	0.891714866	0.049773993
TWISTNB	0.027846341	0.891714866	0.049773993
Mcm8	-0.052549858	0.892135796	0.049569035
HRASLS5	-0.051929941	0.892135796	0.049569035
Rraga	-0.056453121	0.892161304	0.049556617
Mcm3ap	-0.144875818	0.892252098	0.049512422
Sox13	-0.137610211	0.892252098	0.049512422
Guf1	0.064229143	0.892252098	0.049512422
Krt7	-0.089498373	0.892252098	0.049512422
Ikzf4	-0.122522216	0.892252098	0.049512422
Osr2	0.05812431	0.892252098	0.049512422
Serpina1c	0.048550725	0.892252098	0.049512422
Gas8	0.041235675	0.892252098	0.049512422
Krt75	0.064473951	0.892252098	0.049512422
Clcnka	-0.044127589	0.892252098	0.049512422
Pop4	0.080447695	0.892252098	0.049512422
Iqub	0.056551424	0.892252098	0.049512422

Lman2	0.073005489	0.892252098	0.049512422
Lce1f	0.147580936	0.892252098	0.049512422
Bod1	-0.031667363	0.892252098	0.049512422
mt-Rnr1	0.05370674	0.892252098	0.049512422
Leng8	0.041460874	0.892252098	0.049512422
Idnk	0.053870832	0.892252098	0.049512422
Jmy	0.039030631	0.892252098	0.049512422
Trp63	0.047418783	0.892252098	0.049512422
Golga3	0.027529618	0.892252098	0.049512422
Igfbpl1	-0.132986241	0.892284131	0.049496831
Cyp8b1	-0.14083105	0.892284131	0.049496831
Ino80b	-0.143040337	0.892284131	0.049496831
Il22	0.06767521	0.892284131	0.049496831
Redrum	-0.077598394	0.892284131	0.049496831
Zfp518b	0.046909956	0.892284131	0.049496831
Tdrd9	0.046143452	0.892284131	0.049496831
Fbl	0.07302783	0.892284131	0.049496831
Nipal2	-0.059993249	0.892284131	0.049496831
Gpr152	-0.147650768	0.892284131	0.049496831
Xylb	0.122962174	0.89252382	0.049380185
Cbx3	-0.054816318	0.89252382	0.049380185
Rtca	-0.048371902	0.892578531	0.049353563
Spin2c	0.047331686	0.893350465	0.048978132
Cnot11	0.057383517	0.893350465	0.048978132
Slc22a7	-0.056461967	0.893350465	0.048978132
Agtr1b	0.027373946	0.893350465	0.048978132
Timm21	-0.114921525	0.89349168	0.048909487
Krt18	-0.104199584	0.89349168	0.048909487
Syt11	0.044331354	0.89349168	0.048909487
Rcbtb1	-0.04269486	0.89349168	0.048909487
Nol6	0.035538369	0.89349168	0.048909487
Defb6	-0.048174016	0.89349168	0.048909487
Prop1	0.054965605	0.89349168	0.048909487
Zfp871	-0.027326658	0.89349168	0.048909487
Slc35b1	0.049744819	0.89351519	0.04889806
Kcnab1	-0.054165153	0.893521843	0.048894826
Tmem171	0.051310093	0.893521843	0.048894826
Bbs12	-0.079211481	0.893678653	0.048818616
Zfpl1	-0.133988082	0.893678653	0.048818616
Sec16b	-0.046065566	0.893696789	0.048809802
Zfp608	0.03463385	0.893696789	0.048809802
AY074887	-0.046274918	0.893696789	0.048809802
Blm	0.037928226	0.893696789	0.048809802
Fxyd6	-0.02726966	0.893696789	0.048809802
Olfr1347	0.049444201	0.893696789	0.048809802
DEB1	0.100665979	0.893696789	0.048809802
Cdnf	0.047470226	0.89389052	0.048715668
Hnrnph1	0.072422227	0.894085115	0.048621136

Zfp872	-0.082068224	0.894085115	0.048621136
Phf6	-0.068016333	0.894085115	0.048621136
Rttn	0.062638816	0.894085115	0.048621136
Cebpzos	0.092371232	0.894085115	0.048621136
Lrrc45	0.054640357	0.894085115	0.048621136
Dtna	-0.049540787	0.894085115	0.048621136
Phlda2	-0.073760927	0.894085115	0.048621136
Mfsd10	-0.058953234	0.894085115	0.048621136
Vmn1r206	-0.078138807	0.894085115	0.048621136
Lrrc75a	-0.066207666	0.894085115	0.048621136
H2AFY2	-0.073952353	0.894085115	0.048621136
Abcc6	-0.034201717	0.894085115	0.048621136
Armc2	-0.049232925	0.894085115	0.048621136
Cyb5d1	0.182291941	0.894273626	0.048529578
Tbc1d2	-0.052575372	0.894273626	0.048529578
Calcb	0.071891158	0.894273626	0.048529578
Sox1	-0.063036423	0.894273626	0.048529578
FAM21	0.098704293	0.894273626	0.048529578
Teddm1b	-0.053337245	0.894273626	0.048529578
Vdac1	0.052058353	0.894273626	0.048529578
Elp6	-0.048756238	0.894273626	0.048529578
Ppp1cb	0.036871495	0.894273626	0.048529578
Tonsl	0.088563893	0.894273626	0.048529578
Il17re	-0.038807133	0.894273626	0.048529578
Nphp3	-0.069274575	0.894273626	0.048529578
AU022252	-0.076975785	0.894273626	0.048529578
Csgalnact	-0.058105377	0.894273626	0.048529578
Emilin3	0.027103778	0.894273626	0.048529578
Gabrr3	-0.047750472	0.894484731	0.048427068
Ubap1	-0.028910221	0.894484731	0.048427068
Cse1l	0.045542829	0.894536823	0.048401777
Aadat	0.033912187	0.894536823	0.048401777
D8ERTD82	0.069378465	0.894536823	0.048401777
Olfr544	0.052972125	0.894536823	0.048401777
Foxf2	-0.051171744	0.894536823	0.048401777
Sdhaf1	-0.043390791	0.894536823	0.048401777
Kansl1l	0.028660043	0.894536823	0.048401777
Slc22a14	0.043892163	0.894536823	0.048401777
AAED1	-0.103658689	0.894589911	0.048376004
Eps8	0.040947404	0.894622765	0.048360055
Hras	-0.03875372	0.894652619	0.048345563
Ncbp1	-0.042744728	0.894733736	0.048306187
Slc26a1	-0.039115354	0.894733736	0.048306187
Cyp2s1	0.067436311	0.894801015	0.048273532
Sec14l4	-0.048294389	0.894801015	0.048273532
Pacrg	0.131188764	0.894801015	0.048273532
Alad	0.043054307	0.894801015	0.048273532
Gucy2f	-0.062660261	0.894801015	0.048273532

Dok7	-0.158400095	0.894892696	0.048229037
Tlk1	0.050275859	0.894892696	0.048229037
Zmat1	-0.048800325	0.894892696	0.048229037
Gemin5	0.063879389	0.894892696	0.048229037
Wscd2	0.037595524	0.894892696	0.048229037
Ube2l3	0.079794005	0.894892696	0.048229037
Arxes1	-0.031295924	0.894892696	0.048229037
Cav3	0.044351907	0.894892696	0.048229037
Zfp473	0.047530696	0.894892696	0.048229037
Zbed5	0.062948089	0.894892696	0.048229037
Tnip1	-0.044768629	0.894892696	0.048229037
Tspan9	0.073781745	0.894924641	0.048213534
Fcer1a	0.056643894	0.894924641	0.048213534
Cpsf1	-0.048951548	0.894924641	0.048213534
Fitm2	0.094038471	0.894924641	0.048213534
Mcc	0.107969858	0.894924641	0.048213534
Pax1	0.061120875	0.894924641	0.048213534
Pbx4	0.055380017	0.894924641	0.048213534
Adamts16	0.048128652	0.894924641	0.048213534
Slc22a3	0.041384888	0.894924641	0.048213534
Dennd5a	-0.050642931	0.894924641	0.048213534
Tchp	0.045096251	0.894924641	0.048213534
Agtr1a	-0.076339587	0.894924641	0.048213534
Tm9sf4	0.069969066	0.894924641	0.048213534
Nectin3	-0.069487598	0.894924641	0.048213534
Zfp768	0.111487334	0.894924641	0.048213534
Dctn2	0.061735803	0.894924641	0.048213534
HN1	0.045111998	0.894924641	0.048213534
Nup210	-0.04656838	0.894924641	0.048213534
Nipal3	-0.036865986	0.894924641	0.048213534
Wwp1	-0.052288784	0.894924641	0.048213534
Hddc3	0.034242847	0.894924641	0.048213534
Ankrd37	-0.034999547	0.894924641	0.048213534
Nat14	-0.037278858	0.894924641	0.048213534
Snhg17	0.031550687	0.894924641	0.048213534
Mphosph1	0.051305172	0.894924641	0.048213534
Adamtsl1	0.064592862	0.894924641	0.048213534
Zranb1	-0.058629929	0.894924641	0.048213534
Mycbpap	0.042544464	0.894924641	0.048213534
Sema4a	-0.146166428	0.894924641	0.048213534
Olig3	0.028802866	0.894924641	0.048213534
Ubp1	-0.052434319	0.894924641	0.048213534
Rasgrp4	0.039477046	0.894924641	0.048213534
Rrp36	-0.063996676	0.894924641	0.048213534
Dennd4a	-0.023739313	0.894924641	0.048213534
Metap2	-0.043127936	0.894924641	0.048213534
Abce1	-0.052167286	0.895128453	0.048114638
Pcgf6	-0.050571868	0.895134263	0.048111819

Ccdc158	0.032069846	0.895134263	0.048111819
Uba1	0.03493062	0.895171848	0.048093584
Phf12	0.047628661	0.895236083	0.048062422
Pcdhga6	0.10675343	0.895236083	0.048062422
Sema4b	-0.123056314	0.89527008	0.04804593
KLHL17	0.100020361	0.89527008	0.04804593
Agrn	-0.072194837	0.89556249	0.047904105
Commd9	0.142659214	0.895604651	0.04788366
Ears2	-0.044953612	0.895604651	0.04788366
Timp3	-0.026647822	0.895604651	0.04788366
Vac14	0.048235193	0.895604651	0.04788366
Tmem265	-0.10390588	0.895608908	0.047881596
Cyb5b	0.036597371	0.895886819	0.047746853
Zfp384	-0.083562091	0.896174543	0.047607397
Snrnp27	-0.089409879	0.896218307	0.047586189
Alcam	-0.047398254	0.896354131	0.047520376
BC037034	0.114071591	0.896562761	0.047419304
Letm1	0.11107998	0.897215696	0.047103138
Wbp11	-0.131650305	0.897228844	0.047096773
Sipa1l2	0.037397963	0.897228844	0.047096773
Snapc4	-0.070715825	0.897228844	0.047096773
Coq10b	-0.084477486	0.897228844	0.047096773
H2AFX	-0.097345619	0.897228844	0.047096773
Vill	-0.072431977	0.897228844	0.047096773
Taf12	0.048537339	0.897228844	0.047096773
LYRM5	0.09496777	0.897228844	0.047096773
Usp51	0.053347005	0.897228844	0.047096773
Setd5	0.076947736	0.897228844	0.047096773
Mlxip	0.06499372	0.897228844	0.047096773
Bloc1s6os	-0.109717081	0.897228844	0.047096773
Syt17	-0.060057464	0.897228844	0.047096773
Trip4	0.038021021	0.897228844	0.047096773
Slc35b3	-0.045448407	0.897228844	0.047096773
Armc12	-0.05095242	0.897228844	0.047096773
Fgd4	-0.122506403	0.897228844	0.047096773
Hexim1	-0.048016735	0.897228844	0.047096773
Insr	-0.054363358	0.897228844	0.047096773
Rfx5	-0.041135152	0.897228844	0.047096773
Tnnc2	-0.040953522	0.897228844	0.047096773
Slc44a3	-0.059659135	0.897228844	0.047096773
Rmi1	-0.039000292	0.897228844	0.047096773
Cct6a	0.087137058	0.897228844	0.047096773
Mrpl33	0.035479697	0.897228844	0.047096773
Scamp3	0.071440905	0.897228844	0.047096773
Cfap74	0.070166311	0.897228844	0.047096773
Trnt1	-0.048708536	0.897228844	0.047096773
Tmem183	-0.044472747	0.897228844	0.047096773
Mrpl11	0.047968367	0.897339642	0.047043146

Glod4	0.07390358	0.897452665	0.046988449
Atg12	0.070794338	0.897513181	0.046959165
Znhit1	-0.050618018	0.897637751	0.046898891
Mthfd2l	-0.053623164	0.898154774	0.046648817
Maea	-0.053516019	0.898158328	0.046647099
Bcar3	0.110227859	0.898158328	0.046647099
Zbtb34	0.060866162	0.898158328	0.046647099
Cript	0.039902257	0.898158328	0.046647099
Wif1	-0.066118906	0.898158328	0.046647099
Fbxo28	0.04103884	0.898158328	0.046647099
Esyt2	0.053837408	0.898158328	0.046647099
Bid	0.087775591	0.898158328	0.046647099
Retn	-0.058937032	0.898158328	0.046647099
Aldh5a1	0.038352536	0.898158328	0.046647099
Znrf3	0.096305923	0.898271232	0.046592509
Tmem69	0.062798528	0.898556671	0.046454527
Fam32a	0.042769769	0.898556671	0.046454527
Cdk17	0.19812941	0.898649147	0.046409833
Vmn1r21	-0.042912462	0.898649147	0.046409833
Slc20a1	-0.144468028	0.898696691	0.046386857
Zfp9	-0.118396972	0.898696691	0.046386857
Ccdc112	-0.04671597	0.898696691	0.046386857
ATP5S	-0.055095556	0.898696691	0.046386857
Timm8a2	-0.056599313	0.898696691	0.046386857
Agxt2	-0.03247956	0.898696691	0.046386857
Dazap1	0.066921301	0.898805087	0.046334478
Mbtps2	0.053640695	0.898805087	0.046334478
Eif4g1	0.062490222	0.898805087	0.046334478
Rnf146	-0.081241672	0.898840094	0.046317563
Sdr9c7	0.108852537	0.898840094	0.046317563
Ripply1	-0.057178153	0.898840094	0.046317563
Zfp335	-0.071362572	0.898840094	0.046317563
Cox8a	-0.101325697	0.898840094	0.046317563
Fzd8	0.070013588	0.898840094	0.046317563
Asb8	-0.067249631	0.898840094	0.046317563
Zfp3	-0.093715035	0.898840094	0.046317563
Glb1l2	-0.122365851	0.898840094	0.046317563
Nup210l	-0.180656292	0.898840094	0.046317563
Clmp	-0.148743385	0.898840094	0.046317563
HIST2H3C2	-0.071726442	0.898840094	0.046317563
Senp3	0.039867489	0.898840094	0.046317563
Tsen34	0.054469462	0.898840094	0.046317563
Apobec4	0.063462251	0.898840094	0.046317563
Zfx	0.073250382	0.898840094	0.046317563
Zmynd11	0.068823764	0.898840094	0.046317563
Galnt13	0.046361567	0.898840094	0.046317563
Capn15	0.044920229	0.898840094	0.046317563
Cmtr1	0.054584151	0.898840094	0.046317563

Cfap43	0.07039748	0.898840094	0.046317563
Crebzf	0.067416621	0.898840094	0.046317563
Fcho2	-0.046740872	0.898840094	0.046317563
Ell2	0.034902743	0.898840094	0.046317563
Rpl7l1	0.038577389	0.898840094	0.046317563
Rhbdd2	-0.075429069	0.898840094	0.046317563
Fbxl5	-0.052584706	0.898840094	0.046317563
Avpr1a	0.105463039	0.898840094	0.046317563
Ttc39c	-0.065872792	0.898840094	0.046317563
Hlcs	0.032669783	0.898840094	0.046317563
Ccdc171	-0.048007853	0.898840094	0.046317563
Nbeal1	-0.218062843	0.898840094	0.046317563
Ndufa9	0.035880951	0.898840094	0.046317563
F2rl1	-0.035837269	0.898840094	0.046317563
Ifnlr1	0.03781248	0.898840094	0.046317563
Calml4	0.078496616	0.898840094	0.046317563
Cd55	-0.053697867	0.898840094	0.046317563
Ppp2r3a	0.118121202	0.898840094	0.046317563
Noc2l	0.033879941	0.898840094	0.046317563
Cenpw	0.081486249	0.898840094	0.046317563
Scamp1	-0.045134933	0.898840094	0.046317563
Ppp4r4	-0.045780086	0.898840094	0.046317563
N4bp1	0.037019993	0.898840094	0.046317563
Adamts20	-0.036009996	0.898840094	0.046317563
Bmp3	-0.02618431	0.898840094	0.046317563
Atp6ap1	0.086200592	0.898840094	0.046317563
Spast	0.043499175	0.898840094	0.046317563
FAM159B	0.085005074	0.898840094	0.046317563
Ybx3	-0.027821534	0.898840094	0.046317563
Dars	-0.08970387	0.898844365	0.0463155
Mtmr3	-0.070113187	0.898844365	0.0463155
Erich3	0.049222579	0.898844365	0.0463155
Fiz1	-0.101970392	0.898844365	0.0463155
Ckm	0.093933776	0.898872766	0.046301778
Elmod3	-0.080737786	0.898872766	0.046301778
Zscan25	0.056997162	0.898872766	0.046301778
Prr13	0.048571134	0.898872766	0.046301778
Spcs1	0.134337593	0.898872766	0.046301778
Hspa1a	-0.137628937	0.898872766	0.046301778
Pcbd1	0.22822108	0.898872766	0.046301778
Rlf	0.060342691	0.898872766	0.046301778
Fancd2	0.036600249	0.89904182	0.046220106
Asb6	0.098078613	0.89904182	0.046220106
Arg2	0.067508684	0.89904182	0.046220106
Taar3	-0.050346405	0.89906284	0.046209952
Mau2	0.069098714	0.899069196	0.046206882
Chmp6	0.041031206	0.899069196	0.046206882
Pde6b	-0.05383113	0.899154459	0.046165698

Elmod2	0.146294908	0.899154459	0.046165698
Art5	-0.070896176	0.899154459	0.046165698
Ttc13	-0.030291699	0.899154459	0.046165698
FAM109A	-0.059286039	0.899154459	0.046165698
Rp9	0.036171172	0.899154459	0.046165698
Dph1	0.05609036	0.899154459	0.046165698
Hfm1	-0.086393757	0.899154469	0.046165693
Thoc5	-0.128393442	0.899460449	0.046017929
R3hcc1	-0.078808925	0.899460449	0.046017929
Olfr1393	-0.108217135	0.899460449	0.046017929
Vmn2r118	-0.054279986	0.899460449	0.046017929
Elmo2	-0.108987106	0.899460449	0.046017929
Fbxo40	-0.087336596	0.899460449	0.046017929
Drc1	-0.096078101	0.899460449	0.046017929
Mpc1	0.040473852	0.899460449	0.046017929
Krtap11-1	0.20063705	0.899460449	0.046017929
Tinag	0.067569697	0.899460449	0.046017929
Frmd7	-0.202245182	0.899460449	0.046017929
Aip	0.071568402	0.899460449	0.046017929
Cnnm3	0.043942704	0.899460449	0.046017929
Exoc2	-0.050168075	0.899460449	0.046017929
St7	-0.0712831	0.899460449	0.046017929
Exosc6	0.049257257	0.899460449	0.046017929
Hdc	-0.057411251	0.899460449	0.046017929
Mien1	-0.083453632	0.899460449	0.046017929
Slco4a1	-0.052346933	0.899460449	0.046017929
Galnt5	-0.067892162	0.899460449	0.046017929
Fbxo16	0.047119738	0.899460449	0.046017929
Klb	0.046297838	0.899460449	0.046017929
Ifitm6	-0.053684898	0.899460449	0.046017929
Hmcn2	-0.061895796	0.899460449	0.046017929
Bhlhb9	-0.046727213	0.899460449	0.046017929
Dnmt3b	0.069236045	0.899460449	0.046017929
Myl6	-0.04315993	0.899460449	0.046017929
7-Mar	-0.05861147	0.899460449	0.046017929
AW55491	-0.055513929	0.899460449	0.046017929
Sag	0.060798621	0.899460449	0.046017929
Wipi2	0.062850816	0.899460449	0.046017929
Ppp1r12a	0.056136307	0.899460449	0.046017929
Cfap57	0.049547648	0.899460449	0.046017929
Piwil1	-0.035419254	0.899460449	0.046017929
Med19	-0.037801408	0.899460449	0.046017929
Rps27a	-0.088953864	0.899460449	0.046017929
Rpf2	-0.066900147	0.899460449	0.046017929
WRB	-0.027967156	0.899460449	0.046017929
Ido2	-0.05464968	0.899460449	0.046017929
Zfp948	0.086080048	0.899460449	0.046017929
Spef2	0.064774757	0.899460449	0.046017929

Osbpl8	0.058944464	0.899460449	0.046017929
Zdhhc18	0.055424145	0.899460449	0.046017929
Ipo5	0.061179181	0.899460449	0.046017929
Pp2d1	-0.033598298	0.899460449	0.046017929
Iqcd	-0.07003845	0.899460449	0.046017929
Exoc3l2	0.025611163	0.899460449	0.046017929
Rbm12b2	0.023463629	0.899460449	0.046017929
Ndufa12	0.074362969	0.899467243	0.046014648
Zfp512	-0.085109221	0.899631547	0.045935324
Gria1	0.113694738	0.899705532	0.045899609
Cdrt4	-0.071444036	0.899781591	0.045862896
Ankk1	0.095795429	0.899781591	0.045862896
Gpr139	-0.059611848	0.899781591	0.045862896
Crybb2	0.059977164	0.899781591	0.045862896
Mblac2	-0.117067592	0.899819451	0.045844623
Cdh13	-0.073616396	0.899819451	0.045844623
TROVE2	-0.068342927	0.899819451	0.045844623
Med15	-0.121269074	0.899819451	0.045844623
Pgrmc1	0.078112401	0.899819451	0.045844623
Pls3	-0.092697622	0.899819451	0.045844623
Senp8	0.059338649	0.899819451	0.045844623
Cpne5	-0.067419028	0.899819451	0.045844623
Grhpr	-0.057164734	0.899819451	0.045844623
Tmem241	0.096852941	0.899819451	0.045844623
Agpat3	0.04474087	0.899819451	0.045844623
Hsf3	-0.038492144	0.899819451	0.045844623
Pced1a	-0.068152095	0.899819451	0.045844623
Tcerg1	-0.044475869	0.899819451	0.045844623
Gpr101	-0.15608401	0.899819451	0.045844623
Oc90	-0.041376636	0.899819451	0.045844623
Rcn2	-0.115569632	0.899819451	0.045844623
Slc47a2	-0.051655979	0.899819451	0.045844623
Acsl1	0.065921949	0.899819451	0.045844623
Mrpl16	-0.051005605	0.899819451	0.045844623
Tas1r1	0.055367234	0.899819451	0.045844623
Eif2b1	-0.111883589	0.899819451	0.045844623
Nsdhl	-0.040363964	0.899819451	0.045844623
Zfp771	0.034998378	0.899819451	0.045844623
Stk35	0.074464851	0.899819451	0.045844623
Slain1os	0.059419488	0.899819451	0.045844623
Rnf4	-0.069778152	0.899819451	0.045844623
Irak1	-0.061780795	0.899819451	0.045844623
Map2k2	0.060096502	0.899819451	0.045844623
Cox15	0.152543284	0.899819451	0.045844623
Frat2	0.052537538	0.899819451	0.045844623
Nmu	0.052223629	0.899819451	0.045844623
Efnb1	-0.033935837	0.899819451	0.045844623
Naa10	-0.078837894	0.899819451	0.045844623

Gjd4	-0.052381473	0.899819451	0.045844623
Rnps1	0.067771048	0.899819451	0.045844623
Gorasp1	-0.030383504	0.899819451	0.045844623
Eftud2	0.050679764	0.899819451	0.045844623
Lipm	0.053050573	0.899819451	0.045844623
Myocd	0.056357304	0.899819451	0.045844623
Phf13	-0.053151381	0.899819451	0.045844623
Unc5d	0.034165734	0.899819451	0.045844623
Arl9	-0.066057234	0.899819451	0.045844623
Arid1b	0.135425942	0.899819451	0.045844623
Colgalt2	0.043102027	0.899819451	0.045844623
Cd160	0.048186913	0.899819451	0.045844623
Psmd14	-0.044774977	0.899819451	0.045844623
Fcmr	0.032960596	0.899819451	0.045844623
Pgpep1l	-0.030944713	0.899819451	0.045844623
Ebf4	-0.161523872	0.899819451	0.045844623
Rngtt	0.05113624	0.899819451	0.045844623
Mettl6	-0.09848966	0.899819451	0.045844623
Grm3	0.104267183	0.899819451	0.045844623
Il7	-0.042296774	0.899819451	0.045844623
Kif6	0.068742953	0.899819451	0.045844623
Cables1	-0.036490515	0.899819451	0.045844623
Meioc	-0.034120722	0.899819451	0.045844623
Mkrn3	0.047390958	0.899819451	0.045844623
Smpd1	0.0527233	0.899819451	0.045844623
U2surp	-0.052278768	0.899819451	0.045844623
Klrg1	-0.052515186	0.899819451	0.045844623
CRAMP1L	-0.033806368	0.899819451	0.045844623
Gprin2	0.040353541	0.899819451	0.045844623
Lsm7	-0.086680664	0.899819451	0.045844623
Ift46	-0.028340547	0.899819451	0.045844623
NARFL	-0.03868087	0.899819451	0.045844623
Cdh23	0.054334163	0.899819451	0.045844623
Pnkp	-0.044592526	0.899819451	0.045844623
Lrrc30	0.047361771	0.899819451	0.045844623
Becn1	-0.025249953	0.899819451	0.045844623
Xlr3c	-0.025216627	0.899819451	0.045844623
Gpr182	-0.027661703	0.899819451	0.045844623
Smarca4	0.030983423	0.899819451	0.045844623
Il21	-0.034702849	0.899819451	0.045844623
Lce1a1	-0.048586843	0.899819451	0.045844623
Ctdspl2	0.076767235	0.899819451	0.045844623
Egln1	0.132940102	0.899819451	0.045844623
Deaf1	0.025461225	0.899819451	0.045844623
Htr1b	0.025229253	0.899819451	0.045844623
Ercc6	-0.047754274	0.899986868	0.045763828
Usp22	-0.042179019	0.900020467	0.045747614
Tas2r143	0.047616192	0.900145529	0.045687271

Fam120c	-0.212526406	0.900152322	0.045683994
Dock9	-0.061433496	0.900152322	0.045683994
Pgp	0.245454733	0.900152322	0.045683994
Adam19	0.038716416	0.900152322	0.045683994
FAM159A	-0.033142011	0.900152322	0.045683994
Tceanc	0.042094544	0.900152322	0.045683994
Zfp664	-0.05268615	0.900452525	0.04553918
Parp11	-0.062080755	0.900484289	0.04552386
Rplp1	0.041989751	0.900484289	0.04552386
Aqp8	0.037534986	0.900484289	0.04552386
Pcdh18	0.02624603	0.900484289	0.04552386
Tex14	-0.058184299	0.900548785	0.045492756
Atp1a2	0.02885071	0.900548785	0.045492756
Slc12a3	0.033237218	0.900548785	0.045492756
Btbd17	-0.04372447	0.900548785	0.045492756
Snx12	-0.070553113	0.900621084	0.04545789
Bcl6b	0.046777223	0.900621084	0.04545789
Nppb	0.172930904	0.900621084	0.04545789
Krt23	-0.03424522	0.900621084	0.04545789
Sertad4	0.025078671	0.900621084	0.04545789
Coq6	0.049666265	0.900928729	0.045309564
Ppp1r3a	0.102476313	0.900928729	0.045309564
Dnajc2	0.047490502	0.900928729	0.045309564
Htr5b	-0.041766784	0.900928729	0.045309564
Ubiad1	-0.05585506	0.900961685	0.045293678
Cped1	-0.049633371	0.900961685	0.045293678
Lrp2bp	-0.048537002	0.900961685	0.045293678
Hcfc2	0.041269543	0.900961685	0.045293678
MFSD7C	0.041482228	0.900961685	0.045293678
Mnt	-0.037134812	0.900961685	0.045293678
Usp3	0.05576378	0.900961685	0.045293678
Lbhd1	-0.068520899	0.900961685	0.045293678
Rps23	-0.059628025	0.900961685	0.045293678
Xlr4c	-0.07827691	0.900961685	0.045293678
Otulin	-0.07101801	0.900961685	0.045293678
Odf3l2	-0.129352316	0.900961685	0.045293678
Zfp775	0.10329155	0.900961685	0.045293678
TMEM57	0.050746134	0.900961685	0.045293678
Krtcap3	-0.067380923	0.900961685	0.045293678
Plcd1	-0.03304157	0.900961685	0.045293678
Sec31a	-0.08830224	0.900961685	0.045293678
Lrp2	0.051983671	0.900961685	0.045293678
Arl4a	-0.037179548	0.900961685	0.045293678
Ftsj3	-0.035678139	0.900961685	0.045293678
Six3os1	-0.068986878	0.900961685	0.045293678
Cacng4	-0.040449714	0.900961685	0.045293678
Nup133	0.042294247	0.900961685	0.045293678
Pank2	-0.037663379	0.900961685	0.045293678

Fem1a	-0.063452564	0.900961685	0.045293678
Ccdc126	0.06881453	0.900961685	0.045293678
Plg	0.04552241	0.900961685	0.045293678
Tas2r113	-0.04358565	0.900961685	0.045293678
MUM1L1	-0.071229445	0.900961685	0.045293678
Cldn3	0.031449662	0.900961685	0.045293678
Chd4	-0.050272312	0.900961685	0.045293678
Ppp1r1c	-0.028678653	0.900961685	0.045293678
Rab4b	-0.042897426	0.900961685	0.045293678
Asb9	-0.024936253	0.900961685	0.045293678
Tpte	-0.055123879	0.900961685	0.045293678
Clec3b	-0.035487637	0.900961685	0.045293678
Mtx3	-0.049218347	0.900961685	0.045293678
Arl15	0.107060884	0.900961685	0.045293678
Hdhd2	0.064426547	0.900961685	0.045293678
Tmem63b	0.054574321	0.900961685	0.045293678
Cct8	0.048916505	0.900961685	0.045293678
Adra2a	0.024882124	0.900961685	0.045293678
Zfp932	-0.083850723	0.901106785	0.04522374
Enpep	0.053723619	0.901197307	0.045180115
Maoa	-0.056847222	0.901197307	0.045180115
Lime1	-0.152476588	0.901459638	0.045053714
Leng1	-0.142914188	0.901459638	0.045053714
Kdm4b	-0.078078438	0.901459638	0.045053714
Taf3	0.068944187	0.901459638	0.045053714
Ppat	-0.034880509	0.901459638	0.045053714
Tcp11	-0.040943973	0.901459638	0.045053714
Slc35e3	0.045227741	0.901652757	0.044960685
AI429214	0.056544696	0.901652757	0.044960685
Tspo2	-0.117091137	0.902226628	0.04468436
Tmem231	-0.089874456	0.902252012	0.044672141
Mettl18	-0.056330988	0.902252012	0.044672141
Mybpc2	-0.08650145	0.902379982	0.044610548
Veph1	-0.096707752	0.902379982	0.044610548
Fam122a	-0.171433224	0.902379982	0.044610548
Cox14	-0.046647216	0.902379982	0.044610548
Rdh1	0.077134381	0.902379982	0.044610548
Lrch1	0.085428574	0.902379982	0.044610548
Klf16	0.040678368	0.902379982	0.044610548
Slc25a48	0.064717636	0.902379982	0.044610548
Aoc1	-0.044558191	0.902379982	0.044610548
Vps29	0.0428377	0.902379982	0.044610548
TMEM28	0.051206908	0.902379982	0.044610548
Hdac7	0.024029145	0.902379982	0.044610548
Ppp1r3e	-0.037933184	0.902379982	0.044610548
Zswim4	-0.064628347	0.902379982	0.044610548
Ubac2	-0.061234447	0.902379982	0.044610548
Birc2	0.047297905	0.902379982	0.044610548

Nudc	-0.03819114	0.902379982	0.044610548
Il15ra	-0.022225141	0.902379982	0.044610548
Gnas	0.031873767	0.902379982	0.044610548
Rpl37rt	0.059194036	0.902379982	0.044610548
Unc5cl	-0.048791487	0.902379982	0.044610548
Top1	0.034086139	0.902434908	0.044584114
Sec62	0.04101586	0.902434908	0.044584114
Fhit	-0.106224324	0.902530539	0.044538094
Smarcd3	0.038738861	0.902598746	0.044505274
Hook2	-0.087863474	0.902649441	0.044480882
Spic	0.069390405	0.902649441	0.044480882
HMGA1-RS	-0.065844493	0.902649441	0.044480882
Ints4	-0.051827801	0.902649441	0.044480882
Csta1	-0.048693728	0.902649441	0.044480882
Lingo3	0.046996551	0.902649441	0.044480882
Slc26a4	-0.046374239	0.902649441	0.044480882
Slc38a5	-0.037125211	0.902649441	0.044480882
Mmachc	-0.083246534	0.902649441	0.044480882
Nop16	-0.028544963	0.902649441	0.044480882
Myh8	-0.045232065	0.902649441	0.044480882
Fdxacb1	0.063915877	0.902649441	0.044480882
Tmem245	0.05306638	0.902649441	0.044480882
Magix	-0.046841189	0.902649441	0.044480882
Igsf23	-0.058753685	0.902649441	0.044480882
Gcsh	0.04572762	0.902649441	0.044480882
Yrdc	-0.051179354	0.902649441	0.044480882
Pcdha8	0.044424621	0.902649441	0.044480882
Dhx35	-0.031746474	0.902649441	0.044480882
Lca5	0.04688175	0.902649441	0.044480882
Edn1	-0.039057775	0.902649441	0.044480882
Srp54c	-0.024547133	0.902649441	0.044480882
Slc22a23	-0.024536564	0.902649441	0.044480882
Bhmt2	0.058080491	0.902649441	0.044480882
Ass1	0.045704154	0.902671371	0.044470331
Syap1	0.052034622	0.902871721	0.044373949
Tubgcp4	0.035589642	0.902950705	0.044335959
Arhgef16	-0.108672619	0.90299946	0.04431251
Gprc5b	0.061485183	0.90299946	0.04431251
Akap9	-0.035120432	0.90299946	0.04431251
Zfp316	-0.030193127	0.90299946	0.04431251
Idi2	-0.064914755	0.90299946	0.04431251
Pnisr	0.04186712	0.90299946	0.04431251
MPP6	-0.048636747	0.903013142	0.044305929
Dnajc22	0.038461857	0.903156303	0.044237083
Zcchc17	-0.040670749	0.903156303	0.044237083
Catsper4	0.041998582	0.903294185	0.044170786
Fam83e	-0.032715567	0.903526896	0.044058915
Trappc2	0.072698274	0.903555669	0.044045085

Itga4	0.037207051	0.903555669	0.044045085
Rad51ap2	-0.024399709	0.903555669	0.044045085
Hmox2	-0.029871916	0.903555669	0.044045085
Ipp	0.024406847	0.903555669	0.044045085
Adipoq	-0.088607008	0.903633794	0.044007536
AA387200	-0.074945956	0.903784633	0.043935047
Itsn2	-0.152355224	0.903784633	0.043935047
Rcor2	0.070849182	0.903784633	0.043935047
Acacb	-0.033745571	0.903784633	0.043935047
Fryl	0.078267073	0.903784633	0.043935047
Mdh2	-0.062325525	0.903784633	0.043935047
Fam133b	0.065456909	0.903784633	0.043935047
Zcchc3	0.045481785	0.903784633	0.043935047
Ndufaf4	0.110245992	0.903784633	0.043935047
Wwc2	-0.048141357	0.903784633	0.043935047
Ostm1	0.035510416	0.903784633	0.043935047
Atraid	0.059460481	0.903784633	0.043935047
Mob4	0.051101833	0.903784633	0.043935047
Tmem182	0.040121957	0.903784633	0.043935047
Rrp1	-0.048445937	0.903784633	0.043935047
Slc25a33	0.047847281	0.903784633	0.043935047
Paqr7	0.03622117	0.903784633	0.043935047
Gak	0.039480571	0.903784633	0.043935047
Zim2	-0.024302495	0.903784633	0.043935047
Grb2	0.041084355	0.903784633	0.043935047
Pigc	0.058060915	0.903784633	0.043935047
Gp1ba	0.035005525	0.903784633	0.043935047
Tmem161	0.071235075	0.903784633	0.043935047
Sesn1	0.026701196	0.903784633	0.043935047
Cdh1	0.041356639	0.903793619	0.043930729
Dgat1	-0.042188691	0.903979	0.043841659
Tbc1d24	0.037604695	0.903979	0.043841659
Caly	0.040651513	0.903979	0.043841659
Ckb	-0.062793314	0.903979	0.043841659
Tnrc18	-0.069114533	0.904105576	0.043780852
Dnajc1	0.075446275	0.904105576	0.043780852
Nxf3	0.132361289	0.904147577	0.043760677
Gpr20	0.040154963	0.904147577	0.043760677
Zfp786	-0.031625049	0.904147577	0.043760677
Slx4	-0.050786351	0.904155057	0.043757084
Nup155	0.027765666	0.904155057	0.043757084
Taok3	0.150895137	0.90437223	0.043652782
Ten1	0.087946421	0.90437223	0.043652782
Mrps14	-0.036117697	0.90437223	0.043652782
Tdg	-0.046516069	0.904418395	0.043630613
Zfp574	0.052590765	0.9044738	0.043604009
Ntmt1	-0.052330485	0.9044738	0.043604009
Kif5b	0.042361898	0.9044738	0.043604009

Hspd1	0.083198297	0.9044738	0.043604009
Pde1b	0.08621279	0.9044738	0.043604009
Utp18	-0.045510901	0.9044738	0.043604009
Pold2	-0.051948477	0.9044738	0.043604009
Speer3	-0.057909654	0.9044738	0.043604009
Olfr320	0.070634929	0.9044738	0.043604009
Slc39a6	0.039466179	0.9044738	0.043604009
Strn	-0.077887914	0.90450124	0.043590833
Abca7	-0.073401709	0.90450124	0.043590833
Kcna6	-0.045419784	0.90450124	0.043590833
Nfyb	-0.078919632	0.90450124	0.043590833
Ddrgk1	-0.059559774	0.90450124	0.043590833
Ints5	-0.108363839	0.90450124	0.043590833
Hspb11	-0.068951236	0.90450124	0.043590833
Churc1	0.060871453	0.90450124	0.043590833
Ube2e3	-0.048238268	0.90450124	0.043590833
Map2k5	-0.094323035	0.90450124	0.043590833
Chst4	0.091420193	0.90450124	0.043590833
Slc2a2	-0.046253519	0.90450124	0.043590833
Krba1	-0.035897037	0.90450124	0.043590833
Thumpd1	0.030015375	0.90450124	0.043590833
Zfp809	0.063766043	0.90450124	0.043590833
Fbxo10	-0.034172675	0.90450124	0.043590833
Psmd3	0.055980607	0.90450124	0.043590833
Kif12	0.055776329	0.90450124	0.043590833
Fa2h	-0.033616718	0.90450124	0.043590833
Rap1gap	-0.069550573	0.90450124	0.043590833
Zfp729b	-0.106965977	0.90450124	0.043590833
Rab4a	0.052727417	0.90450124	0.043590833
Srp54a	0.035023133	0.90450124	0.043590833
Pkp4	-0.040505995	0.90450124	0.043590833
Avpr1b	0.031867458	0.90450124	0.043590833
Lrit3	-0.03843113	0.90450124	0.043590833
Sntn	-0.093464678	0.90450124	0.043590833
Snhg20	0.061275457	0.90450124	0.043590833
Slc13a1	-0.081858766	0.90450124	0.043590833
Baiap2	0.033892983	0.90450124	0.043590833
OLFR329-F	-0.038485368	0.90450124	0.043590833
Il5	-0.061320825	0.90450124	0.043590833
Phip	-0.063768618	0.90450124	0.043590833
Cep295	0.043981256	0.90450124	0.043590833
Trappc13	-0.024037629	0.90450124	0.043590833
Trappc10	-0.023995366	0.90450124	0.043590833
Apol7e	-0.040351902	0.90450124	0.043590833
Zbtb24	0.130839232	0.90450124	0.043590833
Nanos1	0.024132109	0.90450124	0.043590833
Mark4	-0.08349955	0.904815147	0.043440138
Rasl10a	-0.061903758	0.904815147	0.043440138

Lrat	-0.042560966	0.904815147	0.043440138
Dcaf10	0.168181335	0.904815147	0.043440138
Psg17	-0.034863639	0.904815147	0.043440138
Cstf3	-0.056724642	0.904815147	0.043440138
Olfr19	-0.040829721	0.904815147	0.043440138
Srsf12	-0.035074611	0.904815147	0.043440138
Hcfc1r1	0.077042431	0.904815147	0.043440138
Tceal8	-0.05186536	0.904815147	0.043440138
Fam110c	-0.08364593	0.904815147	0.043440138
Tbcel	0.09766053	0.904815147	0.043440138
Csnk1d	-0.039213712	0.904815147	0.043440138
Zfp729a	0.042904203	0.904815147	0.043440138
Dpf1	0.102552402	0.904815147	0.043440138
Hipk1	0.028167286	0.904815147	0.043440138
Ccdc6	0.055798058	0.904815147	0.043440138
Wfdc3	-0.109338752	0.904815147	0.043440138
Pmpcb	-0.068563961	0.904815147	0.043440138
Prpf3	0.06498223	0.904815147	0.043440138
Fhad1os2	0.032175289	0.904815147	0.043440138
Ppard	-0.037265359	0.904815147	0.043440138
Sike1	-0.036455247	0.904815147	0.043440138
Celf4	0.043680785	0.904815147	0.043440138
Ntsr1	-0.041845262	0.904815147	0.043440138
Tatdn2	-0.051969277	0.904815147	0.043440138
Lin37	0.034226003	0.904815147	0.043440138
Zfp1	0.060766478	0.904815147	0.043440138
Eif3m	0.060246886	0.904815147	0.043440138
Pfas	0.038697911	0.904815147	0.043440138
Scaf11	0.042508536	0.904815147	0.043440138
Slc7a10	-0.041228737	0.904815147	0.043440138
FAM92B	0.038636229	0.904815147	0.043440138
Slc22a8	0.094112317	0.904815147	0.043440138
Hars	0.053355793	0.904815147	0.043440138
Rtn4	0.061035271	0.904815147	0.043440138
Tmem131	0.036651303	0.904815147	0.043440138
Pde11a	-0.097442139	0.90491168	0.043393806
Edc4	-0.088442131	0.90491168	0.043393806
Mtmr2	0.055630474	0.90491168	0.043393806
Bop1	0.077722635	0.90491168	0.043393806
Higd2a	0.046938499	0.90491168	0.043393806
Cep135	0.086621323	0.90491168	0.043393806
Eif2s1	0.147747001	0.90491168	0.043393806
Tfb2m	0.040392639	0.90491168	0.043393806
FDX1L	-0.072145347	0.90491168	0.043393806
Olfr156	0.039224808	0.90491168	0.043393806
Gpatch11	-0.071516511	0.90491168	0.043393806
Bcap31	0.046226192	0.90491168	0.043393806
Ogdh	0.042067274	0.90491168	0.043393806

Neil2	-0.049253046	0.90491168	0.043393806
Nup85	0.035318825	0.90491168	0.043393806
Tfam	0.060888483	0.90491168	0.043393806
Avl9	0.044649618	0.90491168	0.043393806
Polr2f	0.051831493	0.90491168	0.043393806
Pcyox1l	0.050954597	0.90491168	0.043393806
Gabrp	-0.040626894	0.90491168	0.043393806
Eif1	0.03914169	0.90491168	0.043393806
Ssr2	-0.082948244	0.90491168	0.043393806
Mrpl51	-0.063922284	0.90491168	0.043393806
Mrpl44	-0.040538236	0.90491168	0.043393806
S1pr1	0.075546787	0.90491168	0.043393806
Nsun2	-0.046548762	0.90491168	0.043393806
Tmem107	0.039316134	0.90491168	0.043393806
Thbd	-0.034480731	0.90491168	0.043393806
Tmem209	-0.023767075	0.90491168	0.043393806
Sun3	0.041736857	0.90491168	0.043393806
Plekhd1os	0.028932715	0.90491168	0.043393806
Hes1	0.025033509	0.90491168	0.043393806
Fzr1	-0.037745151	0.90491168	0.043393806
Cdca4	0.023693255	0.90491168	0.043393806
TMEM254E	0.023684693	0.90491168	0.043393806
Lrp12	-0.051560851	0.905070732	0.043317479
Drg2	-0.085458462	0.905113371	0.043297019
Olfr1375	-0.222726291	0.905113371	0.043297019
Olfr54	-0.074216219	0.905113371	0.043297019
Olfr1359	-0.041189827	0.905113371	0.043297019
Hrh3	-0.051772198	0.905113371	0.043297019
Lig3	-0.057031012	0.905113371	0.043297019
Hao2	0.037234626	0.905113371	0.043297019
S100pbp	0.037788682	0.905113371	0.043297019
Rab23	-0.040868225	0.905113371	0.043297019
Sult1a1	0.044287161	0.905113371	0.043297019
Clrn3	0.100469153	0.905113371	0.043297019
Anapc2	0.108208423	0.905113371	0.043297019
Ctla4	-0.083212977	0.905113371	0.043297019
Rsrc2	-0.050570447	0.905113371	0.043297019
Obsl1	-0.026588938	0.905113371	0.043297019
Gpr156	-0.023616345	0.905113371	0.043297019
AI413582	0.036113087	0.905113371	0.043297019
Ubxn4	0.055463422	0.905118052	0.043294773
Txndc12	-0.169527569	0.905118052	0.043294773
Eef1e1	-0.080053827	0.905380902	0.043168671
Mgll	0.033995672	0.905451707	0.043134708
Rnf2	0.13731263	0.905451707	0.043134708
Ndufb5	0.041401563	0.905451707	0.043134708
Vapa	0.097812407	0.905451707	0.043134708
Comt	0.050975705	0.905451707	0.043134708

Olfr239	-0.088372865	0.905451707	0.043134708
Cntn2	0.038680721	0.905451707	0.043134708
Tmem128	-0.07141568	0.905451707	0.043134708
Trdn	-0.040484385	0.905451707	0.043134708
Prrc2a	0.054176675	0.905451707	0.043134708
Naif1	0.049554549	0.905451707	0.043134708
Cyb5r4	-0.041518275	0.905451707	0.043134708
Sbp	0.094923861	0.905451707	0.043134708
Ahrr	0.032967438	0.905451707	0.043134708
Dspp	0.056783094	0.90548908	0.043116783
Maf1	0.024257476	0.90548908	0.043116783
Arl4c	0.050191323	0.90548908	0.043116783
Slc22a1	0.103993953	0.905502074	0.043110551
Rab9	-0.052580089	0.905502074	0.043110551
Zfp777	-0.058113068	0.905502074	0.043110551
Ppp2r2cos	0.040106706	0.905502074	0.043110551
Tmem163	-0.038057267	0.90572071	0.043005702
Zc4h2	-0.029239607	0.90572071	0.043005702
Ube2d2b	-0.030366231	0.905979893	0.042881441
Kir3dl2	-0.050894331	0.90598897	0.042877089
Cfap54	-0.100614808	0.906013685	0.042865242
Adcy4	0.036884757	0.906013685	0.042865242
Zzef1	0.190794224	0.906013685	0.042865242
Pigu	0.03790934	0.906013685	0.042865242
Mmrn1	0.057886943	0.906013685	0.042865242
Rerg	-0.02342127	0.906013685	0.042865242
Ppp1r21	0.084599546	0.906013685	0.042865242
Zfp354c	0.061202994	0.90603046	0.042857201
Slc38a2	0.07063112	0.90603046	0.042857201
Brat1	-0.041373183	0.906251504	0.04275126
Tbc1d32	0.04698675	0.906261719	0.042746365
Hpca	0.042057201	0.906419394	0.042670811
Med30	0.097945782	0.906419394	0.042670811
Basp1	-0.042470918	0.906796368	0.042490228
Cebpe	0.047538094	0.906883788	0.042448362
Arl2bp	0.034913677	0.906883788	0.042448362
Cpeb4	0.198240478	0.906883788	0.042448362
Ccdc57	-0.085348965	0.906941215	0.042420862
Mpv17l2	-0.036567049	0.906941215	0.042420862
Orc6	0.06657033	0.906941215	0.042420862
Mogat1	-0.119566856	0.907024603	0.042380933
Chmp2a	-0.179557866	0.907024603	0.042380933
Mroh8	0.092736355	0.907024603	0.042380933
Cir1	0.060418536	0.907024603	0.042380933
Atrip	0.062064371	0.907024603	0.042380933
Elk1	0.077050452	0.907024603	0.042380933
Chn2	0.048218569	0.907024603	0.042380933
Cntnap5b	-0.039631298	0.907024603	0.042380933

Gprc5a	-0.043039752	0.907024603	0.042380933
Higd1a	-0.05046472	0.907024603	0.042380933
Vmn2r27	0.084259265	0.907024603	0.042380933
Dennd2d	-0.09143507	0.907030653	0.042378036
Trappc2l	-0.095604281	0.907154054	0.042318954
Abi3bp	-0.099703011	0.907154054	0.042318954
Tuft1	0.04699428	0.907154054	0.042318954
Slc26a3	0.081249632	0.90731322	0.042242761
Trim80	0.072143825	0.90731322	0.042242761
Neu1	0.043150417	0.90731322	0.042242761
Slc4a1ap	-0.087465458	0.90731322	0.042242761
Mcfd2	-0.053161062	0.90731322	0.042242761
Pigk	0.057417525	0.90731322	0.042242761
Olfr574	-0.050282234	0.90731322	0.042242761
Puf60	-0.04039564	0.90731322	0.042242761
Ubc	0.031858044	0.90731322	0.042242761
Commd3	-0.044956602	0.90731322	0.042242761
Dusp12	-0.033611439	0.90731322	0.042242761
Zfp712	0.02801301	0.90731322	0.042242761
Slc16a8	0.079751792	0.90731322	0.042242761
Trim24	0.279891839	0.90731322	0.042242761
Reps1	0.101889124	0.90731322	0.042242761
Fermt1	0.047090135	0.90731322	0.042242761
Smim5	-0.0419782	0.90731322	0.042242761
Srsf3	0.074362927	0.907468687	0.042168352
Cdc42ep3	0.087003234	0.907468687	0.042168352
Insm1	-0.051811527	0.907468687	0.042168352
Kcnj2	0.050248672	0.907468687	0.042168352
Trub2	0.057792866	0.907468687	0.042168352
Tbc1d4	0.026367006	0.907468687	0.042168352
Atg5	-0.0631517	0.907468687	0.042168352
Ppp1r27	-0.035252264	0.907468687	0.042168352
Pik3c2b	-0.111716761	0.907468687	0.042168352
Ccdc174	0.036372924	0.907468687	0.042168352
Lrba	-0.020629535	0.907468687	0.042168352
Setd1a	-0.032694296	0.907468687	0.042168352
Gfi1	0.039451835	0.907468687	0.042168352
Tm2d2	0.050200513	0.907545818	0.04213144
Olfr982	0.03451603	0.907545818	0.04213144
Itpa	0.058398717	0.907545818	0.04213144
Tnfrsf25	0.045446436	0.907545818	0.04213144
Hp1bp3	0.023086044	0.907834172	0.041993474
Emc7	-0.045676192	0.907960413	0.041933086
Cmc1	-0.064364569	0.90801385	0.041907527
Cma1	0.051963437	0.90801385	0.041907527
Rac1	0.050014555	0.90801385	0.041907527
Prkg1	0.071333513	0.90801385	0.041907527
Bbs2	0.051098918	0.90801385	0.041907527

Dph6	0.059844995	0.90801385	0.041907527
Ilvbl	-0.055004757	0.90801385	0.041907527
Psmb11	-0.044868567	0.90801385	0.041907527
Fscn2	0.034782514	0.90801385	0.041907527
Ksr1	0.036867926	0.90801385	0.041907527
Atxn1l	-0.032759299	0.90801385	0.041907527
Sv2c	-0.037282269	0.90801385	0.041907527
Tbc1d20	-0.041685002	0.90801385	0.041907527
Ccdc15	0.079218847	0.90801385	0.041907527
Awat2	0.033641444	0.90801385	0.041907527
Rpl31-ps1	0.033923601	0.90801385	0.041907527
AI463229	-0.035719442	0.90801385	0.041907527
Per3	0.029048326	0.90801385	0.041907527
Dhx36	0.046145234	0.90801385	0.041907527
Prss8	0.078158151	0.90801385	0.041907527
Reg3g	0.063369982	0.90801385	0.041907527
Tcf7	0.022209912	0.90801385	0.041907527
Clec2f	-0.023028385	0.90801385	0.041907527
Zfp811	-0.034604487	0.90801385	0.041907527
Il13ra2	0.023840388	0.90801385	0.041907527
Gabrr1	-0.101357438	0.90801385	0.041907527
Aqp11	0.023006748	0.90801385	0.041907527
Ces2b	0.022981358	0.90801385	0.041907527
Bckdk	-0.095896095	0.908201979	0.041817556
Rpap3	-0.115443593	0.908230441	0.041803946
Srgap2	-0.149957537	0.908230441	0.041803946
Traf2	0.076482003	0.908230441	0.041803946
Trpc4ap	0.049804417	0.908230441	0.041803946
Ifi202b	-0.067473404	0.908230441	0.041803946
BB218582	0.058925172	0.908230441	0.041803946
Cldn6	0.067045952	0.908230441	0.041803946
Hormad2	0.045405522	0.908230441	0.041803946
Csrnp2	0.105565013	0.908230441	0.041803946
Gnptg	0.034085459	0.908230441	0.041803946
Dbp	0.102771928	0.908230441	0.041803946
Cc2d1a	0.040535059	0.908230441	0.041803946
Rdh9	-0.051962486	0.908230441	0.041803946
Trim8	-0.045521965	0.908230441	0.041803946
Emg1	0.122756974	0.908230441	0.041803946
Sstr5	-0.029798605	0.908230441	0.041803946
B3galnt2	-0.042041068	0.908230441	0.041803946
Cpa3	-0.026262394	0.908230441	0.041803946
Fev	-0.075119554	0.908230441	0.041803946
Ufc1	-0.032660834	0.908292454	0.041774294
Scara5	-0.054553933	0.908330622	0.041756044
Nck1	0.05783413	0.90838652	0.041729319
Ttc37	-0.059805003	0.90838652	0.041729319
Fasl	0.025006693	0.90838652	0.041729319

Pou4f3	0.059809618	0.90838652	0.041729319
Cma2	0.022852441	0.90838652	0.041729319
Apol7c	-0.028246961	0.908473508	0.041687733
Maz	-0.118163564	0.908535255	0.041658216
Ern2	0.041697707	0.908535255	0.041658216
Fbxw4	0.066255064	0.908535255	0.041658216
Cers1	-0.048188116	0.908535255	0.041658216
Ep300	-0.071081785	0.908535255	0.041658216
Ndufb8	-0.140659859	0.908535255	0.041658216
Ptbp2	-0.030814785	0.908535255	0.041658216
Prkd1	-0.051160887	0.908535255	0.041658216
Atp2c1	-0.038472514	0.908535255	0.041658216
Zfp282	0.042401739	0.908535255	0.041658216
Itgb3bp	-0.043467852	0.908535255	0.041658216
Pirt	0.027990536	0.908535255	0.041658216
Psmc2	-0.054103382	0.908535255	0.041658216
Ndufv2	-0.083883226	0.908535255	0.041658216
Mapk1ip1	-0.061925149	0.908535255	0.041658216
Sntb1	-0.03366025	0.908535255	0.041658216
Ackr2	0.055555666	0.908535255	0.041658216
Golga1	0.041984808	0.908535255	0.041658216
Akr1cl	0.042417089	0.908535255	0.041658216
Nup37	0.045499781	0.908535255	0.041658216
Ankrd12	0.036756234	0.908535255	0.041658216
Eri2	0.038783242	0.908535255	0.041658216
Eif4ebp3	0.036832473	0.908535255	0.041658216
Cog5	-0.066305197	0.908535255	0.041658216
Pradc1	-0.046825684	0.908535255	0.041658216
Actbl2	-0.039346955	0.908535255	0.041658216
Tle4	0.036480296	0.908535255	0.041658216
Clpb	-0.03728649	0.908535255	0.041658216
Zfp57	-0.026790641	0.908535255	0.041658216
Klk13	0.045835257	0.908535255	0.041658216
Ly6g6f	-0.034959445	0.908535255	0.041658216
Zfp780b	-0.037991573	0.908535255	0.041658216
Mbd3	0.07623286	0.908535255	0.041658216
Msl2	-0.040744834	0.908535255	0.041658216
Ptp4a3	-0.028442242	0.908535255	0.041658216
Gstt4	-0.028328182	0.908535255	0.041658216
Akap5	0.062658071	0.908535255	0.041658216
Xkr7	0.021268119	0.908535255	0.041658216
Prps1	0.043024173	0.908535255	0.041658216
Cox7a2	-0.048241711	0.908535255	0.041658216
Pdgfra	0.027262416	0.908535255	0.041658216
Yipf1	0.056527614	0.908535255	0.041658216
Kars	0.036769967	0.908540913	0.041655511
Rcan1	-0.056519835	0.908540913	0.041655511
B3galt4	0.031815321	0.908540913	0.041655511

Zfp963	-0.031948736	0.908540913	0.041655511
Baat	0.080933306	0.908540913	0.041655511
Srsf10	-0.058426058	0.908540913	0.041655511
Btaf1	0.059685266	0.908540913	0.041655511
Cog4	-0.040494091	0.908540913	0.041655511
Hrg	0.032891596	0.908540913	0.041655511
Luzp2	0.0827412	0.908540913	0.041655511
Bbs5	-0.076696772	0.908540913	0.041655511
Ndufc1	0.050603851	0.908540913	0.041655511
Xiap	-0.072571763	0.908601263	0.041626664
Jmjd7	0.139611622	0.908637036	0.041609565
Znhit6	-0.061115474	0.908733767	0.041563334
Dffb	0.0531613	0.90875745	0.041552016
Nsd1	0.034290676	0.90875745	0.041552016
Ebag9	-0.046979735	0.908839762	0.04151268
Lin54	-0.085090101	0.908963033	0.041453779
Gtf3c4	-0.0993005	0.908963033	0.041453779
Sdccag8	0.037893654	0.908963033	0.041453779
Unkl	0.051310452	0.908963033	0.041453779
Cnot10	0.102284275	0.909217066	0.041332421
Dimt1	-0.088560273	0.909217066	0.041332421
Vegfb	-0.052547762	0.909217066	0.041332421
Zfp954	0.094364987	0.909217066	0.041332421
Ccdc85c	0.027210258	0.909217066	0.041332421
SNHG7	0.043934653	0.909217066	0.041332421
Capza2	-0.051329621	0.909217066	0.041332421
Ap2s1	-0.022537497	0.909217066	0.041332421
Spsb4	-0.078895611	0.909290183	0.041297498
Map3k19	0.057671071	0.909290183	0.041297498
Cers6	-0.079542253	0.909290183	0.041297498
B3gntl1	0.049206422	0.909290183	0.041297498
Gtsf1l	0.071366959	0.909290183	0.041297498
Bcas3	-0.029333443	0.909290183	0.041297498
Tceanc2	0.048741661	0.909290183	0.041297498
Ppan	-0.085217536	0.909290183	0.041297498
Lipk	0.03573508	0.909290183	0.041297498
Smad9	0.056290477	0.909290183	0.041297498
Npy	0.077232852	0.909290183	0.041297498
Psd2	0.058623898	0.909290183	0.041297498
Sptlc3	0.037855267	0.909290183	0.041297498
Pex13	-0.044592781	0.909290183	0.041297498
Foxl1	-0.042335653	0.909290183	0.041297498
Zfp750	0.036546641	0.909290183	0.041297498
Syt15	0.054430943	0.909290183	0.041297498
Rab5a	0.03158103	0.909290183	0.041297498
Casr	0.028874541	0.909290183	0.041297498
Fam98b	-0.05010438	0.909290183	0.041297498
Senp7	0.041957662	0.909290183	0.041297498

Ap4e1	-0.029140674	0.909290183	0.041297498
Prss56	-0.029439289	0.909290183	0.041297498
Hao1	0.025786981	0.909290183	0.041297498
FAM134B	-0.036513517	0.909290183	0.041297498
Samd8	-0.066272937	0.909323831	0.041281427
Oca2	0.109547417	0.909323831	0.041281427
Dnaja3	-0.062305162	0.909323831	0.041281427
Strada	0.036164442	0.909323831	0.041281427
Aacs	0.041398119	0.909323831	0.041281427
Xkr6	0.053986545	0.909517316	0.041189028
Rapgef1	-0.025067743	0.909546675	0.04117501
Ulk4	0.044155846	0.909569921	0.04116391
Sf1	0.03870049	0.909739911	0.041082752
Prps1l3	-0.052501441	0.909803528	0.041052383
Spef1	-0.045227547	0.909813773	0.041047493
Pnma5	0.065535655	0.909813773	0.041047493
Has1	0.198884902	0.909813773	0.041047493
Rcor1	-0.053284761	0.909813773	0.041047493
Tmed10	0.22238021	0.909813773	0.041047493
Abhd14b	0.04057884	0.909813773	0.041047493
Otud6a	-0.043919644	0.909813773	0.041047493
Mrps34	0.101524754	0.909813773	0.041047493
Unc119	0.038979108	0.909813773	0.041047493
Coq9	0.090293465	0.909813773	0.041047493
Slc12a8	-0.041855186	0.909813773	0.041047493
Abcf3	-0.037169725	0.909813773	0.041047493
Rps15a	-0.058471082	0.909813773	0.041047493
Vwce	-0.041862047	0.909813773	0.041047493
Dgkg	-0.08067585	0.909813773	0.041047493
Nyx	-0.044127561	0.909813773	0.041047493
Dbhos	-0.100703978	0.909847917	0.041031195
Fam47e	-0.053590166	0.909847917	0.041031195
Btla	-0.078772346	0.909847917	0.041031195
Rpl26	-0.074807888	0.909847917	0.041031195
Abca8b	-0.155100226	0.909847917	0.041031195
Lbx1	0.096076464	0.909847917	0.041031195
Blzf1	-0.042185703	0.909847917	0.041031195
Sec24a	-0.030633596	0.909847917	0.041031195
Mmp21	0.036690002	0.909847917	0.041031195
Fut8	0.084667775	0.909847917	0.041031195
Mllt3	0.064705625	0.909847917	0.041031195
Chrdl2	0.042823021	0.909847917	0.041031195
Tcstv1	-0.058852282	0.909847917	0.041031195
Usp46	0.035747073	0.909847917	0.041031195
Galnt2	-0.051886339	0.909847917	0.041031195
Papss1	0.100374163	0.909847917	0.041031195
Tmem95	-0.076884654	0.909847917	0.041031195
Arl5a	0.048609939	0.909847917	0.041031195

Slc25a34	0.078056685	0.909847917	0.041031195
Rfc2	0.187977085	0.909847917	0.041031195
Ttyh1	0.036717361	0.909847917	0.041031195
Rab3b	-0.073583223	0.909847917	0.041031195
Sntg2	0.06702374	0.909847917	0.041031195
Erlec1	0.042465573	0.909847917	0.041031195
Pdp2	0.051918477	0.909847917	0.041031195
Eme2	-0.04959352	0.909847917	0.041031195
Aff4	-0.089248676	0.909847917	0.041031195
Tmem203	0.033274817	0.909847917	0.041031195
Csmd2os	0.068128431	0.909847917	0.041031195
Slc25a16	-0.032628016	0.909847917	0.041031195
Lzts1	0.056355561	0.909847917	0.041031195
Hexdc	0.049880818	0.909847917	0.041031195
Slc5a4a	-0.07119791	0.909847917	0.041031195
FAM35A	-0.034912899	0.909847917	0.041031195
Coq3	-0.049318332	0.909847917	0.041031195
Nrbf2	0.024050187	0.909847917	0.041031195
Dennd1a	0.039068387	0.909847917	0.041031195
Rassf9	0.049611008	0.909847917	0.041031195
Slc19a1	0.033406995	0.909847917	0.041031195
Fam53b	-0.043603534	0.909847917	0.041031195
Zeb2	0.074993172	0.909847917	0.041031195
Cbll1	0.062549204	0.909847917	0.041031195
Ptpn2	0.031516078	0.909847917	0.041031195
Farsa	-0.029191925	0.909847917	0.041031195
Thg1l	0.058380816	0.909847917	0.041031195
Tdh	0.032744193	0.909847917	0.041031195
Fbxo25	0.043851123	0.909847917	0.041031195
Rit1	0.064359317	0.909847917	0.041031195
Swsap1	0.061751712	0.909847917	0.041031195
Inpp5k	0.029513981	0.909847917	0.041031195
Fam13c	0.069157596	0.909847917	0.041031195
Bet1l	-0.039892294	0.909847917	0.041031195
Dnah3	-0.040298739	0.909847917	0.041031195
Zfp236	-0.099547299	0.909847917	0.041031195
Klra7	0.070101008	0.909847917	0.041031195
Inafm1	-0.049475609	0.909847917	0.041031195
Hyal3	0.045621237	0.909847917	0.041031195
Slc7a15	-0.0232267	0.909847917	0.041031195
Gpr149	0.048461265	0.909847917	0.041031195
Otud6b	-0.057046341	0.909847917	0.041031195
Ube2cbp	-0.07411374	0.909847917	0.041031195
Acap2	-0.037852646	0.909847917	0.041031195
Map3k4	-0.037085846	0.909847917	0.041031195
Exoc8	-0.048624062	0.909847917	0.041031195
Klra3	-0.032869653	0.909847917	0.041031195
Cmtr2	-0.047218364	0.909847917	0.041031195

Srbd1	0.049950887	0.909847917	0.041031195
Klhl4	-0.027922965	0.909847917	0.041031195
COL4A3BF	0.032905547	0.909847917	0.041031195
HIST1H3H	0.043373932	0.909847917	0.041031195
Samd7	0.068956396	0.909847917	0.041031195
Ccdc169	0.083067668	0.909847917	0.041031195
Lrrc59	-0.065320879	0.909847917	0.041031195
Slc5a5	-0.021994613	0.909847917	0.041031195
Epha7	0.040754568	0.909847917	0.041031195
Cldn23	-0.029648315	0.909847917	0.041031195
Qpctl	0.063978544	0.909847917	0.041031195
Pdap1	0.069711103	0.909847917	0.041031195
Cep85	0.035636006	0.909847917	0.041031195
Wiz	0.052696187	0.909847917	0.041031195
BC006965	0.037990665	0.909847917	0.041031195
Tmsb15b1	0.035451139	0.909847917	0.041031195
Slf1	-0.040745467	0.909847917	0.041031195
Naa50	0.086521927	0.909847917	0.041031195
Brd7	-0.054903548	0.909847917	0.041031195
Pstpip2	0.070315648	0.909847917	0.041031195
Pex19	0.02202041	0.909847917	0.041031195
BC040756	0.021987792	0.909847917	0.041031195
Stx16	0.034584123	0.910038175	0.040940389
Ddc	-0.066098408	0.910038175	0.040940389
Borcs8	-0.036380494	0.910102866	0.040909518
Bnip1	0.034127054	0.910274751	0.040827503
Tmbim4	0.035188062	0.910360732	0.040786484
Uckl1	0.05056783	0.910381032	0.040776799
Rpap1	-0.107066463	0.910494231	0.040722802
Cd40lg	-0.051730586	0.910616861	0.040664313
Arfgap3	-0.061896259	0.910616861	0.040664313
Hsd17b2	-0.047018317	0.910633818	0.040656225
Hoxb9	0.06295653	0.910710505	0.040619654
Vps33b	-0.036319595	0.91082228	0.040566355
U2af1l4	-0.100744336	0.910823224	0.040565904
Sp8	0.046982881	0.910823224	0.040565904
Ube2h	0.052638076	0.910823224	0.040565904
Snip1	-0.043719887	0.910823224	0.040565904
Pigz	0.032333237	0.910823224	0.040565904
Zfp28	-0.035973746	0.910823224	0.040565904
Mageb3	0.073966879	0.910823224	0.040565904
Tmsb15b2	0.105481225	0.910823224	0.040565904
Pogz	0.045964689	0.910823224	0.040565904
Rhox4a2	-0.050363635	0.910823224	0.040565904
Mettl9	0.029177118	0.910823224	0.040565904
Npr2	0.171404529	0.910823224	0.040565904
Plekha1	-0.027299089	0.910823224	0.040565904
Sytl3	0.024425254	0.910823224	0.040565904

Setdb1	0.031669696	0.910823224	0.040565904
Rev3l	-0.044468813	0.910823224	0.040565904
Vmn1r3	0.021878281	0.910823224	0.040565904
Slc24a4	-0.067080746	0.911083456	0.04044184
Mrps31	-0.025746467	0.911232631	0.040370737
Flad1	0.047370122	0.911232631	0.040370737
Cfap58	-0.039723063	0.911240395	0.040367036
Pcdhga12	0.044771127	0.91129	0.040343395
P3h3	-0.045583473	0.91129	0.040343395
Sec63	-0.035998797	0.91129	0.040343395
BC017158	-0.074802292	0.911298043	0.040339562
Tsr1	-0.028785445	0.911298043	0.040339562
FAM45A	0.042966991	0.911303012	0.040337194
Zscan20	0.029023932	0.911303012	0.040337194
Samd11	-0.035402168	0.91161736	0.040187413
Lamtor3	-0.047049838	0.911752368	0.0401231
Slc35a1	0.033968417	0.911752368	0.0401231
PPP1R3FO	0.04519879	0.911752368	0.0401231
Pou2f3	-0.053433865	0.911752368	0.0401231
C7	-0.114428843	0.911752368	0.0401231
ZFP607	-0.072875779	0.911752368	0.0401231
Lipo4	0.036509854	0.911752368	0.0401231
Itch	-0.100121656	0.91186297	0.04007042
Cdc23	0.114174944	0.91186297	0.04007042
Naca	-0.088479503	0.91186297	0.04007042
Pkm	0.068454424	0.91186297	0.04007042
Olfr727	0.056493849	0.91186297	0.04007042
Llgl2	-0.074952373	0.91186297	0.04007042
Idh3g	-0.050543329	0.91186297	0.04007042
Bag2	0.045664713	0.91186297	0.04007042
Gpatch4	-0.055776122	0.91186297	0.04007042
Tk2	0.058321193	0.91186297	0.04007042
Olfr1437	-0.045352322	0.91186297	0.04007042
Spag1	-0.029701439	0.91186297	0.04007042
Kcnh6	-0.047101183	0.91186297	0.04007042
Tufm	0.089051774	0.91186297	0.04007042
Rsph3a	0.036101624	0.91186297	0.04007042
Pdf	-0.029772927	0.91186297	0.04007042
Tpp2	0.052177478	0.91186297	0.04007042
Poldip3	0.035725663	0.91186297	0.04007042
Pex11g	-0.059095493	0.911874214	0.040065065
Ccnk	0.068118309	0.911876615	0.040063922
ICT1	-0.085776265	0.911915846	0.040045238
Zfp330	0.075828121	0.911915846	0.040045238
Ndnf	0.027489766	0.911915846	0.040045238
Prh1	0.038018007	0.911915846	0.040045238
Nfatc2ip	0.027814052	0.911915846	0.040045238
Cfc1	0.10663371	0.911915846	0.040045238

Gstp1	-0.088015289	0.911915846	0.040045238
Zmym6	0.026956277	0.911915846	0.040045238
Ttc39a	-0.092920611	0.912109806	0.039952875
Pde3a	-0.071387697	0.912256233	0.039883161
Smchd1	0.054126545	0.912256233	0.039883161
Api5	0.041646058	0.912256233	0.039883161
Msantd4	0.04319633	0.912256233	0.039883161
Otud5	-0.05107706	0.912256233	0.039883161
Sult1c2	-0.057873543	0.912256233	0.039883161
Grwd1	-0.048293506	0.912256233	0.039883161
Zkscan17	0.080536221	0.912256233	0.039883161
Lonp2	-0.096737864	0.912256233	0.039883161
Ccdc182	0.05221132	0.912256233	0.039883161
Bpifb3	-0.051760521	0.912256233	0.039883161
Slc39a13	0.034430618	0.912256233	0.039883161
Ung	-0.027476423	0.912256233	0.039883161
Dnajc28	-0.048930213	0.912256233	0.039883161
Adal	-0.04660678	0.912390562	0.039819216
Pde4b	-0.075572476	0.912434578	0.039798265
Mup10	-0.035352114	0.912434578	0.039798265
Nsrp1	-0.083460669	0.912566615	0.039735423
Nono	0.110643438	0.912566615	0.039735423
Vgll3	-0.039956048	0.912566615	0.039735423
Rgl1	-0.023858826	0.912566615	0.039735423
Top3b	-0.087116932	0.912566615	0.039735423
Hnrnpr	-0.068012431	0.912566615	0.039735423
Uchl3	0.091521566	0.912566615	0.039735423
SDPR	0.143782373	0.912566615	0.039735423
Zfyve16	-0.029949033	0.912566615	0.039735423
Adi1	-0.028060319	0.912566615	0.039735423
Tulp1	-0.08245765	0.912566615	0.039735423
Klhl10	0.062899267	0.912566615	0.039735423
HDHD1A	0.044586067	0.912566615	0.039735423
Ift43	-0.045262337	0.912566615	0.039735423
Wapl	-0.028108669	0.912566615	0.039735423
Glrx	0.041028655	0.912566615	0.039735423
Olfr460	0.05661381	0.912566615	0.039735423
Myrfl	0.034314124	0.912566615	0.039735423
Zfp78	0.042849622	0.912566615	0.039735423
Rft1	0.086236715	0.912566615	0.039735423
Usp8	-0.036979639	0.912630269	0.039705131
Flcn	-0.06189836	0.912630269	0.039705131
Tmub1	0.033099213	0.912630269	0.039705131
Stt3b	-0.083904984	0.912630269	0.039705131
Xirp1	0.039403781	0.912630269	0.039705131
Man2c1	-0.046122815	0.912730928	0.039657233
Cnih4	-0.053308767	0.912730928	0.039657233
Zranb3	0.021418566	0.912730928	0.039657233

Pex11a	-0.028640747	0.912966942	0.039544948
Pnpla1	0.055395161	0.912966942	0.039544948
Nudt15	-0.099733169	0.912984358	0.039536663
Cdk8	0.351834357	0.912984358	0.039536663
Rgp1	-0.063576281	0.912984358	0.039536663
Frs3	-0.090235987	0.912984358	0.039536663
Gpm6b	-0.062062244	0.912984358	0.039536663
H1FX	-0.065436798	0.912984358	0.039536663
Fbxo43	-0.0696876	0.912984358	0.039536663
Cav2	-0.115227632	0.912984358	0.039536663
Cuta	0.045318989	0.912984358	0.039536663
Tax1bp1	0.028870491	0.912984358	0.039536663
Zfp493	-0.03203979	0.912984358	0.039536663
Tnni3k	-0.031411513	0.912984358	0.039536663
Zfp719	0.114227227	0.912984358	0.039536663
Kyat1	-0.047298006	0.912984358	0.039536663
Foxg1	0.064506233	0.912984358	0.039536663
Hic2	-0.056546215	0.912984358	0.039536663
Spock3	0.038050779	0.912984358	0.039536663
Mylk	0.114812964	0.912984358	0.039536663
Zzz3	0.041417187	0.912984358	0.039536663
Amy2a3	-0.106517499	0.912984358	0.039536663
Ctps	-0.058254962	0.912984358	0.039536663
Nctc1	-0.030366532	0.912984358	0.039536663
Rab5b	-0.044711707	0.912984358	0.039536663
Neurod2	0.053471824	0.912984358	0.039536663
Mysm1	-0.053274706	0.912984358	0.039536663
Agr3	-0.023786609	0.912984358	0.039536663
Ubxn6	-0.034040779	0.912984358	0.039536663
Orm3	0.076854633	0.912984358	0.039536663
Ggcx	0.051385137	0.912984358	0.039536663
Nol7	-0.030824977	0.912984358	0.039536663
Cox5b	-0.055885036	0.912984358	0.039536663
Slc7a8	0.021812419	0.912984358	0.039536663
Cnksr1	-0.035315029	0.912984358	0.039536663
Cwf19l1	0.05004855	0.912984358	0.039536663
Sema6c	0.024600694	0.912984358	0.039536663
Spns1	-0.06372445	0.913129937	0.039467418
Cnih1	0.03252763	0.913129937	0.039467418
Nrg3os	0.040486312	0.913129937	0.039467418
Copb1	0.021254685	0.913129937	0.039467418
Kpna3	-0.141395303	0.913242647	0.039413816
Rab2b	0.099117328	0.913242647	0.039413816
Fbxl13	-0.122308498	0.913242647	0.039413816
Kiss1	-0.046159548	0.913242647	0.039413816
Ifit1bl2	-0.063443133	0.91329094	0.039390851
Otx1	0.036553555	0.913340363	0.039367349
Mctp1	0.053696249	0.913450866	0.039314808

Mrpl19	0.032008484	0.913450866	0.039314808
Sprtn	0.127927576	0.913450866	0.039314808
Mccc2	0.068935438	0.913450866	0.039314808
ZCCHC11	0.045751828	0.913450866	0.039314808
Uqcrc1	0.035364912	0.913450866	0.039314808
Naip1	0.028622819	0.913450866	0.039314808
Hprt	0.024722849	0.913450866	0.039314808
Grxcr2	0.054349307	0.913450866	0.039314808
Kctd14	-0.032181093	0.913450866	0.039314808
Prelid2	0.057120423	0.913450866	0.039314808
Zcchc2	0.032652414	0.913450866	0.039314808
Crls1	0.030110665	0.913472374	0.039304583
Platr8	0.057355398	0.913521922	0.039281026
Plxnd1	-0.059916583	0.913521922	0.039281026
Nrxn2	-0.111849527	0.913642479	0.039223717
Hps6	-0.036516478	0.913694411	0.039199032
Ttll3	0.140802787	0.91371497	0.039189259
Hnrnpm	-0.057382114	0.913828196	0.039135446
Coa5	0.035084373	0.913828196	0.039135446
Methig1	0.071711242	0.913828196	0.039135446
Pigl	-0.07574522	0.913828196	0.039135446
Ccndbp1	0.047235851	0.913828196	0.039135446
Xrcc6	0.03044621	0.913828196	0.039135446
Fam149b	-0.04782109	0.913828196	0.039135446
Hsd3b3	-0.046992164	0.913828196	0.039135446
Pptc7	0.03425727	0.913828196	0.039135446
Txndc15	0.060369373	0.913828196	0.039135446
Npw	0.056429687	0.913828196	0.039135446
Slc25a15	0.021115072	0.913828196	0.039135446
Eef1d	-0.045672051	0.914061998	0.039024346
Gsc	0.064002099	0.914073542	0.039018862
Bbs7	0.048269855	0.914073542	0.039018862
Ash2l	0.040614362	0.914073542	0.039018862
Tdrd12	0.034337627	0.914073542	0.039018862
Serpinb7	-0.039116551	0.914073542	0.039018862
Rpgrip1	-0.045318699	0.914161778	0.038976941
Ptpn23	0.047067091	0.914161778	0.038976941
Tspan1	-0.061637759	0.914161778	0.038976941
Skint3	-0.096548766	0.914161778	0.038976941
Nr1i3	-0.095103807	0.914161778	0.038976941
Xpo6	0.035295565	0.914161778	0.038976941
Zbtb48	-0.053054914	0.914161778	0.038976941
Arsi	-0.075111006	0.914161778	0.038976941
Thop1	0.042934803	0.914161778	0.038976941
Nbr1	-0.096871945	0.91420293	0.038957391
Zfp787	-0.093778916	0.91420293	0.038957391
BC023829	-0.032894768	0.91420293	0.038957391
Kcne3	0.085592163	0.91420293	0.038957391

Pbld1	0.057891948	0.91420293	0.038957391
Stx3	-0.029133681	0.91420293	0.038957391
Mslnl	0.042760633	0.91420293	0.038957391
Akr1c19	0.057624109	0.91420293	0.038957391
Atp6v1g1	0.055393959	0.91420293	0.038957391
Snrpg	-0.039356161	0.91420293	0.038957391
Uchl1	-0.04867166	0.91420293	0.038957391
Ccdc47	-0.038985085	0.91420293	0.038957391
Clp1	-0.03740135	0.914312584	0.038905303
Uso1	0.051211441	0.914312584	0.038905303
Nat8f7	0.039785051	0.914312584	0.038905303
Ush1c	-0.039030623	0.914319979	0.03890179
Hsbp1	0.035633611	0.914319979	0.03890179
Aqp7	0.020955477	0.914445927	0.03884197
Luc7l3	-0.079034811	0.9145488	0.038793116
Angel1	-0.129491421	0.914575381	0.038780493
Tubgcp5	0.09466935	0.914575381	0.038780493
Olfr95	-0.161667472	0.914575381	0.038780493
Dcaf12	-0.074529468	0.914575381	0.038780493
Stmn4	-0.04552896	0.914575381	0.038780493
Kdr	-0.062603509	0.914575381	0.038780493
Gnpnat1	0.081306571	0.914575381	0.038780493
Tmem141	-0.12429047	0.914575381	0.038780493
Plk2	-0.070692011	0.914575381	0.038780493
Cidec	-0.040088722	0.914575381	0.038780493
Pcdhb18	0.103111523	0.914575381	0.038780493
Mex3b	0.037852397	0.914575381	0.038780493
HIST2H2AI	0.058750027	0.914575381	0.038780493
Pex16	-0.042116343	0.914575381	0.038780493
Fer1l6	0.046780791	0.914575381	0.038780493
Siah1b	-0.055874703	0.914575381	0.038780493
Ifitm10	-0.030677714	0.914575381	0.038780493
Naa38	-0.030466511	0.914575381	0.038780493
Fbxl20	0.040443606	0.914575381	0.038780493
Atg16l1	-0.05149255	0.914575381	0.038780493
Mmp28	-0.045912457	0.914575381	0.038780493
Krt73	-0.032100894	0.914575381	0.038780493
WBP5	-0.038893376	0.914575381	0.038780493
Dpy19l1	-0.072388246	0.914575381	0.038780493
Wdr3	0.028736449	0.914575381	0.038780493
Alg6	-0.044161478	0.914575381	0.038780493
Alg9	0.044107109	0.914575381	0.038780493
Dnah6	0.099241639	0.914575381	0.038780493
Csrp1	-0.030019919	0.914575381	0.038780493
Cnot6l	-0.057550833	0.914575381	0.038780493
Tigit	0.037938266	0.914575381	0.038780493
Ntan1	-0.034767291	0.914575381	0.038780493
Spesp1	0.07477414	0.914575381	0.038780493

Oplah	0.045447304	0.914575381	0.038780493
Gap43	-0.049976158	0.914575381	0.038780493
Olfr214	0.040476593	0.914575381	0.038780493
Akap8	0.053253833	0.914575381	0.038780493
Ivns1abp	0.029050735	0.914575381	0.038780493
Ctdsp2	-0.057518641	0.914575381	0.038780493
Gnb2	0.035928351	0.914575381	0.038780493
Taf11	-0.022116698	0.914575381	0.038780493
Hgd	-0.056713676	0.914575381	0.038780493
Samd4	-0.060907264	0.914575381	0.038780493
Rgs13	0.04750287	0.914575381	0.038780493
Adamts10	-0.02275192	0.914575381	0.038780493
Irak1bp1	0.044483499	0.914575381	0.038780493
Usp37	-0.025398615	0.914575381	0.038780493
Casp2	-0.03018908	0.914575381	0.038780493
Tespa1	0.020856154	0.914575381	0.038780493
Tfdp2	-0.072519344	0.914590998	0.038773077
Zfand6	-0.13342399	0.914590998	0.038773077
Tmem121	0.030167587	0.914590998	0.038773077
Mrpl4	0.038230126	0.9148302	0.038659507
Prp2	0.037968544	0.914841145	0.038654311
Canx	0.076561653	0.914846343	0.038651844
Fam187b	0.078268135	0.914900345	0.038626209
Ugt2b37	-0.040062974	0.915153191	0.038506202
Cdkl4	0.039429956	0.915153191	0.038506202
Ndor1	-0.030250762	0.915153191	0.038506202
Polq	-0.069119116	0.915153191	0.038506202
HIST1H2AI	-0.035813156	0.915153191	0.038506202
Vps11	-0.0856306	0.915153191	0.038506202
Utf1	-0.048609162	0.915153191	0.038506202
Rars	-0.061147734	0.915153191	0.038506202
Mettl14	-0.032081409	0.915153191	0.038506202
Fam110a	0.074073336	0.915153191	0.038506202
Trappc5	-0.045222042	0.915153191	0.038506202
Pdgfc	-0.066564826	0.915153191	0.038506202
Tmem251	0.066772863	0.915153191	0.038506202
Akt2	0.053916001	0.915153191	0.038506202
Olfr1033	-0.04798324	0.915153191	0.038506202
Rbm11	-0.051130849	0.915153191	0.038506202
Dbnl	-0.044902128	0.915153191	0.038506202
Gtpbp3	-0.078000607	0.915153191	0.038506202
Nfe2	0.031004968	0.915153191	0.038506202
Utrn	0.075105726	0.915153191	0.038506202
Frs2	-0.034992022	0.915153191	0.038506202
Tmem17	0.040303784	0.915153191	0.038506202
Them4	0.040328797	0.915153191	0.038506202
Nab1	-0.032759983	0.915153191	0.038506202
G6pc	0.035469847	0.915153191	0.038506202

MFI2	-0.028966604	0.915153191	0.038506202
Pebp1	-0.045779699	0.915153191	0.038506202
Hmgb3	0.020664578	0.915153191	0.038506202
ALS2CR12	0.154683784	0.915171008	0.038497747
MTL5	-0.039802288	0.915171008	0.038497747
Zfp810	0.063274759	0.915171008	0.038497747
Zfp426	-0.03221867	0.915171008	0.038497747
Fam222b	0.036579338	0.915171008	0.038497747
Plekhj1	0.031218077	0.915171008	0.038497747
Gtf2a2	0.042255557	0.915171008	0.038497747
Tsen2	-0.046752308	0.915171008	0.038497747
Vps36	0.083645632	0.915171008	0.038497747
Glipr1l3	0.045973536	0.915171008	0.038497747
Oxnad1	0.061563457	0.915171008	0.038497747
Olfr283	-0.0510343	0.915171008	0.038497747
Klhl42	0.043941262	0.915171008	0.038497747
Myot	-0.132791423	0.915548227	0.038318774
Zfp930	0.030857817	0.915587435	0.038300176
Arpc4	0.073830751	0.915689333	0.038251845
Psmc6	0.040956697	0.915689333	0.038251845
Nipal1	-0.080736039	0.915689858	0.038251596
Eif2b3	-0.081129788	0.915769055	0.038214036
Dcst2	0.036958156	0.915769055	0.038214036
Pds5a	0.088415776	0.915769055	0.038214036
Fam166a	0.05850864	0.915769055	0.038214036
Ifi44l	-0.025156569	0.915769055	0.038214036
Gsdmc4	-0.028660991	0.915834187	0.038183149
Sra1	-0.110009398	0.915908455	0.038147932
Dnmt1	-0.110996585	0.915908455	0.038147932
Myl12b	-0.114946402	0.915908455	0.038147932
Sf3b2	-0.115228129	0.915908455	0.038147932
Pacsin2	-0.087884067	0.915908455	0.038147932
Mvk	-0.06162581	0.915908455	0.038147932
Armcx6	-0.064952227	0.915908455	0.038147932
Rnf5	-0.074848915	0.915908455	0.038147932
Stk11ip	0.080259506	0.915908455	0.038147932
Brd1	0.069424537	0.915908455	0.038147932
Sorcs2	-0.055798495	0.915908455	0.038147932
Cep170	0.063726236	0.915908455	0.038147932
Spatc1	-0.044337187	0.915908455	0.038147932
Mtif3	0.103542354	0.915908455	0.038147932
Osbpl7	-0.038014439	0.915908455	0.038147932
Ubxn1	-0.044353319	0.915908455	0.038147932
FAM92A	0.060413482	0.915908455	0.038147932
FAM178A	-0.041809233	0.915908455	0.038147932
Golgb1	-0.040223346	0.915908455	0.038147932
Psmb4	-0.087215375	0.915908455	0.038147932
Hpf1	-0.096872818	0.915908455	0.038147932

Mirt2	-0.05613031	0.915908455	0.038147932
Ubb	0.047134454	0.915908455	0.038147932
FAM26E	-0.062173049	0.915908455	0.038147932
Tmem129	0.064561687	0.915908455	0.038147932
Atxn10	-0.072664185	0.915908455	0.038147932
Jak1	-0.037720442	0.915908455	0.038147932
Htra2	0.053246887	0.915908455	0.038147932
Pdk1	-0.068887416	0.915908455	0.038147932
Jagn1	0.092250992	0.915908455	0.038147932
Chpf	-0.044724816	0.915908455	0.038147932
Smco4	-0.064228768	0.915908455	0.038147932
Tmigd1	0.051501425	0.915908455	0.038147932
Smc5	0.063930814	0.915908455	0.038147932
Cldn2	0.035861702	0.915908455	0.038147932
Olfr985	0.042490289	0.915908455	0.038147932
Nim1k	-0.049189067	0.915908455	0.038147932
Zfp758	-0.026970927	0.915908455	0.038147932
Rfx7	0.040170457	0.915908455	0.038147932
Cldn34c3	0.043747903	0.915908455	0.038147932
Kansl2	-0.060969904	0.915908455	0.038147932
Mapkapk5	0.172779759	0.915908455	0.038147932
Med13	0.078960444	0.915908455	0.038147932
Vps26a	-0.048505308	0.915908455	0.038147932
Clec4b1	0.080592192	0.915908455	0.038147932
APOA1BP	-0.044208671	0.915908455	0.038147932
Prr12	0.0366781	0.915908455	0.038147932
Ddhd1	-0.031113093	0.915908455	0.038147932
Ccr1l1	0.058476214	0.915908455	0.038147932
Skint7	0.039992637	0.915908455	0.038147932
Spaca3	0.065721755	0.915908455	0.038147932
Zfp184	0.048563635	0.915908455	0.038147932
Psma5	-0.045306037	0.915908455	0.038147932
Edn2	-0.030657702	0.915908455	0.038147932
Vps4b	-0.047507374	0.915908455	0.038147932
FBXO18	-0.053958518	0.915908455	0.038147932
Smn1	0.211408642	0.915908455	0.038147932
Farsb	0.06069565	0.915908455	0.038147932
N6amt1	-0.040001156	0.915908455	0.038147932
Cdh15	0.058143412	0.915908455	0.038147932
Efcab8	0.041377892	0.915908455	0.038147932
Ftsj1	0.236033696	0.915908455	0.038147932
Rab2a	0.049056373	0.915908455	0.038147932
Cd226	0.073686511	0.915908455	0.038147932
Zdhhc3	0.103726681	0.915908455	0.038147932
Trmt44	0.044456157	0.915908455	0.038147932
Fastkd3	-0.035473936	0.915908455	0.038147932
Pfdn4	-0.060330431	0.915908455	0.038147932
Trim40	-0.029543712	0.915908455	0.038147932

Clk4	0.042287292	0.915908455	0.038147932
Srp19	0.096292986	0.915908455	0.038147932
Blmh	-0.044430405	0.915908455	0.038147932
Tox4	0.030735846	0.915908455	0.038147932
Nr4a2	0.042781395	0.915908455	0.038147932
Aldoart2	-0.077107932	0.915908455	0.038147932
Zfp804a	0.03225288	0.915908455	0.038147932
Trnau1ap	0.033298303	0.915908455	0.038147932
C1s2	0.054637099	0.915908455	0.038147932
FAM198B	-0.063934221	0.915908455	0.038147932
Scrt2	0.100939758	0.915908455	0.038147932
Msantd1	-0.027710451	0.915908455	0.038147932
Kcnk2	-0.032798084	0.915908455	0.038147932
Slc9a2	0.033116557	0.915908455	0.038147932
Olfr622	0.045535041	0.915908455	0.038147932
Cyp27b1	0.040402539	0.915908455	0.038147932
Prph2	0.041164391	0.915908455	0.038147932
Cabin1	0.05484166	0.915908455	0.038147932
Zfp800	-0.029244169	0.915908455	0.038147932
Vps37c	0.058368729	0.915908455	0.038147932
Nkx2-1	-0.03478938	0.915908455	0.038147932
Cyp11b1	-0.05176679	0.915908455	0.038147932
Nat3	0.066927005	0.915908455	0.038147932
Olfr513	0.04009894	0.915908455	0.038147932
Prelid1	-0.042602653	0.915908455	0.038147932
Hsf5	-0.046240397	0.915908455	0.038147932
Tbc1d10a	-0.036583441	0.915908455	0.038147932
Pgc	-0.038266697	0.915908455	0.038147932
Spink13	-0.050592693	0.915908455	0.038147932
Dnase1l3	-0.042397347	0.915908455	0.038147932
Zfp746	0.055414358	0.915908455	0.038147932
Marveld1	-0.073796933	0.915908455	0.038147932
Fam104a	0.157628891	0.915908455	0.038147932
Igsf5	0.061844163	0.915908455	0.038147932
Dusp27	-0.070001837	0.915908455	0.038147932
Gtf3c3	-0.116091912	0.915908455	0.038147932
Gpat2	0.064110569	0.915908455	0.038147932
Baiap2l1	-0.059081969	0.915908455	0.038147932
Sf3a2	-0.046202564	0.915908455	0.038147932
Pmpca	-0.044019192	0.915908455	0.038147932
Ctdp1	0.027176937	0.915908455	0.038147932
Dhx58os	-0.06943083	0.915908455	0.038147932
Npy6r	-0.07428429	0.915908455	0.038147932
Atp6v1e2	0.048743336	0.915908455	0.038147932
Dclk3	0.061670783	0.915908455	0.038147932
N4bp2l2	-0.070610016	0.915908455	0.038147932
Casq1	0.051369063	0.915908455	0.038147932
Slmap	0.051745643	0.915908455	0.038147932

Tnmd	0.187548978	0.915908455	0.038147932
Zp1	-0.036542289	0.915908455	0.038147932
Cyp2j9	-0.03451714	0.915908455	0.038147932
Clnk	0.036100513	0.915908455	0.038147932
Zc3h14	-0.039040487	0.915908455	0.038147932
Tcf23	-0.140810792	0.915908455	0.038147932
Necab2	0.042238933	0.915908455	0.038147932
Prss12	0.050227782	0.915908455	0.038147932
Vasn	-0.070054648	0.915908455	0.038147932
Ints2	0.065511595	0.915908455	0.038147932
Zpld1	0.039409735	0.915908455	0.038147932
Atp11c	0.072351838	0.915908455	0.038147932
Pabpn1l	-0.040390255	0.915908455	0.038147932
Foxk1	-0.042117631	0.915908455	0.038147932
Pla2g2f	0.040079209	0.915908455	0.038147932
Rybp	0.032421815	0.915908455	0.038147932
Slc18b1	-0.072929246	0.915908455	0.038147932
Ankrd23	0.048469837	0.915908455	0.038147932
Rab30	-0.038907498	0.915908455	0.038147932
Pop7	0.031601882	0.915908455	0.038147932
Med6	-0.034052627	0.915908455	0.038147932
Zfp383	0.040410195	0.915908455	0.038147932
Smco1	0.061154922	0.915908455	0.038147932
SGOL2B	0.067966921	0.915908455	0.038147932
Igsf3	-0.067803349	0.915908455	0.038147932
Piga	0.03659822	0.915908455	0.038147932
Fktn	0.034453486	0.915908455	0.038147932
WDR60	0.097161184	0.915908455	0.038147932
Gipc2	-0.037453878	0.915908455	0.038147932
Cnot8	-0.066960819	0.915908455	0.038147932
Nfrkb	-0.049457622	0.915908455	0.038147932
Spata4	-0.026098985	0.915908455	0.038147932
Ihh	-0.081012643	0.915908455	0.038147932
Spdef	-0.029201513	0.915908455	0.038147932
Ppm1n	-0.034096124	0.915908455	0.038147932
Snd1	-0.133379655	0.915908455	0.038147932
Gspt2	0.025733902	0.915908455	0.038147932
Wbp4	0.064243837	0.915908455	0.038147932
U2af1	-0.032104342	0.915908455	0.038147932
Ttc36	0.06927377	0.915908455	0.038147932
Zw10	-0.045569582	0.915908455	0.038147932
Blcap	-0.034894175	0.915908455	0.038147932
Olfr114	0.067237996	0.915908455	0.038147932
Lmod3	0.026330259	0.915908455	0.038147932
Mtr	0.048140055	0.915908455	0.038147932
Mrgprb5	0.048380524	0.915908455	0.038147932
Arl3	-0.048170089	0.915908455	0.038147932
Thap2	0.068043684	0.915908455	0.038147932

Dyrk1b	0.029965326	0.915908455	0.038147932
Wnt8a	-0.047440239	0.915908455	0.038147932
Usp50	-0.042818362	0.915908455	0.038147932
Rprml	0.066102049	0.915908455	0.038147932
Rpl31	0.033244649	0.915908455	0.038147932
Scel	0.032224544	0.915908455	0.038147932
Dcun1d1	-0.078566918	0.915908455	0.038147932
MAATS1	-0.038595631	0.915908455	0.038147932
Hnrnpu	0.023452227	0.915908455	0.038147932
Ggct	-0.050306893	0.915908455	0.038147932
Lrch3	0.098492496	0.915908455	0.038147932
Il17rd	-0.036087994	0.915908455	0.038147932
Gatad2b	-0.027667241	0.915908455	0.038147932
Dnali1	0.044882069	0.915908455	0.038147932
BC016548	0.026889026	0.915908455	0.038147932
Scoc	0.03741213	0.915908455	0.038147932
Hmces	0.040945459	0.915908455	0.038147932
Krt1	0.034473337	0.915908455	0.038147932
Pcolce2	-0.040664578	0.915908455	0.038147932
Brs3	-0.039406137	0.915908455	0.038147932
Gpr180	0.041906947	0.915908455	0.038147932
Trim11	0.040758929	0.915908455	0.038147932
Tmem259	-0.034111385	0.915908455	0.038147932
Atf7ip	-0.043139533	0.915908455	0.038147932
Arpp21	0.074009865	0.915908455	0.038147932
Rnf217	0.059524388	0.915908455	0.038147932
Agmat	0.031385824	0.915908455	0.038147932
Cdh20	-0.029627054	0.915908455	0.038147932
Gorab	0.069415856	0.915908455	0.038147932
Ydjc	-0.032534995	0.915908455	0.038147932
Atf7ip2	-0.037344277	0.915908455	0.038147932
DPM1-ADN	0.050402246	0.915908455	0.038147932
Ahdc1	0.049396168	0.915908455	0.038147932
Tcam1	0.042826463	0.915908455	0.038147932
Arl10	0.088455924	0.915908455	0.038147932
BC039771	-0.04770278	0.915908455	0.038147932
Fbxw27	0.046008671	0.915908455	0.038147932
Setd2	-0.036682961	0.915908455	0.038147932
Tpm1	0.031394552	0.915908455	0.038147932
Ccdc180	0.031636268	0.915908455	0.038147932
Ranbp9	0.021779875	0.915908455	0.038147932
Zc3h12c	0.035608602	0.915908455	0.038147932
Utp15	-0.023048755	0.915908455	0.038147932
Rnaseh2c	-0.046406448	0.915908455	0.038147932
Zbtb8b	0.04607518	0.915908455	0.038147932
Cox7a2l	0.034811154	0.915908455	0.038147932
Bola1	0.041969044	0.915908455	0.038147932
Slc2a8	0.059968044	0.915908455	0.038147932

Cacna1s	0.033602667	0.915908455	0.038147932
Olfr178	0.06167956	0.915908455	0.038147932
Poglut1	-0.050518049	0.915908455	0.038147932
Drd3	-0.041235428	0.915908455	0.038147932
Zc3h10	-0.019976047	0.915908455	0.038147932
Sh3rf1	-0.019971674	0.915908455	0.038147932
Chtop	0.028463665	0.915908455	0.038147932
Muc13	-0.051644933	0.915908455	0.038147932
Mier3	-0.048064224	0.915908455	0.038147932
Suco	0.032566532	0.915908455	0.038147932
Ecsit	0.027621461	0.915908455	0.038147932
Actr1a	0.064444884	0.915908455	0.038147932
Agbl1	0.085521706	0.915908455	0.038147932
Ankrd33	-0.053956535	0.915908455	0.038147932
Xpot	0.023847037	0.915908455	0.038147932
Uqcr11	0.042905886	0.915908455	0.038147932
Rab1b	0.066116802	0.915908455	0.038147932
Ggnbp1	-0.025878669	0.915908455	0.038147932
Grm5	0.021795159	0.915908455	0.038147932
Insm2	-0.063640946	0.915908455	0.038147932
Nck2	0.056656346	0.915908455	0.038147932
Lats1	-0.043356707	0.915908455	0.038147932
Lsm6	0.067859828	0.915908455	0.038147932
Olfr654	0.063403863	0.915908455	0.038147932
Trip12	0.029866064	0.915908455	0.038147932
Nifk	-0.03827855	0.915908455	0.038147932
Mcpt1	-0.033683004	0.915908455	0.038147932
Pip5kl1	0.03666203	0.915908455	0.038147932
Il1r2	-0.034729609	0.915908455	0.038147932
Smc1a	-0.032467628	0.915908455	0.038147932
Fgf8	0.033679793	0.915908455	0.038147932
Plekhb1	0.019966535	0.915908455	0.038147932
Slc16a12	0.0199304	0.915908455	0.038147932
Zswim7	0.017581403	0.915908455	0.038147932
Podxl	0.082934206	0.915935895	0.038134921
Stx12	0.041490646	0.915935895	0.038134921
Phf2	-0.053251878	0.915935895	0.038134921
Zpbp2	0.031752202	0.915935895	0.038134921
Sult5a1	-0.086401475	0.915935895	0.038134921
Tmem199	0.031554436	0.915935895	0.038134921
Dennd2a	0.038748981	0.915935895	0.038134921
Rpl4	-0.034909426	0.915935895	0.038134921
Zfp623	0.03056605	0.915935895	0.038134921
Sugp1	0.03683495	0.915935895	0.038134921
Yme1l1	-0.035139301	0.915935895	0.038134921
BC020402	0.022458626	0.915935895	0.038134921
Cd300c	-0.033926344	0.915935895	0.038134921
Sorbs2os	0.035058285	0.915935895	0.038134921

Hs6st1	0.029838234	0.915935895	0.038134921
Abca3	0.044208186	0.915935895	0.038134921
Dusp19	0.02763113	0.915935895	0.038134921
Stk19	-0.03638251	0.915935895	0.038134921
Baz2a	-0.033169589	0.915935895	0.038134921
Tsga10ip	-0.067599778	0.916069029	0.0380718
Rnf40	0.069863343	0.916069029	0.0380718
Adgra3	-0.128821	0.916069029	0.0380718
Krt42	-0.065819535	0.916069029	0.0380718
BC02413	-0.080547722	0.916069029	0.0380718
Ak6	0.043607742	0.916069029	0.0380718
Agpat4	0.047013374	0.916069029	0.0380718
Nabp2	-0.030492104	0.916069029	0.0380718
Cdk13	0.029076482	0.916069029	0.0380718
Cel	0.097643034	0.916069029	0.0380718
Dthd1	-0.069657108	0.916069029	0.0380718
B4galnt2	-0.026640542	0.916069029	0.0380718
Fkbp2	-0.06666406	0.916069029	0.0380718
Slc30a6	-0.065283817	0.916069029	0.0380718
Fam118a	-0.05032857	0.916069029	0.0380718
Sppl3	0.032642353	0.916069029	0.0380718
Rnase13	-0.05468133	0.916069029	0.0380718
Inhbc	0.035194094	0.916069029	0.0380718
Pde6a	-0.029895647	0.916069029	0.0380718
Gnpat	0.049476278	0.916069029	0.0380718
Retsat	0.031826914	0.916069029	0.0380718
Zfp85	0.032482397	0.916069029	0.0380718
Exosc8	-0.05481063	0.916069029	0.0380718
Osbpl2	0.045239834	0.916069029	0.0380718
Adamts5	0.081566047	0.916069029	0.0380718
Preb	0.082191149	0.916113546	0.038050695
Slc6a3	0.188115984	0.916113546	0.038050695
Srsf11	-0.054035224	0.916113546	0.038050695
Rimklb	0.036062636	0.916113546	0.038050695
Mfsd9	-0.033219947	0.916113546	0.038050695
Atp9b	-0.047693936	0.916137329	0.038039421
Brf2	-0.042509059	0.916137329	0.038039421
Copb2	0.035998303	0.916137329	0.038039421
Crispld1	-0.028149753	0.916137329	0.038039421
Rho	-0.029743281	0.916137329	0.038039421
Styxl1	0.145170098	0.916202049	0.038008741
Secisbp2l	-0.104068331	0.916202049	0.038008741
Gcc1	-0.136243024	0.916202049	0.038008741
Ccdc130	-0.115883627	0.916202049	0.038008741
Ufm1	-0.05410724	0.916202049	0.038008741
Phb2	0.069074595	0.916202049	0.038008741
Dynlrb1	0.12678915	0.916202049	0.038008741
Lyrm9	-0.073664529	0.916202049	0.038008741

Epgn	-0.086379233	0.916202049	0.038008741
Tada2b	0.082917762	0.916202049	0.038008741
Bmp15	-0.088988577	0.916202049	0.038008741
Scyl3	-0.055979147	0.916202049	0.038008741
ISRAA	0.148062683	0.916202049	0.038008741
Tusc1	0.07120716	0.916202049	0.038008741
Acrbp	-0.08506605	0.916202049	0.038008741
Atg101	0.041897421	0.916202049	0.038008741
Pcdhb7	0.064381743	0.916202049	0.038008741
Xist	0.040726029	0.916202049	0.038008741
Pex10	0.162983348	0.916202049	0.038008741
Rab12	-0.092704309	0.916202049	0.038008741
Mfsd14b	0.074859085	0.916202049	0.038008741
Ppara	0.039970355	0.916202049	0.038008741
Mrpl36	0.032145709	0.916202049	0.038008741
Rtl1	-0.029330388	0.916202049	0.038008741
Arhgap20c	-0.051215071	0.916202049	0.038008741
Trim65	-0.048328466	0.916202049	0.038008741
Rps14	0.048625967	0.916202049	0.038008741
Tubb4a	-0.05619272	0.916202049	0.038008741
Ahsg	0.029152648	0.916202049	0.038008741
Foxp2	-0.063192652	0.916202049	0.038008741
Themis	0.030980124	0.916202049	0.038008741
Rrn3	0.053948256	0.916202049	0.038008741
Cd209c	0.060857198	0.916202049	0.038008741
Fam83g	0.041834173	0.916202049	0.038008741
Aldh6a1	0.072310735	0.916202049	0.038008741
BC018473	-0.036305177	0.916202049	0.038008741
Arpc1a	-0.065637555	0.916202049	0.038008741
Ube2s	-0.035544353	0.916202049	0.038008741
7-Sep	-0.058654569	0.916202049	0.038008741
Pbrm1	0.090636251	0.916202049	0.038008741
Fkbpl	0.097223589	0.916202049	0.038008741
Vps52	-0.046281758	0.916202049	0.038008741
Srgap1	0.037274166	0.916202049	0.038008741
Eif4e2	0.058205703	0.916202049	0.038008741
Dcbld1	0.057789071	0.916202049	0.038008741
Sdhaf4	-0.032443883	0.916202049	0.038008741
Gnpda2	0.046224445	0.916202049	0.038008741
Reep6	-0.022399874	0.916202049	0.038008741
Vmo1	-0.049588648	0.916202049	0.038008741
Fbxo21	0.068949615	0.916202049	0.038008741
TMEM55B	-0.032571061	0.916202049	0.038008741
Usf1	-0.052279549	0.916202049	0.038008741
Nkx2-4	0.030508938	0.916202049	0.038008741
Trip10	0.043222188	0.916202049	0.038008741
Slc4a1	0.041895687	0.916202049	0.038008741
Sbf1	-0.025765103	0.916202049	0.038008741

Hdac8	0.04606077	0.916202049	0.038008741
Zfp558	0.042599673	0.916202049	0.038008741
Il1bos	-0.043820568	0.916202049	0.038008741
Mta1	-0.067451899	0.916202049	0.038008741
Ddx56	-0.021978154	0.916202049	0.038008741
Cox6b1	0.028439649	0.916202049	0.038008741
Aar2	0.112863145	0.916202049	0.038008741
Olfr209	0.047515361	0.916202049	0.038008741
Gimap8	0.054001719	0.916202049	0.038008741
Xrcc1	0.029334625	0.916202049	0.038008741
Xpc	0.055837022	0.916202049	0.038008741
Skida1	0.023322969	0.916202049	0.038008741
Tnrc6c	-0.031461472	0.916202049	0.038008741
Rrp1b	-0.026178662	0.916202049	0.038008741
Tmigd3	0.029882904	0.916202049	0.038008741
Taar1	-0.084247169	0.916202049	0.038008741
Tnpo3	-0.063981821	0.916202049	0.038008741
P2rx3	-0.037280296	0.916202049	0.038008741
Mab21l2	-0.082256106	0.916202049	0.038008741
Cdrt4os2	0.039021113	0.916202049	0.038008741
Zbtb1	-0.081744118	0.916202049	0.038008741
Tiam1	-0.045049105	0.916202049	0.038008741
Ctu1	-0.021581062	0.916202049	0.038008741
Mboat4	0.023451304	0.916202049	0.038008741
Aldoc	0.047384607	0.916202049	0.038008741
Ccny	-0.033973748	0.916202049	0.038008741
Sema4g	0.039602169	0.916202049	0.038008741
Ccdc116	-0.023681471	0.916202049	0.038008741
Ddx20	0.020317383	0.916202049	0.038008741
Def8	-0.028771846	0.916202049	0.038008741
Ino80	0.048337557	0.916202049	0.038008741
Trpm1	-0.031117065	0.916202049	0.038008741
Eif2d	-0.049431844	0.916202049	0.038008741
Cep120	-0.073125311	0.916202049	0.038008741
Xrcc5	-0.0548525	0.916202049	0.038008741
Foxf1	-0.019448391	0.916202049	0.038008741
Gse1	-0.019251941	0.916202049	0.038008741
Slc25a38	0.019899351	0.916202049	0.038008741
Synm	-0.05932459	0.916202049	0.038008741
Hhatl	0.027119603	0.916202049	0.038008741
Brd4	0.046557946	0.916202049	0.038008741
Dnah12	-0.04308248	0.91632177	0.037951995
Fbrs	-0.091563382	0.916451938	0.037890306
Mfsd4b1	0.07438416	0.916451938	0.037890306
Usp6nl	-0.041702139	0.916451938	0.037890306
Msln	0.039194616	0.916451938	0.037890306
Pik3c2g	-0.040498163	0.916451938	0.037890306
Ofd1	0.093964703	0.916451938	0.037890306

Col22a1	-0.041804435	0.916451938	0.037890306
Kirrel3	0.061887037	0.916451938	0.037890306
Gadd45gip	0.051137111	0.916634196	0.037803945
Spink2	0.050374863	0.916695625	0.037774841
Tubgcp3	0.06172871	0.916695625	0.037774841
Slc22a17	-0.052503831	0.916695625	0.037774841
Sf3a1	0.036856865	0.916695625	0.037774841
Rwdd4a	-0.033906333	0.916695625	0.037774841
Nwd1	-0.043474698	0.916695625	0.037774841
Mtch1	0.131574614	0.916695625	0.037774841
Bloc1s6	-0.028728425	0.916695625	0.037774841
Flg	-0.069077026	0.916695625	0.037774841
Synrg	0.051588456	0.916695625	0.037774841
Spon1	0.035208049	0.916695625	0.037774841
Enoph1	0.032139242	0.916695625	0.037774841
Ncr1	-0.040063658	0.916695625	0.037774841
Ptprt	0.036002602	0.916695625	0.037774841
Cd27	0.029560248	0.916695625	0.037774841
Stub1	0.041863081	0.916695625	0.037774841
Irs3	-0.053367242	0.916695625	0.037774841
Cpa5	0.031390345	0.916695625	0.037774841
Zc3h7a	-0.046975662	0.916695625	0.037774841
Myoz2	-0.038887364	0.916695625	0.037774841
Smc6	-0.069678524	0.916695625	0.037774841
St13	-0.094148711	0.916695625	0.037774841
Olfr1419	0.064706954	0.916695625	0.037774841
Zfp950	-0.023115376	0.916695625	0.037774841
Cant1	-0.119908254	0.91675023	0.037748972
B4galt4	-0.019038174	0.91675023	0.037748972
Paip2	-0.058956669	0.916937156	0.037660429
Tlx2	-0.040903119	0.916937156	0.037660429
Sirt1	0.085062795	0.917055687	0.037604292
Efcab1	0.069720546	0.917055687	0.037604292
Ceacam16	0.038338538	0.917055687	0.037604292
Mrpl14	0.055497079	0.917055687	0.037604292
Ilf3	0.043634746	0.917055687	0.037604292
Krt28	-0.043474908	0.917055687	0.037604292
Parg	-0.036306245	0.917055687	0.037604292
Slc25a51	0.055837894	0.917055687	0.037604292
Fmo4	0.053071568	0.917055687	0.037604292
Mettl7b	0.113161256	0.917055687	0.037604292
Sap18	0.044775356	0.917055687	0.037604292
Gata2	-0.040250976	0.917055687	0.037604292
Ing4	0.039675776	0.917055687	0.037604292
Chmp4c	0.060233876	0.917055687	0.037604292
Fxyd3	0.045563906	0.917055687	0.037604292
Acot9	-0.052580013	0.917055687	0.037604292
Dnajc13	-0.032428531	0.917055687	0.037604292

Tbcb	-0.084077944	0.917055687	0.037604292
Drg1	-0.033985447	0.917055687	0.037604292
Gmppa	0.029582143	0.917055687	0.037604292
Alkbh5	-0.038388411	0.917055687	0.037604292
Pou5f1	0.020006583	0.917055687	0.037604292
Lrrn4cl	0.050002107	0.917055687	0.037604292
Pla2g2e	-0.026827802	0.917055687	0.037604292
Dpf3	-0.035294596	0.917055687	0.037604292
Actn4	-0.040500197	0.917055687	0.037604292
Acot4	0.043805329	0.917055687	0.037604292
Trim58	0.033783953	0.917055687	0.037604292
Bsnd	0.04119628	0.917055687	0.037604292
Map3k7cl	-0.056608494	0.917324167	0.037477165
Ppp1r15a	0.030046833	0.917357005	0.037461618
Atp5l	0.039503867	0.917357005	0.037461618
Med1	-0.043905107	0.917371172	0.037454912
Cxcr5	-0.04272027	0.917400934	0.037440822
Tubb5	-0.032175372	0.917400934	0.037440822
Slc22a16	-0.049719582	0.917400934	0.037440822
Krt27	0.036115644	0.917400934	0.037440822
Golph3l	-0.045243839	0.917400934	0.037440822
Pax3	0.074436736	0.917400934	0.037440822
Srsf5	-0.111481329	0.917400934	0.037440822
Gtf3c5	-0.103350941	0.917400934	0.037440822
Wdpcp	-0.031136301	0.917400934	0.037440822
TCTEX1D4	0.028085853	0.917400934	0.037440822
Zfand3	0.0183678	0.917400934	0.037440822
Tdp1	-0.057243234	0.917455717	0.037414889
Rps21	0.062180291	0.917653471	0.037321288
Ndufv3	-0.030713588	0.917653471	0.037321288
E4f1	-0.02022986	0.917653471	0.037321288
Lrrc8e	-0.026111886	0.917653471	0.037321288
Ndufaf7	0.055325797	0.917653471	0.037321288
Baiap2l2	0.077339712	0.917878112	0.037214986
Arid2	-0.068218116	0.917878112	0.037214986
Wdr91	-0.046561509	0.91809754	0.037111177
AI504432	0.0358831	0.91809754	0.037111177
Ptcd3	0.068947463	0.91809754	0.037111177
Chn1os3	-0.035023229	0.918112426	0.037104135
Banf1	-0.115851292	0.918263735	0.037032567
Sars2	-0.093112036	0.918263735	0.037032567
Eid2b	0.053254063	0.918263735	0.037032567
Smpd3	-0.066558476	0.918263735	0.037032567
Olfr986	-0.045525977	0.918263735	0.037032567
Lypd1	0.044766195	0.918263735	0.037032567
Tex10	0.037284408	0.918263735	0.037032567
Kbtbd13	-0.067987874	0.918263735	0.037032567
Clpx	0.062879212	0.918263735	0.037032567

Tmed7	-0.042339682	0.918263735	0.037032567
Pcdhga11	0.034585014	0.918263735	0.037032567
Ywhaz	-0.056793041	0.918263735	0.037032567
Trip11	0.03365214	0.918263735	0.037032567
Cdkn2d	-0.035675685	0.918263735	0.037032567
Fmo6	-0.071788678	0.918263735	0.037032567
Coro1a	0.080703562	0.918263735	0.037032567
Polr3k	0.050362018	0.918263735	0.037032567
Wrap53	0.030947932	0.918263735	0.037032567
Plekhn1	0.024398461	0.918263735	0.037032567
Hnrnpa2b1	-0.042957102	0.918263735	0.037032567
Txn2	-0.049220233	0.918263735	0.037032567
Rxfp4	-0.041941908	0.918263735	0.037032567
Zfp428	0.020351147	0.918263735	0.037032567
Slc30a8	-0.021289965	0.918263735	0.037032567
Cdsn	-0.027156564	0.918263735	0.037032567
Bank1	0.115335598	0.918263735	0.037032567
Mlf2	0.021701489	0.918263735	0.037032567
Pdc	0.055150534	0.918386025	0.036974734
Dedd	-0.035351776	0.918416709	0.036960223
Prkag1	0.114108582	0.91845189	0.036943588
Plekha8	-0.046211115	0.918804902	0.036776696
Smcr8	-0.128535651	0.918819314	0.036769884
Dytn	-0.063354034	0.918819314	0.036769884
BC048644	0.074870747	0.918819314	0.036769884
Pcyox1	-0.037056378	0.918819314	0.036769884
Brd8	0.020557578	0.918819314	0.036769884
Zbtb8os	0.080521091	0.918819314	0.036769884
D6Ertd527	-0.030964663	0.918819314	0.036769884
Tyw1	0.047501851	0.918819314	0.036769884
Dhx38	-0.062944983	0.918819314	0.036769884
FAM65B	-0.023282724	0.918819314	0.036769884
Fbf1	0.032712405	0.918819314	0.036769884
Ctrc	-0.040377208	0.918819314	0.036769884
Tldc2	-0.060048797	0.918819314	0.036769884
Nfkbib	0.027721352	0.918819314	0.036769884
Hs3st5	0.074788192	0.91886581	0.036747908
HIST1H2AI	0.033912323	0.918964028	0.036701488
Mgarp	-0.034596308	0.918964028	0.036701488
Paip1	-0.020313589	0.918964028	0.036701488
G3bp1	-0.052142785	0.91899217	0.036688189
Mrpl34	-0.074848797	0.919053944	0.036658997
Ythdf2	-0.081246828	0.919053944	0.036658997
HIST1H2BI	0.06508428	0.919053944	0.036658997
Pkd1	-0.0372444	0.919053944	0.036658997
Il6	0.034939321	0.919053944	0.036658997
Fbxo6	0.065465957	0.919053944	0.036658997
Ccdc103	-0.089432924	0.919053944	0.036658997

Crb3	-0.037157729	0.919053944	0.036658997
Bloc1s3	-0.071544878	0.919053944	0.036658997
Scmh1	-0.10140637	0.919053944	0.036658997
Numa1	-0.036449542	0.919053944	0.036658997
Znhit2	0.032720049	0.919053944	0.036658997
Psmc3	-0.048821317	0.919053944	0.036658997
Anxa10	0.066995747	0.919053944	0.036658997
Plekha5	-0.044149373	0.919135433	0.036620491
Amy1	-0.099501145	0.919135433	0.036620491
Adck5	-0.182638826	0.919135433	0.036620491
Cdan1	0.038009761	0.919135433	0.036620491
Atp13a1	-0.047266301	0.919135433	0.036620491
Isyna1	0.070012185	0.919164096	0.036606948
Cdkal1	-0.099733287	0.919287725	0.036548539
Tssk2	-0.083903379	0.919287725	0.036548539
Ubn2	0.062924264	0.919287725	0.036548539
Rdh12	0.195616482	0.919287725	0.036548539
Faf1	-0.114227308	0.919287725	0.036548539
Clec11a	-0.086848227	0.919287725	0.036548539
Fsbp	0.06433259	0.919287725	0.036548539
Pibf1	-0.034897258	0.919287725	0.036548539
Olfr5	0.048535619	0.919287725	0.036548539
Nhej1	0.052368986	0.919287725	0.036548539
QTRTD1	-0.0496199	0.919287725	0.036548539
Ttc39d	0.067528027	0.919287725	0.036548539
Adprh	0.041326902	0.919287725	0.036548539
Cldn13	0.029652528	0.919287725	0.036548539
Sbk3	0.025425061	0.919287725	0.036548539
Tmem201	0.034204482	0.919287725	0.036548539
Cr2	-0.07314827	0.919287725	0.036548539
Zfp202	0.029413341	0.919287725	0.036548539
Mup16	-0.101223394	0.919287725	0.036548539
Ccng2	-0.132680468	0.919287725	0.036548539
Mrgprb2	-0.033991565	0.919287725	0.036548539
Nt5m	0.034180831	0.919287725	0.036548539
Zfp783	-0.050380455	0.919287725	0.036548539
H2-Q10	-0.085687	0.919287725	0.036548539
Zfp619	-0.030276733	0.919287725	0.036548539
Krtap10-1(	-0.041607576	0.919287725	0.036548539
Nr1h2	0.030218449	0.919287725	0.036548539
Kif21b	0.036103468	0.919287725	0.036548539
Gpr155	0.064512343	0.919287725	0.036548539
Ccdc61	0.038917157	0.919287725	0.036548539
Pex3	0.02800567	0.919287725	0.036548539
Lama3	0.074057447	0.919287725	0.036548539
Rassf7	-0.033777284	0.919287725	0.036548539
Crk	0.067348929	0.919287725	0.036548539
Mep1a	0.040186513	0.919287725	0.036548539

Morn5	0.054662613	0.919287725	0.036548539
Rsph3b	0.03548338	0.919287725	0.036548539
Sh3kbp1	-0.027595733	0.919287725	0.036548539
Bloc1s4	0.036071059	0.919287725	0.036548539
Gpatch8	-0.045743394	0.919287725	0.036548539
Rimkla	-0.041206589	0.919287725	0.036548539
Rab18	0.035075672	0.919287725	0.036548539
Atp6v0b	0.031082133	0.919287725	0.036548539
Wdr20	-0.040312429	0.919287725	0.036548539
Cep250	0.06467922	0.919287725	0.036548539
Sumo1	0.054895366	0.919287725	0.036548539
Lrfn4	-0.029831585	0.919287725	0.036548539
Mrpl17	0.028110181	0.919287725	0.036548539
Zglp1	0.058721609	0.919287725	0.036548539
Nanog	-0.034530214	0.919287725	0.036548539
Isx	0.072431117	0.919287725	0.036548539
Ercc6l2	0.061278401	0.919287725	0.036548539
FAM69A	-0.034301406	0.919287725	0.036548539
Nedd8	0.036651674	0.919287725	0.036548539
Ipo13	0.032982625	0.919287725	0.036548539
Psip1	-0.048506185	0.919287725	0.036548539
Trmt10c	0.058137006	0.919287725	0.036548539
Syce1	-0.061853601	0.919287725	0.036548539
Nadk	0.030050778	0.919287725	0.036548539
Ireb2	0.046274189	0.919378896	0.036505469
Irf6	-0.02872859	0.919378896	0.036505469
Ikzf5	-0.034915633	0.919378896	0.036505469
Mlh1	0.039156901	0.919378896	0.036505469
Fam117b	-0.023594421	0.919378896	0.036505469
Plaa	0.028975277	0.919378896	0.036505469
Rbm44	-0.052557177	0.919392598	0.036498997
Cbwd1	-0.082551432	0.919392598	0.036498997
Tmem70	0.043916476	0.919392598	0.036498997
Dnah7b	0.026911128	0.919392598	0.036498997
Ppp2r2d	0.025264936	0.919392598	0.036498997
FAM213A	0.03736662	0.919509576	0.036443743
MTSS1L	0.033272198	0.919509576	0.036443743
AU01583€	0.037707192	0.919509576	0.036443743
Olfr652	-0.079408035	0.919543334	0.036427799
Vmn2r20	0.060192134	0.919543334	0.036427799
Vmn2r21	-0.076707783	0.919543334	0.036427799
Vmn2r22	-0.042444122	0.919543334	0.036427799
AMICA1	-0.027961466	0.919543334	0.036427799
Olfr874	0.061135486	0.919543334	0.036427799
Pcif1	0.025237742	0.919543334	0.036427799
Bcl7c	0.058708154	0.919543334	0.036427799
Usp24	-0.046080644	0.919543334	0.036427799
Dcaf17	0.03019756	0.919543334	0.036427799

C1rb	0.048008643	0.919543334	0.036427799
Camk1	-0.059762041	0.919543334	0.036427799
Serpina10	0.037205654	0.919543334	0.036427799
Agpat5	-0.018262726	0.919543334	0.036427799
Rbm4	-0.018252424	0.919543334	0.036427799
Otof	0.022448813	0.919543334	0.036427799
Zfp185	-0.087654127	0.919551887	0.03642376
Tmem186	0.037393351	0.919551887	0.03642376
Fbxo36	0.025174941	0.919551887	0.03642376
Brix1	-0.038190097	0.919551887	0.03642376
Myo15	0.03097151	0.919551887	0.03642376
Dhx37	0.043888105	0.919551887	0.03642376
Wnt11	0.061493167	0.919551887	0.03642376
Selenbp1	0.086880892	0.919551887	0.03642376
Ddx5	-0.0359142	0.919551887	0.03642376
Spib	-0.025982937	0.919551887	0.03642376
Dleu2	-0.044114799	0.919551887	0.03642376
Med11	-0.018224408	0.919551887	0.03642376
Endou	-0.018215088	0.919551887	0.03642376
Slc9b1	-0.032256797	0.919551887	0.03642376
Hes3	0.033038926	0.919551887	0.03642376
Macrod1	-0.025203848	0.91956012	0.036419872
Baz2b	-0.086765539	0.919585171	0.036408041
Wdr19	-0.023796437	0.919651925	0.036376516
Trub1	-0.024398497	0.919685059	0.036360869
Ccdc148	-0.06545068	0.91979473	0.036309083
Zfp40	0.07876382	0.91979473	0.036309083
Tnfsf13b	0.066113183	0.919919324	0.036250258
Mgat4c	-0.029080748	0.919919324	0.036250258
Zfp146	0.044098016	0.920176854	0.036128695
Ei24	0.05496574	0.920323395	0.036059538
Emc8	-0.040764249	0.920500622	0.035975914
Gpr143	-0.087489785	0.920566212	0.035944969
Wdr61	0.045927221	0.920566212	0.035944969
Hspa5	-0.125144437	0.920566212	0.035944969
Dok4	0.050910895	0.920566212	0.035944969
Trappc3l	0.05807216	0.920566212	0.035944969
Ckmt2	0.073730334	0.920566212	0.035944969
Tex30	0.03033174	0.920566212	0.035944969
Tm9sf3	0.06325297	0.920566212	0.035944969
SKP1A	-0.066678158	0.920566212	0.035944969
Sftpc	0.069993024	0.920566212	0.035944969
Focad	0.140038746	0.920566212	0.035944969
Morf4l2	0.066023378	0.920566212	0.035944969
Pigw	-0.036314795	0.920566212	0.035944969
Fam214a	0.041970785	0.920566212	0.035944969
Atr	-0.047166063	0.920566212	0.035944969
Llph	-0.042733369	0.920566212	0.035944969

Igsf9	-0.107622874	0.920566212	0.035944969
Syce3	-0.033752461	0.920566212	0.035944969
Cep164	0.053610157	0.920566212	0.035944969
Atg3	0.096314676	0.920566212	0.035944969
Arsj	-0.048447379	0.920566212	0.035944969
Tuba1b	0.045375833	0.920566212	0.035944969
Lrrc43	-0.116160737	0.920566212	0.035944969
Zfp639	-0.053151261	0.920566212	0.035944969
Ankmy1	-0.031684881	0.920566212	0.035944969
Sarnp	-0.037909622	0.920566212	0.035944969
Gdf6	0.029866831	0.920566212	0.035944969
Clec1a	-0.045891723	0.920566212	0.035944969
Usf2	-0.025669902	0.920566212	0.035944969
Ankrd10	-0.023867654	0.920566212	0.035944969
Nmur1	-0.111646224	0.920566212	0.035944969
Stxbp4	0.024540958	0.920566212	0.035944969
Cmtm2b	-0.031316798	0.920566212	0.035944969
Teddm1a	-0.084850263	0.920566212	0.035944969
Spg7	0.046818158	0.920566212	0.035944969
Gtf2h1	0.032442548	0.920566212	0.035944969
Csnk1a1	0.053235435	0.920566212	0.035944969
Zfp706	0.04567063	0.920566212	0.035944969
Pard3	0.026060234	0.920566212	0.035944969
Syt10	0.061517254	0.920566212	0.035944969
BC01657[illegible]	-0.086225154	0.920566212	0.035944969
Grb7	-0.031044944	0.920566212	0.035944969
Slc5a12	-0.031062406	0.920566212	0.035944969
Dicer1	0.053605965	0.920566212	0.035944969
Dstyk	-0.035154321	0.920566212	0.035944969
Eif3a	-0.045253841	0.920566212	0.035944969
Alpk2	-0.027871698	0.920566212	0.035944969
Hspe1	-0.06213476	0.920566212	0.035944969
Efna1	0.049007986	0.920566212	0.035944969
Nup98	-0.04466348	0.920566212	0.035944969
Srrt	-0.031968543	0.920566212	0.035944969
Copg2	-0.032710707	0.920566212	0.035944969
Dmc1	0.028707465	0.920566212	0.035944969
Tom1	0.027160634	0.920566212	0.035944969
Lrsam1	-0.057799905	0.920566212	0.035944969
Msh6	0.076875163	0.920566212	0.035944969
Parp16	-0.026595811	0.920566212	0.035944969
Taf1b	0.054779089	0.920566212	0.035944969
Sap18b	0.040517675	0.920566212	0.035944969
Hemgn	-0.030928941	0.920566212	0.035944969
Actn1	0.024299493	0.920566212	0.035944969
Sik3	0.024127094	0.920566212	0.035944969
Hnrnpa3	-0.025527404	0.920566212	0.035944969
Fhl5	0.040848165	0.920566212	0.035944969

Cad	-0.047277341	0.920566212	0.035944969
Hdac10	-0.054642088	0.920566212	0.035944969
Six5	0.038985479	0.920566212	0.035944969
AA388235	-0.043327624	0.920566212	0.035944969
BC052040	-0.066929211	0.920566212	0.035944969
Muc19	0.018046341	0.920566212	0.035944969
Cacul1	-0.066675276	0.920629077	0.035915313
Zfp512b	-0.049129952	0.920629077	0.035915313
Itgad	-0.042139045	0.920629077	0.035915313
Pid1	-0.068187464	0.920629077	0.035915313
Med4	0.036219883	0.920629077	0.035915313
Cbx8	-0.033839928	0.920629077	0.035915313
Sumo2	0.052843201	0.920629077	0.035915313
Zmat5	-0.040991674	0.920629077	0.035915313
Nkain4	-0.054751824	0.920629077	0.035915313
Exosc2	0.03546957	0.920629077	0.035915313
Pcdhb14	-0.05611214	0.920629077	0.035915313
Akr1e1	-0.064154362	0.920629077	0.035915313
Amfr	-0.040200715	0.920629077	0.035915313
Sfpq	0.02660469	0.920629077	0.035915313
Lrpap1	0.049093548	0.920629077	0.035915313
Snupn	-0.040792917	0.920629077	0.035915313
Chd1l	0.060113175	0.920629077	0.035915313
Tnp2	0.043971646	0.920629077	0.035915313
Rabl3	-0.023159726	0.920629077	0.035915313
Uhrf2	-0.037487224	0.920629077	0.035915313
Agxt	0.060059283	0.920629077	0.035915313
Slc35a5	-0.029286552	0.920629077	0.035915313
Ms4a2	-0.03113741	0.920629077	0.035915313
Slc6a19	0.071388685	0.920629077	0.035915313
Ufl1	0.045458613	0.920629077	0.035915313
Lilra6	0.050790505	0.920629077	0.035915313
Akr7a5	0.031575154	0.920629077	0.035915313
Mrps26	-0.032618864	0.920629077	0.035915313
Pdcd4	0.036255341	0.920629077	0.035915313
AV320801	-0.037902475	0.920629077	0.035915313
Copa	0.034559362	0.920629077	0.035915313
Zik1	0.040675916	0.920629077	0.035915313
Synpr	-0.052064205	0.920629077	0.035915313
Ubxn8	-0.035695774	0.920629077	0.035915313
Plcl1	-0.049618525	0.920629077	0.035915313
Tnfaip8l3	-0.072820783	0.920629077	0.035915313
Cmtm1	0.112700308	0.920629077	0.035915313
Duox1	-0.035880767	0.920629077	0.035915313
Abca17	0.113097449	0.920629077	0.035915313
Tcf4	0.029720967	0.920629077	0.035915313
Pprc1	0.029241509	0.920629077	0.035915313
Trpm5	-0.041453484	0.920629077	0.035915313

Bclaf1	-0.02481738	0.920629077	0.035915313
Jrkl	0.028834675	0.920629077	0.035915313
Olfr1378	0.02121431	0.920629077	0.035915313
Osbp	-0.021022608	0.920629077	0.035915313
Sec61a2	-0.038659316	0.920629077	0.035915313
Smyd4	0.039260779	0.920629077	0.035915313
Ttc38	0.04562014	0.920629077	0.035915313
Rbm15b	0.021248942	0.920629077	0.035915313
Tmco3	-0.020069646	0.920629077	0.035915313
Ino80d	-0.031424325	0.920629077	0.035915313
Lemd2	0.024311634	0.920629077	0.035915313
Timm50	0.027761705	0.920629077	0.035915313
Mrfap1	-0.048721669	0.920629077	0.035915313
Capns2	0.049833486	0.920629077	0.035915313
Mtmr6	-0.02478704	0.921002083	0.035739388
Txlnb	0.049589032	0.92104474	0.035719273
Tpsb2	0.055677447	0.92104474	0.035719273
Eda	-0.080952122	0.92104474	0.035719273
Ddb1	0.048612378	0.92104474	0.035719273
Nudt9	0.026543555	0.92104474	0.035719273
Safb	0.048439337	0.92104474	0.035719273
Snapc5	0.043709586	0.92104474	0.035719273
Rwdd2b	-0.02417055	0.921201424	0.035645399
Slc25a40	-0.079483963	0.921257621	0.035618907
Exosc4	-0.040677368	0.921257621	0.035618907
Dhx15	-0.060103579	0.921257621	0.035618907
Polr3gl	-0.051213381	0.921257621	0.035618907
Csnk2a1	0.07701282	0.921257621	0.035618907
Ist1	-0.033380613	0.921257621	0.035618907
Trp53rkb	0.026561652	0.921257621	0.035618907
Haus2	0.048027703	0.921257621	0.035618907
CISD3B	0.030488113	0.921257621	0.035618907
Gstcd	-0.036855427	0.921257621	0.035618907
Prdm12	-0.035579056	0.921257621	0.035618907
En1	0.076381512	0.921257621	0.035618907
Gpr161	0.101678876	0.921257621	0.035618907
Orai3	-0.064911515	0.921370719	0.035565594
FAM19A2	-0.031551659	0.921370719	0.035565594
Upf3b	0.037732285	0.921370719	0.035565594
Tmem39b	-0.066055358	0.921370719	0.035565594
Prpf6	-0.017637823	0.921370719	0.035565594
Lats2	-0.108193585	0.921379912	0.035561261
Edf1	-0.049795965	0.921379912	0.035561261
Fndc1	-0.04098252	0.921379912	0.035561261
Apold1	0.03472606	0.921379912	0.035561261
E2f3	-0.03408206	0.921379912	0.035561261
Olfr722	0.051138621	0.921379912	0.035561261
Ldah	-0.036551424	0.921379912	0.035561261

Xlr5c	-0.046475081	0.921379912	0.035561261
Plekhg3	0.027477046	0.921379912	0.035561261
Tmod4	-0.045122022	0.921406326	0.03554881
Tomm70a	0.036473336	0.921406326	0.03554881
Myt1	0.037805226	0.921406326	0.03554881
Dbh	0.059925803	0.921406326	0.03554881
Cpeb2	0.066338399	0.921406326	0.03554881
Cd3eap	-0.045500634	0.921406326	0.03554881
Snx10	0.103862606	0.921406326	0.03554881
Pex12	0.058265077	0.921468055	0.035519716
Tmem106	0.079656755	0.92148065	0.03551378
Strc	0.044800259	0.92148065	0.03551378
Hmbox1	0.027877713	0.92148065	0.03551378
Dmrta2os	0.028027187	0.92148065	0.03551378
Nrbp2	-0.121238531	0.921529001	0.035490993
Dnmt3a	0.042479691	0.921564338	0.03547434
Tomm20	-0.033052494	0.921589863	0.035462311
Numb	0.034703702	0.921589863	0.035462311
Katnal2	0.020138575	0.921589863	0.035462311
Slc5a11	0.091091987	0.921680364	0.035419665
Praf2	-0.038842827	0.921680364	0.035419665
Prrt4	-0.094051953	0.921680364	0.035419665
Dus1l	0.074287818	0.921680364	0.035419665
ADPRHL2	-0.025354807	0.921680364	0.035419665
Ndufs8	0.03308028	0.921680364	0.035419665
Mis12	0.025902326	0.921680364	0.035419665
Dhcr7	0.019878013	0.921680364	0.035419665
Dnajb11	-0.089502942	0.921680364	0.035419665
Plekhg6	-0.109491633	0.921680364	0.035419665
Pigx	-0.036714814	0.921680364	0.035419665
Nat8	0.031318465	0.921680364	0.035419665
Sepsecs	0.075740255	0.921722589	0.035399769
Atp5g1	-0.245299793	0.921799953	0.035363318
Lpar1	-0.046896806	0.921799953	0.035363318
Pdcd2	0.044848868	0.921799953	0.035363318
Gnrhr	-0.107025766	0.921801672	0.035362508
Zfp24	0.061843398	0.921801672	0.035362508
Sbsn	0.079341664	0.922104986	0.03521963
Fam81b	0.082587656	0.922104986	0.03521963
Fancc	-0.058928046	0.922104986	0.03521963
Olfr688	-0.036724465	0.922104986	0.03521963
Slc38a11	-0.083047799	0.922104986	0.03521963
Pan3	0.02245841	0.922104986	0.03521963
Sptlc1	-0.036130082	0.922104986	0.03521963
Tbx22	0.022450042	0.922104986	0.03521963
Vldlr	0.073949759	0.922104986	0.03521963
D17ERTD6	0.040300313	0.922104986	0.03521963
Cep162	0.034897993	0.922104986	0.03521963

Ssna1	-0.025335761	0.922104986	0.03521963
Ptrh1	-0.064872511	0.922104986	0.03521963
Sirpa	0.032966354	0.922104986	0.03521963
Pax8	0.066816859	0.922104986	0.03521963
Mmadhc	-0.054992734	0.922104986	0.03521963
Dsc3	0.031288445	0.922104986	0.03521963
Tmc5	0.036033672	0.922104986	0.03521963
Tdrd3	-0.067558837	0.922250914	0.035150906
Phf8	-0.06112389	0.922250914	0.035150906
Ddx3x	0.030622999	0.922250914	0.035150906
Smim15	0.049432893	0.922250914	0.035150906
Pinlyp	-0.071845429	0.922250914	0.035150906
Msgn1	0.030823371	0.922250914	0.035150906
Chfr	-0.059924795	0.922250914	0.035150906
Tmem138	-0.032859865	0.922250914	0.035150906
Dynlt1a	-0.028336249	0.922250914	0.035150906
BC03050(	0.040676721	0.922250914	0.035150906
Unc119b	-0.073363795	0.922263324	0.035145062
Theg	0.034786168	0.922301605	0.035127035
Med29	0.032079517	0.922301605	0.035127035
Cfap221	0.036428051	0.922301605	0.035127035
Prr23a1	0.085511961	0.922421575	0.035070548
Sf3b3	-0.067690182	0.922484608	0.035040871
Fam136a	0.023377768	0.922562873	0.035004027
Ggnbp2	0.0494779	0.922617431	0.034978345
Rps27	-0.057392349	0.922617431	0.034978345
Pbdc1	0.047527344	0.922617431	0.034978345
Adamts8	-0.034838734	0.922617431	0.034978345
Tmem174	-0.028763839	0.922617431	0.034978345
Chodl	-0.045418775	0.922617431	0.034978345
Fbxo7	0.032922119	0.922617431	0.034978345
Trim60	0.081839047	0.922617431	0.034978345
Cutal	-0.026888911	0.922617431	0.034978345
Nmnat1	-0.028939804	0.922617431	0.034978345
Chil5	0.032750748	0.922617431	0.034978345
TMEM261	-0.033516222	0.922617431	0.034978345
Wasf3	-0.034976364	0.922617431	0.034978345
Eif3j2	-0.074150283	0.922617431	0.034978345
LINCRED1	-0.02690971	0.922617431	0.034978345
Dcaf11	0.048679521	0.922617431	0.034978345
Slc25a3	0.028972675	0.922617431	0.034978345
Prdx5	-0.017295257	0.922617431	0.034978345
Atp6v1b1	-0.028788902	0.922617431	0.034978345
Gtpbp2	0.025050552	0.922617431	0.034978345
Elfn1	-0.059921895	0.922797742	0.034893477
Klra5	0.02275318	0.922797742	0.034893477
Tmem59	0.067740872	0.922797742	0.034893477
Musk	0.08606228	0.922797742	0.034893477

Hmgxb4	0.034143464	0.922797742	0.034893477
Ccdc137	0.07966189	0.922797742	0.034893477
BC049352	-0.017951054	0.922797742	0.034893477
Gast	0.029242317	0.922797742	0.034893477
Taf2	-0.084856318	0.922797742	0.034893477
Plagl1	0.098623804	0.922866561	0.03486109
Kcmf1	-0.086442783	0.922952876	0.034820473
Sec14l1	-0.117709168	0.922952876	0.034820473
Rrm2b	-0.068216186	0.922952876	0.034820473
Speer2	0.035903995	0.922952876	0.034820473
Mif	-0.031647306	0.922952876	0.034820473
Sox5os4	-0.101307635	0.922952876	0.034820473
CASC4	-0.026018933	0.922952876	0.034820473
Srsf7	0.039840337	0.922952876	0.034820473
Fermt2	-0.030614498	0.922952876	0.034820473
Mrpl27	-0.056216553	0.922952876	0.034820473
Cngb3	-0.048839709	0.922952876	0.034820473
MIRA	-0.064654022	0.922952876	0.034820473
Ttc30b	0.044759176	0.922952876	0.034820473
Gnat1	-0.027456539	0.922952876	0.034820473
Gp5	-0.046099538	0.922952876	0.034820473
Mlycd	-0.048712465	0.922952876	0.034820473
Acsf3	-0.053344451	0.922952876	0.034820473
Klra8	0.068965164	0.922952876	0.034820473
Fgf22	-0.026920325	0.922952876	0.034820473
Qrsl1	0.017195085	0.922952876	0.034820473
Lman1l	0.0248362	0.923034965	0.034781847
Cggbp1	-0.20844633	0.923073838	0.034763558
Cpb1	0.132791069	0.923073838	0.034763558
Mapk1	-0.026016938	0.923073838	0.034763558
Tmtc2	-0.089833017	0.923073838	0.034763558
Top2b	0.06660848	0.923073838	0.034763558
Sar1b	0.058839636	0.923073838	0.034763558
Man2c1os	0.11832798	0.923073838	0.034763558
Pate2	0.105307999	0.923073838	0.034763558
Ocln	-0.017157606	0.923073838	0.034763558
Txlna	0.027110012	0.923073838	0.034763558
Sos1	-0.031493425	0.923138144	0.034733304
Zcchc13	0.027025423	0.923138144	0.034733304
Cptp	-0.031064931	0.923138144	0.034733304
Sh2d3c	0.028889608	0.923159706	0.03472316
Mettl22	-0.026855549	0.923217768	0.034695846
Vps37a	0.060859962	0.9232333	0.034688539
Lrrfip1	0.119874205	0.9232333	0.034688539
Gpr107	0.042004452	0.9232333	0.034688539
Oxct2a	0.04243539	0.9232333	0.034688539
Tamm41	-0.035086799	0.9232333	0.034688539
F11	-0.046332759	0.9232333	0.034688539

Txndc16	0.028231735	0.92331381	0.034650669
Taok2	-0.041918107	0.92331381	0.034650669
Platr25	-0.038147077	0.92331381	0.034650669
Iqce	0.091218695	0.92331381	0.034650669
Slc37a4	-0.064126772	0.92331381	0.034650669
Vax2	0.025072809	0.92331381	0.034650669
Dkk1	0.064509612	0.92331381	0.034650669
Taok1	-0.038964388	0.92331381	0.034650669
Nrg4	-0.033986782	0.92331381	0.034650669
Zdhhc13	-0.031333777	0.92331381	0.034650669
Tars2	0.03152757	0.92331381	0.034650669
Guca2b	0.07182598	0.92331381	0.034650669
Ostn	0.067384663	0.92331381	0.034650669
Ubxn10	0.021672606	0.92331381	0.034650669
Ermp1	0.074514467	0.92331381	0.034650669
AW54987	-0.038683066	0.92331381	0.034650669
Cyp2b10	0.06090832	0.92331381	0.034650669
Mdga1	0.074915784	0.92331381	0.034650669
Mgat2	-0.030077946	0.92331381	0.034650669
Prn	0.043564343	0.92331381	0.034650669
Tut1	0.019552757	0.92331381	0.034650669
Wdhd1	0.073484734	0.92331381	0.034650669
Timm17a	-0.017056193	0.92331381	0.034650669
Pdcd6ip	-0.027252858	0.92331381	0.034650669
Esr1	0.036580157	0.92331381	0.034650669
Mrgprf	0.019018487	0.92331381	0.034650669
Tnr	0.01703952	0.92331381	0.034650669
Mgme1	0.08065075	0.92378279	0.034430133
Tex33	-0.026920427	0.92378279	0.034430133
Rpl32l	0.02611551	0.92378279	0.034430133
Mrps22	-0.032931002	0.92378279	0.034430133
Acly	0.043905294	0.92378279	0.034430133
Cdc14b	-0.032398211	0.92378279	0.034430133
Carf	-0.017002523	0.92378279	0.034430133
Arid4b	0.080695752	0.92378279	0.034430133
Vps39	-0.027762979	0.923827405	0.034409159
Syncrip	0.066408615	0.923827405	0.034409159
Zfp62	-0.083337856	0.923868063	0.034390046
Tlr5	0.052948771	0.923868063	0.034390046
St3gal4	-0.100385047	0.923868063	0.034390046
Nup54	-0.036075567	0.923868063	0.034390046
Vmac	-0.083099531	0.923868063	0.034390046
Atp11b	-0.045206758	0.923868063	0.034390046
Mbd3l2	-0.119760908	0.923868063	0.034390046
Fra10ac1	-0.047480703	0.923868063	0.034390046
Rph3al	-0.044721503	0.923868063	0.034390046
Zkscan1	0.027365201	0.923868063	0.034390046
Nme1	-0.050920003	0.923868063	0.034390046

HIST1H4K	0.054218917	0.923868063	0.034390046
Trappc9	0.035994437	0.923868063	0.034390046
Ikbkg	0.027538206	0.923868063	0.034390046
Pik3r1	-0.050864329	0.923868063	0.034390046
Ube2d3	-0.029776307	0.923868063	0.034390046
Sytl5	-0.058591831	0.923868063	0.034390046
Abat	-0.027449242	0.923868063	0.034390046
Tssk4	-0.029283771	0.923868063	0.034390046
Srprb	-0.029575511	0.923868063	0.034390046
Adsl	0.025856292	0.923868063	0.034390046
Zfand4	0.029461233	0.923868063	0.034390046
Zfp850	-0.0256224	0.923868063	0.034390046
Ranbp3	-0.100045269	0.923868063	0.034390046
Snhg12	-0.070754218	0.923868063	0.034390046
Polr3f	-0.040001736	0.923868063	0.034390046
Ik	-0.049846293	0.923868063	0.034390046
Tas1r3	0.02687867	0.923868063	0.034390046
Osgin2	0.036052677	0.923868063	0.034390046
Tmem65	-0.046942298	0.923868063	0.034390046
Agbl5	-0.042192137	0.923916069	0.034367479
Ccdc153	-0.088910482	0.923969291	0.034342463
Odf3	0.047548026	0.923969291	0.034342463
Cenpc1	-0.071491999	0.923969291	0.034342463
Ehmt2	0.047313721	0.923969291	0.034342463
Iqca	-0.041035472	0.923969291	0.034342463
Cwc22	0.044588328	0.923969291	0.034342463
Tbx20	-0.054609335	0.923969291	0.034342463
Hddc2	0.043243798	0.923969291	0.034342463
Slc25a5	-0.039875703	0.923969291	0.034342463
Nlrp6	0.066510785	0.923969291	0.034342463
Caap1	0.081871189	0.923969291	0.034342463
Cish	0.031067052	0.923969291	0.034342463
Prlh	-0.058902025	0.923969291	0.034342463
Ccdc163	-0.023929634	0.923969291	0.034342463
Dbr1	0.040403262	0.923969291	0.034342463
Map7	-0.053088491	0.923969291	0.034342463
Rubcn	-0.033688318	0.923969291	0.034342463
GCN1L1	0.066949365	0.923969291	0.034342463
Ces2e	-0.034168805	0.923969291	0.034342463
Sel1l2	0.056026744	0.924029858	0.034313995
Gdpgp1	0.036649185	0.924029858	0.034313995
Gata4	0.059550513	0.924057516	0.034300996
Gpr142	-0.027870685	0.924057516	0.034300996
BC046251	0.102884527	0.924057516	0.034300996
Tmem255	-0.034570101	0.924057516	0.034300996
Cilp	0.065332996	0.924057516	0.034300996
FAM96B	0.021023487	0.924057516	0.034300996
Daw1	0.022171721	0.924149356	0.034257835

Rfesd	-0.061248757	0.924187733	0.0342398
Amz2	0.042227871	0.924187733	0.0342398
Prim2	0.032954683	0.924187733	0.0342398
Nop56	-0.036782222	0.924187733	0.0342398
Pbld2	0.123128318	0.924187733	0.0342398
Acot6	0.062755753	0.924187733	0.0342398
Slc25a11	-0.055766494	0.924187733	0.0342398
Usp16	0.041602183	0.924187733	0.0342398
Ccdc14	0.03518767	0.924187733	0.0342398
Zfp329	-0.028123197	0.924187733	0.0342398
Vwa3b	-0.03301872	0.924187733	0.0342398
Tbck	0.100366176	0.924187733	0.0342398
Pde9a	0.024050198	0.924187733	0.0342398
Nsun3	0.033867846	0.924187733	0.0342398
P4ha2	-0.030935156	0.924187733	0.0342398
Trim10	0.044825805	0.924187733	0.0342398
Hnrnpd	0.044932563	0.924187733	0.0342398
Ppdpf	-0.023704108	0.924187733	0.0342398
Gmeb1	0.041740888	0.924187733	0.0342398
Lrrc26	0.026221835	0.924187733	0.0342398
Rtp1	0.035069199	0.924187733	0.0342398
Cbr1	-0.035497783	0.924187733	0.0342398
Cpa2	0.072859101	0.924187733	0.0342398
Klf5	0.041471152	0.924187733	0.0342398
Fbxw8	0.027525861	0.924187733	0.0342398
Unc45bos	0.049422626	0.924187733	0.0342398
Smarcc1	0.031989406	0.924187733	0.0342398
H2AFV	0.077164177	0.924187733	0.0342398
Stau1	-0.023941568	0.924187733	0.0342398
Nkx2-5	0.086920997	0.924187733	0.0342398
Igfl3	-0.03368184	0.924187733	0.0342398
Ahctf1	0.018518838	0.924187733	0.0342398
Tmcc1	0.029086257	0.924187733	0.0342398
Foxi2	0.036969767	0.924187733	0.0342398
Poldip2	0.030176257	0.924187733	0.0342398
CECR5	0.021711305	0.924187733	0.0342398
Pcnt	0.032876426	0.924187733	0.0342398
Alg10b	0.0311319	0.924187733	0.0342398
Spata22	-0.037619525	0.924187733	0.0342398
Fancl	-0.017684807	0.924187733	0.0342398
Nfasc	-0.022699756	0.924187733	0.0342398
BC00205[illegible]	-0.01675034	0.924187733	0.0342398
Lce3f	0.032657117	0.924187733	0.0342398
Rfwd3	-0.065306513	0.924187733	0.0342398
Mkrn2	-0.022643501	0.924187733	0.0342398
Ndufa11	-0.052693696	0.924215256	0.034226867
Zfp386	0.036805214	0.924337938	0.034169222
Defb22	0.042752144	0.924337938	0.034169222

Grina	0.031745326	0.924478875	0.034103008
Lypd8	0.101087726	0.924478875	0.034103008
Tvp23a	-0.082556448	0.924591058	0.034050311
Zfp652	-0.070410401	0.924591058	0.034050311
Rtn4ip1	-0.076375005	0.924591058	0.034050311
Mtdh	0.039611925	0.924591058	0.034050311
Ubr7	-0.055253636	0.924591058	0.034050311
PAPD5	0.075597295	0.924591058	0.034050311
Slc50a1	0.070201075	0.924591058	0.034050311
Hnrnpk	0.089323639	0.924591058	0.034050311
Stpg3	-0.050419614	0.924591058	0.034050311
Nudt13	0.068625815	0.924591058	0.034050311
Gstm1	0.02487365	0.924591058	0.034050311
Il4	0.045298354	0.924591058	0.034050311
Sez6	0.048849384	0.924591058	0.034050311
Pard6b	0.034176528	0.924591058	0.034050311
Nrd1	0.036345822	0.924591058	0.034050311
Ppp1r1a	-0.036656272	0.924591058	0.034050311
Zfp281	-0.036248769	0.924591058	0.034050311
Wdr18	0.041742183	0.924591058	0.034050311
Gckr	0.060848865	0.924591058	0.034050311
Pard3bos2	-0.048820282	0.924591058	0.034050311
Capn7	-0.053140266	0.924591058	0.034050311
Spopl	-0.029634922	0.924591058	0.034050311
AU022754	0.033754641	0.924591058	0.034050311
Tbx2	0.030824315	0.924591058	0.034050311
Myo1b	0.034445867	0.924591058	0.034050311
Mzt2	0.029552236	0.924591058	0.034050311
Nup88	0.049130972	0.924591058	0.034050311
Zfp111	0.030715282	0.924591058	0.034050311
Olfr1417	0.026536414	0.924591058	0.034050311
Mlst8	0.025097233	0.924591058	0.034050311
Prpsap1	-0.020116511	0.924591058	0.034050311
Pdgfb	0.039910008	0.924591058	0.034050311
Vps28	-0.036791453	0.924591058	0.034050311
Ptgfrn	-0.030640166	0.924591058	0.034050311
Dusp6	0.033128129	0.924591058	0.034050311
Polr2k	-0.023193518	0.924591058	0.034050311
Mc2r	0.033746688	0.924591058	0.034050311
Ms4a1	-0.069404584	0.924591058	0.034050311
Vmn1r32	-0.109675403	0.924663796	0.034016146
Gsdma2	-0.080189718	0.924663796	0.034016146
Lhx3	0.028427193	0.924663796	0.034016146
Mab21l3	-0.029067603	0.924663796	0.034016146
Ropn1l	-0.032642285	0.924663796	0.034016146
Atp8b1	0.065967885	0.924663796	0.034016146
Tbc1d23	-0.021851849	0.924663796	0.034016146
D10JHU81	0.021780502	0.924663796	0.034016146

Desi2	0.016508494	0.924663796	0.034016146
Nynrin	0.046898713	0.924793633	0.033955169
Wfdc2	0.049102756	0.924793633	0.033955169
Nit1	0.05201418	0.924793633	0.033955169
Entpd5	0.129913701	0.924793633	0.033955169
Lncenc1	-0.038805068	0.924793633	0.033955169
BC048609	0.031619008	0.924793633	0.033955169
Clgn	0.057553824	0.925169453	0.033778715
Omd	0.064063539	0.925205804	0.033761652
BC107364	-0.041986138	0.925205804	0.033761652
Gimap9	-0.049876665	0.925265158	0.033733792
Uqcrfs1	0.025357003	0.925349525	0.033694194
Slc35c1	-0.017885234	0.925349525	0.033694194
Apmap	-0.034635843	0.925397128	0.033671853
Kctd6	-0.027003501	0.925397128	0.033671853
Psmb3	0.074319413	0.925591521	0.033580632
Sspo	0.053176903	0.925635956	0.033559784
Prmt2	-0.070436535	0.925635956	0.033559784
Unc5b	-0.046166998	0.925635956	0.033559784
Slc5a8	0.041913316	0.925635956	0.033559784
Ccdc88a	0.034079989	0.925635956	0.033559784
Apon	0.017947892	0.925635956	0.033559784
Lrrc61	-0.033223612	0.925635956	0.033559784
Fbxl21	0.035952918	0.925635956	0.033559784
Nnt	0.059752266	0.925635956	0.033559784
Lsm12	0.026185895	0.925635956	0.033559784
Gk5	0.09511486	0.925635956	0.033559784
Ngdn	-0.049755649	0.925684077	0.033537207
Pcdh17	0.08275855	0.925684077	0.033537207
Hgfac	0.048813077	0.925684077	0.033537207
Cwc27	-0.049833415	0.925684077	0.033537207
Phactr2	0.058598881	0.925684077	0.033537207
Trappc4	-0.025284926	0.925684077	0.033537207
Ubash3a	0.034131212	0.925684077	0.033537207
Helt	-0.082652733	0.925684077	0.033537207
Ccdc191	0.056137241	0.925723747	0.033518596
Fam171a1	-0.036721828	0.925723747	0.033518596
Phf23	0.058863685	0.925723747	0.033518596
Zfp747	0.029431389	0.925723747	0.033518596
Zfp120	0.051924577	0.925909539	0.033431442
Svep1	0.029487724	0.925909539	0.033431442
Ahsa2	0.069150551	0.925909539	0.033431442
Yipf6	0.032236512	0.925909539	0.033431442
Fam131c	0.047059512	0.925909539	0.033431442
Mrps33	0.028954164	0.925909539	0.033431442
Ctcf	-0.016332972	0.925909539	0.033431442
Dpysl4	0.01835908	0.925909539	0.033431442
Slc39a5	-0.068263169	0.926118774	0.033333312

Adgre4	-0.061023107	0.926118774	0.033333312
Peak1os	-0.029871091	0.926118774	0.033333312
Map1s	-0.024350151	0.926118774	0.033333312
Adam4	0.026457024	0.926118774	0.033333312
MYEOV2	0.02606278	0.926118774	0.033333312
Pigs	-0.051618037	0.926118774	0.033333312
Pin4	-0.027441087	0.926118774	0.033333312
Arhgap8	-0.036555118	0.926118774	0.033333312
Sema5b	0.038447156	0.926134893	0.033325753
Cbx5	0.02794644	0.926134893	0.033325753
SELT	-0.033228098	0.926134893	0.033325753
Bace1	-0.0426606	0.926134893	0.033325753
Ret	0.028588737	0.926134893	0.033325753
Sec22b	0.029279076	0.926235661	0.033278502
Cox16	0.078551525	0.926235661	0.033278502
Fam184a	0.03772469	0.926392312	0.033205058
Svs3b	0.033221793	0.926392312	0.033205058
R3hdml	0.044908069	0.926392312	0.033205058
Ccdc25	-0.016260959	0.926392312	0.033205058
Aplp1	0.016257209	0.926392312	0.033205058
Hsp90b1	-0.031032776	0.92643691	0.033184151
Il17d	-0.049481465	0.926627451	0.033094838
Cxcl14	-0.048382679	0.926627451	0.033094838
Best2	0.03310944	0.927049285	0.032897177
Bco1	0.060105701	0.927115742	0.032866045
Cdkn2aip	0.088866753	0.927149402	0.032850277
Senp6	-0.024392022	0.927324039	0.032768482
Akap12	0.090835046	0.927516286	0.032678456
Gfra3	-0.099943689	0.927516286	0.032678456
Heyl	-0.066671918	0.927516286	0.032678456
Rpf1	-0.035135385	0.927516286	0.032678456
Cnih2	-0.029746902	0.927516286	0.032678456
Hoxa2	0.03561324	0.927516286	0.032678456
Them7	-0.112531526	0.927516286	0.032678456
Tbrg4	-0.03568946	0.927516286	0.032678456
Ftcd	-0.034051019	0.927516286	0.032678456
Eif1ad	0.042691329	0.927627687	0.032626297
Pigq	-0.031799818	0.927646032	0.032617709
Sptlc2	0.044025651	0.927646032	0.032617709
Hccs	-0.017664063	0.927646032	0.032617709
Anapc11	-0.026507425	0.92770084	0.03259205
Grid2	0.126195449	0.927807599	0.032542075
Cntln	0.02905737	0.927807599	0.032542075
Tmem106	-0.02805921	0.927807599	0.032542075
FAM63B	-0.112749172	0.92804673	0.032430155
Kdm5b	-0.035281594	0.92804673	0.032430155
Tead4	0.025034728	0.92804673	0.032430155
Aldh3a2	-0.0540107	0.92804673	0.032430155

Nkd1	-0.060073725	0.92804673	0.032430155
Mterf3	-0.032847738	0.92804673	0.032430155
Nxnl1	0.034839493	0.92804673	0.032430155
Tmem9	-0.093548144	0.928090361	0.032409738
Gabra2	-0.051232857	0.928090361	0.032409738
Vmn2r86	0.049034529	0.928090361	0.032409738
Tmem51o:	-0.088481424	0.928090361	0.032409738
Slk	-0.074059741	0.928090361	0.032409738
Polr3h	0.042698838	0.928090361	0.032409738
Rasef	-0.95423329	0.928090361	0.032409738
Glra1	0.031933941	0.928090361	0.032409738
Plac1	0.083238186	0.928090361	0.032409738
Nabp1	-0.040623956	0.928090361	0.032409738
Ube4b	-0.066092181	0.928090361	0.032409738
Zcchc12	0.031403579	0.928090361	0.032409738
Gtpbp6	-0.066728345	0.928090361	0.032409738
Abhd12	0.02773776	0.928090361	0.032409738
H2-M1	-0.0381781	0.928090361	0.032409738
Triap1	0.030557305	0.928090361	0.032409738
Gnl2	-0.057256421	0.928090361	0.032409738
Rae1	-0.043707902	0.928090361	0.032409738
Zmiz1os1	-0.031790815	0.928090361	0.032409738
Bmt2	0.036441909	0.928090361	0.032409738
Sebox	-0.241280409	0.928090361	0.032409738
Xndc1	-0.04471841	0.928090361	0.032409738
Fgf16	-0.03052435	0.928090361	0.032409738
Ift140	0.074989103	0.928090361	0.032409738
Pbx2	0.024910619	0.928090361	0.032409738
Acot8	-0.059387287	0.928090361	0.032409738
Aen	-0.037893653	0.928090361	0.032409738
Blvra	0.03160278	0.928090361	0.032409738
Mfhas1	0.038870903	0.928090361	0.032409738
Ctbp2	0.034060138	0.928090361	0.032409738
Tnfrsf8	0.081234736	0.928090361	0.032409738
Tmem242	0.033298217	0.928090361	0.032409738
Chst15	0.024932843	0.928090361	0.032409738
St6galnac:	0.051787248	0.928090361	0.032409738
FAM206A	-0.023295046	0.928090361	0.032409738
Gnal	-0.062673073	0.928090361	0.032409738
ARMC4	0.05110202	0.928090361	0.032409738
Henmt1	0.029539698	0.928090361	0.032409738
PQLC2	-0.023308822	0.928090361	0.032409738
Acin1	-0.041264305	0.928090361	0.032409738
Ptcra	0.02531672	0.928090361	0.032409738
Tbl2	-0.044448336	0.928090361	0.032409738
Rdh13	-0.024766934	0.928090361	0.032409738
FAM192A	-0.047700332	0.928090361	0.032409738
Pcbp4	0.0271426	0.928090361	0.032409738

Enox2	0.062962251	0.928090361	0.032409738
Nfyc	0.032701389	0.928090361	0.032409738
Cct2	0.032810727	0.928090361	0.032409738
Prok1	-0.02961861	0.928090361	0.032409738
Ptx4	0.038512016	0.928090361	0.032409738
Cers5	0.028061233	0.928090361	0.032409738
Ppp4r2	0.01858211	0.928090361	0.032409738
Klhdc8b	-0.036580367	0.928090361	0.032409738
Hdlbp	-0.047778158	0.928090361	0.032409738
Zmym1	0.022068405	0.928090361	0.032409738
Klhdc1	-0.02676677	0.928090361	0.032409738
Glo1	-0.028764581	0.928090361	0.032409738
Nav2	-0.04634787	0.928090361	0.032409738
Ddx39b	-0.017640413	0.928090361	0.032409738
BC05339[illegible]	-0.017600222	0.928090361	0.032409738
Atp6v0a4	0.038496254	0.928090361	0.032409738
Cstf1	0.015947688	0.928090361	0.032409738
Pccb	-0.098936559	0.928140387	0.032386329
Epc1	0.066753728	0.928140387	0.032386329
Opn4	-0.049630871	0.928140387	0.032386329
Gspt1	0.054282153	0.928140387	0.032386329
CD163L1	-0.051779418	0.928140387	0.032386329
Map3k2	0.040255886	0.928439575	0.032246356
Rprm	0.053541183	0.928439575	0.032246356
Gpr119	-0.033861333	0.928439575	0.032246356
Abcc10	0.033644679	0.928439575	0.032246356
Mst1	0.027418331	0.928439575	0.032246356
Impg2	0.034698726	0.928439575	0.032246356
Fam209	0.02686884	0.928439575	0.032246356
Lsm8	0.036849682	0.928439575	0.032246356
Pgap3	0.029318212	0.928439575	0.032246356
Sf3a3	0.028609355	0.928439575	0.032246356
Dot1l	-0.059214724	0.928439575	0.032246356
Gtpbp4	0.024912553	0.928439575	0.032246356
Hsd17b12	-0.019787345	0.928439575	0.032246356
Chmp1a	-0.029473458	0.928439575	0.032246356
Nsun7	0.027184248	0.928439575	0.032246356
Msl3	0.035152785	0.928439575	0.032246356
Cdk2ap2	0.02431074	0.928439575	0.032246356
Ncoa3	0.060244433	0.928439575	0.032246356
Lrrcc1	0.060620784	0.928475603	0.032229504
Fam71d	0.053963299	0.928475603	0.032229504
Ankrd26	0.96219415	0.928475603	0.032229504
Nop2	-0.03175138	0.928475603	0.032229504
Bach2	0.048987909	0.928475603	0.032229504
Umps	-0.025482974	0.928475603	0.032229504
Car12	0.026424073	0.928475603	0.032229504
Ccdc142	-0.023751796	0.928475603	0.032229504

Matn2	0.054069431	0.928475603	0.032229504
Il10	-0.020452696	0.928475603	0.032229504
Prpf4b	0.051947055	0.928475603	0.032229504
Sco1	0.030058049	0.928475603	0.032229504
Fam50b	0.033692428	0.928475603	0.032229504
Mmp1a	-0.036678783	0.928475603	0.032229504
Caprin2	-0.028267227	0.928475603	0.032229504
Ppm1g	-0.041788796	0.928475603	0.032229504
Naa40	0.038156416	0.928475603	0.032229504
Tfap2a	0.031984827	0.928475603	0.032229504
Gpc3	-0.028701069	0.928475603	0.032229504
Chmp5	-0.031600845	0.928475603	0.032229504
Setd3	-0.015813743	0.928475603	0.032229504
Ubap2l	-0.045703135	0.928486434	0.032224437
Mrps23	0.047932914	0.92860719	0.032167958
Spem1	0.036255334	0.92860719	0.032167958
C6	-0.051159584	0.92860719	0.032167958
G6B	0.055209648	0.92860719	0.032167958
Adprhl1	-0.064086	0.92860719	0.032167958
Gfpt1	-0.069388637	0.92860719	0.032167958
MPP5	0.016697887	0.92860719	0.032167958
Rfxap	0.027849392	0.92860719	0.032167958
Chst11	0.06622152	0.92860719	0.032167958
Tctn1	0.033588029	0.92860719	0.032167958
Sst	-0.031920035	0.928787551	0.032083614
Cyp2c55	0.02880488	0.928787551	0.032083614
Hspa13	0.019210213	0.928835348	0.032061266
St8sia3os	0.080606429	0.928865251	0.032047284
Apol11b	0.017980342	0.929050564	0.031960649
Dcdc2c	-0.035049503	0.929157033	0.031910881
Dynlt3	0.024141575	0.92916558	0.031906887
Synb	0.029297373	0.929218195	0.031882295
Xirp2	-0.053442153	0.929218195	0.031882295
Ctrl	-0.038095985	0.929218195	0.031882295
Trpm6	-0.051555166	0.929218195	0.031882295
Itgb2l	-0.024808999	0.929218195	0.031882295
Pfkfb2	0.015708796	0.929218195	0.031882295
Dcst1	-0.068923203	0.929289514	0.031848963
Sall4	-0.085215807	0.929520557	0.031741001
Fam162a	-0.045000046	0.929520557	0.031741001
Zfp143	-0.068903436	0.929520557	0.031741001
Acvr2a	-0.057613591	0.929520557	0.031741001
Dis3	0.051091469	0.929520557	0.031741001
Barx1	0.059748327	0.929520557	0.031741001
Rnf169	0.028043972	0.929520557	0.031741001
Meiob	0.026701794	0.929520557	0.031741001
Zfp319	0.023121225	0.929520557	0.031741001
Clic3	-0.033037232	0.929520557	0.031741001

Odf2	0.035693055	0.929520557	0.031741001
Zfp655	-0.034927909	0.929520557	0.031741001
Nmd3	0.024427201	0.929520557	0.031741001
Pxk	-0.016845067	0.929520557	0.031741001
Myo5b	-0.032837944	0.929520557	0.031741001
Zfp346	0.038747864	0.929520557	0.031741001
Xrn1	-0.023150465	0.929520557	0.031741001
Akirin2	0.024834263	0.929520557	0.031741001
Vmn2r1	0.015640297	0.929520557	0.031741001
Erich4	0.029623508	0.92952542	0.031738729
Use1	0.08161095	0.92952542	0.031738729
Kdm6a	0.07577187	0.929554297	0.031725237
Pomgnt2	0.076667514	0.929554297	0.031725237
Cdkn1b	0.029606891	0.929554297	0.031725237
Slc16a13	-0.107592393	0.929554297	0.031725237
Arl2	0.038982673	0.929554297	0.031725237
Arsa	0.028609317	0.929554297	0.031725237
Nom1	-0.040644464	0.929554297	0.031725237
Pla2g4f	0.029844437	0.929554297	0.031725237
Myo7b	0.034519596	0.929554297	0.031725237
Lce1g	0.138406217	0.929554297	0.031725237
Trdmt1	-0.047534151	0.929663964	0.031674003
Kpna4	-0.024500924	0.929663964	0.031674003
Bub3	0.056942637	0.929663964	0.031674003
Alg12	0.020272718	0.929663964	0.031674003
Etv3l	0.058477392	0.929692416	0.031660712
Ring1	-0.045004068	0.929692416	0.031660712
BC051665	-0.087248455	0.929692416	0.031660712
Slc25a36	0.037444746	0.929692416	0.031660712
Arf2	0.028491914	0.929692416	0.031660712
Thoc3	-0.046617364	0.929798334	0.031611236
Defb20	0.139314072	0.929800916	0.03161003
Slc22a29	-0.044372564	0.929810839	0.031605395
Hsd3b5	-0.043215341	0.930042703	0.03149711
Spr	-0.026978695	0.930042703	0.03149711
Pggt1b	-0.03020106	0.930080547	0.031479439
TMEM254C	-0.049182498	0.930080547	0.031479439
Gdi2	-0.032767089	0.930161378	0.031441697
NOV	0.029713067	0.930161378	0.031441697
Ngly1	0.022218967	0.930161378	0.031441697
Fam219b	0.026042664	0.930298031	0.031377898
Pou3f1	0.034107835	0.930298031	0.031377898
Atp6v1g3	0.06431712	0.930391501	0.031334265
Sycp1	0.036771159	0.930391501	0.031334265
Rnf121	0.04128417	0.930391501	0.031334265
Rdh19	-0.084156272	0.930641623	0.031217528
Phyhipl	0.026345624	0.930641623	0.031217528
Atxn7l1os2	-0.046658489	0.930641623	0.031217528

Pcdh19	-0.099434943	0.930641623	0.031217528
Cfdp1	0.022040829	0.930730084	0.031176248
Prr14	-0.026600085	0.930792496	0.031147127
Saal1	-0.081065185	0.930820725	0.031133955
Scn10a	0.175788272	0.930820725	0.031133955
Tada2a	0.050325258	0.930820725	0.031133955
Rapgef6	0.067791563	0.930820725	0.031133955
Klhl2	-0.047359502	0.930820725	0.031133955
Ppm1m	-0.081584381	0.930820725	0.031133955
Fam118b	-0.054665788	0.930820725	0.031133955
Exd1	0.023319867	0.930820725	0.031133955
Atl2	-0.034509661	0.930820725	0.031133955
Pax5	0.034828355	0.930820725	0.031133955
Nfat5	-0.019596395	0.930820725	0.031133955
Tbx19	-0.040202466	0.930820725	0.031133955
Sdhd	0.047723154	0.930820725	0.031133955
Ces1f	0.017839756	0.930820725	0.031133955
Snrpd1	0.026192281	0.930820725	0.031133955
Ddx41	-0.015429813	0.930820725	0.031133955
Ric8b	-0.020485233	0.930820725	0.031133955
Xkrx	0.016789169	0.930820725	0.031133955
Cby1	0.015430924	0.930820725	0.031133955
Sigmar1	-0.066951466	0.930887039	0.031103017
Astl	0.03540656	0.930887039	0.031103017
FAM175A	-0.055876855	0.930887039	0.031103017
Zfp606	-0.015415715	0.930887039	0.031103017
Brf1	-0.058821321	0.930975475	0.031061759
Tnks2	0.095317414	0.931043289	0.031030126
Syne2	0.124753666	0.931043289	0.031030126
Wdr31	-0.109365404	0.931043289	0.031030126
Slc37a1	-0.101658681	0.931043289	0.031030126
Rdm1	0.108267931	0.931043289	0.031030126
Actl7b	-0.0692588	0.931043289	0.031030126
Rbm26	-0.059344725	0.931043289	0.031030126
Ankrd65	0.067236431	0.931043289	0.031030126
Ttn	-0.169185087	0.931043289	0.031030126
P4ha1	-0.031363534	0.931043289	0.031030126
Fbxw23	-0.032540717	0.931043289	0.031030126
Gmnc	0.038874598	0.931043289	0.031030126
Taco1	0.04663481	0.931043289	0.031030126
Ppt2	-0.026989861	0.931043289	0.031030126
Sirt7	-0.049261555	0.931043289	0.031030126
Fmo3	-0.038793326	0.931043289	0.031030126
Ppil4	0.041867314	0.931043289	0.031030126
Abhd16b	0.03305133	0.931043289	0.031030126
Slc7a14	-0.038988743	0.931043289	0.031030126
Plpp5	0.02997305	0.931043289	0.031030126
Il11ra1	-0.020076049	0.931043289	0.031030126

Zbtb22	0.02609469	0.931043289	0.031030126
E2f4	0.033490567	0.931043289	0.031030126
Mrgpra2a	0.05460711	0.931043289	0.031030126
MINOS1	0.019208278	0.931043289	0.031030126
Ptprb	0.02913579	0.931043289	0.031030126
Rnase2a	0.075207546	0.931043289	0.031030126
Akap1	-0.048300308	0.931090114	0.031008284
Tbc1d8b	0.07142793	0.931090114	0.031008284
Neb	0.057819535	0.931090114	0.031008284
Boll	-0.029367562	0.931090114	0.031008284
Thrap3	-0.075560596	0.931090114	0.031008284
Entpd8	-0.056515648	0.931300693	0.030910074
Tex35	0.041629678	0.931325101	0.030898692
Ndufa13	0.029400155	0.931325101	0.030898692
Zgrf1	0.039486549	0.931325101	0.030898692
Cpne7	-0.046976463	0.931360205	0.030882323
Ski	-0.030254467	0.931360205	0.030882323
BC089597	-0.098110613	0.931443748	0.030843368
Daam1	-0.026758611	0.931581397	0.030779192
Csf2	0.043595561	0.931581397	0.030779192
Nudcd1	-0.076350068	0.931581397	0.030779192
Sh3bgr	-0.0229233	0.931581397	0.030779192
Ccdc189	0.018703696	0.931581397	0.030779192
Snrpd2	0.030458641	0.931581397	0.030779192
Polr2b	0.03190351	0.931625106	0.030758816
P2ry4	0.070490709	0.931714561	0.030717117
Rasgrp2	-0.090214943	0.93185048	0.030653767
Adgrv1	-0.060038938	0.93185048	0.030653767
Cyp2c65	0.025687223	0.93185048	0.030653767
Gli2	0.034163403	0.931854352	0.030651962
Irf2	0.062695902	0.931854352	0.030651962
Chtf8	0.048000885	0.931854352	0.030651962
Ikbkb	0.026465971	0.931931598	0.030615963
Diaph1	0.092572137	0.932218651	0.030482212
Hnrnpa0	-0.027943007	0.932218651	0.030482212
Anks3	0.025504274	0.932316107	0.030436812
Rab26	-0.037492286	0.932316107	0.030436812
Nat8f4	-0.046105042	0.932316107	0.030436812
Ulk3	0.050933321	0.932340414	0.03042549
Fnip1	0.08046065	0.932379317	0.030407369
Ptov1	0.030660608	0.932379317	0.030407369
Capn10	0.096167775	0.932379317	0.030407369
Psmd2	-0.023178181	0.932379317	0.030407369
Mrpl21	-0.044278351	0.932475633	0.030362508
Ndufb3	0.03632001	0.932507435	0.030347697
Scfd1	0.023981688	0.932507435	0.030347697
Tprg	-0.043193812	0.932509879	0.030346558
Tti1	0.016387937	0.932509879	0.030346558

Orc2	-0.02513905	0.932509879	0.030346558
Khdc1a	0.032127665	0.932509879	0.030346558
Meikin	-0.034718233	0.932509879	0.030346558
Prss57	0.023869756	0.932509879	0.030346558
Htr4	0.024769359	0.932578477	0.030314612
Foxd2os	0.019772161	0.932578477	0.030314612
BORG	0.043767617	0.932597451	0.030305776
Ddx50	-0.033414136	0.932597451	0.030305776
HYMAI	-0.049781879	0.932597451	0.030305776
Lactb	-0.025979807	0.932597451	0.030305776
Manea	0.041162678	0.932597451	0.030305776
Taf5	0.028005787	0.932597451	0.030305776
AI506816	-0.041211906	0.932597451	0.030305776
Eif3k	0.047901619	0.932597451	0.030305776
Meis3	-0.031650239	0.932597451	0.030305776
Zfp459	-0.032552415	0.932784383	0.030218734
Steap1	-0.032421107	0.932784383	0.030218734
Asic3	-0.041502991	0.932784383	0.030218734
Cfap70	0.050265085	0.932784383	0.030218734
Stx17	-0.101765432	0.932784383	0.030218734
Fxyd4	-0.025431942	0.932879115	0.03017463
Zfp119b	-0.028033615	0.932879115	0.03017463
Lrp6	-0.024672372	0.932879115	0.03017463
Pisd	0.040955269	0.933000679	0.03011804
Ltb4r1	0.026435113	0.933000679	0.03011804
Pygo1	0.032732552	0.933005096	0.030115984
Gtf3a	0.033921515	0.933005096	0.030115984
Asb7	-0.035889224	0.933005096	0.030115984
Zfp764	-0.031020267	0.933005096	0.030115984
Lrrn4	-0.060321999	0.933048568	0.030095749
Zfp566	0.039692279	0.93305981	0.030090517
Anapc7	0.029973163	0.93305981	0.030090517
Mylk4	-0.144988841	0.933131942	0.030056944
Tmsb15l	0.046728289	0.933131942	0.030056944
Ubl3	-0.037200594	0.933146594	0.030050125
ISPD	0.021397428	0.933146594	0.030050125
Bcl6	0.047271994	0.933146594	0.030050125
Gatc	0.035128688	0.933146594	0.030050125
Mtpap	0.032821521	0.93328801	0.029984313
Zfp82	0.021850787	0.933331007	0.029964306
Atf7	0.034152344	0.933387331	0.029938098
Spata33	0.039339614	0.933387331	0.029938098
Cox10	-0.04906515	0.933387331	0.029938098
Fam91a1	0.040348616	0.933387331	0.029938098
Dnajb12	-0.068027989	0.933792591	0.029749576
Abcb10	-0.023303836	0.933792591	0.029749576
Zxdc	0.038102702	0.933792591	0.029749576
Pdcd5	-0.069404492	0.933792591	0.029749576

Vangl2	0.030350644	0.933855046	0.02972053
Gosr1	-0.046329497	0.933855046	0.02972053
Eif2b5	0.038877038	0.933855046	0.02972053
Htr3b	0.030606261	0.933855046	0.02972053
Dcun1d5	0.025231057	0.933855046	0.02972053
Srxn1	0.070136438	0.933855046	0.02972053
Mfsd4b2	0.037629575	0.933855046	0.02972053
Ift80	0.066740614	0.933855046	0.02972053
Rnf138rt1	0.029156199	0.933855046	0.02972053
Spaca4	0.088444704	0.934004786	0.029650898
Cnot1	0.035423754	0.934004786	0.029650898
Mpg	0.033754821	0.934004786	0.029650898
Jup	-0.030625735	0.934004786	0.029650898
Xlr5b	-0.049561468	0.934004786	0.029650898
Prr3	-0.033733656	0.934004786	0.029650898
Cphx1	-0.020806514	0.934004786	0.029650898
Hnrnpa1	-0.052948581	0.934103871	0.029604828
Tpd52l2	-0.024840992	0.934103871	0.029604828
Smr3a	0.037891885	0.934103871	0.029604828
Ccdc50	-0.019429817	0.934103871	0.029604828
Sh3tc2	-0.031094741	0.934103871	0.029604828
Ibtk	-0.030706087	0.934103871	0.029604828
Zfp131	-0.016225116	0.934103871	0.029604828
Mrpl49	0.033825828	0.934137365	0.029589256
Tg	0.102079323	0.934159166	0.029579121
Rif1	0.022099009	0.934159166	0.029579121
Khdc1b	-0.086678995	0.934198346	0.029560906
Cryaa	0.023249831	0.934198346	0.029560906
Lrrc14	0.085050456	0.934224524	0.029548736
Rbms3	-0.03603706	0.934305895	0.029510911
Ldb1	-0.032630826	0.934658998	0.029346809
Uchl1os	-0.040302471	0.9347956	0.029283341
Zkscan7	-0.115564722	0.9347956	0.029283341
Zscan2	0.028774841	0.9347956	0.029283341
Usp12	-0.024182506	0.9347956	0.029283341
Prdx3	0.030432894	0.934918213	0.02922638
Gfm2	0.050324532	0.934918213	0.02922638
Papola	0.090161826	0.934918213	0.02922638
Ampd1	-0.028224382	0.934918213	0.02922638
Tmem214	0.022183253	0.93492511	0.029223176
Actr3	0.058459827	0.935039015	0.029170267
Eif3i	-0.100493454	0.935039015	0.029170267
Bbox1	0.019361526	0.935176946	0.029106208
Enpp2	-0.050058222	0.935372699	0.02901531
Gkn1	0.031523297	0.935372699	0.02901531
Acat3	-0.049699888	0.935410237	0.028997881
Gpr52	-0.040401694	0.935410237	0.028997881
Palmd	0.043901236	0.935410237	0.028997881

TMEM194E	0.028020796	0.935410237	0.028997881
Cdc14a	0.029124215	0.935410237	0.028997881
Swt1	0.029700697	0.935410237	0.028997881
Wipf3	-0.026679008	0.935410237	0.028997881
Rbbp9	-0.051772919	0.935410237	0.028997881
Gtpbp1	0.01895408	0.935410237	0.028997881
Tia1	-0.016049039	0.935410237	0.028997881
Gys1	-0.03563011	0.935458028	0.028975694
Morn1	-0.060725763	0.935491586	0.028960114
Wdr44	-0.080694947	0.935491586	0.028960114
Slc17a5	0.024107841	0.935491586	0.028960114
Kdm5a	0.014773195	0.935516227	0.028948675
Ccng1	-0.06436433	0.935685867	0.02886993
Slc6a19os	-0.025726832	0.935685867	0.02886993
Grik5	-0.040744667	0.935695251	0.028865575
Gcm2	-0.077602253	0.935740079	0.028844769
Nthl1	-0.042496419	0.935863998	0.028787259
Dnajc8	0.014742576	0.935943899	0.028750182
Ttll2	-0.041417627	0.935989001	0.028729255
Pwwp2a	-0.042515053	0.935989001	0.028729255
Fam162b	0.063201573	0.935989001	0.028729255
Efcab6	-0.021349046	0.935989001	0.028729255
Zfp663	-0.029527739	0.935989001	0.028729255
Phlda1	-0.057184116	0.936511552	0.028486861
Nsfl1c	-0.082928176	0.936511552	0.028486861
Taf7	-0.031569813	0.936511552	0.028486861
Kis2	-0.024259271	0.936511552	0.028486861
Afm	-0.049690882	0.936511552	0.028486861
Impg1	0.022185296	0.936511552	0.028486861
Efna2	-0.052598164	0.936511552	0.028486861
Frmpd1	0.026315236	0.936511552	0.028486861
Dnah8	-0.032457524	0.936511552	0.028486861
Asic5	-0.014681896	0.936511552	0.028486861
Ctcflos	-0.025688649	0.936511552	0.028486861
Dennd1b	0.035518192	0.936553819	0.028467261
Gpihbp1	-0.043916981	0.936553819	0.028467261
Vhl	-0.061540574	0.936638546	0.028427974
Pdgfa	0.041084693	0.936638546	0.028427974
Txnrd3	0.031777486	0.936638546	0.028427974
Pdia6	0.045977841	0.936638546	0.028427974
Zfp169	0.028837662	0.936638546	0.028427974
Ormdl3	-0.022737443	0.936638546	0.028427974
Mep1b	-0.018154151	0.936638546	0.028427974
TCEB2	0.071924073	0.936638546	0.028427974
Snf8	0.220115965	0.936638546	0.028427974
Pskh1	-0.038128581	0.936638546	0.028427974
Wdr54	0.041775541	0.936638546	0.028427974
Myef2	-0.070498604	0.936725123	0.028387832

Secisbp2	-0.025290088	0.936817866	0.028344836
Dnajc7	0.025924862	0.936947062	0.028284946
Trps1	0.062758666	0.936990467	0.028264828
Gigyf1	-0.084513711	0.937049526	0.028237455
Cartpt	-0.042467298	0.937049526	0.028237455
Tpgs1	0.028561825	0.937049526	0.028237455
Klc4	-0.020950536	0.937049526	0.028237455
Nenf	0.042756407	0.937049526	0.028237455
Cdc27	0.025576608	0.937247889	0.028145529
Kcnn3	0.025763214	0.93728103	0.028130173
Heph	-0.043691764	0.93728103	0.028130173
Syt6	-0.014584351	0.93728103	0.028130173
Stk31	0.025901628	0.93728103	0.028130173
Chil4	-0.030204338	0.937334111	0.028105578
Zfp422	0.030113049	0.937334111	0.028105578
Grin3b	0.022716013	0.937334111	0.028105578
Lrrc8c	-0.016012431	0.937334111	0.028105578
Olfr523	0.024353764	0.937443329	0.028054977
Cyp2d22	-0.019485173	0.937443329	0.028054977
Thoc1	0.029953219	0.937654359	0.027957223
Otop1	0.024027587	0.937659159	0.027955
Col6a6	0.149048109	0.937675592	0.027947389
Eif2b2	0.04059012	0.937675592	0.027947389
Tmem41a	-0.015612404	0.937675592	0.027947389
Pelp1	0.064836194	0.937874437	0.027855301
Cdc25a	0.178484952	0.938375458	0.027623359
Mier2	-0.046490825	0.938641186	0.027500394
Msmp	0.040601118	0.938641186	0.027500394
Snrpa1	-0.029392727	0.938641186	0.027500394
Vrtn	-0.033262093	0.938641186	0.027500394
Lyzl4os	-0.036399966	0.938641186	0.027500394
Abhd2	0.037235767	0.938641186	0.027500394
Rsbn1	0.04946088	0.938641186	0.027500394
Dnah10	-0.032235622	0.938641186	0.027500394
Mpped2	0.03082074	0.938641186	0.027500394
DNAIC1	0.059373857	0.938641186	0.027500394
Bcorl1	0.0632771	0.938641186	0.027500394
Slc7a9	-0.050808344	0.938641186	0.027500394
Tmem234	0.026534922	0.938641186	0.027500394
Cpb2	0.026922921	0.938641186	0.027500394
Cpe	-0.0237676	0.938641186	0.027500394
Olfr70	0.042119501	0.938641186	0.027500394
Tlk2	-0.022467788	0.938641186	0.027500394
Rbm19	-0.018375129	0.938641186	0.027500394
Klk10	-0.039218424	0.938641186	0.027500394
Sowahd	-0.024615601	0.938641186	0.027500394
Eid2	-0.019648445	0.938641186	0.027500394
Eif3b	-0.042223622	0.938641186	0.027500394

C8a	-0.031796999	0.938641186	0.027500394
Donson	0.08652569	0.938641186	0.027500394
Snapc2	0.048044553	0.938641186	0.027500394
Krtap13-1	0.040859095	0.938641186	0.027500394
BB123696	-0.015665792	0.938641186	0.027500394
Rnasek	-0.032267112	0.938641186	0.027500394
Atp5h	0.020531873	0.938641186	0.027500394
Setd1b	-0.042128271	0.938641186	0.027500394
Clasrp	0.025206805	0.938641186	0.027500394
Ppp1ca	0.058162616	0.938641186	0.027500394
Fer1l5	-0.030596364	0.938777445	0.027437353
Saxo2	-0.02657428	0.938777445	0.027437353
Esco1	0.029116627	0.938777445	0.027437353
TMEM110	0.020760643	0.938866829	0.027396004
Sh2d2a	-0.041027847	0.938890312	0.027385142
Tmem175	-0.075301078	0.938934026	0.027364922
Uevld	-0.036507989	0.93954856	0.027080769
Vmn1r235	0.027948566	0.93954856	0.027080769
Zfp275	-0.033433074	0.939815216	0.026957528
Rccd1	0.096896483	0.939815216	0.026957528
Catsperg1	0.025899402	0.939815216	0.026957528
Rufy1	0.035881748	0.939815216	0.026957528
Pcdha7	0.024885242	0.939815216	0.026957528
Ube2i	0.024667673	0.939815216	0.026957528
Sftpb	-0.029157283	0.939815216	0.026957528
Rnf222	0.208740506	0.939815216	0.026957528
Atxn3	0.024636806	0.939815216	0.026957528
Ankrd53	0.019951029	0.939815216	0.026957528
Tbx10	0.058350346	0.939842724	0.026944816
Slc51a	0.024589886	0.939968718	0.0268866
Adamtsl5	0.031427553	0.939970383	0.02688583
Wdr75	0.088280556	0.940093006	0.026829178
Meox2	-0.054081335	0.940093006	0.026829178
Cs	-0.035733303	0.940093006	0.026829178
Gga2	-0.032998338	0.940093006	0.026829178
Angptl1	0.028817184	0.940093006	0.026829178
Prdm4	-0.038920503	0.940093006	0.026829178
Prdm9	-0.030725648	0.940093006	0.026829178
Tpd52l1	-0.019587047	0.940093006	0.026829178
Gpr173	0.031052023	0.940093006	0.026829178
Dgat2	0.038871218	0.940093006	0.026829178
Cul5	-0.058864923	0.940163923	0.026796418
Drap1	0.050323875	0.940163923	0.026796418
Ldha	0.060879641	0.940163923	0.026796418
Arvcf	0.023187348	0.940163923	0.026796418
Arpin	-0.025745047	0.940163923	0.026796418
Platr3	-0.030480792	0.940163923	0.026796418
Ddi1	0.10666494	0.940163923	0.026796418

Mrpl53	0.056284548	0.940163923	0.026796418
Barhl1	-0.029694408	0.940193981	0.026782533
Tanc1	-0.097268584	0.940193981	0.026782533
Hyal1	0.043440787	0.940193981	0.026782533
Slc39a9	0.03097382	0.940193981	0.026782533
Plac8l1	-0.086556827	0.940193981	0.026782533
Fdx1	-0.058458385	0.940287347	0.026739408
Map7d1	0.028737357	0.940287347	0.026739408
Rpl21	0.041202703	0.940287347	0.026739408
Clrn2	0.015025569	0.940287347	0.026739408
Ldhc	0.031792037	0.940287347	0.026739408
Gnao1	-0.190705669	0.940593635	0.026597965
Nexn	-0.036358822	0.940639522	0.026576778
Atf6b	0.021615929	0.940639522	0.026576778
Fut11	-0.030184256	0.940639522	0.026576778
Med16	0.072356119	0.940639522	0.026576778
OIT1	-0.035129677	0.940829321	0.026489156
Nat8f2	-0.019181755	0.940829321	0.026489156
Usp48	0.027319831	0.940829321	0.026489156
Gin1	0.08688175	0.940846379	0.026481282
BC024978	0.035601037	0.940846379	0.026481282
Duoxa2	0.07897578	0.940846379	0.026481282
Dstn	-0.085564574	0.940885693	0.026463135
Sec22a	-0.073512297	0.940885693	0.026463135
Pecr	0.055198351	0.940885693	0.026463135
Faap100	-0.140242495	0.940885693	0.026463135
Mief1	0.043608352	0.940885693	0.026463135
Eef2k	-0.029443372	0.940885693	0.026463135
Fcamr	0.053023882	0.940885693	0.026463135
Aanat	0.033851177	0.940885693	0.026463135
Rwdd3	-0.074562851	0.940885693	0.026463135
Zfp943	-0.028357137	0.940885693	0.026463135
Spint2	-0.051500009	0.940885693	0.026463135
Eif3f	0.040775708	0.940885693	0.026463135
Ifnb1	-0.038142491	0.940885693	0.026463135
Matn3	0.065643486	0.940885693	0.026463135
Casp9	-0.050939799	0.940885693	0.026463135
Bambi	0.025341871	0.940885693	0.026463135
Cd177	0.059635734	0.940885693	0.026463135
Amy2a4	0.031601665	0.940885693	0.026463135
Zfp688	-0.026077817	0.940885693	0.026463135
Ddi2	0.028464842	0.940885693	0.026463135
Ikzf3	0.051493106	0.940885693	0.026463135
St6galnac	0.029623931	0.940885693	0.026463135
Mto1	0.01547163	0.940885693	0.026463135
Trpv1	0.035552152	0.940885693	0.026463135
Med27	0.027940221	0.940925553	0.026444737
Clk2	-0.026888792	0.941028528	0.02639721

Tha1	-0.066240334	0.941067011	0.026379451
Cdc34	0.055510869	0.941104764	0.026362028
Snrpf	-0.025002329	0.941104764	0.026362028
Gmds	-0.026459485	0.941104764	0.026362028
Mir9-3hg	-0.067510927	0.941142623	0.026344558
Cutc	0.075064458	0.941258124	0.026291262
Fbxo42	0.083664476	0.941258124	0.026291262
Lcmt1	0.022302104	0.941258124	0.026291262
Csnka2ip	0.01511268	0.941258124	0.026291262
Bcl2l14	0.023183737	0.941390437	0.026230218
Brpf3	0.116131596	0.941543348	0.02615968
Bdh2	-0.035270435	0.94156647	0.026149016
Gart	0.035764373	0.94156647	0.026149016
Eea1	0.062484946	0.94156647	0.026149016
Xpo4	-0.030256766	0.94156647	0.026149016
Khnyn	-0.054418799	0.94156647	0.026149016
Fbxl3	-0.026545804	0.94156647	0.026149016
Ttc41	0.042522469	0.94156647	0.026149016
Acbd5	0.076206672	0.94156647	0.026149016
Larp4b	-0.027058686	0.94156647	0.026149016
Mrps10	0.024738361	0.94156647	0.026149016
Zfp599	-0.016464594	0.94156647	0.026149016
Mtrr	-0.036433904	0.94156647	0.026149016
Ttc24	0.039439543	0.94156647	0.026149016
Phka1	0.054737252	0.94156647	0.026149016
UTP11L	-0.016856384	0.94156647	0.026149016
Rbm6	0.014052013	0.94156647	0.026149016
Rhov	0.030620121	0.941570589	0.026147115
Ovol3	-0.035052683	0.941639255	0.026115445
Riiad1	0.04568715	0.941639255	0.026115445
Cnr1	0.031694128	0.941639255	0.026115445
Aasdhppt	0.046524821	0.941639255	0.026115445
Bmp2	0.019444204	0.941639255	0.026115445
Arrdc2	0.030585824	0.941639255	0.026115445
Poll	-0.019428861	0.941639255	0.026115445
Il23r	0.034706384	0.941639255	0.026115445
Gtf2f2	-0.028243834	0.941639255	0.026115445
Tmem94	0.062348952	0.941639255	0.026115445
BC031181	0.030740053	0.941639255	0.026115445
Parn	0.024346968	0.941639255	0.026115445
Vmn2r54	-0.025658615	0.941774406	0.026053116
Slc32a1	0.024306219	0.941774406	0.026053116
Mocs2	-0.030827728	0.941774406	0.026053116
Slc35g1	-0.051503798	0.941774406	0.026053116
Nodal	-0.073741752	0.941774406	0.026053116
Lrp8os2	0.024086281	0.941774406	0.026053116
Rbx1	-0.061458892	0.941918155	0.025986832
Heatr6	-0.081251398	0.941918155	0.025986832

Yeats4	-0.058317414	0.941918155	0.025986832
D11Wsu47	-0.076206524	0.941918155	0.025986832
Dchs1	0.098373275	0.941918155	0.025986832
Wfdc12	0.264449603	0.941918155	0.025986832
Cab39l	0.026282577	0.941918155	0.025986832
Capn1	0.045005518	0.941918155	0.025986832
Sart1	-0.051928182	0.941918155	0.025986832
Ces4a	-0.053998278	0.941918155	0.025986832
Hpse2	-0.063066883	0.941918155	0.025986832
Kptn	0.063375014	0.941918155	0.025986832
Ruvbl2	-0.03655095	0.941918155	0.025986832
Sost	0.03673476	0.941918155	0.025986832
RNF8-CMT	0.027745656	0.941918155	0.025986832
Smarcad1	-0.084893754	0.941918155	0.025986832
Rps6kl1	-0.045113473	0.941918155	0.025986832
Rab36	0.061310298	0.941918155	0.025986832
Foxr2	0.038974833	0.941918155	0.025986832
Adgrf5	0.032692919	0.941918155	0.025986832
Lrch2	0.042993682	0.941918155	0.025986832
Lep	-0.029154508	0.941918155	0.025986832
Fbxo32	-0.052900223	0.941918155	0.025986832
Pdzk1	-0.058216457	0.941918155	0.025986832
Tmem30c	-0.018752571	0.941918155	0.025986832
Mettl15	0.078946424	0.941918155	0.025986832
Rcan3	0.029741553	0.941918155	0.025986832
Oas1e	0.024843097	0.941918155	0.025986832
Pcdh10	0.042468685	0.941918155	0.025986832
Klrb1f	0.02733248	0.941918155	0.025986832
Wwp2	0.06937432	0.941918155	0.025986832
Irf3	-0.019529197	0.941918155	0.025986832
Slco5a1	-0.045194986	0.941918155	0.025986832
Dtx2	-0.041950918	0.941918155	0.025986832
Jade3	0.02480881	0.941918155	0.025986832
Gprc5d	-0.020524338	0.941918155	0.025986832
Eml1	-0.028479322	0.941918155	0.025986832
Ttc29	0.050847565	0.941918155	0.025986832
Anapc4	-0.049186659	0.941918155	0.025986832
Kctd21	0.018230237	0.941918155	0.025986832
Trcg1	0.026531826	0.941918155	0.025986832
Pdcl	0.027604598	0.941918155	0.025986832
Tra2a	0.025242196	0.941918155	0.025986832
Mrps9	0.043162417	0.941918155	0.025986832
Fxr1	-0.021394839	0.941918155	0.025986832
Rps6kb1	-0.061224034	0.941918155	0.025986832
Morc2a	0.029496347	0.941918155	0.025986832
Dck	0.023070643	0.941918155	0.025986832
Ssxb5	-0.083734919	0.941918155	0.025986832
Rfpl4	0.022550818	0.941918155	0.025986832

Gpx4	0.039626125	0.941918155	0.025986832
Olfr1342	0.034567945	0.941918155	0.025986832
Dcdc5	-0.022426202	0.941918155	0.025986832
Med25	0.022445432	0.941918155	0.025986832
Mapkap1	-0.025228771	0.941918155	0.025986832
Eci1	0.032340783	0.941918155	0.025986832
Lars	-0.034852782	0.941918155	0.025986832
Rad54l2	-0.045851612	0.941918155	0.025986832
Abl2	-0.017562672	0.941918155	0.025986832
Slc25a28	0.024013741	0.941918155	0.025986832
Rap1gapo:	0.036267187	0.941918155	0.025986832
Taf15	-0.028272509	0.941918155	0.025986832
Slc34a1	0.028054965	0.941918155	0.025986832
Gje1	0.035409158	0.941918155	0.025986832
Agbl3	0.025043336	0.941918155	0.025986832
Ndst1	-0.031995371	0.941918155	0.025986832
Prcc	0.026935557	0.941918155	0.025986832
HIST1H1B	0.037417691	0.941918155	0.025986832
Dmp1	-0.045607461	0.941918155	0.025986832
Bfar	0.041721936	0.941918155	0.025986832
ZNRD1	-0.049886156	0.941918155	0.025986832
Fam53a	0.029103579	0.941918155	0.025986832
Plscr5	-0.025810173	0.941918155	0.025986832
Foxa2	0.054572146	0.941918155	0.025986832
Dnah17	0.062947561	0.941918155	0.025986832
Slc9a1	-0.035324237	0.941918155	0.025986832
Psmd1	-0.053118716	0.941918155	0.025986832
Hoxb5	-0.043289945	0.941918155	0.025986832
Gorasp2	0.048661951	0.941918155	0.025986832
Abcd2	-0.036204671	0.941918155	0.025986832
Tex26	0.048095203	0.941918155	0.025986832
Prmt6	-0.047231547	0.941918155	0.025986832
Zdhhc11	-0.048590211	0.941918155	0.025986832
Fhdc1	0.024387126	0.941918155	0.025986832
Timd2	-0.0372748	0.941918155	0.025986832
BC022687	0.057559423	0.941918155	0.025986832
Ptms	0.086675193	0.941918155	0.025986832
Lmod1	0.019475462	0.941918155	0.025986832
FAM188B	0.026063212	0.941918155	0.025986832
Fam71a	-0.020434428	0.941918155	0.025986832
Lgsn	0.02759322	0.941918155	0.025986832
Gk	-0.04023278	0.941918155	0.025986832
Arel1	0.03373335	0.941918155	0.025986832
Tmem92	-0.023707635	0.941918155	0.025986832
Nup188	-0.023700245	0.941918155	0.025986832
Mef2a	0.026397348	0.941918155	0.025986832
Prss2	0.02079697	0.941918155	0.025986832
Mterf1a	-0.033791579	0.941918155	0.025986832

Srrm1	0.02496463	0.941918155	0.025986832
Ier3ip1	-0.027513704	0.941918155	0.025986832
Cirbp	0.030275607	0.941918155	0.025986832
Tbl1x	0.026486817	0.941918155	0.025986832
TMEM5	0.050687375	0.941918155	0.025986832
Kcnv2	-0.067142417	0.941918155	0.025986832
Gp9	0.050599985	0.941918155	0.025986832
Nrarp	-0.031062499	0.941918155	0.025986832
Edc3	0.03553783	0.941918155	0.025986832
Fcgbp	0.022875781	0.941918155	0.025986832
Dnajc14	0.054171433	0.941918155	0.025986832
Rbm4b	0.05174284	0.941918155	0.025986832
Tmem184	0.018472013	0.941918155	0.025986832
Actr5	0.054796613	0.941918155	0.025986832
Naa11	0.030690984	0.941918155	0.025986832
Krtap2-4	0.029246486	0.941918155	0.025986832
Tma7	-0.041086452	0.941918155	0.025986832
Samd4b	0.032279576	0.941918155	0.025986832
Rorc	0.019083591	0.941918155	0.025986832
Npy4r	-0.013915001	0.941918155	0.025986832
XRCC6BP1	0.048034523	0.941918155	0.025986832
Bbx	0.014415591	0.941918155	0.025986832
Mia2	0.025981512	0.941918155	0.025986832
Plrg1	0.08439763	0.941918155	0.025986832
Fbxl15	-0.023597337	0.941918155	0.025986832
Chchd1	0.021765253	0.941918155	0.025986832
Tll1	0.066147374	0.941918155	0.025986832
Havcr1	0.090210783	0.941918155	0.025986832
Gprin3	-0.023026797	0.941918155	0.025986832
Serpina1e	0.024253532	0.941918155	0.025986832
Pold3	-0.039874549	0.941918155	0.025986832
Irgc1	0.045149086	0.941918155	0.025986832
Fasn	-0.031916883	0.941918155	0.025986832
Ttll13	0.037577169	0.941918155	0.025986832
Txndc2	-0.030901789	0.941918155	0.025986832
Ltv1	-0.027748684	0.941918155	0.025986832
MUT	0.028930839	0.941918155	0.025986832
Pcdh8	-0.030676393	0.941918155	0.025986832
Wdr88	-0.031094445	0.941918155	0.025986832
Exoc6	-0.013735435	0.941918155	0.025986832
Lmln	-0.013686267	0.941918155	0.025986832
Nrtn	-0.030273244	0.941918155	0.025986832
Slirp	0.046721947	0.941918155	0.025986832
Snrpa	0.027295727	0.941918155	0.025986832
Nppa	-0.04652614	0.941918155	0.025986832
Cnpy1	0.019819811	0.942058397	0.025922175
Ccar1	-0.024253079	0.942184121	0.025864219
Pank3	0.029037785	0.942184121	0.025864219

Tmem88	0.055357644	0.942459204	0.02573744
Slc35a4	-0.07565972	0.942459204	0.02573744
CN725425	-0.022743693	0.942459204	0.02573744
Srp9	0.044390048	0.942593395	0.025675608
Hmx2	-0.050769734	0.942593395	0.025675608
Esrp1	-0.070960076	0.942593395	0.025675608
Serpina5	-0.030150675	0.942593395	0.025675608
Luc7l2	-0.029175101	0.942593395	0.025675608
Vangl1	-0.047508473	0.942593395	0.025675608
Etv4	0.037850562	0.942593395	0.025675608
Pcdhga1	-0.02720175	0.942593395	0.025675608
Ptar1	0.039988722	0.942593395	0.025675608
Ms4a8a	-0.023083187	0.942670394	0.025640132
HIST1H2BE	-0.03763857	0.942670394	0.025640132
Adcyap1r1	-0.023994372	0.942670394	0.025640132
Taar4	0.029339905	0.942718624	0.025617913
AI464131	-0.054299737	0.942718624	0.025617913
Mad2l1bp	0.06460581	0.942718624	0.025617913
Tmem87a	-0.033901394	0.942718624	0.025617913
Gcsam	0.014167926	0.942718624	0.025617913
Hoxa4	-0.03211708	0.942782095	0.025588674
Rundc1	0.033199435	0.942782095	0.025588674
Cpox	-0.025826915	0.94282008	0.025571176
Tomm22	-0.041463366	0.94293359	0.025518893
Mrps21	-0.032548254	0.94293359	0.025518893
Tmem260	0.028206257	0.94293359	0.025518893
Oas1d	-0.130351026	0.943047811	0.025466289
Armc10	0.039188717	0.943047811	0.025466289
Rpl3	0.033172655	0.943047811	0.025466289
Iqch	-0.028486901	0.943047811	0.025466289
Foxj2	-0.016749269	0.943047811	0.025466289
Lcor	-0.034751905	0.943047811	0.025466289
Psmd6	0.033765468	0.943047811	0.025466289
Sf3b4	-0.04151383	0.943111008	0.025437186
HIST3H2BA	0.064262428	0.943111008	0.025437186
AU019990	-0.039521395	0.943111008	0.025437186
Snx3	0.024310941	0.943111008	0.025437186
Tbl3	0.033044101	0.943111008	0.025437186
Myo7a	0.138189025	0.943111008	0.025437186
Abcf1	-0.062642529	0.943111008	0.025437186
Dnajb9	-0.029370345	0.943111008	0.025437186
Ndrg2	0.021895994	0.943111008	0.025437186
Stpg2	0.018406592	0.943111008	0.025437186
Tomm5	-0.022013223	0.943111008	0.025437186
Stam2	-0.018203672	0.943111008	0.025437186
Klra1	0.071168182	0.943111008	0.025437186
AI597479	-0.026282814	0.943111008	0.025437186
Imp3	-0.031561815	0.943137935	0.025424787

Nr1i2	0.066826568	0.943137935	0.025424787
Mrps17	-0.050887537	0.943140169	0.025423758
Rps6ka6	0.026640119	0.943140169	0.025423758
Ptpn11	0.078867228	0.943140169	0.025423758
Sox15	-0.026715307	0.943140169	0.025423758
Uggt1	-0.030610481	0.943196118	0.025397995
Eppk1	-0.04631923	0.943243596	0.025376135
Tubb1	0.040245417	0.943273311	0.025362453
Ptpn20	-0.040756738	0.943302433	0.025349045
Cacna2d2	-0.045033588	0.943412848	0.025298213
Timm22	0.028933146	0.943460999	0.025276048
Dad1	0.033597592	0.94356919	0.025226248
Cnbp	-0.013373449	0.94356919	0.025226248
Vsx1	0.043408996	0.943657761	0.025185484
Zswim1	0.027238066	0.943767086	0.025135173
Mrgpre	-0.063084165	0.943767086	0.025135173
C1d	0.017347024	0.943767086	0.025135173
Samd5	-0.063893155	0.943804228	0.025118081
Anapc10	-0.033668634	0.943804228	0.025118081
Mgat5	0.043315977	0.943812587	0.025114235
Doc2b	-0.043934748	0.943812587	0.025114235
Bccip	0.044139478	0.943812587	0.025114235
Pacsin3	0.05223168	0.943841633	0.02510087
Aatf	-0.013336452	0.943841633	0.02510087
Uckl1os	0.031815426	0.943944684	0.025053455
Htatsf1	-0.052233342	0.944011393	0.025022764
HIST1H2AI	-0.031123388	0.94403288	0.025012879
Ccdc138	0.075584719	0.94403288	0.025012879
Odf2l	0.030768758	0.94403288	0.025012879
HILS1	-0.019210997	0.944117199	0.024974091
Eral1	-0.037423025	0.944168012	0.024950717
Mc1r	0.033159708	0.944629536	0.024738479
Cyth3	0.059749111	0.944629536	0.024738479
EFCAB13	0.02701508	0.944629536	0.024738479
Efnb3	0.060769183	0.944629536	0.024738479
Ddx4	0.097995508	0.944629536	0.024738479
Trim72	0.014160629	0.944629536	0.024738479
Anxa13	-0.024124643	0.944629536	0.024738479
Phrf1	0.041340363	0.944629536	0.024738479
Cacna1h	0.074402719	0.944629536	0.024738479
Tubd1	-0.048154495	0.944629536	0.024738479
Sh3rf2	-0.024338038	0.944629536	0.024738479
Ccz1	0.025191357	0.944797249	0.02466138
Trmt2a	-0.068431218	0.944845902	0.024639016
Khdc1c	-0.031561729	0.944845902	0.024639016
Trim29	0.031478913	0.944845902	0.024639016
Fshr	0.015678985	0.944845902	0.024639016
Zbtb25	-0.019872063	0.944845902	0.024639016

Galp	-0.033308163	0.944845902	0.024639016
Dlx6os2	0.014806135	0.944845902	0.024639016
Fam76a	0.01739401	0.944845902	0.024639016
Nupl2	0.051008504	0.94488363	0.024621675
Sf3b1	0.021992694	0.944940086	0.024595727
Cabp7	-0.035591948	0.944971587	0.02458125
Klhl21	-0.032529438	0.944982206	0.024576369
Rps15	-0.030616341	0.944982206	0.024576369
Arhgap5	0.026979055	0.944982206	0.024576369
Tab1	-0.036368166	0.945038511	0.024550493
Zeb1	-0.027064542	0.945038511	0.024550493
Lamtor5	-0.038769709	0.945147984	0.024500188
CCDC64B	0.021451814	0.945147984	0.024500188
Psca	0.013330348	0.945147984	0.024500188
A3galt2	-0.036815747	0.945150392	0.024499081
Hoxa1	-0.032911552	0.945150392	0.024499081
Tnfrsf9	0.067659409	0.945150392	0.024499081
Glra2	0.04229199	0.945150392	0.024499081
Ing5	0.017262762	0.945150392	0.024499081
Cherp	-0.02293182	0.945150392	0.024499081
Mtss1	-0.033005833	0.945218391	0.024467837
Trap1	-0.042576747	0.945218391	0.024467837
Pdlim7	-0.072918993	0.945361226	0.024402214
Ccdc178	-0.039212136	0.945361226	0.024402214
Gsta1	-0.034427028	0.945361226	0.024402214
Twf2	-0.016864912	0.945361226	0.024402214
Gins3	0.029678607	0.945361226	0.024402214
Gtsf1	-0.056040635	0.945361226	0.024402214
Ric1	0.026600606	0.945361226	0.024402214
Lpin1	0.017592632	0.945361226	0.024402214
Odf1	0.020420077	0.945361226	0.024402214
Scn5a	-0.036235229	0.945361226	0.024402214
Srd5a2	0.023480004	0.945361226	0.024402214
Zfp362	-0.020213256	0.945361226	0.024402214
Sirt5	0.019819314	0.945361226	0.024402214
Ecd	-0.013937113	0.945361226	0.024402214
Slc39a7	0.034487957	0.945361226	0.024402214
Pcdhgb6	-0.013127632	0.945361226	0.024402214
Nphp1	-0.073928221	0.945361226	0.024402214
Ckap5	-0.065073494	0.945379273	0.024393924
Xpo1	0.015105564	0.945379273	0.024393924
Klhdc7a	0.019154391	0.945379273	0.024393924
Scap	0.034203705	0.945386042	0.024390814
Phkg2	0.073422593	0.945386042	0.024390814
Pigf	0.026113899	0.945386042	0.024390814
Olfml2a	-0.026845282	0.945386042	0.024390814
Gpr37	0.03503674	0.945516883	0.024330712
Ap2a2	-0.040936443	0.945516883	0.024330712

Gnptab	-0.043420661	0.945581247	0.02430115
Cxcr1	-0.021421751	0.945581247	0.02430115
Lrtm1	-0.021206765	0.945581247	0.02430115
Taf9	0.028224958	0.945581247	0.02430115
Whamm	-0.031158931	0.9455918	0.024296302
Stag1	-0.056158871	0.945616322	0.02428504
Ctu2	-0.029163797	0.945616322	0.02428504
Kcnk15	-0.025727301	0.945632417	0.024277648
Cks1brt	-0.017558023	0.945632417	0.024277648
Pgm3	-0.020972137	0.945632417	0.024277648
Dqx1	-0.024089413	0.945682835	0.024254494
Cetn3	-0.057838855	0.945690575	0.024250939
Clec2g	0.023378041	0.945690575	0.024250939
Cct5	0.030578387	0.945690575	0.024250939
Mmp15	-0.053828477	0.945690575	0.024250939
Rbm8a	0.031214945	0.945690575	0.024250939
Mamdc4	0.056230466	0.945730548	0.024232583
Tex15	0.027253328	0.946113543	0.024056741
Upk2	-0.036989111	0.946113543	0.024056741
Angptl8	0.022746721	0.946113543	0.024056741
BC051537	0.024532564	0.946113543	0.024056741
Junos	0.033736712	0.946113543	0.024056741
Rab22a	0.035748055	0.946113543	0.024056741
Lypla2	0.023552254	0.946113543	0.024056741
Rnf103	0.018662253	0.946113543	0.024056741
Tbc1d10b	-0.017051366	0.946113543	0.024056741
Psmd7	-0.041358516	0.946113543	0.024056741
Arl14ep	-0.021922042	0.946229167	0.024003669
Ino80e	-0.023814293	0.946229167	0.024003669
Cpt1c	-0.017686862	0.946229167	0.024003669
Utp20	0.01459649	0.946229167	0.024003669
Afap1	-0.043439672	0.946229167	0.024003669
Ncl	-0.012746144	0.946229167	0.024003669
Chrna2	-0.078153991	0.946328731	0.023957974
Pkig	0.145904791	0.946328731	0.023957974
Paf1	0.047197167	0.946360233	0.023943517
Prdm13	0.090964213	0.946547466	0.023857603
Ptpdc1	0.032170923	0.946547466	0.023857603
Neurog3	0.033979899	0.946547466	0.023857603
Hipk2	0.057179677	0.946547466	0.023857603
Shtn1	-0.038440399	0.946547466	0.023857603
Copz1	0.058234856	0.946547466	0.023857603
Vps54	-0.027800497	0.946547466	0.023857603
Klk1b24	-0.024978842	0.946547466	0.023857603
Colec10	-0.021971037	0.946547466	0.023857603
Dtwd1	0.026738719	0.946547466	0.023857603
Mea1	-0.050618459	0.946547466	0.023857603
Fam199x	-0.025141374	0.946547466	0.023857603

Ago1	0.025023554	0.946547466	0.023857603
Sp5	0.065468881	0.946547466	0.023857603
Alk	-0.037317364	0.946807022	0.02373853
Stx18	-0.048712599	0.947007428	0.023646615
Ywhaq	-0.117671983	0.947007428	0.023646615
Rtel1	-0.066167681	0.947007428	0.023646615
Thap3	0.047386525	0.947007428	0.023646615
Mlx	-0.061684383	0.947007428	0.023646615
Dctn4	-0.05256275	0.947007428	0.023646615
Ncam1	0.064405074	0.947007428	0.023646615
Abcc2	0.079592362	0.947007428	0.023646615
Ncbp3	0.050897807	0.947007428	0.023646615
Itm2c	0.060313582	0.947007428	0.023646615
Prr23a3	-0.029198791	0.947007428	0.023646615
Srm	0.053299874	0.947007428	0.023646615
Fthl17a	0.054970575	0.947007428	0.023646615
Pdxdc1	0.026708962	0.947007428	0.023646615
Hmga1	-0.031231291	0.947007428	0.023646615
Ergic2	0.02915293	0.947007428	0.023646615
Tbk1	0.038252286	0.947007428	0.023646615
Mybbp1a	-0.045074185	0.947007428	0.023646615
Kiz	-0.028667907	0.947007428	0.023646615
Xpr1	-0.03468219	0.947007428	0.023646615
Phf10	0.026861445	0.947007428	0.023646615
Fut2	0.017120954	0.947007428	0.023646615
Eif5a	-0.049953653	0.947007428	0.023646615
Lypla1	-0.020684477	0.947007428	0.023646615
Serpinb6e	0.076543739	0.947007428	0.023646615
Sesn3	0.030392504	0.947007428	0.023646615
Glrx5	-0.090299355	0.947007428	0.023646615
Efnb2	0.09525802	0.947007428	0.023646615
Trim61	0.040732183	0.947007428	0.023646615
Akt1	0.028747901	0.947007428	0.023646615
Pus3	0.045230697	0.947007428	0.023646615
Kcnmb1	-0.025731988	0.947007428	0.023646615
Tex16	-0.021927491	0.947007428	0.023646615
Klra10	-0.021528239	0.947007428	0.023646615
Hdac2	-0.026723501	0.947007428	0.023646615
Nmrk2	-0.024745798	0.947007428	0.023646615
Id2	-0.028222777	0.947007428	0.023646615
Naa20	-0.032116608	0.947007428	0.023646615
Smarca5	0.020730372	0.947007428	0.023646615
Sncg	0.021660648	0.947007428	0.023646615
Ophn1	-0.028261099	0.947007428	0.023646615
Lrrc3c	-0.02377225	0.947007428	0.023646615
Vma21	0.056095907	0.947007428	0.023646615
Olfr18	0.02593546	0.947007428	0.023646615
Ffar1	-0.031233097	0.947007428	0.023646615

Lncbate1	-0.082994119	0.947007428	0.023646615
Lyzl6	-0.059993988	0.947007428	0.023646615
Ercc8	-0.022894855	0.947007428	0.023646615
Rhox4c	-0.034954577	0.947007428	0.023646615
Gabarapl2	0.031956632	0.947007428	0.023646615
ZFP33B	-0.042172419	0.947007428	0.023646615
Ntng2	-0.023877659	0.947007428	0.023646615
Mip	-0.057809076	0.947007428	0.023646615
Ccl25	0.024874124	0.947007428	0.023646615
Dnajc5b	0.026419744	0.947007428	0.023646615
Ccdc175	-0.016748134	0.947007428	0.023646615
Dus4l	0.022137877	0.947007428	0.023646615
Psma2	-0.023974626	0.947007428	0.023646615
Rab40c	-0.038127581	0.947007428	0.023646615
B3gnt4	0.035624358	0.947007428	0.023646615
Ccdc117	0.020615617	0.947007428	0.023646615
Sox17	0.029730719	0.947007428	0.023646615
Tfcp2	0.047934838	0.947007428	0.023646615
Catsper2	0.043262707	0.947007428	0.023646615
Gas2	-0.026369106	0.947007428	0.023646615
Muc2	-0.024550982	0.947007428	0.023646615
Gipr	-0.035584887	0.947007428	0.023646615
Nf2	-0.025129529	0.947007428	0.023646615
Olfr967	-0.059268843	0.947007428	0.023646615
ILTIFB	-0.014920196	0.947007428	0.023646615
Krt17	-0.017057829	0.947007428	0.023646615
FMR1OS	-0.022436858	0.947007428	0.023646615
Sema6d	-0.036567229	0.947007428	0.023646615
Cpped1	-0.029155262	0.947007428	0.023646615
Atp13a3	0.051126141	0.947007428	0.023646615
BC03996[illegible]	0.053242912	0.947007428	0.023646615
Dio1	-0.016148371	0.947007428	0.023646615
Ephx3	-0.017844824	0.947007428	0.023646615
Zfp68	-0.032728572	0.947007428	0.023646615
Fbxw2	0.019804033	0.947007428	0.023646615
Igfals	-0.027734356	0.947007428	0.023646615
Aldh1a7	-0.056156762	0.947007428	0.023646615
Cfap161	-0.014746242	0.947007428	0.023646615
Oser1	-0.019446392	0.947007428	0.023646615
Zfp600	0.022352305	0.947007428	0.023646615
Psma7	0.016461166	0.947007428	0.023646615
ASNA1	-0.012847729	0.947007428	0.023646615
Rab33a	-0.027396276	0.947007428	0.023646615
Ccdc97	-0.031574201	0.947007428	0.023646615
Crnde	0.02084619	0.947007428	0.023646615
VWA9	-0.028147687	0.947007428	0.023646615
Csnk1g1	-0.039445169	0.947007428	0.023646615
Clec2h	0.048943728	0.947007428	0.023646615

Stk16	-0.045129045	0.947007428	0.023646615
Sh3gl3	0.030849263	0.947007428	0.023646615
Hsh2d	0.047142291	0.947029222	0.02363662
Dock7	0.033591529	0.947029222	0.02363662
Evi5	0.048002366	0.947029222	0.02363662
Oas1h	-0.031344514	0.947029222	0.02363662
Dppa1	-0.021743662	0.947029222	0.02363662
Mtch2	0.021770309	0.947029222	0.02363662
Fuom	0.025217878	0.947029222	0.02363662
Zfp52	0.034992197	0.947029222	0.02363662
Mrpl45	0.021128104	0.947029222	0.02363662
Mb	0.026028175	0.947029222	0.02363662
Apol7d	0.024518735	0.947029222	0.02363662
Ppp1r3d	0.059817689	0.947029222	0.02363662
Nlrc4	-0.017971147	0.947029222	0.02363662
Tfip11	-0.012661696	0.947029222	0.02363662
Creld2	0.021181917	0.947041088	0.023631178
Olfr1132	-0.018732656	0.947041088	0.023631178
Rps6kb2	0.046164197	0.947086385	0.023610407
Tas2r116	0.047004435	0.947131195	0.023589859
Nme8	-0.057221485	0.947172734	0.023570812
Cep76	0.047864844	0.947172734	0.023570812
Tmem53	0.054063966	0.947317814	0.023504296
Dph2	-0.07622791	0.947317814	0.023504296
Tdrd1	-0.077818035	0.947317814	0.023504296
Dnpep	-0.025306902	0.947317814	0.023504296
Fam135a	0.039034006	0.947317814	0.023504296
Figla	0.029782874	0.947317814	0.023504296
Gsdmc	0.020664483	0.947317814	0.023504296
Oard1	0.028857352	0.947317814	0.023504296
Ppp3cc	0.024496779	0.947317814	0.023504296
Mir133a-1	0.046849532	0.947317814	0.023504296
Olfr857	-0.050022813	0.947317814	0.023504296
Fer1l4	0.017395589	0.947317814	0.023504296
Fggy	-0.06026555	0.947317814	0.023504296
Mannr	-0.036839539	0.947317814	0.023504296
Mon1a	0.022913838	0.947317814	0.023504296
Atp13a2	-0.060487818	0.947317814	0.023504296
9-Mar	-0.027464113	0.947317814	0.023504296
Tbccd1	-0.030983126	0.947317814	0.023504296
Olfr1425	0.033729287	0.947317814	0.023504296
Bhlha9	-0.04622895	0.947317814	0.023504296
Rcc2	0.025556062	0.947317814	0.023504296
Doc2g	-0.022654326	0.947317814	0.023504296
Rbm22	0.036263997	0.947317814	0.023504296
Qrich2	0.022108191	0.947317814	0.023504296
Naf1	0.014628448	0.947317814	0.023504296
Dnajc10	0.020507221	0.947317814	0.023504296

12-Sep	0.028038727	0.947317814	0.023504296
Vmn1r200	-0.019235322	0.947317814	0.023504296
Tomt	0.012591004	0.947317814	0.023504296
Ces1h	0.012584807	0.947317814	0.023504296
D2hgdh	0.032886826	0.947397754	0.023467649
Olfr262	-0.015417842	0.947496409	0.023422427
BC027072	0.022294809	0.947555877	0.023395171
Hrh4	-0.039975958	0.94757761	0.023385209
Kcnk7	0.030871394	0.94757761	0.023385209
Pelo	0.028880125	0.94757761	0.023385209
Olfr1513	0.02881823	0.94757761	0.023385209
Abcb11	-0.024819555	0.94757761	0.023385209
Foxi1	-0.05159432	0.94757761	0.023385209
Slc25a29	-0.063924916	0.94757761	0.023385209
Fbxl14	0.025960956	0.94757761	0.023385209
Snrnp48	-0.03976933	0.94757761	0.023385209
Sec24c	-0.020725175	0.94757761	0.023385209
Il22ra1	0.022240561	0.94757761	0.023385209
Cbl	-0.052229981	0.94757761	0.023385209
Fgf15	-0.063568857	0.94757761	0.023385209
Fancb	-0.018378367	0.94757761	0.023385209
Hmgb1	0.028949182	0.94757761	0.023385209
Mapk12	0.054606917	0.94757761	0.023385209
Bcl9l	-0.018603123	0.94757761	0.023385209
Golga5	-0.117355406	0.94757761	0.023385209
Ddx42	0.042670106	0.94757761	0.023385209
Pfdn6	-0.027071487	0.94757761	0.023385209
Inf2	0.036064666	0.94757761	0.023385209
Olfr577	0.021724251	0.94757761	0.023385209
Vmn1r54	0.022038357	0.94757761	0.023385209
C2cd2l	0.017921503	0.94757761	0.023385209
Lrit2	0.013706316	0.94757761	0.023385209
Tssk1	-0.023668699	0.94757761	0.023385209
Olfr639	0.026465881	0.94757761	0.023385209
Adam25	-0.022323843	0.94757761	0.023385209
Vtcn1	0.013172116	0.94757761	0.023385209
Cars2	0.037612688	0.947924138	0.023226418
Zp2	-0.020064663	0.947924138	0.023226418
Wdr20rt	0.051438606	0.948003043	0.023190269
Mob1b	0.043128874	0.948003043	0.023190269
Cep131	0.062365962	0.948003043	0.023190269
Lipn	-0.035096958	0.948003043	0.023190269
Ccdc134	0.028060252	0.948003043	0.023190269
Sin3a	0.023661433	0.948003043	0.023190269
Mpi	-0.039032402	0.948003043	0.023190269
Dido1	-0.019266303	0.948003043	0.023190269
MESDC2	-0.068706017	0.948003043	0.023190269
Crlf1	0.020715022	0.948003043	0.023190269

Meis2	-0.065824077	0.948003043	0.023190269
Snrnp70	-0.012419126	0.948003043	0.023190269
Fam43b	-0.047961124	0.948199327	0.023100357
Ints6	-0.241144831	0.948352292	0.023030302
Slc6a4	0.038544395	0.948352292	0.023030302
Kdm2a	-0.015697064	0.948411019	0.023003409
Ddo	-0.032413545	0.948487511	0.022968383
Sorbs1	-0.033120453	0.948487511	0.022968383
Myh14	0.053985044	0.948547077	0.02294111
Cyb561d2	-0.020725057	0.948547077	0.02294111
Sfr1	-0.06172393	0.948547077	0.02294111
Sap130	-0.042855217	0.948547077	0.02294111
Tyw5	0.019982478	0.948547077	0.02294111
Trp73	0.066742228	0.948547077	0.02294111
Foxn4	0.024484377	0.948798636	0.022825948
Pfkl	-0.028612256	0.948798636	0.022825948
Thap11	-0.033500519	0.948798636	0.022825948
Gas2l2	-0.021388449	0.948798636	0.022825948
Sh3d19	-0.013863018	0.948798636	0.022825948
Homez	0.023769021	0.948798636	0.022825948
Rnf166	-0.063429744	0.948798636	0.022825948
Dgka	0.0309601	0.948923248	0.022768913
Defb23	0.024124928	0.948923248	0.022768913
Plb1	0.03247773	0.948926364	0.022767487
Olfr1444	0.03263711	0.948926364	0.022767487
Eaf2	0.0437868	0.948926364	0.022767487
Nop10	-0.035347212	0.948926364	0.022767487
Ap4m1	-0.034952713	0.948926364	0.022767487
BC100451	0.035754541	0.948926364	0.022767487
Mcts2	0.049059407	0.948926364	0.022767487
Bsg	-0.029475966	0.948926364	0.022767487
Fundc1	-0.059807436	0.948926364	0.022767487
Zfp583	0.052595177	0.948926364	0.022767487
A1cf	-0.019847361	0.948926364	0.022767487
Tcap	0.018963014	0.948926364	0.022767487
C1qbp	-0.028704612	0.948926364	0.022767487
Kctd20	-0.021922124	0.948926364	0.022767487
Cdc42se2	-0.01766536	0.948926364	0.022767487
Ptrhd1	0.021409442	0.948926364	0.022767487
Parp8	-0.061747784	0.948926364	0.022767487
Olfr222	0.02832542	0.948926364	0.022767487
Olfr33	0.025231846	0.948926364	0.022767487
Zfp277	-0.01227951	0.948926364	0.022767487
Cadm1	0.048127544	0.948926364	0.022767487
Tm2d1	0.04276176	0.948926364	0.022767487
Olfr683	0.031691377	0.949502112	0.022504065
Alkbh7	-0.039892409	0.949794026	0.022370566
Zfp629	-0.032135088	0.949879554	0.02233146

Commd5	0.051634351	0.949931128	0.022307881
Dedd2	-0.065633978	0.949931128	0.022307881
Mynn	-0.068421037	0.949931128	0.022307881
Pnn	-0.039642889	0.949931128	0.022307881
Zfp879	-0.027702787	0.949931128	0.022307881
Apobec2	-0.0264755	0.949931128	0.022307881
Hsd17b13	-0.055451338	0.949931128	0.022307881
Mael	-0.025026574	0.949931128	0.022307881
PDDC1	0.035589571	0.949931128	0.022307881
Fam214b	-0.028832578	0.949931128	0.022307881
Paxbp1	0.021798862	0.949931128	0.022307881
Krt2	-0.0186586	0.949931128	0.022307881
Ccdc162	0.017905939	0.949931128	0.022307881
Firre	0.026931184	0.949931128	0.022307881
SUV420H2	-0.034605371	0.949931128	0.022307881
Rnf181	0.032151306	0.949931128	0.022307881
Sh3bp1	0.022360924	0.949931128	0.022307881
Pabpc4	0.065655603	0.949931128	0.022307881
Zbtb44	0.041691822	0.949931128	0.022307881
Dvl2	-0.054520437	0.949931128	0.022307881
Spata19	-0.030563245	0.949931128	0.022307881
Sox5os3	0.137599019	0.949931128	0.022307881
Gar1	-0.036578787	0.949931128	0.022307881
Rhbdl3	0.025568537	0.949931128	0.022307881
Olfr512	-0.052907466	0.949931128	0.022307881
Krtap4-2	0.100277258	0.949931128	0.022307881
Ube2d-ps	0.026980644	0.949931128	0.022307881
Fam20c	0.030180657	0.949931128	0.022307881
Pus10	0.037501832	0.949931128	0.022307881
Yy2	-0.026073731	0.949931128	0.022307881
Cldn34d	-0.028531382	0.949931128	0.022307881
Ldlrad2	0.021826158	0.949931128	0.022307881
Tmem67	0.061075179	0.949931128	0.022307881
Siah2	-0.029134675	0.949931128	0.022307881
Cdh2	0.043194081	0.949931128	0.022307881
Ndufc2	-0.03231319	0.949931128	0.022307881
Proc	-0.02755287	0.949931128	0.022307881
Il17f	0.014453212	0.949931128	0.022307881
Myh13	0.032607365	0.949931128	0.022307881
Nus1	-0.019999995	0.949931128	0.022307881
Grpel1	0.028626434	0.949931128	0.022307881
Yipf7	-0.01478047	0.949931128	0.022307881
Mief2	-0.024796914	0.949931128	0.022307881
Ccdc110	0.024368793	0.949931128	0.022307881
Adh1	0.045550116	0.949969064	0.022290537
Cdk12	-0.092310704	0.949969064	0.022290537
Slc36a3os	-0.054400815	0.949969064	0.022290537
Zfp334	0.025854672	0.949969064	0.022290537

Ptges3	-0.040040001	0.949969064	0.022290537
Ndufaf3	-0.018326435	0.949969064	0.022290537
Pklr	0.01971187	0.949969064	0.022290537
Pir	0.030675306	0.949969064	0.022290537
Slc6a20b	-0.017422849	0.949969064	0.022290537
Fras1	0.174852976	0.950097664	0.022231749
Gsta3	-0.025482091	0.950097664	0.022231749
Cct3	-0.019211589	0.950493593	0.022050806
Plbd2	-0.032157401	0.95057126	0.022015321
Adam1a	-0.014741829	0.950603824	0.022000443
Fam83h	0.049086915	0.950674025	0.021968372
Mup4	0.066743944	0.950674025	0.021968372
Fgd1	0.022680512	0.950674025	0.021968372
Rogdi	-0.032317916	0.950876551	0.021875862
Adam7	-0.063216671	0.950948935	0.021842804
Ythdf3	0.030453084	0.950948935	0.021842804
Asb12	-0.240419459	0.950948935	0.021842804
Igll1	-0.047035224	0.950948935	0.021842804
Abcb8	0.024821745	0.950948935	0.021842804
Pkp2	-0.026315579	0.950948935	0.021842804
Cox18	-0.210793078	0.950948935	0.021842804
Tmem120	0.064468058	0.950948935	0.021842804
Dubr	0.057373609	0.950948935	0.021842804
Fam120a	0.019424788	0.950948935	0.021842804
Hsd3b1	-0.031060051	0.950948935	0.021842804
Rad9b	-0.01942758	0.950948935	0.021842804
Aif1l	-0.038485525	0.950948935	0.021842804
Rabac1	-0.01844127	0.950948935	0.021842804
Fbxw9	-0.027506153	0.950948935	0.021842804
Lpcat2b	-0.023007353	0.950948935	0.021842804
Smim19	0.024041444	0.950948935	0.021842804
Coro7	0.026598579	0.950948935	0.021842804
Ccdc185	-0.023506096	0.950948935	0.021842804
Cwh43	-0.030401869	0.950948935	0.021842804
Armc3	-0.028693201	0.950948935	0.021842804
Efcab7	-0.040625102	0.950948935	0.021842804
Mup18	0.027075212	0.950948935	0.021842804
Ado	-0.015755089	0.950948935	0.021842804
Rab10	-0.027582132	0.950948935	0.021842804
Zswim2	-0.049023446	0.950952897	0.021840994
Olfr315	0.035967398	0.950952897	0.021840994
Dctn5	0.023478167	0.950952897	0.021840994
Fam114a2	0.026280129	0.950952897	0.021840994
Eif1a	0.01573466	0.950954925	0.021840068
Gcg	0.030676989	0.951038354	0.021801968
Pole4	0.021370882	0.951038354	0.021801968
Rad23a	-0.077784448	0.951038354	0.021801968
Sh2d7	-0.082215248	0.951176541	0.021738869

Grb14	-0.052662654	0.951230584	0.021714195
E2f6	-0.072696764	0.951230584	0.021714195
Ubn1	0.022236989	0.951230584	0.021714195
Tlr11	-0.022405455	0.951230584	0.021714195
Olfr215	0.021239087	0.951230584	0.021714195
Adam34	-0.029293267	0.951230584	0.021714195
Arhgap12	0.035496655	0.951230584	0.021714195
Spaca6	-0.033351066	0.951230584	0.021714195
Zfyve1	0.035790502	0.951230584	0.021714195
DIRC2	-0.028475714	0.95124617	0.021707079
Abhd14a	0.015284925	0.95124617	0.021707079
Sftpa1	0.012160232	0.951250537	0.021705085
Dtnbp1	0.030502244	0.951392971	0.021640061
Nars2	0.016948102	0.951392971	0.021640061
Carnmt1	-0.017473723	0.951479154	0.021600722
Svopl	-0.055270679	0.951671602	0.02151289
Yipf5	-0.054925285	0.951671602	0.02151289
Ttc5	-0.035709498	0.951671602	0.02151289
Ttc21b	-0.020147846	0.951671602	0.02151289
Tigd4	0.020457369	0.951671602	0.02151289
Trappc1	0.018966484	0.951671602	0.02151289
Crb1	-0.027628073	0.951671602	0.02151289
Ano4	-0.028864221	0.951671602	0.02151289
Espn	-0.040351407	0.951671602	0.02151289
Dnaaf1	-0.062417332	0.951722104	0.021489844
Pcbp1	-0.071983032	0.951722104	0.021489844
Arhgef37	-0.043933174	0.951722104	0.021489844
Nip7	-0.035554895	0.951722104	0.021489844
Dll3	0.033118162	0.951722104	0.021489844
Crx	-0.054255138	0.951722104	0.021489844
Clca4b	-0.041952859	0.951722104	0.021489844
Ndufs3	-0.029741516	0.951722104	0.021489844
Srfbp1	-0.025722958	0.951722104	0.021489844
Tmem202	0.022790573	0.951722104	0.021489844
Olfr318	0.02158124	0.951722104	0.021489844
Klrc2	0.013299838	0.951722104	0.021489844
FAM134C	0.02601565	0.951722104	0.021489844
Vcp	0.030756062	0.951722104	0.021489844
PQLC1	-0.024475288	0.951722104	0.021489844
Dok2	-0.021762136	0.951722104	0.021489844
D8Ertd738	-0.115595326	0.951737592	0.021482776
B4galt7	0.023281122	0.951737592	0.021482776
Scly	0.062301535	0.951737592	0.021482776
Pnliprp2	0.019066101	0.951737592	0.021482776
Tigd2	0.019495907	0.951737592	0.021482776
Rgs3	-0.027772401	0.951994514	0.021365554
Sh2d5	-0.015445233	0.951994514	0.021365554
Gmcl1	-0.085451603	0.952079003	0.021327013

Bhmt	0.170509641	0.952079003	0.021327013
Saxo1	-0.038260982	0.952079003	0.021327013
Abca12	-0.10969101	0.952079003	0.021327013
Pcnp	-0.03810472	0.952079003	0.021327013
Abca15	-0.042270788	0.952079003	0.021327013
Pifo	0.026622809	0.952079003	0.021327013
Reg4	0.064529188	0.952079003	0.021327013
Cdh19	0.078158417	0.952079003	0.021327013
Rin1	-0.027024324	0.952079003	0.021327013
Cers3	0.019150218	0.952079003	0.021327013
Exoc4	-0.030918452	0.952079003	0.021327013
Tceal7	-0.028565183	0.952079003	0.021327013
Gfral	-0.023724392	0.952079003	0.021327013
Zmym5	-0.046786406	0.952079003	0.021327013
Rcl1	0.071318072	0.952079003	0.021327013
Tsks	0.027624678	0.952079003	0.021327013
Bpifa2	0.030277128	0.952079003	0.021327013
Rnf133	0.029794801	0.952079003	0.021327013
Sf3b6	0.042024468	0.952079003	0.021327013
Pes1	0.027064675	0.952079003	0.021327013
St7l	-0.027620251	0.952079003	0.021327013
Ntn4	-0.04283611	0.952079003	0.021327013
Mtrf1	-0.019315512	0.952079003	0.021327013
Sh3yl1	-0.022638723	0.952079003	0.021327013
Fnbp1	0.060213444	0.952079003	0.021327013
Pcdhgb1	0.01660202	0.952079003	0.021327013
Jmjd1c	-0.022990571	0.952079003	0.021327013
H2-T3	-0.02413775	0.952079003	0.021327013
Trim39	0.07323472	0.952079003	0.021327013
Nipbl	-0.02709924	0.952079003	0.021327013
Slc19a2	-0.038823163	0.952079003	0.021327013
Ebna1bp2	-0.022002651	0.952079003	0.021327013
Bpgm	0.023898446	0.952079003	0.021327013
Vmn1r43	0.02930714	0.952079003	0.021327013
Gcnt7	-0.08414806	0.952079003	0.021327013
Slc6a11	0.027117175	0.952079003	0.021327013
Cdc73	0.026513128	0.952079003	0.021327013
Olfr876	0.019200188	0.952079003	0.021327013
Riok2	0.025881636	0.952079003	0.021327013
Ccdc84	0.015664403	0.952079003	0.021327013
Olfr521	0.028894056	0.952079003	0.021327013
Mrpl20	0.028968926	0.952079003	0.021327013
Ncbp2	0.023905302	0.952079003	0.021327013
Gtf3c2	-0.021915073	0.952079003	0.021327013
Avpr2	-0.019551017	0.952079003	0.021327013
Fam221a	-0.02485664	0.952079003	0.021327013
Aldh3b3	-0.020515129	0.952079003	0.021327013
Cecr2	0.020350784	0.952079003	0.021327013

Panx3	-0.021937324	0.952079003	0.021327013
Tbx3os2	0.019855012	0.952079003	0.021327013
Podn	0.022463721	0.952079003	0.021327013
Ubap2	-0.023395193	0.952079003	0.021327013
Rbmx	0.020520966	0.952079003	0.021327013
Grcc10	0.02497384	0.952079003	0.021327013
Ranbp10	-0.022619345	0.952079003	0.021327013
Wdr77	0.020418541	0.952079003	0.021327013
Pigr	-0.018223831	0.952079003	0.021327013
Inpp5f	0.022030914	0.952079003	0.021327013
Khsrp	-0.023078728	0.952079003	0.021327013
Ceacam18	0.016647448	0.952079003	0.021327013
Spta1	-0.022839238	0.952079003	0.021327013
Rbm45	0.023800941	0.952079003	0.021327013
Pmfbp1	-0.020763192	0.952079003	0.021327013
Fam71e2	-0.014056302	0.952079003	0.021327013
N4bp3	-0.018792134	0.952079003	0.021327013
Zkscan3	0.020833851	0.952079003	0.021327013
Triml2	-0.026653501	0.952079003	0.021327013
Slco6b1	-0.037828147	0.952079003	0.021327013
Vmn1r4	-0.021744965	0.952079003	0.021327013
Mepce	-0.011780336	0.952079003	0.021327013
Ccdc34os	-0.014493324	0.952079003	0.021327013
Olfr1029	-0.019968537	0.952079003	0.021327013
Grap2	0.02050013	0.952079003	0.021327013
Acot12	-0.035666752	0.952079003	0.021327013
Pym1	0.011807615	0.952079003	0.021327013
Prep	-0.040828873	0.952286428	0.021232405
Mipepos	0.038907928	0.952286428	0.021232405
Slfnl1	0.100685631	0.952321873	0.021216241
Shoc2	0.044485607	0.952321873	0.021216241
C1galt1c1	0.025291053	0.952321873	0.021216241
Scaf4	0.118603188	0.952321873	0.021216241
Ipmk	-0.037451625	0.952336328	0.021209649
BB014433	-0.021743177	0.952336328	0.021209649
SUV420H1	-0.021188957	0.952336328	0.021209649
Galr3	0.012106629	0.952336328	0.021209649
Zfp407	-0.018363393	0.952336328	0.021209649
Scyl1	0.19096795	0.952399435	0.021180871
Rtkn2	-0.024843166	0.952399435	0.021180871
Trappc3	-0.055996777	0.952399435	0.021180871
Lrguk	0.04705373	0.952399435	0.021180871
Chrna6	0.021485311	0.952399435	0.021180871
Star	0.068669017	0.952447048	0.02115916
Tsga8	-0.076889895	0.952546016	0.021114035
Mtf1	-0.029091881	0.952546016	0.021114035
Qrich1	0.02395438	0.952684912	0.021050713
Dcpp3	0.042627916	0.952684912	0.021050713

H13	0.01380216	0.952809036	0.020994133
Themis3	-0.026670984	0.95285502	0.020973174
Klf1	0.047246534	0.952855063	0.020973154
Kctd10	-0.024070497	0.952909999	0.020948116
Hoxd8	-0.032241256	0.952918448	0.020944265
Sgpp1	-0.021926118	0.952918448	0.020944265
Gxylt1	0.022026135	0.9529904	0.020911474
Mms19	0.050982595	0.95303485	0.020891218
Ptgdr2	-0.024106552	0.95303485	0.020891218
Ctsg	0.029077854	0.953119801	0.020852508
Otol1	-0.028100076	0.953119801	0.020852508
Dhx33	0.056819478	0.953119801	0.020852508
Commd2	-0.024201642	0.953119801	0.020852508
LINCRED2	-0.076589762	0.953198439	0.020816678
Slc1a7	-0.053666995	0.953198439	0.020816678
Mvb12b	-0.026018378	0.953198439	0.020816678
Aqp5	-0.024756568	0.953198439	0.020816678
Serpinb1b	0.034557651	0.953198439	0.020816678
Fkbp8	-0.018024214	0.953198439	0.020816678
Mttp	-0.022645058	0.953198439	0.020816678
Ankrd39	0.02796282	0.953198439	0.020816678
Slc43a2	-0.060950239	0.953252722	0.020791946
9-Sep	0.073709759	0.953252722	0.020791946
Zfp553	0.068009184	0.953252722	0.020791946
Kdm3b	-0.045305848	0.953252722	0.020791946
Ntrk2	0.024309448	0.953252722	0.020791946
Ms4a3	0.0398055	0.953252722	0.020791946
Rev1	-0.041093578	0.953252722	0.020791946
Hap1	-0.030112222	0.953252722	0.020791946
Adamts18	-0.01954921	0.953252722	0.020791946
Cry1	0.018273177	0.953252722	0.020791946
Pax6os1	0.030248341	0.953252722	0.020791946
Max	0.014982247	0.953252722	0.020791946
AV039307	0.027736202	0.953252722	0.020791946
Clca4a	-0.023401626	0.953252722	0.020791946
Oscar	0.013834778	0.953252722	0.020791946
Glce	0.02283992	0.953285927	0.020776818
Entpd7	-0.022269077	0.953471947	0.02069208
Pxt1	-0.059947753	0.953487786	0.020684866
Ccbe1	-0.053698107	0.953487786	0.020684866
Tcl1b2	-0.022921194	0.953487786	0.020684866
Dsg1a	-0.022938227	0.953487786	0.020684866
Ficd	0.054636469	0.953487786	0.020684866
Tsn	0.024525698	0.953487786	0.020684866
L7RN6	0.028240818	0.953487786	0.020684866
Ufsp2	-0.020558376	0.953487786	0.020684866
Zfp511	0.023773019	0.953487786	0.020684866
Snx15	0.049636066	0.953487786	0.020684866

Fahd2a	-0.015900494	0.953487786	0.020684866
HIST3H2A	0.03721141	0.953487786	0.020684866
Elac2	-0.025641089	0.953487786	0.020684866
Psg19	0.06350418	0.953487786	0.020684866
Tmprss11l	0.038844574	0.953487786	0.020684866
Nkain3	-0.026141586	0.953487786	0.020684866
Clca1	0.016280767	0.953487786	0.020684866
Pabpc1l	0.055647758	0.953487786	0.020684866
Nacad	0.026494814	0.953487786	0.020684866
Vps16	0.021434236	0.953487786	0.020684866
IRGM	-0.027423519	0.953487786	0.020684866
Efhd1	0.0249773	0.953487786	0.020684866
Keap1	0.021245139	0.953487786	0.020684866
Adgrg2	0.024203009	0.953487786	0.020684866
Cd200r2	0.025585725	0.953487786	0.020684866
Eya3	0.028165812	0.953487786	0.020684866
Mss51	-0.021690322	0.953487786	0.020684866
Zfp628	-0.019499983	0.953487786	0.020684866
Fcf1	0.05352072	0.953487786	0.020684866
Mgat4b	-0.027872367	0.953487786	0.020684866
Txnl1	0.032334221	0.953487786	0.020684866
Ankrd49	-0.015894531	0.953487786	0.020684866
Atp5j	-0.04310354	0.953487786	0.020684866
Aox2	-0.022970579	0.953487786	0.020684866
Lexm	0.027712295	0.953487786	0.020684866
Timd4	0.058579782	0.953487786	0.020684866
Rc3h2	-0.036295518	0.953487786	0.020684866
Tomm6	0.018529001	0.953487786	0.020684866
Invs	0.022296525	0.953487786	0.020684866
Cwc15	-0.018390192	0.953487786	0.020684866
Rpp40	-0.018034607	0.953487786	0.020684866
Hormad1	-0.012858161	0.953487786	0.020684866
Naa35	0.026922117	0.953487786	0.020684866
Gpa33	-0.027315368	0.953493578	0.020682228
UFD1L	-0.02099869	0.953493578	0.020682228
Krt86	0.035940342	0.953500319	0.020679158
Slc18a3	-0.030441141	0.953504514	0.020677246
Rc3h1	0.021237358	0.953669009	0.02060233
Iffo2	0.031624883	0.953669009	0.02060233
Prkag2	0.036341519	0.953669009	0.02060233
Zfp959	-0.028623035	0.953669009	0.02060233
Inip	-0.025405012	0.953669009	0.02060233
Xrcc2	0.021958118	0.953669009	0.02060233
Zfp108	0.043603781	0.953704076	0.020586361
Phf14	-0.016718025	0.953706905	0.020585073
Chst13	0.031927263	0.953706905	0.020585073
Pcbp2	-0.042573824	0.953709523	0.020583881
Hmx3	-0.029364445	0.953880023	0.020506246

Txndc9	-0.014410864	0.953910054	0.020492574
Lage3	0.02803176	0.953953536	0.020472778
Ube4a	-0.026722597	0.954306774	0.020311994
Lama1	-0.092983899	0.954550632	0.02020103
Nedd4	-0.027950652	0.954550632	0.02020103
Gpr87	0.047901685	0.954550632	0.02020103
Card14	0.019447315	0.954550632	0.02020103
Fgl1	0.014179701	0.954550632	0.02020103
Klra6	-0.025108118	0.954550632	0.02020103
Slco3a1	0.033732678	0.954550632	0.02020103
Pga5	0.017538988	0.954550632	0.02020103
Ankrd16	0.077956404	0.954941586	0.020023194
Slc44a4	-0.019871083	0.954941586	0.020023194
Eqtn	0.103790162	0.95496922	0.020010626
Surf2	-0.023419447	0.95496922	0.020010626
Prkar2b	0.023783826	0.9550031	0.019995219
Chid1	0.031797466	0.955060399	0.019969162
Ttc9c	0.048881042	0.955060399	0.019969162
Hal	-0.015503348	0.955133756	0.019935806
Atp6v1f	0.019443793	0.955133756	0.019935806
Pfkfb1	-0.023755816	0.955133756	0.019935806
Airn	-0.017674473	0.955133756	0.019935806
Ugt2b5	-0.021667664	0.955133756	0.019935806
Eno3	-0.040368561	0.955313938	0.019853886
Esr2	-0.041195714	0.955647065	0.01970247
Card10	0.021585402	0.955647065	0.01970247
Rbm39	-0.035931494	0.955647065	0.01970247
Gsg1l2	0.031971289	0.955663737	0.019694893
Zfp93	0.053005815	0.955663737	0.019694893
Rbbp6	0.019129133	0.955663737	0.019694893
Mrps36	0.036529344	0.955663737	0.019694893
Hydin	-0.02451605	0.955663737	0.019694893
Cacng6	0.021787397	0.955663737	0.019694893
Cylc1	0.030363842	0.955663737	0.019694893
Creb3l4	-0.019303689	0.955663737	0.019694893
Topaz1	-0.036320556	0.955663737	0.019694893
Prss38	-0.02893379	0.955663737	0.019694893
Gfy	-0.121593212	0.95582332	0.019622378
Pla2g4d	0.308584205	0.95582332	0.019622378
Ankfn1	-0.055078036	0.95582332	0.019622378
Obp2b	0.055680286	0.95582332	0.019622378
Bex4	-0.055593693	0.95582332	0.019622378
Ulk2	0.019982681	0.95582332	0.019622378
Alox12e	0.023717663	0.95582332	0.019622378
Antxrl	-0.04685013	0.95582332	0.019622378
Olfr1256	0.044488272	0.95582332	0.019622378
Cmss1	-0.063829494	0.95582332	0.019622378
Dcaf7	0.024523547	0.95582332	0.019622378

Serf1	0.030027334	0.95582332	0.019622378
Paics	-0.099789215	0.95582332	0.019622378
Frmpd1os	-0.0393216	0.95582332	0.019622378
Map2k3os	0.019771582	0.95582332	0.019622378
Zfand5	0.027829756	0.95582332	0.019622378
Klk12	-0.040995419	0.95582332	0.019622378
Zfp825	0.023320434	0.95582332	0.019622378
Edrf1	0.01787268	0.95582332	0.019622378
Prmt5	0.021007582	0.95582332	0.019622378
Dyrk1a	0.028027221	0.95582332	0.019622378
Ppil1	0.040923731	0.95582332	0.019622378
Tfap4	-0.029016355	0.95582332	0.019622378
Zfp526	-0.018597844	0.95582332	0.019622378
AY702102	-0.031904568	0.95582332	0.019622378
Bdh1	-0.022317043	0.95582332	0.019622378
Ccdc105	-0.030378872	0.95582332	0.019622378
Krtap5-5	0.048872287	0.955873061	0.019599778
Mrpl43	0.041325033	0.955979309	0.019551507
Eif3d	-0.024239693	0.955979309	0.019551507
Psmd12	0.02306132	0.955990018	0.019546643
Tmem62	0.074565023	0.956003795	0.019540384
Eif4e3	-0.040108925	0.956003795	0.019540384
Dll4	-0.021193275	0.956003795	0.019540384
Pin1rt1	0.018615208	0.956003795	0.019540384
Calcoco1	0.019018587	0.956003795	0.019540384
Mup6	0.016463936	0.956003795	0.019540384
Gatad1	0.017523829	0.956003795	0.019540384
Car5a	0.024103869	0.956003795	0.019540384
Ccr4	-0.025909756	0.956003795	0.019540384
Zfp26	0.030833387	0.956003795	0.019540384
Prkrip1	-0.031861847	0.956003795	0.019540384
Tcea1	-0.011067585	0.956003795	0.019540384
Mfsd2a	0.047362053	0.95606433	0.019512885
Ankrd55	0.027983024	0.95606433	0.019512885
Eml2	-0.028829395	0.95606433	0.019512885
Zfp944	-0.025894612	0.95606433	0.019512885
Isl2	-0.028850092	0.95606433	0.019512885
Csf3	-0.064520793	0.956102045	0.019495753
Zscan30	-0.034014945	0.956102045	0.019495753
Prss37	-0.019677586	0.956102045	0.019495753
Bcam	0.06791823	0.956110821	0.019491767
Mmgt1	0.020984853	0.956110821	0.019491767
Fbxo38	0.023926235	0.956110821	0.019491767
Ppfia1	0.019763009	0.956110821	0.019491767
Hnf4g	0.036491984	0.956110821	0.019491767
Usp38	0.02823434	0.956246311	0.019430227
Ap3s1	0.058442165	0.956246311	0.019430227
Mthfd1l	0.028217606	0.956382491	0.019368384

Dctn6	0.018666497	0.956535699	0.019298817
Appbp2os	-0.074131032	0.956584481	0.019276669
Aida	-0.139404585	0.956584481	0.019276669
Tet3	0.058115285	0.956584481	0.019276669
Rbm5	0.044500916	0.956584481	0.019276669
AA543186	0.019991356	0.956584481	0.019276669
Nfu1	-0.070577303	0.956584481	0.019276669
Rab24	0.061614031	0.956584481	0.019276669
Cyp2d26	0.024048087	0.956584481	0.019276669
Spata25	-0.02777714	0.956584481	0.019276669
Svs3a	0.034327608	0.956584481	0.019276669
Gstm3	0.074966778	0.956584481	0.019276669
Tsr3	0.030659663	0.956584481	0.019276669
Trap1a	0.221060871	0.956584481	0.019276669
Fshb	-0.025966355	0.956584481	0.019276669
Stk11	0.031531899	0.956584481	0.019276669
Peak1	-0.026491635	0.956584481	0.019276669
Il13	-0.049731535	0.956584481	0.019276669
Cttn	0.05016495	0.956584481	0.019276669
Klrb1	0.018627704	0.956584481	0.019276669
Ctnnd1	0.020614524	0.956584481	0.019276669
Ak9	0.015204767	0.956584481	0.019276669
Pnpla5	-0.025674475	0.956584481	0.019276669
SEPW1	0.02055376	0.956584481	0.019276669
Cym	0.024329024	0.956584481	0.019276669
Mrps25	0.050252679	0.956584481	0.019276669
Sohlh2	0.015806215	0.956584481	0.019276669
Gnat3	-0.040034684	0.956584481	0.019276669
Acrv1	-0.023042439	0.956584481	0.019276669
Nle1	0.029647403	0.956584481	0.019276669
Ren1	0.044061096	0.956584481	0.019276669
Jhy	-0.018870325	0.956584481	0.019276669
5-Mar	0.017006789	0.956584481	0.019276669
Heatr9	0.017254623	0.956584481	0.019276669
Nxf2	-0.06054555	0.956652354	0.019245856
Acnat2	0.031319516	0.956652354	0.019245856
Ifitm5	-0.039745791	0.956652354	0.019245856
TUSC5	0.019650161	0.956652354	0.019245856
Zdhhc24	-0.033047765	0.956652354	0.019245856
Hoxc13	0.016933556	0.956652354	0.019245856
Gstm7	0.0259791	0.956652354	0.019245856
Hint2	-0.079228344	0.956793492	0.019181787
Clcc1	0.03695173	0.956793492	0.019181787
Msantd2	0.042332122	0.956793492	0.019181787
Gdpd4	-0.022465072	0.956793492	0.019181787
Nr1h5	0.066825852	0.956793492	0.019181787
Mon2	0.035614793	0.956793492	0.019181787
Exosc10	-0.048362555	0.956793492	0.019181787

Ppp4r1	-0.079401105	0.956793492	0.019181787
Pcgf3	0.031924217	0.956793492	0.019181787
Rbm28	0.02703261	0.956793492	0.019181787
Tesc	0.019939238	0.956793492	0.019181787
HIST1H3G	0.017984031	0.956793492	0.019181787
Echdc1	0.039972091	0.956793492	0.019181787
Sit1	0.059690211	0.956798042	0.019179722
Supt7l	-0.030087201	0.956798042	0.019179722
Prodh2	-0.022529886	0.956798042	0.019179722
Atat1	0.019450272	0.956846787	0.019157597
Clint1	-0.047670084	0.956924168	0.019122477
Klk1b27	-0.025920378	0.956967713	0.019102715
Gimap1os	-0.026769862	0.957037569	0.019071013
Mecom	0.027943197	0.957060066	0.019060805
Cyp4x1os	-0.023424623	0.957060066	0.019060805
Rbck1	-0.022146125	0.957060066	0.019060805
Slbp	0.044266876	0.957060066	0.019060805
Tmem236	0.023239469	0.957060066	0.019060805
Zfp957	0.020852981	0.957060066	0.019060805
Mccc1	-0.024385965	0.957060066	0.019060805
Asah2	0.021200977	0.957295761	0.018953864
Cplx3	-0.019971591	0.957295761	0.018953864
Ptpn22	0.015699325	0.957295761	0.018953864
Wfdc1	0.021801542	0.957395837	0.018908465
WASH1	0.025129831	0.957434874	0.018890758
Tnk2os	0.010789477	0.957434874	0.018890758
Inpp1	-0.028511156	0.957458373	0.018880099
R3hcc1l	-0.022191954	0.95746405	0.018877524
Rmi2	-0.082150658	0.957494952	0.018863507
Psen1	-0.02069644	0.957494952	0.018863507
Zfp433	0.018362128	0.957494952	0.018863507
Myh6	-0.038981289	0.957494952	0.018863507
Nr2f6	0.035298203	0.957494952	0.018863507
Mrpl22	0.013022306	0.957531852	0.01884677
Enkd1	0.048249843	0.957572334	0.01882841
Adgrf1	-0.035543122	0.957572334	0.01882841
Cox4i1	0.041194674	0.957572334	0.01882841
Upf1	0.013518252	0.957572334	0.01882841
Il19	-0.026118986	0.957572334	0.01882841
Cps1	-0.042600404	0.957572334	0.01882841
Dph5	0.074409313	0.957572334	0.01882841
Klhl20	0.028303319	0.957572334	0.01882841
Zfp423	0.020312086	0.957572334	0.01882841
Slc25a53	0.048460946	0.957572334	0.01882841
BC03409(	0.026894638	0.957582636	0.018823738
Rpap2	0.022494145	0.957582636	0.018823738
Cipc	-0.040214879	0.957615777	0.018808708
Trnp1	-0.026111462	0.957615777	0.018808708

Prss39	0.034077142	0.957615777	0.018808708
WDR78	-0.025850651	0.957615777	0.018808708
Zadh2	-0.03473423	0.957615777	0.018808708
Urod	-0.043624967	0.957615777	0.018808708
Ubac1	-0.054601148	0.957615777	0.018808708
Iqcj	0.013736766	0.957615777	0.018808708
Msl3l2	0.025456075	0.957615777	0.018808708
Ifng	0.037530685	0.957615777	0.018808708
Il1rap	0.015488166	0.957615777	0.018808708
Ndufb7	-0.022893756	0.957615777	0.018808708
Krtap1-4	0.029321007	0.957615777	0.018808708
Eepd1	-0.029785416	0.957615777	0.018808708
Ndc1	0.03314307	0.957615777	0.018808708
Sec16a	0.021544559	0.957615777	0.018808708
Sumo3	0.016550999	0.957615777	0.018808708
Med8	-0.015588638	0.957615777	0.018808708
Oxsr1	-0.027649406	0.957615777	0.018808708
Chst12	-0.067088884	0.957615777	0.018808708
CLK2-SCAl	-0.01712398	0.957615777	0.018808708
Olfr750	0.013620413	0.957615777	0.018808708
Cactin	-0.019115714	0.957725486	0.018758955
Tsc22d2	-0.024379925	0.957763334	0.018741793
Pom121l2	0.016779285	0.957827022	0.018712915
Mbip	0.058067738	0.957827022	0.018712915
Prune2	-0.019218216	0.957827022	0.018712915
Cfap36	-0.022403428	0.957827022	0.018712915
Yipf3	0.043449211	0.957827022	0.018712915
Ube2d1	-0.078669652	0.958033016	0.018619524
Gmfb	0.048927838	0.958033016	0.018619524
Clybl	-0.035863332	0.958033016	0.018619524
Rsf1	-0.031639722	0.958033016	0.018619524
Fahd1	0.091111618	0.958033016	0.018619524
Slco6c1	-0.024412384	0.958033016	0.018619524
Timm13	-0.019991304	0.958033016	0.018619524
Sap30l	0.0232927	0.958033016	0.018619524
Zfp410	-0.026600501	0.958033016	0.018619524
Il22ra2	0.098146092	0.958033016	0.018619524
Dpep1	0.0343524	0.958033016	0.018619524
Apela	-0.037603847	0.958033016	0.018619524
Tiprl	0.028064269	0.958033016	0.018619524
Ppp1r3g	-0.030694468	0.958033016	0.018619524
Mrps18a	0.032828212	0.958033016	0.018619524
Calcr	-0.036636022	0.958033016	0.018619524
Vmn2r30	-0.016867815	0.958033016	0.018619524
Ptp4a1	-0.030154854	0.958033016	0.018619524
Nup214	-0.022925577	0.958033016	0.018619524
Dgat2l6	-0.03112554	0.958033016	0.018619524
Csn2	0.026846769	0.958033016	0.018619524

Hoxb3	-0.031933113	0.958033016	0.018619524
Zfp105	0.054945542	0.958033016	0.018619524
Fxyd2	0.037863055	0.958033016	0.018619524
Igbp1b	0.025497235	0.958033016	0.018619524
Zdhhc16	0.035934357	0.958033016	0.018619524
Timm44	-0.014607533	0.958033016	0.018619524
Mrgpra2b	0.031717305	0.958033016	0.018619524
Usp26	0.025718614	0.958033016	0.018619524
Zfp213	-0.022262158	0.958033016	0.018619524
Myf6	-0.031782113	0.958033016	0.018619524
Dcp1a	0.020799751	0.958033016	0.018619524
Mllt10	0.019404137	0.958033016	0.018619524
Mixl1	-0.039955943	0.958033016	0.018619524
Park7	-0.017304394	0.958033016	0.018619524
Cfap45	0.043839929	0.958033016	0.018619524
Evl	0.021652336	0.958033016	0.018619524
Mgat4d	-0.025344005	0.958033016	0.018619524
FAM173B	0.032311361	0.958033016	0.018619524
AA387883	0.029488475	0.958033016	0.018619524
Mxi1	0.019950187	0.958033016	0.018619524
Rhox13	0.032079389	0.958033016	0.018619524
Rhbg	0.018648229	0.958033016	0.018619524
Mospd1	0.029582795	0.958033016	0.018619524
Exosc1	0.01762595	0.958033016	0.018619524
Tpo	0.021946897	0.958033016	0.018619524
Iyd	-0.017062987	0.958033016	0.018619524
Mgam	-0.031964614	0.958033016	0.018619524
Olfr53	-0.027906789	0.958033016	0.018619524
Slco1c1	0.027274859	0.958033016	0.018619524
Pabpc4l	-0.023346967	0.958033016	0.018619524
Paqr4	-0.031687297	0.958033016	0.018619524
Utp6	-0.026839231	0.958033016	0.018619524
Flywch2	-0.020088529	0.958033016	0.018619524
ZCCHC6	0.022525844	0.958033016	0.018619524
Stc1	0.027409189	0.958033016	0.018619524
Trim35	0.029684172	0.958033016	0.018619524
Zfp11	-0.023275899	0.958033016	0.018619524
Mrpl30	-0.014159572	0.958033016	0.018619524
Tdp2	0.011881161	0.958033016	0.018619524
Eef1g	0.062708138	0.958033016	0.018619524
Mettl25	0.017098324	0.958033016	0.018619524
Mak16	-0.015178183	0.958033016	0.018619524
Sptbn5	0.020115082	0.958033016	0.018619524
Epor	0.017564883	0.958033016	0.018619524
Vezf1	-0.033726705	0.958033016	0.018619524
Pcdhga2	-0.010639668	0.958033016	0.018619524
Hhat	0.019000238	0.958033016	0.018619524
Ogfod2	0.02933071	0.958033016	0.018619524

Nos3	-0.019269011	0.958033016	0.018619524
Atad3a	-0.019115837	0.958033016	0.018619524
Brd3	-0.020466339	0.958033016	0.018619524
Tmbim7	0.011003214	0.958033016	0.018619524
Klk11	0.010586476	0.958033016	0.018619524
Ercc4	-0.026710179	0.958077415	0.018599397
Ccne2	-0.021643289	0.958083287	0.018596736
Serpinb9c	-0.031604405	0.958115561	0.018582106
Trhr	-0.084294319	0.958115561	0.018582106
Prss34	0.03811487	0.958120077	0.018580059
Zbtb41	-0.027968729	0.958120077	0.018580059
Ryr3	-0.024079957	0.958120077	0.018580059
Ippk	0.018761884	0.958260455	0.018516434
Tle1	-0.041488144	0.958303432	0.018496956
Clcnkb	-0.056208172	0.958382786	0.018460996
Anxa6	-0.060077889	0.958382786	0.018460996
Klhl9	0.041979454	0.958382786	0.018460996
Slc5a4b	0.0367341	0.958382786	0.018460996
Dnajc9	0.072272397	0.958382786	0.018460996
Cdcp2	0.028156532	0.958382786	0.018460996
Babam1	-0.026870545	0.958382786	0.018460996
Ceacam15	-0.020277341	0.958382786	0.018460996
Dpm2	0.015681123	0.958382786	0.018460996
Hnrnpl	-0.041907575	0.958382786	0.018460996
Gde1	0.024532133	0.958382786	0.018460996
Tango6	-0.023546852	0.958382786	0.018460996
Ube2g1	0.072261209	0.958382786	0.018460996
Adh4	0.03045881	0.958382786	0.018460996
Zfp667	0.022833031	0.958382786	0.018460996
DOXL2	0.01913907	0.958382786	0.018460996
Ctc1	-0.028146653	0.958382786	0.018460996
L3mbtl4	0.018981206	0.958382786	0.018460996
RNMTL1	-0.01765683	0.958382786	0.018460996
Arglu1	-0.036114941	0.958382786	0.018460996
Snx11	0.018242238	0.958382786	0.018460996
Ddx47	0.026556097	0.958382786	0.018460996
Wdr83	0.026550333	0.958382786	0.018460996
Ankmy2	0.024883945	0.958382786	0.018460996
Pdcl2	-0.019987005	0.958382786	0.018460996
Fundc2	0.018069427	0.958382786	0.018460996
Cyp2r1	0.037263207	0.958382786	0.018460996
Smco2	0.013186623	0.958382786	0.018460996
Agt	0.022150938	0.958382786	0.018460996
Igf2bp1	-0.045233634	0.958382786	0.018460996
Cdhr5	0.021684754	0.958382786	0.018460996
Il5ra	0.019191285	0.958382786	0.018460996
Cdc40	-0.022154595	0.958382786	0.018460996
Dcaf12l2	0.012495274	0.958382786	0.018460996

Krt10	0.019957785	0.958382786	0.018460996
Aqp6	0.031065827	0.958382786	0.018460996
Gan	-0.020865894	0.958382786	0.018460996
Pla2g12a	0.026287853	0.958382786	0.018460996
Bmp10	-0.0239116	0.958382786	0.018460996
Pabpc2	-0.0111416	0.958382786	0.018460996
Gpr82	-0.026437855	0.958382786	0.018460996
Scn4a	-0.01825971	0.958382786	0.018460996
Rce1	0.010378424	0.958382786	0.018460996
Scn11a	0.028667106	0.958384077	0.01846041
Gipc1	0.084907743	0.958384077	0.01846041
Pate3	-0.031973457	0.958384077	0.01846041
Ifi27l2b	0.011356463	0.958431014	0.018439141
Xkr9	-0.013292879	0.958443949	0.01843328
Acad8	-0.020498553	0.958576547	0.018373201
Rpn1	-0.098555268	0.95859195	0.018366222
Cep78	-0.086880008	0.95859195	0.018366222
Gzmk	-0.057428855	0.95859195	0.018366222
Zfp518a	-0.026164087	0.95859195	0.018366222
Pygo2	-0.023617308	0.95859195	0.018366222
Nsg2	-0.017014364	0.95859195	0.018366222
Cstl1	0.027992982	0.95859195	0.018366222
BC094916	-0.066733146	0.95859195	0.018366222
Zcrb1	-0.02118453	0.95859195	0.018366222
Pck1	-0.128587257	0.95859195	0.018366222
Mat1a	-0.025529432	0.95859195	0.018366222
Vmn2r35	0.015017995	0.95859195	0.018366222
Ermard	-0.021358678	0.95859195	0.018366222
Chchd3	0.028458747	0.95859195	0.018366222
Zic3	0.129069359	0.958682991	0.018324978
Krtap1-3	-0.026858516	0.958682991	0.018324978
Bcas2	0.014121894	0.958682991	0.018324978
Platr21	-0.015771418	0.958702572	0.018316108
Plekhh2	-0.011115953	0.958702572	0.018316108
Tnrc6a	-0.017965251	0.959066689	0.018151193
Dhtkd1	0.046300376	0.959098388	0.018136839
Ttyh3	0.015828385	0.959177801	0.018100881
Kdm2b	-0.023601957	0.959242268	0.018071693
Sac3d1	-0.05681704	0.959242268	0.018071693
Nanos3	-0.050449675	0.959242268	0.018071693
BC106175	0.02018313	0.959242268	0.018071693
Nudt2	0.014315251	0.959242268	0.018071693
Olfr875	0.021111763	0.959242268	0.018071693
Atad3aos	-0.101088031	0.959242268	0.018071693
Atic	-0.018497368	0.959242268	0.018071693
SELK	0.017143121	0.959242268	0.018071693
Eva1c	0.016620566	0.959242268	0.018071693
Apoa4	0.016197356	0.959242268	0.018071693

Tpra1	-0.010238601	0.959242268	0.018071693
Khdrbs1	-0.013091903	0.959242268	0.018071693
Usp44	-0.010869104	0.959463538	0.017971525
Vmn2r55	-0.026876237	0.959531233	0.017940884
Evc	-0.018746327	0.959608513	0.017905908
Znrf4	-0.019794429	0.959608513	0.017905908
Tmem237	-0.025690572	0.959634734	0.017894041
Bzw1	-0.025977556	0.959634734	0.017894041
Oxld1	0.027538756	0.959634734	0.017894041
Cdh3	0.028468067	0.959634734	0.017894041
Lmnb2	-0.023920653	0.959634734	0.017894041
Atp5o	0.020383469	0.959634734	0.017894041
Tmem248	-0.022211564	0.959634734	0.017894041
Nol11	0.015857811	0.959634734	0.017894041
Trmt6	0.023476714	0.959634734	0.017894041
Apof	-0.031134295	0.959634734	0.017894041
Platr7	0.04529869	0.959634734	0.017894041
Brca2	0.037876701	0.959634734	0.017894041
Gen1	-0.030082303	0.959634734	0.017894041
Sdr16c5	0.015463845	0.959634734	0.017894041
Tada3	-0.032112763	0.959634734	0.017894041
Ptprh	0.016717639	0.959634734	0.017894041
Lrrc37a	0.030644333	0.959634734	0.017894041
Srrd	0.031603937	0.959634734	0.017894041
Itga9	-0.021299328	0.959634734	0.017894041
Klhl31	-0.042554993	0.959634734	0.017894041
Nlrp5	0.024537061	0.959634734	0.017894041
Dpysl2	-0.02447142	0.959634734	0.017894041
Atg7	0.018344896	0.959634734	0.017894041
Dyrk2	-0.019506632	0.959634734	0.017894041
Cluap1	0.022095462	0.959634734	0.017894041
Cnep1r1	-0.010655514	0.959634734	0.017894041
Chil6	-0.060242674	0.959660113	0.017882556
Mtg2	0.040374347	0.959660113	0.017882556
Ubl5	0.078065217	0.959660113	0.017882556
Cwc25	0.04815912	0.959660113	0.017882556
Tll2	0.037949575	0.959674961	0.017875836
Zfp160	-0.029632057	0.959674961	0.017875836
Prr7	-0.021027938	0.959703504	0.01786292
Bzw2	-0.020302655	0.959703504	0.01786292
LRRC16A	-0.023103709	0.959703504	0.01786292
Bcap29	-0.020690895	0.959823535	0.017808605
Slc34a3	0.023430611	0.959956867	0.01774828
Grb10	-0.027123401	0.960012977	0.017722896
Dusp13	-0.025761045	0.960012977	0.017722896
Syde2	0.021119089	0.960012977	0.017722896
Zic4	0.014754296	0.960012977	0.017722896
Lgr6	0.011373667	0.960044802	0.0177085

IPW	-0.026479593	0.960044802	0.0177085
Ap1m1	0.026786293	0.960080792	0.017692219
Nudt11	0.021562758	0.960123613	0.017672849
Pias1	-0.030510679	0.960123613	0.017672849
Tmod1	0.029837324	0.960123613	0.017672849
Fech	-0.019189948	0.960123613	0.017672849
FAM188A	-0.069860475	0.960123613	0.017672849
Trim55	-0.022287049	0.960123613	0.017672849
Kctd19	-0.019817459	0.960123613	0.017672849
Lig4	-0.028808356	0.960123613	0.017672849
Setd6	0.033080848	0.96012942	0.017670222
Srsf4	0.043226098	0.96012942	0.017670222
Grik2	0.027094007	0.96012942	0.017670222
Apoo	-0.046299215	0.960154516	0.017658871
Fam228a	-0.055796699	0.960203263	0.017636823
Vmn2r56	0.036242818	0.960203263	0.017636823
Tc2n	-0.02589677	0.960208673	0.017634376
Rnf141	-0.034047109	0.960208673	0.017634376
Caprin1	0.018988565	0.960530006	0.017489064
Spata7	-0.066419465	0.960530006	0.017489064
Kmt2b	-0.024559706	0.960530006	0.017489064
Mettl8	0.026622407	0.960530006	0.017489064
Zfhx4	-0.036378617	0.960530006	0.017489064
Prpsap2	0.040065121	0.960530006	0.017489064
Hsd3b2	0.017829688	0.960530006	0.017489064
Strn3	-0.018173859	0.960530006	0.017489064
H2-M10.5	0.033282441	0.960530006	0.017489064
Skap1	-0.019567594	0.960530006	0.017489064
Kcnk18	-0.039979104	0.960530006	0.017489064
Ifrd2	-0.010945939	0.960530006	0.017489064
Il25	0.033675159	0.960620881	0.017447977
Lkaaear1	-0.016519409	0.960620881	0.017447977
Mrpl28	-0.030803881	0.960620881	0.017447977
Fhad1	-0.019839911	0.960620881	0.017447977
Vsig8	0.026708393	0.960620881	0.017447977
Pla2g12b	-0.045744857	0.960620881	0.017447977
Fkbp1a	0.015092266	0.960620881	0.017447977
Exosc3	0.021576263	0.960620881	0.017447977
Cdkn2aipr	-0.013765579	0.960620881	0.017447977
Pgam2	0.026024846	0.960620881	0.017447977
Ffar2	0.016836137	0.960627552	0.017444961
Ints8	-0.085329974	0.960627552	0.017444961
Cdyl	0.023317265	0.960627552	0.017444961
Ldb3	0.018509746	0.960627552	0.017444961
Eif5	0.033434408	0.960712402	0.017406603
Yeats2	0.026572123	0.960712402	0.017406603
Gpcpd1	-0.016591731	0.960753816	0.017387882
Cyp2t4	0.129937154	0.960753816	0.017387882

Tgfbr3	-0.046680519	0.960753816	0.017387882
Hoga1	0.036142768	0.960753816	0.017387882
Abca14	0.033659119	0.960753816	0.017387882
Fgf17	-0.031035638	0.960753816	0.017387882
Zfp54	0.029638913	0.960753816	0.017387882
Spry1	0.063806502	0.960753816	0.017387882
Pfn3	-0.023665378	0.960753816	0.017387882
TAZ	0.051546108	0.960753816	0.017387882
Awat1	-0.020342358	0.960753816	0.017387882
Hrnr	0.025528728	0.960753816	0.017387882
Aknad1	0.042770541	0.960753816	0.017387882
Opn5	0.02165397	0.960753816	0.017387882
Lifr	0.01899814	0.960753816	0.017387882
Mrpl2	-0.022199144	0.960753816	0.017387882
Pabpn1	0.025335724	0.960753816	0.017387882
Mroh2a	-0.033065998	0.960753816	0.017387882
Pkd1l1	-0.049662954	0.960753816	0.017387882
Chuk	0.035519677	0.960753816	0.017387882
Atxn7	0.03581545	0.960753816	0.017387882
Csnk2b	-0.021740459	0.960753816	0.017387882
Olfr267	0.052565779	0.960753816	0.017387882
Anapc1	0.017995047	0.960753816	0.017387882
Mfsd14a	-0.016138538	0.960753816	0.017387882
Catsperd	0.033938821	0.960753816	0.017387882
Tmeff2	-0.015122614	0.960753816	0.017387882
Rsl1d1	-0.028265153	0.960753816	0.017387882
Art2b	0.015895944	0.960753816	0.017387882
Zbtb10	0.017327437	0.960753816	0.017387882
Ccdc51	-0.012618763	0.960753816	0.017387882
Aebp2	0.027244698	0.960753816	0.017387882
Mfsd4b5	0.043807397	0.960753816	0.017387882
Qser1	-0.049816757	0.960753816	0.017387882
Sema6a	0.021135439	0.960753816	0.017387882
Ddx6	0.013974217	0.960753816	0.017387882
Flrt3	-0.010335521	0.960753816	0.017387882
Ackr1	-0.009897031	0.960753816	0.017387882
Hltf	-0.027934689	0.960753816	0.017387882
Vax2os	0.026346059	0.96076375	0.017383391
Ovgp1	0.024408801	0.96076375	0.017383391
Lins1	-0.069217482	0.960844414	0.01734693
Ifnz	-0.01729291	0.960844414	0.01734693
Ofcc1	0.01515568	0.960844414	0.01734693
Zp3	-0.024938069	0.960844414	0.01734693
Tmem235	0.022173804	0.960844414	0.01734693
Atp5d	-0.025947311	0.960844414	0.01734693
Insrr	0.026229259	0.960844414	0.01734693
Slc15a5	-0.036845857	0.960844414	0.01734693
Elp4	-0.023361929	0.960844414	0.01734693

Nudt16l1	0.021210546	0.960844414	0.01734693
Tmem61	-0.022029401	0.960844414	0.01734693
Nkx6-1	0.082563177	0.960899228	0.017322155
Ttll9	0.020230065	0.960899228	0.017322155
Stoml3	0.02206496	0.961071548	0.01724428
Abhd17b	-0.050685099	0.96109371	0.017234265
Brox	-0.021364473	0.96109371	0.017234265
Syce1l	-0.093307016	0.961215984	0.017179016
Akr1c18	-0.092135608	0.961215984	0.017179016
Tomm6os	0.033991688	0.961215984	0.017179016
Cep63	0.018343412	0.961215984	0.017179016
Cux1	-0.053310647	0.961215984	0.017179016
Mrpl12	-0.068266983	0.961354456	0.017116456
Csgalnact	0.085114355	0.961399832	0.017095958
Skint8	-0.109879913	0.961463992	0.017066976
Psmg1	-0.031815496	0.961463992	0.017066976
Phb	0.015344816	0.961463992	0.017066976
Spata31d1	-0.029347256	0.961480061	0.017059718
Zfp97	0.012647991	0.961573497	0.017017515
Mirt1	0.044267591	0.961715801	0.016953248
Srek1	0.015552265	0.961715801	0.016953248
Otx2	0.033674457	0.961865533	0.016885637
Stx8	0.058222789	0.961865533	0.016885637
PIH1D3	0.06203437	0.961865533	0.016885637
Tmem9b	-0.04550863	0.961865533	0.016885637
Olfr658	0.017994654	0.961865533	0.016885637
Olfr286	0.018185955	0.961865533	0.016885637
Acsm3	-0.027191679	0.961865533	0.016885637
Fer	0.04417156	0.961865533	0.016885637
Kcna10	0.039947898	0.961865533	0.016885637
Xpo5	-0.014921157	0.961865533	0.016885637
Nxt2	-0.023771228	0.961865533	0.016885637
Exoc5	0.019863114	0.961865533	0.016885637
Larp7	0.024432437	0.961865533	0.016885637
Mrpl39	-0.021684369	0.961865533	0.016885637
Hmx1	-0.040852573	0.961865533	0.016885637
Ccne1	0.018418996	0.961865533	0.016885637
Neo1	-0.024755675	0.961865533	0.016885637
Usp21	-0.017848204	0.961865533	0.016885637
Map2k1	-0.015818954	0.961865533	0.016885637
Pcdhgb7	0.027527032	0.961865533	0.016885637
BC030870	-0.017400328	0.961878815	0.01687964
Senp1	0.0242072	0.961959755	0.016843097
Gucy1b2	-0.03054686	0.961986012	0.016831243
H60c	-0.020579048	0.962118815	0.016771292
Nphs1	0.220581137	0.962139934	0.016761759
H60b	0.044173859	0.962139934	0.016761759
Cldn34b2	-0.092908553	0.962139934	0.016761759

Optc	-0.026087743	0.962139934	0.016761759
Htr2c	-0.028397553	0.962139934	0.016761759
Serpina7	0.018763434	0.962139934	0.016761759
Rbm27	-0.016497046	0.962139934	0.016761759
SSSCA1	-0.013734684	0.962139934	0.016761759
BC004004	-0.021570333	0.962139934	0.016761759
Vstm2b	-0.03756862	0.962139934	0.016761759
Inafm2	0.018314721	0.962139934	0.016761759
Kcnq1	0.014410141	0.962139934	0.016761759
Zfp462	0.020935975	0.962139934	0.016761759
Rsph9	0.026266869	0.962139934	0.016761759
Lhb	0.015621684	0.962139934	0.016761759
Tsx	-0.017276494	0.962139934	0.016761759
Pknox1	0.016658891	0.962139934	0.016761759
Ccnb1ip1	0.061284465	0.962139934	0.016761759
Flii	-0.016059357	0.962139934	0.016761759
Phxr4	0.015445118	0.962139934	0.016761759
ASTX	-0.01616055	0.962139934	0.016761759
Wdr5b	-0.009660131	0.962139934	0.016761759
Lactbl1	0.01137929	0.962139934	0.016761759
Ccdc83	-0.029177215	0.962139934	0.016761759
SMEK1	-0.023284214	0.962157764	0.016753711
Dbil5	0.020435252	0.962157764	0.016753711
Mrpl40	-0.016273855	0.962157764	0.016753711
Tmem126	0.017889204	0.962157764	0.016753711
Ube2g2	0.032190668	0.962157764	0.016753711
Zdhhc19	-0.015088426	0.962157764	0.016753711
Actl7a	-0.011911274	0.962157764	0.016753711
Mthfd1	0.027463323	0.962157764	0.016753711
Dpy19l2	-0.016014248	0.962157764	0.016753711
Cdc20b	0.01369467	0.962157764	0.016753711
Lyg2	-0.016674264	0.962157764	0.016753711
Ptf1a	-0.021070839	0.962203577	0.016733033
Wnt6	-0.022233881	0.962203577	0.016733033
Ubxn7	0.020930429	0.9623637	0.016660767
Dbt	-0.047411062	0.962552929	0.01657538
Uck2	-0.06796903	0.962552929	0.01657538
BB094273	0.030805211	0.962619521	0.016545335
Dcaf4	0.028650426	0.962619521	0.016545335
Ppif	0.159302014	0.962671696	0.016521797
Tmem115	-0.024743469	0.962671696	0.016521797
Gpatch2	0.028926952	0.962671696	0.016521797
Zfp661	-0.042149722	0.962671696	0.016521797
Nup153	0.071330676	0.962671696	0.016521797
Trpv2	-0.014600194	0.962671696	0.016521797
MICALCL	-0.021202101	0.962671696	0.016521797
Fnbp4	-0.026569472	0.962671696	0.016521797
Vbp1	0.118652956	0.962671696	0.016521797

Zfp952	0.022672177	0.962671696	0.016521797
Tuba3b	-0.020722091	0.962671696	0.016521797
Hoxaas2	0.026083881	0.962671696	0.016521797
Ptpmt1	-0.016156873	0.962671696	0.016521797
Cpa6	0.015538432	0.962671696	0.016521797
Tspan10	-0.013940468	0.962671696	0.016521797
Mamstr	-0.049300274	0.962702798	0.016507766
Epas1	-0.077828939	0.962702798	0.016507766
Speer4a	0.064697138	0.962702798	0.016507766
Pdcd2l	0.027068891	0.962702798	0.016507766
F2	-0.049474516	0.962702798	0.016507766
Gcnt3	0.063314059	0.962702798	0.016507766
Cacng8	-0.05329209	0.962702798	0.016507766
Gpx2	0.040573641	0.962702798	0.016507766
Dcakd	-0.030565361	0.962702798	0.016507766
BB283400	-0.020757442	0.962702798	0.016507766
Ear2	0.025467498	0.962702798	0.016507766
Cep89	-0.019048176	0.962702798	0.016507766
Atp5c1	0.035820551	0.962702798	0.016507766
Gstt1	0.029678712	0.962702798	0.016507766
Krt33b	-0.018842486	0.962702798	0.016507766
Grk1	-0.023133446	0.962702798	0.016507766
Abhd10	-0.032074533	0.962702798	0.016507766
Zfp687	-0.020911298	0.962702798	0.016507766
Nprl3	0.020699337	0.962702798	0.016507766
Tek	-0.015007911	0.962702798	0.016507766
AU022751	0.047008447	0.962702798	0.016507766
Zfp113	0.019923872	0.962702798	0.016507766
Emd	-0.014690396	0.962702798	0.016507766
Itgb1bp2	-0.02665619	0.962702798	0.016507766
Adam32	0.012187953	0.962702798	0.016507766
Nin	0.020655658	0.962702798	0.016507766
Rspo4	0.071492118	0.962702798	0.016507766
Eno4	0.014873663	0.962702798	0.016507766
Defb30	0.023401518	0.962702798	0.016507766
Pgk2	0.019768198	0.962702798	0.016507766
Stx7	-0.018436099	0.962702798	0.016507766
Hjurp	-0.024385709	0.962702798	0.016507766
Mrps30	0.039848591	0.962702798	0.016507766
Mdp1	-0.021640916	0.962702798	0.016507766
Cxxc1	0.011881716	0.962702798	0.016507766
Fscn3	0.018416586	0.962702798	0.016507766
Zfp955a	0.019360358	0.962702798	0.016507766
St18	0.032176566	0.962702798	0.016507766
SVS2	0.015628655	0.962702798	0.016507766
Tm9sf2	-0.016860877	0.962702798	0.016507766
Sppl2b	-0.01840087	0.962702798	0.016507766
Immp1l	0.031137599	0.962702798	0.016507766

Slc20a2	0.016126738	0.962702798	0.016507766
Synpo2l	0.031710046	0.962702798	0.016507766
Pou4f2	-0.020117943	0.962702798	0.016507766
Saraf	0.022406039	0.962702798	0.016507766
Armc7	0.022076925	0.962702798	0.016507766
Crisp1	0.023115283	0.962702798	0.016507766
Lmo7	0.012586666	0.962702798	0.016507766
Chmp4b	-0.024398318	0.962702798	0.016507766
D10WSU1(	-0.0098415	0.962702798	0.016507766
Tulp4	0.010017778	0.962702798	0.016507766
Esm1	0.024866899	0.962702798	0.016507766
Dgkeos	0.009734597	0.962702798	0.016507766
Krt31	-0.041774133	0.962798347	0.016464664
Pnliprp1	0.033704215	0.962967352	0.016388437
Lrriq3	-0.029884942	0.962967352	0.016388437
Upb1	-0.016254586	0.962967352	0.016388437
Abcg8	-0.029044588	0.962967352	0.016388437
Mdh1b	-0.016556511	0.962967352	0.016388437
Speer4c	0.032461737	0.962967352	0.016388437
Fam177a	-0.02655569	0.962967352	0.016388437
Nbn	-0.017832084	0.962967352	0.016388437
Serpinb10	-0.08606342	0.962967352	0.016388437
Rbm41	0.020312927	0.962967352	0.016388437
Grin2c	0.02604182	0.963055	0.016348909
Ldlrad1	-0.018381517	0.963055	0.016348909
Rslcan18	0.016672137	0.963161181	0.01630103
AIM1L	-0.017014866	0.963161181	0.01630103
Scml1	-0.092414378	0.9633008	0.016238079
Scyl2	-0.028938449	0.9633008	0.016238079
Rreb1	0.020703101	0.9633008	0.016238079
Pou3f3	0.018585631	0.9633008	0.016238079
Cyp24a1	-0.025079933	0.9633008	0.016238079
Crnkl1	0.014172776	0.9633008	0.016238079
Mok	-0.022523502	0.9633008	0.016238079
Cog3	0.015009782	0.9633008	0.016238079
Ncoa5	0.016094425	0.9633008	0.016238079
Clca3b	0.014970228	0.9633008	0.016238079
Cdx4	0.011784901	0.9633008	0.016238079
Kdm4c	-0.030084657	0.9633008	0.016238079
Sppl2c	-0.01523368	0.9633008	0.016238079
St8sia4	-0.02168725	0.9633008	0.016238079
Npr1	0.057482931	0.9633008	0.016238079
Apol7a	0.021097798	0.963491568	0.016152082
Col9a1	-0.086894542	0.963544464	0.016128239
Dzip1l	0.017648671	0.963544464	0.016128239
Has2os	0.023795842	0.963544464	0.016128239
Otx2os1	0.032443107	0.963544464	0.016128239
Tmem167	0.019172116	0.963544464	0.016128239

Cep112it	0.018629756	0.963544464	0.016128239
Nphs2	0.010052497	0.963544464	0.016128239
Abhd18	-0.034677757	0.963639476	0.016085417
H2AFZ	0.037638057	0.963709261	0.016053968
Tgfb3	0.03596531	0.963709261	0.016053968
Olfr90	-0.02021116	0.963709261	0.016053968
FAM46D	-0.014094803	0.963709261	0.016053968
Casq2	-0.038070906	0.963709261	0.016053968
Rad52	0.016657742	0.963709261	0.016053968
Rrp15	0.0129876	0.963740047	0.016040094
Cyp2w1	-0.063453049	0.963833046	0.015998187
Faim	-0.016887748	0.963895327	0.015970125
Zswim3	-0.037487585	0.96400309	0.015921574
Fga	-0.030784647	0.96400309	0.015921574
Fan1	-0.047265576	0.964035268	0.015907077
Vps72	-0.042170168	0.964035268	0.015907077
Polg	0.021917845	0.964035268	0.015907077
Nol4l	0.013886284	0.96416263	0.015849705
Adcy10	0.061491095	0.964256106	0.015807602
Ctsf	0.015005325	0.964256106	0.015807602
Calr	-0.017635636	0.964256106	0.015807602
Entpd3	0.042233327	0.964312249	0.015782317
Prmt9	0.017115537	0.964312249	0.015782317
Msi2	0.028602048	0.964312249	0.015782317
Arhgap23	-0.012138633	0.964312249	0.015782317
Olfr921	0.027445862	0.964324032	0.01577701
Dis3l	0.02363431	0.964474437	0.015709279
Supt16	0.030755058	0.964474437	0.015709279
Myoz3	-0.023053027	0.964583556	0.015660146
Xlr5a	0.031965765	0.964583556	0.015660146
Pi4kb	0.024719209	0.964583556	0.015660146
Mrpl42	0.019843017	0.964583556	0.015660146
Zfp345	0.022581786	0.964625177	0.015641407
Ifne	0.029367198	0.964625177	0.015641407
Ccdc150	-0.024149213	0.964625177	0.015641407
Prr15l	-0.0958046	0.964625177	0.015641407
Cdc26	0.015278579	0.964625177	0.015641407
Coq7	-0.071597123	0.964674339	0.015619274
Papln	0.013131058	0.964674339	0.015619274
Cd207	-0.120438419	0.964674339	0.015619274
Shisa2	0.017478812	0.964674339	0.015619274
Fbxw15	0.017686549	0.964674339	0.015619274
Kbtbd6	-0.04198156	0.964674339	0.015619274
Arf5	0.019234739	0.964794291	0.015565275
Eif4ebp2	-0.025808296	0.965015871	0.015465544
Efhb	0.018547835	0.965015871	0.015465544
Spata16	0.107709558	0.965160071	0.015400653
Rnf26	0.009177863	0.965160071	0.015400653

Dynll1	-0.053263164	0.965230111	0.015369139
ORAOV1	-0.051535935	0.965230111	0.015369139
Ascc2	-0.060936552	0.965230111	0.015369139
Rrs1	-0.038128786	0.965230111	0.015369139
Ces2g	-0.046233087	0.965230111	0.015369139
Atad2b	-0.033309525	0.965230111	0.015369139
Fam98a	-0.069103297	0.965230111	0.015369139
Dmgdh	0.038896142	0.965230111	0.015369139
Pa2g4	0.042985725	0.965230111	0.015369139
Hnrnpc	-0.016203688	0.965230111	0.015369139
Lrriq4	-0.060828742	0.965230111	0.015369139
Nudt21	-0.025937744	0.965230111	0.015369139
Thyn1	0.036979338	0.965230111	0.015369139
Mepe	-0.028843117	0.965230111	0.015369139
Clec3a	0.040198184	0.965230111	0.015369139
Fcrlb	-0.05053813	0.965230111	0.015369139
Angptl6	0.026478054	0.965230111	0.015369139
Nlrp12	-0.049325932	0.965230111	0.015369139
Olfr1426	-0.030025678	0.965230111	0.015369139
Mageb16	0.055487435	0.965230111	0.015369139
Krt24	0.016563093	0.965230111	0.015369139
Prps1l1	0.028274075	0.965230111	0.015369139
Otud4	-0.022641514	0.965230111	0.015369139
Arid4a	0.054430536	0.965230111	0.015369139
Pcsk7	0.021076107	0.965230111	0.015369139
Pcnx3	0.033157101	0.965230111	0.015369139
Ptk6	0.016191867	0.965230111	0.015369139
Tspear	0.019130976	0.965230111	0.015369139
Mbtps1	0.065604811	0.965230111	0.015369139
Dmbt1	0.056321735	0.965230111	0.015369139
H2AFY	-0.020841327	0.965230111	0.015369139
Tspan3	0.018094491	0.965230111	0.015369139
Caskin2	0.019614851	0.965230111	0.015369139
Ano1	0.035387304	0.965230111	0.015369139
Cdhr2	-0.01628578	0.965230111	0.015369139
Suz12	0.018052339	0.965230111	0.015369139
Gtf2b	-0.033998794	0.965230111	0.015369139
Trp53rka	0.015028159	0.965230111	0.015369139
Cox7c	-0.015272255	0.965230111	0.015369139
Myo3a	-0.031993322	0.965230111	0.015369139
Rsl24d1	0.023208611	0.965230111	0.015369139
Ano9	-0.023350966	0.965230111	0.015369139
Rnd2	-0.016046619	0.965230111	0.015369139
Phf21a	0.016615843	0.965230111	0.015369139
Ssc4d	-0.025972209	0.965230111	0.015369139
Ccm2	-0.017604634	0.965230111	0.015369139
Adck2	0.020487347	0.965230111	0.015369139
Sim1	0.018619511	0.965230111	0.015369139

Prss48	0.020649285	0.965230111	0.015369139
Otoa	0.017110382	0.965230111	0.015369139
Usp39	0.024590695	0.965230111	0.015369139
Cldn15	0.025298266	0.965230111	0.015369139
Kctd8	-0.024084849	0.965230111	0.015369139
Pkhd1l1	0.052314877	0.965230111	0.015369139
Eif2ak3	-0.034158942	0.965230111	0.015369139
Mpz	-0.03769282	0.965230111	0.015369139
Cabp4	-0.037161341	0.965230111	0.015369139
Sprr2a1	0.02664274	0.965230111	0.015369139
Drc7	0.022738859	0.965230111	0.015369139
Gnb1l	-0.016552586	0.965230111	0.015369139
Zfp442	0.017200209	0.965230111	0.015369139
Gjb6	0.031462144	0.965230111	0.015369139
Fig4	-0.030770364	0.965230111	0.015369139
Gosr2	0.012547765	0.965230111	0.015369139
Unc93a	-0.025189471	0.965230111	0.015369139
Chrna9	-0.019889943	0.965230111	0.015369139
Magee2	-0.013171471	0.965230111	0.015369139
Muc6	-0.035593267	0.965230111	0.015369139
Zcchc8	0.049753394	0.965230111	0.015369139
Zpbp	0.02580077	0.965230111	0.015369139
Phc3	-0.041320918	0.965230111	0.015369139
Dusp18	0.029570425	0.965230111	0.015369139
Slc17a2	-0.021734667	0.965230111	0.015369139
Med10	0.026109037	0.965230111	0.015369139
Skiv2l	0.021334394	0.965230111	0.015369139
Ubr5	-0.019923407	0.965230111	0.015369139
Arl16	0.026335327	0.965230111	0.015369139
Rab40b	-0.024832229	0.965230111	0.015369139
Ddx28	-0.02675295	0.965230111	0.015369139
Acot11	0.022059126	0.965230111	0.015369139
Ccr3	-0.061047921	0.965230111	0.015369139
Foxb1	0.017175001	0.965230111	0.015369139
FAM195B	-0.030996259	0.965230111	0.015369139
Hat1	0.02158424	0.965230111	0.015369139
Crhr2	0.019201196	0.965230111	0.015369139
Smg5	-0.020111943	0.965230111	0.015369139
Npat	0.012290616	0.965230111	0.015369139
Magohb	-0.037546133	0.965230111	0.015369139
Zfp46	0.022185713	0.965230111	0.015369139
Arcn1	0.020568655	0.965230111	0.015369139
Aspdh	-0.030622364	0.965230111	0.015369139
Topors	0.019904113	0.965230111	0.015369139
Ssb	-0.017781571	0.965230111	0.015369139
Tomm7	-0.018614796	0.965230111	0.015369139
Pkhd1	-0.029329384	0.965230111	0.015369139
Anapc5	0.025076317	0.965230111	0.015369139

Dnah2	0.033182844	0.965230111	0.015369139
Rgs20	0.01314504	0.965230111	0.015369139
Pdcd1lg2	0.026927755	0.965230111	0.015369139
Rars2	0.015244596	0.965230111	0.015369139
Slc9a3r2	-0.016448732	0.965230111	0.015369139
Th	0.026249403	0.965230111	0.015369139
Alox15	-0.021042168	0.965230111	0.015369139
Olfm5	-0.023877638	0.965230111	0.015369139
H1FOO	-0.029712816	0.965230111	0.015369139
Zfp873	0.017273435	0.965230111	0.015369139
Smim20	0.016936319	0.965230111	0.015369139
Dhx16	0.015987865	0.965230111	0.015369139
Pigh	-0.026495435	0.965230111	0.015369139
Krt6a	0.01823168	0.965230111	0.015369139
Eif4g2	0.020761549	0.965230111	0.015369139
Olfr91	0.018216343	0.965230111	0.015369139
Tph2	-0.022021239	0.965230111	0.015369139
Oaz2	0.039483158	0.965230111	0.015369139
Adh7	-0.050244826	0.965230111	0.015369139
Nrf1	0.023203739	0.965230111	0.015369139
Slc36a1os	-0.013788895	0.965230111	0.015369139
C87499	0.018036356	0.965230111	0.015369139
Pkd1l3	-0.018876222	0.965230111	0.015369139
Klhl24	-0.019108679	0.965230111	0.015369139
Ces1g	-0.029716885	0.965230111	0.015369139
Ndufa3	-0.015339384	0.965230111	0.015369139
Mgat4a	-0.031752294	0.965230111	0.015369139
Mfap1a	0.018674686	0.965230111	0.015369139
Tada1	-0.027445754	0.965230111	0.015369139
Scnm1	0.013409718	0.965230111	0.015369139
Zfp534	0.01885556	0.965230111	0.015369139
Ctdspl	-0.012975333	0.965230111	0.015369139
Klf10	-0.033732749	0.965230111	0.015369139
Ogfod3	-0.011313374	0.965230111	0.015369139
Serpinb6c	0.01091655	0.965230111	0.015369139
Dennd6a	0.0550239	0.965230111	0.015369139
Adam30	0.038823475	0.965230111	0.015369139
Zfp182	-0.009760235	0.965230111	0.015369139
MORF4L1E	0.031342683	0.965230111	0.015369139
ADCK3	0.054441454	0.965230111	0.015369139
Met	0.017842462	0.965230111	0.015369139
Nbl1	0.040105089	0.965230111	0.015369139
Gapdh	0.044732276	0.965230111	0.015369139
Rsl1	-0.010364073	0.965230111	0.015369139
Gmpr	0.009130533	0.965230111	0.015369139
Mroh2b	0.009047166	0.965230111	0.015369139
Aamp	0.009093287	0.965230111	0.015369139
Mogs	0.015859256	0.965293306	0.015340706

Klhl7	0.015926023	0.965360861	0.015310313
Slc35f5	0.050847034	0.965360861	0.015310313
Zcchc14	0.015782661	0.965360861	0.015310313
Ndufa1	-0.016855877	0.965360861	0.015310313
Jmjd8	-0.020927602	0.965360861	0.015310313
Erich1	0.017764561	0.965360861	0.015310313
Dpy30	0.034941616	0.965455041	0.015267946
Ppwd1	0.025792162	0.965539199	0.01523009
Klf6	0.028458217	0.965539199	0.01523009
Rp1l1	0.019657083	0.965539199	0.01523009
Spdya	0.035653972	0.965609125	0.015198639
Asph	-0.03197633	0.965609125	0.015198639
Ppcs	-0.026028268	0.965609125	0.015198639
Tekt2	-0.019464501	0.965609125	0.015198639
Tert	-0.020310309	0.965609125	0.015198639
Exosc7	0.06054235	0.965609125	0.015198639
Nubpl	0.017957175	0.965609125	0.015198639
Acyp2	-0.021296382	0.965609125	0.015198639
Prdm5	-0.020874667	0.965609125	0.015198639
Anks6	0.019474343	0.965609125	0.015198639
Suv39h1	-0.012488155	0.965609125	0.015198639
Amd2	-0.02236236	0.965609125	0.015198639
Ddx10	-0.033061492	0.965618978	0.015194207
Exosc9	0.035672988	0.965706993	0.015154624
Rrp9	-0.015035773	0.965745775	0.015137183
Vwa8	0.024467918	0.965745775	0.015137183
Specc1l	-0.02826081	0.965745775	0.015137183
C2cd4d	0.02572455	0.965745775	0.015137183
Pax9	-0.025664049	0.965745775	0.015137183
Cntnap3	-0.045830221	0.965796549	0.015114351
Glra3	0.03852858	0.965796549	0.015114351
Slc34a2	-0.017000685	0.965796549	0.015114351
Tmem45a	0.033671358	0.965796549	0.015114351
Bpifb9b	0.025806659	0.965796549	0.015114351
Akap3	-0.024394811	0.965796549	0.015114351
Sct	-0.022707599	0.965796549	0.015114351
Pagr1a	0.022145572	0.965796549	0.015114351
Cfap46	0.020438446	0.965796549	0.015114351
Spata31d	-0.016484996	0.965796549	0.015114351
Klre1	-0.01665285	0.965796549	0.015114351
Ddx18	0.041808537	0.965796549	0.015114351
Por	-0.020210874	0.965796549	0.015114351
Gsdma3	-0.017651058	0.965796549	0.015114351
Bnc1	-0.010617456	0.965796549	0.015114351
Smim4	-0.013882845	0.965796549	0.015114351
Sall2	-0.013090672	0.965796549	0.015114351
Rpl37	0.008740707	0.965796549	0.015114351
Nmb	0.035384197	0.965815463	0.015105846

Prr30	0.014877546	0.965958328	0.015041609
Slx4ip	0.022510563	0.965958328	0.015041609
Rnf25	0.015851478	0.966233752	0.014917796
Trpm3	-0.03986136	0.966233752	0.014917796
Elavl1	-0.154285291	0.966237829	0.014915963
Fam71b	-0.056478537	0.966237829	0.014915963
Pias3	0.067891155	0.966237829	0.014915963
Nhlh1	-0.038863263	0.966237829	0.014915963
Trim28	-0.03785796	0.966237829	0.014915963
Gpatch3	0.107589991	0.966237829	0.014915963
Ppp1r7	0.058068858	0.966237829	0.014915963
Mars2	-0.017372017	0.966237829	0.014915963
Robo1	0.017617334	0.966237829	0.014915963
Zfp397	0.035380429	0.966237829	0.014915963
Kbtbd3	0.020488325	0.966237829	0.014915963
Otc	0.064117342	0.966237829	0.014915963
Fip1l1	0.039877893	0.966237829	0.014915963
Lor	0.01961016	0.966237829	0.014915963
Tgs1	-0.039775109	0.966237829	0.014915963
Pcsk4	0.048005153	0.966237829	0.014915963
Zmynd10	-0.025046146	0.966237829	0.014915963
Vsig1	0.023249883	0.966237829	0.014915963
Dapl1	-0.012203493	0.966237829	0.014915963
L1td1	-0.041338973	0.966237829	0.014915963
Etv3	0.025731451	0.966237829	0.014915963
Zfp830	0.032301218	0.966237829	0.014915963
Nepn	0.034742604	0.966237829	0.014915963
AI481877	0.020544973	0.966237829	0.014915963
Sod1	-0.011434217	0.966237829	0.014915963
Cd209e	0.019173425	0.966237829	0.014915963
Vgll4	0.010898795	0.966237829	0.014915963
Fndc3a	-0.014918236	0.966237829	0.014915963
Tmprss2	-0.049934949	0.966237829	0.014915963
Ptgs2os2	-0.025026148	0.966237829	0.014915963
RGAG1	-0.01723946	0.966237829	0.014915963
Cox6a1	0.0134862	0.966237829	0.014915963
Heatr3	-0.012701679	0.966237829	0.014915963
Lyar	0.017224199	0.966237829	0.014915963
Adam28	0.022298375	0.966237829	0.014915963
Scrib	-0.018388842	0.966237829	0.014915963
Fabp12	0.016504771	0.966237829	0.014915963
Thoc2	-0.053081806	0.966237829	0.014915963
Znhit3	0.02220752	0.966237829	0.014915963
Prss42	0.012477209	0.966237829	0.014915963
Slc36a3	-0.015222946	0.966237829	0.014915963
Zbtb26	-0.022068295	0.966237829	0.014915963
Ddx54	-0.028735907	0.966237829	0.014915963
Ptrh2	0.023658319	0.966237829	0.014915963

Dpcd	-0.018912217	0.966237829	0.014915963
Il17ra	0.029955528	0.966237829	0.014915963
Gcdh	0.016064935	0.966237829	0.014915963
Cenpt	0.018924324	0.966237829	0.014915963
Wdr64	-0.032551797	0.966237829	0.014915963
Mmp1b	0.013110264	0.966238046	0.014915866
Vit	-0.017912488	0.966238046	0.014915866
Susd1	-0.01894092	0.966341507	0.014869366
Mcpt9	0.037335688	0.966355997	0.014862854
Erf	-0.0250065	0.966355997	0.014862854
Socs2	-0.012865804	0.966360315	0.014860913
Asb15	-0.02058412	0.966380765	0.014851723
Dppa2	0.011171262	0.966416587	0.014835624
Hemk1	0.01873897	0.966664804	0.014724094
Prss51	-0.023374254	0.966664804	0.014724094
Mrpl41	0.020683357	0.966664804	0.014724094
Cyp26c1	-0.018594934	0.966664804	0.014724094
Olfr1392	-0.016461857	0.966749931	0.01468585
Tecrl	-0.033570886	0.966749931	0.01468585
Arl6	-0.021695005	0.966749931	0.01468585
Cntnap5c	-0.016251519	0.966749931	0.01468585
Ptpra	0.037397413	0.966846905	0.014642289
Ism2	-0.01836601	0.966846905	0.014642289
Ganab	-0.020804608	0.967079464	0.014537839
Asxl2	-0.019946372	0.967079464	0.014537839
Pcdhb1	-0.024219112	0.967079464	0.014537839
Sec23b	0.064204924	0.967228118	0.014471087
Pomp	0.041759158	0.967228118	0.014471087
MIR1A-1H(	0.018801286	0.967228118	0.014471087
Hdgf	0.023133087	0.967228118	0.014471087
Tmprss11f	0.044765523	0.967228118	0.014471087
She	0.018966151	0.967228118	0.014471087
Vdac2	-0.015227302	0.967228118	0.014471087
Inhbb	-0.050290338	0.967228118	0.014471087
Zfp839	0.063962807	0.967228118	0.014471087
Nfil3	0.041896323	0.967228118	0.014471087
Phf7	-0.030970736	0.967228118	0.014471087
Lrrc75b	-0.016473477	0.967228118	0.014471087
Kdm4a	-0.023847393	0.967228118	0.014471087
Trak2	0.041128153	0.967228118	0.014471087
Zfhx3	-0.027845802	0.967228118	0.014471087
Tktl2	-0.026358138	0.967228118	0.014471087
Pmm1	-0.022609584	0.967228118	0.014471087
Sfi1	-0.037324012	0.967228118	0.014471087
Slc25a35	0.032916363	0.967228118	0.014471087
Ssu72	-0.016397065	0.967228118	0.014471087
Apln	-0.016253213	0.967228118	0.014471087
Ccnc	-0.042273355	0.967228118	0.014471087

Cdhr3	-0.02148611	0.967228118	0.014471087
Cyth1	0.017510153	0.967228118	0.014471087
Nt5c	-0.018064766	0.967228118	0.014471087
Zfyve19	0.014202776	0.967228118	0.014471087
Rab11fip4	0.017122899	0.967228118	0.014471087
Faap24	-0.019287201	0.967228118	0.014471087
Slc7a6	0.015206885	0.967228118	0.014471087
Prg4	0.051917991	0.967228118	0.014471087
Rps29	-0.008497982	0.967228118	0.014471087
Pou2f1	0.01976911	0.967228118	0.014471087
MKL1	0.008464684	0.967228118	0.014471087
Mus81	0.030173776	0.967283261	0.014446328
Mesp1	0.031254767	0.967283261	0.014446328
Sdhaf2	0.033499811	0.967285468	0.014445337
Hpd	0.016545225	0.967285468	0.014445337
Tnip3	-0.031616189	0.967285468	0.014445337
Slc13a2	0.0084418	0.967285468	0.014445337
Sbf2	0.056150347	0.967293394	0.014441778
Acvr1	-0.020935688	0.967293394	0.014441778
TMEM180	-0.024595481	0.967316536	0.014431388
Dydc1	-0.027550032	0.967423527	0.014383355
Rad21	0.076687343	0.967667363	0.014273906
Saa4	0.019992659	0.967667363	0.014273906
Zc3h11a	0.020603377	0.967667363	0.014273906
Tmf1	0.029546491	0.967667363	0.014273906
Mrpl10	-0.025439789	0.967667363	0.014273906
Vmn2r112	-0.014852072	0.967667363	0.014273906
Foxa3	0.017036482	0.967667363	0.014273906
Tmprss11	0.019604499	0.967667363	0.014273906
Cyp2b13	0.033320755	0.967667363	0.014273906
Krtap16-1	0.02958532	0.967667363	0.014273906
Slc22a2	-0.014957702	0.967667363	0.014273906
Ankrd27	0.035324272	0.967667363	0.014273906
Ugt2b38	0.012721027	0.967667363	0.014273906
Lrrc72	-0.027421617	0.967667363	0.014273906
Itgb6	-0.014159021	0.967667363	0.014273906
Wfdc6a	-0.035359995	0.967667363	0.014273906
Ccpg1os	0.030024081	0.967667363	0.014273906
Gale	0.023343985	0.967667363	0.014273906
Brwd3	-0.037255059	0.967667363	0.014273906
Fkbp5	-0.018014606	0.967667363	0.014273906
Pkd2	0.029310069	0.967667363	0.014273906
Psmd13	-0.017658393	0.967667363	0.014273906
Clk3	-0.029618378	0.967896793	0.014170949
Dcun1d3	-0.033094116	0.967896793	0.014170949
Srp72	-0.018189823	0.967896793	0.014170949
Srsf1	0.009476538	0.967896793	0.014170949
2-Mar	0.034332724	0.967896793	0.014170949

Pou5f2	-0.037471683	0.967906487	0.0141666
Ric8a	0.018109832	0.967906487	0.0141666
Ktn1	0.020209658	0.967906487	0.0141666
Them5	-0.020367339	0.967906487	0.0141666
Gnrh1	0.010026658	0.967906487	0.0141666
CCDC168	-0.008770427	0.967906487	0.0141666
Dnaaf2	-0.015525438	0.968245232	0.014014633
Tmc2	0.027964128	0.968245648	0.014014446
Dnd1	-0.077740638	0.968267244	0.01400476
Btbd18	0.02494516	0.968267244	0.01400476
Lcn12	-0.026084938	0.968267244	0.01400476
Ube2u	-0.025417729	0.968267244	0.01400476
Cisd3	0.018146752	0.968267244	0.01400476
Slc38a6	-0.058228742	0.968267244	0.01400476
Oxa1l	-0.025615305	0.968267244	0.01400476
Emc2	-0.045627018	0.968267244	0.01400476
Sin3b	-0.014676224	0.968267244	0.01400476
Zfp354a	0.050440692	0.968267244	0.01400476
Smad2	0.037123613	0.968267244	0.01400476
Acbd3	0.021289221	0.968267244	0.01400476
Ankrd36	-0.027058241	0.968267244	0.01400476
Elovl3	-0.015584283	0.968267244	0.01400476
Slc10a1	-0.018726462	0.968267244	0.01400476
Cftr	-0.027986744	0.968267244	0.01400476
Uba5	-0.018711801	0.968267244	0.01400476
Pdcd10	-0.011012521	0.968267244	0.01400476
Slc5a3	0.016507507	0.968267244	0.01400476
Slc16a11	0.017746314	0.968267244	0.01400476
Cyp3a57	-0.021592192	0.968267244	0.01400476
Bpi	-0.025629454	0.968267244	0.01400476
Mgat4e	0.010479255	0.968267244	0.01400476
Lrrc31	-0.01651482	0.968292708	0.013993339
Polr1e	0.020468685	0.968324914	0.013978894
Foxo4	-0.036113665	0.968353147	0.013966232
Nkx2-3	0.020360731	0.968353147	0.013966232
Dolpp1	-0.014514921	0.968353147	0.013966232
Cpsf3	-0.02736063	0.968353147	0.013966232
Ssh1	-0.01746206	0.968353147	0.013966232
Zbtb49	0.013501159	0.968353147	0.013966232
Sltm	-0.019335334	0.968353147	0.013966232
Slc6a14	0.018241054	0.968380165	0.013954114
Slc35g3	0.030282355	0.968481749	0.013908559
Otogl	-0.010783021	0.96860904	0.013851482
Thra	0.029034141	0.968625389	0.013844152
Wtap	0.102509014	0.968829674	0.013752568
Arxes2	0.080452678	0.968890596	0.013725259
Tmprss15	-0.039179806	0.968890596	0.013725259
Ap5z1	0.023056594	0.968890596	0.013725259

Pfdn1	0.012522229	0.968890596	0.013725259
Misp	0.050328475	0.968890596	0.013725259
Nkain2	-0.017451885	0.968890596	0.013725259
Rex2	-0.015491579	0.968890596	0.013725259
Klk14	0.013046916	0.968893908	0.013723775
Cacng5	0.015097253	0.969009716	0.013671868
Safb2	0.029516572	0.969025606	0.013664747
Aldh1a3	0.018918736	0.969025606	0.013664747
Btnl10	0.013401913	0.969025606	0.013664747
Cfap157	-0.029326895	0.969146726	0.013610467
Rnf20	0.018600831	0.969188807	0.01359161
Endog	0.01966982	0.969188807	0.01359161
Cyp2b23	-0.069218202	0.969188807	0.01359161
Chrna1	-0.019758967	0.969188807	0.01359161
Fbxl6	-0.012455013	0.969188807	0.01359161
Slc12a1	0.016185443	0.969188807	0.01359161
Pla2g4c	0.028557872	0.969188807	0.01359161
Foxp4	0.028831208	0.969188807	0.01359161
Nudt5	0.012775956	0.969188807	0.01359161
Chrm4	0.029044339	0.969188807	0.01359161
Dnttip1	0.018031333	0.969188807	0.01359161
Lrriq1	-0.041612038	0.969188807	0.01359161
Abcb5	-0.028455565	0.969188807	0.01359161
Ypel5	0.032036452	0.969188807	0.01359161
Vnn3	-0.044330042	0.969188807	0.01359161
Cyyr1	-0.055723807	0.969220065	0.013577604
Slc9a8	0.052312391	0.969220065	0.013577604
Ndufs5	-0.031311903	0.969220065	0.013577604
Slc6a18	-0.045038872	0.969220065	0.013577604
Gatb	-0.016831673	0.969220065	0.013577604
A	-0.014682264	0.969220065	0.013577604
TCTEX1D2	-0.033009175	0.969220065	0.013577604
Bloc1s2	0.01275265	0.969220065	0.013577604
Rpp38	-0.015576002	0.969220065	0.013577604
Rnf144a	-0.022757536	0.969220065	0.013577604
Snrpc	-0.022571824	0.969220065	0.013577604
Trmt13	-0.039649625	0.969220065	0.013577604
Tet2	-0.021291121	0.969220065	0.013577604
Agfg2	0.015592824	0.969220065	0.013577604
T	0.020487294	0.969220065	0.013577604
Wfdc8	-0.018739199	0.969220065	0.013577604
Kat6a	0.045262565	0.969220065	0.013577604
Clec2e	-0.015874625	0.969220065	0.013577604
Cysrt1	-0.016099165	0.969220065	0.013577604
Scn4b	-0.028928644	0.969220065	0.013577604
Atp1a4	-0.018301908	0.969220065	0.013577604
Aurkc	-0.042151135	0.969220065	0.013577604
Cntfr	0.022043367	0.969220065	0.013577604

Pip4k2a	-0.0231716	0.969220065	0.013577604
Acp1	-0.017532578	0.969220065	0.013577604
Pomt2	-0.025071125	0.969220065	0.013577604
D2WSU81I	-0.030693838	0.969220065	0.013577604
Abi1	0.01352482	0.969220065	0.013577604
Tchhl1	0.014635291	0.969220065	0.013577604
Larp4	-0.01956656	0.969220065	0.013577604
AES	-0.014335376	0.969220065	0.013577604
Foxp3	0.029996882	0.969220065	0.013577604
Dmkn	0.012121203	0.969302682	0.013540586
Mex3d	0.015286963	0.969302682	0.013540586
Frg1	-0.01745901	0.969369772	0.013510527
Dclre1a	-0.021117206	0.969395691	0.013498915
Unc50	-0.027770689	0.969407988	0.013493406
Hsf2	0.023129719	0.969407988	0.013493406
Mfsd3	-0.034772676	0.969683286	0.01337009
Fam90a1b	-0.012872684	0.969731825	0.013348351
BB114814	0.022427568	0.969741409	0.013344059
Ivl	0.027468979	0.969916034	0.013265861
Ptpn12	0.029913285	0.969916034	0.013265861
Cnga4	0.030520745	0.969916034	0.013265861
Art4	-0.018156263	0.969916034	0.013265861
Snx29	-0.023853011	0.969916034	0.013265861
Fhl4	-0.026523267	0.969916034	0.013265861
Trim43b	0.020999673	0.969916034	0.013265861
Mrpl9	0.012875306	0.969916034	0.013265861
Scnn1b	-0.022186883	0.969916034	0.013265861
Hey1	0.039339846	0.969916034	0.013265861
HIST1H1T	0.017884323	0.969916034	0.013265861
Prpf4	0.013721353	0.969916034	0.013265861
Cmc4	0.019650215	0.969916034	0.013265861
Uqcrq	-0.017079075	0.969916034	0.013265861
Tmem72	-0.035038172	0.969916034	0.013265861
Uroc1	-0.038432226	0.969916034	0.013265861
Wdr11	-0.014752653	0.969916034	0.013265861
Cyp4f40	-0.019907037	0.969916034	0.013265861
Wdr55	-0.011764139	0.969916034	0.013265861
Yipf2	0.020354548	0.969916034	0.013265861
Slc22a28	-0.058559534	0.969916034	0.013265861
Rnf145	-0.027009733	0.969916034	0.013265861
Rgs9bp	-0.018018709	0.969916034	0.013265861
PRAME	0.025880041	0.969916034	0.013265861
Mrpl1	0.022157323	0.969916034	0.013265861
Etf1	-0.018555226	0.969916034	0.013265861
Nudt10	-0.015997721	0.969916034	0.013265861
Cpn2	-0.019470229	0.969916034	0.013265861
Gpc1	-0.026092213	0.969916034	0.013265861
Nek11	-0.012769387	0.969916034	0.013265861

Clec12b	0.026758409	0.969916034	0.013265861
Kera	-0.020986967	0.969916034	0.013265861
Psma3	-0.026516896	0.969916034	0.013265861
Bivm	-0.016107308	0.969916034	0.013265861
Slc35f1	-0.012720452	0.969916034	0.013265861
Slc25a17	-0.012826449	0.969916034	0.013265861
Adtrp	0.018547373	0.969916034	0.013265861
Chpf2	0.017957529	0.969916034	0.013265861
Lrit1	0.03810997	0.969916034	0.013265861
Ceacam3	0.036865062	0.969916034	0.013265861
Aicda	-0.013582282	0.969916034	0.013265861
Tmem147	0.011388258	0.969916034	0.013265861
Rint1	0.010396965	0.969916034	0.013265861
Ncapd3	0.015617903	0.969916034	0.013265861
AI314278	-0.016868796	0.969916034	0.013265861
Cyp2j13	-0.013631622	0.969916034	0.013265861
Rd3	0.022857064	0.969975251	0.013239347
Otog	-0.01688738	0.970136818	0.013167013
Me2	0.016035882	0.970141568	0.013164887
Zfp385a	0.010421755	0.970141568	0.013164887
Slfn14	0.044887701	0.970141568	0.013164887
Catsperg2	-0.048390715	0.970209576	0.013134443
Zfp280c	-0.022878184	0.970209576	0.013134443
Apip	0.021616182	0.970209576	0.013134443
Tapt1	0.023915692	0.970209576	0.013134443
Clec4f	-0.01508481	0.970209576	0.013134443
FAM175B	-0.013992668	0.970209576	0.013134443
Cops6	0.010511119	0.970209576	0.013134443
Mettl7a1	-0.019938401	0.970209576	0.013134443
Gpn3	0.017208501	0.970209576	0.013134443
Dr1	0.034218589	0.97037342	0.013061108
Dnah5	0.020874341	0.970380535	0.013057924
Grhl2	-0.015294835	0.97039748	0.01305034
Lrrc57	0.011663902	0.970490812	0.013008572
Nobox	-0.033547228	0.9705101	0.012999941
Rtcb	0.047123717	0.9705101	0.012999941
Tmed4	-0.029222563	0.9705101	0.012999941
Kdf1	0.034925146	0.9705101	0.012999941
Adgrf3	-0.018216098	0.9705101	0.012999941
Tat	0.023071426	0.9705101	0.012999941
Uhrf1bp1	0.024779243	0.9705101	0.012999941
Crh	0.024105077	0.9705101	0.012999941
Ccdc68	-0.040550279	0.9705101	0.012999941
Slco1a6	0.016561273	0.9705101	0.012999941
Sdf2	-0.017945508	0.9705101	0.012999941
Tmem211	0.012974482	0.9705101	0.012999941
Tmem139	0.043106464	0.9705101	0.012999941
Krtap5-2	-0.019642481	0.9705101	0.012999941

Rwdd1	-0.027703667	0.9705101	0.012999941
Letmd1	0.050812035	0.9705101	0.012999941
Zfp593	0.02420574	0.9705101	0.012999941
Zbtb6	-0.015623239	0.9705101	0.012999941
Dnajc15	-0.01165148	0.9705101	0.012999941
Nrg2	0.015138409	0.9705101	0.012999941
Trim31	-0.011775543	0.9705101	0.012999941
Ptdss1	0.047374094	0.9705101	0.012999941
Tmem217	-0.011904112	0.970570594	0.012972871
Slc22a22	0.01608965	0.970665713	0.012930311
Loxl4	-0.015885677	0.970665713	0.012930311
Nat8f6	-0.017050269	0.970665713	0.012930311
Trim43a	-0.012530342	0.970665713	0.012930311
Zfp207	0.018241542	0.970706264	0.012912168
Foxred1	-0.042811952	0.970762499	0.012887009
Sla2	0.032242537	0.970762499	0.012887009
Pxylp1	-0.035928817	0.970762499	0.012887009
Eif6	-0.024717384	0.970762499	0.012887009
Slc2a6	0.017419961	0.970762499	0.012887009
Sox2ot	0.034130193	0.970762499	0.012887009
Tcp10a	0.030478109	0.970762499	0.012887009
Sbk2	-0.030996463	0.970859214	0.012843743
Atg16l2	0.034012799	0.970992353	0.01278419
Lrrc4b	0.023309336	0.970992353	0.01278419
Smug1	0.029068566	0.970992353	0.01278419
Psmd5	0.014615599	0.971018125	0.012772663
Speer4b	-0.167768153	0.971297249	0.012647841
Tubal3	0.017691671	0.971318761	0.012638223
Iqcf3	-0.057046599	0.971443357	0.012582517
Ralgps2	0.022585024	0.971554565	0.012532803
Zfp748	0.013099512	0.971554565	0.012532803
Clptm1l	-0.01239082	0.971554565	0.012532803
Ghrhr	-0.045677826	0.971588361	0.012517696
Olfr112	0.039726675	0.971588361	0.012517696
Oaz1	0.025690579	0.971588361	0.012517696
Mfsd6l	-0.025889515	0.971588361	0.012517696
Trib2	0.027618723	0.971588361	0.012517696
Magoh	-0.031834371	0.971588361	0.012517696
Gtf2ird1	0.01837806	0.971588361	0.012517696
Pkp3	-0.030209473	0.971588361	0.012517696
Atp5j2	-0.012767978	0.971588361	0.012517696
Tcf24	-0.050545933	0.971588361	0.012517696
Apoa5	0.01207455	0.971588361	0.012517696
Parp2	-0.01979844	0.971588361	0.012517696
Dsg1b	0.010626427	0.971588361	0.012517696
Runx2	0.011920027	0.971588361	0.012517696
Tubb2a	-0.014487028	0.971588361	0.012517696
Hoxa7	0.007723256	0.971588361	0.012517696

Cspg5	0.038372007	0.971624526	0.012501531
Tgm5	-0.085153047	0.971624526	0.012501531
Tmem263	0.160723607	0.971624526	0.012501531
Dnmt3l	-0.037166818	0.971624526	0.012501531
Cbx1	0.017294962	0.971624526	0.012501531
Dtd2	-0.032249126	0.971624526	0.012501531
Zfp324	-0.020130546	0.971624526	0.012501531
Sertad2	0.030542285	0.971624526	0.012501531
Impa1	-0.030979389	0.971624526	0.012501531
Dusp9	0.015743148	0.971624526	0.012501531
Pphln1	0.019013978	0.971624526	0.012501531
Htr6	-0.065521107	0.971624526	0.012501531
Tmc4	-0.014501649	0.971624526	0.012501531
Thumpd3	0.021348215	0.971624526	0.012501531
Emc6	0.011415749	0.971624526	0.012501531
Zfp317	0.036156218	0.971624526	0.012501531
Bsdc1	-0.012331292	0.971624526	0.012501531
Vpreb1	0.013439528	0.971624526	0.012501531
Eif3c	-0.012932899	0.971624526	0.012501531
Hoxd4	-0.008052569	0.971624526	0.012501531
Plin1	0.014403918	0.971624526	0.012501531
Cdh9	-0.014775605	0.971624526	0.012501531
Nts	-0.015196908	0.971850162	0.012400689
Nol12	0.075227256	0.971850162	0.012400689
F13b	0.038106875	0.972048715	0.012311969
Myog	0.070011511	0.972083878	0.01229626
Plekhh3	0.018426117	0.972083878	0.01229626
Mrgprb1	0.011366343	0.972083878	0.01229626
Lin28a	0.020208407	0.972107979	0.012285492
Foxd3	-0.0197871	0.972152861	0.012265441
Cops3	-0.021714543	0.972152861	0.012265441
Endod1	-0.010442439	0.972152861	0.012265441
Cadm4	0.023424883	0.972152861	0.012265441
Lipi	0.019306051	0.972152861	0.012265441
Vmn2r79	0.020953233	0.972152861	0.012265441
Zfp715	0.017998629	0.972152861	0.012265441
Rasl12	-0.008925085	0.972152861	0.012265441
Mtfmt	0.034028669	0.972432083	0.012140721
Clec4g	0.02006557	0.972462996	0.012126915
Gid8	-0.040132422	0.972462996	0.012126915
Cep68	0.020306379	0.972462996	0.012126915
Usp49	0.020572073	0.972462996	0.012126915
Vmn2r2	-0.013984173	0.972462996	0.012126915
Dyrk3	-0.015804615	0.972462996	0.012126915
Fap	0.014332138	0.972462996	0.012126915
Zc3h18	-0.017243947	0.972462996	0.012126915
Pan2	0.019338599	0.972500944	0.012109969
Chad	-0.034918514	0.972529747	0.012097106

Zfas1	0.020303654	0.972529747	0.012097106
Npm2	0.23278337	0.972980033	0.011896072
Ssr1	-0.053342052	0.972980033	0.011896072
Gdpd5	-0.051078648	0.972980033	0.011896072
Spink5	0.028750229	0.972980033	0.011896072
Grk4	-0.023938346	0.972980033	0.011896072
Ppp1r42	-0.022626316	0.972980033	0.011896072
Tmem126	-0.018756268	0.972980033	0.011896072
Ankrd7	-0.021411378	0.972980033	0.011896072
Cspp1	0.013792945	0.972980033	0.011896072
Pik3r4	-0.019778669	0.972980033	0.011896072
Fxn	0.016869026	0.972980033	0.011896072
Mbd1	0.012406708	0.972980033	0.011896072
Nisch	0.03619128	0.972980033	0.011896072
Rab17	0.023015961	0.972980033	0.011896072
Msh3	0.01372844	0.972980033	0.011896072
Slco2a1	0.018382348	0.972980033	0.011896072
Snx31	0.04407011	0.972980033	0.011896072
Sema3g	-0.018753073	0.972980033	0.011896072
Nol9	-0.01451736	0.972980033	0.011896072
Prnp	0.021684599	0.972980033	0.011896072
Med31	0.021904079	0.972980033	0.011896072
Epha3	0.01518285	0.972980033	0.011896072
Fam151a	0.02809573	0.972980033	0.011896072
Itpk1	-0.027616518	0.972980033	0.011896072
Ercc5	0.013482656	0.972980033	0.011896072
Ggt6	0.030567949	0.972980033	0.011896072
Slc6a2	-0.025583545	0.972980033	0.011896072
Muc4	0.013466336	0.972980033	0.011896072
Prmt1	0.019656524	0.972980033	0.011896072
Vmn2r5	0.016478663	0.972980033	0.011896072
Hacd3	0.012866736	0.972980033	0.011896072
Thada	0.017470464	0.973031685	0.011873017
Smc3	0.026843009	0.973031685	0.011873017
Phlpp1	0.125067894	0.973177149	0.011808097
Psma6	-0.01813869	0.973177149	0.011808097
Nadsyn1	0.017526791	0.973177149	0.011808097
Jund	-0.012920169	0.973177149	0.011808097
Hyal6	-0.020914478	0.973177149	0.011808097
Mfsd2b	0.009447808	0.973177149	0.011808097
Lrrc63	-0.013381544	0.973177149	0.011808097
Izumo1r	-0.016216178	0.973177149	0.011808097
Dmrtc2	0.04745121	0.973544475	0.011644203
Rasa1	0.013876557	0.973544475	0.011644203
Tex21	0.033240392	0.973544475	0.011644203
Ccl17	0.01246472	0.973544475	0.011644203
Bmp8b	0.026308179	0.973544475	0.011644203
Adh6b	0.016303992	0.973544475	0.011644203

Rbm12	-0.02575232	0.973544475	0.011644203
Glb1l3	0.011808631	0.973544475	0.011644203
Clpp	-0.016440807	0.973544475	0.011644203
Eps8l3	0.016739058	0.973544475	0.011644203
FAM173A	0.026977402	0.97364638	0.011598746
Adam29	-0.036934973	0.97367479	0.011586074
Rgr	0.03053913	0.97367479	0.011586074
Cfap44	0.020546534	0.97367479	0.011586074
Cox19	-0.028537183	0.97367479	0.011586074
Tmem190	-0.014572616	0.97367479	0.011586074
FAM129C	0.013297121	0.97367479	0.011586074
Smok3a	-0.023926262	0.97367479	0.011586074
Dner	0.009949045	0.97367479	0.011586074
Arl6ip4	-0.018545683	0.97367479	0.011586074
Brpf1	-0.01382994	0.97367479	0.011586074
Rala	0.080023907	0.973720841	0.011565535
SVS1	0.062028557	0.973720841	0.011565535
Tnxb	-0.078680676	0.973820694	0.011521001
Ppp3r2	-0.047370651	0.973820694	0.011521001
Ivd	-0.025226861	0.973820694	0.011521001
Tmem135	0.025942952	0.973820694	0.011521001
Rspry1	-0.03618752	0.973820694	0.011521001
Nek4	-0.017000075	0.973820694	0.011521001
Ewsr1	-0.011781661	0.973820694	0.011521001
Ufsp1	0.008859901	0.973820694	0.011521001
Prdm14	-0.023385545	0.973820694	0.011521001
Cnga1	0.017010921	0.973820694	0.011521001
Morc1	0.040775277	0.973899421	0.011485892
Phf11c	-0.007607515	0.97390191	0.011484783
Slc5a9	-0.013190951	0.973921729	0.011475944
Dmrt1	0.036153935	0.973921729	0.011475944
Ribc2	-0.042095876	0.973921729	0.011475944
Aurkaip1	0.020634816	0.973921729	0.011475944
Slc17a3	0.018810807	0.973951434	0.011462699
Irs1	-0.01372964	0.974116808	0.011388963
Snrpb	-0.024567118	0.974294002	0.011309971
Rpl38	0.027205094	0.974352746	0.011283786
H2-Eb2	-0.041974389	0.974352746	0.011283786
Raf1	-0.020677168	0.974352746	0.011283786
Pitx3	0.023554623	0.974352746	0.011283786
Eif2s3x	-0.011912557	0.974352746	0.011283786
Fgf11	0.023775528	0.974352746	0.011283786
Isy1	0.019028508	0.974352746	0.011283786
NDNL2	0.024024999	0.974352746	0.011283786
Ucp3	0.016519323	0.974352746	0.011283786
Slc27a3	-0.015816139	0.974352746	0.011283786
Slc22a15	0.020233196	0.974352746	0.011283786
Bpifb9a	-0.015212523	0.974352746	0.011283786

Osgep	-0.021159671	0.974352746	0.011283786
Rbm42	-0.016307672	0.974352746	0.011283786
Tmprss11g	-0.013259823	0.974352746	0.011283786
Gdf2	-0.011928682	0.974352746	0.011283786
Olfr898	0.041406122	0.974352746	0.011283786
Onecut2	0.019890164	0.974352746	0.011283786
Fam172a	0.014002528	0.974352746	0.011283786
Slc33a1	-0.011811654	0.974352746	0.011283786
Polr1a	-0.012153481	0.974352746	0.011283786
Prdx6b	-0.030642502	0.974352746	0.011283786
Trim38	0.0124087	0.974352746	0.011283786
Nasp	-0.007473642	0.974352746	0.011283786
Lrrc66	-0.017289786	0.974504313	0.011216234
Slc23a1	0.025379952	0.97467701	0.011139278
Slc48a1	-0.007711775	0.974752206	0.011105773
H2-Q1	0.034998189	0.974776864	0.011094787
BRE	-0.025085318	0.974776864	0.011094787
Iffo1	-0.014261165	0.974776864	0.011094787
Vmn2r96	0.012606007	0.974776864	0.011094787
Taf1d	-0.038748535	0.974789375	0.011089213
Zcchc4	0.025291572	0.974789375	0.011089213
Ace3	-0.013221784	0.974789375	0.011089213
Pcdhgb8	0.018221494	0.974789375	0.011089213
Snrpd3	0.024218068	0.974789375	0.011089213
Nat9	-0.014625806	0.974789375	0.011089213
Zfp580	0.007909736	0.974789375	0.011089213
FAM120AC	-0.019872492	0.974876433	0.011050428
Rnaseh2a	-0.012298024	0.974946652	0.011019148
Trpm7	0.01420877	0.974946652	0.011019148
Pum3	-0.020366887	0.974946652	0.011019148
Tmprss13	0.011220016	0.974946652	0.011019148
Tmem161	-0.015443192	0.975131415	0.010936852
Tas1r2	-0.017254619	0.975249095	0.010884444
Cox17	0.01234815	0.975249095	0.010884444
Tbc1d13	-0.034010985	0.975249095	0.010884444
Vipas39	-0.016430806	0.975345851	0.010841359
Nt5c1b	-0.015478651	0.975345851	0.010841359
Cpne6	-0.016235648	0.975465339	0.010788158
Hirip3	0.018033841	0.975465339	0.010788158
Scml4	-0.016354541	0.975534118	0.010757537
Man2a2	-0.010712161	0.975534118	0.010757537
Adrm1	0.01911979	0.975534118	0.010757537
Mrpl47	0.024543785	0.975534118	0.010757537
Tsg101	-0.016755445	0.975534118	0.010757537
Prmt3	-0.008022435	0.975534118	0.010757537
Sbk1	0.022609545	0.975534327	0.010757444
Tshz1	0.01318515	0.975534327	0.010757444
Spdye4b	-0.015123693	0.975534327	0.010757444

Ccdc43	0.044971438	0.97557267	0.010740375
C1galt1	0.021922832	0.97557267	0.010740375
Mpv17	-0.026623161	0.97557267	0.010740375
Ninl	-0.015797293	0.97557267	0.010740375
Lonp1	-0.033098589	0.97557267	0.010740375
Eif2ak4	0.012784619	0.97557267	0.010740375
Tinagl1	0.02464507	0.97557267	0.010740375
Art3	0.012048697	0.97557267	0.010740375
BC005537	0.016191996	0.97557267	0.010740375
Aknaos	-0.009911314	0.97557267	0.010740375
Uqcr10	0.015195186	0.97557267	0.010740375
Dgkb	0.012533068	0.97557267	0.010740375
Ugt2b34	0.013990251	0.97557267	0.010740375
Otos	-0.022913088	0.975671719	0.010696283
Gprc6a	-0.037907503	0.975671719	0.010696283
Coasy	0.016979963	0.975671719	0.010696283
Polr1c	-0.012601778	0.975671719	0.010696283
Lipt2	-0.047097033	0.975671719	0.010696283
Pfpl	-0.016384518	0.975671719	0.010696283
Ttf1	0.016230795	0.975671719	0.010696283
Ccdc154	-0.011648938	0.975671719	0.010696283
Commd7	-0.021518256	0.975671719	0.010696283
Tmem102	0.026476456	0.975671719	0.010696283
Gbp2b	0.007566558	0.975671719	0.010696283
Msrb2	-0.031566058	0.975805059	0.010636935
WBSCR27	-0.018000067	0.975864482	0.010610488
Mcat	0.026964887	0.975877331	0.01060477
Arid5b	-0.017603654	0.975877331	0.01060477
Nt5c3b	-0.015442903	0.975887419	0.010600281
Lrrn1	0.013369761	0.975887419	0.010600281
Sphk2	-0.029240441	0.976021689	0.010540532
Opalin	-0.048503209	0.976079214	0.010514936
D1Pas1	-0.016337952	0.976079214	0.010514936
Rbfa	0.011444442	0.976079214	0.010514936
Slc27a1	0.025840048	0.976079214	0.010514936
Malrd1	-0.030872123	0.976079214	0.010514936
Recql4	0.019321379	0.976079214	0.010514936
Aldh1a2	0.014698987	0.976079214	0.010514936
Psg29	0.013609849	0.976079214	0.010514936
Mre11a	0.02392569	0.976079214	0.010514936
Fzd5	0.007723973	0.976079214	0.010514936
Glrx3	0.015862042	0.976097514	0.010506793
Sphk1	0.007131923	0.976097514	0.010506793
St6galnac	-0.025124765	0.97610635	0.010502862
Pabpc6	0.012687566	0.97610635	0.010502862
Adamdec1	0.014390154	0.976111583	0.010500534
Usp1	-0.05718646	0.976191963	0.010464772
Plekhh1	-0.02646549	0.976191963	0.010464772

Tpr	0.046337597	0.976195532	0.010463184
Slc22a30	0.016071229	0.976195532	0.010463184
Dnah14	0.040350197	0.976195532	0.010463184
Isca2	0.011183251	0.976195532	0.010463184
Btg4	-0.016765592	0.976195532	0.010463184
Lrwd1	0.013406341	0.976195532	0.010463184
Bcl2l1	0.0257028	0.976737986	0.010221922
Zkscan6	-0.016782292	0.976737986	0.010221922
Mecr	-0.022789195	0.976737986	0.010221922
Robo4	-0.011261199	0.976737986	0.010221922
Snhg7os	0.019883845	0.976737986	0.010221922
Sharpin	0.038668576	0.976737986	0.010221922
Smad6	-0.015339832	0.976737986	0.010221922
Trmt1l	-0.012192697	0.976737986	0.010221922
Siah1a	0.044221812	0.976737986	0.010221922
Lhx4	-0.008790623	0.976737986	0.010221922
Nmt1	-0.021538322	0.976737986	0.010221922
Cldn4	0.015309395	0.976737986	0.010221922
Psmd11	-0.018143154	0.976916941	0.010142359
Spata31d1	-0.011495214	0.976916941	0.010142359
Fmo9	0.018882356	0.976938321	0.010132855
Utp14a	0.015280192	0.976938321	0.010132855
Kcne1	0.014630183	0.976938321	0.010132855
Rfxank	-0.011772153	0.976970214	0.010118677
Kctd7	-0.011894798	0.976970214	0.010118677
Pusl1	-0.012364316	0.977168529	0.010030528
Sim2	0.079965554	0.977220354	0.010007496
Ran	0.036550057	0.977220354	0.010007496
Zcchc7	-0.019124253	0.977220354	0.010007496
Ankrd22	0.017922186	0.977220354	0.010007496
Kdsr	-0.018878847	0.977220354	0.010007496
Slc4a9	0.01456219	0.977220354	0.010007496
PEO1	0.027461122	0.977220354	0.010007496
Tkt	0.015727419	0.977220354	0.010007496
Sar1a	0.018247442	0.977309954	0.009967678
Khdrbs2	-0.02356625	0.977309954	0.009967678
Ypel1	-0.013300478	0.977309954	0.009967678
Arhgef28	0.068969786	0.977316819	0.009964627
Psme4	0.018868474	0.977316819	0.009964627
Tyr	-0.011020784	0.977316819	0.009964627
Rnf19b	0.018803122	0.977316819	0.009964627
Mtx1	0.021229445	0.977368393	0.00994171
Ercc1	0.041829403	0.977368393	0.00994171
Serpina1f	0.021487148	0.977368393	0.00994171
Krt78	0.015063635	0.977368393	0.00994171
Reck	-0.010839581	0.977368393	0.00994171
Ap3b1	-0.018652765	0.977368393	0.00994171
Cd19	-0.010680057	0.977454386	0.0099035

Zfp961	-0.014788378	0.977454386	0.0099035
Nav1	0.014652379	0.977454386	0.0099035
Eif4b	0.019560752	0.977454386	0.0099035
Sycp2	-0.030027784	0.977528175	0.009870716
U2af2	0.021868685	0.977528175	0.009870716
Fastkd5	-0.046405962	0.977528175	0.009870716
Ppp2r5a	0.02242924	0.977598741	0.009839366
Acaa1b	0.018497784	0.977598741	0.009839366
Eef2	-0.013079763	0.977693633	0.009797213
Ube2a	0.0181115	0.977805726	0.009747424
Fbxo8	0.016096809	0.977805726	0.009747424
Ccdc146	0.016060287	0.977842598	0.009731047
Cdc37	0.019532431	0.977842598	0.009731047
Rpl9	-0.016657722	0.977842598	0.009731047
Ovol1	0.030729851	0.977968197	0.009675268
Trim67	0.008919497	0.977968197	0.009675268
Stk38l	0.01612164	0.978094293	0.009619275
Vwa2	-0.017945639	0.978178265	0.009581992
Ndufb11	-0.038704092	0.978224749	0.009561354
Ptchd3	0.016455544	0.978224749	0.009561354
Tmem80	0.011903321	0.978224749	0.009561354
Rnf207	-0.037586409	0.978224749	0.009561354
Sprr2a2	-0.017524407	0.978224749	0.009561354
Timmdc1	0.012881099	0.97826173	0.009544936
PACRGL	0.011888578	0.97826173	0.009544936
Kif27	0.024597992	0.97826173	0.009544936
Arid1a	-0.010488272	0.97826173	0.009544936
Mpp4	-0.013119221	0.97826173	0.009544936
Rpgr	-0.017252795	0.978404859	0.009481399
Ppfia4	-0.020481999	0.978508837	0.009435248
AU040320	0.018799571	0.978572104	0.009407169
Gp6	0.02359077	0.978572104	0.009407169
Gucy2d	0.020663893	0.978572104	0.009407169
Fis1	-0.009731916	0.978572104	0.009407169
Smndc1	0.011277919	0.978572104	0.009407169
Mfsd12	-0.015512321	0.978572104	0.009407169
Ceacam19	-0.018727285	0.978583128	0.009402276
Tmem184	-0.013435585	0.978583128	0.009402276
Fgd5	0.03288221	0.978596842	0.00939619
Bysl	0.018777879	0.978596842	0.00939619
Rfx6	0.034228406	0.978596842	0.00939619
Yy1	0.027723693	0.978596842	0.00939619
Tmem170	0.012250699	0.978596842	0.00939619
Ccin	0.015286373	0.978596842	0.00939619
T2	0.012258785	0.978596842	0.00939619
Uck1	-0.020904654	0.978596842	0.00939619
Rbmxl1	0.026960471	0.978596842	0.00939619
Alg13	-0.012838992	0.978596842	0.00939619

Bfsp1	0.012165691	0.978673419	0.009362207
Clptm1	-0.014310752	0.97868086	0.009358905
Zfp647	-0.04342917	0.978779275	0.009315235
Fcrl5	0.031155111	0.978779275	0.009315235
Foxd4	-0.028598595	0.978779275	0.009315235
Ybx2	-0.066065192	0.978779275	0.009315235
Spata17	0.02085672	0.978779275	0.009315235
Rbbp4	-0.014875543	0.978779275	0.009315235
TMEM8	0.039087354	0.978779275	0.009315235
Clca3a2	0.01883846	0.978779275	0.009315235
Tmem109	-0.012500001	0.978779275	0.009315235
Cul4b	-0.016499796	0.978779275	0.009315235
Mfrp	-0.022771365	0.978779275	0.009315235
Zfp707	0.012964969	0.978779275	0.009315235
Kat8	0.023986622	0.978779275	0.009315235
Sdhb	0.015642004	0.978779275	0.009315235
Lman2l	0.012075568	0.978779275	0.009315235
Dnah7a	-0.016835934	0.978779275	0.009315235
Zfp35	-0.007787279	0.978779275	0.009315235
Il9r	-0.016035491	0.978801702	0.009305284
Nsmaf	0.015873607	0.978801702	0.009305284
Prss33	-0.022175414	0.978801702	0.009305284
Zfp942	-0.016416132	0.978801702	0.009305284
Fut7	0.018644945	0.978825182	0.009294866
Hoxa3	0.013750422	0.978867849	0.009275936
Ccdc59	-0.027800139	0.978881223	0.009270002
Vars2	-0.011395244	0.978881223	0.009270002
Zfp84	-0.016224912	0.978881223	0.009270002
Smarcb1	0.006703879	0.978881223	0.009270002
Zic5	-0.031673466	0.978904892	0.009259501
Glyat	0.030043927	0.978973082	0.009229249
Olfr536	0.024968199	0.979417313	0.009032223
Kpnb1	-0.04073769	0.979417313	0.009032223
Fam53c	0.013332931	0.979417313	0.009032223
Nebl	-0.029443364	0.979501485	0.008994901
Irx4	0.018824957	0.979501485	0.008994901
Mxd1	0.010084518	0.979501485	0.008994901
Rnf19a	0.032866427	0.979543815	0.008976133
Gbe1	-0.017907458	0.979560407	0.008968777
Rbm48	0.012515421	0.979644912	0.008931313
Zfp654	0.168354989	0.979781099	0.008870943
Nfib	0.037863148	0.979781099	0.008870943
Map3k6	0.008855466	0.979884798	0.00882498
Slc13a2os	0.044261547	0.979894508	0.008820676
Ube2j2	0.012976657	0.979900559	0.008817994
Klf14	0.014691557	0.979900559	0.008817994
Triqk	0.020066918	0.979900559	0.008817994
AY512915	0.015238457	0.979900559	0.008817994

Lamb3	0.020422443	0.979900559	0.008817994
Ccdc71	-0.045367435	0.979921344	0.008808783
Clca2	0.014202029	0.979921344	0.008808783
Lrrc28	0.015879349	0.979921344	0.008808783
Tsfm	-0.027192963	0.979921344	0.008808783
Kmo	0.008591375	0.979921344	0.008808783
Chchd2	-0.030181899	0.980114963	0.008722981
Trp53tg5	-0.026652455	0.980114963	0.008722981
Zbtb43	-0.042845287	0.980114963	0.008722981
Gpsm1	-0.031638459	0.980114963	0.008722981
Zscan10	-0.026040662	0.980114963	0.008722981
Toe1	-0.034281463	0.980114963	0.008722981
Gstz1	-0.034480251	0.980114963	0.008722981
Stard7	0.070955431	0.980114963	0.008722981
Nr1h4	0.015102265	0.980114963	0.008722981
Klf8	0.023842921	0.980114963	0.008722981
Rbm14	-0.011722975	0.980114963	0.008722981
Ift52	-0.017599528	0.980114963	0.008722981
Akt1s1	0.011833375	0.980114963	0.008722981
Ngf	0.013328738	0.980114963	0.008722981
Psmc4	-0.013147806	0.980114963	0.008722981
Emc9	-0.012593251	0.980114963	0.008722981
Cbx4	0.011756737	0.980114963	0.008722981
Trpc2	0.014708093	0.980114963	0.008722981
Vmn2r47	-0.016837513	0.980114963	0.008722981
Rnf34	0.026734241	0.980114963	0.008722981
Stx4a	0.010020128	0.980114963	0.008722981
Cldnd1	0.015777289	0.980114963	0.008722981
Clrn1	0.032051062	0.980114963	0.008722981
Serbp1	0.011475347	0.980114963	0.008722981
Egf	-0.026507513	0.980114963	0.008722981
Ypel2	0.021269619	0.980114963	0.008722981
Slain1	0.014724225	0.980114963	0.008722981
Map3k7	-0.024381619	0.980114963	0.008722981
Slc29a4	0.010402456	0.980114963	0.008722981
Rasd2	-0.008475685	0.980114963	0.008722981
Dsg3	0.014432865	0.980114963	0.008722981
PAPD4	0.028775063	0.980114963	0.008722981
Ttll10	-0.01884314	0.980114963	0.008722981
Dsel	0.021124321	0.980114963	0.008722981
Enthd1	-0.009366546	0.980114963	0.008722981
Dnmt3aos	-0.008945202	0.980114963	0.008722981
Gja8	-0.021633608	0.980114963	0.008722981
Arhgap24	-0.006547216	0.980114963	0.008722981
Qrfp	-0.009707581	0.980130144	0.008716254
Clcn5	-0.011206204	0.980130144	0.008716254
Ttll4	0.018869948	0.980130144	0.008716254
Med7	-0.008147795	0.980130144	0.008716254

Smc1b	0.036045845	0.980136628	0.008713381
Tmem230	0.046104332	0.980207087	0.008682162
Psapl1	-0.012897687	0.980217324	0.008677626
Arx	-0.014978719	0.980217324	0.008677626
Syde1	-0.040269949	0.980288124	0.008646259
Zc3hav1l	-0.033568286	0.980288124	0.008646259
Kxd1	0.017008865	0.980288124	0.008646259
Tcaim	0.020384355	0.980288124	0.008646259
Nfs1	0.021324718	0.980288124	0.008646259
Wee2	-0.026803456	0.980288124	0.008646259
Utp3	-0.016865641	0.980288124	0.008646259
GRASP	-0.017922058	0.980288124	0.008646259
Rap2a	-0.010340109	0.980288124	0.008646259
Fah	-0.017281335	0.980288124	0.008646259
Rras2	-0.021526565	0.980288124	0.008646259
Herpud2	-0.038376156	0.980485861	0.008558665
Nxf1	0.017653921	0.980485861	0.008558665
Rbm17	0.015848919	0.980485861	0.008558665
Cops5	-0.038906994	0.980485861	0.008558665
Get4	0.023498484	0.980485861	0.008558665
Frs3os	0.015826375	0.980485861	0.008558665
Epb42	0.014235926	0.980485861	0.008558665
Dpf2	-0.040783361	0.980485861	0.008558665
Kcnq1ot1	0.011241349	0.980485861	0.008558665
Cyp4f39	-0.012191582	0.980485861	0.008558665
Arf1	0.01062466	0.980485861	0.008558665
Enho	0.009149631	0.980485861	0.008558665
Dmap1	-0.018847001	0.980485861	0.008558665
Gsta2	-0.010996894	0.980485861	0.008558665
Cyp2a5	-0.01171228	0.980485861	0.008558665
Aifm1	-0.026750543	0.980485861	0.008558665
GBAS	0.009871454	0.980485861	0.008558665
SMEK2	0.018580113	0.980485861	0.008558665
Cdh26	0.006447187	0.980485861	0.008558665
Acy1	0.021039217	0.980503867	0.008550689
Tgm7	-0.017272761	0.980503867	0.008550689
Ctr9	0.033005093	0.980503867	0.008550689
Dnal4	0.015101344	0.980579649	0.008517125
Itih5l-ps	0.015068181	0.980654113	0.008484146
Kel	0.050887055	0.980654113	0.008484146
Stoml2	0.011467978	0.980698739	0.008464383
Ptprq	0.030740432	0.980725521	0.008452523
Ankar	-0.021679964	0.980965354	0.008346331
Prpf39	-0.029667514	0.980977556	0.008340929
Tiam2	0.042892782	0.980987388	0.008336576
Slx1b	0.01252524	0.980990503	0.008335197
Trim41	0.017371058	0.980990503	0.008335197
Stmnd1	0.009466041	0.980990503	0.008335197

Impdh2	0.0117709	0.980990503	0.008335197
Flnb	-0.022093522	0.980990503	0.008335197
Smad4	-0.024728176	0.980990503	0.008335197
Pi16	-0.014240305	0.980990503	0.008335197
Med20	0.054952529	0.980990503	0.008335197
Tpk1	0.012345484	0.980990503	0.008335197
Tm4sf4	0.010076509	0.980990503	0.008335197
Kdm8	-0.008994548	0.980990503	0.008335197
Tial1	0.012857125	0.980990503	0.008335197
Mrap2	-0.015299324	0.980990503	0.008335197
Tnfsf15	-0.019118246	0.980990503	0.008335197
Bbip1	0.006487981	0.980990503	0.008335197
Cfap100	-0.01571932	0.981144715	0.008266931
Fat2	-0.02135556	0.981144715	0.008266931
Smurf1	-0.017441941	0.981144715	0.008266931
Prkdc	-0.010901074	0.981144715	0.008266931
Cope	0.0591106	0.981288378	0.008203345
Polr2l	-0.026857681	0.981344834	0.00817836
Bend3	-0.026273402	0.981432025	0.008139775
Hnrnpll	-0.019158118	0.981432025	0.008139775
Mei4	0.018414991	0.981432025	0.008139775
Fam83a	0.022469839	0.981432025	0.008139775
Man1b1	-0.022404386	0.981432025	0.008139775
Rxfp3	0.01700633	0.981432025	0.008139775
Cul4a	-0.01728296	0.981432025	0.008139775
Perm1	0.044027981	0.981432025	0.008139775
Fbxl4	0.018585133	0.981432025	0.008139775
Hdac3	0.020508709	0.981432025	0.008139775
Hs3st6	0.028892469	0.981432025	0.008139775
Psmd4	0.034806852	0.981432025	0.008139775
Calml3	-0.015088105	0.981432025	0.008139775
Bahd1	0.013595315	0.981432025	0.008139775
Ip6k2	-0.01635964	0.981432025	0.008139775
Gtf2e2	0.016907547	0.981432025	0.008139775
Tbcc	0.056276326	0.981432025	0.008139775
Psmb1	0.022686953	0.981432025	0.008139775
Dguok	0.017874791	0.981432025	0.008139775
Cog2	-0.023547928	0.981432025	0.008139775
Cacng1	0.023696045	0.981432025	0.008139775
Rnaseh1	-0.015399663	0.981432025	0.008139775
Cul7	0.021898531	0.981432025	0.008139775
Gal3st2	0.011434138	0.981432025	0.008139775
Art1	0.017602998	0.981432025	0.008139775
Trh	0.014391596	0.981432025	0.008139775
Ncln	0.041176122	0.981432025	0.008139775
Tbc1d5	-0.018949297	0.981432025	0.008139775
Ccdc85b	-0.009973424	0.981432025	0.008139775
Ephb1	0.012131499	0.981432025	0.008139775

Kif16b	0.011309415	0.981432025	0.008139775
Mrps18b	-0.021412702	0.981432025	0.008139775
Zfp263	0.014612745	0.981432025	0.008139775
Iars2	-0.013024947	0.981432025	0.008139775
Gabpb1	-0.023968046	0.981432025	0.008139775
Ap1s1	0.012635181	0.981432025	0.008139775
Cdk11b	0.028433595	0.981432025	0.008139775
Erich5	0.019131336	0.981432025	0.008139775
Klkb1	0.011755226	0.981432025	0.008139775
Cpne8	0.013669151	0.981432025	0.008139775
Lsm14a	-0.008926574	0.981432025	0.008139775
Bspry	0.018399273	0.981432025	0.008139775
Nploc4	0.01180469	0.981432025	0.008139775
Dhrs7b	0.009810562	0.981432025	0.008139775
Irx3os	-0.033197255	0.981432025	0.008139775
Acp6	-0.031490077	0.981432025	0.008139775
Amigo3	-0.011257823	0.981432025	0.008139775
Sult1c1	-0.006548682	0.981432025	0.008139775
Ssrp1	-0.00952066	0.981432025	0.008139775
4-Sep	0.012615278	0.981432025	0.008139775
Hgh1	-0.014111351	0.981432025	0.008139775
Mfsd5	-0.01544122	0.981432025	0.008139775
Ankdd1a	0.011739758	0.981432025	0.008139775
Acan	0.021618477	0.981432025	0.008139775
Golph3	-0.020026954	0.981461865	0.00812657
Cox4i2	0.026349215	0.981461865	0.00812657
Ttc8	-0.021699788	0.981461865	0.00812657
Dclk2	-0.014249305	0.981461865	0.00812657
Btnl9	-0.029216266	0.981461865	0.00812657
Folr1	0.016642218	0.981461865	0.00812657
Srf	-0.014579805	0.981461865	0.00812657
Hnf4a	0.012704593	0.981461865	0.00812657
Dnajc12	-0.010035279	0.981461865	0.00812657
Kdm1a	0.015108036	0.981461865	0.00812657
TBRG3	-0.019251236	0.981461865	0.00812657
Psmg2	0.00644622	0.981461865	0.00812657
Ehmt1	0.026339363	0.981588515	0.008070532
Capn8	-0.020219479	0.981588515	0.008070532
Sec23ip	-0.019082751	0.981588515	0.008070532
Lrrc40	-0.014612336	0.981588515	0.008070532
Trmt2b	-0.011397079	0.981588515	0.008070532
Mroh5	0.016119349	0.981588515	0.008070532
Rag1	-0.006903466	0.981588515	0.008070532
Zfp937	0.01377623	0.981648303	0.00804408
Taf5l	-0.028772214	0.981778823	0.00798634
Mpnd	-0.026829537	0.981778823	0.00798634
Naa30	-0.013193426	0.981778823	0.00798634
Lamc3	0.012324854	0.981778823	0.00798634

FUK	0.014698679	0.981778823	0.00798634
Ces5a	0.016345471	0.981881495	0.007940925
Popdc3	0.015845435	0.981881495	0.007940925
Exo5	-0.012426264	0.981881495	0.007940925
Zfp618	0.012024965	0.981881495	0.007940925
Amacr	-0.011239407	0.981881495	0.007940925
Padi6	-0.009592142	0.981881495	0.007940925
Ercc3	-0.014702546	0.981881495	0.007940925
Patl2	0.022633779	0.981881495	0.007940925
Kcnh8	0.007182704	0.981881495	0.007940925
Asgr1	-0.015837494	0.981995942	0.007890307
CIRH1A	0.013521732	0.981995942	0.007890307
Krt87	0.01074468	0.981995942	0.007890307
Gjb4	0.028500631	0.98206769	0.007858577
Dda1	0.053033144	0.98206769	0.007858577
CCDC67	0.011454956	0.982171642	0.007812609
Arid3b	-0.029950161	0.982174827	0.007811201
Macc1	0.014135324	0.982196753	0.007801506
Ppia	0.017076873	0.982274373	0.007767186
Dmrtc1a	-0.023966872	0.98229762	0.007756908
Maob	0.020859413	0.98229762	0.007756908
Tgm6	-0.013216663	0.98229762	0.007756908
Cdh11	0.011933501	0.98229762	0.007756908
Dync2li1	-0.009960296	0.98229762	0.007756908
Pglyrp3	0.021341201	0.982342355	0.00773713
Bcr	0.01040361	0.982342355	0.00773713
Cnppd1	-0.011588531	0.982342355	0.00773713
Flot1	-0.011385499	0.982342355	0.00773713
Gper1	-0.01910969	0.982342355	0.00773713
Slc35c2	-0.023933241	0.982416473	0.007704364
Slc6a12	-0.013459601	0.982416473	0.007704364
DUXBL3	-0.020441845	0.98246123	0.007684579
Poteg	-0.011608834	0.982485241	0.007673965
Rmdn2	-0.024880442	0.982485241	0.007673965
Eif1b	-0.022293857	0.982499113	0.007667833
Oxct1	-0.021712508	0.982676444	0.007589454
Ccdc28b	-0.014239855	0.982842971	0.007515864
Amy2a2	0.013281217	0.982914492	0.007484262
Ugt1a1	0.008400983	0.9830005	0.007446261
Eif3j1	-0.019966436	0.983098304	0.007403053
Abca13	-0.008523367	0.983098304	0.007403053
Bud13	-0.014694953	0.983333286	0.00729926
Zfp626	0.02545372	0.983351183	0.007291355
Capn13	0.018004664	0.983351183	0.007291355
Strap	0.010662309	0.983351183	0.007291355
Trp53i11	0.023688612	0.983432835	0.007255296
Caln1	-0.010343454	0.983432835	0.007255296
Bves	0.022718988	0.983460778	0.007242956

Ttc6	-0.006159885	0.983531688	0.007211643
Creb3	-0.013001224	0.983573218	0.007193305
Tbx5	0.033391553	0.98358331	0.007188849
Wdr83os	-0.013387311	0.983595402	0.00718351
Prkra	0.012407572	0.983595402	0.00718351
Cyb5d2	-0.014016739	0.983700907	0.007136928
Rnpc3	-0.0185082	0.983700907	0.007136928
Naa15	-0.017084203	0.983700907	0.007136928
Ermap	0.011716831	0.983700907	0.007136928
Stx5a	0.027063129	0.983875341	0.007059924
Bricd5	0.016240244	0.98390505	0.00704681
Wdr6	0.015332148	0.98390505	0.00704681
Disc1	-0.013722936	0.984043461	0.00698572
Fgf3	0.011825839	0.984126481	0.006949082
Trpc7	-0.014078029	0.984252473	0.006893485
Apex1	-0.017551495	0.984252473	0.006893485
Spata2l	-0.01737805	0.984252473	0.006893485
Fntb	0.007157707	0.984252473	0.006893485
Cbln3	-0.014193701	0.984284788	0.006879227
Wdr25	0.059749576	0.984284788	0.006879227
Tex19.1	0.013636029	0.98429637	0.006874117
Rp1	0.043648812	0.984437161	0.006812001
Faf2	-0.018247385	0.984437161	0.006812001
Foxn1	-0.012379525	0.984437161	0.006812001
Btbd1	-0.026999949	0.984437161	0.006812001
Heatr1	-0.012353033	0.984437161	0.006812001
Zfp212	-0.007920878	0.984437161	0.006812001
C1ql4	0.011157379	0.984437161	0.006812001
Zfp598	0.021610611	0.984596958	0.00674151
Tcp10c	-0.048214499	0.984651912	0.006717271
Specc1	-0.022195084	0.984651912	0.006717271
Ereg	0.019547709	0.984651912	0.006717271
Lrrc41	0.012272461	0.984651912	0.006717271
Nup107	-0.009599804	0.984651912	0.006717271
Gclc	-0.012968372	0.984651912	0.006717271
Gnb3	0.013467298	0.984662366	0.006712661
Ankrd54	0.023360321	0.984662366	0.006712661
Fcrla	0.052324169	0.9846753	0.006706956
Ccdc27	-0.046800552	0.9846753	0.006706956
Rnf168	0.035252003	0.9846753	0.006706956
Fut1	0.013978778	0.9846753	0.006706956
Mtmr14	0.026220437	0.9846753	0.006706956
Orc3	-0.027090549	0.9846753	0.006706956
Set	0.016638602	0.9846753	0.006706956
Mogat2	-0.011583156	0.9846753	0.006706956
Slfn3	0.021793558	0.9846753	0.006706956
Zfp395	0.034991498	0.9846753	0.006706956
Nron	-0.011691014	0.9846753	0.006706956

Pus1	-0.014540252	0.9846753	0.006706956
SELM	-0.025325261	0.9846753	0.006706956
Prtn3	-0.018107717	0.9846753	0.006706956
Uncx	-0.015636791	0.9846753	0.006706956
Wdr70	0.012956445	0.9846753	0.006706956
Ankrd40	-0.009596496	0.9846753	0.006706956
Ndufab1	0.024956441	0.9846753	0.006706956
Mrpl55	-0.018310855	0.9846753	0.006706956
Mlec	-0.033730379	0.9846753	0.006706956
Jak2	-0.01008359	0.9846753	0.006706956
Tmem167	0.011367378	0.9846753	0.006706956
Cfl2	0.014874949	0.9846753	0.006706956
Nhlrc2	-0.014610426	0.9846753	0.006706956
Cdh17	0.015798665	0.9846753	0.006706956
Neu3	-0.017620506	0.9846753	0.006706956
C2cd2	-0.015870328	0.984677863	0.006705826
Blk	0.01584859	0.984677863	0.006705826
Tmem108	-0.014810605	0.984677863	0.006705826
Mgat1	0.010730733	0.984677863	0.006705826
Mcrs1	0.016560015	0.984677863	0.006705826
Slc4a5	-0.014757924	0.984677863	0.006705826
Dsc1	-0.013979376	0.984677863	0.006705826
Med18	0.012191101	0.984677863	0.006705826
Tbc1d1	0.013820974	0.984677863	0.006705826
Ppp6r3	-0.011029872	0.984677863	0.006705826
Nup50	-0.022104779	0.984677863	0.006705826
Pard6g	0.015610696	0.984677863	0.006705826
Gulo	-0.02581309	0.984677863	0.006705826
Tgfbrap1	-0.013101705	0.984677863	0.006705826
Zfp326	-0.010954089	0.984677863	0.006705826
Grm6	0.029772867	0.984677863	0.006705826
Rdh5	0.01749303	0.984677863	0.006705826
Rhno1	0.028118157	0.984780475	0.00666057
C9	0.020852619	0.984796362	0.006653564
Drd4	0.014297108	0.984796362	0.006653564
Hephl1	-0.023981946	0.984796362	0.006653564
Manbal	-0.018171954	0.984796362	0.006653564
Parp1	-0.017415128	0.984796362	0.006653564
Gucd1	0.024292954	0.984796362	0.006653564
Zfp740	0.009836459	0.984796362	0.006653564
Ctdnep1	0.014602297	0.984796362	0.006653564
Pde7b	0.014428881	0.984796362	0.006653564
Mrps35	0.019359101	0.984796362	0.006653564
Zfp790	-0.015964128	0.984796362	0.006653564
Slc22a13	-0.027921926	0.984796362	0.006653564
Ly6h	0.039918682	0.984878276	0.006617442
Zfp882	0.010816697	0.984878276	0.006617442
Dnajb4	0.01452493	0.984935239	0.006592324

Rlim	-0.014547747	0.985068063	0.006533761
Icam5	-0.015134193	0.985111094	0.00651479
Loxhd1	0.010760949	0.985151167	0.006497124
Kcng3	-0.018743194	0.985296662	0.006432988
Vwa3a	0.022034818	0.985342329	0.00641286
AY512931	0.014436367	0.985667225	0.006269684
Denr	-0.007492691	0.985867816	0.006181311
Gpbp1l1	0.012331298	0.985982531	0.00613078
Phf5a	0.01629042	0.986116822	0.006071633
PTCHD2	-0.01011385	0.986189706	0.006039535
Fam102a	0.02913317	0.986383836	0.005954053
Thsd7b	0.013945642	0.986383836	0.005954053
Rfx1	0.008702464	0.986383836	0.005954053
Cacna1f	-0.029247165	0.986383836	0.005954053
Ddx52	-0.018358436	0.986383836	0.005954053
Mrrf	0.015681546	0.986383836	0.005954053
Nox1	-0.020126319	0.986383836	0.005954053
Yipf4	-0.009723225	0.986383836	0.005954053
Mtnr1a	0.014679634	0.986383836	0.005954053
Ascc3	-0.009155799	0.986383836	0.005954053
Olfr558	0.008623226	0.986383836	0.005954053
DUPD1	-0.013085287	0.986383836	0.005954053
D5Ertd615	0.016347897	0.986383836	0.005954053
Pdilt	0.012132877	0.986383836	0.005954053
Phtf1os	0.013777856	0.986383836	0.005954053
Stag2	-0.015937868	0.986383836	0.005954053
Sectm1a	-0.014902409	0.986383836	0.005954053
Tbl1xr1	-0.010041837	0.986383836	0.005954053
Mfn1	-0.012148585	0.986383836	0.005954053
Cuedc1	-0.020947231	0.986383836	0.005954053
Spag17	0.028329408	0.986383836	0.005954053
Rnf144b	-0.010611769	0.986383836	0.005954053
Seh1l	0.017389436	0.986383836	0.005954053
Cdr2	-0.01206089	0.986383836	0.005954053
Bdkrb1	-0.00921976	0.986383836	0.005954053
Rfx3	0.031908407	0.986562362	0.005875457
AW04620(	0.014985391	0.986639281	0.005841598
BC037032	0.01422511	0.986639281	0.005841598
Slc38a7	-0.045342845	0.986947776	0.005705827
Tnfrsf21	-0.025948239	0.986947776	0.005705827
Tnfaip1	0.012591426	0.986947776	0.005705827
Muc3	-0.013908055	0.986947776	0.005705827
Rgs22	-0.014472496	0.986947776	0.005705827
Atm	0.014233701	0.986947776	0.005705827
Atp10a	-0.020509483	0.986947776	0.005705827
Ddx59	0.011230408	0.986947776	0.005705827
Ripk4	0.012336333	0.986947776	0.005705827
Gas2l1	-0.013230491	0.986947776	0.005705827

Ppef1	0.037687335	0.986947776	0.005705827
Rab1a	0.012859942	0.986947776	0.005705827
Dydc2	0.01142103	0.986947776	0.005705827
Tdrp	-0.015359893	0.986947776	0.005705827
Urm1	-0.026077193	0.986947776	0.005705827
Cldnd2	0.015254626	0.986947776	0.005705827
Dpp3	0.012779327	0.986947776	0.005705827
Rhbdd3	0.008716591	0.986947776	0.005705827
C8b	0.020836934	0.986947776	0.005705827
Crip2	0.012725255	0.986947776	0.005705827
Pdpr	0.013059801	0.986947776	0.005705827
Dapk3	0.023885017	0.987090402	0.005643071
Pgm2	0.015944167	0.987090402	0.005643071
Prtg	0.015296009	0.987090402	0.005643071
Mrps2	0.01864068	0.987090402	0.005643071
Wdr53	0.008696686	0.987090402	0.005643071
Pla2g6	-0.011338737	0.987090402	0.005643071
Hnrnpdl	0.029958487	0.987090402	0.005643071
Actr2	0.014419926	0.987090402	0.005643071
Sntb2	-0.007243855	0.987090402	0.005643071
Zfp287	-0.006860138	0.987090402	0.005643071
Srl	0.014987098	0.987090402	0.005643071
Ttll6	0.016907844	0.987090402	0.005643071
Sync	-0.019530146	0.987109735	0.005634565
Snrnp25	0.040148306	0.987143122	0.005619876
Paqr8	-0.026528627	0.987143122	0.005619876
Cygb	-0.058929454	0.987143122	0.005619876
Spata31d1	-0.020445845	0.987143122	0.005619876
Mettl7a3	-0.012980066	0.987143122	0.005619876
Anp32a	0.028337277	0.987143122	0.005619876
Dpy19l3	0.019251441	0.987143122	0.005619876
Bcas3os1	0.0662733	0.987143122	0.005619876
Thoc7	0.03610312	0.987143122	0.005619876
Xpnpep2	0.021938765	0.987143122	0.005619876
Son	-0.018299104	0.987143122	0.005619876
Ube2f	-0.038679673	0.987143122	0.005619876
Arl8a	0.017842684	0.987143122	0.005619876
Fancd2os	0.012000771	0.987143122	0.005619876
Pnldc1	-0.006580756	0.987143122	0.005619876
Anapc15	0.011064191	0.987143122	0.005619876
Acat1	-0.021917724	0.987143122	0.005619876
METTL10	0.016798892	0.987143122	0.005619876
Aldh3b2	0.011670493	0.987143122	0.005619876
Zfp646	0.028640037	0.987143122	0.005619876
Ankra2	-0.007556383	0.987143122	0.005619876
Cd209b	0.009569446	0.987143122	0.005619876
Olfr221	0.022277467	0.987143122	0.005619876
Rer1	0.008943714	0.987143122	0.005619876

Psmg3	0.008550769	0.987143122	0.005619876
Dus2	-0.013005146	0.987143122	0.005619876
Gpkow	0.010256832	0.987143122	0.005619876
Ndufa7	0.014652569	0.987143122	0.005619876
Nr2c1	0.020776401	0.987143122	0.005619876
Csde1	-0.008987869	0.987143122	0.005619876
Mrpl18	0.013571239	0.987143122	0.005619876
Bpifb5	-0.014364016	0.987143122	0.005619876
Ppp2r2a	-0.012988715	0.987143122	0.005619876
Ppp6c	-0.057836465	0.987143122	0.005619876
Serpina1b	-0.012359731	0.987143122	0.005619876
Gja10	0.00937596	0.987143122	0.005619876
Dync1i2	-0.009738727	0.987143122	0.005619876
Ush2a	-0.011994511	0.987143122	0.005619876
Spen	0.079995882	0.987143122	0.005619876
Mrpl15	-0.011948208	0.987143122	0.005619876
Tprgl	0.025593794	0.987143122	0.005619876
Tmem18	0.010445239	0.987143122	0.005619876
FAM26D	-0.016110774	0.987143122	0.005619876
Tubb4b	-0.035533759	0.987295707	0.005552751
Tbc1d14	-0.017260372	0.987295707	0.005552751
Mdm4	-0.010996587	0.987295707	0.005552751
Zfp408	-0.009048085	0.987295707	0.005552751
Tmed9	0.014261487	0.987295707	0.005552751
Eprs	0.015378882	0.987295707	0.005552751
Tcof1	-0.008988184	0.987295707	0.005552751
Brk1	0.017632207	0.987295707	0.005552751
Psmd10	0.013215066	0.987295707	0.005552751
Chd1	0.011343519	0.987295707	0.005552751
Kbtbd12	-0.00809945	0.987489582	0.005467478
Angptl3	-0.017922884	0.987489582	0.005467478
Med23	0.012360294	0.987489582	0.005467478
WBSCR22	-0.009648506	0.987584873	0.005425571
Tmem132	-0.014705248	0.987587722	0.005424318
Mrpl37	0.013195403	0.987587722	0.005424318
Bend7	0.045630216	0.987587722	0.005424318
AI450353	-0.015710814	0.987587722	0.005424318
Zfp865	0.008757922	0.987587722	0.005424318
Uba2	0.032691886	0.987587722	0.005424318
MINA	-0.012070732	0.987780518	0.005339544
Hsd17b14	0.015774312	0.987780518	0.005339544
Ube2d2a	0.024389052	0.987780518	0.005339544
Fchsd1	-0.014884858	0.987780518	0.005339544
Pramel1	-0.012324289	0.987780518	0.005339544
Tfg	-0.009035234	0.987781747	0.005339003
Nucks1	0.020501259	0.987781747	0.005339003
Eif3e	-0.009601955	0.987781747	0.005339003
Tcp11l2	-0.016975238	0.987888401	0.005292114

Hes2	-0.013235146	0.987898318	0.005287754
Ntn3	-0.015036299	0.987898318	0.005287754
Zfp592	-0.008654076	0.987942376	0.005268386
Slc10a5	-0.013662721	0.987942376	0.005268386
Taf4	0.016349356	0.987942376	0.005268386
Tor1a	-0.024112503	0.987942376	0.005268386
Rptoros	0.034528072	0.988123801	0.00518864
Bnip3l	0.01409268	0.988123801	0.00518864
Gpank1	-0.035929014	0.988123801	0.00518864
Muc16	0.005336329	0.988123801	0.00518864
Mark3	0.014712535	0.988321821	0.005101616
Ctnnbip1	-0.009211737	0.988321821	0.005101616
Pycrl	-0.017907114	0.988417266	0.005059677
Spag7	-0.041797092	0.988417266	0.005059677
Tnk1	0.021541078	0.988435106	0.005051838
Mageh1	0.018705521	0.988435106	0.005051838
Dusp11	-0.01143812	0.988553651	0.004999755
P2rx2	-0.01997078	0.988574415	0.004990633
Glyatl3	0.007619162	0.988591389	0.004983177
Vwde	0.016976482	0.988597869	0.00498033
Rfk	-0.01174986	0.988597869	0.00498033
Hsdl1	0.0216047	0.988598866	0.004979892
Anxa7	0.024822069	0.98866189	0.004952206
D7Ertd443	-0.025198415	0.98866189	0.004952206
Catip	0.015060748	0.98866189	0.004952206
Cops7b	-0.007321058	0.98869431	0.004937965
Dennd3	-0.012419497	0.98869431	0.004937965
Tti2	-0.020452188	0.988694837	0.004937734
Oxct2b	0.012150753	0.988694837	0.004937734
Taf8	0.027069264	0.988694837	0.004937734
Pkia	-0.014674195	0.988777246	0.004901536
Unc45b	-0.01213021	0.988777246	0.004901536
Rab7	0.010357678	0.988857448	0.004866311
Cyld	0.011272792	0.988857448	0.004866311
Dcaf15	0.009001723	0.988857448	0.004866311
Olfr317	-0.020854341	0.98893302	0.004833122
Pcdhga8	0.02854231	0.98893302	0.004833122
Ccr6	-0.010880645	0.98893302	0.004833122
Platr6	0.021070634	0.988956905	0.004822633
Zfp617	0.015777722	0.988956905	0.004822633
Zyg11a	0.159451961	0.988956905	0.004822633
GLTSCR1	-0.044181648	0.988956905	0.004822633
Abcc9	0.048211974	0.988956905	0.004822633
Corin	-0.015110163	0.988956905	0.004822633
Rbm25	0.014987677	0.988956905	0.004822633
Crocc2	-0.014491776	0.988956905	0.004822633
Crcp	0.025621029	0.988956905	0.004822633
Srpr	0.011416794	0.988956905	0.004822633

Otop3	0.023025398	0.988956905	0.004822633
Gimap3	-0.029489945	0.988956905	0.004822633
Emc1	-0.009074011	0.988956905	0.004822633
Slc25a31	-0.016336023	0.988956905	0.004822633
Phax	-0.01448439	0.988956905	0.004822633
Uri1	0.008685341	0.988956905	0.004822633
Cnga3	0.017232458	0.988956905	0.004822633
Arntl2	-0.009920415	0.988956905	0.004822633
Tcf15	-0.013254823	0.988956905	0.004822633
Spats2	0.013556795	0.988956905	0.004822633
Oxgr1	-0.008071427	0.988956905	0.004822633
Klf17	-0.011461741	0.989148591	0.004738463
Erp44	-0.018957294	0.989148591	0.004738463
Dlx3	0.014905181	0.989455393	0.00460378
Riok1	-0.009199458	0.989661053	0.004513521
Stk4	0.02321846	0.989813515	0.00444662
Cops4	0.011626881	0.989813515	0.00444662
Cfap73	0.015058771	0.989813515	0.00444662
Dhx9	-0.006878935	0.989813515	0.00444662
Cops2	0.035145628	0.990029314	0.004351946
Brd2	-0.009902735	0.990029314	0.004351946
Kirrel3os	0.017475883	0.990029314	0.004351946
Pdcd6	0.016619757	0.990029314	0.004351946
Slc45a2	-0.018237883	0.990029314	0.004351946
Ece2	0.013364826	0.990029314	0.004351946
Tsix	0.012465794	0.990029314	0.004351946
Dok3	0.018127659	0.990029314	0.004351946
Trmt112	-0.013534562	0.990029314	0.004351946
Tbkbp1	0.0154211	0.990029314	0.004351946
Il2ra	-0.013226896	0.990029314	0.004351946
BC003331	0.01373825	0.990029314	0.004351946
Mov10l1	0.01278951	0.990029314	0.004351946
Fus	0.006844502	0.990029314	0.004351946
Wdr45b	0.011658861	0.990029314	0.004351946
Cmpk1	-0.009963017	0.990029314	0.004351946
Top1mt	0.006000351	0.990029314	0.004351946
Ywhae	0.009784444	0.990029314	0.004351946
Hint1	0.014543111	0.990029314	0.004351946
Clip4	0.015684335	0.990127935	0.004308686
Klhdc4	0.018283947	0.990184345	0.004283944
Pex7	-0.022948106	0.990278169	0.004242795
Pcca	-0.009115058	0.990278169	0.004242795
Usp54	-0.008313663	0.990278169	0.004242795
Treml1	-0.01038066	0.990278169	0.004242795
Rnf38	-0.013159547	0.990278169	0.004242795
CTAGE5	-0.008834592	0.99027971	0.004242119
Lrig2	0.013757043	0.990373672	0.004200913
Repin1	-0.014659381	0.990373672	0.004200913

ASUN	-0.011721686	0.990373672	0.004200913
Nol8	-0.017191116	0.990373672	0.004200913
Zan	0.011468785	0.990418975	0.004181048
Mrm2	0.022907786	0.990606307	0.004098911
Pard3bos1	-0.008220088	0.990606307	0.004098911
Fam227b	-0.00969471	0.990606307	0.004098911
Ercc2	-0.00748738	0.990606307	0.004098911
Psmb7	0.031358588	0.990606307	0.004098911
Rufy4	-0.011515476	0.990606307	0.004098911
Rdh16	-0.015559246	0.990606307	0.004098911
Zfp59	0.007988223	0.990606307	0.004098911
Adgrg4	-0.009915453	0.990606307	0.004098911
Iscu	0.013936457	0.990606307	0.004098911
ENTHD2	0.019278383	0.990606307	0.004098911
Cnnm4	-0.009656676	0.990606307	0.004098911
CARKD	-0.013645949	0.990606307	0.004098911
Cct4	-0.019640616	0.990683909	0.004064891
Thumpd2	0.020436191	0.990683909	0.004064891
Muc15	-0.023115912	0.990683909	0.004064891
Slc10a6	0.021282808	0.990683909	0.004064891
Ramp1	0.022953476	0.990683909	0.004064891
Nek10	0.044718922	0.990683909	0.004064891
Tmem150	0.010951795	0.990683909	0.004064891
Stab2	-0.014440429	0.990683909	0.004064891
Klri1	-0.009721231	0.990683909	0.004064891
Eps15l1	0.035917291	0.990683909	0.004064891
Fam83b	-0.020295481	0.990683909	0.004064891
Angel2	-0.016306127	0.990683909	0.004064891
Cnpy3	0.011465264	0.990683909	0.004064891
Sdr42e1	-0.007229312	0.990683909	0.004064891
Uqcrb	0.014893139	0.990683909	0.004064891
Manf	0.007731183	0.990683909	0.004064891
B3gat2	-0.008557825	0.990683909	0.004064891
Rab21	-0.01126675	0.990683909	0.004064891
Ptdss2	0.007144977	0.990847832	0.003993037
Ccdc93	0.011150369	0.990847832	0.003993037
Slc24a1	-0.010205244	0.990847832	0.003993037
Sec14l2	0.010230995	0.990909183	0.003966147
Pm20d2	0.020828904	0.990910934	0.003965379
Suclg1	-0.01059342	0.990910934	0.003965379
Spink8	0.011451078	0.990910934	0.003965379
Mir22hg	0.010658641	0.990910934	0.003965379
Prpf38a	0.015564988	0.990910934	0.003965379
Stat5b	0.010444312	0.990910934	0.003965379
Polr2a	-0.02060944	0.990910934	0.003965379
Caml	-0.016455279	0.990910934	0.003965379
Nkx6-3	0.0088044	0.990910934	0.003965379
Cdpf1	0.009766227	0.990910934	0.003965379

Gcgr	-0.009242645	0.990910934	0.003965379
Slc38a9	0.012070137	0.990910934	0.003965379
Arfgap2	0.014828485	0.990910934	0.003965379
Scfd2	-0.018245625	0.990910934	0.003965379
Pcdhb13	0.02297623	0.990910934	0.003965379
Tfap2e	-0.017894067	0.990910934	0.003965379
Prss32	0.014084134	0.990910934	0.003965379
Kdm3a	0.009613404	0.990910934	0.003965379
Npy2r	-0.007165089	0.990910934	0.003965379
Slc25a41	0.010429084	0.990910934	0.003965379
Igsf1	-0.01405207	0.990910934	0.003965379
Zbtb21	-0.010448156	0.990910934	0.003965379
Nap1l4	0.016536194	0.9909803	0.003934979
Ilkap	0.019911056	0.9909803	0.003934979
Ccdc38	0.039473746	0.991139719	0.003865119
Nkap	-0.014594228	0.991139719	0.003865119
Noa1	0.018931893	0.99126752	0.003809124
Pdk3	0.011110082	0.99126752	0.003809124
Cebpz	-0.012886409	0.99126752	0.003809124
Tcf21	-0.007748171	0.991528536	0.003694782
Upf3a	0.022947118	0.991528536	0.003694782
Phyh	-0.012274965	0.991560281	0.003680878
Rhpn1	0.01385572	0.991560281	0.003680878
Xab2	0.012105912	0.991560281	0.003680878
Stk32b	0.032707703	0.991560281	0.003680878
Kdm5c	0.010030124	0.991560281	0.003680878
Ddx17	-0.011086289	0.991560281	0.003680878
Simc1	0.014471086	0.991637441	0.003647084
Snx30	0.027038935	0.991690468	0.003623861
Dtnb	0.021876626	0.992063481	0.003460537
Tdrd5	0.009372739	0.992063481	0.003460537
Golt1b	0.02804506	0.992069073	0.003458089
Med21	0.011493038	0.992069073	0.003458089
Eif3h	0.021284222	0.992069073	0.003458089
Dnase2b	0.011293471	0.992069073	0.003458089
Hacd1	0.019558594	0.992069073	0.003458089
Bahcc1	0.015881954	0.992069073	0.003458089
Tbcd	-0.012151494	0.992069073	0.003458089
Muc5b	-0.018786919	0.992069073	0.003458089
Rps27rt	-0.009104	0.992069073	0.003458089
Raly	0.032064009	0.992069073	0.003458089
Eno1	0.008996381	0.992069073	0.003458089
Gramd1a	-0.009167991	0.992069073	0.003458089
METTL13	-0.013312534	0.992069073	0.003458089
Ppp1cc	-0.008419393	0.992069073	0.003458089
Tmem117	0.009591126	0.992069073	0.003458089
Trim15	-0.010655602	0.992069073	0.003458089
Cxadr	-0.022196349	0.992069073	0.003458089

Gnat2	-0.01506095	0.992069073	0.003458089
Hspa14	-0.008456591	0.992069073	0.003458089
Zswim5	0.015144235	0.992069073	0.003458089
Slc30a5	-0.01286697	0.992069073	0.003458089
Tbx4	-0.010863526	0.992069073	0.003458089
Ddx3y	0.013565711	0.992069073	0.003458089
Eif2s3y	-0.008115568	0.992069073	0.003458089
Kdm5d	-0.031691604	0.992069073	0.003458089
Uty	0.011068859	0.992069073	0.003458089
SCARNA13	0.017617885	0.992069073	0.003458089
Uba1y	-0.010438284	0.992069073	0.003458089
S100a5	0.019455943	0.992069073	0.003458089
Insl3	0.014582621	0.992150577	0.003422411
HIST1H2AI	-0.012942419	0.992150874	0.003422281
Rpl34-ps1	-0.033040608	0.992192612	0.003404011
Scgb1c1	-0.009247717	0.992192612	0.003404011
Prm2	-0.029819054	0.992205474	0.003398381
Fabp6	0.018314626	0.992223276	0.003390589
Rnu2-10	-0.045444077	0.992253184	0.003377499
Slurp2	0.016716826	0.992253184	0.003377499
RNU3B3	-0.020461181	0.992253184	0.003377499
Snord17	0.009098229	0.992253184	0.003377499
RNU3B2	0.014884465	0.992253184	0.003377499
Ccl1	0.009614553	0.992253184	0.003377499
Gpha2	-0.008862212	0.992253184	0.003377499
Olfr96	-0.009896452	0.992253184	0.003377499
Ptprtos	-0.014379398	0.992253184	0.003377499
Cib3	0.009607498	0.992253184	0.003377499
Tomm20l	-0.007643048	0.992253184	0.003377499
Mup12	0.008342724	0.992253184	0.003377499
D14Ertd67	-0.015618809	0.992253184	0.003377499
WBSCR28	0.01161653	0.992253184	0.003377499
HIST1H2BI	0.005751838	0.992253184	0.003377499
Cnfn	-0.015420157	0.992634182	0.003210773
Epo	-0.013674249	0.992634182	0.003210773
Olfr49	-0.022837253	0.992634182	0.003210773
Hoxb7	-0.008208941	0.992634182	0.003210773
Azgp1	0.010329977	0.992634182	0.003210773
Vaultrc5	0.015664338	0.992634182	0.003210773
Klk1b16	-0.016547598	0.992634182	0.003210773
Reg3a	-0.013878639	0.992634182	0.003210773
Prap1	-0.013828494	0.992634182	0.003210773
Olfr10	-0.008265932	0.992634182	0.003210773
Foxi3	0.012589198	0.992634182	0.003210773
Hopxos	0.009820588	0.992634182	0.003210773
Stfa1	-0.008458017	0.992634182	0.003210773
Vgll1	0.008398755	0.992634182	0.003210773
Otor	-0.025984328	0.992634182	0.003210773

Halr1	0.027748638	0.992634182	0.003210773
Spink4	0.009599951	0.992634182	0.003210773
Tex43	-0.007642644	0.992634182	0.003210773
Lce1i	-0.012095347	0.992634182	0.003210773
Ang2	-0.015807654	0.992634182	0.003210773
Prm3	-0.008167836	0.992634182	0.003210773
Bcl2l10	-0.009967877	0.992634182	0.003210773
Prr23a2	0.007609357	0.992634182	0.003210773
Hesx1	-0.011800254	0.992634182	0.003210773
Speer4e	-0.008803959	0.992634182	0.003210773
Lyrm7os	-0.028400105	0.992634182	0.003210773
Olfr1257	0.008439922	0.992634182	0.003210773
Pax4	-0.008827519	0.992634182	0.003210773
Klk1b22	0.013956756	0.992647927	0.00320476
Olfr195	0.009328837	0.992647927	0.00320476
Olfr560	0.011062281	0.992708691	0.003178176
Olfr1336	0.008629724	0.992708691	0.003178176
Cldn34b4	-0.013400507	0.992708691	0.003178176
Olfr677	0.007559189	0.992708691	0.003178176
Olfr273	0.016070207	0.992730535	0.00316862
Olfr533	0.0096393	0.992730535	0.00316862
Olfr651	0.031906304	0.992756945	0.003157066
Fkbp6	0.035812246	0.992756945	0.003157066
Olfr859	-0.022355557	0.992756945	0.003157066
Vmn1r29	0.012671296	0.992942026	0.003076107
Eras	0.01200167	0.992942026	0.003076107
Olfr1371	-0.024478249	0.992942026	0.003076107
Olfr992	-0.011627531	0.992942026	0.003076107
Olfr1383	-0.012147628	0.992942026	0.003076107
Bcas3os2	-0.009266046	0.992942026	0.003076107
Grip1os1	0.010302486	0.992942026	0.003076107
Olfr455	0.011535767	0.992942026	0.003076107
Cst13	-0.010839369	0.992942026	0.003076107
Scgb2b2	0.006106015	0.992942026	0.003076107
Olfr668	0.013421508	0.992942026	0.003076107
Olfr1389	0.009066311	0.992942026	0.003076107
Tmem207	-0.014584882	0.992942026	0.003076107
Prkag2os1	-0.010343305	0.992942026	0.003076107
Cxcl17	-0.0121537	0.992942026	0.003076107
Prss27	-0.028712863	0.992942026	0.003076107
Arl14epl	-0.009907738	0.992942026	0.003076107
Olfr585	-0.009418742	0.992942026	0.003076107
Nkx3-2	0.007013751	0.992942026	0.003076107
BC065403	0.009183287	0.992942026	0.003076107
Cst12	-0.0345023	0.992965294	0.003065931
Lce3a	0.010114487	0.992965294	0.003065931
Wfdc5	-0.01308988	0.992965294	0.003065931
Tnfsf4	0.007388278	0.992965294	0.003065931

Gpbar1	0.006980016	0.992965294	0.003065931
Lce3c	0.010804267	0.992965294	0.003065931
Csl	-0.018611735	0.992966171	0.003065547
U90926	0.01481729	0.992966171	0.003065547
Sprr2i	-0.013886232	0.992966171	0.003065547
Ifna9	-0.017026207	0.992966171	0.003065547
Slfn5os	0.015612648	0.992966171	0.003065547
Adig	0.006259379	0.992966171	0.003065547
Svs5	-0.007809642	0.992966171	0.003065547
Iqcf1	-0.017098723	0.992966171	0.003065547
Rnf186	-0.013452085	0.992966171	0.003065547
Ssxb8	0.01393449	0.992998908	0.003051229
Prss28	-0.018985268	0.992998908	0.003051229
Clec4a4	-0.009676652	0.992998908	0.003051229
Pldi	-0.013664775	0.992998908	0.003051229
Olfr538	0.016522909	0.992998908	0.003051229
Oosp2	-0.016680453	0.992998908	0.003051229
Ifnab	0.008368289	0.992998908	0.003051229
Amelx	0.012582301	0.992998908	0.003051229
Lcn3	-0.011238326	0.992998908	0.003051229
Olfr153	-0.008570605	0.992998908	0.003051229
Tlx3	0.013892488	0.992998908	0.003051229
Rbakdn	0.015581297	0.992998908	0.003051229
Olfr1358	0.010772081	0.992998908	0.003051229
Wnt3a	-0.009529742	0.992998908	0.003051229
Fam219ac	-0.014330072	0.992998908	0.003051229
Tmco2	-0.006615743	0.992998908	0.003051229
Tex12	0.023395798	0.992998908	0.003051229
Klk4	-0.009068961	0.992998908	0.003051229
Mup20	0.009213792	0.992998908	0.003051229
Serpina3m	0.010945712	0.992998908	0.003051229
Crp	0.021129147	0.99299983	0.003050826
Klk5	-0.010976247	0.993050997	0.003028448
Ms4a13	-0.015667764	0.993050997	0.003028448
Spint4	-0.009451304	0.993050997	0.003028448
Arrdc5	0.007522656	0.993050997	0.003028448
Rhox10	-0.007755319	0.993050997	0.003028448
Slc22a19	0.012141738	0.993050997	0.003028448
Ugt2a2	-0.014241071	0.993124335	0.002996376
Sbpl	0.013469316	0.993124335	0.002996376
Krtap5-3	0.028395177	0.993124335	0.002996376
Pth	-0.013930278	0.993124335	0.002996376
Rhox4b	0.015703141	0.993124335	0.002996376
Cyp11b2	0.006724684	0.993124335	0.002996376
Tmem54	0.011961369	0.993124335	0.002996376
Sprr1b	0.011523015	0.993124335	0.002996376
Fcrl6	-0.010623603	0.993124335	0.002996376
Vmn1r31	-0.008593114	0.993124335	0.002996376

Olfr917	-0.007986994	0.993124335	0.002996376
Calm4	0.008623262	0.993124335	0.002996376
Olfr311	-0.009623663	0.993124335	0.002996376
Cyp4f37	0.011081236	0.993124335	0.002996376
Olfr462	-0.012377969	0.993124335	0.002996376
Pitpnm2os	0.013089554	0.993124335	0.002996376
Olfr1377	-0.008139735	0.993124335	0.002996376
Olfr24	-0.009157028	0.993124335	0.002996376
Oas1f	0.013609861	0.993124335	0.002996376
Mup17	0.008379137	0.993124335	0.002996376
Olfr561	-0.01123692	0.993124335	0.002996376
Olfr566	-0.013258179	0.993124335	0.002996376
Olfr1512	0.007467619	0.993124335	0.002996376
Olfr552	-0.006563696	0.993124335	0.002996376
Olfr993	0.00924508	0.993124335	0.002996376
Trim50	0.016021812	0.993124335	0.002996376
Olfr1395	0.010491565	0.993124335	0.002996376
Olfr224	-0.010459839	0.993173061	0.002975069
Try10	-0.020243648	0.993173061	0.002975069
Cela2a	0.032994012	0.993173061	0.002975069
Olfr638	0.026737622	0.993173061	0.002975069
Olfr414	0.015740334	0.993173061	0.002975069
Tcaf3	-0.016675397	0.993173061	0.002975069
Tas2r135	0.009823921	0.993173061	0.002975069
Olfr1340	0.017200913	0.993173061	0.002975069
Olfr1195	0.01989166	0.993173061	0.002975069
Obp2a	0.010760048	0.993173061	0.002975069
Hoxb13	-0.007034589	0.993173061	0.002975069
Fhad1os1	-0.013234448	0.993173061	0.002975069
Olfr1339	-0.006797657	0.993173061	0.002975069
Pate4	-0.007970251	0.993173061	0.002975069
Tas2r125	-0.014381406	0.993173061	0.002975069
Vmn1r210	0.018007266	0.993221337	0.002953959
Olfr235	0.008104506	0.993221337	0.002953959
Krtap4-16	-0.012865589	0.993221337	0.002953959
Olfr564	-0.01974835	0.993221337	0.002953959
BC048502	-0.006782509	0.993221337	0.002953959
Olfr1305	-0.020106068	0.993246614	0.002942907
Il20	-0.01987886	0.993254534	0.002939444
Ly6g6c	-0.013545981	0.993254534	0.002939444
BC024386	-0.008236527	0.993254534	0.002939444
Tmem225	-0.014258272	0.993254534	0.002939444
Krt79	-0.015098395	0.993254534	0.002939444
TEX13	-0.011166624	0.993254534	0.002939444
Prl2c5	0.007687117	0.993254534	0.002939444
Zfy1	0.023607623	0.993254534	0.002939444
Hnf1aos1	-0.017743395	0.993254534	0.002939444
Calhm1	0.007269968	0.993254534	0.002939444

Gzmg	0.014898719	0.993254534	0.002939444
Cyp3a11	-0.011144891	0.993254534	0.002939444
Ceacam2(	-0.015159777	0.993254534	0.002939444
Krtap4-1	0.008704503	0.993254534	0.002939444
Tm4sf20	-0.00820237	0.993254534	0.002939444
Taar5	-0.021005355	0.993254534	0.002939444
Cabp2	0.010000897	0.993254534	0.002939444
Cyp2d40	0.007034328	0.993254534	0.002939444
Olfr1128	-0.009453195	0.993254534	0.002939444
Dhrs7c	0.010929341	0.993254534	0.002939444
Kpna7	0.007668484	0.993254534	0.002939444
Olfr1364	0.01814189	0.993254534	0.002939444
Rdh8	-0.024500839	0.993446722	0.002855419
Actc1	0.029571697	0.993446722	0.002855419
Ambn	-0.009633882	0.993446722	0.002855419
Krt36	-0.009963791	0.993482801	0.002839647
Fbxw25	0.014509772	0.993562824	0.002804667
Krt82	-0.01099469	0.993562824	0.002804667
Ccr8	0.010960208	0.993562824	0.002804667
Rfpl4b	0.007969892	0.993562824	0.002804667
Mettl21e	-0.013773621	0.993562824	0.002804667
Ankrd60	0.006559657	0.993562824	0.002804667
Grxcr1	-0.013347286	0.993562824	0.002804667
Ugt3a2	-0.021868091	0.993562824	0.002804667
Olfr510	-0.008585414	0.993562824	0.002804667
Prss52	0.008085404	0.993562824	0.002804667
Mmp20	0.010580887	0.993562824	0.002804667
Cyp2ab1	0.008114108	0.993562824	0.002804667
Krt88	-0.014639088	0.993586713	0.002794225
Akr1c21	0.019278608	0.993596724	0.002789849
Olfr812	0.012344651	0.993596724	0.002789849
Olfr1428	-0.029035812	0.993596724	0.002789849
Runx2os3	-0.025046629	0.993596724	0.002789849
Capza3	0.008488835	0.993734502	0.002729632
Gzmn	-0.035790189	0.993754981	0.002720681
Olfr57	-0.016645455	0.993791111	0.002704892
CPO	-0.016178416	0.993791111	0.002704892
Aadac	0.010641082	0.993791111	0.002704892
ACPT	-0.010663979	0.993791111	0.002704892
Krt83	0.018700002	0.993791111	0.002704892
Acsm2	0.006223566	0.993791111	0.002704892
Bpifb2	0.007965276	0.993791111	0.002704892
Bpifb6	-0.010060638	0.993791111	0.002704892
G6BOS	-0.020519	0.993791111	0.002704892
Myl10	-0.01055845	0.993791111	0.002704892
Prss29	0.009546107	0.993791111	0.002704892
Hoxb6	0.011094844	0.993791111	0.002704892
Vmn1r58	-0.01203601	0.993791111	0.002704892

ALS2CR11	-0.010215112	0.993791111	0.002704892
Fam170a	-0.010922496	0.993791111	0.002704892
Becn2	0.010022914	0.993798483	0.002701671
Rnf224	0.019374104	0.993798483	0.002701671
Crisp2	0.012393934	0.993798483	0.002701671
Platr11	0.013749378	0.993798483	0.002701671
Nutm2	0.012818148	0.993802546	0.002699895
Toporsl	-0.008504447	0.993802546	0.002699895
Klrc3	-0.023409015	0.993869457	0.002670656
Dcpp1	0.021795352	0.993869457	0.002670656
Pinc	0.012215991	0.993869457	0.002670656
Mnx1	-0.010769179	0.993869457	0.002670656
Krt34	-0.015083787	0.993930177	0.002644124
Serpinb3a	-0.131747814	0.993964393	0.002629173
Krt32	0.036012479	0.993964393	0.002629173
D7ERTD14	-0.010482027	0.993964393	0.002629173
Krt33a	0.017601754	0.993964393	0.002629173
Fbxw19	-0.009644398	0.993964393	0.002629173
Ugt1a2	0.015979116	0.993964393	0.002629173
Foxr1	0.006770137	0.993964393	0.002629173
Krt13	-0.017031259	0.993964393	0.002629173
Arhgap15c	-0.020981597	0.993964393	0.002629173
BC048671	0.014954824	0.993964393	0.002629173
Nlrp4g	-0.01393751	0.993964393	0.002629173
Smok2a	0.010991067	0.993964393	0.002629173
A1bg	0.011455969	0.993964393	0.002629173
GIF	0.008284335	0.993964393	0.002629173
Pramel3	0.011392103	0.993964393	0.002629173
Hoxb2	-0.005573894	0.993964393	0.002629173
Ccdc183	-0.00692543	0.993964393	0.002629173
Mycs	0.014821182	0.993964393	0.002629173
Hoxa5	0.009087668	0.993964393	0.002629173
Olfr157	0.006931346	0.993964393	0.002629173
Tcp11x2	0.005066576	0.993964393	0.002629173
Trpc5os	-0.008116209	0.993964393	0.002629173
Zscan5b	-0.01367311	0.993964393	0.002629173
Adad1	0.007451509	0.993964393	0.002629173
Kcnj1	0.011081482	0.993973073	0.002625381
Ccnb3	0.009602467	0.994013868	0.002607557
Skint4	-0.01807221	0.994013868	0.002607557
Fgg	-0.015374241	0.994047593	0.002592822
Mageb18	0.007759133	0.994047593	0.002592822
Tdg-ps	0.009182865	0.994047593	0.002592822
Mrgprg	0.013923745	0.994047593	0.002592822
Tssk5	0.018506767	0.99407608	0.002580376
Btnl1	-0.008840175	0.99407608	0.002580376
Nr2e3	-0.008980475	0.99420619	0.002523537
Hoxc10	0.023234409	0.994376965	0.002448945

Prss44	-0.004596111	0.994376965	0.002448945
Hoxb1	0.008932681	0.994411566	0.002433833
Fbxo39	0.009898394	0.994503113	0.002393853
Mrgprx2	0.012408577	0.994597687	0.002352555
Pglyrp4	0.014810752	0.994597687	0.002352555
Fam71f1	-0.013386054	0.994597687	0.002352555
Kif2b	-0.010404506	0.994597687	0.002352555
Chrng	-0.009672245	0.994597687	0.002352555
Krt76	-0.015765774	0.994597687	0.002352555
Ctcfl	0.02203294	0.994597687	0.002352555
Duxf3	0.01462872	0.994597687	0.002352555
Vmn2r94	0.011343226	0.994597687	0.002352555
Tex24	0.010775168	0.994597687	0.002352555
Muc20	0.010087025	0.994597687	0.002352555
Myf5	0.010441095	0.994597687	0.002352555
Ugt2b1	0.010722246	0.994597687	0.002352555
Hoxb8	-0.007799679	0.994597687	0.002352555
Cldn18	0.028118031	0.994597687	0.002352555
Gabra6	0.008904261	0.994597687	0.002352555
Zfy2	-0.011262264	0.994597687	0.002352555
Apol10a	0.012336443	0.994597687	0.002352555
Slco6d1	-0.033295726	0.994597687	0.002352555
Nlrp4c	0.020734494	0.994710096	0.002303474
Rag2	-0.013690024	0.994710096	0.002303474
Rbbp8nl	0.010382632	0.994710096	0.002303474
Actl11	-0.009407607	0.994710096	0.002303474
Cd200r3	-0.01688684	0.994710096	0.002303474
Slc10a2	-0.00813968	0.994710096	0.002303474
Slco1a1	-0.007229618	0.994710096	0.002303474
Skint5	0.015986302	0.994812718	0.002258671
Muc5ac	-0.010526811	0.994943436	0.002201609
Pzp	-0.0201035	0.995005814	0.002174381
Atp1b4	0.007396627	0.995005814	0.002174381
Olfr613	0.011551862	0.995005814	0.002174381
Evx2	0.014238121	0.995101175	0.002132761
Mug1	-0.007761387	0.995247624	0.002068851
Ces2f	-0.025757166	0.995265925	0.002060865
Fsip2	0.009111043	0.995290397	0.002050186
Hoxc6	0.01118463	0.995290397	0.002050186
AF357355	-0.020862892	0.995290397	0.002050186
AF357359	-0.015818976	0.995290397	0.002050186
AF357425	-0.005704901	0.995290397	0.002050186
AF357426	0.015828434	0.995317114	0.002038528
AF366264	0.028923563	0.995317114	0.002038528
ATP6	-0.021602597	0.995317114	0.002038528
ATP8	0.02409851	0.995317114	0.002038528
AU040096	-0.0590914	0.995317114	0.002038528
AY761184	-0.016734822	0.995317114	0.002038528

BB287469	-0.007809822	0.995317114	0.002038528
BC025446	0.013881969	0.995317114	0.002038528
BC049702	0.007572691	0.995317114	0.002038528
BC049730	0.007245534	0.995317114	0.002038528
BC061212	0.008165345	0.995317114	0.002038528
BC080695	-0.006999313	0.995317114	0.002038528
BC100530	0.010504189	0.995317114	0.002038528
BC117090	-0.015715669	0.995317114	0.002038528
BTG1-PS1	-0.018827521	0.995317114	0.002038528
BTG1-PS2	-0.00811771	0.995317114	0.002038528
C87414	0.009208363	0.995317114	0.002038528
C87977	0.007740045	0.995317114	0.002038528
COX1	-0.008645052	0.995317114	0.002038528
COX2	0.006563318	0.995430741	0.001988952
COX3	0.009747106	0.995430741	0.001988952
CPHX2	0.008296207	0.995430741	0.001988952
CPHX3	0.010182061	0.995430741	0.001988952
CYTB	0.010508349	0.995430741	0.001988952
D5ERTD57	0.015565027	0.995430741	0.001988952
Pbsn	-0.010464312	0.995430741	0.001988952
Fgf6	-0.012805077	0.995430741	0.001988952
Wap	0.01837649	0.995699454	0.001871731
Vmn2r88	-0.00508691	0.995737639	0.001855076
Tcl1b5	-0.011022031	0.995750792	0.001849339
Hoxa10	0.006723564	0.995750792	0.001849339
Hoxc11	-0.010590618	0.995815564	0.00182109
Hoxc8	0.021824292	0.99582099	0.001818724
Dsg4	0.010922077	0.99582099	0.001818724
Hoxd13	0.017945882	0.99582099	0.001818724
Hoxd12	0.004262004	0.99582099	0.001818724
Prr27	-0.011148826	0.995861912	0.001800878
Cyp2c29	-0.009288138	0.995861912	0.001800878
Psg18	0.008222159	0.995861912	0.001800878
Gpx5	0.013094233	0.995861912	0.001800878
Pcp2	-0.010376495	0.995861912	0.001800878
Tmed11	0.015017225	0.995861912	0.001800878
G6pc2	-0.018267933	0.995861912	0.001800878
Casp14	0.016895159	0.995861912	0.001800878
Evx1	-0.007588749	0.995861912	0.001800878
Prl4a1	-0.007958619	0.995861912	0.001800878
Rhox6	-0.007038581	0.996070662	0.001709851
Etv2	0.019319034	0.996101324	0.001696483
Prl7a1	-0.01059067	0.996101324	0.001696483
Prl8a9	0.009418199	0.996182465	0.001661107
Itih1	-0.010741981	0.996222218	0.001643777
Defb2	-0.022285794	0.996236896	0.001637378
Cabs1	0.030560088	0.996236896	0.001637378
Ceacam5	0.031365911	0.996236896	0.001637378

Tppp2	-0.029215494	0.996236896	0.001637378
Odam	0.025710357	0.996236896	0.001637378
Spaca7	0.061137187	0.996236896	0.001637378
Asz1	0.008346995	0.996236896	0.001637378
Phox2b	0.014970607	0.996236896	0.001637378
Sval2	0.017636648	0.996236896	0.001637378
Gip	-0.025739818	0.996236896	0.001637378
Nipsnap3a	-0.013558255	0.996236896	0.001637378
Gzmf	0.008529049	0.996236896	0.001637378
Gata5	0.011349665	0.996236896	0.001637378
Abo	0.011445394	0.996236896	0.001637378
Nlrp14	-0.008313378	0.996236896	0.001637378
Svs4	-0.019851266	0.996236896	0.001637378
Svs6	0.014118767	0.996236896	0.001637378
Ccdc70	-0.01148743	0.996236896	0.001637378
Prl5a1	-0.005495021	0.996236896	0.001637378
Pyy	-0.008670168	0.996236896	0.001637378
Ppy	-0.006599213	0.996236896	0.001637378
Eppin	0.015720199	0.996236896	0.001637378
Prl3c1	0.021916947	0.996236896	0.001637378
Wfdc15b	0.009618401	0.996236896	0.001637378
Prl8a2	0.007688284	0.996236896	0.001637378
Mmp7	-0.007667572	0.996236896	0.001637378
Il3	-0.009526038	0.996236896	0.001637378
Prl8a1	0.014214888	0.996236896	0.001637378
Zc2hc1b	-0.006707702	0.996236896	0.001637378
Ros1	0.00855014	0.996236896	0.001637378
Olfr299	0.009288246	0.996236896	0.001637378
Glipr1l2	-0.010295858	0.996236896	0.001637378
Smim23	0.008862202	0.996236896	0.001637378
Akr1c6	-0.00750238	0.996236896	0.001637378
Slc17a1	-0.005200481	0.996236896	0.001637378
Slc17a4	0.014642819	0.996236896	0.001637378
Prl8a6	-0.012527298	0.996236896	0.001637378
Prl8a8	-0.00740546	0.996236896	0.001637378
Prl7b1	0.007395964	0.996236896	0.001637378
Prl7d1	-0.008470784	0.996236896	0.001637378
Ctsq	-0.006793896	0.996236896	0.001637378
Cts7	0.005989325	0.996236896	0.001637378
Cts6	0.007281105	0.996236896	0.001637378
Il9	-0.005441469	0.996236896	0.001637378
S100z	-0.011064859	0.996236896	0.001637378
Sftpd	-0.00714443	0.996236896	0.001637378
Msmb	-0.013671864	0.996236896	0.001637378
Erich6b	0.013000229	0.996236896	0.001637378
Trim52	0.0063886	0.996236896	0.001637378
Ttc23l	0.033549759	0.996236896	0.001637378
Hoxc5	0.009597545	0.996236896	0.001637378

Ly6f	0.011242702	0.996236896	0.001637378
Miox	0.010031505	0.996236896	0.001637378
Adgrg7	0.008396875	0.996236896	0.001637378
Prl2a1	0.008507265	0.996236896	0.001637378
Stfa2	0.005472029	0.996236896	0.001637378
Krtap15	0.011924508	0.996236896	0.001637378
Fam3b	0.007960326	0.996236896	0.001637378
Krt6b	0.009036437	0.996236896	0.001637378
Fabp2	-0.011895669	0.996236896	0.001637378
Reg2	-0.004453572	0.996236896	0.001637378
Ssxb2	0.004163257	0.996236896	0.001637378
Ceacam14	0.012004353	0.996236896	0.001637378
Lcn9	0.004537865	0.996236896	0.001637378
Guca2a	-0.005340496	0.996236896	0.001637378
Cypt1	-0.01471239	0.996236896	0.001637378
Sva	-0.00761888	0.996236896	0.001637378
Gcm1	-0.015336899	0.996298255	0.00161063
Kcnk16	0.009904904	0.996298255	0.00161063
Cela3b	0.012349178	0.996298255	0.00161063
Esx1	-0.012561369	0.996298255	0.00161063
Rhag	0.007598627	0.996298255	0.00161063
Tff2	0.004419631	0.996298255	0.00161063
Tff3	0.00845549	0.996298255	0.00161063
Tff1	-0.007593927	0.996369856	0.00157942
Prss21	0.006945455	0.996369856	0.00157942
Clpsl2	-0.006299543	0.996496501	0.001524222
Lyzl1	-0.00982988	0.996496501	0.001524222
H2-M10.1	0.020477651	0.996521133	0.001513487
Spink1	-0.018129681	0.996620699	0.001470097
Ftmt	0.0195561	0.996620699	0.001470097
Dynap	0.013351664	0.996620699	0.001470097
Keg1	0.0089356	0.996626371	0.001467625
Ms4a10	0.013013632	0.996639362	0.001461965
Trpd52l3	-0.021103434	0.996639362	0.001461965
Cyp2c39	0.006677869	0.996639362	0.001461965
Cyp2c40	-0.004705359	0.996639362	0.001461965
Dusp21	0.015652044	0.996828843	0.001379404
Samt4	0.011174977	0.996890293	0.001352633
Habp2	0.008401367	0.997255906	0.001193383
Tlx1	0.017470259	0.997369262	0.001144021
Hsd17b6	0.011528336	0.997369262	0.001144021
Crisp3	-0.007872168	0.997369262	0.001144021
Satl1	0.009360006	0.997378266	0.0011401
Crisp4	0.00883999	0.997503874	0.001085409
Ccdc7a	-0.007169143	0.997503874	0.001085409
Pramel6	0.007734054	0.997503874	0.001085409
Pramel7	-0.008738589	0.997503874	0.001085409
Il17a	0.007892486	0.997503874	0.001085409

Crygf	-0.014241211	0.997535571	0.001071609
Crygc	0.0108598	0.997590756	0.001047584
Lyg1	0.011552989	0.997590756	0.001047584
Ccl20	-0.007480726	0.997590756	0.001047584
Tnp1	-0.007725189	0.997590756	0.001047584
Alppl2	-0.011547197	0.997590756	0.001047584
Chrnd	-0.021374433	0.997590756	0.001047584
Il24	0.007491606	0.997590756	0.001047584
Mptx1	-0.008256292	0.997590756	0.001047584
Apcs	-0.010188721	0.997590756	0.001047584
Lcn4	-0.005586074	0.997590756	0.001047584
Lcn5	-0.017235221	0.997590756	0.001047584
Tmem210	0.01004747	0.997590756	0.001047584
Prg3	-0.018698452	0.997590756	0.001047584
Prg2	-0.007360334	0.997590756	0.001047584
Smtnl1	0.006320515	0.997590756	0.001047584
Terb2	0.007507114	0.997590756	0.001047584
Scp2d1	0.016420147	0.997590756	0.001047584
Cst8	-0.012684332	0.997590756	0.001047584
Cst9	0.010462084	0.997590756	0.001047584
Sun5	0.018260569	0.997622512	0.001033759
Bpifa3	0.007691687	0.997622512	0.001033759
Bpifa5	0.006318705	0.997759458	0.000974147
Fabp9	-0.01301884	0.997796912	0.000957845
Cypt12	0.068192337	0.997815879	0.000949589
Il2	-0.006386705	0.997815879	0.000949589
Slc25a54	-0.016677818	0.997815879	0.000949589
Lce1m	0.009638813	0.997831083	0.000942972
Lce1b	0.00750655	0.997831083	0.000942972
Lelp1	0.008606613	0.997831083	0.000942972
Cyp7a1	-0.011567823	0.997831083	0.000942972
Slc46a2	0.016255089	0.99783496	0.000941284
Izumo3	-0.011631155	0.99783496	0.000941284
Cyp4a31	-0.005641806	0.99783496	0.000941284
Cyp4a14	-0.008399229	0.99783496	0.000941284
Galntl5	0.006763708	0.99783496	0.000941284
Uts2	0.006638372	0.99783496	0.000941284
Olfr109	0.012724128	0.997945479	0.000893185
Sult1b1	-0.010017998	0.998075377	0.000836658
Sult1e1	0.014520536	0.998075377	0.000836658
Sult1d1	-0.013559715	0.998075377	0.000836658
Smr2	0.030978658	0.998075377	0.000836658
Amtn	0.014383604	0.998075377	0.000836658
Enam	-0.007301435	0.998075377	0.000836658
Cxcl15	0.005854665	0.998179627	0.000791299
Il31	0.019944986	0.998229519	0.000769592
Pla2g1b	0.007308468	0.998229519	0.000769592
Cyp3a25	-0.012258679	0.998231007	0.000768944

Pdx1	0.013020227	0.998370035	0.000708462
Cdx2	-0.008530972	0.998370035	0.000708462
Gngt1	-0.008151404	0.998370035	0.000708462
Hyal5	-0.004070437	0.998370035	0.000708462
Hyal4	0.008416034	0.998370035	0.000708462
Spam1	0.01579181	0.998617976	0.000600621
Tmem213	-0.007926629	0.99862079	0.000599397
Stra8	-0.005903988	0.99862079	0.000599397
Sval1	0.010324753	0.998709203	0.000560948
Moxd2	-0.012025957	0.998709203	0.000560948
Gkn2	-0.008156189	0.998709203	0.000560948
Mug2	-0.01598944	0.998849484	0.00049995
Tmem52b	0.007676723	0.998849484	0.00049995
Tas2r103	-0.01948162	0.998849484	0.00049995
Clec2j	-0.026677889	0.998849484	0.00049995
Ceacam1[illegible]	0.007077844	0.998849484	0.00049995
Ceacam1[illegible]	0.010797303	0.998849484	0.00049995
Psg28	-0.006438063	0.998849484	0.00049995
Sult2a8	-0.006863867	0.998849484	0.00049995
Lypd5	-0.010461405	0.999038097	0.00041795
Slc5a2	-0.020095812	0.999089944	0.000395412
Fam24a	0.015620857	0.999150819	0.000368951
Umod	0.014480467	0.99915616	0.00036663
Gata1	-0.008805732	0.99915616	0.00036663
Ctag2	0.01538021	0.99915616	0.00036663
Kir3dl1	-0.005978226	0.99915616	0.00036663
Atp4b	-0.016746735	0.999256284	0.000323112
Defb8	0.007225366	0.999256284	0.000323112
Dkk4	-0.007385846	0.999256284	0.000323112
Triml1	0.012331482	0.999256284	0.000323112
Mt4	-0.022900706	0.999256284	0.000323112
Cdh16	0.007078625	0.999256284	0.000323112
Dpep3	0.008692678	0.999256284	0.000323112
Tmed6	0.005448809	0.999256284	0.000323112
Treh	0.006112004	0.999256284	0.000323112
Cyp1a2	0.007268743	0.999256284	0.000323112
Trim42	-0.007867548	0.999256284	0.000323112
Kap	-0.009997104	0.999256284	0.000323112
Cyp2c38	-0.017146089	0.999392842	0.000263766
Olfr281	0.027026918	0.999401342	0.000260072
Hsd17b3	0.012789703	0.999401342	0.000260072
Cst10	-0.017456552	0.999401342	0.000260072
Slc9c1	0.009241348	0.999401342	0.000260072
Magea4	-0.018739508	0.999401342	0.000260072
Acsm1	-0.010506405	0.999401342	0.000260072
Fgb	-0.005196066	0.999401342	0.000260072
Tpbpa	-0.008266649	0.999401342	0.000260072
Olfr1015	0.010885759	0.999401342	0.000260072

Nlrp4b	-0.014042976	0.999401342	0.000260072
Nlrp4d	-0.005789714	0.999401342	0.000260072
Serpini2	0.008649913	0.999401342	0.000260072
Skint2	0.007123344	0.999401342	0.000260072
Ly6g5c	-0.011330826	0.999401342	0.000260072
Prss55	0.006670426	0.999401342	0.000260072
Nlrp2	-0.00719187	0.999401342	0.000260072
Mageb4	0.006103548	0.999401342	0.000260072
Samt3	-0.022942149	0.999401342	0.000260072
Vmn1r172	0.008018364	0.999401342	0.000260072
Olfr1509	0.015155385	0.999401342	0.000260072
Ugt2a3	0.015170151	0.999401342	0.000260072
Ins1	0.005267594	0.999423824	0.000250302
Ugt2b35	0.007971898	0.999425531	0.000249561
Fam122c	-0.005988583	0.999425531	0.000249561
Hoxc9	-0.006580321	0.999425531	0.000249561
Leap2	-0.012291396	0.999438616	0.000243874
Tbc1d21	0.009725801	0.999438616	0.000243874
Glt6d1	-0.006309358	0.999438616	0.000243874
Lcn8	-0.006315813	0.999545949	0.000197236
Akp3	0.014563895	0.999545949	0.000197236
Olfr6	-0.016160862	0.999545949	0.000197236
Olfr11	-0.006376128	0.999545949	0.000197236
Olfr701	-0.006270783	0.999545949	0.000197236
Pramel5	-0.009046575	0.999545949	0.000197236
Noxa1	-0.008452437	0.999545949	0.000197236
Trpv5	0.005658992	0.999632291	0.000159723
Mucl2	0.009017511	0.999632291	0.000159723
Try5	0.006859998	0.99965078	0.000151691
H2-M10.6	0.006407603	0.999718544	0.000122252
Spaca5	0.059246173	0.999778252	9.63146E-05
Banf2	0.018089507	0.999778252	9.63146E-05
Hand1	-0.018947387	0.999778252	9.63146E-05
Taar9	0.011468673	0.999778252	9.63146E-05
Uqcrh-ps1	-0.009156959	0.999778252	9.63146E-05
Slc22a20	0.008001972	0.999778252	9.63146E-05
Prss40	0.012469933	0.999778252	9.63146E-05
H2-M11	0.012891341	0.999778252	9.63146E-05
Tmem247	0.00666737	0.999778252	9.63146E-05
Mbl1	0.05093334	0.999778252	9.63146E-05
Defb7	0.007641296	0.999778252	9.63146E-05
Olfr16	-0.008433478	0.999778252	9.63146E-05
Cldn16	-0.007689438	0.999778252	9.63146E-05
Hand2	0.006872839	0.999778252	9.63146E-05
Hoxa13	0.006352625	0.999778252	9.63146E-05
Itln1	0.005297303	0.999778252	9.63146E-05
Hoxa11	-0.00549416	0.999778252	9.63146E-05
Hoxa9	0.005968666	0.999778252	9.63146E-05

Serpinb9f	-0.007037679	0.999778252	9.63146E-05
Catsper1	-0.005290489	0.999778252	9.63146E-05
Akr1d1	-0.008741551	0.999778252	9.63146E-05
Cyp3a16	0.004434322	0.999778252	9.63146E-05
Mbd3l1	0.008836832	0.999778252	9.63146E-05
Txndc8	-0.01061751	0.999778252	9.63146E-05
Omt2b	0.007753975	0.999778252	9.63146E-05
Scgb3a2	0.009082438	0.999778252	9.63146E-05
Prl3a1	-0.015981436	0.999808139	8.33323E-05
Prl3b1	-0.055446414	0.999818531	7.88183E-05
Spdye4a	-0.019179505	0.999818531	7.88183E-05
Tex19.2	0.009530009	0.999818531	7.88183E-05
Sectm1b	-0.00507561	0.999818531	7.88183E-05
Cylc2	-0.005074746	0.999818531	7.88183E-05
Olfr303	-0.013239615	0.999841753	6.87313E-05
Slc7a12	0.012344158	0.999841753	6.87313E-05
Defb3	0.016634312	0.999841753	6.87313E-05
Defb5	-0.009887332	0.999841753	6.87313E-05
Olfr906	0.008089485	0.999841753	6.87313E-05
Rdh7	0.013033399	0.999841753	6.87313E-05
Nlrp4a	-0.015463638	0.999887826	4.87192E-05
Nlrp9c	-0.018028183	0.99996478	1.52959E-05
Cyp2b9	-0.011987275	0.99996478	1.52959E-05
Arhgef38	0.027227396	1	0
Slc7a13	0.093111812	1	0
Lamp3	-0.034354175	1	0
Tcl1	0.013870173	1	0
Serpina3a	0.009579015	1	0
Obox6	0.008595497	1	0
Iapp	0.012162129	1	0
Oosp1	-0.008485569	1	0
Rptn	0.013002804	1	0
Lce3b	-0.017799368	1	0
Lce1c	0.010918114	1	0
Sprr2d	-0.014482254	1	0
Olfr631	-0.068473936	1	0
Cyp2c37	-0.004448156	1	0
Hoxd11	-0.004586983	1	0
Olfr1353	-0.011257484	1	0
Olfr1032	-0.01464881	1	0
Olfr769	-0.004063096	1	0
Vmn1r226	0.01090953	1	0
Olfr1414	-0.010688386	1	0
Olfr1026	0.008304663	1	0
Olfr1370	0.010720299	1	0
Olfr1260	0.001705956	1	0
Olfr648	0.004666639	1	0
Upk3b	0.005603265	1	0

Ifnk	-0.003338335	1	0
Fscb	0.017083143	1	0
Vmn1r66	0.01170543	1	0
Usp17le	-0.019956906	1	0
Olfr855	-0.008973333	1	0
Olfr448	0.004192303	1	0
Hoxa6	0.007728728	1	0
Olfr1009	-0.015277496	1	0
Olfr1031	-0.019491787	1	0
Olfr1123	0.007151767	1	0
Vmn1r75	0.004082413	1	0
Olfr571	-0.003297382	1	0
Olfr131	-0.012906927	1	0
Olfr30	0.007388729	1	0
Cox8c	0.000717421	1	0
Olfr975	-0.001076944	1	0
Hoxd9	0.00855351	1	0
Olfr187	-0.003460322	1	0
Magea10	-0.002284632	1	0
Lce3d	0.010599517	1	0
Olfr266	0.011045034	1	0
Vmn1r225	0.001288861	1	0
Cldn34c4	-0.000975838	1	0
Olfr13	-0.001688382	1	0
Olfr399	0.005614732	1	0
Olfr62	-0.009792887	1	0
Olfr479	-0.004120374	1	0
Tas2r118	0.00481052	1	0
Olfr323	0.020053709	1	0
Olfr1018	-0.016782406	1	0
Olfr922	0.010246575	1	0
Adam6a	-0.003797552	1	0
Krtap19-4	-0.009257848	1	0
Olfr790	0.007658405	1	0
Olfr1288	0.006081631	1	0
Olfr1497	0.005383401	1	0
Olfr225	0.00742978	1	0
Olfr868	0.008368967	1	0
Olfr1384	0.01390125	1	0
Nkx2-6	0.001637002	1	0
Olfr983	-0.010346797	1	0
Olfr1094	0.010905828	1	0
Defb13	0.006607746	1	0
Vmn1r45	-0.048671113	1	0
Defb29	-0.003913846	1	0
Olfr978	0.006485774	1	0
Olfr794	-0.007761121	1	0
Krt84	0.0068016	1	0

Olfr1442	0.005205122	1	0
Olfr867	0.00616811	1	0
Klk1b11	0.003842167	1	0
Olfr1112	0.001029908	1	0
Olfr1302	0.005302001	1	0
Olfr670	0.009668505	1	0
Defb10	-0.011135145	1	0
Olfr923	0.01199052	1	0
Olfr159	0.006927339	1	0
Defb36	0.004261593	1	0
Olfr821	-0.004258458	1	0
Olfr649	0.000561493	1	0
Psg22	-0.009194917	1	0
Olfr1030	-0.004117993	1	0
Olfr124	-0.015322543	1	0
Olfr711	0.005535424	1	0
Acer1	0.018008971	1	0
Olfr1443	0.000210701	1	0
Krtap4-7	-0.017584519	1	0
Taar6	0.003066731	1	0
Olfr1445	0.005729851	1	0
Olfr620	-0.000112232	1	0
Olfr1255	-0.001168737	1	0
Olfr1161	0.001199234	1	0
Olfr123	-0.008594213	1	0
Olfr835	-0.003530528	1	0
Olfr1152	-0.011206514	1	0
Krtap5-4	0.00843416	1	0
Tas2r119	0.006865134	1	0
Olfr734	0.004941813	1	0
Hsfy2	0.001266157	1	0
Vmn1r70	-0.004724245	1	0
Olfr167	-0.002105561	1	0
Olfr433	0.007672692	1	0
Olfr1499	0.011540039	1	0
Vmn1r230	0.010144885	1	0
Olfr1390	0.001510853	1	0
Olfr1368	0.005864725	1	0
Olfr459	0.000742335	1	0
Olfr1367	0.00550989	1	0
Olfr891	-0.007802903	1	0
Sprr3	-0.002791464	1	0
Olfr600	0	1	0
Olfr827	-0.00901662	1	0
Sprr4	-0.006275871	1	0
Vmn1r233	-0.001253214	1	0
Olfr710	-0.008055193	1	0
Olfr610	-0.001807007	1	0

Tmprss12	-0.009976424	1	0
Olfr1489	-0.005630137	1	0
Lcn6	-0.000587335	1	0
Nlrp4e	-0.000336725	1	0
Olfr447	-0.024404207	1	0
Olfr624	0.00376266	1	0
Olfr578	-0.016493261	1	0
Olfr984	-0.005441312	1	0
Hdgfl1	0.014694323	1	0
Olfr1461	0.009852316	1	0
Olfr803	-0.00596807	1	0
Vmn1r184	-0.009179431	1	0
Olfr918	-0.009382551	1	0
Olfr735	0.012867514	1	0
Sprr2h	0.01208714	1	0
Adam20	0.002782021	1	0
Olfr1412	-0.016274549	1	0
Olfr609	-0.00778541	1	0
Olfr518	0.01193007	1	0
Scgb2b24	0.003464638	1	0
Olfr71	-0.004612211	1	0
Olfr231	0.000991147	1	0
Olfr1352	-0.001954043	1	0
Actrt1	-0.003457226	1	0
Olfr218	0.001366668	1	0
Olfr1440	0.009870007	1	0
Lce1l	0.006875536	1	0
Olfr981	-0.007618968	1	0
Enpp7	0.000918703	1	0
Vmn1r67	0.003611654	1	0
Adam24	-0.002312099	1	0
Olfr1341	-0.001468636	1	0
Phgr1	-0.012547726	1	0
Hnf4aos	0.006987234	1	0
Il1f10	-0.004113656	1	0
Olfr374	-0.001256568	1	0
Prl7a2	-0.002548448	1	0
Olfr1451	0.010151714	1	0
Vmn1r179	0.013083758	1	0
Vmn1r193	-0.006752572	1	0
Spz1	-0.002886018	1	0
Olfr1020	0.004096615	1	0
Catsperb	-0.00109292	1	0
Vmn1r68	0.007627895	1	0
Olfr1151	-0.006767578	1	0
Olfr432	-0.009745886	1	0
Olfr914	0.007597465	1	0
Ube2dnl2	-0.00135581	1	0

Tas2r139	-0.000724988	1	0
Olfr1052	-0.000204128	1	0
Olfr1495	0.005810059	1	0
Olfr684	-0.00480616	1	0
Fbxw21	0.006213931	1	0
Krtap1-5	-0.009689685	1	0
Olfr370	-0.002489961	1	0
Smgc	0.002513928	1	0
Olfr976	-0.004215677	1	0
Lcn10	0.00532624	1	0
Olfr139	0.005969773	1	0
Olfr67	0.011665154	1	0
Olfr616	-0.007723381	1	0
Olfr629	-0.000626976	1	0
Krtap3-1	-0.002635156	1	0
Olfr1122	0.008645558	1	0
Olfr791	0.009053576	1	0
Vmn1r74	0.003514515	1	0
Olfr26	-0.002497713	1	0
Pdha2	0.001775158	1	0
Ccdc144b	0.003946523	1	0
Olfr1388	0.014061915	1	0
Olfr685	-0.006977589	1	0
Oog4	-0.002222179	1	0
Olfr770	-0.003650557	1	0
Serpinb6d	0.007540946	1	0
Olfr186	0.00800009	1	0
Olfr1093	-0.003963804	1	0
Cypt4	-0.01381335	1	0
Krt35	0.025682767	1	0
Fpr-rs4	0.003461089	1	0
Olfr731	0.008806244	1	0
Olfr1324	0.008066278	1	0
Olfr1153	-0.011684208	1	0
Olfr1212	0.008971732	1	0
H2-M10.4	0	1	0
Ovch2	-0.013413541	1	0
Tas2r126	-0.011124622	1	0
Krtap4-13	0.006608837	1	0
Olfr148	0.005527327	1	0
Olfr1496	0.007248374	1	0
Olfr1394	-0.003631951	1	0
Olfr843	-0.006486739	1	0
Olfr686	-0.005426919	1	0
Olfr1448	0.010694865	1	0
Defb15	-0.00270908	1	0
Olfr938	0.007928717	1	0
Adam26a	-0.004044804	1	0

Cypt3	-1.46E-05	1	0
Olfr435	-0.021380545	1	0
Vmn1r26	-0.001660232	1	0
Olfr820	0.00972637	1	0
Skint10	-0.007525276	1	0
Serpinb13	0.00386938	1	0
Olfr202	-0.011855594	1	0
Olfr223	0.005239541	1	0
Olfr1366	-0.006949392	1	0
Olfr729	-0.006725869	1	0
Olfr1467	-0.00200553	1	0
Olfr368	-0.002290788	1	0
Olfr873	0.010397209	1	0
Olfr398	-0.000157583	1	0
Olfr367-ps	-0.004948576	1	0
Olfr147	0.002119356	1	0
Flg2	-0.000813984	1	0
Olfr1258	0.011924459	1	0
Olfr449	0.010399857	1	0
Olfr788	0.00107604	1	0
Olfr509	-0.002658325	1	0
Olfr348	-0.0049064	1	0
Olfr902	0.002141574	1	0
Zg16	0.005807934	1	0
Olfr173	0.008057447	1	0
Olfr1183	0.004520945	1	0
Olfr1404	-0.003354653	1	0
Olfr1449	0.001152831	1	0
Olfr429	-0.003355752	1	0
Olfr780	0.008987193	1	0
Lce1h	0.004321132	1	0
Olfr418	-0.008441338	1	0
Olfr103	-0.003692728	1	0
Olfr714	0.00058995	1	0
Cyp2g1	-0.010452893	1	0
Olfr27	0.008383178	1	0
Olfr1361	0.002690071	1	0
Olfr1318	-0.004881477	1	0
Olfr642	-0.005999159	1	0
Olfr466	-0.001923953	1	0
Krtap9-3	-0.003734118	1	0
Olfr250	0.003673187	1	0
Olfr808	-0.008834456	1	0
Skint9	0.026895127	1	0
Olfr350	0.00185287	1	0
Olfr1176	-0.004301979	1	0
Olfr745	-0.00834114	1	0
Olfr728	-0.005597312	1	0

Akap4	0.005601871	1	0
Sprr2b	0.007834622	1	0
Olfr1023	0.001634592	1	0
Olfr430	-0.009113939	1	0
Olfr165	-0.00836194	1	0
Olfr768	-0.006383838	1	0
Krtap13	0.002875448	1	0
Krtap24-1	-0.005467849	1	0
Olfr809	-8.36E-05	1	0
Olfr630	-0.006012606	1	0
Hoxc12	-0.015234656	1	0
Olfr524	0.004943302	1	0
Hoxd10	0.002900379	1	0
Ube2dnl1	0.004003857	1	0
Hamp	0.008350537	1	0
Olfr807	0.000379387	1	0
Olfr1323	-0.001863048	1	0
Gk2	0.007158339	1	0
Olfr1038-p	0.010972763	1	0
Olfr1008	0.014884425	1	0
Olfr125	0.005045187	1	0
Sprr2f	0.003242951	1	0
BC048562	-0.006679253	1	0
Defb19	-0.014633943	1	0
Mrgpra1	-0.008964251	1	0
Ccdc54	-0.003680572	1	0
Olfr164	-0.003469871	1	0
Ear14	-0.001510134	1	0
Olfr1124	-0.002831137	1	0
Olfr1317	-0.007102356	1	0
Olfr419	0.001424082	1	0
Olfr866	0.00683246	1	0
Oog3	-0.002306625	1	0
Olfr332	0.000581924	1	0
Olfr1441	0.007026341	1	0
Olfr330	-0.001278848	1	0
Olfr958	-0.010302748	1	0
Olfr1494	0.005871321	1	0
Mrgprb8	-0.002327222	1	0
Mtnr1b	0.009463709	1	0
Fgf4	-0.002412871	1	0
Vmn1r231	-0.001841527	1	0
AY358078	0.004158994	1	0
Cetn1	-0.008574809	1	0
Olfr161	0.006573044	1	0
Rhox11	-0.003375497	1	0
Spink14	0.001358076	1	0
Olfr77	-0.000605724	1	0

Tas2r105	-0.003904399	1	0
Olfr1491	-0.002528848	1	0
Olfr853	0.005819547	1	0
Olfr672	-0.006774303	1	0
Zfp42	-0.00612495	1	0
Olfr522	0.000260764	1	0
Olfr655	-0.00737169	1	0
Olfr1356	0.003536423	1	0
Mrgprd	0.002115766	1	0
Olfr1362	-0.002327141	1	0
Actrt2	0.003586474	1	0
Olfr1262	0.004502624	1	0
Olfr645	0.007225657	1	0
Olfr589	0.003861793	1	0
Olfr1402	-0.009349621	1	0
Olfr829	-0.00621469	1	0
Olfr1184	-0.002738748	1	0
Ubqlnl	0.000412531	1	0
Krtap31-2	0.004659434	1	0
Olfr974	0.004872141	1	0
Olfr424	-0.002086888	1	0
Olfr697	0.000266203	1	0
Olfr272	0.004526845	1	0
Ubqln3	-0.002063547	1	0
Olfr693	0.001538679	1	0
Vmn1r73	-0.006079874	1	0
Olfr1325	0.008759175	1	0
Wfdc21	0.010469318	1	0
Olfr284	0.009942695	1	0
Krtap19-5	-0.000867506	1	0
Adam6b	-0.00868675	1	0
Rhox2a	0.00444131	1	0
Vmn2r72	0.002795103	1	0
Tex37	0.006215373	1	0
Abca16	0.00412229	1	0
Tas2r144	-9.02E-05	1	0
Prss58	-0.009539534	1	0
Olfr371	0.000906041	1	0
Olfr796	0.005811307	1	0
Olfr901	0	1	0
Olfr849	-0.003015236	1	0
Hbb-bh1	0.005020052	1	0
Hoxd3os1	0	1	0
Rnase9	0.00156363	1	0
Olfr720	-0.010716071	1	0
Cyp2j5	-0.002971488	1	0
Olfr198	0.002707712	1	0
Arl13a	-0.004636855	1	0

Defb34	-0.000514033	1	0
Olfr851	0.004810607	1	0
Olfr813	0.001175886	1	0
Adh6a	-0.00069622	1	0
Olfr352	0.014766503	1	0
Cib4	0.024344551	1	0
Tas2r136	-0.00618392	1	0
Olfr212	9.81E-05	1	0
Olfr1013	-0.009691519	1	0
Slc22a26	-0.007813471	1	0
Tas2r107	0.005200251	1	0
Olfr211	-0.007746889	1	0
Gykl1	-0.000170211	1	0
Defb40	0.002525625	1	0
Defb37	-0.013374899	1	0
Klk1b26	0.002125411	1	0
Vmn2r43	-0.005824995	1	0
Spinkl	0.002528926	1	0
Defb38	-0.006886446	1	0
Olfr744	0.001501407	1	0
Bcl2a1c	-0.003426279	1	0
Dnajc5g	0.001587236	1	0
Slc51b	-0.004174873	1	0
Ang5	0.002380547	1	0
Mill1	0.007014762	1	0
Adam39	-0.004452422	1	0
Olfr279	-0.019474372	1	0
Olfr309	0.005149674	1	0
Fbxw28	-0.004468721	1	0
Nlrp9a	-0.006154758	1	0
Try4	0.004302447	1	0
Zdhhc25	-0.001142636	1	0
Vmn1r236	-0.004094062	1	0
Sprr2k	0.002791678	1	0
Olfr481	0.001703238	1	0
Serpinb9d	-0.008126314	1	0
Zscan4c	-0.005591175	1	0
Obox1	0.005177031	1	0
Olfr411	-0.005233182	1	0
Cyp3a44	0.003856926	1	0
Fabp1	0.009976437	1	0
Olfr450	-0.003854153	1	0
Cpa1	-0.004422899	1	0
Tas2r130	0.004345334	1	0
Olfr308	0.017350322	1	0
Ms4a5	0.00055	1	0
Olfr1500	0.007643417	1	0
Tmprss11[illegible]	0.002964975	1	0

Usp17la	-0.002796627	1	0
Olfr63	-0.000386063	1	0
AU018091	0.009798637	1	0
Akr1c20	-0.002944904	1	0
Cyp2c50	0.010390471	1	0
Stfa3	0.006141219	1	0
Olfr1346	-0.004247013	1	0
Olfr137	0.006564321	1	0
Sprr2e	0.005408536	1	0
Olfr420	-0.013830013	1	0
Olfr354	0	1	0
Spink6	0.002247806	1	0
Klk15	0.004380064	1	0
Ctsj	0.009508542	1	0
Rnf212	0.004646782	1	0
Hottip	-0.000356459	1	0
Vmn2r81	-0.000565409	1	0
Olfr305	0.003605505	1	0
Hba-x	0.004814452	1	0
Olfr307	0.002972472	1	0
Ctsr	0.004130801	1	0
Ces2a	-0.00420692	1	0
Magea2	-0.009046597	1	0
Gsdmc3	-0.014578212	1	0
Olfr357	0.002594029	1	0
Oosp3	-0.003099388	1	0
Obox7	0.000304921	1	0
BC05107€	0.007010872	1	0
Olfr378	0.00315307	1	0
Ccdc7b	-0.010820855	1	0
Gata5os	0.000906695	1	0
Tas2r134	-0.004733419	1	0
Spata31	-0.000880878	1	0
Prr9	-0.001147883	1	0
Olfr1322	0.005351113	1	0
Gsdmc2	0.00172173	1	0
Prl2c3	-0.009412174	1	0
Mettl7a2	0.025438123	1	0
Defb21	0.002009139	1	0
Krt72	0.001279808	1	0
Olfr1350	-0.0004928	1	0
Ctsll3	0.005259104	1	0
Olfr667	0.005847793	1	0
Olfr166	0.003325679	1	0
Olfr765	-0.006886455	1	0
Olfr1502	0.000458044	1	0
Olfr702	0.002284999	1	0
Tas2r102	0.005049305	1	0

Vmn2r107	-0.001216663	1	0
Olfr394	0.00147225	1	0
Olfr919	-0.003307774	1	0
Olfr1178	-0.000653694	1	0
Cfhr1	0.010714779	1	0
Olfr397	0.004255439	1	0
Olfr297	-0.011762711	1	0
Olfr1301	-0.007727096	1	0
Prl3d1	0.005487899	1	0
Krtap19-9l	-0.001013816	1	0
Olfr747	0.004348854	1	0
Ceacam13	-0.004497502	1	0
Vmn1r234	0.001294173	1	0
Olfr1501	0	1	0
Usp17ld	-0.001911383	1	0
Olfr948	0.001564265	1	0
Ces1c	-0.005209218	1	0
Olfr934	-0.004550492	1	0
Olfr138	0.001790711	1	0
Olfr915	-0.008970261	1	0
Cts8	0.002459095	1	0
Olfr1205	0.011782464	1	0
Olfr591	-0.012683013	1	0
Olfr1415	-0.002372575	1	0
Olfr1459	0.002519688	1	0
Vmn1r177	0.005200803	1	0
Olfr310	-0.00531235	1	0
Vmn1r48	0.002254127	1	0
Krtap19-2	6.94E-05	1	0
Olfr328	0.00484792	1	0
Vmn1r53	-0.001432885	1	0
Tas2r131	-0.001606531	1	0
Serpinb9g	0.013361048	1	0
Tas2r106	-0.001311687	1	0
Olfr1022	0.005308668	1	0
Olfr135	0.005484409	1	0
Olfr1446	-0.003429998	1	0
Olfr739	0.003375318	1	0
Skint11	4.35E-05	1	0
Vmn1r12	-0.00362852	1	0
Olfr541	0.002956598	1	0
Tdpoz3	0.003976242	1	0
Olfr502	0.004182864	1	0
Serpinb3d	0.013073976	1	0
Vmn1r237	-0.003466378	1	0
Defb35	0.005939932	1	0
Olfr818	-0.005082797	1	0
Olfr825	0.003934477	1	0

Olfr127	0.003537113	1	0
H2-M10.3	-0.004557918	1	0
Vmn1r82	0.004752434	1	0
Krtap6-1	-0.003383084	1	0
Olfr746	0.004837551	1	0
Olfr1126	-0.002948954	1	0
Olfr66	0.008233282	1	0
Serpina3k	-0.009710664	1	0
Olfr506	-0.000531877	1	0
Tas2r138	-0.007108892	1	0
Olfr822	-0.002303744	1	0
Olfr25	-0.000764156	1	0
Olfr412	0.006101594	1	0
Tas2r117	-0.00193739	1	0
Krtap21-1	0.001190961	1	0
Btnl4	-0.003327912	1	0
Olfr869	0.002106341	1	0
Pip	-0.007987969	1	0
Olfr801	0.005918065	1	0
Olfr933	0.000607267	1	0
Dppa4	0.002072726	1	0
Defb50	-0.008644993	1	0
Vmn1r63	-0.003822058	1	0
Speer4f1	-0.008149673	1	0
Olfr58	0.007351672	1	0
Olfr69	-0.003895616	1	0
Vmn2r51	-0.00059406	1	0
Olfr846	-0.001746542	1	0
Olfr97	-0.000678633	1	0
Olfr331	0.001969017	1	0
Olfr146	-0.009474296	1	0
Olfr952	0.003949968	1	0
Olfr1413	-0.000665273	1	0
Usp17lc	-0.005359569	1	0
Olfr1406	0.005058538	1	0
Olfr1201	-0.000618496	1	0
Olfr128	0.001713956	1	0
Olfr482	0.004244506	1	0
Klk1b9	-0.008768204	1	0
Olfr15	-0.004615594	1	0
Olfr749	0	1	0
Olfr698	0.004764928	1	0
Olfr1504	0	1	0
Olfr961	0.008303063	1	0
Olfr1263	0.004245022	1	0
Lrrc74a	-0.005394402	1	0
Ifnl2	-0.004058857	1	0
Olfr814	-0.002385645	1	0

Krt40	-0.003948373	1	0
Olfr913	0.004755202	1	0
Olfr1130	0.008815965	1	0
Vmn1r71	0.00434532	1	0
Defb4	-0.002376795	1	0
Olfr345	0.007827156	1	0
Olfr836	0.001112082	1	0
Olfr295	-0.003705457	1	0
Aldoart1	0.001446471	1	0
Olfr959	-0.006691603	1	0
Olfr427	0.019231478	1	0
Vmn1r33	0.010083542	1	0
Olfr1014	0.002424626	1	0
Tas2r120	-0.003955325	1	0
Olfr434	-0.007556495	1	0
Olfr365	0.009417769	1	0
Spag11b	-0.004519694	1	0
Olfr979	-0.009008666	1	0
Olfr248	0.006587457	1	0
Olfr314	0.004937856	1	0
Fbxw26	-0.007052657	1	0
Olfr935	0.017120284	1	0
Rnase2b	-0.004796969	1	0
Olfr39	0.0067771	1	0
Rhox3e	0.008268226	1	0
Krtap8-1	0.004519192	1	0
Rnase11	-0.005235682	1	0
Reg1	0.001823993	1	0
Krt4	0.006800158	1	0
Olfr108	-0.007243492	1	0
Olfr1385	0.001557444	1	0
Olfr767	0.004674439	1	0
Taar2	0.001787212	1	0
Olfr847	-0.00614917	1	0
Kprp	0.00691481	1	0
Olfr826	0.006469138	1	0
Olfr960	-0.002147313	1	0
Olfr603	0.000976641	1	0
Olfr1265	-0.000576517	1	0
Olfr119	0.004020801	1	0
Olfr121	-0.006593059	1	0
Vmn1r213	0	1	0
Olfr193	0	1	0
Defa26	-0.004010221	1	0
Olfr748	-0.001074463	1	0
Olfr504	0.01228655	1	0
Olfr60	0.004218076	1	0
Olfr910	0.010441532	1	0

Spink7	0.007508341	1	0
Defa17	-0.003302618	1	0
Vmn1r228	0.003646562	1	0
Olfr980	0.000366563	1	0
Tdpoz4	0.01694151	1	0
Olfr1447	6.75E-05	1	0
Olfr384	-0.005014972	1	0
Cyp2a12	0	1	0
Tas2r124	-0.003579783	1	0
Krtap19-3	-0.002884757	1	0
Olfr171	0.010259138	1	0
Rpl10l	0	1	0
Olfr715	0.000225255	1	0
Nlrp9b	-0.006866614	1	0
Olfr726	-0.00409837	1	0
Olfr1418	0.00058451	1	0
Olfr881	0.004118701	1	0
Cyp2c70	-0.002956753	1	0
Ang4	-0.000758204	1	0
Zfp735	-0.010620767	1	0
Fthl17c	-0.003418488	1	0
Olfr292	0.006727157	1	0
Krtap19-1	0	1	0
Vmn1r35	0	1	0
Fpr-rs3	0.006415372	1	0
Vmn1r47	0.003767485	1	0
Tmsb15a	0.002080043	1	0
Prl7c1	0.005293528	1	0
Olfr1076	-0.009738967	1	0
Ifnl3	-0.006416582	1	0
Olfr503	0.002832667	1	0
Olfr325	-0.000392578	1	0
Serpina6	-0.000905903	1	0
Vmn1r52	-0.010469215	1	0
Olfr1193	0.00208504	1	0
Olfr51	0.001294238	1	0
Etd	0.001751585	1	0
Olfr530	0.007845144	1	0
Olfr507	0.007117288	1	0
Vmn1r222	-0.004514742	1	0
Olfr160	0	1	0
Tmprss11	-0.00020035	1	0
Olfr1289	0.002185107	1	0
Vmn1r8	-0.004906353	1	0
Olfr47	-0.001882186	1	0
Cyp3a59	-0.0020085	1	0
Olfr1261	0	1	0
Olfr116	0.000553351	1	0

Olfr1168	0.011178269	1	0
Olfr168	0.001836861	1	0
Olfr771	-0.004498861	1	0
Vmn1r219	0.003503658	1	0
Olfr1490	-0.005432226	1	0
Csn1s2b	-0.00453923	1	0
Mageb5	0.016035445	1	0
Olfr871	-0.011719932	1	0
Olfr525	-0.001048188	1	0
Olfr301	-0.000609657	1	0
Olfr373	0.006544049	1	0
Vmn1r78	0.00094409	1	0
Olfr845	-0.000880636	1	0
Olfr12	0.001980102	1	0
Olfr68	0.002664798	1	0
Olfr1457	0	1	0
Vmn1r46	-0.003710453	1	0
Fbxw20	-0.003267446	1	0
Cfd	-0.009618914	1	0
Olfr1204	-0.000202603	1	0
Tbpl2	0.010685385	1	0
Ces2c	0.000423273	1	0
Vmn1r214	0	1	0
Defa35	0.014479044	1	0
Defb39	-0.001722933	1	0
Olfr1148	0.00351713	1	0
BC048679	0.004563972	1	0
Csn1s2a	0.021343729	1	0
Olfr815	-0.018297256	1	0
Cldn24	-0.005742385	1	0
Tas2r104	-0.001389251	1	0
Olfr392	0.000145903	1	0
Olfr285	-0.005055025	1	0
Olfr294	-0.001648929	1	0
Slc2a7	0.00026224	1	0
Olfr924	-4.10E-05	1	0
Olfr183	-0.005893023	1	0
Olfr149	0.00489507	1	0
Defb45	-0.005753248	1	0
Olfr20	0	1	0
Olfr569	-0.001123564	1	0
Ear6	-0.006973149	1	0
Mageb1	-0.009628539	1	0
Vmn1r232	-0.008789774	1	0
Ces3b	-0.003210487	1	0
Olfr395	0.001503513	1	0
Olfr1465	0.002536296	1	0
Vmn2r7	-0.001083182	1	0

Prl3d3	-0.003351335	1	0
Olfr1373	-0.004807577	1	0
Olfr170	-0.000225114	1	0
Olfr1129	0.000226609	1	0
Fbxw24	-0.003907411	1	0
Olfr1505	-0.00091625	1	0
Serpinb9e	0.005322974	1	0
Usp17lb	-0.015894277	1	0
Krtap6-5	-0.006359663	1	0
Olfr304	0.014022891	1	0
Krtap6-2	-0.001414274	1	0
Olfr516	-0.006093665	1	0
Vmn1r171	-0.003824546	1	0
Olfr1411	0.007437854	1	0
Olfr1408	-0.000718724	1	0
Tas2r109	0.006221897	1	0
V1ra8	0	1	0
Prl2c1	0.002049718	1	0
Scgb1b2	0.003327202	1	0
Tex28	0	1	0
Vmn1r178	0.001520554	1	0
Olfr911-ps	0.00659716	1	0
Cyp2c67	-0.009870349	1	0
Olfr44	0.001130463	1	0
Tpbpb	-0.001662008	1	0
Olfr531	0.007700215	1	0
Lypd4	-0.000394186	1	0
Prl3d2	-0.001749529	1	0
Olfr1206	-0.000241594	1	0
Tex101	-0.001166226	1	0
Olfr527	-0.002854108	1	0
Olfr1158	-0.00171551	1	0
Ssxa1	0.005079904	1	0
Vmn1r51	-0.007551187	1	0
Sval3	0.002462049	1	0
Olfr1458	0.003709097	1	0
Olfr830	0	1	0
Olfr1355	0.007953489	1	0
Olfr298	-0.005997954	1	0
Olfr1450	0.001961882	1	0
Olfr777	-0.002906681	1	0
Tas2r110	0	1	0
Olfr194	-0.005364124	1	0
Klk1b8	-0.000107555	1	0
Olfr410	-0.00370984	1	0
Olfr480	-0.00124755	1	0
Klk1b1	-0.004912893	1	0
Olfr177	0.010340744	1	0

Olfr811	0.003905845	1	0
Olfr955	0.003530932	1	0
Olfr107	-0.000561805	1	0
Defa34	0.01179165	1	0
Olfr1351	0.010161404	1	0
Olfr930	-0.001036197	1	0
Olfr535	0.003259416	1	0
Serpina11	0.004307213	1	0
Olfr133	0.014313623	1	0
Psg20	-0.002984287	1	0
Olfr763	0.004988815	1	0
Ifna13	0.011814789	1	0
Olfr945	-0.010868473	1	0
Olfr1387	-0.002548002	1	0
Olfr293	0.00395484	1	0
Tas2r114	0.008013411	1	0
Olfr322	0.006800112	1	0
Olfr666	0.011329135	1	0
Olfr1410	0.001055522	1	0
Olfr64	-0.001224617	1	0
Olfr98	0.00650364	1	0
Olfr816	-7.23E-05	1	0
Magea6	0.005000834	1	0
Tas2r129	-0.004764862	1	0
Olfr508	0.007769445	1	0
Olfr1469	0.002231179	1	0
Olfr282	-0.002632348	1	0
Olfr532	0.002297165	1	0
Olfr1382	0.008343625	1	0
Olfr862	0.018891819	1	0
Olfr1276	0.000874679	1	0
Olfr376	0.002070618	1	0
Adam26b	0.002967702	1	0
Cyp4a32	-0.01132762	1	0
Olfr120	0.007581246	1	0
Vmn1r211	0.00179661	1	0
Olfr190	-0.006808005	1	0
Olfr319	-0.008740459	1	0
Olfr1202	-0.000890928	1	0
Olfr963	0.004376899	1	0
Rhox8	0.006010808	1	0
Krt39	-0.008660568	1	0
Zfp936	-0.003607246	1	0
Defa24	-0.000498194	1	0
Olfr694	-0.002477816	1	0
Vmn1r13	-0.008322617	1	0
Platr26	-0.005557173	1	0
Olfr926	-0.008319457	1	0

DQ26710:	-0.004211271	1	0
Snord49b	-0.01169774	1	0
Snord37	-0.001774808	1	0
Snord68	-0.004474971	1	0
Snora28	-0.001617702	1	0
DQ26710:	-0.00383466	1	0
Snord42a	0.00137969	1	0
Snora7a	-0.001982586	1	0
Snora41	0	1	0
Snora44	-0.008003404	1	0
Snora20	0	1	0
Snora52	0	1	0
Snord96a	0.001964801	1	0
Snord45b	-0.001431387	1	0
Snord14a	0	1	0
Snord35b	0.008957813	1	0
Snord95	0.007083474	1	0
Snord82	0.001812831	1	0
Snora75	0.003161706	1	0
Snord58b	0	1	0
Snord118	0.002807538	1	0
Snora21	0.005742541	1	0
Snora62	0.023070581	1	0
Rny3	-0.000742566	1	0
Snora61	0.006007684	1	0
Snord15b	-0.005823191	1	0
Snord47	0.005465597	1	0
Snord73a	0.006671741	1	0
Snora3	-0.008540506	1	0
Snord22	0.000109799	1	0
Snord1a	0.000440028	1	0
Snora16a	0.007366127	1	0
Snord61	0	1	0
Snora65	0.002902954	1	0
Snord104	-0.005202273	1	0
Snora31	0.003855188	1	0
Rnu12	0.007814586	1	0
Snord85	-0.005083445	1	0
Snord32a	-0.00286579	1	0
Snora30	0.002068994	1	0
Snord57	-0.006909422	1	0
Snora73b	-0.000935308	1	0
Mir25	0.000447271	1	0
Mir193a	-0.004411844	1	0
Mir99b	-0.005972444	1	0
Mir155	-0.006203682	1	0
Mir188	-0.003274771	1	0
Mir133a-1	0.007203231	1	0

Mir200a	0.006415904	1	0
Mir144	0.003966769	1	0
Mir122	-0.001948635	1	0
Mir18	0.011754487	1	0
Mir201	0.000591897	1	0
Mir30a	0.002603831	1	0
Mirlet7i	0	1	0
Mir135a-1	-0.002397103	1	0
Mir31	-0.010333681	1	0
Mir298	-0.01030683	1	0
Mir195a	-0.00394598	1	0
Mir29b-2	0.005569984	1	0
Mir367	0.002239189	1	0
Mir138-1	0.005637232	1	0
Mir217	0.002106532	1	0
Mir19a	-0.007162297	1	0
Mir340	-0.00384476	1	0
Mir322	0.002119454	1	0
Mir142b	0.001904006	1	0
Mirlet7a-1	-0.005710906	1	0
Mir221	-0.003009096	1	0
Mir181a-2	-0.002896539	1	0
Mir9-2	-0.00509223	1	0
Mir325	0.006149236	1	0
Mir134	-0.003131762	1	0
Mir429	-0.009497557	1	0
Mir382	-0.005596428	1	0
Mir345	-0.005424561	1	0
Mir26a-2	0.000149097	1	0
Mir186	-0.013021686	1	0
Mir208a	0.003145004	1	0
Mir370	0	1	0
Mir7-1	-0.011541013	1	0
Mir135b	-0.003015327	1	0
Mir342	0.000332856	1	0
Mir30d	0.001056952	1	0
Mir377	-0.005554848	1	0
Mir140	-0.003651317	1	0
Mirlet7g	0.008056079	1	0
Mir128-2	-0.013811846	1	0
Mir20a	-0.003790403	1	0
Mir196b	0	1	0
Mir27a	-0.000681423	1	0
Mir143	0.004367716	1	0
Mir139	0.003637041	1	0
Mir302d	-0.000513775	1	0
Mir154	0.003633702	1	0
Mir365-1	-0.001428192	1	0

Mir448	0.010048542	1	0
Mir101a	0.000834222	1	0
Mir207	0.009183247	1	0
Mirlet7d	0.004910344	1	0
Mir124a-3	-0.003595232	1	0
Mir21a	-0.002619833	1	0
Mir106a	-0.001835715	1	0
Mir383	-0.006298976	1	0
Mir181b-1	-0.001610889	1	0
Mir133a-2	-0.002184956	1	0
Mir200c	0.008108852	1	0
Snord45c	0	1	0
Mir185	0.000642838	1	0
Mir33	-0.004365305	1	0
Mir26b	0.002200943	1	0
Mir129-1	0.000553719	1	0
Mir149	-0.001631322	1	0
Mir222	0.014728217	1	0
Mir125b-2	0	1	0
Mir19b-2	-0.004749635	1	0
Mir141	-0.001952079	1	0
Mir27b	0.00984417	1	0
Mir30b	0	1	0
Mir411	0.001751098	1	0
Mir125a	-0.005670963	1	0
Mir133b	-0.000780596	1	0
Mir346	0.004731279	1	0
Mir302c	-0.005983082	1	0
Mir181c	0.000313391	1	0
Mir130a	-0.006466221	1	0
Mir219a-2	-0.002114655	1	0
Mir450-1	0.000367699	1	0
Mir196a-2	-3.97E-05	1	0
Mir365-2	0.005834592	1	0
Mir30c-1	0.003237283	1	0
Mir7b	-0.004305114	1	0
Mir34b	0.006099241	1	0
Mir34a	0.004624256	1	0
Mir28a	0	1	0
Mir150	0.001200909	1	0
Mir410	-0.007600764	1	0
Mir379	-0.001127538	1	0
Mir384	-0.002860913	1	0
Mir10b	-0.000558718	1	0
Mir335	0.0103216	1	0
Mir202	0	1	0
Mir351	0.001344277	1	0
Mir148a	0	1	0

Mir204	0	1	0
Mir17	0	1	0
Mir421	-0.005287633	1	0
Mir361	-0.017163056	1	0
Mir129-2	0.007246182	1	0
Mir138-2	0.00436134	1	0
Mir26a-1	-0.002750742	1	0
Mir106b	0.004177592	1	0
Mir152	0.003791197	1	0
Mir214	0.002957599	1	0
Mir423	0	1	0
Mir10a	0.00342495	1	0
Mir128-1	0.000630931	1	0
Mir296	0.011326889	1	0
Mir192	0.000651504	1	0
Mir135a-2	-0.002097214	1	0
Mir337	-0.007015527	1	0
Mir93	-0.001820858	1	0
Mir320	0.005921993	1	0
Mir22	0.002442842	1	0
Mir99a	0.004062864	1	0
Mir187	0.001509263	1	0
Mir205	-0.00436447	1	0
Mir324	0.003161584	1	0
Mir98	0.019603553	1	0
Mir132	0.006214874	1	0
Mir153	-0.005096389	1	0
Mir216a	-0.009217943	1	0
Mir126a	-0.003410573	1	0
Mir24-2	0.011762527	1	0
Mir224	-0.022298203	1	0
Mir330	0.002757599	1	0
Mir32	-0.004865486	1	0
Mir196a-1	0.007691216	1	0
Mir199a-1	0.000718338	1	0
Mir29c	0	1	0
Mir200b	0	1	0
Mir210	-0.001782803	1	0
Mir302a	0.005394395	1	0
Mir103-1	0	1	0
Mir219a-1	0.000364077	1	0
Mir101b	-0.005287691	1	0
Mirlet7c-1	0	1	0
Mir206	0.000173964	1	0
Mir148b	0	1	0
Mir369	-3.59E-05	1	0
Mir215	-0.002670935	1	0
Mir103-2	-0.000172249	1	0

Mirlet7b	-0.008250656	1	0
Mir181a-1	0.001725931	1	0
Mir30c-2	-0.001502745	1	0
Mir344	0.003548862	1	0
Mir137	0	1	0
Mir412	0.007909662	1	0
Mir326	0.001061192	1	0
Mir130b	0.001557599	1	0
Mir350	-0.001172646	1	0
Mir203	0.004708025	1	0
Mir449a	-0.00094876	1	0
Mir329	-0.00434035	1	0
Mir181b-2	-0.000309163	1	0
Mir425	0.00994941	1	0
Mir15b	-0.001894328	1	0
Mir194-1	-0.002049991	1	0
Mir194-2	0.000234912	1	0
Mir218-2	-0.00414742	1	0
Mir211	0.00493396	1	0
Mir96	0.005428885	1	0
Mir34c	-0.004921108	1	0
Mir301	-7.16E-05	1	0
Mir212	-0.003335777	1	0
Mir145a	0.005817892	1	0
Mir339	0.003970461	1	0
Mir107	-0.001653588	1	0
Mir184	-0.002120533	1	0
Mir124a-1	-0.002801676	1	0
Mir23b	-0.005572729	1	0
Mir338	-0.002887777	1	0
Mir146	0	1	0
Mirlet7f-2	-0.005946166	1	0
Mir218-1	0.009920718	1	0
Mir29b-1	-0.003605474	1	0
Mir16-2	0.001276152	1	0
Mir331	-0.009744161	1	0
Mirlet7c-2	0.000306284	1	0
Mir7-2	-0.013628205	1	0
Mir29a	0.004336689	1	0
Mir23a	0.006462505	1	0
Mir151	-0.000742868	1	0
Mir92-2	0	1	0
Mir375	0.006760829	1	0
Mir323	-0.002857826	1	0
Mir183	0.001639117	1	0
Snord1c	-0.011710566	1	0
Snora69	-0.005901807	1	0
Snora74a	0	1	0

Snord42b	0.001864162	1	0
Snord38a	0.003466172	1	0
Snora5c	-0.003419137	1	0
Rny1	0.005783892	1	0
Snord7	-0.00332021	1	0
Snord49a	-0.006793944	1	0
Snord35a	0.004757407	1	0
Snord15a	0.000426846	1	0
Snord34	0.004179109	1	0
Snora2b	0.001237816	1	0
Defa37	0.004457119	1	0
Oog2	0.001270694	1	0
Olfr1335	-0.006879322	1	0
Cyp4a12a	-0.002942777	1	0
Cyp4a10	-0.002390313	1	0
Cyp2j11	0.00019593	1	0
Krtap5-1	0.006217029	1	0
Olfr45	0	1	0
Mup21	-0.001393566	1	0
Olfr519	0.008836028	1	0
Olfr517	0	1	0
Olfr514	0.004807953	1	0
Olfr467	-0.000280989	1	0
Olfr206	-0.003196549	1	0
Olfr640	0.007731837	1	0
Olfr586	0.000890098	1	0
Olfr575	0	1	0
Tcl1b1	-0.005730236	1	0
Serpina3b	0	1	0
Serpina1a	-0.003541892	1	0
Vmn2r65	-0.006963062	1	0
Omt2a	-0.002186286	1	0
Izumo2	-0.006626865	1	0
Klk1b5	0.000699396	1	0
Klk1b4	-0.0076706	1	0
Klk1b3	0	1	0
Klk1b21	0.009921459	1	0
Scgb1b27	0.002104963	1	0
Scgb2b27	-0.000633817	1	0
Scgb2b26	-0.000498319	1	0
Olfr220	-0.003161943	1	0
Olfr417	0	1	0
Cyp2b19	0	1	0
Olfr145	0.001070044	1	0
Obox3	-0.007495313	1	0
Vmn1r84	-0.002958939	1	0
Vmn1r83	0.003958351	1	0
Vmn2r28	-0.00890981	1	0

Vmn2r37	0.009091412	1	0
Vmn1r65	-0.008127747	1	0
Olfr872	-0.002572002	1	0
Olfr870	-0.0052002	1	0
Olfr860	0.000934254	1	0
Vmn2r10	-0.006444352	1	0
Olfr1416	0.002146353	1	0
AY702103	0	1	0
Mrgpra4	-0.00543397	1	0
Olfr132	0.004821728	1	0
H2-M9	0.005117457	1	0
Cyp2c54	0	1	0
Cyp2c66	0.002063886	1	0
Crygd	0.007230263	1	0
Olfr1436	-0.00427045	1	0
Olfr76	0.009698403	1	0
Olfr1427	-0.006507123	1	0
Olfr1424	-0.00674455	1	0
Prb1	0.004556524	1	0
Dmrtc1c2	0.004927595	1	0
Dmrtc1c1	0.00184292	1	0
Krt81	0.000837506	1	0
Slc22a27	0.001530053	1	0
Obp1b	-0.004100646	1	0
Obp1a	-0.004478969	1	0
Wfdc13	0.001268242	1	0
Clec4b2	0.019004336	1	0
Defb41	0.009543428	1	0
Slxl1	0.001914474	1	0
Vmn1r227	-0.001982834	1	0
Olfr1321	-0.002790009	1	0
Rhox9	-0.003157714	1	0
Cyp2d11	-0.002701689	1	0
Cyp2d9	-0.001017731	1	0
Olfr203	-0.006559777	1	0
Ssxb9	-0.004114756	1	0
Ssxb10	0	1	0
Vmn1r42	-0.001612159	1	0
Vmn1r44	-0.000202971	1	0
Noto	0.004207967	1	0
Reg3d	-0.002352497	1	0
Olfr742	0.003554521	1	0
Olfr725	-0.003774692	1	0
Olfr169	0.006902766	1	0
Olfr458	-0.005227598	1	0
Gml2	-0.016126956	1	0
Olfr1278	-0.005511363	1	0
Olfr1259	-0.000328223	1	0

Olfr1189	0.000474374	1	0
Olfr1188	-0.006343408	1	0
Olfr1145	0.002108111	1	0
Olfr1143	-0.000110513	1	0
Olfr152	-0.010430782	1	0
Olfr1140	0	1	0
Olfr1131	-0.001119449	1	0
Lce1j	-0.001263875	1	0
Lce1e	0.012004558	1	0
Lce1a2	-0.003327433	1	0
Tcl1b3	0.00413125	1	0
Olfr366	-0.003073918	1	0
Olfr338	0.003835569	1	0
Sry	0.002515285	1	0
Usp9y	0.001863179	1	0
Lcn11	0.007983088	1	0
Prl2b1	0.002502532	1	0
Prl6a1	-0.006358442	1	0
Vmn1r223	-0.0017216	1	0
Vmn1r203	0.001011081	1	0
Vmn1r199	-0.007224099	1	0
Vmn1r197	0.003896135	1	0
Vmn1r196	-0.004431887	1	0
Vmn1r195	0.006523729	1	0
Vmn1r194	0	1	0
Vmn1r188	0.011444333	1	0
Olfr707	0	1	0
Olfr810	0	1	0
Olfr9	-0.001898334	1	0
Zfp616	0	1	0
Krtap10-4	0.001842762	1	0
Krtap12-1	0.005690607	1	0
Krtap3-2	0.003948207	1	0
Krtap3-3	0.002862918	1	0
Olfr23	0.007554595	1	0
Olfr390	0.011164599	1	0
Olfr1	-0.003785125	1	0
Ces3a	-0.002422267	1	0
Olfr372	-0.006352494	1	0
Snora33	-0.00101175	1	0
Mir465	-0.002042589	1	0
Mir433	0.001203984	1	0
Mir470	-0.001414397	1	0
Mir484	0.012607508	1	0
Mir490	0.001927501	1	0
Mir127	-0.000457341	1	0
Mir491	0.004491767	1	0
Mir463	0.003654974	1	0

Mir431	0.001466642	1	0
Mir505	0.000631333	1	0
Mir486	0	1	0
Mir466	-0.006426644	1	0
Mir362	-0.000697114	1	0
Mir341	-0.002220673	1	0
Mir455	-0.00110699	1	0
Mir488	0.001619628	1	0
Mir471	0	1	0
Mir495	-0.002363764	1	0
Mir363	0.000336757	1	0
Mir489	-0.00460046	1	0
Mir500	-0.006330134	1	0
Mir497b	-0.007512651	1	0
Mir504	-0.004670106	1	0
Mir199a-2	-0.001695105	1	0
Mir146b	0	1	0
Mir485	-0.005329317	1	0
Mir3071	0.004073423	1	0
Mir328	0.001805503	1	0
Mir434	0.004237685	1	0
Mir496a	-0.000228083	1	0
Mir452	-0.005922038	1	0
Mir532	0.006648595	1	0
Mir483	-0.005104756	1	0
Mir494	-0.002182285	1	0
Mir1a-1	0.011649834	1	0
Olfr894	-0.002373556	1	0
Fbxw22	-0.004835941	1	0
Krtap31-1	0.002921889	1	0
Krtap9-1	-0.001850867	1	0
Amy2a1	-0.005772845	1	0
Olfr59	-0.008218586	1	0
Olfr406	0.005339533	1	0
Olfr43	0.006044024	1	0
Olfr403	0	1	0
Olfr402	0	1	0
Olfr401	-0.006595501	1	0
Olfr389	0.002656968	1	0
Olfr2	0.004644975	1	0
Olfr313	0.001562606	1	0
Vmn2r89	0.012444273	1	0
Vmn2r73	0	1	0
Olfr290	-0.005919442	1	0
Olfr291	0.000323287	1	0
Ccl26	0.006181903	1	0
Wfdc16	-0.006434908	1	0
Wfdc6b	-0.004801593	1	0

Mrgprb3	0.004204663	1	0
Mrgprb4	0.00136025	1	0
Mrgprx1	-0.001687565	1	0
Csn1s1	-0.007306492	1	0
Ugt2b36	-0.001631077	1	0
Psg21	0.001525962	1	0
Psg27	-0.002372597	1	0
Psg25	-0.00156539	1	0
Psg26	-0.001952546	1	0
Sult2a6	-0.001610564	1	0
Sult2a2	-0.005274439	1	0
Vmn1r87	0.01258634	1	0
Vmn1r86	0.002027799	1	0
Vmn1r85	0	1	0
Zscan4f	0.008328663	1	0
Vmn2r34	0.001320632	1	0
Vmn2r42	0.004556906	1	0
Olfr1121	-0.006160595	1	0
Olfr1116	8.00E-06	1	0
Olfr1115	0.002768054	1	0
Olfr1113	0.000982377	1	0
Cryge	0.003932859	1	0
Olfr1100	-0.003191539	1	0
Zfp352	-0.001872156	1	0
Ifna4	0.007143818	1	0
Speer4d	0.004760208	1	0
Olfr356	0.001453305	1	0
Olfr270	0.001114864	1	0
Foxe1	-0.000221044	1	0
Olfr155	0.006414606	1	0
Sdr16c6	-0.003247676	1	0
Olfr806	-0.006638727	1	0
Tas2r140	0	1	0
Tas2r115	-0.001028248	1	0
Tas2r121	-0.012572933	1	0
Defb46	0.012454377	1	0
Serpina1d	0.002480081	1	0
Serpina16	-0.000112973	1	0
Olfr1357	0.003598815	1	0
Fpr-rs6	-0.001016471	1	0
Fpr-rs7	0.001992343	1	0
Vmn1r27	-0.000810576	1	0
Krtap26-1	0.003743196	1	0
Olfr437	-0.001997966	1	0
Vmn1r212	0.00505963	1	0
Vmn1r209	0	1	0
Vmn1r208	-0.009620375	1	0
Olfr172	-0.001539206	1	0

Prss3	-0.004294053	1	0
Olfr263	-0.007285835	1	0
Olfr1477	-0.000130738	1	0
Tex13a	0.004423118	1	0
Cldn34b3	-0.006190716	1	0
Olfr1320	-0.008492969	1	0
Rhox12	0.002714405	1	0
Rhox7a	-0.000102129	1	0
Rhox3h	-0.005352359	1	0
Rhox4e	0.007251152	1	0
Rhox3a	-0.00757475	1	0
Rhox1	0.005515891	1	0
Fthl17e	-0.01195847	1	0
Mroh9	-0.001408347	1	0
Vmn2r121	0.004207108	1	0
Cldn34b1	0	1	0
BC061237	0.002994077	1	0
Fthl17f	0.002043249	1	0
Eddm3b	0.000931307	1	0
Ang6	-0.01621457	1	0
Ear1	-0.002084155	1	0
Ugt3a1	-0.000540982	1	0
Olfr381	0.002028469	1	0
Olfr380	-0.000947113	1	0
Lrcol1	0	1	0
Vmn2r24	0.000967667	1	0
Mir546	-0.004555085	1	0
Samt2	-0.003279062	1	0
Mir540	-0.000320632	1	0
Cpxcr1	-0.001219622	1	0
Fnd3c2	-0.001773262	1	0
Dmrtc1b	0.000791594	1	0
Igkv4-71	-0.000342713	1	0
Atoh1	-0.001536506	1	0
Mageb2	-9.29E-05	1	0
Olfr444	-0.001050015	1	0
Olfr446	0.006109298	1	0
AU016765	-0.000267909	1	0
Esp1	0.004262068	1	0
Smok2b	-0.005165557	1	0
Sh2d1b2	-0.001696658	1	0
AA792892	0.000709773	1	0
Spink11	-0.001084629	1	0
Serpinb3c	-0.000917687	1	0
Serpinb3b	0.000586237	1	0
Crygb	-0.001171609	1	0
Pramel4	-0.002092969	1	0
Defb18	-0.001520709	1	0

Olfr1330	0.001511813	1	0
Olfr1331	0.001247948	1	0
Ifna12	-0.006258433	1	0
Mup14	-0.004581671	1	0
Mup11	0.003329444	1	0
Mup-ps12	-0.001041668	1	0
Mup-ps2	0.004517007	1	0
Mup7	0.004093466	1	0
Olfr472	0.010157658	1	0
Rbmxl2	-0.004417128	1	0
Olfr716	0.011389794	1	0
Olfr17	0.002974448	1	0
Olfr713	0.003276645	1	0
Olfr704	-0.003238905	1	0
Olfr703	0.003624888	1	0
Olfr678	0.003452567	1	0
Olfr676	0.001194235	1	0
Olfr669	-0.014552001	1	0
Olfr665	-0.003393709	1	0
Olfr661	-0.004509379	1	0
Olfr659	-0.002111508	1	0
Olfr657	0.000822039	1	0
Olfr656	9.29E-05	1	0
Olfr653	0.005286291	1	0
Olfr646	-0.001382061	1	0
Olfr641	0.001579655	1	0
Olfr633	0.000490505	1	0
Olfr632	-0.005951432	1	0
Olfr619	0.006811733	1	0
Olfr618	0.003003104	1	0
Olfr617	0.003162456	1	0
Olfr615	-0.002507821	1	0
Olfr608	0.002366213	1	0
Olfr606	-0.00265555	1	0
Olfr599	-0.003069798	1	0
Olfr598	0.007027768	1	0
Olfr597	-0.003115829	1	0
Olfr596	0.001541963	1	0
Olfr594	0.002932916	1	0
Olfr593	-0.00179548	1	0
Olfr592	0.000951633	1	0
Olfr584	0.010430309	1	0
Olfr583	0.000429301	1	0
Olfr582	0.003042998	1	0
Olfr576	0.001410522	1	0
Olfr572	0.004597838	1	0
Olfr568	8.99E-05	1	0
Olfr557	0.001817128	1	0

Olfr556	0.001151803	1	0
Olfr555	-0.008155742	1	0
Olfr554	-0.006543714	1	0
Olfr553	0.003042167	1	0
Olfr551	0.008112743	1	0
Olfr549	-0.002324197	1	0
Olfr547	-0.009144368	1	0
Cnbd1	-0.003262742	1	0
Fbxw18	-0.003179509	1	0
Fbxw16	-0.006071305	1	0
Mrgpra9	-0.006433789	1	0
Cmtm2a	0.010170104	1	0
Cyp2a4	0.003323053	1	0
Vmn1r168	0.008184146	1	0
Vmn1r139	0.000644856	1	0
Vmn1r132	-0.006882556	1	0
Gldnos	0.000633233	1	0
Psg23	0.000744824	1	0
Crxos	0.00284735	1	0
Obox5	-0.000160691	1	0
Obox2	0.009321608	1	0
Sult2a3	0.000224413	1	0
Sult2a4	-0.001408964	1	0
Lce3e	-0.000905456	1	0
Defa28	0.000159476	1	0
Smcp	-0.010280631	1	0
Defa29	0.003994547	1	0
Defa5	0.00090003	1	0
Defa3	0.000896579	1	0
Defa31	0.00051681	1	0
Defa22	0.00915828	1	0
Defa30	-0.000290454	1	0
Sprr2a3	0.004562591	1	0
Defa23	0.004358207	1	0
Defa21	-0.001054161	1	0
Defb33	0.004901275	1	0
Ubtfl1	-0.000806366	1	0
Spint5	0.000285875	1	0
Spint3	0.00081636	1	0
Defb28	-0.009551524	1	0
Defb26	0.001765843	1	0
AU015228	-0.00479885	1	0
Spdye4c	-0.002085044	1	0
Cts3	-0.000269093	1	0
Ctsm	0.006106867	1	0
Cyp2c68	-0.006599443	1	0
EU599041	-0.000266202	1	0
Eif4e1b	-0.005687064	1	0

Krtap14	0.004824491	1	0
Olfr1314	-0.008690506	1	0
Olfr1313	0.001364202	1	0
Olfr1312	0.001095891	1	0
Olfr1308	0.001021193	1	0
Olfr1277	0.001124856	1	0
Olfr1275	0.00032392	1	0
Wt1os	-0.001311364	1	0
Olfr201	-0.005848255	1	0
Olfr199	-0.002092903	1	0
Olfr1272	0	1	0
Olfr1271	-0.001981108	1	0
Olfr142	0.006225814	1	0
Olfr1506	-0.022122819	1	0
Olfr1270	0.001044405	1	0
Olfr32	-0.001720523	1	0
Olfr140	4.34E-05	1	0
Olfr1264	-0.000889822	1	0
Olfr48	-0.003967859	1	0
Olfr1254	0.000580243	1	0
Olfr1253	0.000501466	1	0
Olfr1250	0.001487362	1	0
Olfr1249	-0.003296797	1	0
Olfr1247	0.002534928	1	0
Olfr1243	0	1	0
Olfr1242	0.007132211	1	0
Olfr1241	-0.002069419	1	0
Olfr1239	-5.06E-05	1	0
Olfr1234	-0.00113261	1	0
Olfr1233	0.000502235	1	0
Olfr1232	0	1	0
Olfr1231	0.005008613	1	0
Olfr1230	-0.001627826	1	0
Olfr1229	-0.009770017	1	0
Olfr1226	0.003458211	1	0
Olfr1223	0.002063475	1	0
Olfr1222	-0.007796744	1	0
Olfr1221	0.00062392	1	0
Olfr1219	-0.000441832	1	0
Olfr1218	0.003584235	1	0
Olfr1216	-0.000177367	1	0
Olfr1214	-0.000348423	1	0
Olfr1213	-0.004442608	1	0
Olfr1211	0.005185239	1	0
Olfr1209	-0.004208206	1	0
Olfr1208	0.000568647	1	0
Olfr1200	-0.00420166	1	0
Olfr1198	0.007470854	1	0

Olfr1197	-0.004343743	1	0
Olfr1196	0.000407124	1	0
Olfr1181	-0.003476991	1	0
Olfr1179	-0.002018949	1	0
Olfr1173	1.28E-05	1	0
Olfr1170	0.000571976	1	0
Olfr1164	0.004842627	1	0
Olfr1163	-0.001627389	1	0
Olfr1162	0.004701032	1	0
Olfr73	-0.00027812	1	0
Olfr1160	-0.004539238	1	0
Olfr74	-0.005400222	1	0
Olfr1157	0.00302211	1	0
Olfr1156	0.002011956	1	0
Olfr1155	-0.009173204	1	0
Olfr1154	0.007468588	1	0
Olfr1141	-0.00555562	1	0
Olfr1138	0.000281346	1	0
Olfr1137	3.99E-05	1	0
Olfr1136	-0.000577385	1	0
Olfr1135	-0.000491491	1	0
Olfr1133	-0.000571623	1	0
Olfr1111	-0.006147568	1	0
Olfr1110	-0.001924389	1	0
Olfr259	0.003746392	1	0
Olfr1109	-0.000568888	1	0
Olfr1107	0.004020104	1	0
Olfr1106	0.002350288	1	0
Olfr1105	-0.000789316	1	0
Olfr1104	0.009316571	1	0
Olfr1101	4.15E-05	1	0
Olfr1099	0.005418692	1	0
Olfr1098	0.001538439	1	0
Olfr1097	-0.002530625	1	0
Olfr1095	-0.002145269	1	0
Olfr1090	0.00116304	1	0
Olfr1087	-0.002789083	1	0
Olfr1086	0.00074058	1	0
Olfr1085	0.01073854	1	0
Olfr1079	-0.000864788	1	0
Olfr1066	0.001226778	1	0
Olfr1061	-0.010906353	1	0
Olfr1058	-0.001034059	1	0
Olfr1057	-0.00412476	1	0
Olfr1056	0.004563069	1	0
Olfr1055	-0.003429855	1	0
Olfr1054	0.021461144	1	0
Olfr1053	-0.000864804	1	0

Olfr1051	0.001161687	1	0
Olfr1049	-0.002940756	1	0
Olfr1048	0.005159236	1	0
Olfr1047	0.003876993	1	0
Olfr1046	0.00327106	1	0
Olfr1045	-0.011458798	1	0
Olfr52	0.003228394	1	0
Olfr1044	0.004071418	1	0
Olfr1043	-0.008143605	1	0
Olfr1042	-0.002238813	1	0
Olfr1040	0.002518536	1	0
Olfr1039	0.002823696	1	0
Olfr1037	0.003290419	1	0
Olfr1024	0.004512678	1	0
Olfr1019	0.001301593	1	0
Olfr1016	0.002849392	1	0
Olfr1012	-0.001665026	1	0
Olfr1006	0.000343267	1	0
Olfr154	0.000341667	1	0
Olfr1002	-0.007977174	1	0
Olfr1000	-0.003640301	1	0
Olfr995	-0.004688339	1	0
Olfr994	-0.002149965	1	0
Olfr988	-0.00258119	1	0
Olfr987	-0.001118459	1	0
Olfr362	0.003700524	1	0
Olfr361	-0.000497117	1	0
Olfr358	-0.003295413	1	0
Olfr355	-0.018228521	1	0
Olfr353	-0.001494762	1	0
Olfr351	-0.001534064	1	0
Olfr3	0.002592425	1	0
Olfr341	0.002111818	1	0
Hoxc4	-0.007112086	1	0
Urad	-0.006200376	1	0
Cyp3a41a	0.003641861	1	0
Cyp3a41b	-0.000414235	1	0
Krtap4-6	-0.001129788	1	0
Defb43	0.008412386	1	0
Defb48	0.003901029	1	0
Smok3c	-0.005227313	1	0
Mir547	0.002113191	1	0
Mir376c	-0.005839329	1	0
Mir688	-0.002240848	1	0
Mir615	0.002293062	1	0
Mir652	-0.004304037	1	0
Mir705	0.040945021	1	0
Mir669a-2	-0.004143435	1	0

Mir669a-3	0.00634321	1	0
Mir692-3	0.010474383	1	0
Mir20b	0.001547458	1	0
Mir710	0	1	0
Mir598	0	1	0
Mir450b	0	1	0
Mir707	0	1	0
Mir541	0	1	0
Mir704	0	1	0
Mir92-1	0	1	0
Mir539	0	1	0
Mir690	0	1	0
Mir681	0	1	0
Mir223	0	1	0
Mir592	0	1	0
Mir678	0	1	0
Mir669c	0	1	0
Mir698	0	1	0
Mir551b	0	1	0
Mir687	0	1	0
Mir503	0	1	0
Mir700	0	1	0
Mir669b	0	1	0
Mir718	0	1	0
Mir686	0	1	0
Mir542	0	1	0
Mir693	0	1	0
Mir711	0	1	0
Mir708	0	1	0
Mir709	0	1	0
Mir679	0	1	0
Mir449c	0	1	0
Mir694	0	1	0
Mir701	0	1	0
Mir680-1	0	1	0
Mir717	0	1	0
Mir695	0	1	0
Mir706	0	1	0
Mir692-1	0	1	0
Mir487b	0	1	0
Mir297b	0	1	0
Mir682	0	1	0
Mir702	0	1	0
Mir543	0	1	0
Mir501	0	1	0
Mir302b	0	1	0
Mir680-3	0	1	0
Mir92b	0	1	0

Mir19b-1	0	1	0
Mir374b	0	1	0
Mir719	0	1	0
Mir697	0	1	0
Mir666	0	1	0
Mir675	0	1	0
Mir672	0	1	0
Mir301b	0	1	0
Mir147	0	1	0
Mir499	0	1	0
Mir665	0	1	0
Mir343	0	1	0
Mir673	0	1	0
Mir216b	0	1	0
Mir599	0	1	0
Mir670	0	1	0
Mir181d	0	1	0
Mir653	0	1	0
Mir182	0	1	0
Mir568	0	1	0
Mir674	0	1	0
Mir190a	0	1	0
Mir671	0	1	0
Mir667	0	1	0
Mir676	0	1	0

val)
